# Deaminative C(*sp* ^3^)—C(*sp* ^3^) Cross-Coupling of Benzylamines with Alcohols and Carboxylic Acids via Radical Sorting

**DOI:** 10.1021/jacs.6c04676

**Published:** 2026-05-07

**Authors:** William Y. Zhao, Noriyuki Takanashi, Albert Cabré, Joseph R. Martinelli, David W. C. MacMillan

**Affiliations:** † Merck Center for Catalysis at Princeton University, Princeton, New Jersey 08544, United States; ‡ Centro de Investigación, Lilly S.A., Madrid 28108, Spain; § Eli Lilly and Company, Lilly Institute of Genetic Medicine, Lilly Seaport Innovation Center, Boston, Massachusetts 02210, United States

## Abstract

Benzylamines represent a promising yet underexplored class of building blocks in C­(sp^3^)—C­(sp^3^) bond formation. The scarcity of deaminative coupling methods of benzylamines with other ubiquitous native functionalities, such as alcohols and carboxylic acids, has constrained their synthetic utility. Herein, we report two metallaphotoredox C­(sp^3^)—C­(sp^3^) cross-coupling reactions that merge structurally diverse benzylamines with carboxylic acids and 3° alcohols. In both transformations, the key bond-forming step proceeds via a Fe-porphyrin-catalyzed S_H_2 radical sorting pathway. Both reactions exhibit broad substrate scope, with their synthetic utility further highlighted in the synthesis of complex, biologically active compounds, semisaturated aromatic scaffolds, and enantiopure pyrrolidine derivatives.

## Graphical abstract

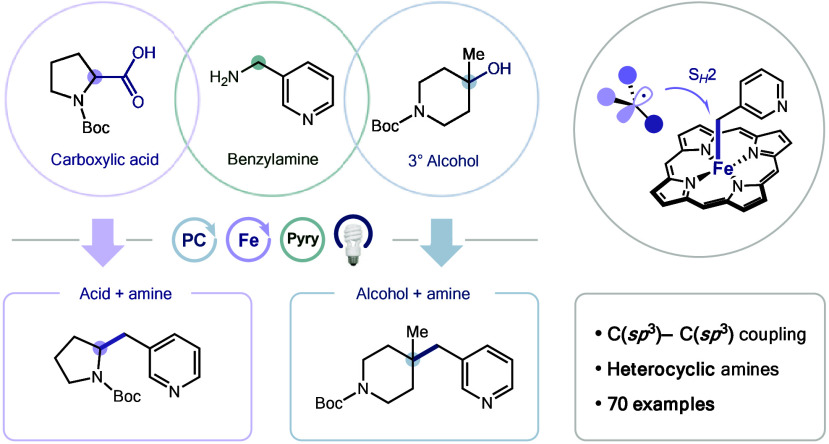

Alcohols, 1° alkylamines, and carboxylic acids are among the most common functional groups in organic compounds.^1,2^ In particular, benzylamines offer access to unique chemical space, because of their commercial availability and synthetic accessibility. Heterocyclic benzylamines are especially attractive, because they exhibit superior bench stability, relative to their benzyl bromide counterparts, which are prone to polymerization and other decomposition pathways.^3^ In addition, heterocyclic benzylamines are three to five times more abundant than the corresponding benzyl bromides^4^ and are readily accessible from a broad portfolio of precursors (Figure 1A).^5–7^

**Figure 1:**
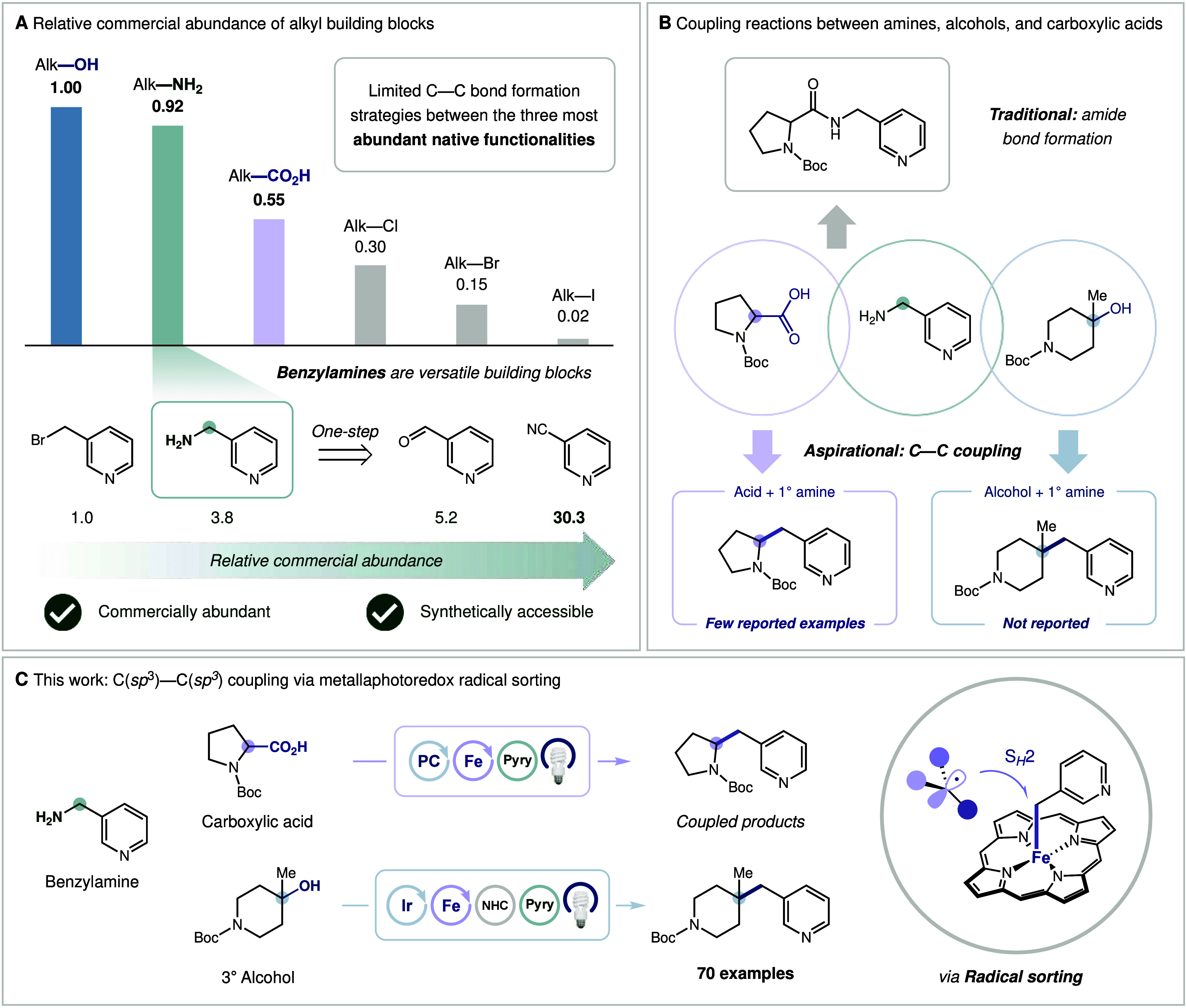
Cross-coupling of 1° alkyl amines with carboxylic acids and alcohols.

Despite their potential synthetic value, methods for direct C­(sp^3^)—C­(sp^3^) coupling of amines with other ubiquitous native functionalities, such as alcohols or carboxylic acids, remain underdeveloped. While amines and carboxylic acids are routinely merged to form amide bonds,^8,9^ particularly in the context of peptide synthesis,^10,11^ few direct C­(sp^3^)—C­(sp^3^) cross-coupling reactions between these two substrate classes have been reported.^12,13^ Direct C­(sp^3^)—C­(sp^3^) coupling of amines and alcohols remains similarly elusive (Figure 1B).

Recent developments in synthetic organic chemistry have enabled the use of native functionalities in lieu of organometallic species in C—C bond formation. In particular, first-row transition metals are capable of catalyzing the direct C—C coupling of a wide range of precursors under mild conditions with excellent functional group tolerance.^14^ Typically, the key organometallic complex is formed via capture of an alkyl radical species—generated directly from one or both substrates—by the metal center, followed by reductive elimination to deliver the coupled product.^15^ Within this framework, 1° alkylamine substrates are usually preactivated by conversion to 2,4,6-triphenylpyridinium fluoroborate salts,^16–18^ termed Katritzky salts. These bench-stable salts are readily prepared via condensation of free amines with commercially available 2,4,6-triphenylpyrylium fluoroborate,^19^ followed by simple filtration, trituration, and chromatography if necessary.^16^ First developed by A. R. Katritzky as pseudohalide-like electrophiles for polar substitution reactions,^20^ 2,4,6-triphenylpyridinium salts have since emerged as practical alkyl radical precursors.^17^ Groundbreaking work by the Watson group showcased the synthetic potential of the Katritzky salts in cross-coupling reactions with a variety of coupling partners.^21–25^ More recently, the Cernak group harnessed these salts in the formal C­(sp^3^)—C­(sp^3^) cross-coupling between alkyl carboxylic acids and 1° alkylamines via preactivation of both coupling partners, constituting one of the few reported transformations of this kind.^26^

Inspired by these precedents, we aimed to develop a redox-neutral metallaphotoredox platform for the selective coupling of alkyl radicals derived from amines with either carboxylic acid or alcohol coupling partners. Our group has previously introduced activation strategies that directly convert carboxylic acids or alcohols into radical intermediates capable of participating in efficient C­(sp^3^)—C­(sp^3^) cross-couplings. In these reactions, aliphatic carboxylic acids undergo direct deprotonation–oxidation, followed by transition-metal catalyzed C—C bond formation with a range of coupling partners.^27–30^ Similarly, our deoxygenation strategy, which involves in-situ oxidative activation of alkyl alcohols,^31^ has enabled diverse cross-coupling reactions—most prominently, C­(sp^3^)—C­(sp^3^) bond formation via radical sorting and homolytic substitution (S_H_2).^32–34^ This approach allows selective and efficient cross-coupling between two alkyl radicals, bypassing oxidative addition and transmetalation pathways commonly involved in nickel catalysis that could compromise cross-selectivity.^35^

Among the radical sorting platforms developed to date, iron porphyrins have emerged as particularly promising catalysts for cross-couplings between 1° alkyl or benzyl radicals and 2° or 3° alkyl radicals. First disclosed by our group in 2021,^36^ Fe­(II)-porphyrin-catalyzed C­(sp^3^)—C­(sp^3^) radical cross-couplings have seen numerous applications across a wide range of bond-forming reactions, including asymmetric catalysis.^37^ Recent publications from the Shenvi group further validated the efficacy of this catalytic platform for benzylation reactions.^38,39^ Therefore, Fe­(II)-porphyrins were of great appeal to us as the optimal S_H_2 catalyst for our proposed transformations.

Herein, we report two metallaphotoredox-catalyzed deaminative C­(sp^3^)—C­(sp^3^) cross-coupling reactions that merge benzylamines with carboxylic acids or 3° alcohols via S_H_2-mediated radical sorting enabled by an Fe­(II)-porphyrin catalyst (Figure 1C). Collectively, these transformations provide modular and rapid access to structurally diverse and complex scaffolds, with complementary advantages from a retrosynthetic perspective.

Mechanistically, the reaction systems share several key features: (a) oxidative activation of the carboxylic acid or 3° alcohol substrate, (b) reductive activation of the 1° alkylamine-derived 2,4,6-triphenylpyridinium partner, and (c) subsequent bond formation via S_H_2. A proposed mechanism for the deaminative-decarboxylative coupling reaction is outlined in Figure 2. First, free carboxylic acid **I** is deprotonated to form the corresponding carboxylate. Under blue-light irradiation, the excited-state photocatalyst (*E*_1/2_^red^[PC*/PC^**•** –^] = +1.35 V vs SCE, τ = 5.1 μs) is reductively quenched by the carboxylate,^40^ and the resultant *O*-centered radical undergoes rapid decarboxylation to generate 3° alkyl radical **II**. The reduced-state photocatalyst (*E*_1/2_^red^[PC/PC^**•** –^] = −1.21 V vs SCE)^40^ is then turned over by pyridinium salt **III** (*E*_1/2_^red^ ≈ 0.9 V vs SCE),^41^ generating a dihydropyridyl radical that undergoes facile β-scission to afford benzyl radical **IV** and an inert pyridine byproduct (Ph_3_Py). Benzyl radical **IV** is preferentially captured by Fe­(II)-porphyrin complex **V**, forming Fe­(III)-alkyl complex **VI**, which then engages in homolytic substitution with 3° alkyl radical **III**, forging the desired cross-coupled product and regenerating the ground state Fe­(II) catalyst. Analogously for alcohol substrates, in-situ activation with a benzoxazolium reagent forms an adduct that, upon oxidation by the excited-state photocatalyst, undergoes β-scission to generate the key 3° alkyl radical (Figure S1). Our proposed mechanisms are supported by control experiments, and the presence of a benzyl radical intermediate is substantiated by the observed formation of the corresponding dimerization byproducts (see the Supporting Information (SI)).

**Figure 2:**
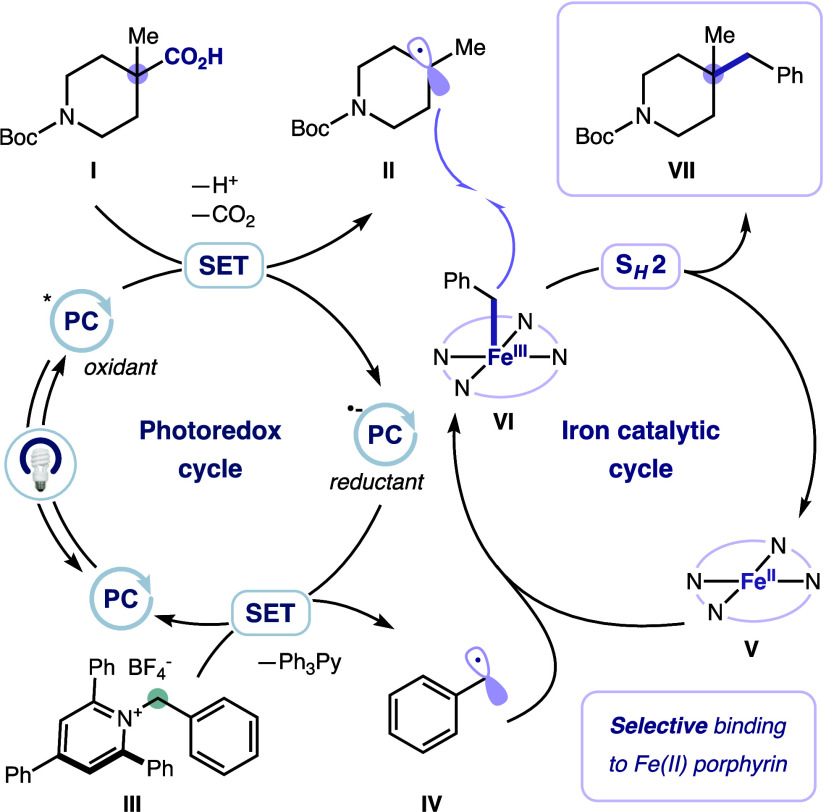
Proposed mechanism for the deaminative-decarboxylative cross-coupling reaction.

After extensive optimization studies, we identified the most appropriate reaction conditions, under which carboxylic acid (0.5 mmol), pyridinium salt (1.5 equiv), potassium carbonate (1.5–2.5 equiv), Fe­(OEP)­Cl (5 mol %), and 4CzIPN (5 mol %) in DMA/*i*-PrOH (1:1, 0.05 M) are irradiated at 450 nm by blue LEDs for 24 h (see the SI for selected optimization details). With optimized conditions in hand, we proceeded to evaluate the scope of this transformation (Table 1). Recognizing the correlation between the electronic environment of the substituted benzyl radical^42^ and the strength of the corresponding Fe—C bond,^43^ we first examined this parameter by varying the *para*-substituent of the benzylamine coupling partner. We were pleased to find that both electron-deficient and electron-rich benzylamines provided the desired products in favorable to excellent yields (**1**–**6**, 58%–84% yield), including base-sensitive ester **1** and sulfonamide **4**. Functional groups prone to oxidative addition (**7**, bromide) or transmetalation (**8**, boronic ester) in traditional transition-metal catalysis were also tolerated, and the presence of *ortho*-substituents had minimal impact on coupling efficiency (**9**, 76% yield; **10**, 66% yield). Protected anilines (**11**) and benzylamines (**12**) similarly proved to be competent coupling partners, providing handles for subsequent orthogonal functionalization.

**Table 1:**
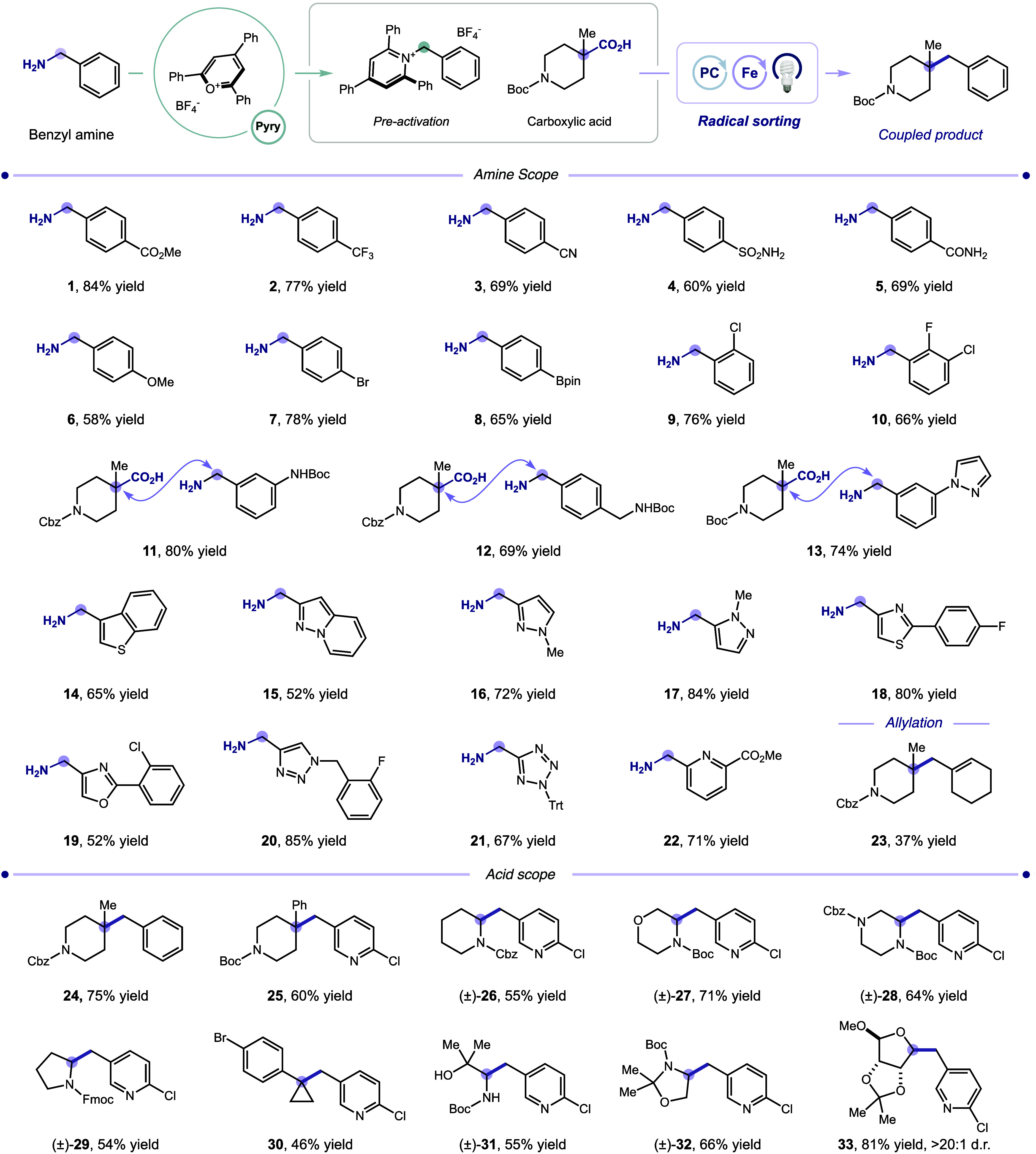
Benzylamine and Carboxylic Acid Scope^a^

a Typically performed with carboxylic acid (0.5 mmol), benzylamine-derived pyridinium salt (1.5 equiv), K_2_CO_3_ (2.5 equiv), Fe­(OEP)Cl (5 mol %), 4CzIPN (5 mol %), DMA/*i*-PrOH (1:1, 0.05 M) under IPR (450 nm) irradiation for 24 h. All yields are isolated unless otherwise noted. See the SI for experimental details.

Five-membered heteroaromatics are of particular interest in medicinal chemistry, because of their ability to modulate pharmacokinetic properties.^44,45^ To validate their compatibility with our conditions, we were pleased to observe that pyrazole-containing benzylamine **13** provided the desired product in 74% yield. Encouraged by this result, we then evaluated the scope of five-membered heterocyclic benzylamines. Promising results were obtained with fused five-six heterocycles (**14**, 65% yield; **15**, 52% yield), as well as a broad range of differentially substituted five-membered heterocycles (**16**–**22**, 52%–84% yield). Notably, highly electron-deficient tetrazole **21** delivered the desired product in 67% yield, thereby enabling a direct one-step conversion of a native carboxylic acid into its corresponding bioisostere.^46^ In addition, this method was expanded to encompass less-activated allylamines, with the coupled product obtained in modest 37% yield (**23**). The lower coupling efficiency is likely due to competing radical polymerization and the slower β-scission of the dihydropyridyl radical intermediate compared to that of benzyl substrates, leading to other deleterious pathways. The latter hypothesis is further supported by the incompatibility of 1° alkyl amines with our system (Scheme S2).

Next, we evaluated the scope of this system with respect to the carboxylic acid component. In the case of α-3° carboxylic acids, varying the α-alkyl substituent from methyl (**24**, 76% yield) to phenyl (**25**, 60% yield) resulted in only a slight deduction in yield. Moreover, we —were delighted to observe that protected α-amino acids **26**–**29** efficiently engaged in the desired transformation, with electron-withdrawing β-heteroatom substituents (**27**, 71% yield, **28**, 64% yield) exerting a minor impact on reaction efficiency. Base-labile amine protecting groups are also compatible with our system, as illustrated by Fmoc-protected l-proline derivative **29** (58% yield). Additionally, a carboxylic acid precursor of an *s*-rich 3° radical underwent coupling (**30**, 46% yield), as did a substrate bearing a free alcohol and significant steric hindrance at the β-position (**31**, 55% yield). Lastly, serine-derived Garner’s acid^47^ **32** and protected-ribose **33** delivered the products in high yields (**32**, 66% yield; **33**, 81% yield), and in the case of **33**, excellent diastereoselectivity (>20:1 d.r.), likely as a result of exceptional facial selectivity in the bond formation step, highlighting the utility of this method in the synthesis of unnatural bioactive building blocks.

All-carbon quaternary centers are highly valuable motifs in drug discovery campaigns due to their role in reinforcing metabolic stability and binding selectivity.^48,49^ Traditionally, the modular construction of these modalities has presented a formidable synthetic challenge.^50–52^ Encouraged by the successful formation of all-carbon quaternary centers from α-3° carboxylic acids, we sought to harness other 3° alkyl radical precursors to further expand the chemical space accessible. In a complementary effort, we evaluated 3° alcohols—which are exceptionally abundant and synthetically accessible—as alternative 3° radical precursors.^53^ With modified reaction conditions, we developed a protocol involving in-situ activation of 3° alcohols using the benzoxazolium activator (“**NHC**”) developed in our group.^31^ To compare the effectiveness of this reaction to its decarboxylative counterpart, we subjected a collection of electronically diverse pyridine-derived benzylamines to these conditions (r-**40**, 71%–73% yield). Other heterocyclic amines, simple benzylamines, and amide protecting groups performed comparably (**41**–**46**, 66%–85% yield).

Having established this system as a capable complement to the decarboxylative reaction, we then examined the scope of 3°alcohols (Table 2). A series of cyclic alcohols with different ring sizes and heteroatoms (**47**–**50**), as well as spirocyclic (**51**–**54**) and fused bicyclic alcohols (**55**), all furnished the coupled product in good to excellent yields (50%–82% yield). Remarkably, this method overcomes significant steric hindrance (**53**) and geometric strain (**55**) in selected substrates. Linear alcohols with carbamate-protected amines also underwent efficient coupling (**56**, 67% yield; **57**, 64% yield). Moreover, complex heterocycle- and alkene-containing alcohols (**60**), together with moieties prone to oxidative addition (**58**) and transmetalation (**59**), are compatible with this system thanks to the mechanistic nature of the proposed S_H_2 pathway. Alcohols bearing sterically demanding α-substituents may also serve as effective, modular building blocks, facilitating rapid buildup of structural complexity (**61**, 50% yield). While our platform proved capable of quaternary center formation from a broad range of 3° alcohols, 1° and 2° alcohols remain unsuitable substrates, likely due to the reduced nucleophilicity of the corresponding alkyl radicals.

**Table 2:**
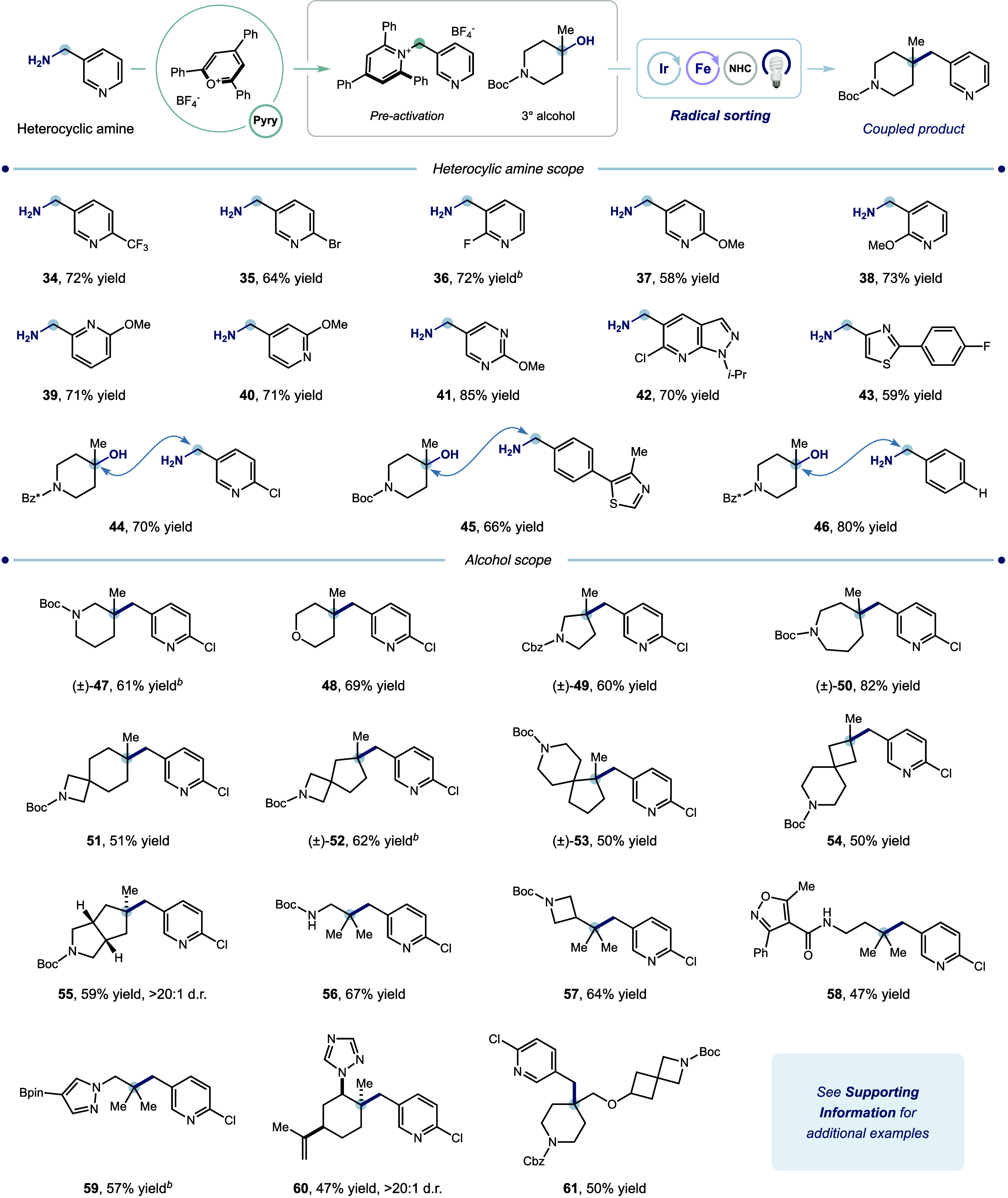
Construction of Quaternary Centers from 3° Alcohols^a^

a Typically performed with alcohol (2.0 equiv., 1.0 mmol), NHC (2.2 equiv) and pyridine (2.15 equiv) in PhCF_3_ with stirring at −25 to 0 °C over 2 h. Then, pyridinium salt (0.5 mmol), Ir­[dF­(CF_3_)­ppy]_2_(dtbbpy)­PF_6_ (1.5 mol %), Fe­(OEP)Cl (2.5 mol %), and KOPiv (4.0 equiv) in acetone/*i*-PrOH (1:1, 0.05 M) are irradiated for 2 h with 450 nm LEDs. All yields are isolated unless otherwise noted.

b Assay yield reported due to challenging isolations. See the SI for experimental details. Bz* = 3,5-di-*tert*-butylbenzoyl; **NHC** = 5,7-di-*tert*-butyl-3-(4-(trifluoromethyl)­phenyl)­benzo­[*d*]­oxazol-3-ium tetrafluoroborate.

Finally, we explored the synthetic applications of our platforms. A selection of pharmaceuticals and biologically active molecules (**62**–**65**, 38%–73% yield) were successfully engaged in decarboxylative coupling, highlighting the amenability of this method to functionally and skeletally complex scaffolds (Table 3). Moreover, in a key extension of our recently introduced “couple-close” platform, we incorporated this novel cross-coupling as a first “couple” step en route to semisaturated aromatic scaffolds (**66**–**68**).^54^ Finally, we explored the potential utility of this reaction in the modular, one-step asymmetric synthesis of 3-hydroxypyrrolidines, a class of medicinally valuable motifs traditionally prepared de novo.^55^ Functionalized 3-hydroxypyrrolidine **69** can be now be accessed with our method in one step in excellent yield and diastereoselectivity, with subsequent deoxygenation affording enantioenriched pyrrolidine derivative **70**.

**Table 3:**
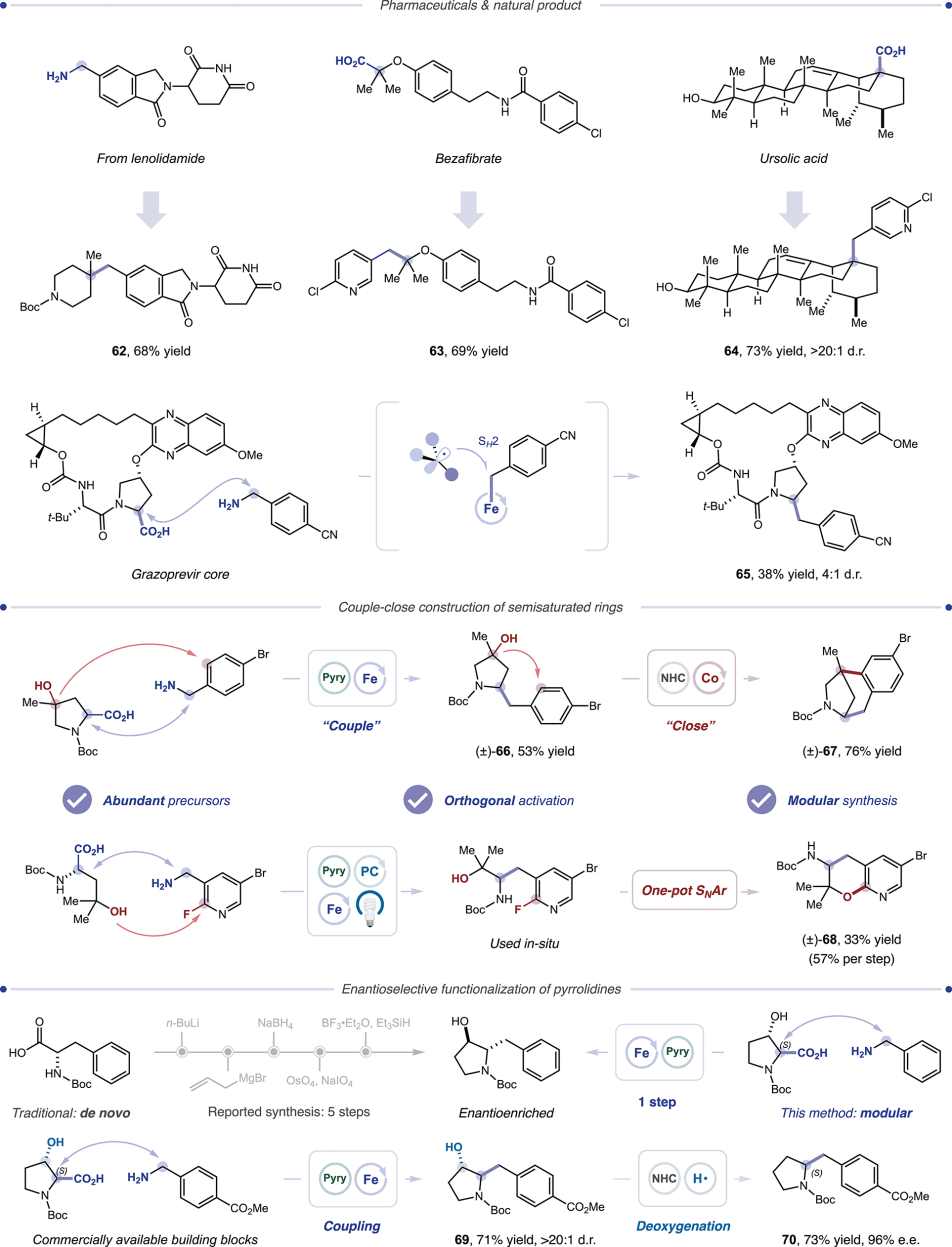
Complex Scaffolds and Synthetic Applications: Deaminative—Decarboxylative Cross-coupling^a^

a All yields isolated. See the SI for experimental details.

In conclusion, we report herein the metallaphotoredox C­(sp^3^)—C­(sp^3^) cross-coupling of benzylamines with carboxylic acids and, for the first time, 3° alcohols. Mechanistically, both reactions achieve radical sorting through a Fe-porphyrin-catalyzed S_H_2 pathway. The synthetic potential of these reactions is highlighted by their performance in complex biologically active molecules and by their application to couple-close sequences and asymmetric synthesis. Given the prevalence of these native functionalities and the value of the resulting scaffolds, we anticipate this method will be of great interest to the synthetic community.

## Supplementary Material



## Notes

Additional experimental details, materials, and methods, including characterization data and NMR spectra of all compounds (PDF)
