# Supplementary material for: Deaminative C(sp 3)—C(sp 3) Cross-Coupling of Benzylamines with Alcohols and Carboxylic Acids via Radical Sorting
Source: J Am Chem Soc. 2026 May 7;148(19):19453–60. doi: 10.1021/jacs.6c04676 (PMC13195648; doi:10.1021/jacs.6c04676)
Supplement: Supplementary file 1 [file ja6c04676_si_001.pdf]

Supporting Information

Deaminative C(sp^3)—C(sp^3) Cross-Coupling of Benzylamines with Alcohols and Carboxylic Acids via Radical Sorting

William Y. Zhao[1†], Noriyuki Takanashi[1†], Albert Cabré[2], Joseph R. Martinelli[3], and David W. C. MacMillan[1]*

[1]Merck Center for Catalysis at Princeton University, Princeton, New Jersey, 08544, United States

[2]Centro de Investigación Lilly S.A., Madrid 28108, Spain

[3]Eli Lilly and Company, Lilly Institute of Genetic Medicine, Lilly Seaport Innovation Center, Boston, Massachusetts 02210, United States

*Corresponding author. Email: dmacmill@princeton.edu

Table of Contents

1. General Information **S3**

2. Proposed Mechanism of Deaminative—deoxygenative C(*sp*^{3})—C(*sp*^{3}) Cross-Coupling **S5**

3. Reaction Optimization and Control Experiments **S7**

4. Additional Examples and Limitations **S15**

5. Synthesis and Characterization of Alcohol Substrates **S20**

6. Synthesis and Characterization of Pyridinium Salts **S24**

7. General Procedures for Deaminative C(*sp*^{3})—C(*sp*^{3}) Cross-Couplings **S60**

8. Isolation and Characterization of Cross-coupled Products **S63**

9. NMR Spectral Data **S163**

10. Bibliography **S304**

1. General Information

Commercial reagents were used without prior purification unless otherwise indicated. All solvents were purchased in their anhydrous form or dried over activated 4Å molecular sieves. Alcohol activation reagent (**NHC**, 5,7-di-*tert*-butyl-3-(4-(trifluoromethyl)phenyl)benzo[*d*]oxazol-3-ium tetrafluoroborate), was prepared according to literature procedures.[1] Recrystallized **NHC** (from 10:1 ethyl acetate/acetonitrile) consistently contains residual ethyl acetate (~10% wt). Filtrations of heterogeneous mixtures from alcohol activation were performed using ChemRus 20 mL disposable filters. Organic solutions were concentrated under reduced pressure on a Büchi rotary evaporator using a temperature-controlled water bath.

Chromatographic purification of products was performed on an automated Teledyne ISCO CombiFlash® NextGen 300+ system using RediSep Rf Gold® Silica Gel Disposable Flash Columns (20–40 microns). Reverse phase chromatography was performed on an automated Biotage IsoleraTM Spektra System system using Silia*Sep*TM C18 cartridges or on a Teledyne ISCO ACCQPrep® HP150 system using Waters XBridge BEH C18 OBD Prep Column (30 mm´ 150 mm, 130 Å, 5 µm) with 0.1% NH_4OH or 0.1% formic acid buffered water and acetonitrile solutions. Thin-layer chromatography (TLC) was performed on Silicycle 0.25 mm or Supelco 0.20 mm silica gel F-254 plates. Visualization of the developed chromatogram was performed by fluorescence quenching, $KMnO_4$ stain, *p*-anisaldehyde, or ninhydrin stains.

^{1}H and ^{13}C NMR spectra were recorded on a Bruker Avance III NMR 500 MHz instrument and are internally referenced to the residual proteo-solvent signals (7.26 ppm and 77.16 ppm, respectively, for $CDCl_3$). ^{19}F NMR spectra were recorded on a Bruker NanoBay Avance III HD

[1] Dong, Z.; MacMillan, D.W.C. Metallaphotoredox-enabled deoxygenative arylation of alcohols. *Nature* **2021**, *598*, 451–456. DOI: 10.1038/s41586-021-03920-6.

NMR 400 MHz and are reported unreferenced. Data for 1H and 19F NMR are reported as follows: chemical shift (d ppm), multiplicity (s = singlet, d = doublet, t = triplet, q = quartet, p = pentet, h = hextet, hept = heptet, m = multiplet, b = broad), coupling constant (Hz), and integration. Data for ^{13}C NMR are reported in terms of chemical shift; multiplicity and coupling constants are included only in the case of coupling with ^{19}F nuclei.

Ultra-Performance liquid chromatography (UPLC) analysis was performed on an Agilent 1200 or Agilent 1290 Infinity II LC system using mesitylene or 1,3,5-trimethoxybenzene as the preferred internal standard. Chiral HPLC (high performance liquid chromatography) was performed on an Agilent 1260 Infinity system using chiral columns as noted. Infrared (IR) spectroscopy was performed on a Thermo Nicolet 6700 FTIR spectrometer with diamond Smart Orbit ATR accessory, and spectra are reported in wavenumbers (cm^{-1}). High resolution mass spectra (HRMS) were obtained from the Princeton University Mass Spectral Facility on Agilent 6220 ESI-TOF LC/MS or Agilent 7200 GC-QTOF systems.

2. Proposed Mechanism of Deaminative—deoxygenative C(sp^3)—C(sp^3) Cross-Coupling

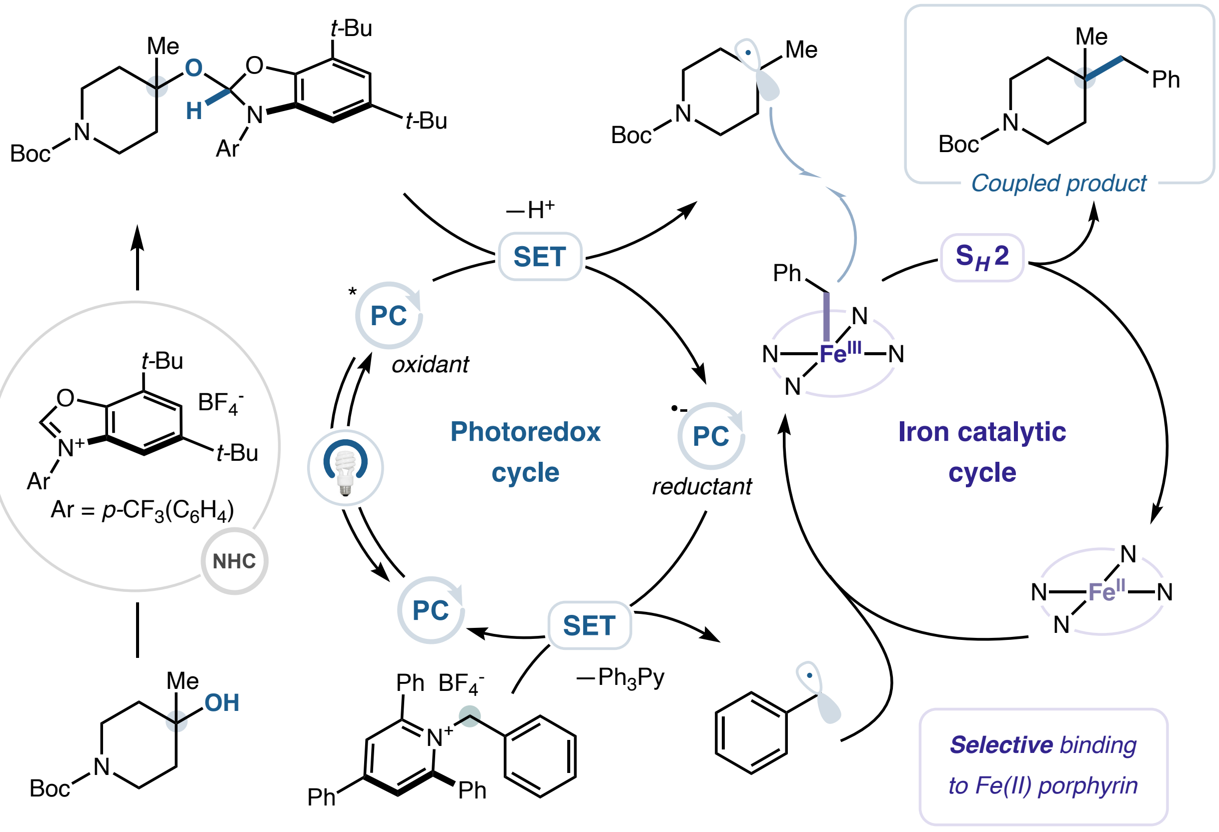

Figure S1. Proposed mechanism of deaminative—deoxygenative C(sp^3)—C(sp^3) cross-coupling.

Discussion on proposed mechanism. There are reported examples in the literature of an S_N2-type oxidative addition pathway involving a reduced-state Fe(I) porphyrin species and an alkyl electrophile that leads to the formation of the key Fe(III)-alkyl complex.[2] However, we propose that our reactions proceed through a benzyl radical intermediate via direct single-electron reduction of the pyridinium salt, followed by the benzyl radical's favourable binding onto Fe(II). In theory, the much higher concentration of the pyridinium salt in solution compared to the Fe(II)-porphyrin catalyst (40:1) and low reduction potential of the former should render the selective formation of a benzyl radical probable. This hypothesis is further supported by the benzylic homo-dimerization product typically observed in our reactions (**Scheme S1**).

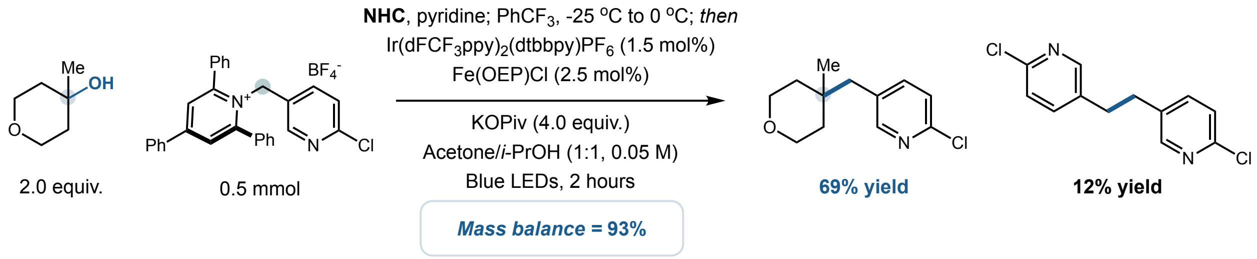

Scheme S1. Reaction profile of a typical deaminative—deoxygenative C(sp^3)—C(sp^3) cross-coupling.

[2] Pace A.L.; Xu, F.; Liu W.; Lavagnino, M.N.; MacMillan, D.W.C. *J. Am. Chem Soc.* **2024** *146*, 32925-32932. DOI: 10.1021/jacs.4c14942.

3. Reaction Optimization and Control Experiments

Deaminative—Decarboxylative cross-coupling

Catalyst stock solution. To an oven-dried 20-mL vial was added 4CzIPN (5.0 mol%), Fe(OEP)Cl (5.0 mol%). Anhydrous DCM (0.005 M) was then added to form a homogenous solution. Sonication may be applied to aid dissolution if necessary.

Pyridinium salt stock solution. To an oven-dried 40-mL vial was added benzyl pyridinium salt (1.5 equiv.). Anhydrous DCM (0.15 M) was then added to form a homogenous solution. Sonication may be applied to aid dissolution if necessary.

Carboxylic acid stock solution. To an oven-dried 40-mL vial was added carboxylic acid (1.0 equiv.). The vial was evacuated and backfilled three times with nitrogen. Anhydrous DMA/*i*-PrOH (1:1, 0.05 M) was then added under inert atmosphere. The resulting stock solution was sparged at 0 °C with nitrogen for 10 min.

General Procedure. To an oven-dried 4-mL vial equipped with a cylindrical stir bar was dispensed **catalyst stock solution** (500 μL, 0.005 M, 2.5 μmol) and **pyridinium salt stock solutio**n (500 μL, 0.15 M, 0.075 mmol) via micropipette. The solution mixture was subsequently concentrated via GeneVac (40 ºC, 200 mbar, 1 h). After complete removal of organic solvents, K_2CO_3 (17.3 mg, 0.125 mmol, 2.5 equiv.) was added into the vial, which was then capped and evacuated and backfilled three times with nitrogen. **Carboxylic acid stock solution** (1.0 mL, 0.05 M, 0.05 mmol) was added via syringe under nitrogen, and the vial was sealed with melted parafilm and placed in a PennPhD m1 integrated photoreactor. The reaction was irradiated with 450 nm

blue LEDs (75% light intensity, 5200 rpm fans, 1500 rpm stirring) for 24 hours. The procedure was modified as necessary to screen the desired variables. After irradiation, internal standard acetanilide (50 µL, 0.5 M stock solution in MeCN, 0.025 mmol, 0.5 equiv.) was added. An aliquot (35 µL) of the crude reaction mixture was then removed and analyzed via uHPLC analysis (Waters Acquity BEH C18 1.7 µm column, 3.0 × 50 mm, 5-100% MeCN/H2O with 0.40 mM 1:1 formate/formic acid buffer) monitoring absorption at 195 nm.

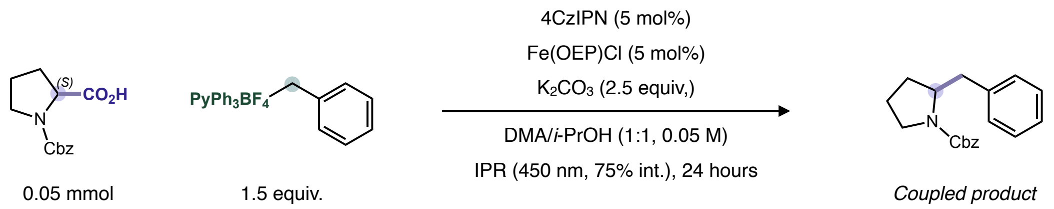

Deviation	***Yield***
none	**50%**
No Fe	**20%**
No PC	**N.R.**
No base	**N.R.**
No light	**N.R.**

Table S1. Control experiments.

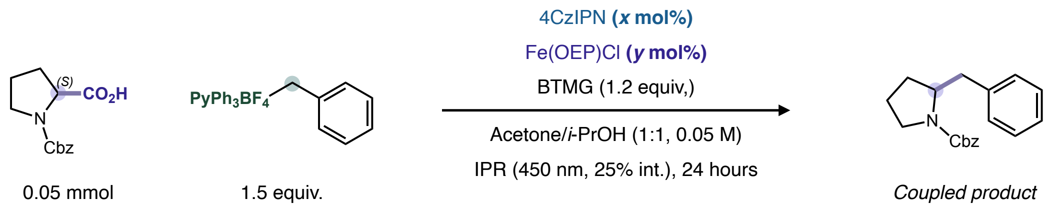

Fe \ PC	0.5 mol%	1.0 mol%	1.5 mol%	2.0 mol%	2.5 mol%
**2.5 mol%**	18% yield	13% yield	21% yield	22% yield	12% yield
**5.0 mol%**	23% yield	18% yield	**26% yield**	18% yield	16% yield
**7.5 mol%**	10% yield	25% yield	24% yield	22% yield	23% yield
**10 mol%**	6% yield	20% yield	19% yield	22% yield	20% yield

Table S2. Photocatalyst vs. Fe(OEP)Cl loading screen.

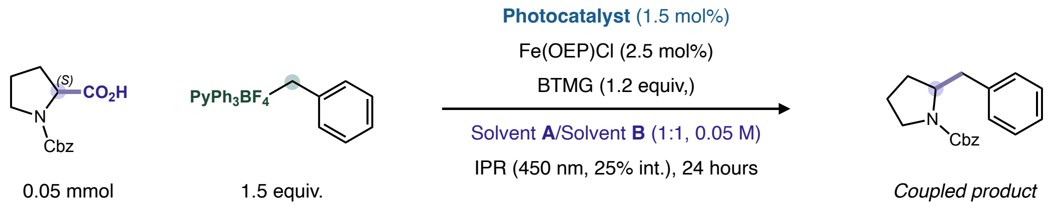

Solvent \ PC	4CzIPN (5 mol%)	$Ir(dFCF_3ppy)_2(dtbbpy)PF_6$	$Ir(dFMeppy)_2(dtbbpy)PF_6$
**MeCN**	13% yield	3% yield	7% yield
**MeCN/acetone**	11% yield	0% yield	0% yield
**MeCN/*i*-PrOH**	**26% yield**	21% yield	14% yield
**MeCN/*t*-AmOH**	16% yield	14% yield	11% yield

Table S3. Evaluation of photocatalyst and reaction solvent.

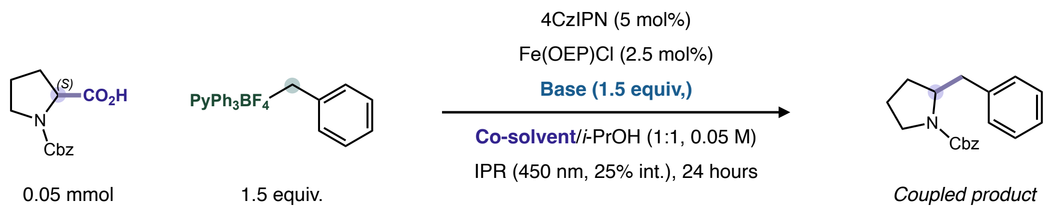

Co-solvent / *Base*	BTMG	Cs_2CO_3	K_2CO_3	K_2HPO_4
**None**	9% yield	26% yield	**49% yield**	0% yield
**Acetone**	15% yield	34% yield	28% yield	0% yield
**DMSO**	9% yield	21% yield	36% yield	0% yield
**DMF**	15% yield	29% yield	**47% yield**	0% yield
**DCE**	20% yield	27% yield	12% yield	0% yield

Table S4. Evaluation of base and reaction co-solvent.

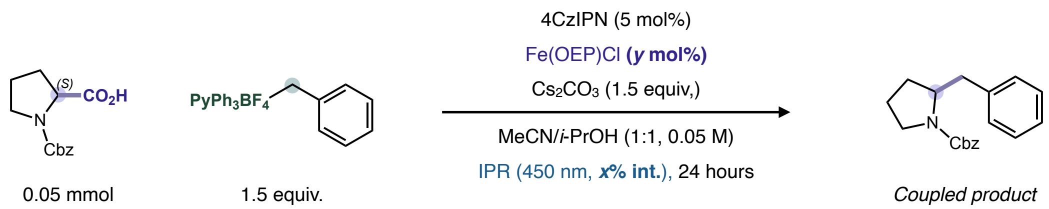

Fe	25% int.	50% int.	75% int.	100% int.
**2.5 mol%**	28% yield	29% yield	33% yield	29% yield
**5.0 mol%**	16% yield	16% yield	**42% yield**	36% yield
**7.5 mol%**	16% yield	17% yield	17% yield	37% yield
**10 mol%**	15% yield	0% yield	–	21% yield

Table S5. Evaluation of Fe loading and photoreactor light intensity.

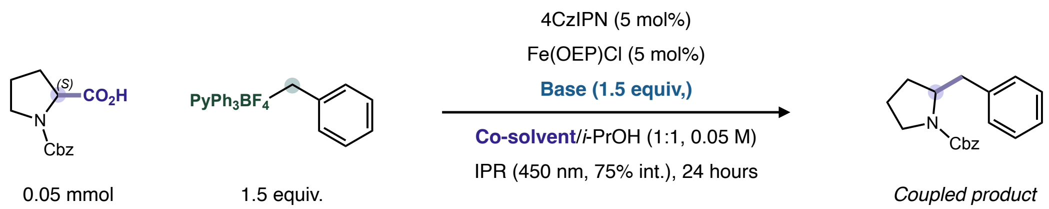

Co-solvent \ Base	Li_2CO_3	Na_2CO_3	K_2CO_3	Cs_2CO_3
DMF	1% yield	42% yield	48% yield	15% yield
DMA	2% yield	47% yield	**51% yield**	35% yield
NMP	1% yield	37% yield	**55% yield**	41% yield
DME	0% yield	13% yield	44% yield	17% yield
DMC	0% yield	9% yield	28% yield	23% yield

Table S6. Further evaluation of carbonate bases and other co-solvents.

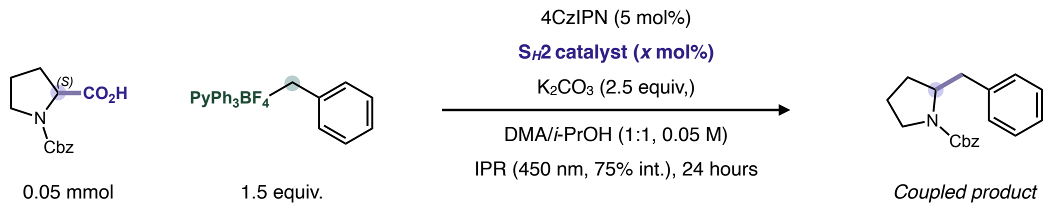

S_H2 Catalyst	Yield
Fe(OEP)Cl (5 mol%)	**50%**
$Ni(acac)_2$ (25 mol%)	**18%**
$Ni(acac)_2$ + KTp* (25 mol%)	**15%**
Fe(TPP)Cl (5 mol%)	**49%**

Table S7. Comparison between Fe(OEP)Cl and other S_H2 catalysts.

Deaminative—Deoxygenative cross-coupling

Catalyst stock solution. To an oven-dried 20-mL vial was added 4CzIPN (5.0 mol%), Fe(OEP)Cl (2.5 mol%). Anhydrous DCM (0.005 M with respect to 4CzIPN) was then added to form a homogenous solution. Sonication may be applied to aid dissolution if necessary.

Pyridinium salt stock solution. To an oven-dried 40-mL vial was added benzyl pyridinium salt (1.0 equiv.). Anhydrous DCM (0.1 M) was then added to form a homogenous solution. Sonication may be applied to aid dissolution if necessary.

Alcohol activation and adduct stock solution. To an oven-dried 40 mL vial equipped with a stir bar was added alcohol (2.0 equiv.) and **NHC** (2.2 equiv.). Next, the vial was evacuated and back-filled three times with N_2, after which $PhCF_3$ (0.1 M) was added via syringe. The vial was then cooled to −25 °C in a dry-ice/*i*-PrOH bath. At this point, pyridine (2.15 equiv.) was added dropwise. The resulting suspension was allowed to warm to 0 °C over two hours while stirring. The resulting orange-red suspension was then syringe-filtered to remove pyridinium salts and unreacted **NHC**, and the dark-red solution was concentrated under reduced pressure by rotary evaporator, followed by vacuum for 5 min. (*It is critical that the activated adduct is placed under vacuum for no longer than 5 minutes to prevent premature decomposition.*) The resulting foamy residue was dissolved in anhydrous acetone /*i*-PrOH (1:1, 0.1 M) and transferred to another oven-dried 40-mL vial charged with KOPiv (4.0 equiv.) under nitrogen. Sonication was applied if necessary to aid dissolution. The stock solution was then sparged at 0 °C with nitrogen for 10 min.

General Procedure. To an oven-dried 4-mL vial equipped with a cylindrical stir bar was dispensed **catalyst stock solution** (500 µL, 0.005 M, 2.5 µmol) and **pyridinium salt stock solution** (500 µL, 0.15 M, 0.05 mmol) via micropipette. The solution mixture was subsequently concentrated via GeneVac (40 ºC, 200 mbar, 1 h). After complete removal of organic solvents, the vial was capped and evacuated and backfilled three times with nitrogen. **Alcohol adduct stock solution** (1.0 mL, 0.1 M, 0.1 mmol) was added via syringe under nitrogen, and the vial was sealed with melted parafilm and placed in a PennPhD m1 integrated photoreactor. The reaction was irradiated with 450 nm blue LEDs (75% light intensity, 5200 rpm fans, 1500 rpm stirring) for 2 hours. The procedure was modified as necessary to screen the desired variables. After irradiation, internal standard mesitylene (50 µL, 0.5 M stock solution in MeCN, 0.025 mmol, 0.5 equiv.) was added. An aliquot (35 µL) of the crude reaction mixture was then removed and analyzed via uHPLC analysis (Waters Acquity BEH C18 1.7 µm column, 3.0 × 50 mm, 5-100% MeCN/H2O with 0.40 mM 1:1 formate/formic acid buffer) monitoring absorption at 220 nm.

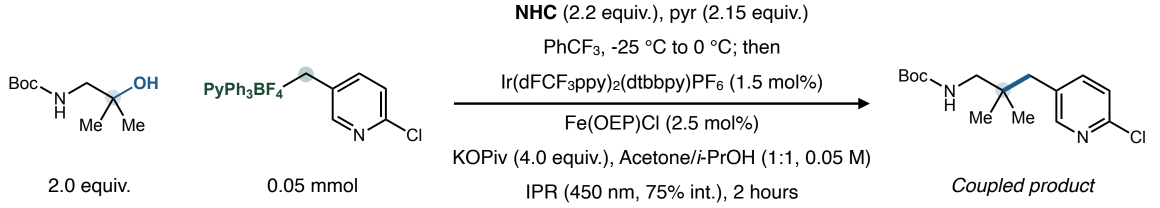

Deviation	Yield
none	67%
No Fe	19%
No PC	N.R.
No base	N.R.
No light	N.R.

Table S8. Control experiments.

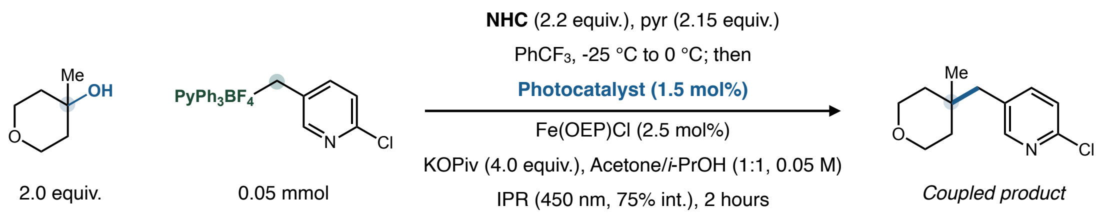

Photocatalyst	*Yield*
$Ir(dFCF_3ppy)_2(dtbbpy)PF_6$	**64%**
4CzIPN (5 mol%)	**59%**
$Ir(ppy)_2(dtbbpy)PF_6$	**59%**
$Ir(dFppy)_3$	**62%**
$Ru(bpy)_3(PF_6)_2$	**46%**

Table S9. Effect of photocatalyst.

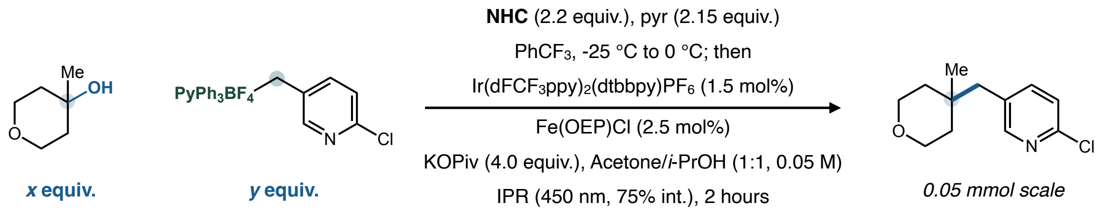

Stoichiometry (x:y)	*Yield*
2.0 : 1.0	**64%**
1.5 : 1.0	**70%**
1.0 : 1.0	**66%**
1.0 : 1.5	**63%**
1.0 : 2.0	**59%**

Table S10. Effect of substrate stoichiometry.

4. Additional Examples and Limitations

<u>Deaminative—Decarboxylative cross-coupling</u>

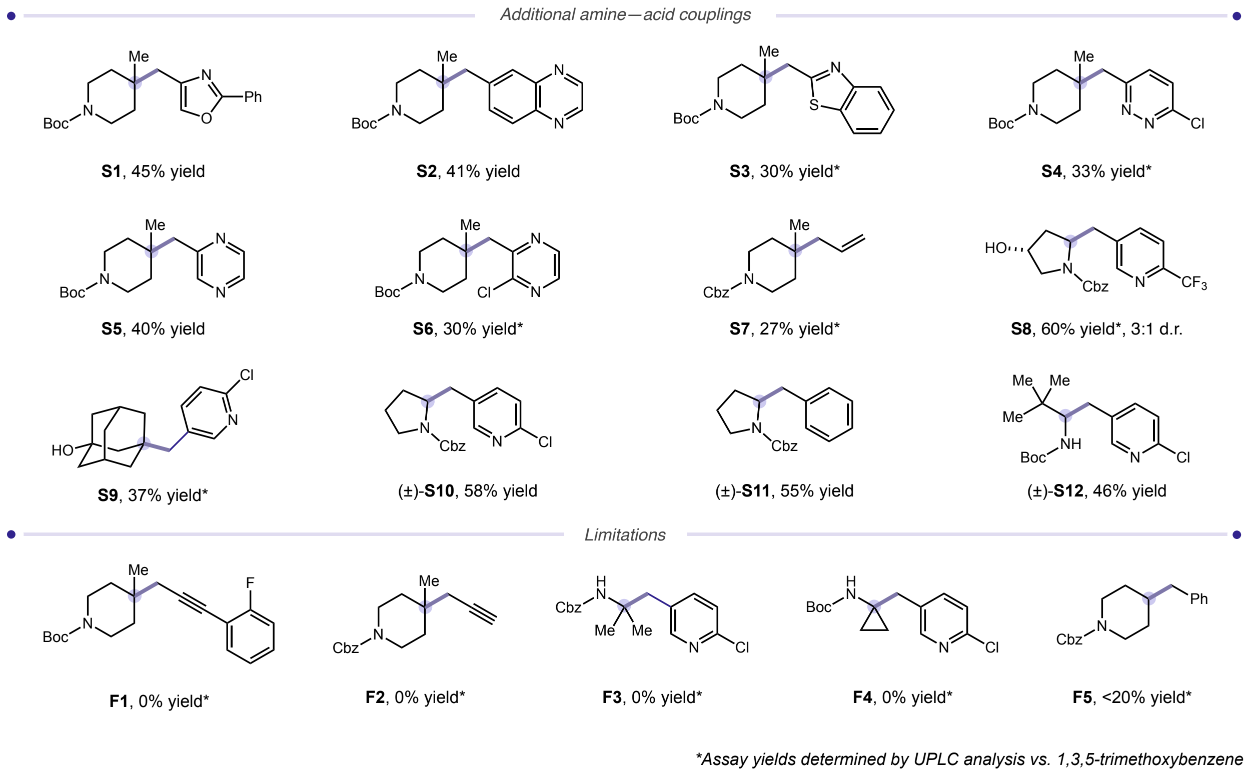

Table S11. Additional deaminative—decarboxylative cross-couplings and limitations.

<u>General remarks:</u>

- Coupling efficiency tends to decrease with increasing Lewis basicity of the heteroaryl nitrogen, likely due to enhanced coordinative deactivation of the iron catalyst.
- In the case of **S3**, significant formation of the alkyl-H side product derived from the pyridinium salt was observed.
- The reduced yields observed in **S4**, and **S6** might be attributed to further homolytic aromatic substitution between the product and a 3° radical generated from the carboxylic acid starting material.

- Neither the desired product nor self-coupling products were detected despite full consumption of both starting materials in the cases of **F1** and **F2**.
- Decomposition was observed when 3° α-amino acid **F3** was used, while no decarboxylation was observed with *s*-rich radical precursor **F4**.
- Compromised yields are obtained in our attempt to engage 2° aliphatic acids in cross-coupling (**F5**). This is likely due to the reduced nucleophilicity of the resultant alkyl radical in relation to 2°α-amino radicals.

Deaminative—Deoxygenative cross-coupling

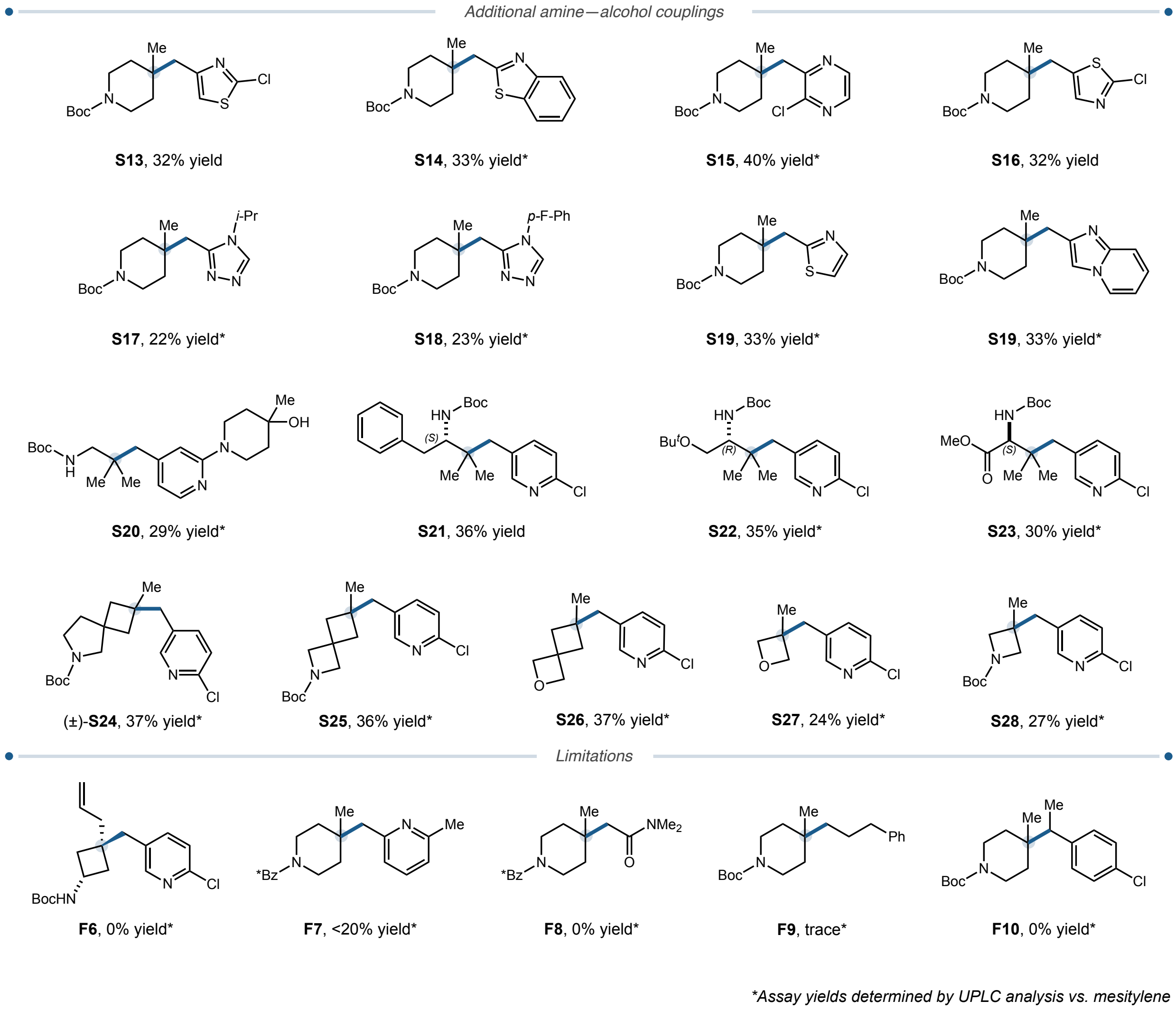

Table S12. Additional deaminative—deoxygenative cross-couplings and limitations.

General remarks:

- Consistent with our hypothesis in the case of decarboxylative couplings, a negative correlation between the nucleophilicity of the alkyl radical derived from the alcohol partner and coupling efficiency was observed. The electronic effect of β-substituents is

demonstrated by **S21**, **S22**, and **S23**, whereas the effect of steric strain on nucleophilicity is demonstrated by **S24-28**.

- Similarly, the aforementioned correlation between heterocycle basicity and coupling efficiency is still evident. This is further supported by the formation of **F7** in only near-background yields under standard conditions.
- In the case of **S14**, significant formation of the alkyl-H side product derived from the pyridinium salt was observed.
- The reduced yields observed in **S15** and **S16** might be attributed to further homolytic aromatic substitution between the product and a 3° radical generated from the carboxylic acid starting material.
- Allylic 3° alcohols (**F6**) proved incompatible with our system, likely due to rapid 5-exo cyclization of the stabilized radical formed after oxidation—deprotonation and subsequent spin-centre shift.[3]
- Attempts in engaging glycine derivative **F8** in cross-coupling were unsuccessful, likely due to the electrophilic nature of the α-carbonyl radical.
- Attempts in 3°-2° cross-couplings (**F10**) were unsuccessful.
- Unactivated α-1° amines (**F9**) were incompatible with our current system due to the slow rate of β-scission after reduction of the pyridinium salt, resulting in radical addition into the dihydropyridyl radical (**Scheme S2**).[4]

[3] Dong, Z.; MacMillan, D.W.C. Metallaphotoredox-enabled deoxygenative arylation of alcohols. *Nature* **2021**, *598*, 451–456. DOI: 10.1038/s41586-021-03920-6.
[4] Liao, J; Basch, C.H.; Hoerrner, M.E.; Talley, M.R.; Boscoe, B.P.; Tucker, J.W.; Garnsey, M.R.; Watson, M.P. *Org. Lett.* **2019** *21*, 2941-2946. DOI: 10.1021/acs.orglett.9b01014.

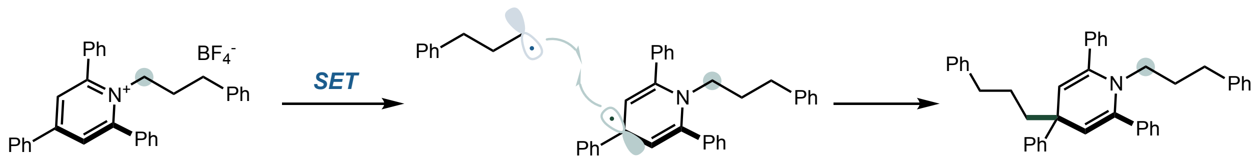

Scheme S2. Formation of the undesired dihydropyridine product.

5. Synthesis and Characterization of Alcohol Substrates

(±)-*tert*-butyl 1-hydroxy-1-methyl-8-azaspiro[4.5]decane-8-carboxylate

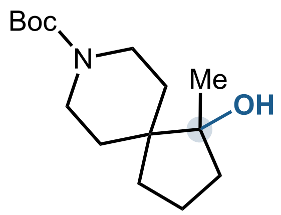

The title compound was prepared according to a modified literature procedure.[5] In an oven-dried 100-mL round-bottom flask equipped with a septum and a magnetic stirring bar, *tert*-butyl 4-oxo-8-azaspiro[4.5]decane-8-carboxylate (1.27 g, 5.0 mmol, 1.0 equiv.) was dissolved in THF (15.0 mL), and the flask was evacuated and backfilled three times with N_2. $LaCl_3\cdot 2LiCl$ (0.6 M in THF, 8.33 mL, 5.0 mmol, 1.0 equiv.) was added via syringe under N_2, and the resulting mixture was stirred for 1 h at room temperature. The reaction was then cooled to 0 °C in an ice bath, and methyllithium (3.2 M in THF, 1.6 mL, 1.0 equiv.) was added dropwise. The resulting mixture was allowed to stir at the same temperature for 1 h, after which saturated aqueous NH_4Cl (25 mL) was slowly added to quench the reaction. The biphasic mixture was poured into a separatory funnel, and the layers were separated. The aqueous layer was extracted with EtOAc (3 × 50 mL), and the combined extracts were washed with brine (3 × 50 mL), dried over Na_2SO_4, and concentrated under reduced pressure. The crude residue was purified by automated flash column chromatography (50-g silica column, 0-40% EtOAc/DCM) to afford the desired product as a colourless oil (1.12 g, 83% yield).

[5] Krasovskiy, A.; Kopp, F.; Knochel, P.; Soluble Lanthanide Salts ($LnCl_3\cdot 2\ LiCl$) for the Improved Addition of Organomagnesium Reagents to Carbonyl Compounds. *Angew. Chem. Intl. Ed.* **2006** *45*, 497-500. DOI: 10.1002/anie.200502485.

^{1}H NMR (500 MHz, $CDCl_3$) δ 4.15 – 3.85 (m, 2H), 2.94 – 2.52 (m, 2H), 1.90 – 1.56 (m, 6H), 1.21 – 1.53 (m, 13H), 1.14 (s, 4H).

^{13}C NMR (126 MHz, $CDCl_3$) δ 155.03, 82.64, 79.38, 46.55, 41.65, 40.60, 38.71, 31.30, 30.56, 29.90, 28.59, 22.22, 18.99.

IR (film) ν_{max} 3465, 2959, 2869, 1669, 1423, 1365, 1243, 1154, 1073, 934, 864, 769 cm^{-1}.

HRMS (ESI-TOF) *m/z* calculated for $C_{15}H_{27}NO_3Na^+$ ($[M+Na]^+$) 292.1883, found 292.1882.

***N*-(3-hydroxy-3-methylbutyl)-5-methyl-3-phenylisoxazole-4-carboxamide**

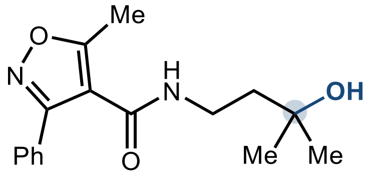

In an oven-dried 50-mL round-bottom flask equipped with a septum and a magnetic stirring bar, 5-methyl-3-phenylisoxazole-4-carboxylic acid (856 mg, 4.21 mmol, 1.0 equiv.) and HBTU (2.08 g, 5.48 mmol, 1.3 equiv.) were dissolved in DMF (10.0 mL). DIPEA (1.47 mL, 8.42 mmol, 2.0 equiv.) was added via micropipette, and the resulting mixture was stirred at room temperature for 10 min. 4-amino-2-methylbutan-2-ol (478 mg, 4.63 mmol, 1.3 equiv., dissolved in 5 mL of DMF) was added via syringe, and the resulting mixture was stirred at the same temperature overnight. 1M aqueous HCl (20 mL) was slowly added to the reaction mixture. The biphasic mixture was poured into a separatory funnel, and the layers were separated. The aqueous layer was extracted with EtOAc (2 × 20 mL), and the combined extracts were washed with 20% aqueous LiCl (2 × 20 mL), dried over $MgSO_4$, and concentrated under reduced pressure. The crude residue was purified by automated flash column chromatography (50g silica column, 0-40-50% EtOAc/DCM) to afford the desired product as a viscous solid (997 mg, 82% yield).

^{1}H NMR (500 MHz, $CDCl_3$) δ 7.62 – 7.55 (m, 2H), 7.54 – 7.46 (m, 3H), 6.34 (t, *J* = 5.4 Hz, 1H), 3.41 (q, *J* = 6.5 Hz, 2H), 2.80 (s, 1H), 2.68 (s, 3H), 1.54 (t, *J* = 6.6 Hz, 2H), 1.14 (s, 6H).

^{13}C NMR (126 MHz, $CDCl_3$) δ 173.64, 162.00, 160.39, 130.35, 129.27, 129.10, 128.30, 111.31, 71.24, 40.71, 35.92, 29.43, 12.82.

IR (film) ν_{max} 3409, 2970, 2932, 1642, 1534, 1466, 1444, 1412, 1379, 1178, 842, 773, 730 cm^{-1}.

HRMS (ESI-TOF) *m/z* calculated for $C_{16}H_{20}N_2O_3$+ ($[M+H]^+$) 311.1366, found 311.1371.

(4*R*)-1-methyl-4-(prop-1-en-2-yl)-2-(1*H*-1,2,4-triazol-1-yl)cyclohexan-1-ol

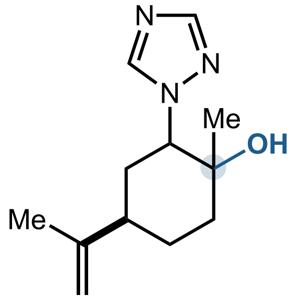

To an oven-dried 40-mL scintillation vial with a magnetic stir bar was added 1*H*-1,2,4-triazole (690.7 mg, 10.0 mmol, 1.0 equiv.) (+)-limonene oxide (1.82 g, 12.0 mmol, 1.2 equiv.), and DMF (15.0 mL) was added to dissolve the reactants. Cs_2CO_3 (3.91 g, 12.0 mmol, 1.2 equiv.) was added in one portion, and the reaction was stirred at 120 °C for 12 hours. The reaction mixture was cooled to room temperature, and solvent was removed by GeneVac HT-4X Centrifugal Vacuum Evaporator (36 °C, < 2 mbar). The residue was then purified by automated flash column chromatography (100 g silica column, 0-100% EtOAc/hexanes) to afford the title compound as an off-white solid (mixture of diastereomers, 9:1 d.r.).

^{1}H NMR (500 MHz, $CDCl_3$) δ 8.16 (s, 1H) [8.18 (s, 1H)], 7.98 (s, 1H) [8.01 (s, 1H)], 4.93 (s, 1H), 4.85 (s, 1H) [4.84 (s, 1H)], 4.40 (dd, *J* = 8.4, 4.4 Hz, 1H), 2.76 (t, *J* = 5.6 Hz, 1H), 2.66 (s, 1H), 2.43 – 2.34 (m, 1H) [2.31 – 2.19 (m, 1H)], 2.10 – 1.86 (m, 3H), 1.79 – 1.70 (m, 5H), 0.97 (s, 5H). **(minor diastereomer in brackets)**

^{13}C NMR (126 MHz, $CDCl_3$) δ 151.65, 151.59, 147.59, 146.49, 143.23, 142.54, 110.75, 110.01, 72.60, 72.02, 66.93, 63.42, 44.16, 38.95, 38.09, 34.89, 32.61, 30.93, 28.07, 25.32, 23.52, 21.78, 20.96, 20.54. **(summary of all diastereomers)**

IR (film) ν_{max} 3269, 2957, 2922, 1642, 1510, 1447, 1273, 1211, 1135, 956, 856, 754, 676 cm^{-1}.

HRMS (ESI-TOF) *m/z* calculated for $C_{12}H_{20}N_3O^+$ ($[M+]^+$) 222.1601, found 222.1601.

6. Synthesis and Characterization of Pyridinium Salts

The pyridinium salts were prepared according to the following procedures previously reported in the literature.[6]

General Procedure PA:

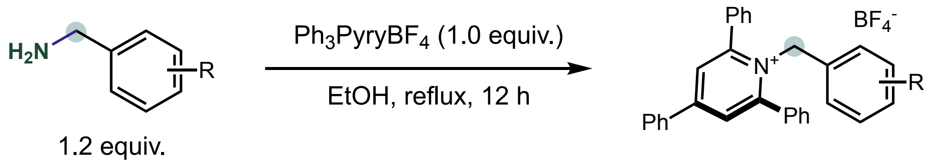

The benzylamine (1.2 equiv) was added to a suspension of 2,4,6-triphenylpyrylium tetrafluoroborate (1.0 equiv) and EtOH (1.0 M) in a 100-mL round-bottomed flask. The flask was then fitted with a reflux condenser. The mixture was stirred and heated at reflux in an oil bath at 80 °C for 12 h. The reaction was then allowed to cool to room temperature, after which Et_2O (2–3x volume of EtOH used) was added and the vigorously stirred for 1 h to induce precipitation. The solids were then collected by filtration, washed with Et_2O until the filtrates are colourless, and dried under vacuum to afford the analytically pure pyridinium salt. If precipitation failed, Et_2O was removed via rotary evaporation, and the crude residue was purified by automated flash column chromatography on silica with acetone/DCM as the eluent.

[6] Basch, C.H; Liao, J.; Xu, J.; Piane, J.J.; Watson, M.P. *J. Am. Chem. Soc.* **2017** *139*, 5313-5316. DOI: 10.1021/jacs.7b02389.

General Procedure PB:

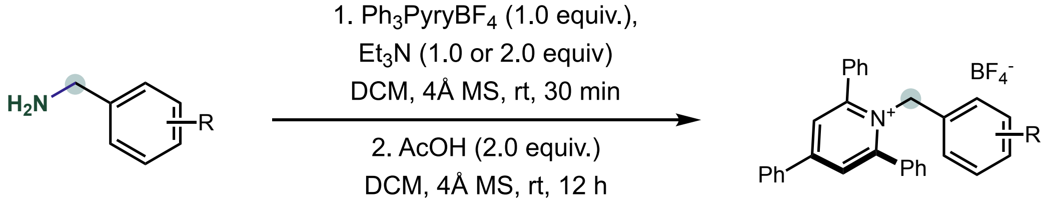

To a suspension of 2,4,6-triphenylpyrylium tetrafluoroborate (1.0 equiv) and powdered activated 4Å molecular sieves (~500 mg/mmol) in DCM (0.5 M) in a 40-mL scintillation vial equipped with a stirbar and a rubber septum was added the benzylamine or benzylamine HCl salt (1.0 equiv.) in one portion. Et_3N (1.0 equiv. for free base amines; 2.0 equiv. for amine hydrochloride salts) was then added via syringe, and the mixture was stirred at room temperature for 30 min, after which AcOH (2.0 equiv) was added in one portion. The reaction was then stirred overnight at room temperature. After complete consumption of the amine starting material, the mixture was filtered through a short pad of Celite, and the filter cake was rinsed with DCM (3 × 20 mL). The filtrate was then washed successively with 1 M HCl (30 mL), saturated aqueous $NaHCO_3$ (30 mL), and brine (30 mL). The combined organic layer was dried over Na_2SO_4 and concentrated under reduced pressure. Et_2O (ca. 100 mL) was added to the resulting residue to precipitate the pyridinium salt, and the suspension was triturated with vigorous stirring at room temperature for 1 h. The solids were then collected by filtration, washed with Et_2O until the filtrates are colourless, and dried under vacuum to afford the analytically pure pyridinium salt. If precipitation failed, Et_2O was removed via rotary evaporation, and the crude residue was purified by automated flash column chromatography on silica with acetone/DCM as the eluent.

1-(4-((aminooxy)carbonyl)benzyl)-2,4,6-triphenylpyridin-1-ium tetrafluoroborate:

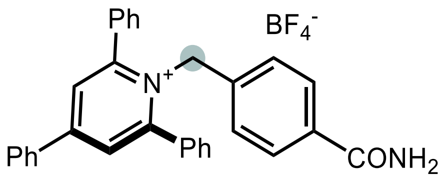

Prepared according to **General Procedure PA** using 2,4,6-triphenylpyrylium tetrafluoroborate (1.65 g, 4.17 mmol, 1.0 equiv) and 4-(aminomethyl)benzamide (751 mg, 5.0 mmol, 1.2 equiv) and the solids were purified by automated flash column chromatography (25 g silica column, 0-30-80% acetone/DCM) to afford the title compound as an off-white solid (1.17 g, 53% yield).

^{1}H NMR (500 MHz, $CDCl_3$) δ 8.00 (s, 2H), 7.85 (d, *J* = 7.6 Hz, 2H), 7.65 – 7.44 (m, 15H), 6.58 (d, *J* = 7.9 Hz, 2H), 6.23 (br-s, 1H), 5.80 (s, 2H), 5.52 (br-s, 1H).

^{13}C NMR (126 MHz, $CDCl_3$) δ 168.22, 157.75, 156.96, 137.65, 133.73, 133.44, 132.81, 132.52, 131.44, 130.05, 129.51, 129.09, 128.38, 128.27, 126.85, 126.40, 58.10.

^{19}F NMR (376 MHz, $CDCl_3$) δ -152.71 (minor, $^{11}BF_4$), -152.76 (major, $^{10}BF_4$).

IR (film) ν_{max} 3455, 3373, 3200, 3063, 1670, 1619, 1600, 1563, 1415, 1385, 1054, 910, 763, 727, 701, 647 cm^{-1}.

HRMS (ESI-TOF) *m/z* calculated for $C_{31}H_{25}N_2O^+$ ([M–BF_4]$^+$) 441.1961, found 441.1963.

2,4,6-triphenyl-1-(4-(4,4,5,5-tetramethyl-1,3,2-dioxaborolan-2-yl)benzyl)pyridin-1-ium tetrafluoroborate:

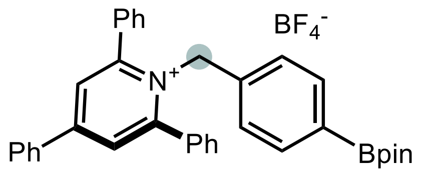

Prepared according to **General Procedure PA** using 2,4,6-triphenylpyrylium tetrafluoroborate (660 mg, 1.67 mmol, 1.0 equiv) and [4-(4,4,5,5-tetramethyl-1,3,2-dioxaborolan-2-yl)phenyl]methanamine (466 mg, 2.0 mmol, 1.2 equiv). The crude solids were purified by automated flash column chromatography (25 g silica column, 0-20% acetone/DCM) to afford the title compound as an off-white solid (735 mg, 72% yield).

^{1}H NMR (500 MHz, $CDCl_3$) δ 7.98 (s, 2H), 7.87 – 7.81 (m, 2H), 7.74 – 7.39 (m, 15H), 6.50 (d, *J* = 8.1 Hz, 2H), 5.76 (s, 2H), 1.32 (s, 12H).

^{13}C NMR (126 MHz, $CDCl_3$) δ 157.69, 156.47, 137.18, 135.24, 133.88, 132.68, 132.52, 131.10, 129.92, 129.25, 129.15, 128.30, 126.68, 125.33, 84.19, 58.29, 24.96.

Note: The carbon directly attached to boron was not observed due to quadrupolar relaxation.

^{19}F NMR (376 MHz, $CDCl_3$) δ -153.26 (minor, $^{11}BF_4$), -153.31 (major, $^{10}BF_4$).

IR (film) ν_{max} 3068, 2979, 1622, 1565, 1409, 1399, 1361, 1326, 1143, 1087, 1056, 789, 726, 701 cm^{-1}.

HRMS (ESI-TOF) *m/z* calculated for $C_{36}H_{35}BNO_2^+$ ($[M–BF_4]^+$) 524.2755, found 524.2762.

1-(3-((*tert*-butoxycarbonyl)amino)benzyl)-2,4,6-triphenylpyridin-1-ium tetrafluoroborate:

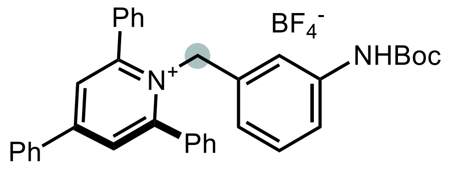

Prepared according to **General Procedure PA** using 2,4,6-triphenylpyrylium tetrafluoroborate (660 mg, 1.67 mmol, 1.0 equiv) and *tert*-butyl N-[3-(aminomethyl)phenyl]carbamate (445 mg, 2.0 mmol, 1.2 equiv) and obtained as an off-white solid (579 mg, 58% yield).

^{1}H NMR (500 MHz, $CDCl_3$) δ 7.98 (s, 2H), 7.88 – 7.83 (m, 2H), 7.70 – 7.44 (m, 13H), 6.96 (t, *J* = 7.9 Hz, 1H), 7.26 (overlap with $CDCl_3$, 1H), 6.71 (s, 1H), 6.69 (s, 1H), 6.03 (d, *J* = 7.7 Hz, 1H), 5.68 (s, 2H), 1.49 (s, 9H).

^{13}C NMR (126 MHz, $CDCl_3$) δ 157.40, 156.57, 152.88, 139.75, 134.61, 134.13, 132.84, 132.27, 130.98, 129.77, 129.24, 129.21, 128.35, 126.96, 120.48, 118.05, 115.82, 80.52, 58.22, 28.36.

^{19}F NMR (376 MHz, $CDCl_3$) δ -152.57 (minor, $^{11}BF_4$), -152.62 (major, $^{10}BF_4$).

IR (film) ν_{max} 3357, 3063, 2979, 1716, 1620, 1600, 1541, 1494, 1444, 1235, 1156, 1052, 1031, 998, 910, 891, 764, 727, 699, 647 cm^{-1}.

HRMS (ESI-TOF) *m/z* calculated for $C_{35}H_{33}N_2O_2^+$ ($[M–BF_4]^+$) 513.2537, found 513.2540.

1-(4-(((*tert*-butoxycarbonyl)amino)methyl)benzyl)-2,4,6-triphenylpyridin-1-ium tetrafluoroborate:

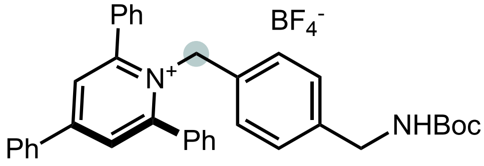

Prepared according to **General Procedure PA** using 2,4,6-triphenylpyrylium tetrafluoroborate (1.98 g, 5.0 mmol, 1.0 equiv) and *tert*-butyl (4-(aminomethyl)benzyl)carbamate (1.42 g, 6.0 mmol, 1.2 equiv) and obtained as an off-white solid (2.01 g, 65% yield).

^{1}H NMR (500 MHz, CDCl$_3$) δ 7.92 (s, 2H), 7.79 (d, *J* = 7.1 Hz, 2H), 7.67 – 7.37 (m, 13H), 7.01 (d, *J* = 7.8 Hz, 2H), 6.41 (d, *J* = 7.8 Hz, 2H), 5.73 (s, 2H), 4.88 (s, 1H), 4.18 (d, *J* = 6.0 Hz, 2H), 1.43 (s, 9H).

^{13}C NMR (126 MHz, CDCl$_3$) δ 157.68, 156.45, 155.99, 139.70, 133.89, 133.11, 132.84, 132.53, 131.12, 129.93, 129.31, 129.20, 128.29, 127.79, 126.71, 126.59, 79.79, 58.12, 44.06, 28.50.

^{19}F NMR (376 MHz, CDCl$_3$) δ -152.98 (minor, $^{11}BF_4$), -153.03 (major, $^{10}BF_4$).

IR (film) ν_{max} 3399, 2977, 1699, 1619, 1561, 1495, 1365, 1248, 1163, 1049, 765, 699, 519 cm^{-1}.

HRMS (ESI-TOF) *m/z* calculated for $C_{36}H_{35}N_2O_2^+$ ($[M–BF_4]^+$) 527.2693, found 527.2692.

2,4,6-triphenyl-1-(pyrazolo[1,5-*a*]pyridin-2-ylmethyl)pyridin-1-ium tetrafluoroborate:

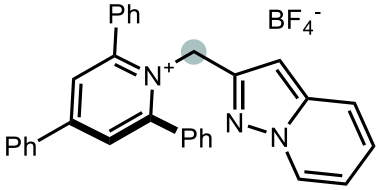

Prepared according to **General Procedure PB** using pyrazolo[1,5-*a*]pyridin-2-ylmethanamine (330 mg, 2.24 mmol, 1.2 equiv.), 2,4,6-triphenylpyrylium tetrafluoroborate (741 mg, 1.87 mmol, 1.0 equiv), powdered activated 4Å molecular sieves (935 mg), Et_3N (261 µL, 1.87 mmol, 1.0 equiv.), and AcOH (214 µL, 3.74 mmol, 2.0 equiv.).

After work-up according to **General Procedure PB**, the crude residue was purified by automated flash column chromatography (50 g silica column, 0-10% acetone/DCM) to afford the title compound as a yellow solid (827.5 mg, 84% yield).

^{1}H NMR (500 MHz, $CDCl_3$) δ 8.31 (dd, *J* = 7.1, 1.0 Hz, 1H), 7.96 (s, 2H), 7.88 – 7.82 (m, 2H), 7.74 – 7.64 (m, 4H), 7.62 – 7.53 (m, 3H), 7.50 – 7.44 (m, 2H), 7.42 – 7.37 (m, 4H), 7.33 (dt, *J* = 8.9, 1.3 Hz, 1H), 7.10 (ddd, *J* = 8.9, 6.7, 1.1 Hz, 1H), 6.75 (td, *J* = 6.9, 1.4 Hz, 1H), 5.80 (s, 2H), 5.74 (s, 1H).

^{13}C NMR (126 MHz, $CDCl_3$) δ 157.53, 156.06, 148.06, 141.14, 134.13, 132.87, 132.29, 130.88, 129.84, 129.06, 128.92, 128.39, 128.24, 126.37, 124.27, 118.17, 112.40, 95.06, 53.51.

^{19}F NMR (376 MHz, $CDCl_3$) δ -153.50 (minor, $^{11}BF_4$), -153.55 (major, $^{10}BF_4$).

IR (film) ν_{max} 3063, 1620, 1599, 1564, 1519, 1495, 1415, 1330, 1254, 1052, 999, 913, 892, 763, 728, 700 cm^{-1}.

HRMS (ESI-TOF) *m/z* calculated for $C_{31}H_{24}N_3^+$ ([M–BF_4]$^+$) 438.1965, found 438.1970.

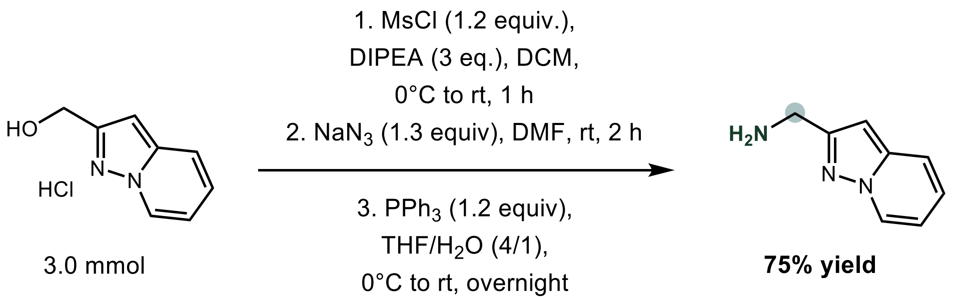

An oven-dried 50-mL round-bottom flask equipped with a magnetic stirring bar and septum was charged with pyrazolo[1,5-*a*]pyridin-2-ylmethanol hydrochloride (554 mg, 3.0 mmol, 1.0 equiv.), which was dissolved in DCM (15 mL). The solution was cooled to 0 °C in an ice bath, followed by sequential addition of DIPEA (1.57 mL, 9.0 mmol, 3.0 equiv.) and methanesulfonyl chloride (279 μL, 3.6 mmol, 1.2 equiv.) via micropipette. The mixture was then allowed to warm to room temperature and stirred for 1 h. After completion, saturated aqueous $NaHCO_3$ (15 mL) was added to quench the reaction. The biphasic mixture was poured into a separatory funnel, and the layers were separated. The aqueous layer was extracted with DCM (3 × 10 mL), dried over Na_2SO_4, and concentrated under reduced pressure. The crude mesylate was used directly in the subsequent reaction without further purification.

A 50-mL round-bottom flask equipped with a magnetic stirring bar and septum was charged with the mesylate intermediate, which was dissolved in DMF (6.4 mL). Sodium azide (242 mg, 3.72 mmol, 1.3 equiv.) was added, and the resulting mixture was stirred at room temperature for 2 h. Water (20 mL) was added, and the biphasic mixture was poured into a separatory funnel. The aqueous layer was extracted with EtOAc (2 × 10 mL), and the combined extracts were washed with 20% aqueous LiCl (2 × 10 mL), dried over Na_2SO_4, and concentrated under reduced pressure. The crude azide was used directly in the subsequent reaction without further purification.

Azide: **^{1}H NMR (500 MHz, $CDCl_3$)** δ 8.44 – 8.41 (m, 1H), 7.51 (dt, *J* = 8.9, 1.2 Hz, 1H), 7.13 (ddd, *J* = 8.9, 6.8, 1.1 Hz, 1H), 6.77 (td, *J* = 6.8, 1.4 Hz, 1H), 6.51 (s, 1H), 4.55 (s, 2H).

Note: isolated with ~0.27 equiv. residual DMF.

A 50-mL round-bottom flask equipped with a magnetic stirring bar and septum was charged with the crude azide (496 mg, 2.86 mmol, 1.0 equiv.), which was dissolved in THF (7.6 mL) and water (1.9 mL). The mixture was cooled to 0 °C in an ice bath, and triphenylphosphine (901 mg, 3.44 mmol, 1.2 equiv.) was added. The mixture was then allowed to warm to room temperature and stirred overnight. After completion, the reaction mixture was concentrated under reduced pressure. The crude residue was purified by automated flash column chromatography (25g silica column, 0-100% EtOAc/hexanes then 0-30% MeOH/EtOAc) to afford the desired product as a yellow solid (332 mg, 75% yield over three steps), which was then converted to the corresponding pyridinium salt as described above.

^{1}H NMR (500 MHz, CD_3OD) δ 8.45 (dd, *J* = 7.0, 1.0 Hz, 1H), 7.58 (d, *J* = 8.9 Hz, 1H), 7.19 (ddd, *J* = 8.9, 6.6, 0.9 Hz, 1H), 6.84 (td, *J* = 6.9, 1.4 Hz, 1H), 6.55 (s, 1H), 4.02 (s, 2H).

1-((1-methyl-1*H*-pyrazol-3-yl)methyl)-2,4,6-triphenylpyridin-1-ium tetrafluoroborate:

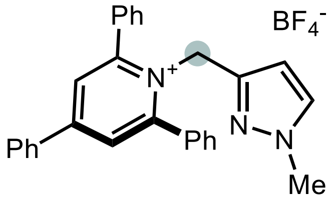

Prepared according to **General Procedure PB** using (1-methylpyrazol-3-yl)methanamine (333 mg, 3.0 mmol, 1.2 equiv.), 2,4,6-triphenylpyrylium tetrafluoroborate (990 mg, 2.5 mmol, 1.0 equiv), powdered activated 4Å molecular sieves (1.25 g), Et_3N (348 µL, 2.50 mmol, 1.0 equiv.), and AcOH (286 µL, 5.00 mmol, 2.0 equiv.).

After work-up according to **General Procedure PB**, the crude residue was purified by automated flash column chromatography (25 g silica column, 0-15-20% acetone/DCM) to afford the title compound as a light yellow solid (834.0 mg, 68% yield).

^{1}H NMR (500 MHz, $CDCl_3$) δ 7.92 (s, 2H), 7.85 – 7.80 (m, 2H), 7.72 – 7.65 (m, 4H), 7.62 – 7.43 (m, 9H), 7.10 (d, *J* = 2.2 Hz, 1H), 5.56 (s, 2H), 5.42 (d, *J* = 2.2 Hz, 1H), 3.80 (s, 3H).

^{13}C NMR (126 MHz, $CDCl_3$) δ 157.37, 155.80, 145.23, 134.09, 132.93, 132.24, 131.52, 130.85, 129.82, 129.06, 128.91, 128.19, 126.31, 104.07, 53.27, 39.19.

^{19}F NMR (376 MHz, $CDCl_3$) δ -153.61 (minor, $^{11}BF_4$), -153.66 (major, $^{10}BF_4$).

IR (film) ν_{max} 3063, 1620, 1599, 1564, 1495, 1049, 1034, 999, 912, 892, 764, 726, 699, 650 cm^{-1}.

HRMS (ESI-TOF) *m/z* calculated for $C_{28}H_{24}N_3^+$ ([M–BF_4]$^+$) 402.1965, found 402.1969.

1-((1-methyl-1*H*-pyrazol-5-yl)methyl)-2,4,6-triphenylpyridin-1-ium tetrafluoroborate:

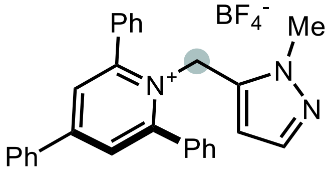

Prepared according to **General Procedure PB** using (2-methylpyrazol-3-yl)methanamine (333 mg, 3.0 mmol, 1.2 equiv.), 2,4,6-triphenylpyrylium tetrafluoroborate (990 mg, 2.5 mmol, 1.0 equiv), powdered activated 4Å molecular sieves (1.25 g), Et_3N (348 µL, 2.50 mmol, 1.0 equiv.), and AcOH (286 µL, 5.00 mmol, 2.0 equiv.).

After work-up according to **General Procedure PB**, the crude residue was purified by automated flash column chromatography (25 g silica column, 0-20-25% acetone/DCM) to afford the title compound as a white solid (950 mg, 77% yield).

^{1}H NMR (500 MHz, $CDCl_3$) δ 7.98 (s, 2H), 7.86 – 7.80 (m, 2H), 7.70 – 7.52 (m, 13H), 7.31 (d, *J* = 1.9 Hz, 1H), 5.77 (d, *J* = 2.0 Hz, 1H), 5.72 (s, 2H), 3.03 (s, 3H).

^{13}C NMR (126 MHz, $CDCl_3$) δ 157.44, 156.81, 138.73, 135.81, 133.57, 132.78, 132.10, 131.41, 129.99, 129.43, 129.00, 128.29, 126.64, 105.60, 50.70, 35.96.

^{19}F NMR (376 MHz, $CDCl_3$) δ -152.84 (minor, $^{11}BF_4$), -152.89 (major, $^{10}BF_4$).

IR (film) ν_{max} 3063, 1621, 1598, 1563, 1496, 1054, 1000, 914, 766, 729, 702 cm^{-1}.

HRMS (ESI-TOF) *m/z* calculated for $C_{28}H_{24}N_3^+$ ($[M–BF_4]^+$) 402.1965, found 402.1972.

1-((2-(4-fluorophenyl)thiazol-4-yl)methyl)-2,4,6-triphenylpyridin-1-ium tetrafluoroborate:

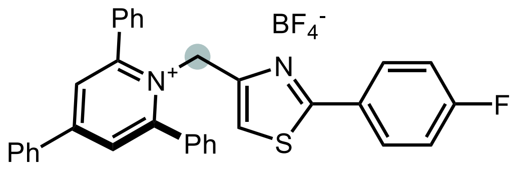

Prepared according to **General Procedure PA** using 2,4,6-triphenylpyrylium tetrafluoroborate (1.32 g, 3.33 mmol, 1.0 equiv) and [2-(4-fluorophenyl)thiazol-4-yl]methanamine (833 mg, 4.0 mmol, 1.2 equiv). The crude solids were purified by automated flash column chromatography (25 g silica column, 0-30-80% acetone/DCM) to afford the title compound as a slightly yellow solid (1.20 g, 61% yield).

^{1}H NMR (500 MHz, CDCl$_3$) δ 7.96 (s, 2H), 7.87 – 7.82 (m, 2H), 7.81 – 7.67 (m, 6H), 7.63 – 7.47 (m, 9H), 7.16 – 7.09 (m, 2H), 6.31 (s, 1H), 5.80 (s, 2H).

^{13}C NMR (126 MHz, CDCl$_3$) δ 167.62, 164.20 (d, J = 251.6 Hz), 157.54, 156.17, 148.81, 133.94, 133.07, 132.46, 131.06, 129.91, 129.33 (d, J = 3.4 Hz), 129.19, 129.16, 128.40 (d, J = 8.6 Hz), 128.24, 126.36, 117.31, 116.37 (d, J = 22.2 Hz), 54.82.

^{19}F NMR (376 MHz, CDCl$_3$) δ -109.23 (tt, J = 8.1, 5.3 Hz, 1F), -153.03 (minor, $^{11}BF_4$), -153.08 (major, $^{10}BF_4$).

IR (film) ν_{max} 3111, 3069, 1620, 1601, 1563, 1510, 1458, 1233, 1158, 1050, 1035, 1002, 909, 893, 841, 762, 726, 699, 647 cm^{-1}.

HRMS (ESI-TOF) *m/z* calculated for $C_{33}H_{24}FN_2S^+$ ([M–BF$_4$]$^+$) 499.1639, found 499.1644.

1-((2-(2-chlorophenyl)oxazol-4-yl)methyl)-2,4,6-triphenylpyridin-1-ium tetrafluoroborate:

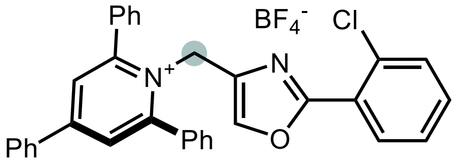

Prepared according to **General Procedure PA** using 2,4,6-triphenylpyrylium tetrafluoroborate (1.65 g, 4.17 mmol, 1.0 equiv) and [2-(2-chlorophenyl)oxazol-4-yl]methanamine (1.04 g, 5.0 mmol, 1.2 equiv). The crude solids were purified by automated flash column chromatography (50 g silica column, 0-6-10% acetone/DCM) to afford the title compound as an off-white solid (1.17 g, 47% yield).

^{1}H NMR (500 MHz, $CDCl_3$) δ 7.95 (s, 2H), 7.88 – 7.78 (m, 7H), 7.63 – 7.48 (m, 10H), 7.41 (td, *J* = 7.7, 1.9 Hz, 1H), 7.36 (td, *J* = 7.6, 1.4 Hz, 1H), 6.76 (s, 1H), 5.73 (s, 2H).

^{13}C NMR (126 MHz, $CDCl_3$) δ 159.75, 157.64, 156.27, 136.57, 134.41, 133.97, 132.97, 132.70, 132.42, 131.77, 131.45, 131.18, 130.80, 129.90, 129.36, 129.21, 128.22, 127.17, 126.46, 125.60, 51.07.

^{19}F NMR (376 MHz, $CDCl_3$) δ -153.26 (minor, $^{11}BF_4$), -153.31 (major, $^{10}BF_4$).

IR (film) ν_{max} 3134, 3064, 1620, 1599, 1564, 1495, 1416, 1051, 1032, 999, 911, 892, 764, 728, 699, 648 cm^{-1}.

HRMS (ESI-TOF) *m/z* calculated for $C_{33}H_{24}ClN_2O^+$ ([M–BF_4]$^+$) 499.1572, found 499.1580.

1-((1-(2-fluorobenzyl)-1H-1,2,3-triazol-4-yl)methyl)-2,4,6-triphenylpyridin-1-ium tetrafluoroborate:

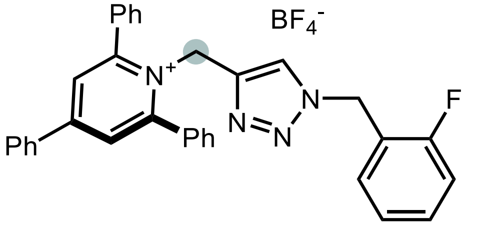

Prepared according to **General Procedure PB** using (1-(2-fluorobenzyl)-1*H*-1,2,3-triazol-4-yl)methanamine hydrochloride (766 mg, 3.0 mmol, 1.2 equiv.), 2,4,6-triphenylpyrylium tetrafluoroborate (990 mg, 2.5 mmol, 1.0 equiv), powdered activated 4Å molecular sieves (1.25 g), Et_3N (766 μL, 5.5 mmol, 2.2 equiv.), and AcOH (286 μL, 5.00 mmol, 2.0 equiv.).

After work-up according to **General Procedure PB**, the crude residue was purified by automated flash column chromatography (25 g silica column, 0-15% acetone/DCM) to afford the title compound as a light yellow solid (820 mg, 56% yield).

^{1}H NMR (500 MHz, $CDCl_3$) δ 7.92 (s, 2H), 7.83 – 7.76 (m, 2H), 7.71 – 7.65 (m, 4H), 7.62 – 7.51 (m, 3H), 7.49 – 7.33 (m, 7H), 7.24 – 7.10 (m, 3H), 6.71 (s, 1H), 5.75 (s, 2H), 5.37 (s, 2H).

^{13}C NMR (126 MHz, $CDCl_3$) δ 160.73 (d, J = 248.8 Hz), 157.42, 156.39, 140.88, 134.00, 132.74, 132.38, 131.35 (d, J = 8.2 Hz), 131.13 (d, J = 3.1 Hz), 131.04, 129.88, 129.04, 129.02, 128.20, 126.49, 124.94 (d, J = 3.8 Hz), 122.40, 121.52 (d, J = 14.8 Hz), 50.72, 47.79 (d, J = 4.0 Hz).

^{19}F NMR (376 MHz, $CDCl_3$) δ -117.39 – -117.47 (m, 1F), -153.16 (minor, $^{11}BF_4$), -153.21 (major, $^{10}BF_4$).

IR (film) ν_{max} 3064, 1620, 1599, 1563, 1494, 1235, 1049, 999, 911, 790, 761, 726, 700, 650 cm^{-1}.

HRMS (ESI-TOF) *m/z* calculated for $C_{33}H_{26}FN_4^+$ ($[M–BF_4]^+$) 497.2136, found 497.2139.

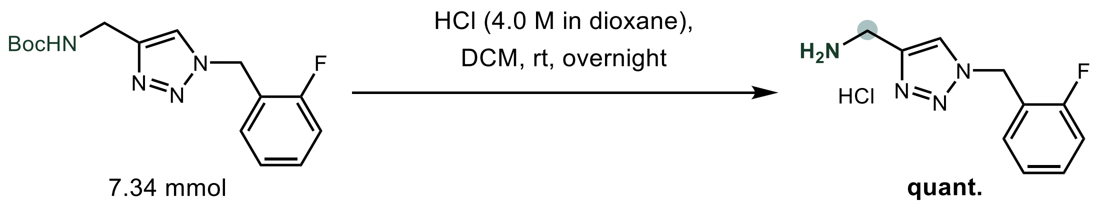

The Boc-protected starting material was prepared according to a literature procedure.[7] An oven-dried 50-mL round-bottom flask equipped with a magnetic stirring bar and septum was charged *tert*-butyl ((1-(2-fluorobenzyl)-1*H*-1,2,3-triazol-4-yl)methyl)carbamate (2.25 g, 7.34 mmol, 1.0 equiv.), which was then dissolved in DCM (16 mL). HCl (6.6 mL, 4.0 M in dioxane) was added in one portion, and the mixture was stirred overnight at room temperature. The precipitated solid was triturated with hexanes (20 mL) and collected by filtration. The solid was dried under high vacuum for 2 h to afford analytically pure (1-(2-fluorobenzyl)-1*H*-1,2,3-triazol-4-yl)methanamine hydrochloride as an off-white solid (1.92 g, quantitative yield), which was then converted to the corresponding pyridinium salt as described above.

^{1}H NMR (500 MHz, DMSO-*d$_6$*) δ 8.35 (br-s, 3H), 8.19 (s, 1H), 7.47 – 7.37 (m, 2H), 7.31 – 7.20 (m, 2H), 5.71 (s, 2H), 4.10 (q, *J* = 5.8 Hz, 1H).

^{19}F NMR (376 MHz, DMSO-*d$_6$*) δ -117.58 – -117.69 (m, 1F).

[7] Shao, C.; Wang, X.; Zhang, Q.; Luo, S.; Zhao, J.; Hu, Y. Acid–Base Jointly Promoted Copper(I)-Catalyzed Azide–Alkyne Cycloaddition. *J. Org. Chem.* **2011,** *76* (16), 6832–6836. DOI: 10.1021/jo200869a.

2,4,6-triphenyl-1-((2-trityl-2H-tetrazol-5-yl)methyl)pyridin-1-ium tetrafluoroborate:

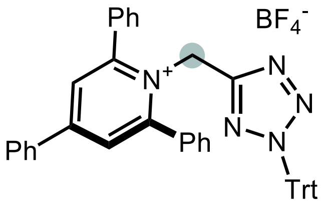

Prepared according to **General Procedure PA** using 2,4,6-triphenylpyrylium tetrafluoroborate (689 mg, 1.74 mmol, 1.0 equiv) and (2-trityltetrazol-5-yl)methanamine (713 mg, 2.09 mmol, 1.2 equiv) and the solids were purified by automated flash column chromatography (50 g silica column, 0-10% acetone/DCM) to afford the title compound as an off-white solid (253 mg, 20% yield).

^{1}H NMR (500 MHz, Acetone-*d*$_6$) δ 8.52 (s, 2H), 8.30 – 8.24 (m, 2H), 7.76 – 7.60 (m, 9H), 7.59 – 7.52 (m, 4H), 7.49 – 7.35 (m, 9H), 7.04 – 6.96 (m, 6H), 6.13 (s, 2H).

^{13}C NMR (126 MHz, Acetone-*d*$_6$) δ 160.34, 158.52, 157.87, 141.89, 134.53, 133.84, 133.52, 132.38, 131.05, 130.82, 130.22, 130.08, 129.79, 129.60, 128.89, 127.59, 84.67, 51.54.

^{19}F NMR (376 MHz, Acetone-*d*$_6$) δ -151.86 (minor, $^{11}BF_4$), -151.91 (major, $^{10}BF_4$).

IR (film) ν_{max} 3062, 1699, 1621, 1598, 1564, 1495, 1447, 1059, 1036, 1000, 765, 749, 700 cm^{-1}.

HRMS (ESI-TOF) *m/z* calculated for $C_{44}H_{34}N_5^+$ ([M–BF$_4$]$^+$) 632.2809, found 632.2813.

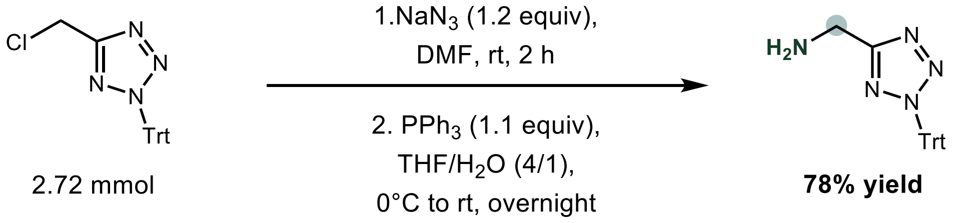

The starting material was prepared according to a literature procedure.[8] In an oven-dried 50-mL round-bottom flask equipped with a septum and a magnetic stirring bar, 5-(chloromethyl)-

[8] Eli Lilly and Company. Selective Beta 3 Adrenergic Agonists. U.S. Patent 5,786,356, 1998.

2-trityl-2*H*-tetrazole (1.0 g, 2.72 mmol, 1.0 equiv.) was dissolved in DMF (6.0 mL). Sodium azide (212 mg, 3.26 mmol, 1.2 equiv.) was added, and the resulting mixture was stirred at room temperature for 2 h. Water (30 mL) was added, and the resulting precipitate was collected by filtration. The solid was washed with water (3 × 10 mL) and 50% Et_2O/hexanes (10 mL). The filtrate was extracted with Et_2O (20 mL), and the organic extracts were dried over Na_2SO_4, and concentrated under reduced pressure. The residue and precipitated solid were used directly in the subsequent reaction without further purification.

Azide: **^{1}H NMR (500 MHz, $CDCl_3$)** δ 7.42 – 7.30 (m, 9H), 7.15 – 7.07 (m, 6H), 4.63 (s, 2H).

A 50-mL round-bottom flask equipped with a magnetic stirring bar and septum was charged with the crude azide (981 mg, 2.67 mmol, 1.0 equiv.), which was dissolved in THF (7.2 mL) and water (1.8 mL). The mixture was cooled to 0 °C in an ice bath, and triphenylphosphine (770 mg, 2.94 mmol, 1.1 equiv.) was added. The mixture was then allowed to warm to room temperature and stirred overnight. After completion, the reaction mixture was concentrated under reduced pressure. The crude residue was purified by automated flash column chromatography (25g silica column, 0-100% EtOAc/hexanes then 0-25% MeOH/EtOAc) to afford the desired product as a crystalline white solid (715 mg, 78% yield over two steps), which was then converted to the corresponding pyridinium salt as described above.

^{1}H NMR (500 MHz, $CDCl_3$) δ 7.39 – 7.30 (m, 9H), 7.13 – 7.06 (m, 6H), 4.16 (s, 2H).

1-((6-(methoxycarbonyl)pyridin-2-yl)methyl)-2,4,6-triphenylpyridin-1-ium tetrafluoroborate:

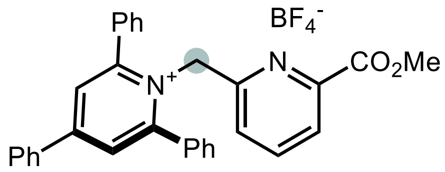

Prepared according to **General Procedure PB** using methyl 6-(aminomethyl)pyridine-2-carboxylate hydrochloride (586 mg, 2.75 mmol, 1.1 equiv.), 2,4,6-triphenylpyrylium tetrafluoroborate (990 mg, 2.5 mmol, 1.0 equiv), powdered activated 4Å molecular sieves (1.25 g), Et_3N (731 μL, 5.25 mmol, 2.1 equiv.), and AcOH (286 μL, 5.00 mmol, 2.0 equiv.).

After work-up according to **General Procedure PB**, the crude residue was purified by automated flash column chromatography (25 g silica column, 0-15% acetone/DCM) to afford the title compound as a light yellow solid (1.20 g, 87% yield).

^{1}H NMR (500 MHz, $CDCl_3$) δ 7.96 (s, 2H), 7.91 (d, J = 7.7 Hz, 1H), 7.87 – 7.81 (m, 2H), 7.73 – 7.64 (m, 4H), 7.62 – 7.51 (m, 4H), 7.50 – 7.45 (m, 2H), 7.44 – 7.38 (m, 4H), 6.70 (d, J = 7.6 Hz, 1H), 5.85 (s, 2H), 3.95 (s, 3H).

^{13}C NMR (126 MHz, $CDCl_3$) δ 164.94, 157.71, 156.13, 153.45, 147.56, 138.04, 134.02, 132.92, 132.40, 130.98, 129.89, 129.11, 129.02, 128.22, 126.12, 125.10, 124.48, 58.71, 52.90.

^{19}F NMR (376 MHz, $CDCl_3$) δ -153.17 (minor, $^{11}BF_4$), -153.23 (major, $^{10}BF_4$).

IR (film) ν_{max} 3065, 1725, 1621, 1599, 1565, 1495, 1444, 1319, 1301, 1249, 1166, 1142, 1051, 997, 913, 893, 762, 728, 701, 671 cm^{-1}.

HRMS (ESI-TOF) *m/z* calculated for $C_{31}H_{25}N_2O_2^+$ ([M–BF_4]$^+$) 457.1911, found 457.1916.

1-(cyclohex-1-en-1-ylmethyl)-2,4,6-triphenylpyridin-1-ium tetrafluoroborate:

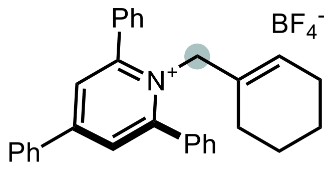

Prepared according to **General Procedure PB** using cyclohex-1-en-1-ylmethanamine hydrochloride (480 mg, 3.25 mmol, 1.3 equiv.), 2,4,6-triphenylpyrylium tetrafluoroborate (990 mg, 2.5 mmol, 1.0 equiv), powdered activated 4Å molecular sieves (1.25 g), Et_3N (801 μL, 5.75 mmol, 2.1 equiv.), and AcOH (286 μL, 5.00 mmol, 2.0 equiv.).

After work-up according to **General Procedure PB**, the crude residue was purified by automated flash column chromatography (25 g silica column, 0-10% acetone/DCM) to afford the title compound as an off-white solid (1.05 g, 86% yield).

^{1}H NMR (500 MHz, $CDCl_3$) δ 7.92 (s, 2H), 7.85 – 7.79 (m, 2H), 7.79 – 7.68 (m, 4H), 7.62 – 7.51 (m, 9H), 4.92 (s, 2H), 4.86 – 4.79 (m, 1H), 1.91 – 1.81 (m, 2H), 1.46 – 1.30 (m, 4H), 1.29 – 1.20 (m, 2H).

^{13}C NMR (126 MHz, $CDCl_3$) δ 157.36, 156.02, 133.99, 132.92, 132.43, 132.35, 131.06, 129.87, 129.19, 129.11, 128.24, 126.44, 125.76, 59.71, 26.36, 24.97, 21.93, 21.61.

^{19}F NMR (376 MHz, $CDCl_3$) δ -153.46 (minor, $^{11}BF_4$), -153.51 (major, $^{10}BF_4$).

IR (film) ν_{max} 3062, 2931, 2833, 1619, 1599, 1561, 1495, 1049, 1034, 999, 911, 891, 782, 765, 726, 700, 647 cm^{-1}.

HRMS (ESI-TOF) *m/z* calculated for $C_{30}H_{28}N^+$ ($[M–BF_4]^+$) 402.2216, found 402.2223.

1-((6-chloropyridin-3-yl)methyl)-2,4,6-triphenylpyridin-1-ium tetrafluoroborate:

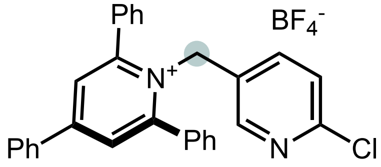

Prepared according to **General Procedure PA** using 2,4,6-triphenylpyrylium tetrafluoroborate (4.95 g, 12.5 mmol, 1.0 equiv) and (6-chloropyridin-3-yl)methanamine (2.14 g, 15.0 mmol, 1.2 equiv) and obtained as an off-white solid (6.29 g, 97% yield).

Note: isolated with ~0.5 equiv. residual EtOH.

^{1}H NMR (500 MHz, $CDCl_3$) δ 7.97 (s, 2H), 7.84 – 7.78 (m, 2H), 7.72 – 7.66 (m, 4H), 7.64 – 7.50 (m, 10H), 7.47 (d, J = 2.6 Hz, 1H), 7.08 (d, J = 8.3 Hz, 1H), 6.86 (dd, J = 8.3, 2.7 Hz, 1H), 5.83 (s, 2H).

^{13}C NMR (126 MHz, $CDCl_3$) δ 157.56, 157.20, 151.74, 147.52, 137.48, 133.70, 132.83, 132.55, 131.68, 130.04, 129.74, 129.25, 128.92, 128.36, 126.95, 124.63, 55.32.

^{19}F NMR (376 MHz, $CDCl_3$) δ -152.67 (minor, $^{11}BF_4$), -152.72 (major, $^{10}BF_4$).

IR (film) ν_{max} 3564, 3000, 1616, 1562, 1454, 1164, 1030, 892, 761, 724, 540 cm^{-1}.

HRMS (ESI-TOF) *m/z* calculated for $C_{29}H_{22}ClN_2^+$ ($[M–BF_4]^+$) 433.1466, found 433.1465.

1-((6-bromopyridin-3-yl)methyl)-2,4,6-triphenylpyridin-1-ium tetrafluoroborate:

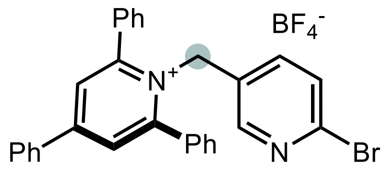

Prepared according to **General Procedure PA** using 2,4,6-triphenylpyrylium tetrafluoroborate (990 mg, 2.5 mmol, 1.0 equiv) and (6-bromopyridin-3-yl)methanamine (561 mg, 3.0 mmol, 1.2 equiv) and obtained as an off-white solid (1.37 g, 93% yield).

Note: isolated with 3% residual EtOH.

^{1}H NMR (500 MHz, $CDCl_3$) δ 8.00 (s, 2H), 7.86 – 7.80 (m, 2H), 7.73 – 7.67 (m, 4H), 7.65 – 7.52 (m, 9H), 7.49 (dd, J = 2.7, 0.8 Hz, 1H), 7.23 (dd, J = 8.3, 0.8 Hz, 1H), 6.72 (dd, J = 8.3, 2.7 Hz, 1H), 5.83 (s, 2H).

^{13}C NMR (126 MHz, $CDCl_3$) δ 157.30, 157.07, 147.99, 142.08, 137.22, 133.77, 132.60 (2C), 131.55, 129.88, 129.59, 129.23, 129.17, 128.55, 128.35, 126.98, 55.30.

Note: One carbon signal overlapped at δ 132.60 ppm

^{19}F NMR (376 MHz, $CDCl_3$) δ -152.70 (minor, $^{11}BF_4$), -152.75 (major, $^{10}BF_4$).

IR (film) ν_{max} 3060, 1620, 1599, 1582, 1561, 1496, 1454, 1417, 1340, 1162, 1082, 1050, 999, 911, 892, 788, 766, 725, 701, 649 cm^{-1}.

HRMS (ESI-TOF) *m/z* calculated for $C_{29}H_{22}BrN_2^+$ ([M–BF_4]$^+$) 477.0961, found 477.0967.

1-((2-fluoropyridin-3-yl)methyl)-2,4,6-triphenylpyridin-1-ium tetrafluoroborate:

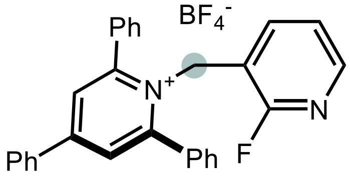

Prepared according to **General Procedure PA** using 2,4,6-triphenylpyrylium tetrafluoroborate (990 mg, 2.5 mmol, 1.0 equiv) and (2-fluoropyridin-3-yl)methanamine (378 mg, 3.0 mmol, 1.2 equiv). The crude solids were purified by automated flash column chromatography (50 g silica column, 0-20% acetone/DCM) to afford the title compound as a light-yellow solid (937 mg, 74% yield).

^{1}H NMR (500 MHz, $CDCl_3$) δ 7.98 – 7.95 (m, 3H), 7.86 – 7.80 (m, 2H), 7.76 – 7.68 (m, 4H), 7.66 – 7.49 (m, 9H), 6.88 (ddd, J = 9.7, 7.4, 2.0 Hz, 1H), 6.81 (ddd, J = 7.3, 4.8, 1.9 Hz, 1H), 5.93 (s, 2H).

^{13}C NMR (126 MHz, $CDCl_3$) δ 160.25 (d, J = 238.2 Hz), 157.72, 156.91, 147.67 (d, J = 15.2 Hz), 140.87 (d, J = 4.1 Hz), 133.75, 132.79, 132.64, 131.45, 129.94, 129.61, 129.18, 128.30, 126.64, 122.09 (d, J = 4.2 Hz), 116.23 (d, J = 28.5 Hz), 54.07 (d, J = 2.8 Hz).

^{19}F NMR (376 MHz, $CDCl_3$) δ -70.77 (d, J = 9.7 Hz, 1F), -152.43 (minor, $^{11}BF_4$), -152.48 (major, $^{10}BF_4$).

IR (film) ν_{max} 3067, 1620, 1578, 1563, 1496, 1440, 1417, 1251, 1161, 1053, 999, 912, 788, 766, 728, 701, 647 cm^{-1}.

HRMS (ESI-TOF) *m/z* calculated for $C_{29}H_{22}FN_2^+$ ([M–BF_4]$^+$) 417.1762, found 417.1769.

1-((6-methoxypyridin-3-yl)methyl)-2,4,6-triphenylpyridin-1-ium tetrafluoroborate:

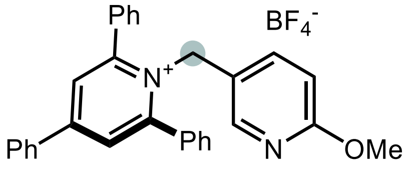

Prepared according to **General Procedure PA** using 2,4,6-triphenylpyrylium tetrafluoroborate (990 mg, 2.5 mmol, 1.0 equiv) and (6-methoxypyridin-3-yl)methanamine (415 mg, 3.0 mmol, 1.2 equiv) and obtained as an off-white solid (316 mg, 24% yield).

^{1}H NMR (500 MHz, $CDCl_3$) δ 7.96 (s, 2H), 7.84 – 7.79 (m, 2H), 7.75 – 7.66 (m, 4H), 7.64 – 7.50 (m, 9H), 7.24 (d, *J* = 2.6 Hz, 1H), 6.63 (dd, *J* = 8.6, 2.6 Hz, 1H), 6.45 (d, *J* = 8.6 Hz, 1H), 5.78 (s, 2H), 3.83 (s, 3H).

^{13}C NMR (126 MHz, $CDCl_3$) δ 164.01, 157.50, 156.58, 145.32, 137.35, 133.77, 132.87, 132.58, 131.39, 129.94, 129.53, 129.31, 128.26, 126.79, 122.63, 111.27, 55.71, 53.73.

^{19}F NMR (376 MHz, $CDCl_3$) δ -153.04 (minor, $^{11}BF_4$), -153.10 (major, $^{10}BF_4$).

IR (film) ν_{max} 3067, 1619, 1564, 1494, 1396, 1286, 1054, 913, 795, 766, 727, 701, 647 cm^{-1}.

HRMS (ESI-TOF) *m/z* calculated for $C_{30}H_{25}N_2O^+$ ($[M–BF_4]^+$) 429.1961, found 429.1967.

1-((2-methoxypyridin-3-yl)methyl)-2,4,6-triphenylpyridin-1-ium tetrafluoroborate:

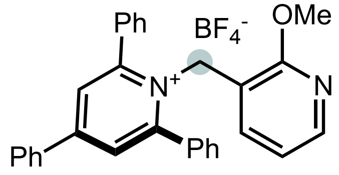

Prepared according to **General Procedure PB** using (2-methoxypyridin-3-yl)methanamine (483.6 mg, 3.5 mmol, 1.0 equiv.), 2,4,6-triphenylpyrylium tetrafluoroborate (1.39 g, 3.5 mmol, 1.0 equiv), powdered activated 4Å molecular sieves (1.75 g), Et_3N (488 μL, 3.5 mmol, 1.0 equiv.), and AcOH (400 μL, 7.0 mmol, 2.0 equiv.) and obtained as an off-white solid (1.45 g, 80% yield).

^{1}H NMR (500 MHz, $CDCl_3$) δ 7.95 – 7.88 (m, 3H), 7.85 – 7.79 (m, 2H), 7.71 (dq, J = 7.2, 2.9 Hz, 4H), 7.62 – 7.45 (m, 9H), 6.62 (dd, J = 7.3, 1.8 Hz, 1H), 6.53 (dd, J = 7.4, 5.0 Hz, 1H), 5.80 (s, 2H), 3.64 (s, 3H).

^{13}C NMR (126 MHz, $CDCl_3$) δ 160.53, 158.11, 156.19, 146.95, 138.15, 133.97, 133.30, 132.50, 131.18, 129.97, 129.44, 129.30, 128.22, 126.28, 117.00, 116.81, 55.72, 53.46.

^{19}F NMR (376 MHz, $CDCl_3$) δ -152.94 (minor, $^{11}BF_4$), -152.99 (major, $^{10}BF_4$).

IR (film) ν_{max} 3062, 1619, 1587, 1584, 1470, 1413, 1261, 1048, 871, 701, 520 cm^{-1}.

HRMS (ESI-TOF) *m/z* calculated for $C_{30}H_{25}N_2O^+$ ([M–BF_4]$^+$) 429.1961, found 429.1961.

1-((6-methoxypyridin-2-yl)methyl)-2,4,6-triphenylpyridin-1-ium tetrafluoroborate:

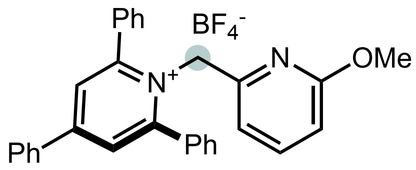

Prepared according to **General Procedure PB** using (6-methoxypyridin-2-yl)methanamine (483.6 mg, 3.5 mmol, 1.0 equiv.), 2,4,6-triphenylpyrylium tetrafluoroborate (1.39 g, 3.5 mmol, 1.0 equiv), powdered activated 4Å molecular sieves (1.75 g), Et_3N (488 μL, 3.5 mmol, 1.0 equiv.), and AcOH (400 μL, 7.0 mmol, 2.0 equiv.) and obtained as a pale-pink solid (1.45 g, 80% yield).

^{1}H NMR (500 MHz, $CDCl_3$) δ 7.94 (s, 2H), 7.86 – 7.80 (m, 2H), 7.69 – 7.53 (m, 7H), 7.51 – 7.39 (m, 6H), 7.25 – 7.22 (m, 1H), 6.56 (d, J = 8.1 Hz, 1H), 6.06 (d, J = 7.3 Hz, 1H), 5.75 (s, 2H), 3.68 (s, 3H).

^{13}C NMR (126 MHz, $CDCl_3$) δ 163.91, 157.91, 156.05, 150.56, 139.50, 134.10, 133.10, 132.40, 130.98, 129.95, 129.16, 129.05, 128.20, 126.01, 114.95, 110.24, 58.98, 53.38.

^{19}F NMR (376 MHz, $CDCl_3$) δ -153.25 (minor, $^{11}BF_4$), -153.30 (major, $^{10}BF_4$).

IR (film) ν_{max} 3059, 1620, 1562, 1471, 1414, 1290, 1057, 887, 763, 699, 520 cm^{-1}.

HRMS (ESI-TOF) *m/z* calculated for $C_{30}H_{25}N_2O^+$ ($[M–BF_4]^+$) 429.1961, found 429.1960.

1-((2-methoxypyridin-4-yl)methyl)-2,4,6-triphenylpyridin-1-ium tetrafluoroborate:

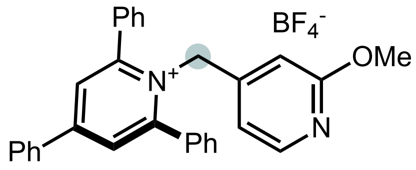

Prepared according to **General Procedure PB** using (2-methoxypyridin-4-yl)methanamine (483.6 mg, 3.5 mmol, 1.0 equiv.), 2,4,6-triphenylpyrylium tetrafluoroborate (1.39 g, 3.5 mmol, 1.0 equiv), powdered activated 4Å molecular sieves (1.75 g), Et_3N (488 μL, 3.5 mmol, 1.0 equiv.), and AcOH (400 μL, 7.0 mmol, 2.0 equiv.) and obtained as a pale-yellow solid (1.45 g, 86% yield).

^{1}H NMR (500 MHz, $CDCl_3$) δ 7.99 (s, 2H), 7.90 (d, J = 5.3 Hz, 1H), 7.86 – 7.80 (m, 2H), 7.71 (d, J = 7.4 Hz, 4H), 7.63 – 7.44 (m, 9H), 6.07 (dd, J = 5.3, 1.6 Hz, 1H), 5.88 (s, 1H), 5.74 (s, 2H), 3.85 (s, 3H).

^{13}C NMR (126 MHz, $CDCl_3$) δ 164.47, 157.76, 156.90, 147.74, 146.01, 133.79, 132.74, 132.42, 131.39, 130.02, 129.44, 129.26, 128.34, 126.70, 114.30, 108.00, 57.11, 53.78.

^{19}F NMR (376 MHz, $CDCl_3$) δ -153.07 (minor, $^{11}BF_4$), -153.08 (major, $^{10}BF_4$).

IR (film) v_{max} 3061, 1622, 1565, 1485, 1449, 1401, 1314, 1158, 1059, 892, 762, 703, 522 cm^{-1}.

HRMS (ESI-TOF) *m/z* calculated for $C_{30}H_{25}N_2O^+$ ($[M–BF_4]^+$) 429.1961, found 429.1962.

1-((2-methoxypyrimidin-5-yl)methyl)-2,4,6-triphenylpyridin-1-ium tetrafluoroborate:

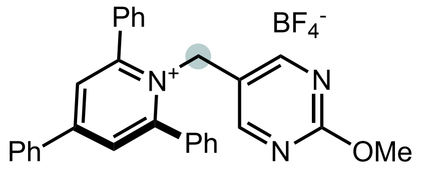

Prepared according to **General Procedure PB** using (2-methoxypyrimidin-5-yl)methanaminium chloride (439.0 mg, 2.5 mmol, 1.0 equiv.), 2,4,6-triphenylpyrylium tetrafluoroborate (990.5 mg, 2.5 mmol, 1.0 equiv), powdered activated 4Å molecular sieves (1.25 g), Et_3N (697 µL, 5.0 mmol, 2.0 equiv.), and AcOH (286 µL, 5.0 mmol, 2.0 equiv.).

After work-up according to **General Procedure PB**, the crude residue was purified by automated flash column chromatography (100 g silica column, 0-50% acetone/DCM) to afford the title compound as an off-white solid (514.0 mg, 40% yield).

^{1}H NMR (500 MHz, $CDCl_3$) δ 7.97 (s, 2H), 7.87 – 7.72 (m, 6H), 7.67 – 7.48 (m, 11H), 5.85 (s, 2H), 3.91 (s, 3H).

^{13}C NMR (126 MHz, $CDCl_3$) δ 165.38, 158.24, 157.50, 157.01, 133.65, 132.85, 132.67, 131.86, 130.07, 129.93, 129.43, 128.30, 126.91, 121.27, 55.43, 53.24.

^{19}F NMR (376 MHz, $CDCl_3$) δ -152.81 (minor, $^{11}BF_4$), -152.86 (major, $^{10}BF_4$).

IR (film) ν_{max} 3060, 1619, 1600, 1558, 1473, 1409, 1329, 1024, 766, 709, 580 cm^{-1}.

HRMS (ESI-TOF) *m/z* calculated for $C_{29}H_{24}N_3O^+$ ($[M–BF_4]^+$) 430.1914, found 430.1919.

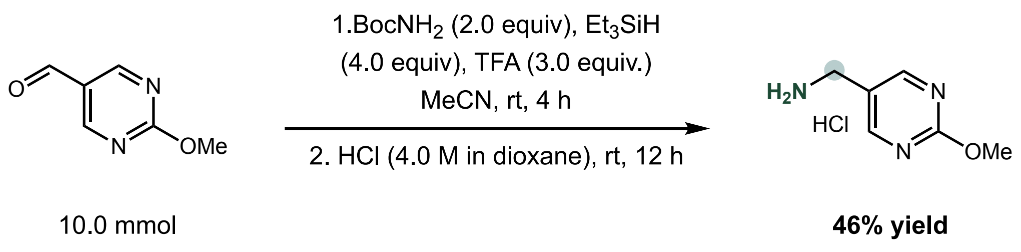

The amine HCl salt starting material was prepared according to a modified literature procedure.[9] To an oven-dried 100-mL round-bottom flask was added 2-methoxypyrimidine-5-carbaldehyde (1.38 g, 10.0 mmol, 1.0 equiv.) and *tert*-butyl carbamate (2.34 g, 20.0 mmol, 2.0 equiv.). MeCN (40 mL) was added to dissolve the starting material and reagents. The solution was cooled to 0 °C in an ice bath, followed by sequential addition of Et_3SiH (6.4 mL, 40.0 mmol, 4.0 equiv.) and TFA (2.3 mL, 30.0 mmol, 3.0 equiv.) via syringe. The reaction was then warmed to room temperature and stirred for 4 hours. After complete consumption of the starting material, the reaction was quenched with NH_4OH (10 mL), concentrated under reduced pressure, and partitioned between EtOAc (60 mL) and saturated aqueous Na_2CO_3 solution (60 mL). The aqueous layer was extracted with EtOAc (3 × 40 mL), and the combined organic layer was washed with brine (3 × 40 mL), dried over Na_2SO_4, and concentrated *in vacuo*. The crude residue was then purified by automated flash column chromatography (100-g silica column, 0-100% EtOAc/hexanes) to afford the carbamate intermediate, which was then dissolved in dioxane (10 mL) and transferred into a 100-mL round-bottom flask equipped with a magnetic stir bar. HCl (10 mL, 4.0 M in dioxane) was added in one portion, and the mixture was stirred at room temperature for 16 h. The reaction was then concentrated via rotary evaporation and dried under high vacuum for 1 h to afford analytically pure (2-methoxypyrimidin-5-yl)methanaminium chloride as a white solid (1.76 g, 46% yield over two steps), which was then converted to the corresponding pyridinium salt as described above.

^{1}H NMR (300 MHz, D_2O) δ 8.69 (s, 2H), 4.23 (s, 2H), 4.04 (s, 3H).

[9] Bolt Biotherapeutics, Inc. Anti-HER2 Immunoconjugates, and Uses Thereof. WO Patent 2022/125904, 2022.

1-((6-chloro-1-isopropyl-1*H*-pyrazolo[3,4-*b*]pyridin-5-yl)methyl)-2,4,6-triphenylpyridin-1-ium tetrafluoroborate:

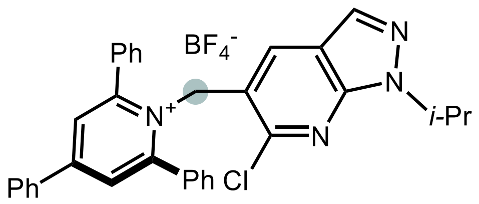

Prepared according to **General Procedure PA** using 2,4,6-triphenylpyrylium tetrafluoroborate (911 mg, 2.30 mmol, 1.0 equiv) and (6-chloro-1-isopropyl-1*H*-pyrazolo[3,4-*b*]pyridin-5-yl)methanamine (639 mg, 2.76 mmol, 1.2 equiv). The crude solids were purified by automated flash column chromatography (25 g silica column, 0-20% acetone/DCM) to afford the title compound as an off-white solid (978 mg, 69% yield).

^{1}H NMR (500 MHz, CDCl$_3$) δ 7.99 (s, 2H), 7.94 – 7.82 (m, 3H), 7.72 – 7.65 (m, 4H), 7.65 – 7.55 (m, 3H), 7.53 – 7.42 (m, 7H), 6.00 (s, 2H), 5.08 (hept, *J* = 6.7 Hz, 1H), 1.51 (d, *J* = 6.7 Hz, 6H).

^{13}C NMR (126 MHz, CDCl$_3$) δ 157.80, 156.96, 147.43, 147.02, 133.94, 132.95, 132.90, 132.49, 131.44, 129.85 (2C), 129.46, 129.08, 128.40, 127.00, 120.35, 114.65, 57.42, 49.02, 22.07.

Note: One carbon signal overlapped at δ 129.85 ppm

^{19}F NMR (376 MHz, CDCl$_3$) δ -152.20 (minor, $^{11}BF_4$), -152.26 (major, $^{10}BF_4$).

IR (film) ν_{max} 3067, 2980, 1621, 1558, 1494, 1410, 1188, 1054, 913, 764, 729, 702 cm^{-1}.

HRMS (ESI-TOF) *m/z* calculated for $C_{33}H_{28}ClN_4^+$ ([M–BF$_4$]$^+$) 515.1997, found 515.2000.

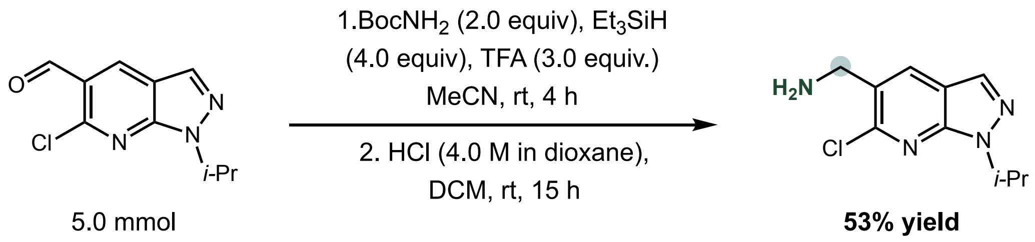

The amine starting material was prepared according to a modified literature procedure.[10] To an oven-dried 100-mL round-bottom flask was added 6-chloro-1-isopropyl-1*H*-pyrazolo[3,4-*b*]pyridine-5-carbaldehyde (1.12 g, 5.0 mmol, 1.0 equiv.) and *tert*-butyl carbamate (1.17 g, 10.0 mmol, 2.0 equiv.). MeCN (20 mL) was added to dissolve the starting material and reagents. The solution was cooled to 0 °C in an ice bath, followed by sequential addition of Et_3SiH (3.2 mL, 20.0 mmol, 4.0 equiv.) and TFA (1.15 mL, 15.0 mmol, 3.0 equiv.) via syringe. The reaction was then warmed to room temperature and stirred for 4 hours. After complete consumption of the starting material, the reaction was quenched with 6M aqueous NaOH (5 mL), concentrated under reduced pressure, and partitioned between EtOAc (20 mL) and water (20 mL). The aqueous layer was extracted with EtOAc (2 × 5 mL), and the combined organic layer was washed with brine (20 mL), dried over Na_2SO_4, and concentrated under reduced pressure. The crude residue was then purified by automated flash column chromatography (25g silica column, 0-25-100% EtOAc/hexanes) to afford the carbamate intermediate. A 50-mL round-bottom flask equipped with a magnetic stirring bar and septum was charged with the intermediate, which was then dissolved in DCM (4 mL). HCl (4 mL, 4.0 M in dioxane) was added in one portion, and the mixture was stirred overnight at room temperature. The precipitated solid was triturated with Et_2O (10 mL) and collected by filtration. The solid was dissolved in water and basified with 6M aqueous NaOH. The aqueous layer was extracted with 10% MeOH/DCM (3 × 20 mL), and the combined extracts were dried over Na_2SO_4, and concentrated under reduced pressure. The residue was dried under high vacuum for 1 h to afford analytically pure (6-chloro-1-isopropyl-1*H*-pyrazolo[3,4-*b*]pyridin-5-yl)methanamine as a yellow solid (621 mg, 53% yield over two steps), which was then converted to the corresponding pyridinium salt as described above.

[10] Ibid.

^{1}H NMR (400 MHz, $CDCl_3$) δ 8.08 (s, 1H), 7.98 (s, 1H), 5.29 – 5.19 (m, 1H), 4.03 (s, 2H), 1.57 (d, *J* = 6.8 Hz, 6H, *overlapped with H_2O peak*).

1-((2-(2,6-dioxopiperidin-3-yl)-1-oxoisoindolin-5-yl)methyl)-2,4,6-triphenylpyridin-1-ium tetrafluoroborate:

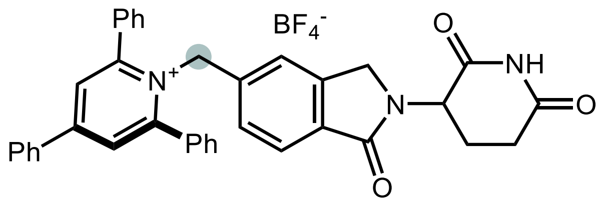

Prepared according to **General Procedure PB** using 3-(5-(aminomethyl)-1-oxoisoindolin-2-yl)piperidine-2,6-dione hydrochloride (599 mg, 1.90 mmol, 1.05 equiv.), 2,4,6-triphenylpyrylium tetrafluoroborate (714 mg, 1.8 mmol, 1.0 equiv), powdered activated 4Å molecular sieves (950 mg), Et_3N (502 μL, 3.6 mmol, 2.0 equiv.), and AcOH (206 μL, 3.6 mmol, 2.0 equiv.).

After work-up according to **General Procedure PB**, the crude residue was purified by automated flash column chromatography (50 g silica column, 0-60-80-100% acetone/DCM) to afford the title compound as an off-white solid (349 mg, 30% yield).

^{1}H NMR (500 MHz, Acetone-*d*$_6$) δ 9.74 (s, 1H), 8.53 (s, 2H), 8.29 – 8.23 (m, 2H), 7.80 – 7.74 (m, 4H), 7.72 – 7.59 (m, 5H), 7.57 – 7.53 (m, 4H), 7.48 (d, *J* = 7.9 Hz, 1H), 6.99 (s, 1H), 6.88 (dd, *J* = 8.0, 1.6 Hz, 1H), 6.03 (s, 2H), 5.12 (dd, *J* = 13.4, 5.1 Hz, 1H), 4.34 (d, *J* = 17.1 Hz, 1H), 4.27 (d, *J* = 17.0 Hz, 1H), 2.94 (ddd, *J* = 17.5, 13.7, 5.5 Hz, 1H), 2.77 – 2.68 (m, 1H), 2.48 (qd, *J* = 13.3, 4.5 Hz, 1H), 2.15 (dtd, *J* = 12.9, 5.4, 2.4 Hz, 1H).

^{13}C NMR (126 MHz, Acetone-*d*$_6$) δ 172.72, 171.23, 168.49, 158.42, 157.27, 143.85, 138.97, 134.63, 134.07, 133.53, 132.87, 131.99, 130.66, 130.16, 130.05, 129.61, 127.72, 127.10, 124.35, 122.75, 59.25, 52.92, 47.88, 32.19, 23.81.

^{19}F NMR (376 MHz, Acetone-*d*$_6$) δ -151.71 (minor, $^{11}BF_4$), -151.77 (major, $^{10}BF_4$).

IR (film) ν_{max} 3201, 3065, 1689, 1620, 1599, 1562, 1454, 1415, 1366, 1347, 1249, 1232, 1197, 1054, 1035, 999, 788, 766, 729, 702, 682 cm^{-1}.

HRMS (ESI-TOF) *m/z* calculated for $C_{37}H_{30}N_3O_3^+$ ($[M–BF_4]^+$) 564.2282, found 564.2286.

1-((5-bromo-2-fluoropyridin-3-yl)methyl)-2,4,6-triphenylpyridin-1-ium tetrafluoroborate:

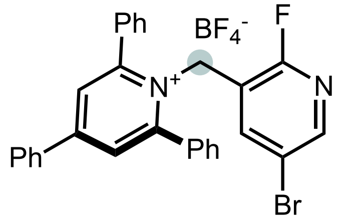

Prepared according to **General Procedure PB** using (5-bromo-2-fluoropyridin-3-yl)methanaminium hydrochloride (362.2 mg, 1.5 mmol, 1.0 equiv.), 2,4,6-triphenylpyrylium tetrafluoroborate (594.3 mg, 1.5 mmol, 1.0 equiv), powdered activated 4Å molecular sieves (500 mg), Et_3N (418 µL, 3.0 mmol, 2.0 equiv.), and AcOH (172 µL, 3.0 mmol, 2.0 equiv.) and obtained as a light-brown solid (742.0 mg, 85% yield).

^{1}H NMR (500 MHz, $CDCl_3$) δ 8.01 – 7.93 (m, 3H), 7.88 – 7.77 (m, 6H), 7.66 – 7.48 (m, 9H), 6.81 (dd, *J* = 8.5, 2.4 Hz, 1H), 6.02 (s, 2H).

^{13}C NMR (126 MHz, $CDCl_3$) δ 159.69 (d, *J* = 238.9 Hz), 158.02, 156.87, 148.41 (d, *J* = 15.9 Hz), 143.77 (d, *J* = 4.8 Hz), 133.62, 132.88, 132.75, 131.70, 130.09, 129.82, 129.50, 128.86, 126.36, 118.07 (d, *J* = 30.5 Hz), 116.91 (d, *J* = 4.4 Hz), 54.63 (d, *J* = 3.7 Hz).

^{19}F NMR (376 MHz, $CDCl_3$) δ -73.38 (d, *J* = 8.6 Hz), -152.53 (minor, $^{11}BF_4$), -152.54 (major, $^{10}BF_4$).

IR (film) ν_{max} 3059, 1618, 1559, 1441, 1249, 1161, 1050, 892, 767, 600, 632, 518 cm^{-1}.

HRMS (ESI-TOF) *m/z* calculated for $C_{29}H_{21}BrFN_2O^+$ ([M–BF_4]$^+$) 495.0867, found 495.0868.

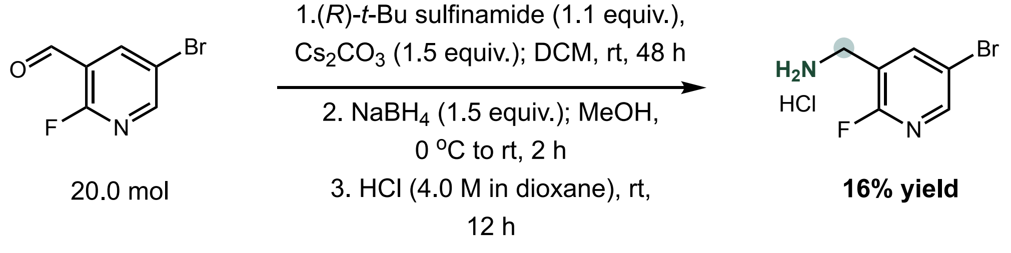

The amine HCl salt starting material was prepared according to the following procedure. In a 250-mL round-bottom flask, 5-bromo-2-fluoro-pyridine-3-carbaldehyde (4.08 g, 20 mmol, 1.0 equiv.) was dissolved in DCM (50.0 mL). (*R*)-*tert*-butyl sulfinamide (2.67 g, 22.00 mmol, 1.1 equiv.) and Cs_2CO_3 (9.78 g, 30.0 mmol, 1.5 equiv.) were added in one portion, and the reaction mixture was stirred at room temperature for 48 h. The heterogenous mixture was then filtered through a plug of Celite, and the filter cake was washed with MeOH (3 × 25 mL). The combined filtrate was then concentrated under reduced pressure to afford the crude sulfinimine, which was used for the next step without further purification.

The crude sulfinimine was transferred into a 250-mL round-bottom flask, re-dissolved in anhydrous MeOH (50.0 mL), and cooled to 0 °C in an ice bath. Sodium borohydride (1.13 g, 30.00 mmol, 1.5 equiv.) was added portionwise over 5 min, and the mixture was stirred for an additional 2 h. The reaction was quenched by addition of a small amount of acetone and concentrated under reduced pressure. The crude residue was partitioned between EtOAc (100 mL) and saturated aqueous NH_4Cl (100 mL) and transferred to a separatory funnel, and the layers were separated. The aqueous layer was extracted three times with EtOAc, and the combined extracts were dried, concentrated under reduced pressure, and purified by automated flash column chromatography (100 g silica column, 0-100% EtOAc/hexanes).

Fractions containing the sulfonamide product was then collected and concentrated, and the residue was transferred into a 100-mL round-bottom flask dissolved in dioxane (10.0 mL). HCl (4.0 M in dioxane, 15.0 mL) was added via syringe, and the mixture was stirred at room temperature for 16 hours. The reaction was concentrated under reduced pressure, and the solids were dried under high vacuum to afford (5-bromo-2-fluoropyridin-3-yl)methanaminium

hydrochloride as an off-white solid (749.9 mg, 16% yield), which was then converted to the corresponding pyridinium salt as described above.

^{1}H NMR (500 MHz, D_2O) δ 8.30 (s, 1H), 8.17 (d, *J* = 8.3 Hz, 1H), 4.20 (s, 2H).

7. General Procedures for Deaminative C(sp^3)—C(sp^3) Cross-Couplings

General Procedure A: Deaminative—decarboxylative C(sp^3)—C(sp^3) Cross-Coupling

To an oven-dried 40-mL scintillation vial with a cross stir bar was added carboxylic acid (0.5 mmol), benzylamine-derived pyridinium salt (1.5 equiv.), K_2CO_3 (1.5 equiv. or 2.5 equiv.), 4CzIPN (5.0 mol%), and Fe(OEP)Cl (5.0 mol%). Next, the vial was evacuated and back-filled three times with N_2, after which DMA (5.0 mL) and *i*-PrOH (5.0 mL) were added via syringe. The reaction mixture was sparged with N_2 at room temperature for 10 mins while stirring (~200 rpm). The vial was then sealed with melted parafilm, wrapped with electric tape, and placed in a PennPhD m1 integrated photoreactor. The reaction was irradiated with 450 nm blue LEDs (75% light intensity, 5200 rpm fans, 1500 rpm stirring) for 24 hours.

After irradiation, the reaction mixture was transferred into a 100-mL round-bottom flask and concentrated in vacuo to remove *i*-PrOH. The resultant crude was then poured into 50 mL of 10 wt% aqueous LiCl solution and transferred to a separatory funnel. The aqueous layer was extracted with EtOAc (3 × 30 mL), and the combined extracts were washed with brine (3 × 25 mL), dried over Na_2SO_4, filtered, and concentrated *in vacuo*. The residue was purified by automated normal-phase flash column chromatography and reverse-phase column chromatography to afford the desired coupled product.

General Procedure B: Deaminative—deoxygenative C(sp^3)—C(sp^3) Cross-Coupling

Alcohol activation. To an oven-dried 40 mL vial equipped with a stir bar was added alcohol (1.0 mmol, 2.0 equiv. with respect to the pyridinium salt) and **NHC** (2.2 equiv.). Next, the vial was evacuated and back-filled three times with N_2, after which $PhCF_3$ (10.0 mL) was added via syringe (if the alcohol is a liquid, it is added at this stage instead as a stock solution in 5.0 mL $PhCF_3$ into the suspension of **NHC** in 5.0 mL $PhCF_3$). The vial was then cooled to −25 °C in a dry-ice/*i*-PrOH bath. At this point, pyridine (2.15 equiv.) was added dropwise. The resulting suspension was allowed to warm to 0 °C over two hours with stirring. The resulting orange-red suspension was then syringe-filtered to remove pyridinium salts and unreacted **NHC**, and the dark-red solution was concentrated under reduced pressure by rotary evaporator, followed by vacuum for 5 min. (*It is critical that the activated adduct be placed under vacuum for no longer than 5 minutes to prevent premature decomposition.*) The resulting foamy residue was dissolved in a 1:1 mixture of acetone (5.0 mL) and *i*-PrOH (5.0 mL). Sonication was applied if necessary to aid dissolution.

Reaction set-up. To an oven-dried 40 mL vial equipped with a cross stir bar was added pyridinium salt (0.5 mmol, 1.0 equiv.), KOPiv (4.0 equiv.), Ir[(dFCF$_3$ppy)$_2$(dtbbpy)]PF$_6$ (1.5 mol%), and Fe(OEP)Cl (2.5 mol%). The vial was placed under nitrogen atmosphere, and the pre-activated alcohol-NHC adduct was added via syringe. The reaction mixture was sparged with N_2 at room temperature for 10 mins while stirring (~200 rpm). The vial was then sealed with melted parafilm, wrapped with electric tape, and placed in a PennPhD m1 integrated photoreactor. The reaction was irradiated with 450 nm blue LEDs (75% light intensity, 5200 rpm fans, 1500 rpm stirring) for 2 hours, after which the reaction mixture was concentrated *in vacuo*. The residue was

purified by automated normal-phase flash column chromatography and reverse-phase column chromatography to afford the desired coupled product.

8. Isolation and Characterization of Cross-coupled Products

***tert*-butyl 4-(4-(methoxycarbonyl)benzyl)-4-methylpiperidine-1-carboxylate (1):**

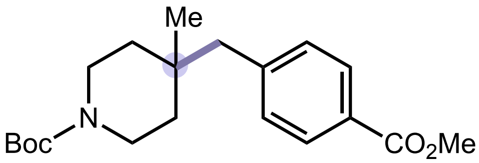

Prepared according to **General Procedure A** using 1-(*tert*-butoxycarbonyl)-4-methylpiperidine-4-carboxylic acid (121.7 mg, 0.5 mmol, 1.0 equiv.), 1-(4-(methoxycarbonyl)benzyl)-2,4,6-triphenylpyridin-1-ium tetrafluoroborate (407.5 mg, 0.75 mmol, 1.5 equiv.), K_2CO_3 (172.8 mg, 1.25 mmol, 2.5 equiv.), 4CzIPN (19.7 mg, 25.0 μmol, 5.0 mol%), Fe(OEP)Cl (15.6 mg, 25.0 μmol, 5.0 mol%), DMA (5.0 mL), and *i*-PrOH (5.0 mL).

After work-up according to **General Procedure A**, the crude residue was purified by automated flash column chromatography (2 × 25 g silica column, 0-30% EtOAc/hexanes) followed by automated reverse-phase column chromatography (30 g C18 column, 55-80% MeCN/H_2O with 0.1% NH_4OH) to afford the title compound as a light-yellow oil (145.9 mg, 84% yield).

^{1}H NMR (500 MHz, $CDCl_3$) δ 7.93 (d, *J* = 8.2 Hz, 2H), 7.16 (d, *J* = 8.4 Hz, 2H), 3.89 (s, 3H), 3.68 (dt, *J* = 13.7, 4.8 Hz, 2H), 3.12 (ddd, *J* = 13.5, 10.1, 3.4 Hz, 2H), 2.60 (s, 2H), 1.44 (s, 9H), 1.38 – 1.49 (m, 2H), 1.31 – 1.22 (m, 2H), 0.89 (s, 3H).

^{13}C NMR (126 MHz, $CDCl_3$) δ 167.20, 155.06, 143.70, 130.74, 129.19, 128.20, 79.45, 52.10, 48.73, 39.95, 36.72, 33.02, 28.56, 22.88.

IR (film) ν_{max} 2927, 1720, 1687, 1416, 1274, 1156, 1098, 1020, 860, 770, 719 cm^{-1}.

HRMS (ESI-TOF) *m/z* calculated for $C_{16}H_{22}NO_4^+$ ([M+H–isobutylene]$^+$) 292.1543, found 292.1545.

***tert*-butyl 4-methyl-4-(4-(trifluoromethyl)benzyl)piperidine-1-carboxylate (2):**

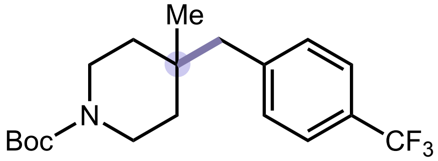

Prepared according to **General Procedure A** using 1-(*tert*-butoxycarbonyl)-4-methylpiperidine-4-carboxylic acid (121.7 mg, 0.5 mmol, 1.0 equiv.), 2,4,6-triphenyl-1-(4-(trifluoromethyl)benzyl)pyridin-1-ium tetrafluoroborate (415.0 mg, 0.75 mmol, 1.5 equiv.), K_2CO_3 (172.8 mg, 1.25 mmol, 2.5 equiv.), 4CzIPN (19.7 mg, 25.0 μmol, 5.0 mol%), Fe(OEP)Cl (15.6 mg, 25.0 μmol, 5.0 mol%), DMA (5.0 mL), and *i*-PrOH (5.0 mL).

After work-up according to **General Procedure A**, the crude residue was purified by automated flash column chromatography (2 × 25 g silica column, 0-50% Et_2O/hexanes) followed by automated reverse-phase column chromatography (30 g C18 column, 65-80% MeCN/H_2O with 0.1% NH_4OH) to afford the title compound as a light-yellow oil (137.2 mg, 77% yield).

^{1}H NMR (500 MHz, $CDCl_3$) δ 7.52 (d, *J* = 8.0 Hz, 2H), 7.21 (d, *J* = 8.0 Hz, 2H), 3.71 (dt, *J* = 13.8, 4.7 Hz, 2H), 3.12 (ddd, *J* = 13.6, 10.2, 3.4 Hz, 2H), 2.61 (s, 2H), 1.45 (s, 9H), 1.49 – 1.37 (m, 2H), 1.33 – 1.23 (m, 2H), 0.90 (s, 3H).

^{13}C NMR (126 MHz, $CDCl_3$) δ 155.10, 142.30 (q, *J* = 1.4 Hz), 130.99, 128.59 (q, *J* = 32.4 Hz), 124.82 (q, *J* = 3.8 Hz), 124.45 (q, *J* = 271.8 Hz), 79.51, 48.64, 39.97, 36.68, 32.97, 28.57, 22.75.

^{19}F NMR (471 MHz, $CDCl_3$) δ -62.34.

IR (film) ν_{max} 2973, 2928, 1688, 1417, 1323, 1246, 1157, 1120, 1019, 850, 770 cm^{-1}.

HRMS (ESI-TOF) *m/z* calculated for $C_{15}H_{19}F_3NO_2^+$ ($[M+H–isobutylene]^+$) 302.1362, found 302.1367.

***tert*-butyl 4-(4-cyanobenzyl)-4-methylpiperidine-1-carboxylate (3):**

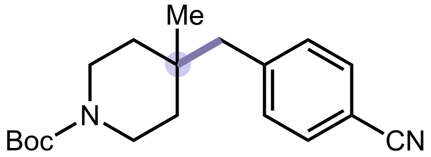

Prepared according to **General Procedure A** using 1-(*tert*-butoxycarbonyl)-4-methylpiperidine-4-carboxylic acid (121.7 mg, 0.5 mmol, 1.0 equiv.), 1-(4-cyanobenzyl)-2,4,6-triphenylpyridin-1-ium tetrafluoroborate (382.8 mg, 0.75 mmol, 1.5 equiv.), K_2CO_3 (172.8 mg, 1.25 mmol, 2.5 equiv.), 4CzIPN (19.7 mg, 25.0 μmol, 5.0 mol%), Fe(OEP)Cl (15.6 mg, 25.0 μmol, 5.0 mol%), DMA (5.0 mL), and *i*-PrOH (5.0 mL).

After work-up according to **General Procedure A**, the crude residue was purified by automated flash column chromatography (2 × 25 g silica column, 0-40% EtOAc/hexanes) followed by automated reverse-phase column chromatography (60 g C18 column, 55-80% MeCN/H_2O with 0.1% NH_4OH) to afford the title compound as an off-white solid (108.6 mg, 69% yield).

^{1}H NMR (500 MHz, $CDCl_3$) δ 7.55 (d, *J* = 8.0 Hz, 2H), 7.20 (d, *J* = 8.0 Hz, 2H), 3.70 (dt, *J* = 13.7, 4.7 Hz, 2H), 3.10 (ddd, *J* = 13.6, 10.3, 3.4 Hz, 2H), 2.60 (s, 2H), 1.44 (s, 9H), 1.43 – 1.38 (m, 2H), 1.31 – 1.22 (m, 2H), 0.89 (s, 3H).

^{13}C NMR (126 MHz, $CDCl_3$) δ 155.01, 143.87, 131.69, 131.42, 119.08, 110.18, 79.52, 49.00, 39.87, 36.65, 33.14, 28.54, 22.68.

IR (film) ν_{max} 2972, 2926, 2227, 1685, 1607, 1417, 1246, 1157, 1097, 851, 770, 555 cm^{-1}.

HRMS (ESI-TOF) *m/z* calculated for $C_{15}H_{19}N_2O_2^+$ [M+H–isobutylene] 259.1441, found 259.1448.

***tert*-butyl 4-methyl-4-(4-sulfamoylbenzyl)piperidine-1-carboxylate (4):**

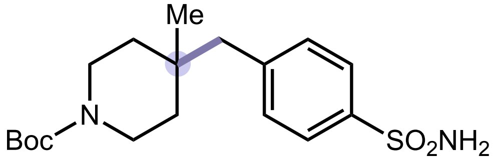

Prepared according to **General Procedure A** using 1-(*tert*-butoxycarbonyl)-4-methylpiperidine-4-carboxylic acid (121.7 mg, 0.5 mmol, 1.0 equiv.), 2,4,6-triphenyl-1-(4-sulfamoylbenzyl)pyridin-1-ium tetrafluoroborate (423.3 mg, 0.75 mmol, 1.5 equiv.), K_2CO_3 (172.8 mg, 1.25 mmol, 2.5 equiv.), 4CzIPN (19.7 mg, 25.0 μmol, 5.0 mol%), Fe(OEP)Cl (15.6 mg, 25.0 μmol, 5.0 mol%), DMA (5.0 mL), and *i*-PrOH (5.0 mL).

After work-up according to **General Procedure A**, the crude residue was purified by automated flash column chromatography (2 × 25 g silica column, 0-75% EtOAc/hexanes) followed by automated reverse-phase column chromatography (30 g C18 column, 40-70% MeCN/H_2O with 0.1% NH_4OH) to afford the title compound as a light-yellow oil (110.9 mg, 60% yield).

^{1}H NMR (500 MHz, $CDCl_3$) δ 7.83 (d, *J* = 8.3 Hz, 2H), 7.24 (d, *J* = 8.3 Hz, 2H), 5.19 (s, 2H), 3.69 (dt, *J* = 14.2, 4.7 Hz, 2H), 3.10 (ddd, *J* = 13.6, 10.1, 3.3 Hz, 2H), 2.61 (s, 2H), 1.49 – 1.36 (m, 11H), 1.31 – 1.22 (m, 2H), 0.90 (s, 3H).

^{13}C NMR (126 MHz, $CDCl_3$) δ 155.09, 143.62, 140.05, 131.31, 126.06, 79.60, 48.65, 39.92, 36.63, 33.06, 28.56, 22.74.

IR (film) ν_{max} 3257, 2973, 2922, 1667, 1528, 1331, 1277, 1157, 1097, 908, 731, 582, 546 cm^{-1}.

HRMS (ESI-TOF) *m/z* calculated for $C_{18}H_{28}N_2NaO_4S^+$ ($[M+Na]^+$) 391.1662, found 391.1665.

***tert*-butyl 4-(4-carbamoylbenzyl)-4-methylpiperidine-1-carboxylate (5):**

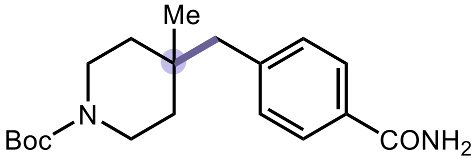

Prepared according to **General Procedure A** using 1-(*tert*-butoxycarbonyl)-4-methylpiperidine-4-carboxylic acid (62.7 mg, 0.25 mmol, 1.0 equiv.), 1-(4-((aminooxy)carbonyl)benzyl)-2,4,6-triphenylpyridin-1-ium tetrafluoroborate (198.1 mg, 0.375 mmol, 1.5 equiv.), K_2CO_3 (86.4 mg, 0.625 mmol, 2.5 equiv.), 4CzIPN (9.9 mg, 12.5 μmol, 5.0 mol%), Fe(OEP)Cl (7.8 mg, 12.5 μmol, 5.0 mol%), DMA (2.5 mL), and *i*-PrOH (2.5 mL).

After work-up according to **General Procedure A**, the crude residue was purified by automated flash column chromatography (50 g silica column, 0-70-80-90% EtOAc/hexanes) to afford the title compound as a light-yellow solid (57.6 mg, 69% yield).

^{1}H NMR (500 MHz, $CDCl_3$) δ 7.72 (d, *J* = 8.2 Hz, 2H), 7.20 (d, *J* = 8.2 Hz, 2H), 6.01 (br-s, 1H), 5.51 (br-s, 1H), 3.87 – 3.56 (m, 2H), 3.13 (ddd, *J* = 13.6, 10.1, 3.4 Hz, 2H), 2.62 (s, 2H), 1.50 – 1.38 (m, 2H), 1.45 (s, 9H), 1.35 – 1.22 (m, 2H), 0.91 (s, 3H).

^{13}C NMR (126 MHz, $CDCl_3$) δ 169.74, 155.06, 142.56, 131.38, 130.88, 127.05, 79.46, 48.58, 40.27, 39.63, 36.65, 32.96, 28.55, 22.85.

IR (film) ν_{max} 3379, 3167, 2974, 2922, 1679, 1653, 1614, 1566, 1416, 1387, 1364, 1350, 1276, 1159, 1124, 1087, 1019, 861, 780, 625, 596, 539 cm^{-1}.

HRMS (ESI-TOF) *m/z* calculated for $C_{19}H_{29}N_2NaO_3^+$ ([M+Na]$^+$) 355.1992, found 355.1997.

***tert*-butyl 4-(4-methoxybenzyl)-4-methylpiperidine-1-carboxylate (6):**

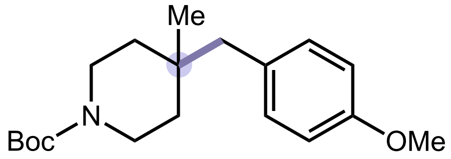

Prepared according to **General Procedure A** using 1-(*tert*-butoxycarbonyl)-4-methylpiperidine-4-carboxylic acid (121.7 mg, 0.5 mmol, 1.0 equiv.), 1-(4-methoxybenzyl)-2,4,6-triphenylpyridin-1-ium tetrafluoroborate (386.5 mg, 0.75 mmol, 1.5 equiv.), K_2CO_3 (172.8 mg, 1.25 mmol, 2.5 equiv.), 4CzIPN (19.7 mg, 25.0 μmol, 5.0 mol%), Fe(OEP)Cl (15.6 mg, 25.0 μmol, 5.0 mol%), DMA (5.0 mL), and *i*-PrOH (5.0 mL).

After work-up according to **General Procedure A**, the crude residue was purified by automated flash column chromatography (2 × 25 g silica column, 0-50% EtOAc/hexanes) followed by automated reverse-phase column chromatography (30 g C18 column, 55-85% MeCN/H_2O with 0.1% NH_4OH) to afford the title compound as a light-yellow oil (93.2 mg, 58% yield).

^{1}H NMR (500 MHz, $CDCl_3$) δ 7.07 – 7.01 (m, 2H), 6.86 – 6.80 (m, 2H), 3.80 (s, 3H), 3.75 – 3.62 (m, 2H), 3.15 (ddd, J = 13.6, 10.0, 3.4 Hz, 2H), 2.52 (s, 2H), 1.47 (s, 9H), 1.46 – 1.39 (m, 2H), 1.33 – 1.26 (m, 2H), 0.90 (s, 3H).

^{13}C NMR (126 MHz, $CDCl_3$) δ 158.10, 155.15, 131.63, 130.15, 113.30, 79.36, 55.30, 47.73, 40.96, 36.60, 32.77, 28.58, 22.94.

IR (film) v_{max} 2928, 1687, 1611, 1511, 1420, 1244, 1035, 836, 757, 530 cm^{-1}.

HRMS (ESI-TOF) *m/z* calculated for $C_{15}H_{22}NO_3^+$ ([M+H–isobutylene]$^+$) 264.1594, found 264.1594.

***tert*-butyl 4-(4-bromobenzyl)-4-methylpiperidine-1-carboxylate (7):**

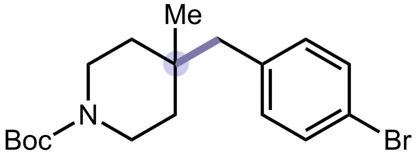

Prepared according to **General Procedure A** using 1-(*tert*-butoxycarbonyl)-4-methylpiperidine-4-carboxylic acid (121.7 mg, 0.5 mmol, 1.0 equiv.), 1-(4-bromobenzyl)-2,4,6-triphenylpyridin-1-ium tetrafluoroborate (423.2 mg, 0.75 mmol, 1.5 equiv.), K_2CO_3 (172.8 mg, 1.25 mmol, 2.5 equiv.), 4CzIPN (19.7 mg, 25.0 μmol, 5.0 mol%), Fe(OEP)Cl (15.6 mg, 25.0 μmol, 5.0 mol%), DMA (5.0 mL), and *i*-PrOH (5.0 mL).

After work-up according to **General Procedure A**, the crude residue was purified by automated flash column chromatography (2 × 25 g silica column, 0-30% Et_2O/hexanes) followed by automated reverse-phase column chromatography (30 g C18 column, 65-95% $MeCN/H_2O$ with 0.1% NH_4OH) to afford the title compound as a light-yellow oil (143.3 mg, 78% yield).

^{1}H NMR (500 MHz, CDCl$_3$) δ 7.38 (d, *J* = 8.4 Hz, 2H), 6.97 (d, *J* = 8.4 Hz, 1H), 3.68 (dt, *J* = 13.7, 4.8 Hz, 2H), 3.11 (ddd, *J* = 13.6, 10.1, 3.4 Hz, 2H), 2.50 (s, 2H), 1.44 (s, 9H), 1.42 – 1.36 (m, 2H), 1.32 – 1.22 (m, 2H), 0.88 (s, 3H).

^{13}C NMR (126 MHz, CDCl$_3$) δ 155.07, 137.04, 132.40, 130.98, 120.16, 79.43, 48.14, 39.96, 36.61, 32.75, 28.57, 22.77.

IR (film) ν_{max} 2971, 2924, 1687, 1476, 1276, 1156, 1011, 768, 533 cm^{-1}.

HRMS (ESI-TOF) *m/z* calculated for $C_{14}H_{19}BrNO_2^+$ ([M+H–isobutylene]$^+$) 312.0594, found 312.0592.

***tert*-butyl 4-methyl-4-(4-(4,4,5,5-tetramethyl-1,3,2-dioxaborolan-2-yl)benzyl)piperidine-1-carboxylate (8):**

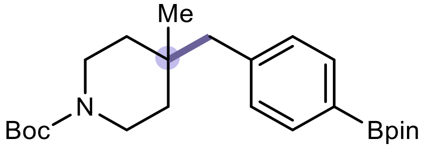

Prepared according to **General Procedure A** using 1-(*tert*-butoxycarbonyl)-4-methylpiperidine-4-carboxylic acid (62.7 mg, 0.25 mmol, 1.0 equiv.), 2,4,6-triphenyl-1-(4-(4,4,5,5-tetramethyl-1,3,2-dioxaborolan-2-yl)benzyl)pyridin-1-ium tetrafluoroborate (229.2 mg, 0.375 mmol, 1.5 equiv.), K_2CO_3 (86.4 mg, 0.625 mmol, 2.5 equiv.), 4CzIPN (9.9 mg, 12.5 μmol, 5.0 mol%), Fe(OEP)Cl (7.8 mg, 12.5 μmol, 5.0 mol%), DMA (2.5 mL), and *i*-PrOH (2.5 mL).

After work-up according to **General Procedure A**, the crude residue was purified by automated flash column chromatography (50 g silica column, 0-10-20-35% EtOAc/hexanes) to afford the title compound as a light yellow solid (67.6 mg, 65% yield).

^{1}H NMR (500 MHz, $CDCl_3$) δ 7.71 (d, *J* = 7.9 Hz, 2H), 7.12 (d, *J* = 7.9 Hz, 2H), 3.68 (dt, *J* = 13.7, 4.8 Hz, 2H), 3.13 (ddd, *J* = 13.5, 9.9, 3.5 Hz, 2H), 2.58 (s, 2H), 1.47 – 1.40 (m, 2H), 1.45 (s, 9H), 1.34 (s, 12H), 1.31 – 1.25 (m, 2H), 0.89 (s, 3H).

^{13}C NMR (126 MHz, $CDCl_3$) δ 155.12, 141.58, 134.39, 130.26, 83.81, 79.36, 48.83, 40.40, 39.65, 36.76, 32.93, 28.60, 25.00, 23.03.

Note: The carbon directly attached to boron was not observed due to quadrupolar relaxation.

IR (film) ν_{max} 2976, 2928, 2869, 1693, 1611, 1422, 1399, 1379, 1361, 1322, 1275, 1262, 1247, 1159, 1146, 1090, 860, 661 cm^{-1}.

HRMS (ESI-TOF) *m/z* calculated for $C_{24}H_{38}BNNaO_4^+$ ($[M+Na]^+$) 438.2786, found 438.2791.

***tert*-butyl 4-(2-chlorobenzyl)-4-methylpiperidine-1-carboxylate (9):**

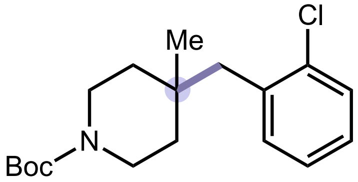

Prepared according to **General Procedure A** using 1-(*tert*-butoxycarbonyl)-4-methylpiperidine-4-carboxylic acid (121.7 mg, 0.5 mmol, 1.0 equiv.), 1-(2-chlorobenzyl)-2,4,6-triphenylpyridin-1-ium tetrafluoroborate (389.8 mg, 0.75 mmol, 1.5 equiv.), K_2CO_3 (172.8 mg, 1.25 mmol, 2.5 equiv.), 4CzIPN (19.7 mg, 25.0 μmol, 5.0 mol%), Fe(OEP)Cl (15.6 mg, 25.0 μmol, 5.0 mol%), DMA (5.0 mL), and *i*-PrOH (5.0 mL).

After work-up according to **General Procedure A**, the crude residue was purified by automated flash column chromatography (2 × 25 g silica column, 0-25% EtOAc/hexanes) followed by automated reverse-phase column chromatography (60 g C18 column, 70-85% MeCN/H_2O with 0.1% NH_4OH) to afford the title compound as a light-yellow oil (122.3 mg, 76% yield).

^{1}H NMR (500 MHz, $CDCl_3$) δ 7.37 – 7.30 (m, 1H), 7.18 – 7.08 (m, 3H), 3.74 (dt, J = 13.9, 4.5 Hz, 2H), 3.05 (ddd, J = 13.8, 10.8, 3.2 Hz, 2H), 2.74 (s, 2H), 1.56 – 1.47 (m, 2H), 1.43 (s, 9H), 1.37 – 1.29 (m, 2H), 0.95 (s, 3H).

^{13}C NMR (126 MHz, $CDCl_3$) δ 153.97, 135.03, 134.20, 131.75, 128.69, 126.53, 125.01, 78.23, 43.93, 38.85, 35.49, 33.07, 27.45, 21.25.

IR (film) ν_{max} 2971, 2928, 1688, 1420, 1246, 1156, 758, 679 cm^{-1}.

HRMS (ESI-TOF) *m/z* calculated for $C_{14}H_{19}ClNO_2^+$ ([M+H–isobutylene]$^+$) 268.1099, found 268.1099.

***tert*-butyl 4-(3-chloro-2-fluorobenzyl)-4-methylpiperidine-1-carboxylate (10):**

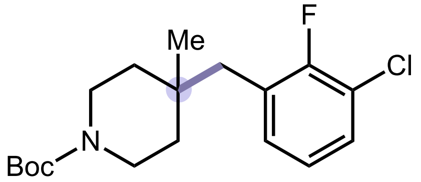

Prepared according to **General Procedure A** using 1-(*tert*-butoxycarbonyl)-4-methylpiperidine-4-carboxylic acid (121.7 mg, 0.5 mmol, 1.0 equiv.), 1-(3-chloro-2-fluorobenzyl)-2,4,6-triphenylpyridin-1-ium tetrafluoroborate (403.3 mg, 0.75 mmol, 1.5 equiv.), K_2CO_3 (172.8 mg, 1.25 mmol, 2.5 equiv.), 4CzIPN (19.7 mg, 25.0 μmol, 5.0 mol%), Fe(OEP)Cl (15.6 mg, 25.0 μmol, 5.0 mol%), DMA (5.0 mL), and *i*-PrOH (5.0 mL).

After work-up according to **General Procedure A**, the crude residue was purified by automated flash column chromatography (3 × 10 g silica column, 0-20% EtOAc/hexanes) followed by automated reverse-phase column chromatography (30 g C18 column, 65-85% MeCN/H_2O with 0.1% NH_4OH) to afford the title compound as a light-yellow oil (112.0 mg, 66% yield).

^{1}H NMR (500 MHz, CDCl$_3$) δ 7.25 – 7.18 (m, 1H), 7.03 – 6.95 (m, 2H), 3.71 (dd, *J* = 12.4, 5.9 Hz, 2H), 3.16 – 3.07 (m, 2H), 2.62 (s, 2H), 1.49 – 1.40 (m, 11H), 1.34 – 1.26 (m, 2H), 0.92 (s, 3H).

^{13}C NMR (126 MHz, CDCl$_3$) δ 156.93 (d, *J* = 247.0 Hz), 155.07, 131.40 (d, *J* = 4.3 Hz), 128.83, 127.00 (d, *J* = 16.3 Hz), 123.86 (d, *J* = 4.6 Hz), 121.24 (d, *J* = 19.3 Hz), 79.43, 41.44, 39.97, 36.51, 33.47, 28.59, 22.74 (d, *J* = 1.5 Hz).

^{19}F NMR (471 MHz, CDCl$_3$) δ -116.55.

IR (film) ν_{max} 2928, 1688, 1456, 1421, 1246, 1157, 783, 740, 655 cm^{-1}.

HRMS (ESI-TOF) *m/z* calculated for $C_{14}H_{18}ClFNO_2^+$ ([M+H–isobutylene]$^+$) 286.1005, found 286.1006.

benzyl 4-(3-((*tert*-butoxycarbonyl)amino)benzyl)-4-methylpiperidine-1-carboxylate (11):

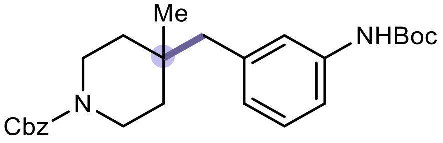

Prepared according to **General Procedure A** using 1-((benzyloxy)carbonyl)-4-methylpiperidine-4-carboxylic acid (71.4 mg, 0.25 mmol, 1.0 equiv.), 1-(3-((*tert*-butoxycarbonyl)amino)benzyl)-2,4,6-triphenylpyridin-1-ium tetrafluoroborate (225.1 mg, 0.375 mmol, 1.5 equiv.), K_2CO_3 (86.4 mg, 0.625 mmol, 2.5 equiv.), 4CzIPN (9.9 mg, 12.5 μmol, 5.0 mol%), Fe(OEP)Cl (7.8 mg, 12.5 μmol, 5.0 mol%), DMA (2.5 mL), and *i*-PrOH (2.5 mL).

After work-up according to **General Procedure A**, the crude residue was purified by automated flash column chromatography (25 g silica column, 0-20-40% EtOAc/hexanes) followed by automated reverse-phase column chromatography (30 g C18 column, 10-70-100% MeCN/H_2O with 0.1% NH_4OH) to afford the title compound as a colorless gum (87.2 mg, 80% yield).

^{1}H NMR (500 MHz, $CDCl_3$) δ 7.39 – 7.28 (m, 5H), 7.24 – 7.14 (m, 2H), 7.11 (s, 1H), 6.78 (d, *J* = 7.2 Hz, 1H), 6.41 (s, 1H), 5.12 (s, 2H), 3.92 – 3.65 (m, 2H), 3.20 (ddd, *J* = 13.7, 10.3, 3.4 Hz, 2H), 2.53 (s, 2H), 1.51 (s, 9H), 1.50 – 1.39 (m, 2H), 1.38 – 1.20 (m, 2H), 0.93 (s, 3H).

^{13}C NMR (126 MHz, $CDCl_3$) δ 155.46, 152.85, 138.86, 138.09, 137.05, 128.55, 128.41, 128.00, 127.92, 125.47, 120.82, 116.47, 80.45, 67.04, 48.74, 40.25, 36.59, 32.79, 28.47, 22.94.

IR (film) ν_{max} 3313, 2972, 2928, 2873, 1680, 1609, 1592, 1540, 1491, 1476, 1434, 1366, 1351, 1275, 1239, 1157, 1092, 1054, 1025, 908, 789, 765, 728, 696, 647, 602 cm^{-1}.

HRMS (ESI-TOF) *m/z* calculated for $C_{26}H_{34}N_2NaO_4^+$ ([M+Na]$^+$) 461.2411, found 461.2418.

benzyl 4-(4-(((*tert*-butoxycarbonyl)amino)methyl)benzyl)-4-methylpiperidine-1-carboxylate (12):

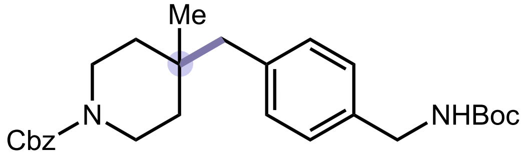

Prepared according to **General Procedure A** using 1-((benzyloxy)carbonyl)-4-methylpiperidine-4-carboxylic acid (138.7 mg, 0.5 mmol, 1.0 equiv.), 1-(4-(((*tert*-butoxycarbonyl)amino)methyl)benzyl)-2,4,6-triphenylpyridin-1-ium tetrafluoroborate (460.9 mg, 0.75 mmol, 1.5 equiv.), K_2CO_3 (172.8 mg, 1.25 mmol, 2.5 equiv.), 4CzIPN (19.7 mg, 25.0 µmol, 5.0 mol%), Fe(OEP)Cl (15.6 mg, 25.0 µmol, 5.0 mol%), DMA (5.0 mL), and *i*-PrOH (5.0 mL).

After work-up according to **General Procedure A**, the crude residue was purified by automated flash column chromatography (2 × 25 g silica column, 0-30% EtOAc/hexanes) followed by preparative HPLC (Waters Xbridge BEH C18 OBD Prep column, 60-70% MeCN/H_2O with 0.1% NH_4OH) to afford the title compound as a light-yellow oil (155.3 mg, 69% yield).

^{1}H NMR (500 MHz, $CDCl_3$) δ 7.39 – 7.27 (m, 5H), 7.18 (d, *J* = 7.7 Hz, 2H), 7.06 (d, *J* = 7.9 Hz, 2H), 5.12 (s, 2H), 4.84 (s, 1H), 4.29 (s, 1H), 3.78 (dt, *J* = 14.4, 4.7 Hz, 2H), 3.21 (ddd, *J* = 13.6, 10.1, 3.4 Hz, 2H), 2.54 (s, 2H), 1.58 – 1.40 (m, 11H), 1.33 – 1.24 (m, 2H), 0.91 (s, 3H).

^{13}C NMR (126 MHz, $CDCl_3$) δ 156.01, 155.48, 137.06, 137.05, 136.82, 130.97, 128.58, 128.03, 127.95, 127.11, 79.55, 67.07, 48.31, 44.50, 40.27, 36.56, 32.81, 28.54, 22.93.

IR (film) ν_{max} 3348, 2928, 1688, 1521, 1475, 1243, 1103, 1091, 1021, 911, 731, 692 cm^{-1}.

HRMS (ESI-TOF) *m/z* calculated for $C_{27}H_{36}N_2NaO_4^+$ ($[M+Na]^+$) 475.2567, found 475.2567.

***tert*-butyl 4-(3-(1*H*-pyrazol-1-yl)benzyl)-4-methylpiperidine-1-carboxylate (13):**

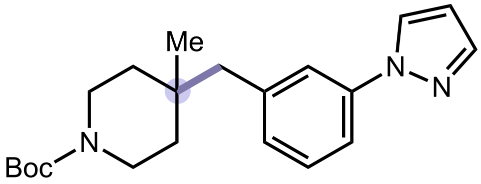

Prepared according to **General Procedure A** using 1-(*tert*-butoxycarbonyl)-4-methylpiperidine-4-carboxylic acid (121.7 mg, 0.5 mmol, 1.0 equiv.), 1-(3-(1*H*-pyrazol-1-yl)benzyl)-2,4,6-triphenylpyridin-1-ium tetrafluoroborate (413.5 mg, 0.75 mmol, 1.5 equiv.), K_2CO_3 (172.8 mg, 1.25 mmol, 2.5 equiv.), 4CzIPN (19.7 mg, 25.0 μmol, 5.0 mol%), Fe(OEP)Cl (15.6 mg, 25.0 μmol, 5.0 mol%), DMA (5.0 mL), and *i*-PrOH (5.0 mL).

After work-up according to **General Procedure A**, the crude residue was purified by automated flash column chromatography (3 × 10 g silica column, 5-30% EtOAc/hexanes) followed by automated reverse-phase column chromatography (30 g C18 column, 60-80% MeCN/H_2O with 0.1% NH_4OH) to afford the title compound as a light-yellow oil (131.7 mg, 74% yield).

^{1}H NMR (500 MHz, $CDCl_3$) δ 7.90 (d, J = 2.4 Hz, 1H), 7.71 (d, J = 1.7 Hz, 1H), 7.53 – 7.47 (m, 2H), 7.33 (t, J = 7.7 Hz, 1H), 7.03 (d, J = 7.6 Hz, 1H), 6.45 (t, J = 2.1 Hz, 1H), 3.75 – 3.68 (m, 2H), 3.11 (ddd, J = 13.7, 10.3, 3.4 Hz, 2H), 2.62 (s, 2H), 1.52 – 1.45 (m, 2H), 1.45 (s, 9H), 1.35 – 1.27 (m, 2H), 0.94 (s, 3H).

^{13}C NMR (126 MHz, $CDCl_3$) δ 155.07, 141.12, 139.93, 139.79, 128.90, 128.85, 126.90, 121.60, 117.03, 107.64, 79.39, 48.88, 39.96, 36.72, 32.91, 28.57, 22.82.

IR (film) ν_{max} 2972, 2927, 1686, 1592, 1423, 1248, 1158, 750 cm^{-1}.

HRMS (ESI-TOF) *m/z* calculated for $C_{17}H_{22}N_3O_2^+$ ([M+H–isobutylene]$^+$) 300.1707, found 300.1708.

***tert*-butyl 4-(benzo[*b*]thiophen-3-ylmethyl)-4-methylpiperidine-1-carboxylate (14):**

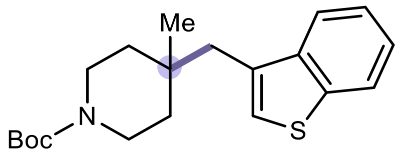

Prepared according to **General Procedure A** using 1-(*tert*-butoxycarbonyl)-4-methylpiperidine-4-carboxylic acid (62.7 mg, 0.25 mmol, 1.0 equiv.), 1-(benzo[*b*]thiophen-3-ylmethyl)-2,4,6-triphenylpyridin-1-ium tetrafluoroborate.[11] (203.0 mg, 0.375 mmol, 1.5 equiv.), K_2CO_3 (86.4 mg, 0.625 mmol, 2.5 equiv.), 4CzIPN (9.9 mg, 12.5 μmol, 5.0 mol%), Fe(OEP)Cl (7.8 mg, 12.5 μmol, 5.0 mol%), DMA (2.5 mL), and *i*-PrOH (2.5 mL).

After work-up according to **General Procedure A**, the crude residue was purified by automated flash column chromatography (25 g silica column, 0-10-30% EtOAc/hexanes) followed by automated reverse-phase column chromatography (30 g C18 column, 10-70-100% MeCN/H_2O with 0.1% NH_4OH) to afford the title compound as a light-yellow gum (56.5 mg, 65% yield).

^{1}H NMR (500 MHz, $CDCl_3$) δ 7.86 (d, *J* = 8.1 Hz, 1H), 7.77 (d, *J* = 7.8 Hz, 1H), 7.38 (td, *J* = 7.6, 1.3 Hz, 1H), 7.33 (td, *J* = 7.5, 1.4 Hz, 1H), 7.08 (s, 2H), 3.78 (dt, *J* = 13.8, 4.5 Hz, 2H), 3.05 (ddd, *J* = 13.9, 11.0, 3.3 Hz, 2H), 2.82 (s, 2H), 1.59 – 1.50 (m, 2H *overlapped with H_2O peak*), 1.45 (s, 9H), 1.36 (dt, *J* = 13.1, 3.3 Hz, 2H), 0.98 (s, 3H).

^{13}C NMR (126 MHz, $CDCl_3$) δ 155.09, 140.30, 140.15, 133.02, 124.03, 123.99, 123.87, 122.86, 122.60, 79.41, 41.05, 40.34, 39.80, 36.90, 33.49, 28.59, 22.91.

IR (film) ν_{max} 3070, 2971, 2916, 2869, 1685, 1476, 1456, 1422, 1364, 1353, 1276, 1246, 1156, 1093, 1020, 769, 760, 734 cm^{-1}.

HRMS (ESI-TOF) *m/z* calculated for $C_{20}H_{27}NNaO_2S^+$ ([M+Na]$^+$) 368.1655, found 368.1652.

[11] Bristol-Myers Squibb Company. Novel 2-Substituted Cyclic Amines as Calcium Sensing Receptor Modulators. WO Patent 2004/069793, 2004.

***tert*-butyl 4-methyl-4-(pyrazolo[1,5-*a*]pyridin-2-ylmethyl)piperidine-1-carboxylate (15):**

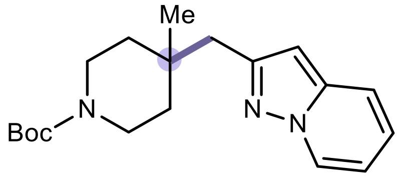

Prepared according to **General Procedure A** using 1-(*tert*-butoxycarbonyl)-4-methylpiperidine-4-carboxylic acid (62.7 mg, 0.25 mmol, 1.0 equiv.), 2,4,6-triphenyl-1-(pyrazolo[1,5-*a*]pyridin-2-ylmethyl)pyridin-1-ium tetrafluoroborate (197.0 mg, 0.375 mmol, 1.5 equiv.), K_2CO_3 (86.4 mg, 0.625 mmol, 2.5 equiv.), 4CzIPN (9.9 mg, 12.5 μmol, 5.0 mol%), Fe(OEP)Cl (7.8 mg, 12.5 μmol, 5.0 mol%), DMA (2.5 mL), and *i*-PrOH (2.5 mL).

After work-up according to **General Procedure A**, the crude residue was purified by automated flash column chromatography (25 g silica column, 0-15-25-50% EtOAc/hexanes) followed by automated reverse-phase column chromatography (30 g C18 column, 10-60-100% MeCN/H_2O with 0.1% NH_4OH) to afford the title compound as a light-yellow gum (43.2 mg, 52% yield).

^{1}H NMR (500 MHz, $CDCl_3$) δ 8.38 (dd, *J* = 7.0, 1.0 Hz, 1H), 7.42 (dt, *J* = 8.9, 1.2 Hz, 1H), 7.04 (ddd, *J* = 8.9, 6.7, 1.1 Hz, 1H), 6.66 (td, *J* = 6.8, 1.4 Hz, 1H), 6.26 (s, 2H), 3.75 – 3.57 (m, 2H), 3.21 (ddd, *J* = 13.4, 9.5, 3.6 Hz, 2H), 2.77 (s, 2H), 1.59 – 1.49 (m, 2H *overlapped with H_2O peak*), 1.45 (s, 9H), 1.41 – 1.31 (m, 2H), 1.01 (s, 3H).

^{13}C NMR (126 MHz, $CDCl_3$) δ 155.15, 152.42, 140.81, 128.25, 123.15, 117.44, 110.96, 97.56, 79.28, 40.73, 40.52, 39.71, 36.75, 32.51, 28.58, 23.85.

IR (film) ν_{max} 3083, 2971, 2923, 2869, 1687, 1635, 1520, 1422, 1392, 1364, 1329, 1276, 1247, 1158, 1094, 773, 748, 735 cm^{-1}.

HRMS (ESI-TOF) *m/z* calculated for $C_{19}H_{28}N_3O_2^+$ ([M+H]$^+$) 330.2176, found 330.2177.

***tert*-butyl 4-methyl-4-((1-methyl-1*H*-pyrazol-3-yl)methyl)piperidine-1-carboxylate (16):**

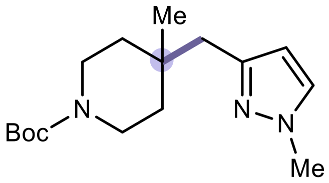

Prepared according to **General Procedure A** using 1-(*tert*-butoxycarbonyl)-4-methylpiperidine-4-carboxylic acid (62.7 mg, 0.25 mmol, 1.0 equiv.), 1-((1-methyl-1*H*-pyrazol-3-yl)methyl)-2,4,6-triphenylpyridin-1-ium tetrafluoroborate (183.5 mg, 0.375 mmol, 1.5 equiv.), K_2CO_3 (86.4 mg, 0.625 mmol, 2.5 equiv.), 4CzIPN (9.9 mg, 12.5 μmol, 5.0 mol%), Fe(OEP)Cl (7.8 mg, 12.5 μmol, 5.0 mol%), DMA (2.5 mL), and *i*-PrOH (2.5 mL).

After work-up according to **General Procedure A**, the crude residue was purified by automated flash column chromatography (25 g silica column, 0-20-45-70% EtOAc/hexanes) followed by automated reverse-phase column chromatography (30 g C18 column, 10-45% MeCN/H_2O with 0.1% NH_4OH) to afford the title compound as a colorless gum (53.1 mg, 72% yield).

^{1}H NMR (500 MHz, CDCl$_3$) δ 7.24 (d, *J* = 2.1 Hz, 1H), 5.98 (d, *J* = 2.1 Hz, 1H), 3.85 (s, 3H), 3.72 – 3.54 (m, 2H), 3.18 (ddd, *J* = 13.4, 9.6, 3.6 Hz, 2H), 2.57 (s, 2H), 1.52 – 1.39 (m, 2H), 1.45 (s, 9H), 1.37 – 1.24 (m, 2H), 0.96 (s, 3H).

^{13}C NMR (126 MHz, CDCl$_3$) δ 155.10, 149.30, 130.21, 106.37, 79.17, 40.38, 39.66, 38.70, 36.56, 32.12, 28.53, 23.64.

IR (film) ν_{max} 2972, 2927, 2870, 1685, 1520, 1420, 1406, 1364, 1354, 1275, 1262, 1246, 1157, 1092, 999, 753 cm^{-1}.

HRMS (ESI-TOF) *m/z* calculated for $C_{16}H_{28}N_3O_2^+$ ([M+H]$^+$) 294.2176, found 294.2174.

***tert*-butyl 4-methyl-4-((1-methyl-1*H*-pyrazol-5-yl)methyl)piperidine-1-carboxylate (17):**

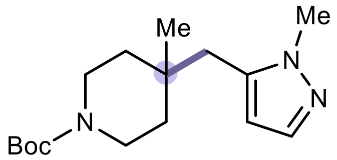

Prepared according to **General Procedure A** using 1-(*tert*-butoxycarbonyl)-4-methylpiperidine-4-carboxylic acid (62.7 mg, 0.25 mmol, 1.0 equiv.), 1-((1-methyl-1*H*-pyrazol-5-yl)methyl)-2,4,6-triphenylpyridin-1-ium tetrafluoroborate (183.5 mg, 0.375 mmol, 1.5 equiv.), K_2CO_3 (86.4 mg, 0.625 mmol, 2.5 equiv.), 4CzIPN (9.9 mg, 12.5 μmol, 5.0 mol%), Fe(OEP)Cl (7.8 mg, 12.5 μmol, 5.0 mol%), DMA (2.5 mL), and *i*-PrOH (2.5 mL).

After work-up according to **General Procedure A**, the crude residue was purified by automated flash column chromatography (25 g silica column, 0-20-45-70% EtOAc/hexanes) followed by automated reverse-phase column chromatography (30 g C18 column, 10-50-100% MeCN/H_2O with 0.1% NH_4OH) to afford the title compound as a light yellow gum (61.7 mg, 84% yield).

^{1}H NMR (500 MHz, $CDCl_3$) δ 7.41 (d, J = 1.9 Hz, 1H), 6.01 (d, J = 1.9 Hz, 1H), 3.82 (s, 3H), 3.79 – 3.65 (m, 2H), 3.08 (ddd, J = 14.0, 10.6, 3.3 Hz, 2H), 2.58 (s, 2H), 1.49 – 1.31 (m, 2H), 1.45 (s, 2H), 1.39 – 1.31 (m, 2H), 0.96 (s, 3H).

^{13}C NMR (126 MHz, $CDCl_3$) δ 154.94, 138.99, 138.04, 106.69, 79.47, 40.13, 39.57, 38.26, 36.85, 36.52, 33.41, 28.51, 22.51.

IR (film) ν_{max} 2972, 2929, 2871, 1686, 1477, 1420, 1399, 1364, 1355, 1276, 1262, 1246, 1157, 1095, 994, 767 cm^{-1}.

HRMS (ESI-TOF) *m/z* calculated for $C_{16}H_{28}N_3O_2^+$ ([M+H]$^+$) 294.2176, found 294.2172.

tert-butyl 4-((2-(4-fluorophenyl)thiazol-4-yl)methyl)-4-methylpiperidine-1-carboxylate (18):

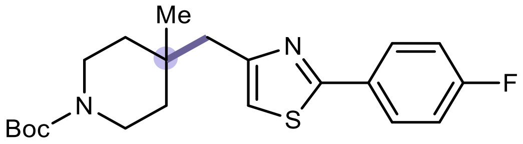

Prepared according to **General Procedure A** using 1-(*tert*-butoxycarbonyl)-4-methylpiperidine-4-carboxylic acid (62.7 mg, 0.25 mmol, 1.0 equiv.), 1-((2-(4-fluorophenyl)thiazol-4-yl)methyl)-2,4,6-triphenylpyridin-1-ium tetrafluoroborate (222.1 mg, 0.375 mmol, 1.5 equiv.), K_2CO_3 (86.4 mg, 0.625 mmol, 2.5 equiv.), 4CzIPN (9.9 mg, 12.5 μmol, 5.0 mol%), Fe(OEP)Cl (7.8 mg, 12.5 μmol, 5.0 mol%), DMA (2.5 mL), and *i*-PrOH (2.5 mL).

After work-up according to **General Procedure A**, the crude residue was purified by automated flash column chromatography (25 g silica column, 0-10-20% Et_2O/hexanes) followed by preparative HPLC (Waters Xbridge BEH C18 OBD Prep column, 10-70% MeCN/H_2O with 0.1% NH_4OH) to afford the title compound as a light-yellow gum (80.7 mg, 80% yield).

^{1}H NMR (500 MHz, $CDCl_3$) δ 7.95 – 7.87 (m, 2H), 7.15 – 7.08 (m, 2H), 6.86 (s, 1H), 3.74 – 3.55 (m, 2H), 3.27 (ddd, *J* = 13.3, 9.1, 3.7 Hz, 2H), 2.78 (s, 2H), 1.57 – 1.49 (m, 2H *overlapped with H_2O peak*), 1.46 (s, 9H), 1.41 – 1.32 (m, 2H), 1.01 (s, 3H).

^{13}C NMR (126 MHz, $CDCl_3$) δ 165.62, 163.80 (d, *J* = 249.9 Hz), 155.19, 155.15, 130.35 (d, *J* = 3.3 Hz), 128.41 (d, *J* = 8.4 Hz), 116.01 (d, *J* = 22.0 Hz), 115.35, 79.31, 42.76, 40.41, 39.73, 36.74, 32.72, 28.59, 24.08.

^{19}F NMR (376 MHz, $CDCl_3$) δ -111.03 (tt, *J* = 8.5, 5.3 Hz, 1F).

IR (film) ν_{max} 3092, 2972, 2925, 2869, 1686, 1518, 1504, 1476, 1454, 1422, 1392, 1365, 1296, 1276, 1261, 1247, 1233, 1156, 1097, 1002, 838, 766 cm^{-1}.

HRMS (ESI-TOF) *m/z* calculated for $C_{21}H_{28}FN_2O_2S^+$ ($[M+H]^+$) 391.1850, found 391.1857.

***tert*-butyl 4-((2-(2-chlorophenyl)oxazol-4-yl)methyl)-4-methylpiperidine-1-carboxylate (19):**

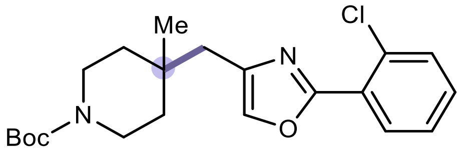

Prepared according to **General Procedure A** *on 0.1 mmol scale in 3 × 8-mL vials* using 1-(*tert*-butoxycarbonyl)-4-methylpiperidine-4-carboxylic acid (25.1 mg, 0.1 mmol, 1.0 equiv.), 1-((2-(2-chlorophenyl)oxazol-4-yl)methyl)-2,4,6-triphenylpyridin-1-ium tetrafluoroborate (88.9 mg, 0.15 mmol, 1.5 equiv.), K_2CO_3 (34.6 mg, 0.25 mmol, 2.5 equiv.), 4CzIPN (3.9 mg, 5.0 μmol, 5.0 mol%), Fe(OEP)Cl (3.1 mg, 5.0 μmol, 5.0 mol%), DMA (1.0 mL), and *i*-PrOH (1.0 mL). The vials were combined for workup.

After work-up according to **General Procedure A**, the crude residue was purified by automated flash column chromatography (25 g silica column, 0-20-45-100% Et_2O/hexanes) followed by preparative HPLC (Waters Xbridge BEH C18 OBD Prep column, 10-60-100% MeCN/H_2O with 0.1% NH_4OH) to afford the title compound as a light-yellow gum (60.8 mg, 52% yield).

^{1}H NMR (500 MHz, $CDCl_3$) δ 7.98 – 7.91 (m, 1H), 7.53 – 7.45 (m, 2H), 7.39 – 7.31 (m, 2H), 3.75 – 3.55 (m, 2H), 3.24 (ddd, *J* = 13.3, 9.3, 3.6 Hz, 2H), 2.58 (s, 2H), 1.56 – 1.48 (m, 2H *overlapped with H_2O peak*), 1.46 (s, 9H), 1.38 (dt, *J* = 13.3, 4.7 Hz, 2H), 1.03 (s, 3H).

^{13}C NMR (126 MHz, $CDCl_3$) δ 159.11, 155.13, 138.75, 136.27, 132.60, 131.19, 131.04, 131.00, 126.87, 126.75, 79.33, 40.42, 39.71, 37.92, 36.64, 32.25, 28.59, 23.81.

IR (film) ν_{max} 2972, 2918, 2870, 1685, 1474, 1457, 1422, 1391, 1364, 1352, 1276, 1261, 1247, 1157, 1089, 1033, 998, 823, 768, 738, 726, 699, 654 cm^{-1}.

HRMS (ESI-TOF) *m/z* calculated for $C_{21}H_{27}ClN_2NaO_3^+$ ([M+Na]$^+$) 413.1602, found 413.1609.

tert-butyl 4-((1-(2-fluorobenzyl)-1_H_-1,2,3-triazol-4-yl)methyl)-4-methylpiperidine-1-carboxylate (20):

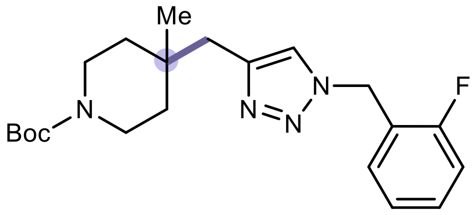

Prepared according to **General Procedure A** using 1-(*tert*-butoxycarbonyl)-4-methylpiperidine-4-carboxylic acid (62.7 mg, 0.25 mmol, 1.0 equiv.), 1-((1-(2-fluorobenzyl)-1*H*-1,2,3-triazol-4-yl)methyl)-2,4,6-triphenylpyridin-1-ium tetrafluoroborate (219.1 mg, 0.375 mmol, 1.5 equiv.), K_2CO_3 (86.4 mg, 0.625 mmol, 2.5 equiv.), 4CzIPN (9.9 mg, 12.5 µmol, 5.0 mol%), Fe(OEP)Cl (7.8 mg, 12.5 µmol, 5.0 mol%), DMA (2.5 mL), and *i*-PrOH (2.5 mL).

After work-up according to **General Procedure A**, the crude residue was purified by automated flash column chromatography (25 g silica column, 0-20-45% EtOAc/hexanes) followed by automated reverse-phase column chromatography (30 g C18 column, 10-45% MeCN/H_2O with 0.1% NH_4OH) to afford the title compound as a light-yellow gum (82.6 mg, 85% yield).

^{1}H NMR (500 MHz, $CDCl_3$) δ 7.35 (tdd, *J* = 7.5, 5.4, 1.8 Hz, 1H), 7.27 (s, 1H), 7.23 (td, *J* = 7.6, 1.8 Hz, 1H), 7.19 – 7.08 (m, 2H), 5.57 (s, 2H), 3.74 – 3.54 (m, 2H), 3.18 (ddd, *J* = 13.4, 9.5, 3.6 Hz, 2H), 2.65 (s, 2H), 1.48 – 1.38 (m, 2H), 1.44 (s, 9H), 1.33 – 1.24 (m, 2H), 0.93 (s, 3H).

^{13}C NMR (126 MHz, $CDCl_3$) δ 160.58 (d, *J* = 247.7 Hz), 155.08, 144.76, 130.86 (d, *J* = 8.1 Hz), 130.50 (d, *J* = 3.3 Hz), 124.92 (d, *J* = 3.6 Hz), 122.45 (d, *J* = 1.7 Hz), 122.30 (d, *J* = 14.6 Hz), 115.88 (d, *J* = 21.1 Hz), 79.34, 47.59 (d, *J* = 4.5 Hz), 40.35, 39.61, 37.70, 36.45, 32.14, 28.55, 23.53.

^{19}F NMR (376 MHz, $CDCl_3$) δ -116.35 – -120.77 (m, 1F).

IR (film) ν_{max} 3128, 2972, 2927, 2870, 1682, 1493, 1457, 1422, 1392, 1364, 1276, 1262, 1247, 1235, 1157, 1095, 1047, 995, 757, 731 cm^{-1}.

HRMS (ESI-TOF) *m/z* calculated for $C_{21}H_{30}FN_4O_2^+$ ($[M+H]^+$) 389.2347, found 389.2351.

***tert*-butyl 4-methyl-4-((2-trityl-2*H*-tetrazol-5-yl)methyl)piperidine-1-carboxylate (21):**

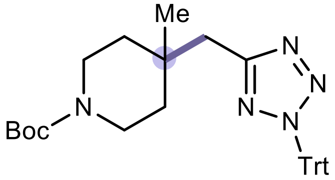

Prepared according to **General Procedure A** using 1-(*tert*-butoxycarbonyl)-4-methylpiperidine-4-carboxylic acid (25.0 mg, 0.1 mmol, 1.0 equiv.), 2,4,6-triphenyl-1-((2-trityl-2*H*-tetrazol-5-yl)methyl)pyridin-1-ium tetrafluoroborate (107.9 mg, 0.15 mmol, 1.5 equiv.), K_2CO_3 (34.6 mg, 0.25 mmol, 2.5 equiv.), 4CzIPN (3.9 mg, 5.0 μmol, 5.0 mol%), Fe(OEP)Cl (3.1 mg, 5.0 μmol, 5.0 mol%), DMA (1.0 mL), and *i*-PrOH (1.0 mL).

After work-up according to **General Procedure A**, the crude residue was purified by automated flash column chromatography (2 × 10 g silica column, 0-20-30-50% Et_2O/hexanes) followed by automated reverse-phase column chromatography (30 g C18 column, 10-80% MeCN/H_2O with 0.1% NH_4OH) and preparative HPLC (Waters Xbridge BEH C18 OBD Prep column, 10-70% MeCN/H_2O with 0.1% NH_4OH) to afford the title compound as a light-yellow solid (35.3 mg, 67% yield).

^{1}H NMR (500 MHz, Acetone-d$_6$) δ 7.53 – 7.28 (m, 9H) 7.18 – 7.04 (m, 6H), 3.52 (ddd, *J* = 13.7, 6.9, 4.1 Hz, 2H), 3.34 – 3.18 (m, 2H), 2.90 (s, 2H), 1.46 – 1.38 (m, 2H), 1.42 (s, 9H), 1.32 – 1.25 (m, 2H), 0.95 (s, 3H).

^{13}C NMR (126 MHz, Acetone-d$_6$) δ 164.12, 155.31, 142.77, 131.10, 129.32, 128.84, 83.51, 79.39, 41.13, 40.20, 37.26, 36.86, 33.35, 30.01, 28.79, 24.77.

IR (film) ν_{max} 3060, 2970, 2927, 2871, 1689, 1492, 1446, 1422, 1391, 1365, 1276, 1262, 1245, 1157, 1088, 1017, 1001, 883, 749, 698, 639 cm^{-1}.

HRMS (ESI-TOF) *m/z* calculated for $C_{32}H_{37}N_5NaO_2^+$ ([M+Na]$^+$) 546.2839, found 546.2841.

methyl 6-((1-(*tert*-butoxycarbonyl)-4-methylpiperidin-4-yl)methyl)picolinate (22):

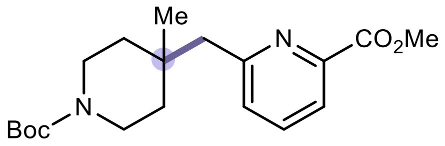

Prepared according to **General Procedure A** using 1-(*tert*-butoxycarbonyl)-4-methylpiperidine-4-carboxylic acid (62.7 mg, 0.25 mmol, 1.0 equiv.), 1-((6-(methoxycarbonyl)pyridin-2-yl)methyl)-2,4,6-triphenylpyridin-1-ium tetrafluoroborate (206.2 mg, 0.375 mmol, 1.5 equiv.), K_2CO_3 (86.4 mg, 0.625 mmol, 2.5 equiv.), 4CzIPN (9.9 mg, 12.5 μmol, 5.0 mol%), Fe(OEP)Cl (7.8 mg, 12.5 μmol, 5.0 mol%), DMA (2.5 mL), and *i*-PrOH (2.5 mL).

After work-up according to **General Procedure A**, the crude residue was purified by automated flash column chromatography (25 g silica column, 0-20-45-100% EtOAc/hexanes) followed by automated reverse-phase column chromatography (30 g C18 column, 10-50% MeCN/H_2O with 0.1% NH_4OH) to afford the title compound as a light-yellow gum (61.7 mg, 71% yield).

^{1}H NMR (500 MHz, $CDCl_3$) δ 7.97 (dd, *J* = 7.8, 1.1 Hz, 1H), 7.73 (t, *J* = 7.7 Hz, 1H), 7.28 (dd, *J* = 7.8, 1.1 Hz, 1H), 3.99 (s, 3H), 3.79 – 3.58 (m, 2H), 3.19 (ddd, *J* = 13.4, 9.7, 3.5 Hz, 2H), 2.88 (s, 2H), 1.51 (ddd, *J* = 13.8, 9.7, 4.2 Hz, 2H), 1.45 (s, 9H), 1.33 (dd, *J* = 12.3, 5.9 Hz, 2H), 0.97 (s, 3H).

^{13}C NMR (126 MHz, $CDCl_3$) δ 166.16, 159.51, 155.07, 147.57, 136.55, 128.19, 122.83, 79.32, 52.92, 49.84, 40.31, 39.50, 36.68, 33.26, 28.54, 23.31.

IR (film) ν_{max} 2951, 2926, 2870, 1744, 1723, 1685, 1587, 1455, 1421, 1364, 1316, 1289, 1276, 1261, 1246, 1233, 1157, 1102, 992, 973, 768, 750, 731 cm^{-1}.

HRMS (ESI-TOF) *m/z* calculated for $C_{19}H_{28}N_2NaO_4^+$ ($[M+Na]^+$) 371.1941, found 371.1948.

benzyl 4-(cyclohex-1-en-1-ylmethyl)-4-methylpiperidine-1-carboxylate (23):

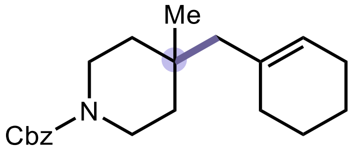

Prepared according to **General Procedure A** using 1-((benzyloxy)carbonyl)-4-methylpiperidine-4-carboxylic acid (71.4 mg, 0.25 mmol, 1.0 equiv.), 1-(cyclohex-1-en-1-ylmethyl)-2,4,6-triphenylpyridin-1-ium tetrafluoroborate (185.4 mg, 0.375 mmol, 1.5 equiv.), K_2CO_3 (86.4 mg, 0.625 mmol, 2.5 equiv.), 4CzIPN (9.9 mg, 12.5 µmol, 5.0 mol%), Fe(OEP)Cl (7.8 mg, 12.5 µmol, 5.0 mol%), DMA (2.5 mL), and *i*-PrOH (2.5 mL).

After work-up according to **General Procedure A**, the crude residue was purified by automated flash column chromatography (25 g silica column, 0-20-50-100% DCM/hexanes) followed by preparative HPLC (Waters Xbridge BEH C18 OBD Prep column, 10-70% MeCN/H_2O with 0.1% NH_4OH) to afford the title compound as a light yellow oil (30.5 mg, 37% yield).

^{1}H NMR (500 MHz, $CDCl_3$) δ 7.46 – 7.27 (m, 5H), 5.39 – 5.33 (m, 1H), 5.12 (s, 2H), 3.78 – 3.60 (m, 2H), 3.24 (ddd, *J* = 13.5, 9.7, 3.6 Hz, 2H), 2.07 – 1.93 (m, 4H), 1.90 (s, 2H), 1.63 – 1.49 (m, 4H), 1.48 – 1.37 (m, 2H), 1.35 – 1.22 (m, 2H), 0.93 (s, 3H).

^{13}C NMR (126 MHz, $CDCl_3$) δ 155.53, 137.17, 134.66, 128.57, 128.00, 127.93, 125.82, 67.02, 50.49, 40.39, 37.29, 37.13, 32.68, 31.94, 25.57, 23.91, 23.37, 22.42.

IR (film) ν_{max} 2921, 2836, 1697, 1427, 1350, 1276, 1238, 1207, 1168, 1090, 1023, 995, 763, 751, 732, 696 cm^{-1}.

HRMS (ESI-TOF) *m/z* calculated for $C_{21}H_{30}NO_2^+$ ([M+H]$^+$) 328.2271, found 328.2277.

benzyl 4-benzyl-4-methylpiperidine-1-carboxylate (24):

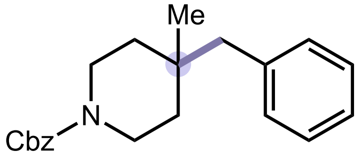

Prepared according to **General Procedure A** using 1-((benzyloxy)carbonyl)-4-methylpiperidine-4-carboxylic acid (138.7 mg, 0.5 mmol, 1.0 equiv.), 1-benzyl-2,4,6-triphenylpyridin-1-iumtetrafluoroborate (364.0 mg, 0.75 mmol, 1.5 equiv.), K_2CO_3 (103.7 mg, 0.75 mmol, 1.5 equiv.), 4CzIPN (19.7 mg, 25.0 μmol, 5.0 mol%), Fe(OEP)Cl (15.6 mg, 25.0 μmol, 5.0 mol%), DMA (5.0 mL), and *i*-PrOH (5.0 mL).

After work-up according to **General Procedure A**, the crude residue was purified by automated reverse-phase column chromatography (60 g C18 column, 60-80% MeCN/H_2O with 0.1% formic acid) to afford the title compound as a tan solid (120.9 mg, 75% yield).

^{1}H NMR (500 MHz, CDCl$_3$) δ 7.38 – 7.19 (m, 8H), 7.14 – 7.08 (m, 2H), 5.14 (s, 2H), 3.85 – 3.76 (m, 2H), 3.23 (ddd, *J* = 13.6, 10.1, 3.5 Hz, 2H), 2.58 (s, 2H), 1.53 – 1.42 (m, 2H), 1.37 – 1.25 (m, 2H), 0.93 (s, 3H).

^{13}C NMR (126 MHz, CDCl$_3$) δ 155.48, 137.95, 137.07, 130.75, 128.56, 128.01, 127.96, 127.93, 127.91, 126.21, 67.05, 48.68, 40.28, 36.59, 32.79, 22.93.

IR (film) ν_{max} 3027, 2920, 1695, 1427, 1241, 1166, 1090, 1024, 994, 760, 697, 608 cm^{-1}.

HRMS (ESI-TOF) *m/z* calculated for $C_{21}H_{26}NO_2^+$ ([M+H]$^+$) 324.1958, found 324.1957.

tert-butyl 4-((6-chloropyridin-3-yl)methyl)-4-phenylpiperidine-1-carboxylate (25):

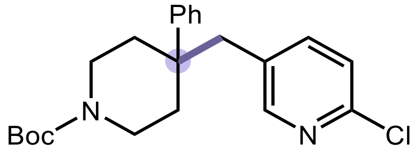

*Prepared according to **General Procedure A** with the following modifications: **NMP** instead of **DMA** was used as the optimal solvent.*

Prepared according to **General Procedure A** using 1-(*tert*-butoxycarbonyl)-4-phenylpiperidine-4-carboxylic acid (80.3 mg, 0.25 mmol, 1.0 equiv.), 1-((6-chloropyridin-3-yl)methyl)-2,4,6-triphenylpyridin-1-ium tetrafluoroborate (195.3 mg, 0.375 mmol, 1.5 equiv.), K_2CO_3 (86.4 mg, 0.625 mmol, 2.5 equiv.), 4CzIPN (9.9 mg, 12.5 μmol, 5.0 mol%), Fe(OEP)Cl (7.8 mg, 12.5 μmol, 5.0 mol%), NMP (2.5 mL), and *i*-PrOH (2.5 mL).

After work-up according to **General Procedure A**, the crude residue was purified by automated flash column chromatography (25 g silica column, 0-15-30% EtOAc/hexanes) followed by automated reverse-phase column chromatography (30 g C18 column, 10-65% MeCN/H_2O with 0.1% NH_4OH) to afford the title compound as a white amorphous solid (58.2 mg, 60% yield).

^{1}H NMR (500 MHz, $CDCl_3$) δ 7.65 (d, *J* = 2.4 Hz, 1H), 7.34 – 7.27 (m, 2H), 7.26 – 7.19 (m, 1H), 7.08 – 7.02 (m, 2H), 6.99 (d, *J* = 8.1 Hz, 1H), 6.62 (dd, *J* = 8.2, 2.5 Hz, 1H), 4.05 – 3.60 (m, 2H), 2.95 (t, *J* = 12.1 Hz, 2H), 2.77 (s, 2H), 2.28 – 2.07 (m, 2H), 1.81 – 1.64 (m, 2H), 1.44 (s, 10H).

^{13}C NMR (126 MHz, $CDCl_3$) δ 155.03, 150.87, 149.55, 141.91, 140.43, 131.52, 128.81, 127.32, 126.82, 123.09, 79.58, 47.33, 41.53, 40.73, 39.86, 34.98, 28.55.

IR (film) ν_{max} 2975, 2934, 2866, 1688, 1459, 1423, 1388, 1365, 1246, 1170, 1118, 1105, 743, 702 cm^{-1}.

HRMS (ESI-TOF) *m/z* calculated for $C_{22}H_{28}ClN_2O_2^+$ ($[M+H]^+$) 387.1834, found 387.1832.

benzyl 2-((6-chloropyridin-3-yl)methyl)piperidine-1-carboxylate ((±)-26):

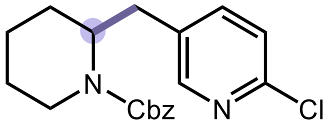

*Prepared according to **General Procedure A** with the following modifications: **NMP** instead of **DMA** was used as the optimal solvent.*

Prepared according to **General Procedure A** using (*S*)-1-(carbobenzyloxy)-2-piperidinecarboxylic acid (66.8 mg, 0.25 mmol, 1.0 equiv.), 1-((6-chloropyridin-3-yl)methyl)-2,4,6-triphenylpyridin-1-ium tetrafluoroborate (195.3 mg, 0.375 mmol, 1.5 equiv.), K_2CO_3 (86.4 mg, 0.625 mmol, 2.5 equiv.), 4CzIPN (9.9 mg, 12.5 μmol, 5.0 mol%), Fe(OEP)Cl (7.8 mg, 12.5 μmol, 5.0 mol%), NMP (2.5 mL), and *i*-PrOH (2.5 mL).

After work-up according to **General Procedure A**, the crude residue was purified by automated flash column chromatography (25 g silica column, 0-20-45% EtOAc/hexanes) followed by automated reverse-phase column chromatography (30 g C18 column, 10-55-65% MeCN/H_2O with 0.1% NH_4OH) to afford the title compound as a colorless oil (47.4 mg, 55% yield).

^{1}H NMR (500 MHz, $CDCl_3$) δ 8.17 (s, 1H), 7.69 – 7.29 (m, 4H), 7.26 – 7.21 (m, 2H *overlapped with solvent peak*), 7.19 – 7.08 (m, 1H), 5.00 (s, 2H), 4.70 – 4.30 (m, 1H), 4.25 – 3.90 (m, 1H), 3.09 – 2.86 (m, 2H), 2.84 – 2.68 (m, 1H), 1.78 – 1.55 (m, 5H), 1.51 – 1.40 (s, 1H).

^{13}C NMR (126 MHz, $CDCl_3$) δ 155.44, 150.08, 149.61, 139.48, 136.69, 133.36, 128.61, 128.16, 128.00, 124.05, 67.20, 52.02, 39.62, 32.64, 27.39, 25.42, 18.90.

IR (film) ν_{max} 2940, 2863, 1693, 1459, 1421, 1385, 1346, 1264, 1167, 1137, 1090, 1040, 739, 698 cm^{-1}.

HRMS (ESI-TOF) *m/z* calculated for $C_{19}H_{22}ClN_2O_2^+$ ($[M+H]^+$) 345.1364, found 345.1364.

***tert*-butyl 3-((6-chloropyridin-3-yl)methyl)morpholine-4-carboxylate ((±)-27):**

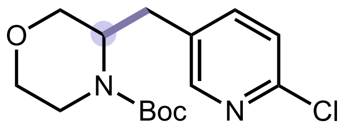

Prepared according to **General Procedure A** using 4-(*tert*-butoxycarbonyl)morpholine-3-carboxylic acid (121.7 mg, 0.5 mmol, 1.0 equiv.), 1-((6-chloropyridin-3-yl)methyl)-2,4,6-triphenylpyridin-1-ium tetrafluoroborate (390.6 mg, 0.75 mmol, 1.5 equiv.), K_2CO_3 (103.7 mg, 0.75 mmol, 1.5 equiv.), 4CzIPN (19.7 mg, 25.0 μmol, 5.0 mol%), Fe(OEP)Cl (15.6 mg, 25.0 μmol, 5.0 mol%), DMA (5.0 mL), and *i*-PrOH (5.0 mL).

After work-up according to **General Procedure A**, the crude residue was purified by automated flash column chromatography (50 g silica column, 0-60% EtOAc/hexanes) followed by automated flash column chromatography (25 g silica column, 0-10% acetone/DCM) to afford the title compound as a brown oil (111.2 mg, 71% yield).

^{1}H NMR (500 MHz, $CDCl_3$) δ 8.23 (d, *J* = 2.5 Hz, 1H), 7.49 (s, 1H), 7.24 (d, *J* = 8.2 Hz, 1H), 4.27 – 3.59 (m, 4H), 3.54 – 3.38 (m, 2H), 3.24 – 3.15 (m, 1H), 2.96 (qd, *J* = 13.7, 7.8 Hz, 2H), 1.34 (s, 9H).

^{13}C NMR (126 MHz, $CDCl_3$) δ 154.41, 150.43, 149.78, 139.89, 133.02, 124.11, 80.41, 68.04, 67.00, 53.20, 51.27, 40.29, 38.77, 31.64, 28.31.

IR (film) ν_{max} 2973, 2860, 1687, 1588, 1565, 1457, 1405, 1385, 1165, 1100, 999, 865, 765 cm^{-1}.

HRMS (ESI-TOF) *m/z* calculated for $C_{11}H_{14}ClN_2O_3^+$ ([M+H–isobutylene]$^+$) 257.0687, found 257.0686.

4-benzyl 1-(tert-butyl) 2-((6-chloropyridin-3-yl)methyl)piperazine-1,4-dicarboxylate ((±)-28):

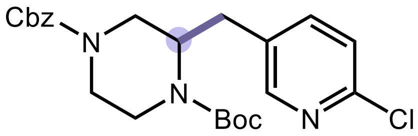

*Prepared according to **General Procedure A** with the following modifications: **NMP** instead of **DMA** was used as the optimal solvent.*

Prepared according to **General Procedure A** using 4-((benzyloxy)carbonyl)-1-(*tert*-butoxycarbonyl)piperazine-2-carboxylic acid (91.1 mg, 0.25 mmol, 1.0 equiv.), 1-((6-chloropyridin-3-yl)methyl)-2,4,6-triphenylpyridin-1-ium tetrafluoroborate (195.3 mg, 0.375 mmol, 1.5 equiv.), K_2CO_3 (51.8 mg, 0.375 mmol, 1.5 equiv.), 4CzIPN (9.9 mg, 12.5 μmol, 5.0 mol%), Fe(OEP)Cl (7.8 mg, 12.5 μmol, 5.0 mol%), DMA (2.5 mL), and *i*-PrOH (2.5 mL).

After work-up according to **General Procedure A**, the crude residue was purified by automated flash column chromatography (25 g silica column, 0-20-30% EtOAc/hexanes) followed by automated reverse-phase column chromatography (30 g C18 column, 10-66% MeCN/H_2O with 0.1% NH_4OH) to afford the title compound as an off-white solid (71.3 mg, 64% yield).

^{1}H NMR (500 MHz, CDCl$_3$) δ 8.33 – 8.08 (m, 1H), 7.70 – 7.31 (m, 6H), 7.14 – 6.94 (m, 1H), 5. 28 – 5.04 (m, 2H), 4.57 – 3.61 (m, 4H), 3.18 – 2.81 (m, 3H), 2.76 (d, J = 7.5 Hz, 2H), 1.37 (s, 9H). **(summary of rotamers)**

^{13}C NMR (126 MHz, CDCl$_3$) δ 155.64, 154.37, 150.42, 149.84, 139.97, 136.42, 136.26, 132.51, 128.74, 128.46, 128.21, 124.02, 80.70, 67.74, 53.20, 51.28, 45.19, 43.79, 43.54, 39.56, 38.45, 32.13, 28.33. **(summary of rotamers)**

IR (film) ν_{max} 2976, 2931, 2867, 1687, 1458, 1430, 1408, 1391, 1365, 1350, 1282, 1248, 1222, 1164, 1128, 1101, 1059, 992, 911, 763, 729, 697 cm^{-1}.

HRMS (ESI-TOF) *m/z* calculated for $C_{23}H_{29}ClN_3O_4^+$ ($[M+Na]^+$) 446.1841, found 446.1843.

(9*H*-fluoren-9-yl)methyl 2-((6-chloropyridin-3-yl)methyl)pyrrolidine-1-carboxylate ((±)-29):

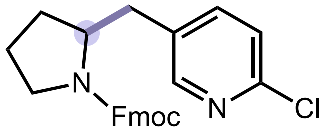

Prepared according to **General Procedure A** using Fmoc-Pro-OH (173.9 mg, 0.5 mmol, 1.0 equiv.), 1-((6-chloropyridin-3-yl)methyl)-2,4,6-triphenylpyridin-1-ium tetrafluoroborate (390.6 mg, 0.75 mmol, 1.5 equiv.), K_2CO_3 (172.8 mg, 1.25 mmol, 2.5 equiv.), 4CzIPN (19.7 mg, 25.0 μmol, 5.0 mol%), Fe(OEP)Cl (15.6 mg, 25.0 μmol, 5.0 mol%), DMA (5.0 mL), and *i*-PrOH (5.0 mL).

After work-up according to **General Procedure A**, the crude residue was purified by automated flash column chromatography (3 × 10 g silica column, 0-40% EtOAc/hexanes) followed by automated reverse-phase column chromatography (30 g C18 column, 55-80% MeCN/H_2O with 0.1% formic acid) to afford the title compound as a light-yellow oil (112.6 mg, 54% yield).

^{1}H NMR (500 MHz, CDCl$_3$) δ 8.21 (s, 1H), 7.75 (dd, *J* = 28.8, 7.6 Hz, 3H), 7.66 – 7.58 (m, 5H), 7.52 – 7.47 (m, 1H), 7.13 (d, *J* = 8.1 Hz, 1H), 4.86 (dd, *J* = 11.0, 4.3 Hz, 1H), 4.50 – 4.37 (m, 2H), 4.23 – 4.18 (m, 1H), 3.49 – 3.37 (m, 2H), 3.14 – 3.07 (m, 1H), 2.68 (dd, *J* = 13.5, 8.9 Hz, 1H), 1.91 – 1.69 (m, 5H). **(1:1 rotamer)**

^{1}H NMR (500 MHz, CDCl$_3$) δ 7.84 (s, 1H), 7.52 – 7.47 (m, 1H), 7.44 – 7.38 (m, 3H), 7.38 – 7.27 (m, 5H), 7.24 (d, *J* = 8.3 Hz, 1H), 6.78 (d, *J* = 2.4 Hz, 1H), 4.77 (dd, *J* = 11.0, 4.2 Hz, 1H), 4.26 (t, *J* = 7.0 Hz, 1H), 4.09 – 4.01 (m, 1H), 3.37 – 3.27 (m, 2H), 3.26 – 3.18 (m, 1H), 2.28 (dd, *J* = 13.5, 3.6 Hz, 1H), 2.06 (dd, *J* = 13.5, 9.4 Hz, 1H), 1.71 – 1.54 (m, 4H), 1.46 – 1.37 (m, 1H). **(1:1 rotamer)**

^{13}C NMR (126 MHz, CDCl$_3$) δ 162.36, 155.05, 154.87, 150.30, 150.01, 149.68, 149.49, 144.13, 144.10, 141.58, 141.54, 141.47, 139.96, 139.71, 133.29, 132.99, 127.81, 127.77, 127.31, 127.25,

127.14, 125.17, 125.12, 124.51, 124.16, 123.91, 120.10, 120.07, 67.11, 65.50, 58.74, 57.62, 47.80, 47.49, 46.95, 46.77, 36.01, 35.93, 29.58, 29.13, 23.71, 22.67. **(summary of romaters)**

IR (film) ν_{max} 2952, 2877, 1691, 1585, 1564, 1450, 1412, 1353, 1333, 1187, 1108, 1024, 909, 818, 737, 621 cm^{-1}.

HRMS (ESI-TOF) *m/z* calculated for $C_{25}H_{24}ClN_2O_2^+$ ($[M+H]^+$) 419.1521, found 419.1520.

5-((1-(4-bromophenyl)cyclopropyl)methyl)-2-chloropyridine (30):

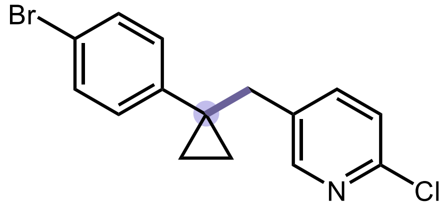

*Prepared according to **General Procedure A** with the following modifications: **Acetone / t-Amyl alcohol** instead of **DMA / i-PrOH** was used as the optimal solvent mixture. **Water** (4.5 μL, 0.25 mmol, 5.0 equiv.) was added via syringe after sparging with N_2.*

Prepared according to **General Procedure A** *on 0.05 mmol scale in 4 × 8-mL vials* using 1-(4-bromophenyl)cyclopropane-1-carboxylic acid (12.4 mg, 0.05 mmol, 1.0 equiv.), 1-((6-chloropyridin-3-yl)methyl)-2,4,6-triphenylpyridin-1-ium tetrafluoroborate (40.3 mg, 75 μmol, 1.5 equiv.), K_2CO_3 (10.4 mg, 752μmol, 1.5 equiv.), 4CzIPN (2.0 mg, 2.5 μmol, 5.0 mol%), Fe(OEP)Cl (1.6 mg, 2.5 μmol, 5.0 mol%), acetone (0.5 mL), t-Amyl-OH (0.5 mL), Water (4.5 μL, 0.25 mmol, 5.0 equiv.). The vials were combined for workup.

After work-up according to **General Procedure A**, the crude residue was purified by automated flash column chromatography (25 g silica column, 0-15-30% EtOAc/hexanes) followed by automated reverse-phase column chromatography (30 g C18 column, 10-65% MeCN/H_2O with 0.1% NH_4OH) to afford the title compound as a white amorphous solid (58.2 mg, 60% yield).

^{1}H NMR (500 MHz, CDCl$_3$) δ 8.00 (d, J = 2.3 Hz, 1H), 7.36 – 7.30 (m, 2H), 7.16 (dd, J = 8.2, 2.4 Hz, 1H), 7.13 (d, J = 8.3 Hz, 1H), 7.01 – 6.94 (m, 2H), 2.83 (s, 2H), 0.89 (s, 4H).

^{13}C NMR (126 MHz, CDCl$_3$) δ 150.14, 149.53, 142.67, 139.54, 133.94, 131.49, 131.12, 123.68, 120.42, 42.49, 26.34, 12.96.

IR (film) ν_{max} 3077, 3000, 2915, 2850, 1584, 1564, 1489, 1456, 1424, 1383, 1136, 1100, 1070, 1056, 1024, 1009, 825, 814, 745, 723, 714, 676, 634 cm^{-1}.

HRMS (ESI-TOF) *m/z* calculated for $C_{15}H_{14}BrClN^+$ ($[M+H]^+$) 321.9993, found 321.9993.

***tert*-butyl (1-(6-chloropyridin-3-yl)-3-hydroxy-3-methylbutan-2-yl)carbamate ((±)-31):**

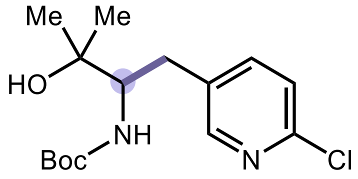

*Prepared according to **General Procedure A** with the following modifications: **NMP** instead of **DMA** was used as the optimal solvent.*

Prepared according to **General Procedure A** using (*S*)-2-((*tert*-butoxycarbonyl)amino)-3-hydroxy-3-methylbutanoic acid (60.1 mg, 0.25 mmol, 1.0 equiv.), 1-((6-chloropyridin-3-yl)methyl)-2,4,6-triphenylpyridin-1-ium tetrafluoroborate (195.3 mg, 0.375 mmol, 1.5 equiv.), K_2CO_3 (86.4 mg, 0.625 mmol, 2.5 equiv.), 4CzIPN (9.9 mg, 12.5 μmol, 5.0 mol%), Fe(OEP)Cl (7.8 mg, 12.5 μmol, 5.0 mol%), NMP (2.5 mL), and *i*-PrOH (2.5 mL).

After work-up according to **General Procedure A**, the crude residue was purified by automated flash column chromatography (25 g silica column, 0-20% EtOAc/hexanes) followed by automated reverse-phase column chromatography (30 g C18 column, 10-45% MeCN/H_2O with 0.1% NH_4OH) to afford the title compound as an off-white solid (43.5 mg, 55% yield).

^{1}H NMR (500 MHz, CDCl$_3$) δ 8.33 – 8.13 (m, 1H), 7.62 – 7.46 (m, 1H), 7.24 (d, *J* = 8.1 Hz, 1H), 4.74 – 4.43 (m, 1H), 3.76 – 3.45 (m, 1H), 3.04 (dd, *J* = 14.3, 3.6 Hz, 1H), 2.71 – 2.39 (m, 1H), 1.99 (s, 1H), 1.37 – 1.18 (m, 15H).

^{13}C NMR (126 MHz, CDCl$_3$) δ 156.14, 150.39, 149.51, 139.63, 133.67, 123.92, 79.79, 72.85, 59.60, 32.61, 28.28, 27.55, 27.28.

IR (film) ν_{max} 3347, 2976, 2933, 1691, 1566, 1515, 1460, 1386, 1366, 1329, 1248, 1168, 1110, 1070, 1025 cm^{-1}.

HRMS (ESI-TOF) *m/z* calculated for $C_{15}H_{24}ClN_2O_3^+$ ([M+H]$^+$) 315.1470, found 315.1475.

***tert*-butyl 4-((6-chloropyridin-3-yl)methyl)-2,2-dimethyloxazolidine-3-carboxylate ((±)-32):**

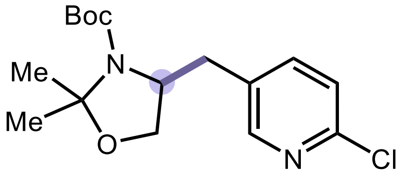

*Prepared according to **General Procedure A** with the following modifications: **NMP** instead of **DMA** was used as the optimal solvent.*

Prepared according to **General Procedure A** using (*S*)-3-(*tert*-butoxycarbonyl)-2,2-dimethyloxazolidine-4-carboxylic acid (62.6 mg, 0.25 mmol, 1.0 equiv.), 1-((6-chloropyridin-3-yl)methyl)-2,4,6-triphenylpyridin-1-ium tetrafluoroborate (195.3 mg, 0.375 mmol, 1.5 equiv.), K_2CO_3 (86.4 mg, 0.625 mmol, 2.5 equiv.), 4CzIPN (9.9 mg, 12.5 μmol, 5.0 mol%), Fe(OEP)Cl (7.8 mg, 12.5 μmol, 5.0 mol%), NMP (2.5 mL), and *i*-PrOH (2.5 mL).

After work-up according to **General Procedure A**, the crude residue was purified by automated flash column chromatography (25 g silica column, 0-20-40% EtOAc/hexanes) followed by automated reverse-phase column chromatography (30 g C18 column, 10-60% MeCN/H_2O with 0.1% NH_4OH) to afford the title compound as a light-yellow gum (56.2 mg, 66% yield).

^{1}H NMR (500 MHz, $CDCl_3$) δ 8.25 (d, *J* = 2.4 Hz, 1H), 7.62 – 7.42 (m, 1H), 7.28 (d, *J* = 8.7 Hz, 1H), 4.16 – 3.90 (m, 1H), 3.88 – 3.80 (m, 1H), 3.69 (dd, *J* = 9.2, 1.2 Hz, 1H), 3.19 – 2.96 (m, 1H), 2.82 – 2.67 (m, 1H), 1.56 (s, 6H), 1.50 (s, 9H). **(summary of rotamers)**

^{13}C NMR (126 MHz, $CDCl_3$) δ 152.34, 151.58, 150.40, 149.95, 149.85, 139.99, 139.63, 132.91, 132.88, 124.30, 124.17, 94.47, 93.92, 80.63, 80.20, 66.16, 65.93, 58.64, 58.52, 36.35, 35.22, 28.62, 28.52, 27.57, 26.97, 24.50, 23.23. **(summary of rotamers)**

IR (film) ν_{max} 2979, 2935, 2876, 1690, 1478, 1459, 1387, 1376, 1364, 1259, 1207, 1170, 1148, 1103, 1092, 1074, 1054, 1022, 854, 837, 815, 768 cm^{-1}.

HRMS (ESI-TOF) *m/z* calculated for $C_{16}H_{24}ClN_2O_3^+$ ($[M+H]^+$) 327.1470, found 327.1474.

2-chloro-5-(((3a*S*,4*S*,6*S*,6a*S*)-6-methoxy-2,2-dimethyltetrahydrofuro[3,4-*d*][1,3]dioxol-4-yl)methyl)pyridine (33):

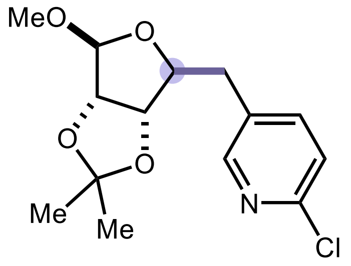

Prepared according to **General Procedure A** using (3a*S*,4*S*,6*R*,6a*R*)-6-methoxy-2,2-dimethyltetrahydrofuro[3,4-*d*][1,3]dioxole-4-carboxylic acid (54.6 mg, 0.25 mmol, 1.0 equiv.), 1-((6-chloropyridin-3-yl)methyl)-2,4,6-triphenylpyridin-1-ium tetrafluoroborate (195.3 mg, 0.375 mmol, 1.5 equiv.), K_2CO_3 (86.4 mg, 0.625 mmol, 2.5 equiv.), 4CzIPN (9.9 mg, 12.5 µmol, 5.0 mol%), Fe(OEP)Cl (7.8 mg, 12.5 µmol, 5.0 mol%), DMA (2.5 mL), and *i*-PrOH (2.5 mL).

After work-up according to **General Procedure A**, the crude residue was purified by automated flash column chromatography (25 g silica column, 0-5-10-20% EtOAc/hexanes) followed by automated reverse-phase column chromatography (30 g C18 column, 10-45% $MeCN/H_2O$ with 0.1% NH_4OH) to afford the title compound as a white amorphous solid, (60.7 mg, 81% yield).

^{1}H NMR (500 MHz, $CDCl_3$) δ 8.26 (d, *J* = 2.5 Hz, 1H), 7.56 (dd, *J* = 8.2, 2.3 Hz, 1H), 7.29 (d, *J* = 8.2 Hz, 1H), 4.97 (s, 1H), 4.68 (d, *J* = 5.9 Hz, 1H), 4.60 (dd, *J* = 6.0, 1.2 Hz, 1H), 4.42 (ddd, *J* = 8.4, 6.8, 1.1 Hz, 1H), 3.35 (s, 3H), 2.94 (dd, *J* = 14.4, 8.7 Hz, 1H), 2.85 (dd, *J* = 14.5, 6.9 Hz, 1H), 1.47 (s, 3H), 1.31 (s, 3H).

^{13}C NMR (126 MHz, $CDCl_3$) δ 150.30, 149.81, 139.22, 132.61, 124.22, 112.72, 110.14, 87.04, 85.53, 83.48, 55.52, 37.64, 26.54, 25.07.

IR (film) ν_{max} 3039, 2986, 2933, 2830, 1588, 1565, 1459, 1379, 1273, 1209, 1105, 1087, 1070, 1050, 1039, 1023, 999, 956, 868, 825, 740, 641 cm^{-1}.

HRMS (ESI-TOF) *m/z* calculated for $C_{14}H_{19}ClNO_4^+$ ($[M+H]^+$) 300.0997, found 300.1001.

***tert*-butyl 4-methyl-4-((6-(trifluoromethyl)pyridin-3-yl)methyl)piperidine-1-carboxylate (34):**

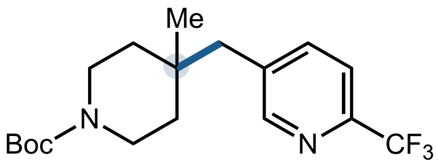

Prepared according to **General Procedure B** using *tert*-butyl 4-hydroxy-4-methylpiperidine-1-carboxylate (215.3 mg, 1.0 mmol, 2.0 equiv.), **NHC** (563.1 mg, 1.10 mmol, 2.2 equiv.), pyridine (87 μL, 1.08 mmol, 2.15 equiv.), $PhCF_3$ (10.0 mL), 2,4,6-triphenyl-1-((6-(trifluoromethyl)pyridin-3-yl)methyl)pyridin-1-ium tetrafluoroborate (277.2 mg, 0.5 mmol, 1.0 equiv.), KOPiv (280.4 mg, 2.0 mmol, 4.0 equiv.), Ir[(dFCF$_3$ppy)$_2$(dtbbpy)]PF$_6$ (8.4 mg, 7.5 μmol, 1.5 mol%), Fe(OEP)Cl (15.6 mg, 12.5 μmol, 2.0 mol%), acetone (5.0 mL), and *i*-PrOH (5.0 mL).

After irradiation, the reaction was concentrated under reduced pressure, and the resulting crude residue was purified by automated flash column chromatography (50 g silica column, 0-35% EtOAc/hexanes) followed by automated reverse-phase column chromatography (30 g C18 column, 45-85% MeCN/H_2O with 0.1% NH_4OH) to afford the title compound as a light-yellow oil (128.7 mg, 72% yield).

^{1}H NMR (500 MHz, $CDCl_3$) δ 8.48 (d, J = 1.6 Hz, 1H), 7.60 (t, J = 1.3 Hz, 2H), 3.73 (dt, J = 13.7, 4.4 Hz, 2H), 3.10 (ddd, J = 13.8, 10.3, 3.4 Hz, 2H), 2.63 (s, 2H), 1.44 (s, 9H), 1.38 – 1.28 (m, 2H), 1.35 – 1.23 (m, 2H), 0.92 (s, 3H).

^{13}C NMR (126 MHz, $CDCl_3$) δ 155.00, 151.63, 146.35 (q, J = 34.7 Hz), 139.08, 137.01, 137.00, 121.80 (q, J = 273.8 Hz), 119.82 (q, J = 2.9 Hz), 79.64, 45.86, 39.83, 36.54, 33.07, 28.55, 22.44.

^{19}F NMR (471 MHz, $CDCl_3$) δ -67.74.

IR (film) ν_{max} 2973, 2987, 1687, 1423, 1337, 1247, 1135, 1085, 1027, 857, 770 cm^{-1}.

HRMS (ESI-TOF) *m/z* calculated for $C_{14}H_{18}F_3N_2O_2^+$ ($[M+H–isobutylene]^+$) 303.1315, found 303.1315.

***tert*-butyl 4-((6-bromopyridin-3-yl)methyl)-4-methylpiperidine-1-carboxylate (35):**

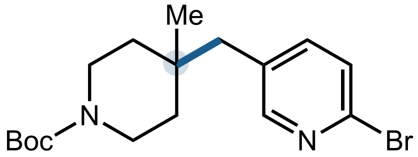

Prepared according to **General Procedure B** using *tert*-butyl 4-hydroxy-4-methylpiperidine-1-carboxylate (107.6 mg, 0.5 mmol, 2.0 equiv.), **NHC** (281.5 mg, 0.55 mmol, 2.2 equiv.), pyridine (43 μL, 0.538 mmol, 2.15 equiv.), $PhCF_3$ (2.5 mL), 1-((6-bromopyridin-3-yl)methyl)-2,4,6-triphenylpyridin-1-ium tetrafluoroborate (146.4 mg, 0.25 mmol, 1.0 equiv.), KOPiv (140.2 mg, 1.0 mmol, 4.0 equiv.), $Ir[(dFCF_3ppy)_2(dtbbpy)]PF_6$ (4.2 mg, 3.75 μmol, 1.5 mol%), Fe(OEP)Cl (3.9 mg, 6.25 μmol, 2.5 mol%), acetone (2.5 mL), and *i*-PrOH (2.5 mL).

After irradiation, the reaction was concentrated under reduced pressure, and the resulting crude residue was purified by automated flash column chromatography (25 g silica column, 0-18-22% EtOAc/hexanes) to afford the title compound as an off-white solid (59.1 mg, 64% yield).

^{1}H NMR (500 MHz, $CDCl_3$) δ 8.15 (s, 1H), 7.41 (d, J = 8.1 Hz, 1H), 7.31 (dd, J = 8.1, 2.3 Hz, 1H), 3.73 (dd, J = 13.6, 5.0 Hz, 2H), 3.10 (ddd, J = 13.7, 10.3, 3.4 Hz, 2H), 2.52 (s, 2H), 1.45 (s, 9H), 1.44 – 1.37 (td, J = 9.8, 5.1 Hz, 1H), 1.32 – 1.24 (m, 1H), 0.90 (s, 3H).

^{13}C NMR (126 MHz, $CDCl_3$) δ 154.99, 151.68, 140.58, 140.06, 132.81, 127.38, 79.58, 45.23, 40.14, 39.53, 36.49, 32.80, 28.56, 22.44.

IR (film) ν_{max} 2972, 2926, 2870, 1684, 1476, 1453, 1421, 1382, 1364, 1276, 1245, 1157, 1085, 1021, 995, 813, 769, 742, 732 cm^{-1}.

HRMS (ESI-TOF) *m/z* calculated for $C_{17}H_{25}BrN_2NaO_2^+$ ($[M+Na]^+$) 391.0992, found 391.0986.

***tert*-butyl 4-((2-fluoropyridin-3-yl)methyl)-4-methylpiperidine-1-carboxylate (36):**

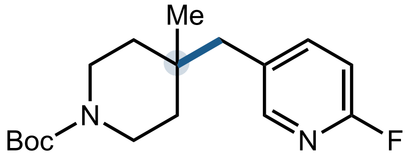

Prepared according to **General Procedure B** using *tert*-butyl 4-hydroxy-4-methylpiperidine-1-carboxylate (107.6 mg, 0.5 mmol, 2.0 equiv.), **NHC** (281.5 mg, 0.55 mmol, 2.2 equiv.), pyridine (43 µL, 0.538 mmol, 2.15 equiv.), $PhCF_3$ (2.5 mL), 1-((2-fluoropyridin-3-yl)methyl)-2,4,6-triphenylpyridin-1-ium tetrafluoroborate (126.1 mg, 0.25 mmol, 1.0 equiv.), KOPiv (140.2 mg, 1.0 mmol, 4.0 equiv.), $Ir[(dFCF_3ppy)_2(dtbbpy)]PF_6$ (4.2 mg, 3.75 µmol, 1.5 mol%), Fe(OEP)Cl (3.9 mg, 6.25 µmol, 2.5 mol%), acetone (2.5 mL), and *i*-PrOH (2.5 mL).

After irradiation, the reaction was concentrated under reduced pressure, and the resulting crude residue was purified by automated flash column chromatography (25 g silica column, 0-15-24% EtOAc/hexanes) followed by automated reverse-phase column chromatography (30 g C18 column, 10-77% MeCN/H_2O with 0.1% NH_4OH) to afford the title compound as a light-yellow oil (41.6 mg, 54% yield).

^{1}H NMR (500 MHz, $CDCl_3$) δ 8.09 (ddd, *J* = 5.0, 2.0, 1.1 Hz, 1H), 7.53 (ddd, *J* = 9.4, 7.3, 2.0 Hz, 1H), 7.12 (ddd, *J* = 7.0, 4.8, 1.8 Hz, 1H), 3.71 (dt, *J* = 13.7, 4.7 Hz, 2H), 3.12 (ddd, *J* = 13.6, 10.2, 3.4 Hz, 2H), 2.59 (s, 2H), 1.44 (s, 9H), 1.50 – 1.39 (m, 2H), 1.36 – 1.27 (m, 2H), 0.93 (s, 3H).

^{13}C NMR (126 MHz, $CDCl_3$) δ 162.61 (d, *J* = 237.9 Hz), 155.01, 145.78 (d, *J* = 14.6 Hz), 143.27 (d, *J* = 5.6 Hz), 121.07 (d, *J* = 4.2 Hz), 120.07 (d, *J* = 31.4 Hz), 79.47, 41.00 (d, *J* = 3.0 Hz), 40.22, 39.56, 36.41, 33.51, 28.55, 22.61 (d, *J* = 1.4 Hz).

^{19}F NMR (376 MHz, $CDCl_3$) δ -69.69 (d, *J* = 9.5 Hz, 1F).

IR (film) ν_{max} 2972, 2928, 2873, 1685, 1476, 1432, 1365, 1277, 1244, 1156, 1126, 1095, 995, 811, 781, 754 cm^{-1}.

HRMS (ESI-TOF) *m/z* calculated for $C_{17}H_{25}FN_2NaO_2^+$ ($[M+Na]^+$) 331.1792, found 331.1789.

***tert*-butyl 4-((6-methoxypyridin-3-yl)methyl)-4-methylpiperidine-1-carboxylate (37):**

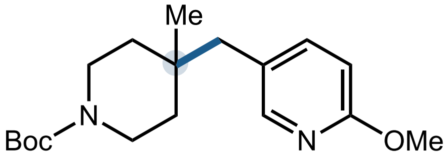

Prepared according to **General Procedure B** using *tert*-butyl 4-hydroxy-4-methylpiperidine-1-carboxylate (107.6 mg, 0.5 mmol, 2.0 equiv.), **NHC** (281.5 mg, 0.55 mmol, 2.2 equiv.), pyridine (43 μL, 0.538 mmol, 2.15 equiv.), $PhCF_3$ (2.5 mL), 1-((6-methoxypyridin-3-yl)methyl)-2,4,6-triphenylpyridin-1-ium tetrafluoroborate (129.1 mg, 0.25 mmol, 1.0 equiv.), KOPiv (140.2 mg, 1.0 mmol, 4.0 equiv.), $Ir[(dFCF_3ppy)_2(dtbbpy)]PF_6$ (4.2 mg, 3.75 μmol, 1.5 mol%), Fe(OEP)Cl (3.9 mg, 6.25 μmol, 2.5 mol%), acetone (2.5 mL), and *i*-PrOH (2.5 mL).

After irradiation, the reaction was concentrated under reduced pressure, and the resulting crude residue was purified by automated flash column chromatography (25 g silica column, 0-15-20% EtOAc/hexanes) followed by automated reverse-phase column chromatography (30 g C18 column, 10-72% $MeCN/H_2O$ with 0.1% NH_4OH) to afford the title compound as a light-yellow oil (46.1 mg, 58% yield).

^{1}H NMR (500 MHz, $CDCl_3$) δ 7.94 (d, J = 2.3 Hz, 1H), 7.42 (dd, J = 8.7, 2.4 Hz, 1fH), 6.73 (d, J = 8.5 Hz, 1H), 3.98 (s, 3H), 3.80 – 3.60 (m, 2H), 3.11 (ddd, J = 13.7, 10.2, 3.4 Hz, 2H), 2.50 (s, 2H), 1.45 (s, 9H), 1.50 – 1.36 (m, 2H), 1.32 – 1.23 (m, 2H), 0.90 (s, 3H).

^{13}C NMR (126 MHz, $CDCl_3$) δ 162.96, 155.05, 147.87, 140.93, 125.99, 110.00, 79.42, 53.38, 44.83, 40.24, 39.61, 36.48, 32.73, 28.57, 22.71.

IR (film) ν_{max} 3006, 2973, 2927, 2870, 2846, 1691, 1605, 1492, 1422, 1392, 1365, 1288, 1259, 1248, 1159, 1028 cm^{-1}.

HRMS (ESI-TOF) *m/z* calculated for $C_{18}H_{29}N_2O_3^+$ ([M+H]$^+$) 321.2173, found 321.2170.

***tert*-butyl 4-((2-methoxypyridin-3-yl)methyl)-4-methylpiperidine-1-carboxylate (38):**

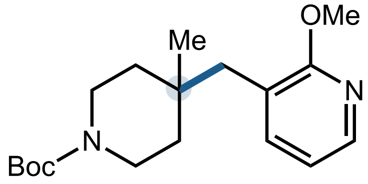

Prepared according to **General Procedure B** using *tert*-butyl 4-hydroxy-4-methylpiperidine-1-carboxylate (215.3 mg, 1.0 mmol, 2.0 equiv.), **NHC** (563.1 mg, 1.10 mmol, 2.2 equiv.), pyridine (87 μL, 1.08 mmol, 2.15 equiv.), $PhCF_3$ (10.0 mL), 1-((2-methoxypyridin-3-yl)methyl)-2,4,6-triphenylpyridin-1-ium tetrafluoroborate (258.2 mg, 0.5 mmol, 1.0 equiv.), KOPiv (280.4 mg, 2.0 mmol, 4.0 equiv.), $Ir[(dFCF_3ppy)_2(dtbbpy)]PF_6$ (8.4 mg, 7.5 μmol, 1.5 mol%), Fe(OEP)Cl (15.6 mg, 12.5 μmol, 2.0 mol%), acetone (5.0 mL), and *i*-PrOH (5.0 mL).

After irradiation, the reaction was concentrated under reduced pressure, and the resulting crude residue was purified by automated flash column chromatography (3 × 10 g silica column, 0-50% Et_2O/hexanes) followed by automated reverse-phase column chromatography (30 g C18 column, 55-75% $MeCN/H_2O$ with 0.1% NH_4OH) to afford the title compound as a light-yellow oil (117.6 mg, 73% yield).

^{1}H NMR (500 MHz, $CDCl_3$) δ 8.02 (dd, *J* = 5.1, 1.9 Hz, 1H), 7.30 (dd, *J* = 7.2, 1.9 Hz, 1H), 6.79 (dd, *J* = 7.2, 5.0 Hz, 1H), 3.90 (s, 3H), 3.69 – 3.61 (m, 2H), 3.16 (ddd, *J* = 13.4, 9.6, 3.5 Hz, 2H), 2.56 (s, 2H), 1.44 (s, 9H), 1.42 – 1.36 (m, 2H), 1.30 – 1.22 (m, 2H), 0.88 (s, 3H).

^{13}C NMR (126 MHz, $CDCl_3$) δ 162.86, 155.11, 144.77, 140.69, 121.14, 116.35, 79.32, 53.28, 40.84, 40.36, 39.71, 36.60, 33.61, 28.59, 23.36.

IR (film) ν_{max} 2928, 1689, 1583, 1464, 1412, 1364, 1247, 1158, 1022, 782 cm^{-1}.

HRMS (ESI-TOF) *m/z* calculated for $C_{14}H_{21}N_2O_3^+$ ([M+H–isobutylene]$^+$) 265.1546, found 265.1549.

***tert*-butyl 4-((6-methoxypyridin-2-yl)methyl)-4-methylpiperidine-1-carboxylate (39):**

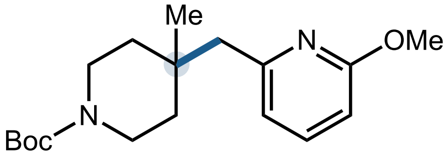

Prepared according to **General Procedure B** using *tert*-butyl 4-hydroxy-4-methylpiperidine-1-carboxylate (215.3 mg, 1.0 mmol, 2.0 equiv.), **NHC** (563.1 mg, 1.10 mmol, 2.2 equiv.), pyridine (87 µL, 1.08 mmol, 2.15 equiv.), $PhCF_3$ (10.0 mL), 1-((6-methoxypyridin-2-yl)methyl)-2,4,6-triphenylpyridin-1-ium tetrafluoroborate (258.2 mg, 0.5 mmol, 1.0 equiv.), KOPiv (280.4 mg, 2.0 mmol, 4.0 equiv.), $Ir[(dFCF_3ppy)_2(dtbbpy)]PF_6$ (8.4 mg, 7.5 µmol, 1.5 mol%), Fe(OEP)Cl (15.6 mg, 12.5 µmol, 2.0 mol%), acetone (5.0 mL), and *i*-PrOH (5.0 mL).

After irradiation, the reaction was concentrated under reduced pressure, and the resulting crude residue was purified by automated flash column chromatography (3 × 10 g silica column, 0-20% EtOAc/hexanes) followed by automated flash column chromatography (3 × 10 g silica column, 0-15% acetone/hexanes) and automated reverse-phase column chromatography (30 g C18 column, 60-80% $MeCN/H_2O$ with 0.1% NH_4OH) to afford the title compound as a light-yellow oil (113.0 mg, 71% yield).

^{1}H NMR (500 MHz, $CDCl_3$) δ 7.44 (t, *J* = 7.2 Hz, 1H), 6.65 (d, *J* = 7.2 Hz, 1H), 6.55 (d, *J* = 8.3 Hz, 1H), 3.87 (s, 3H), 3.63 – 3.54 (m, 2H), 3.33 (ddd, *J* = 13.6, 8.3, 3.8 Hz, 2H), 2.65 (s, 2H), 1.59 – 1.48 (m, 2H), 1.45 (s, 9H), 1.36 – 1.27 (m, 2H), 0.95 (s, 3H).

^{13}C NMR (126 MHz, $CDCl_3$) δ 163.25, 157.01, 155.18, 138.32, 117.62, 107.74, 79.28, 53.28, 48.28, 40.41, 39.70, 36.92, 33.16, 28.61, 24.66.

IR (film) ν_{max} 2926, 1690, 1579, 1465, 1413, 1247, 1158, 1030, 807, 732 cm^{-1}.

HRMS (ESI-TOF) *m/z* calculated for $C_{14}H_{21}N_2O_3^+$ ([M+H–isobutylene]$^+$) 265.1546, found 265.1547.

tert-butyl 4-((2-methoxypyridin-4-yl)methyl)-4-methylpiperidine-1-carboxylate (40):

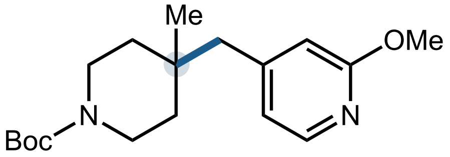

Prepared according to **General Procedure B** using *tert*-butyl 4-hydroxy-4-methylpiperidine-1-carboxylate (215.3 mg, 1.0 mmol, 2.0 equiv.), **NHC** (563.1 mg, 1.10 mmol, 2.2 equiv.), pyridine (87 μL, 1.08 mmol, 2.15 equiv.), $PhCF_3$ (10.0 mL), 1-((2-methoxypyridin-4-yl)methyl)-2,4,6-triphenylpyridin-1-ium tetrafluoroborate (258.2 mg, 0.5 mmol, 1.0 equiv.), KOPiv (280.4 mg, 2.0 mmol, 4.0 equiv.), $Ir[(dFCF_3ppy)_2(dtbbpy)]PF_6$ (8.4 mg, 7.5 μmol, 1.5 mol%), Fe(OEP)Cl (15.6 mg, 12.5 μmol, 2.0 mol%), acetone (5.0 mL), and *i*-PrOH (5.0 mL).

After irradiation, the reaction was concentrated under reduced pressure, and the resulting crude residue was purified by automated flash column chromatography (50 g silica column, 0-20% acetone/hexanes) followed by automated reverse-phase column chromatography (60 g C18 column, 50-75% $MeCN/H_2O$ with 0.1% NH_4OH) to afford the title compound as a light-yellow oil (113.6 mg, 71% yield).

^{1}H NMR (500 MHz, $CDCl_3$) δ 8.02 (d, *J* = 5.2 Hz, 1H), 6.62 (dd, *J* = 5.2, 1.4 Hz, 1H), 6.48 (s, 1H), 3.91 (s, 3H), 3.70 – 3.63 (m, 2H), 3.11 (ddd, *J* = 13.6, 10.0, 3.5 Hz, 2H), 2.48 (s, 2H), 1.43 (s, 9H), 1.43 – 1.38 (m, 2H), 1.32 – 1.20 (m, 2H), 0.90 (s, 3H).

^{13}C NMR (126 MHz, $CDCl_3$) δ 164.22, 155.03, 149.97, 146.05, 119.73, 112.64, 79.48, 53.43, 47.98, 40.24, 39.63, 36.74, 32.84, 28.56, 22.96.

IR (film) ν_{max} 2972, 2927, 1688, 1609, 1559, 1396, 1245, 1156, 1044, 990, 829, 773, 729, 621 cm^{-1}.

HRMS (ESI-TOF) *m/z* calculated for $C_{14}H_{21}N_2O_3^+$ ($[M+H–isobutylene]^+$) 265.1546, found 265.1547.

***tert*-butyl 4-((2-methoxypyrimidin-5-yl)methyl)-4-methylpiperidine-1-carboxylate (41):**

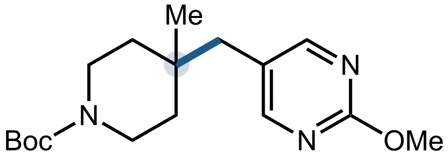

Prepared according to **General Procedure B** using *tert*-butyl 4-hydroxy-4-methylpiperidine-1-carboxylate (215.3 mg, 1.0 mmol, 2.0 equiv.), **NHC** (563.1 mg, 1.10 mmol, 2.2 equiv.), pyridine (87 μL, 1.08 mmol, 2.15 equiv.), $PhCF_3$ (10.0 mL), 1-((2-methoxypyrimidin-5-yl)methyl)-2,4,6-triphenylpyridin-1-ium tetrafluoroborate (258.7 mg, 0.5 mmol, 1.0 equiv.), KOPiv (280.4 mg, 2.0 mmol, 4.0 equiv.), $Ir[(dFCF_3ppy)_2(dtbbpy)]PF_6$ (8.4 mg, 7.5 μmol, 1.5 mol%), Fe(OEP)Cl (15.6 mg, 12.5 μmol, 2.0 mol%), acetone (5.0 mL), and *i*-PrOH (5.0 mL).

After irradiation, the reaction was concentrated under reduced pressure, and the resulting crude residue was purified by automated flash column chromatography (3 × 10 g silica column, 0-20-60% EtOAc/hexanes) followed by automated reverse-phase column chromatography (30 g C18 column, 40-65% $MeCN/H_2O$ with 0.1% NH_4OH) to afford the title compound as a light-yellow oil (135.9 mg, 85% yield).

^{1}H NMR (500 MHz, $CDCl_3$) δ 8.25 (s, 2H), 3.97 (s, 3H), 3.73 – 3.66 (m, 2H), 3.08 (ddd, *J* = 13.8, 10.3, 3.4 Hz, 2H), 2.44 (s, 2H), 1.42 (s, 9H), 1.40 – 1.32 (m, 2H), 1.30 – 1.20 (m, 2H), 0.88 (s, 3H).

^{13}C NMR (126 MHz, $CDCl_3$) δ 164.66, 160.24, 154.95, 124.12, 79.54, 54.87, 42.32, 39.71, 36.32, 32.64, 28.52, 22.33.

IR (film) ν_{max} 2972, 2925, 1687, 1598, 1556, 1471, 1405, 1324, 1246, 1157, 1034, 806, 657 cm^{-1}.

HRMS (ESI-TOF) *m/z* calculated for $C_{17}H_{27}N_3NaO_3^+$ ([M+Na]$^+$) 344.1945, found 344.1947.

tert-butyl 4-((6-chloro-1-isopropyl-1H-pyrazolo[3,4-b]pyridin-5-yl)methyl)-4-methylpiperidine-1-carboxylate (42):

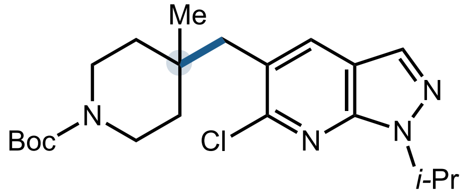

Prepared according to **General Procedure B** using *tert*-butyl 4-hydroxy-4-methylpiperidine-1-carboxylate (107.6 mg, 0.5 mmol, 2.0 equiv.), **NHC** (281.5 mg, 0.55 mmol, 2.2 equiv.), pyridine (43 µL, 0.538 mmol, 2.15 equiv.), $PhCF_3$ (2.5 mL), 1-((6-chloro-1-isopropyl-1*H*-pyrazolo[3,4-*b*]pyridin-5-yl)methyl)-2,4,6-triphenylpyridin-1-ium tetrafluoroborate (153.8 mg, 0.25 mmol, 1.0 equiv.), KOPiv (140.2 mg, 1.0 mmol, 4.0 equiv.), $Ir[(dFCF_3ppy)_2(dtbbpy)]PF_6$ (4.2 mg, 3.75 µmol, 1.5 mol%), Fe(OEP)Cl (3.9 mg, 6.25 µmol, 2.5 mol%), acetone (2.5 mL), and *i*-PrOH (2.5 mL).

After irradiation, the reaction was concentrated under reduced pressure, and the resulting crude residue was purified by automated flash column chromatography (25 g silica column, 0-15-20% EtOAc/hexanes) followed by automated flash column chromatography (25 g silica column, 0-5-40% Et_2O/DCM) to afford the title compound as a light yellow solid (71.5 mg, 70% yield).

^{1}H NMR (500 MHz, $CDCl_3$) δ 7.96 (s, 1H), 7.82 (s, 1H), 5.23 (hept, J = 6.8 Hz, 1H), 3.81 (dt, J = 13.8, 4.4 Hz, 2H), 3.05 (ddd, J = 14.0, 11.1, 3.2 Hz, 2H), 2.87 (s, 2H), 1.58 (s, 3H), 1.57 (s, 3H), 1.55 – 1.51 (m, 2H *overlapped with H_2O peak*), 1.44 (s, 3H), 1.41 – 1.34 (m, 2H), 1.01 (s, 3H).

^{13}C NMR (126 MHz, $CDCl_3$) δ 155.05, 151.53, 147.55, 133.72, 131.39, 124.67, 114.64, 79.48, 48.72, 45.26, 40.26, 39.58, 36.44, 34.27, 28.56, 22.24, 22.13.

IR (film) ν_{max} 2975, 2930, 2871, 1684, 1607, 1424, 1403, 1390, 1365, 1277, 1262, 1246, 1156, 1115, 1096, 1076, 1039, 1025, 996, 912, 837, 769, 730, 646 cm^{-1}.

HRMS (ESI-TOF) *m/z* calculated for $C_{21}H_{31}ClN_4NaO_2^+$ ([M+Na]$^+$) 429.2028, found 429.2030.

***tert*-butyl 4-((2-(4-fluorophenyl)thiazol-4-yl)methyl)-4-methylpiperidine-1-carboxylate (43):**

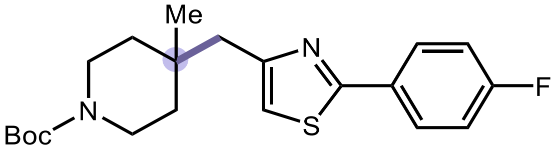

Prepared according to **General Procedure B** using *tert*-butyl 4-hydroxy-4-methylpiperidine-1-carboxylate (107.6 mg, 0.5 mmol, 2.0 equiv.), **NHC** (281.5 mg, 0.55 mmol, 2.2 equiv.), pyridine (43 μL, 0.538 mmol, 2.15 equiv.), $PhCF_3$ (2.5 mL), 1-((2-(4-fluorophenyl)thiazol-4-yl)methyl)-2,4,6-triphenylpyridin-1-ium tetrafluoroborate (148.1 mg, 0.25 mmol, 1.0 equiv.), KOPiv (140.2 mg, 1.0 mmol, 4.0 equiv.), $Ir[(dFCF_3ppy)_2(dtbbpy)]PF_6$ (4.2 mg, 3.75 μmol, 1.5 mol%), Fe(OEP)Cl (3.9 mg, 6.25 μmol, 2.5 mol%), acetone (2.5 mL), and *i*-PrOH (2.5 mL).

After irradiation, the reaction was concentrated under reduced pressure, and the resulting crude residue was purified by automated flash column chromatography (25 g silica column, 0-10-20% Et_2O/hexanes) followed by preparative HPLC (Waters Xbridge BEH C18 OBD Prep column, 10-70% MeCN/H_2O with 0.1% NH_4OH), and a second automated flash column chromatography (25 g silica column, 0-20% Et_2O/DCM) to afford the title compound as a light yellow gum (57.2 mg, 59% yield).

All characterization data are identical to those reported for compound **18**.

(4-((6-chloropyridin-3-yl)methyl)-4-methylpiperidin-1-yl)(3,5-di-*tert*-butylphenyl)methanone (44):

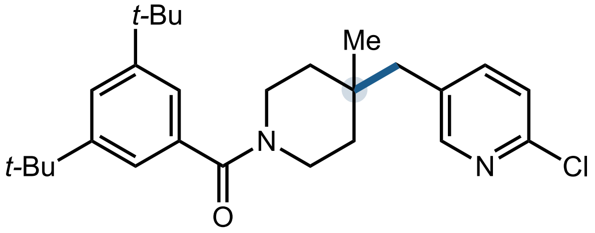

Prepared according to **General Procedure B** using (3,5-di-*tert*-butylphenyl)(4-hydroxy-4-methylpiperidin-1-yl)methanone (331.5 mg, 1.0 mmol, 2.0 equiv.), **NHC** (563.1 mg, 1.10 mmol, 2.2 equiv.), pyridine (87 μL, 1.08 mmol, 2.15 equiv.), $PhCF_3$ (10.0 mL), 1-((6-chloropyridin-3-yl)methyl)-2,4,6-triphenylpyridin-1-ium tetrafluoroborate (260.4 mg, 0.5 mmol, 1.0 equiv.), KOPiv (280.4 mg, 2.0 mmol, 4.0 equiv.), $Ir[(dFCF_3ppy)_2(dtbbpy)]PF_6$ (8.4 mg, 7.5 μmol, 1.5 mol%), Fe(OEP)Cl (15.6 mg, 12.5 μmol, 2.0 mol%), acetone (5.0 mL), and *i*-PrOH (5.0 mL).

After irradiation, the reaction was concentrated under reduced pressure, and the resulting crude residue was purified by automated flash column chromatography (50 g silica column, 0-40% EtOAc/hexanes) followed by automated reverse-phase column chromatography (60 g C18 column, 50-100% $MeCN/H_2O$ with 0.1% NH_4OH) to afford the title compound as a light-yellow foam (156.2 mg, 71% yield).

^{1}H NMR (500 MHz, $CDCl_3$) δ 8.14 (d, *J* = 2.5 Hz, 1H), 7.44 (t, *J* = 1.9 Hz, 1H), 7.40 (dd, *J* = 8.2, 2.5 Hz, 1H), 7.23 (d, *J* = 8.1 Hz, 1H), 7.18 (d, *J* = 1.8 Hz, 2H), 4.23 (s, 1H), 3.54 (s, 1H), 3.28 (s, 2H), 2.58 (s, 2H), 1.64 – 1.14 (m, 22H), 0.96 (s, 3H).

^{13}C NMR (126 MHz, $CDCl_3$) δ 171.45, 150.97, 149.59, 140.61, 135.21, 132.07, 123.62, 123.55, 120.99, 45.05, 44.00, 38.24, 37.30, 36.30, 34.92, 33.15, 31.39, 22.54.

IR (film) ν_{max} 2972, 2925, 1687, 1598, 1556, 1471, 1405, 1324, 1246, 1157, 1034, 806, 657 cm^{-1}.

HRMS (ESI-TOF) *m/z* calculated for $C_{27}H_{37}ClN_2O^+$ ($[M+H]^+$) 441.2667, found 441.2668.

***tert*-butyl 4-methyl-4-(4-(4-methylthiazol-5-yl)benzyl)piperidine-1-carboxylate (45):**

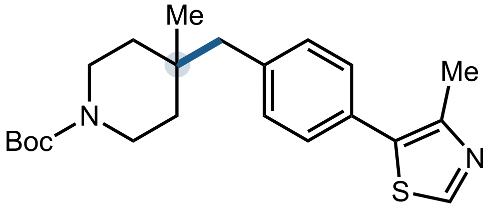

Prepared according to **General Procedure B** using *tert*-butyl 4-hydroxy-4-methylpiperidine-1-carboxylate (215.3 mg, 1.0 mmol, 2.0 equiv.), **NHC** (563.1 mg, 1.10 mmol, 2.2 equiv.), pyridine (87 µL, 1.08 mmol, 2.15 equiv.), $PhCF_3$ (10.0 mL), 1-(4-(4-methylthiazol-5-yl)benzyl)-2,4,6-triphenylpyridin-1-ium tetrafluoroborate (291.2 mg, 0.5 mmol, 1.0 equiv.), KOPiv (280.4 mg, 2.0 mmol, 4.0 equiv.), $Ir[(dFCF_3ppy)_2(dtbbpy)]PF_6$ (8.4 mg, 7.5 µmol, 1.5 mol%), Fe(OEP)Cl (15.6 mg, 12.5 µmol, 2.0 mol%), acetone (5.0 mL), and *i*-PrOH (5.0 mL).

After irradiation, the reaction was concentrated under reduced pressure, and the resulting crude residue was purified by automated flash column chromatography (3 × 10 g silica column, 5-40% EtOAc/hexanes) followed by automated reverse-phase column chromatography (30 g C18 column, 65-85% $MeCN/H_2O$ with 0.1% NH_4OH) to afford the title compound as a light-yellow oil (126.7 mg, 66% yield).

^{1}H NMR (500 MHz, $CDCl_3$) δ 8.67 (s, 1H), 7.34 (d, *J* = 8.2 Hz, 1H), 7.16 (d, *J* = 8.1 Hz, 2H), 3.74 – 3.68 (m, 2H), 3.14 (ddd, *J* = 13.6, 10.1, 3.4 Hz, 2H), 2.59 (s, 2H), 2.54 (s, 3H), 1.52 – 1.46 (m, 2H), 1.45 (s, 9H), 1.35 – 1.27 (m, 2H), 0.94 (s, 3H).

^{13}C NMR (126 MHz, $CDCl_3$) δ 155.12, 150.22, 148.33, 138.12, 132.05, 131.12, 129.79, 128.77, 79.42, 48.46, 40.32, 39.75, 36.68, 32.99, 28.59, 22.96, 16.24.

IR (film) ν_{max} 2972, 2923, 1686, 1415, 1365, 1246, 1157, 908, 850, 729, 645, 551 cm^{-1}.

HRMS (ESI-TOF) *m/z* calculated for $C_{22}H_{31}N_2O_2S^+$ ($[M+H]^+$) 387.2101, found 387.2106.

(4-benzyl-4-methylpiperidin-1-yl)(3,5-di-*tert*-butylphenyl)methanone (46):

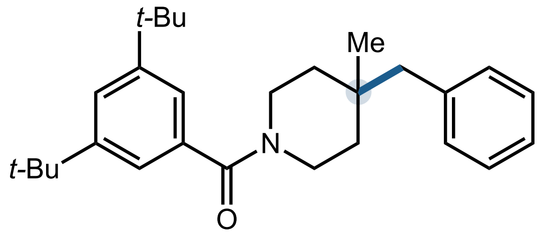

Prepared according to **General Procedure B** using (3,5-di-*tert*-butylphenyl)(4-hydroxy-4-methylpiperidin-1-yl)methanone (331.5 mg, 1.0 mmol, 2.0 equiv.), **NHC** (563.1 mg, 1.10 mmol, 2.2 equiv.), pyridine (87 μL, 1.08 mmol, 2.15 equiv.), $PhCF_3$ (10.0 mL), 1-benzyl-2,4,6-triphenylpyridin-1-ium tetrafluoroborate (242.7 mg, 0.5 mmol, 1.0 equiv.), KOPiv (280.4 mg, 2.0 mmol, 4.0 equiv.), $Ir[(dFCF_3ppy)_2(dtbbpy)]PF_6$ (8.4 mg, 7.5 μmol, 1.5 mol%), Fe(OEP)Cl (15.6 mg, 12.5 μmol, 2.0 mol%), acetone (5.0 mL), and *i*-PrOH (5.0 mL).

After irradiation, the reaction was concentrated under reduced pressure, and the resulting crude residue was purified by automated flash column chromatography (50 g silica column, 0-40% EtOAc/hexanes) followed by automated reverse-phase column chromatography (30 g C18 column, 65-100% MeCN/H_2O with 0.1% NH_4OH) to afford the title compound as an off-whie foam (162.8 mg, 80% yield).

^{1}H NMR (500 MHz, $CDCl_3$) δ 7.45 (t, *J* = 1.9 Hz, 1H), 7.30 – 7.24 (m, 2H), 7.24 – 7.19 (m, 3H), 7.14 – 7.08 (m, 2H), 4.20 (s, 1H), 3.68 – 3.11 (m, 3H), 2.62 (s, 2H), 1.71 – 1.16 (m, 22H), 0.98 (s, 3H).

^{13}C NMR (126 MHz, $CDCl_3$) δ 171.44, 150.93, 137.84, 135.58, 130.72, 127.94, 126.26, 123.50, 121.07, 48.55, 44.27, 38.52, 37.46, 36.53, 34.99, 33.21, 31.48, 23.32.

IR (film) ν_{max} 2961, 2868, 1629, 1594, 1445, 1363, 1245, 1139, 994, 922, 729, 706, 644 cm^{-1}.

HRMS (ESI-TOF) *m/z* calculated for $C_{28}H_{40}NO^+$ ($[M+H]^+$) 406.3104, found 406.3108.

***tert*-butyl 3-((6-chloropyridin-3-yl)methyl)-3-methylpiperidine-1-carboxylate ((±)-47):**

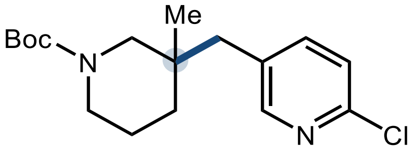

Prepared according to **General Procedure B** using *tert*-butyl 3-hydroxy-3-methylpiperidine-1-carboxylate (226.6 mg, 1.0 mmol, 2.0 equiv.), **NHC** (563.1 mg, 1.10 mmol, 2.2 equiv.), pyridine (87 µL, 1.08 mmol, 2.15 equiv.), $PhCF_3$ (10.0 mL), 2,4,6-triphenyl-1-((6-(trifluoromethyl)pyridin-3-yl)methyl)pyridin-1-ium tetrafluoroborate (277.15 mg, 0.5 mmol, 1.0 equiv.), KOPiv (280.4 mg, 2.0 mmol, 4.0 equiv.), $Ir[(dFCF_3ppy)_2(dtbbpy)]PF_6$ (8.4 mg, 7.5 µmol, 1.5 mol%), Fe(OEP)Cl (15.6 mg, 12.5 µmol, 2.5 mol%), acetone (5.0 mL), and *i*-PrOH (5.0 mL).

After irradiation, the reaction was concentrated under reduced pressure, and the resulting crude residue was purified by automated flash column chromatography (50 g silica column, 0-20-30-80% EtOAc/hexanes) followed by automated reverse-phase column chromatography (30 g C18 column, 10-60-70% $MeCN/H_2O$ with 0.1% NH_4OH) to afford the title compound as a colorless gum (78.5 mg, 48% yield). The assay yield of the reaction was determined to be 61% via UPLC analysis of the crude reaction mixture with 1,3,5-trimethoxybenzene (21 mg, 0.25 mmol, 0.5 equiv.) as the internal standard (see below).

^{1}H NMR (500 MHz, $CDCl_3$) δ 8.17 (d, *J* = 2.5 Hz, 1H), 7.53 – 7.41 (m, 1H), 7.25 (d, *J* = 8.1 Hz, 1H), 3.57 – 3.26 (m, 2H), 3.19 (d, *J* = 13.1 Hz, 1H), 3.16 – 2.96 (m, 1H), 2.57 (d, *J* = 13.7 Hz, 1H), 2.51 (d, *J* = 13.5 Hz, 1H), 1.72 – 1.55 (m, 2H), 1.47 (s, 9H), 1.45 – 1.38 (m, 1H), 1.35 – 1.27 (m, 1H), 0.80 (s, 3H).

^{13}C NMR (126 MHz, $CDCl_3$) δ 155.21, 151.21, 149.58, 140.71, 132.61, 123.61, 79.72, 54.35, 53.18, 44.76, 43.86, 40.85, 35.80, 34.68, 28.59, 23.03, 21.58. **(summary of rotamers)**

IR (film) ν_{max} 2933, 2852, 1687, 1563, 1458, 1424, 1387, 1364, 1277, 1247, 1157, 1102, 1024, 1002, 866, 843, 815, 768, 748, 729 cm^{-1}.

HRMS (ESI-TOF) *m/z* calculated for $C_{17}H_{25}ClN_2NaO_2^+$ ($[M+Na]^+$) 347.1497, found 347.1495.

UPLC traces at 220 nm absorption of the crude reaction mixture

[Product (t_R = 4.17 min), 1,3,5-Trimethoxybenzene (t_R = 2.97 min)]

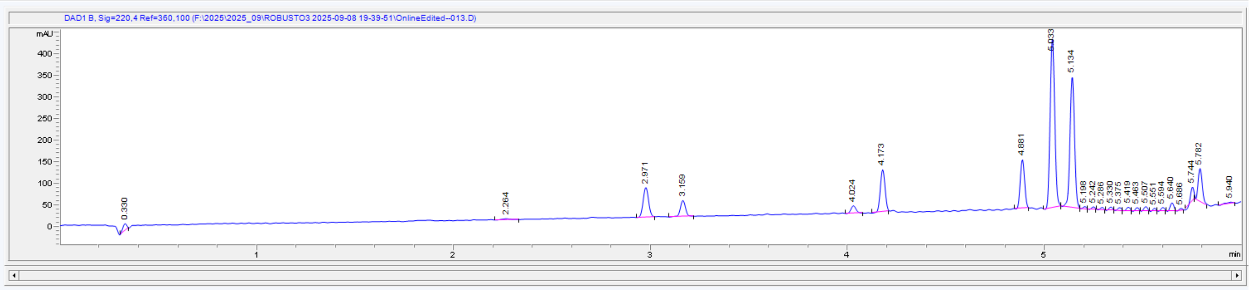

#	Time	Type	Area	Height	Width	Area%	Symmetry
1	0.33	BB	24.3	16.6	0.0251	1.201	1.52
2	2.264	BB	8.3	2.5	0.0458	0.409	0.953
3	2.971	BB	131	68.5	0.0303	6.482	0.952
4	3.159	BB	68.6	37.4	0.0284	3.393	0.977
5	4.024	BB	32.5	17.4	0.0287	1.609	0.84
6	4.173	BB	164.5	97.7	0.0267	8.144	0.97
7	4.881	BB	174.1	113.1	0.024	8.615	0.943
8	5.033	BB	639.2	392.4	0.026	31.636	0.922
9	5.134	BB	487.4	303.9	0.0247	24.123	0.974
10	5.198	BB	6.5	5.3	0.0203	0.321	0.877
11	5.242	BB	7.9	6.1	0.0221	0.393	0.94
[illegible]	[illegible]	[illegible]	[illegible]	[illegible]	[illegible]	[illegible]	[illegible]

UPLC calibration curve and calculation of reaction assay yield

AUC (product)	AUC (standard)	AUC (pdt/std)	Yield
68.1	252.4	0.270	13%
135	253.9	0.532	28%
291.9	286.3	1.020	49%
Yield % = 48.506 × AUC(pdt/std), R^2 = 0.9996			
**Reaction assay yield based on UPLC traces at 220 nm absorption:**			
164.5	131	1.256	**61%**

2-chloro-5-((4-methyltetrahydro-2*H*-pyran-4-yl)methyl)pyridine (48):

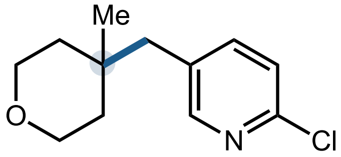

Prepared according to **General Procedure B** using 4-methyltetrahydro-2*H*-pyran-4-ol (116.2 mg, 1.0 mmol, 2.0 equiv.), **NHC** (563.1 mg, 1.10 mmol, 2.2 equiv.), pyridine (87 μL, 1.08 mmol, 2.15 equiv.), $PhCF_3$ (10.0 mL), 1-((6-chloropyridin-3-yl)methyl)-2,4,6-triphenylpyridin-1-ium tetrafluoroborate (260.4 mg, 0.5 mmol, 1.0 equiv.), KOPiv (280.4 mg, 2.0 mmol, 4.0 equiv.), $Ir[(dFCF_3ppy)_2(dtbbpy)]PF_6$ (8.4 mg, 7.5 μmol, 1.5 mol%), Fe(OEP)Cl (15.6 mg, 12.5 μmol, 2.0 mol%), acetone (5.0 mL), and *i*-PrOH (5.0 mL).

After irradiation, the reaction was concentrated under reduced pressure, and the resulting crude residue was purified by automated flash column chromatography (2 × 25 g silica column, 0-50% EtOAc/hexanes) followed by automated reverse-phase column chromatography (30 g C18 column, 25-60% MeCN/H_2O with 0.1% NH_4OH) to afford the title compound as an off-white solid (77.4 mg, 69% yield).

^{1}H NMR (500 MHz, $CDCl_3$) δ 8.15 (s, 1H), 7.41 (dd, *J* = 8.1, 2.5 Hz, 1H), 7.24 (d, *J* = 8.1 Hz, 1H), 3.80 – 3.72 (m, 2H), 3.59 (ddd, *J* = 12.0, 10.1, 2.8 Hz, 2H), 2.55 (s, 2H), 1.54 (ddd, *J* = 14.0, 10.0, 4.3 Hz, 2H), 1.30 – 1.22 (m, 2H), 0.94 (s, 3H).

^{13}C NMR (126 MHz, $CDCl_3$) δ 151.08, 149.48, 140.84, 132.38, 123.58, 63.85, 45.76, 37.33, 32.04, 22.84.

IR (film) ν_{max} 2923, 2849, 1583, 1563, 1457, 1224, 1106, 1018, 841, 817, 747, 541 cm^{-1}.

HRMS (ESI-TOF) *m/z* calculated for $C_{12}H_{17}ClNO^+$ ($[M+H]^+$) 226.0993, found 226.0993.

benzyl 3-((6-chloropyridin-3-yl)methyl)-3-methylpyrrolidine-1-carboxylate ((±)-49):

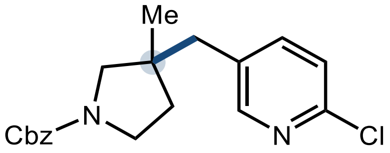

Prepared according to **General Procedure B** using benzyl 3-hydroxy-3-methylpyrrolidine-1-carboxylate (238.4 mg, 1.0 mmol, 2.0 equiv.), **NHC** (563.1 mg, 1.10 mmol, 2.2 equiv.), pyridine (87 μL, 1.08 mmol, 2.15 equiv.), $PhCF_3$ (10.0 mL), 2,4,6-triphenyl-1-((6-(trifluoromethyl)pyridin-3-yl)methyl)pyridin-1-ium tetrafluoroborate (277.15 mg, 0.5 mmol, 1.0 equiv.), KOPiv (280.4 mg, 2.0 mmol, 4.0 equiv.), $Ir[(dFCF_3ppy)_2(dtbbpy)]PF_6$ (8.4 mg, 7.5 μmol, 1.5 mol%), Fe(OEP)Cl (15.6 mg, 12.5 μmol, 2.5 mol%), acetone (5.0 mL), and *i*-PrOH (5.0 mL).

After irradiation, the reaction was concentrated under reduced pressure, and the resulting crude residue was purified by automated flash column chromatography (50 g silica column, 0-30-30-80% EtOAc/hexanes) followed by automated reverse-phase column chromatography (30 g C18 column, 10-55-65% $MeCN/H_2O$ with 0.1% NH_4OH) to afford the title compound as a colorless gum (104.1 mg, 60% yield).

^{1}H NMR (500 MHz, $CDCl_3$) δ 8.22 – 8.13 (m, 1H), 7.47 – 7.28 (m, 6H), 7.25 – 7.21 (m, 1H), 5.20 – 5.08 (m, 2H), 3.61 – 3.41 (m, 2H), 3.36 – 3.25 (m, 1H), 3.21 – 3.07 (m, 1H), 2.70 – 2.60 (m, 2H), 1.84 – 1.73 (m, 1H), 1.64 – 1.59 (m, 1H), 1.03 – 0.97 (m, 3H). **(summary of rotamers)**

^{13}C NMR (126 MHz, $CDCl_3$) δ 155.05, 155.00, 150.73, 150.71, 149.88, 140.22, 136.96, 132.69, 132.67, 128.59, 128.56, 128.11, 128.08, 128.05, 127.95, 123.86, 66.92, 66.88, 57.21, 56.69, 45.03, 44.64, 42.57, 41.69, 41.52, 41.33, 37.04, 36.42, 23.28, 23.18. **(summary of rotamers)**

IR (film) ν_{max} 2960, 2874, 1695, 1456, 1414, 1385, 1343, 1214, 1140, 1093, 1024, 910, 819, 768, 730, 696 cm^{-1}.

HRMS (ESI-TOF) *m/z* calculated for $C_{19}H_{22}ClN_2O_2^+$ ($[M+H]^+$) 345.1364, found 345.1366.

***tert*-butyl 4-((6-chloropyridin-3-yl)methyl)-4-methylazepane-1-carboxylate ((±)-50):**

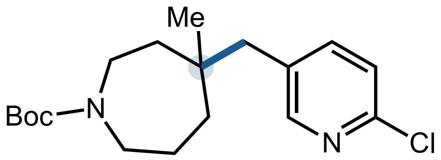

Prepared according to **General Procedure B** using *tert*-butyl 4-hydroxy-4-methylazepane-1-carboxylate (229.3 mg, 1.0 mmol, 2.0 equiv.), **NHC** (563.1 mg, 1.10 mmol, 2.2 equiv.), pyridine (87 μL, 1.08 mmol, 2.15 equiv.), $PhCF_3$ (10.0 mL), 1-((6-chloropyridin-3-yl)methyl)-2,4,6-triphenylpyridin-1-ium tetrafluoroborate (260.4 mg, 0.5 mmol, 1.0 equiv.), KOPiv (280.4 mg, 2.0 mmol, 4.0 equiv.), $Ir[(dFCF_3ppy)_2(dtbbpy)]PF_6$ (8.4 mg, 7.5 μmol, 1.5 mol%), Fe(OEP)Cl (15.6 mg, 12.5 μmol, 2.0 mol%), acetone (5.0 mL), and *i*-PrOH (5.0 mL).

After irradiation, the reaction was concentrated under reduced pressure, and the resulting crude residue was purified by automated flash column chromatography (3 × 10 g silica column, 5-35% EtOAc/hexanes) followed by automated reverse-phase column chromatography (30 g C18 column, 55-75% $MeCN/H_2O$ with 0.1% NH_4OH) to afford the title compound as a colourless oil (138.4 mg, 82% yield).

^{1}H NMR (500 MHz, $CDCl_3$) δ 8.13 (d, *J* = 2.5 Hz, 1H), 7.43 – 7.36 (m, 1H), 7.22 (d, *J* = 8.2 Hz, 1H), 3.62 – 3.07 (m, 4H), 2.55 – 2.45 (m, 2H), 1.83 – 1.49 (m, 3H), 1.47 – 1.27 (m, 12H), 0.85 (s, 3H).

^{13}C NMR (126 MHz, $CDCl_3$) δ 155.65, 155.60, 151.26, 149.47, 149.42, 140.87, 140.82, 132.97, 132.94, 123.47, 79.34, 46.13, 45.48, 45.39, 45.36, 42.02, 41.41, 39.67, 39.61, 38.59, 38.34, 36.69, 36.64, 28.61, 24.68, 24.46, 23.65, 23.29. **(summary of rotamers)**

IR (film) ν_{max} 2970, 2927, 1682, 1523, 1502, 1458, 1411, 1363, 1246, 1161, 1108, 1024, 816, 771, 745, 634 cm^{-1}.

HRMS (ESI-TOF) *m/z* calculated for $C_{18}H_{27}ClN_2NaO_2^+$ ($[M+Na]^+$) 361.1653, found 361.1654.

***tert*-butyl 7-((6-chloropyridin-3-yl)methyl)-7-methyl-2-azaspiro[3.5]nonane-2-carboxylate (51):**

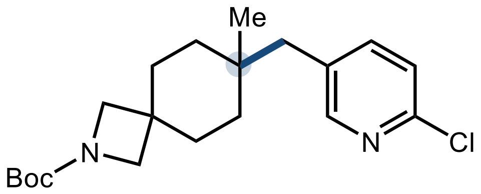

Prepared according to **General Procedure B** using *tert*-butyl 7-hydroxy-7-methyl-2-azaspiro[3.5]nonane-2-carboxylate (255.4 mg, 1.0 mmol, 2.0 equiv.),[12] **NHC** (563.1 mg, 1.10 mmol, 2.2 equiv.), pyridine (87 µL, 1.08 mmol, 2.15 equiv.), $PhCF_3$ (10.0 mL), 2,4,6-triphenyl-1-((6-(trifluoromethyl)pyridin-3-yl)methyl)pyridin-1-ium tetrafluoroborate (277.15 mg, 0.5 mmol, 1.0 equiv.), KOPiv (280.4 mg, 2.0 mmol, 4.0 equiv.), $Ir[(dFCF_3ppy)_2(dtbbpy)]PF_6$ (8.4 mg, 7.5 µmol, 1.5 mol%), Fe(OEP)Cl (15.6 mg, 12.5 µmol, 2.5 mol%), acetone (5.0 mL), and *i*-PrOH (5.0 mL).

After irradiation, the reaction was concentrated under reduced pressure, and the resulting crude residue was purified by automated flash column chromatography (50 g silica column, 0-10-20-40% EtOAc/hexanes) followed by automated reverse-phase column chromatography (30 g C18 column, 10-70% $MeCN/H_2O$ with 0.1% NH_4OH) to afford the title compound as an off-white solid (92.4 mg, 51% yield).

^{1}H NMR (500 MHz, $CDCl_3$) δ 8.14 (d, *J* = 2.5 Hz, 1H), 7.39 (dd, *J* = 8.1, 2.5 Hz, 1H), 7.23 (d, *J* = 8.1 Hz, 1H), 3.57 (s, 2H), 3.56 (s, 2H), 2.49 (s, 2H), 1.73 – 1.69 (m, 2H), 1.68 – 1.59 (m, 2H), 1.44 (s, 9H), 1.33 – 1.19 (m, 4H), 0.82 (s, 3H). **(summary of rotamers)**

[12] Gould, C. A.; Pace, A. L.; MacMillan, D. W. C. Rapid and Modular Access to Quaternary Carbons from Tertiary Alcohols via Bimolecular Homolytic Substitution. *J. Am. Chem. Soc.* **2023,** *145*, 16330–16336. DOI: 10.1021/jacs.3c05405.

^{13}C NMR (126 MHz, CDCl$_3$) δ 156.59, 151.03, 149.36, 140.61, 132.90, 123.42, 79.26, 60.19, 59.73, 59.00, 58.55, 44.68, 34.81, 33.75, 33.15, 31.63, 28.49, 23.32. **(summary of rotamers)**

IR (film) ν_{max} 2973, 2920, 2870, 1693, 1456, 1401, 1363, 1138, 1117, 1100, 1077, 918, 817, 771, 758, 747, 730 cm^{-1}.

HRMS (ESI-TOF) *m/z* calculated for $C_{20}H_{29}ClN_2NaO_2^+$ ([M+Na]$^+$) 387.1810, found 387.1813.

***tert*-butyl -6-((6-chloropyridin-3-yl)methyl)-6-methyl-2-azaspiro[3.4]octane-2-carboxylate ((±)-52):**

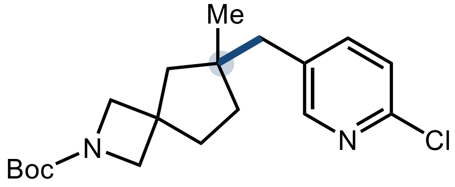

Prepared according to **General Procedure B** using *tert*-butyl 6-hydroxy-6-methyl-2-azaspiro[3.4]octane-2-carboxylate (241.3 mg, 1.0 mmol, 2.0 equiv.),[13] **NHC** (563.1 mg, 1.10 mmol, 2.2 equiv.), pyridine (87 µL, 1.08 mmol, 2.15 equiv.), $PhCF_3$ (10.0 mL), 2,4,6-triphenyl-1-((6-(trifluoromethyl)pyridin-3-yl)methyl)pyridin-1-ium tetrafluoroborate (277.15 mg, 0.5 mmol, 1.0 equiv.), KOPiv (280.4 mg, 2.0 mmol, 4.0 equiv.), $Ir[(dFCF_3ppy)_2(dtbbpy)]PF_6$ (8.4 mg, 7.5 µmol, 1.5 mol%), Fe(OEP)Cl (15.6 mg, 12.5 µmol, 2.5 mol%), acetone (5.0 mL), and *i*-PrOH (5.0 mL).

After irradiation, the reaction was concentrated under reduced pressure, and the resulting crude residue was purified by automated flash column chromatography (50 g silica column, 0-20-30-40-60% EtOAc/hexanes) followed by automated reverse-phase column chromatography (30 g C18 column, 10-70% $MeCN/H_2O$ with 0.1% NH_4OH) to afford the title compound as a light yellow gum (85.4 mg, 49% yield). The assay yield of the reaction was determined to be 62% via UPLC analysis of the crude reaction mixture with 1,3,5-trimethoxybenzene (21 mg, 0.25 mmol, 0.5 equiv.) as the internal standard (see below).

^{1}H NMR (500 MHz, $CDCl_3$) δ 8.17 (d, *J* = 2.5 Hz, 1H), 7.43 (dd, *J* = 8.2, 2.5 Hz, 1H), 7.25 (d, *J* = 8.1 Hz, 1H), 3.92 – 3.68 (m, 4H), 2.57 (s, 2H), 2.03 – 1.88 (m, 2H), 1.77 (d, *J* = 13.3 Hz, 1H),

[13] Ibid.

1.66 (d, J = 13.3 Hz, 1H), 1.62 – 1.51 (m, 1H *overlapped with H_2O peak*), 1.43 (s, 9H), 1.42 – 1.33 (m, 1H). **(summary of rotamers)**

^{13}C NMR (126 MHz, $CDCl_3$) δ 156.36, 150.82, 149.50, 140.30, 133.73, 123.63, 79.38, 63.52, 62.36, 61.57, 51.39, 44.68, 43.64, 40.67, 38.37, 37.99, 28.51, 26.21. **(summary of rotamers)**

IR (film) ν_{max} 2933, 2868, 1695, 1457, 1389, 1365, 1252, 1134, 1101, 1024, 845, 818, 772, 746 cm^{-1}.

HRMS (ESI-TOF) *m/z* calculated for $C_{19}H_{28}ClN_2O_2^+$ ([M+Na]$^+$) 373.1653, found 373.1654.

UPLC traces at 220 nm absorption of the crude reaction mixture

[Product (t_R = 4.35 min), 1,3,5-Trimethoxybenzene (t_R = 3.01 min)]

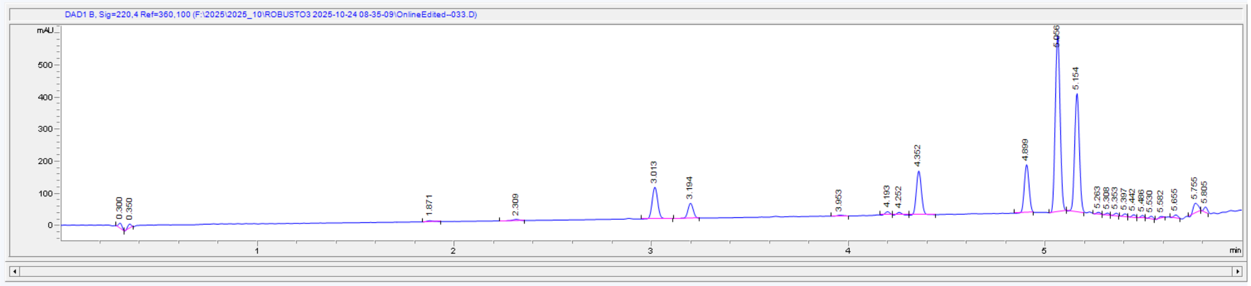

#	Time	Type	Area	Height	Width	Area%	Symmetry
3	1.871	BB	8	3.3	0.0365	0.309	0.619
4	2.309	BB	9.6	3.8	0.0386	0.367	1.031
5	3.013	BB	192.1	98.2	0.0308	7.384	0.915
6	3.194	BB	86.4	46.4	0.0297	3.323	0.959
7	3.953	BB	6.1	2.6	0.0343	0.234	0.739
8	4.193	BB	17.4	10.2	0.0279	0.670	0.967
9	4.252	BB	14.1	8.1	0.0273	0.544	0.825
10	4.352	BB	244.9	136.6	0.0279	9.413	0.895
11	4.899	BB	249.5	149.6	0.0265	9.591	0.937
12	5.056	BB	945.5	551.1	0.027	36.346	0.926
13	5.154	BB	611.2	368.8	0.0263	23.495	0.931
[illegible]	[illegible]	[illegible]	[illegible]	[illegible]	[illegible]	[illegible]	[illegible]

UPLC calibration curve and calculation of reaction assay yield

AUC (product)	AUC (standard)	AUC (pdt/std)	Yield
96	336.6	0.285	14%
173.6	328.6	0.528	26%
233.3	232.2	1.004	49%
Yield % = 48.966 × AUC(pdt/std), R^2 = 0.9989			
**Reaction assay yield based on UPLC traces at 220 nm absorption:**			
244.9	192.1	1.275	**62%**

***tert*-butyl 1-((6-chloropyridin-3-yl)methyl)-1-methyl-8-azaspiro[4.5]decane-8-carboxylate ((±)-53):**

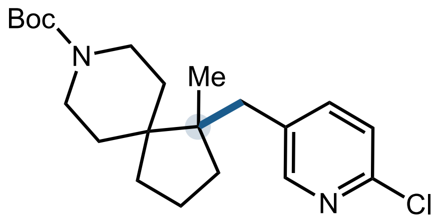

Prepared according to **General Procedure B** using *tert*-butyl 1-hydroxy-1-methyl-8-azaspiro[4.5]decane-8-carboxylate (269.4 mg, 1.0 mmol, 2.0 equiv.), **NHC** (563.1 mg, 1.10 mmol, 2.2 equiv.), pyridine (87 μL, 1.08 mmol, 2.15 equiv.), $PhCF_3$ (10.0 mL), 1-((6-chloropyridin-3-yl)methyl)-2,4,6-triphenylpyridin-1-ium tetrafluoroborate (260.4 mg, 0.5 mmol, 1.0 equiv.), KOPiv (280.4 mg, 2.0 mmol, 4.0 equiv.), $Ir[(dFCF_3ppy)_2(dtbbpy)]PF_6$ (8.4 mg, 7.5 μmol, 1.5 mol%), Fe(OEP)Cl (15.6 mg, 12.5 μmol, 2.0 mol%), acetone (5.0 mL), and *i*-PrOH (5.0 mL).

After irradiation, the reaction was concentrated under reduced pressure, and the resulting crude residue was purified by automated flash column chromatography (3 × 10 g silica column, 0-40% EtOAc/hexanes) followed by automated reverse-phase column chromatography (30 g C18 column, 60-85% $MeCN/H_2O$ with 0.1% NH_4OH) to afford the title compound as a light-yellow oil (93.8 mg, 50% yield).

^{1}H NMR (500 MHz, $CDCl_3$) δ 8.13 (s, 1H), 7.40 (dd, *J* = 8.1, 2.5 Hz, 1H), 7.21 (d, *J* = 8.1 Hz, 1H), 4.05 (s, 2H), 2.94 – 2.62 (m, 2H), 2.55 (d, *J* = 13.0 Hz, 1H), 2.38 (d, *J* = 13.0 Hz, 1H), 1.91 – 1.82 (m, 1H), 1.77 – 1.40 (m, 15H), 1.39 – 1.19 (m, 3H), 0.66 (s, 3H).

^{13}C NMR (126 MHz, $CDCl_3$) δ 155.01, 151.01, 149.22, 140.60, 134.61, 123.49, 79.48, 47.52, 46.11, 41.21 (2C), 38.15, 35.29, 31.58, 30.63, 30.44, 28.60, 20.00, 19.57.

IR (film) ν_{max} 2953, 2874, 1687, 1582, 1564, 1458, 1424, 1386, 1243, 1159, 1111, 1024, 866, 731 cm^{-1}.

HRMS (ESI-TOF) *m/z* calculated for $C_{17}H_{24}ClN_2O_2^+$ ([M+H–isobutylene]$^+$) 323.1521, found 323.1521.

***tert*-butyl 2-((6-chloropyridin-3-yl)methyl)-2-methyl-7-azaspiro[3.5]nonane-7-carboxylate (54):**

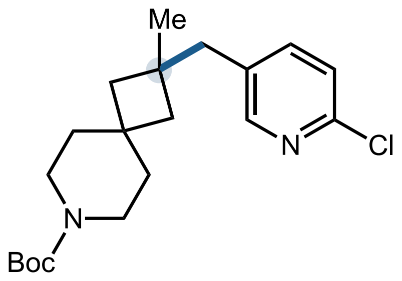

Prepared according to **General Procedure B** using *tert*-butyl 2-hydroxy-2-methyl-7-azaspiro[3.5]nonane-7-carboxylate (255.4 mg, 1.0 mmol, 2.0 equiv.),[14] **NHC** (563.1 mg, 1.10 mmol, 2.2 equiv.), pyridine (87 μL, 1.08 mmol, 2.15 equiv.), $PhCF_3$ (10.0 mL), 1-((6-chloropyridin-3-yl)methyl)-2,4,6-triphenylpyridin-1-ium tetrafluoroborate (260.4 mg, 0.5 mmol, 1.0 equiv.), KOPiv (280.4 mg, 2.0 mmol, 4.0 equiv.), $Ir[(dFCF_3ppy)_2(dtbbpy)]PF_6$ (8.4 mg, 7.5 μmol, 1.5 mol%), Fe(OEP)Cl (15.6 mg, 12.5 μmol, 2.0 mol%), acetone (5.0 mL), and *i*-PrOH (5.0 mL).

After irradiation, the reaction was concentrated under reduced pressure, and the resulting crude residue was purified by automated flash column chromatography (3 × 10 g silica column, 10-65% Et_2O/hexanes) followed by automated reverse-phase column chromatography (30 g C18 column, 60-85% $MeCN/H_2O$ with 0.1% NH_4OH) to afford the title compound as a light-yellow oil (90.4 mg, 50% yield).

^{1}H NMR (500 MHz, Acetone-*d*$_6$) δ 8.22 (d, J = 2.5 Hz, 1H), 7.66 (dd, J = 8.2, 2.5 Hz, 1H), 7.33 (d, J = 8.1 Hz, 1H), 3.28 – 3.21 (m, 4H), 2.73 (s, 2H), 1.88 (d, J = 12.6 Hz, 2H), 1.59 (d, J = 12.9 Hz, 2H), 1.53 – 1.48 (m, 2H), 1.47 – 1.40 (m, 2H), 1.38 (s, 9H), 1.06 (s, 3H).

---

[14] Aguilar Troyano, F.J.; Ballaschk, F.; Jaschinski, M.; Özkaya, Y.; Gómez-Suárez, A. *Chem. Eur. J.* **2019**, *25*, 14054.

^{13}C NMR (126 MHz, Acetone-d_6) δ 155.29, 151.63, 149.88, 141.46, 135.26, 124.46, 79.28, 46.42, 43.76, 41.73, 40.90, 40.13, 39.97, 32.73, 30.64, 29.31, 28.73.

IR (film) v_{max} 2917, 2845, 1688, 1584, 1513, 1457, 1422, 1365, 1241, 1156, 1103, 1023, 974, 815 cm^{-1}.

HRMS (ESI-TOF) *m/z* calculated for $C_{20}H_{29}ClNaN_2O_2^+$ ([M+Na]$^+$) 401.1966, found 401.1966.

***tert*-butyl (3a*R*,5s,6a*S*)-5-((6-chloropyridin-3-yl)methyl)-5-methylhexahydrocyclopenta[*c*]-pyrrole-2(1*H*)-carboxylate (55):**

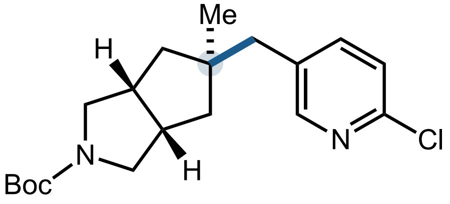

Prepared according to **General Procedure B** using *tert*-butyl (3a*R*,6a*S*)-5-hydroxy-5-methylhexahydrocyclopenta[*c*]pyrrole-2(1*H*)-carboxylate (241.3 mg, 1.0 mmol, 2.0 equiv.), **NHC** (563.1 mg, 1.10 mmol, 2.2 equiv.), pyridine (87 μL, 1.08 mmol, 2.15 equiv.), $PhCF_3$ (10.0 mL), 1-((6-chloropyridin-3-yl)methyl)-2,4,6-triphenylpyridin-1-ium tetrafluoroborate (260.4 mg, 0.5 mmol, 1.0 equiv.), KOPiv (280.4 mg, 2.0 mmol, 4.0 equiv.), $Ir[(dFCF_3ppy)_2(dtbbpy)]PF_6$ (8.4 mg, 7.5 μmol, 1.5 mol%), Fe(OEP)Cl (15.6 mg, 12.5 μmol, 2.0 mol%), acetone (5.0 mL), and *i*-PrOH (5.0 mL).

After irradiation, the reaction was concentrated under reduced pressure, and the resulting crude residue was purified by automated flash column chromatography (3 × 10 g silica column, 0-40% EtOAc/hexanes) followed by automated reverse-phase column chromatography (30 g C18 column, 60-80% $MeCN/H_2O$ with 0.1% NH_4OH) to afford the title compound as a light-yellow oil (102.9 mg, 59% yield, >20:1 d.r.).

^{1}H NMR (500 MHz, $CDCl_3$) δ 8.13 (s, 1H), 7.39 (dd, *J* = 8.1, 2.5 Hz, 1H), 7.23 (d, *J* = 8.1 Hz, 1H), 3.52 – 3.02 (m, 4H), 2.75 (td, *J* = 9.3, 5.4 Hz, 2H), 2.49 (s, 2H), 1.82 (dd, *J* = 14.3, 6.5 Hz, 2H), 1.43 (s, 9H), 1.21 (s, 2H), 0.98 (s, 3H).

^{13}C NMR (126 MHz, $CDCl_3$) δ 154.96, 150.83, 149.47, 140.39, 133.74, 123.62, 79.28, 52.18, 51.98, 45.98, 45.19, 42.74, 42.11, 41.83, 28.61, 26.54.

IR (film) ν_{max} 2931, 2866, 1687, 1583, 1513, 1455, 1386, 1239, 1169, 1130, 1103, 1023, 878, 773, 733 cm^{-1}.

HRMS (ESI-TOF) *m/z* calculated for $C_{15}H_{20}ClN_2O_2^+$ ($[M+H–isobutylene]^+$) 295.1207, found 295.1207.

***tert*-butyl (3-(6-chloropyridin-3-yl)-2,2-dimethylpropyl)carbamate (56):**

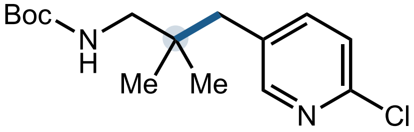

Prepared according to **General Procedure B** using *tert*-butyl (2-hydroxy-2-methylpropyl)carbamate (189.3 mg, 1.0 mmol, 2.0 equiv.), **NHC** (563.1 mg, 1.10 mmol, 2.2 equiv.), pyridine (87 μL, 1.08 mmol, 2.15 equiv.), $PhCF_3$ (10.0 mL), 1-((6-chloropyridin-3-yl)methyl)-2,4,6-triphenylpyridin-1-ium tetrafluoroborate (260.4 mg, 0.5 mmol, 1.0 equiv.), KOPiv (280.4 mg, 2.0 mmol, 4.0 equiv.), $Ir[(dFCF_3ppy)_2(dtbbpy)]PF_6$ (8.4 mg, 7.5 μmol, 1.5 mol%), Fe(OEP)Cl (15.6 mg, 12.5 μmol, 2.0 mol%), acetone (5.0 mL), and *i*-PrOH (5.0 mL).

After irradiation, the reaction was concentrated under reduced pressure, and the resulting crude residue was purified by automated flash column chromatography (2 × 25 g silica column, 0-40% EtOAc/hexanes) followed by automated reverse-phase column chromatography (30 g C18 column, 45-75% $MeCN/H_2O$ with 0.1% NH_4OH) to afford the title compound as a colourless oil (100.2 mg, 67% yield).

^{1}H NMR (500 MHz, CDCl$_3$) δ 8.14 (s, 1H), 7.44 (d, *J* = 6.8 Hz, 0H), 7.23 (d, *J* = 8.2 Hz, 1H), 4.65 (s, 1H), 2.98 (d, *J* = 6.3 Hz, 2H), 2.48 (s, 2H), 1.43 (s, 9H), 0.82 (s, 6H).

^{13}C NMR (126 MHz, CDCl$_3$) δ 156.30, 150.99, 149.42, 140.80, 132.91, 123.65, 79.49, 50.74, 42.07, 35.86, 28.49, 24.41.

IR (film) ν_{max} 3340, 2957, 2930, 2160, 2074, 1699, 1563, 1516, 1457, 1386, 1365, 1248, 1169, 1102, 1024, 847, 772, 635 cm^{-1}.

HRMS (ESI-TOF) *m/z* calculated for $C_{11}H_{16}ClN_2O_2^+$ ([M+H–isobutylene]$^+$) 243.0895, found 243.0894.

***tert*-butyl 3-(1-(6-chloropyridin-3-yl)-2-methylpropan-2-yl)azetidine-1-carboxylate (57):**

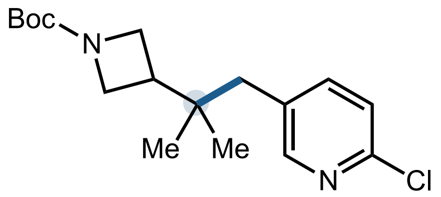

Prepared according to **General Procedure B** using *tert*-butyl 3-(2-hydroxypropan-2-yl)azetidine-1-carboxylate (215.3 mg, 1.0 mmol, 2.0 equiv.), **NHC** (563.1 mg, 1.10 mmol, 2.2 equiv.), pyridine (87 µL, 1.08 mmol, 2.15 equiv.), $PhCF_3$ (10.0 mL), 1-((6-chloropyridin-3-yl)methyl)-2,4,6-triphenylpyridin-1-ium tetrafluoroborate (260.4 mg, 0.5 mmol, 1.0 equiv.), KOPiv (280.4 mg, 2.0 mmol, 4.0 equiv.), $Ir[(dFCF_3ppy)_2(dtbbpy)]PF_6$ (8.4 mg, 7.5 µmol, 1.5 mol%), Fe(OEP)Cl (15.6 mg, 12.5 µmol, 2.0 mol%), acetone (5.0 mL), and *i*-PrOH (5.0 mL).

After irradiation, the reaction was concentrated under reduced pressure, and the resulting crude residue was purified by automated flash column chromatography (3 × 10 g silica column, 0-40% EtOAc/hexanes) followed by automated reverse-phase column chromatography (30 g C18 column, 55-75% $MeCN/H_2O$ with 0.1% NH_4OH) to afford the title compound as a light-yellow oil (103.6 mg, 64% yield).

^{1}H NMR (500 MHz, $CDCl_3$) δ 8.09 (s, 1H), 7.35 (dd, J = 8.1, 2.4 Hz, 1H), 7.23 (d, J = 8.1 Hz, 1H), 3.82 (t, J = 8.8 Hz, 2H), 3.67 (dd, J = 8.9, 6.0 Hz, 2H), 2.43 – 2.33 (m, 3H), 1.42 (s, 9H), 0.86 (s, 6H).

^{13}C NMR (126 MHz, $CDCl_3$) δ 156.31, 150.95, 149.70, 140.50, 132.53, 123.65, 79.51, 50.30, 49.41, 42.69, 37.72, 34.84, 28.51, 22.52.

IR (film) ν_{max} 2694, 2886, 1694, 1584, 1564, 1458, 1389, 1254, 1137, 1024, 860, 773, 745, 634 cm^{-1}.

HRMS (ESI-TOF) *m/z* calculated for $C_{13}H_{18}ClN_2O_2^+$ ([M+H–isobutylene]$^+$) 269.1051, found 269.1050.

***N*-(4-(6-chloropyridin-3-yl)-3,3-dimethylbutyl)-5-methyl-3-phenylisoxazole-4-carboxamide (58):**

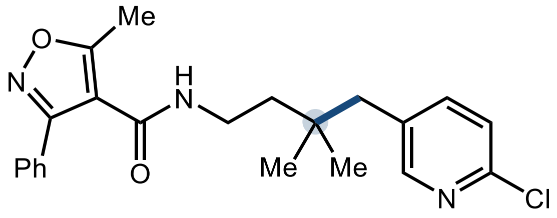

*Prepared according to **General Procedure B** with the following modifications: A 1:1 mixture of **$PhCF_3$ / MTBE** was used as the optimal alcohol activation solvent.*

Prepared according to **General Procedure B** using *N*-(3-hydroxy-3-methylbutyl)-5-methyl-3-phenylisoxazole-4-carboxamide (144.2 mg, 0.5 mmol, 2.0 equiv.), **NHC** (281.5 mg, 0.55 mmol, 2.2 equiv.), pyridine (43 µL, 0.538 mmol, 2.15 equiv.), $PhCF_3$ (2.5 mL), *t*-BuOMe (2.5 mL), 2,4,6-triphenyl-1-((6-(trifluoromethyl)pyridin-3-yl)methyl)pyridin-1-ium tetrafluoroborate (134.2 mg, 0.25 mmol, 1.0 equiv.), KOPiv (140.2 mg, 1.0 mmol, 4.0 equiv.), $Ir[(dFCF_3ppy)_2(dtbbpy)]PF_6$ (4.2 mg, 3.75 µmol, 1.5 mol%), Fe(OEP)Cl (3.9 mg, 6.25 µmol, 2.5 mol%), acetone (2.5 mL), and *i*-PrOH (2.5 mL).

After irradiation, the reaction was concentrated under reduced pressure, and the resulting crude residue was purified by automated flash column chromatography (25 g silica column, 0-20-30-40-60% EtOAc/hexanes) followed by automated reverse-phase column chromatography (30 g C18 column, 10-60% $MeCN/H_2O$ with 0.1% NH_4OH) to afford the title compound as an off-white solid (46.6 mg, 72% yield).

^{1}H NMR (500 MHz, $CDCl_3$) δ 8.10 (d, *J* = 2.5 Hz, 1H), 7.59 – 7.53 (m, 3H), 7.52 – 7.46 (m, 2H), 7.40 (dd, *J* = 8.1, 2.5 Hz, 1H), 7.24 (d, *J* = 8.1 Hz, 1H), 5.27 (br-s, 1H), 3.34 – 3.26 (m, 2H), 2.73 (s, 3H), 2.45 (s, 2H), 1.22 – 1.17 (m, 2H), 0.83 (s, 6H).

^{13}C NMR (126 MHz, $CDCl_3$) δ 174.01, 161.50, 160.09, 151.01, 149.49, 140.63, 132.85, 130.62, 129.18, 129.07, 128.28, 123.54, 111.15, 44.79, 40.67, 35.57, 33.62, 26.40, 12.99.

IR (film) ν_{max} 3278, 3065, 2959, 2870, 1644, 1563, 1535, 1458, 1415, 1384, 1367, 1173, 1102, 1024, 908, 836, 821, 773, 728, 696, 646 cm^{-1}.

HRMS (ESI-TOF) *m/z* calculated for $C_{22}H_{25}ClN_3O_2^+$ ($[M+H]^+$) 398.1630, found 398.1632.

2-chloro-5-(2,2-dimethyl-3-(4-(4,4,5,5-tetramethyl-1,3,2-dioxaborolan-2-yl)-1*H*-pyrazol-1-yl)propyl)pyridine (59):

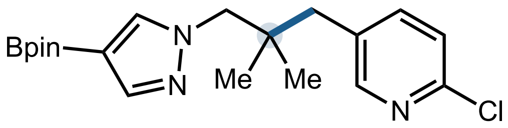

Prepared according to **General Procedure B** using 2-methyl-1-(4-(4,4,5,5-tetramethyl-1,3,2-dioxaborolan-2-yl)-1*H*-pyrazol-1-yl)propan-2-ol (266.1 mg, 1.0 mmol, 2.0 equiv.), **NHC** (563.1 mg, 1.10 mmol, 2.2 equiv.), pyridine (87 μL, 1.08 mmol, 2.15 equiv.), $PhCF_3$ (10.0 mL), 1-((6-chloropyridin-3-yl)methyl)-2,4,6-triphenylpyridin-1-ium tetrafluoroborate (260.4 mg, 0.5 mmol, 1.0 equiv.), KOPiv (280.4 mg, 2.0 mmol, 4.0 equiv.), $Ir[(dFCF_3ppy)_2(dtbbpy)]PF_6$ (8.4 mg, 7.5 μmol, 1.5 mol%), Fe(OEP)Cl (15.6 mg, 12.5 μmol, 2.0 mol%), acetone (5.0 mL), and *i*-PrOH (5.0 mL).

After irradiation, the reaction was concentrated under reduced pressure, and the resulting crude residue was purified by automated flash column chromatography (3 × 10 g silica column, 5-40% EtOAc/hexanes) followed by automated reverse-phase column chromatography (30 g C18 column, 50-75% $MeCN/H_2O$ with 0.1% formic acid) to afford the title compound as a light-yellow oil (75.1 mg, 40% yield). The assay yield of the reaction was determined to be 57% via UPLC analysis of the crude reaction mixture with mesitylene (34.8 μL, 0.25 mmol, 0.5 equiv.) as the internal standard (see below).

^{1}H NMR (500 MHz, $CDCl_3$) δ 8.23 (d, J = 2.4 Hz, 1H), 7.82 (s, 1H), 7.67 (s, 1H), 7.62 (dd, J = 8.2, 2.5 Hz, 1H), 7.28 (d, J = 2.2 Hz, 1H), 3.97 (s, 2H), 2.60 (s, 2H), 1.34 (s, 12H), 0.90 (s, 6H).

^{13}C NMR (126 MHz, $CDCl_3$) δ 151.40, 149.70, 145.42, 141.06, 137.75, 132.62, 123.64, 83.47, 61.93, 42.06, 36.51, 25.09, 24.94.

^{11}B NMR (128 MHz, $CDCl_3$) δ 30.75.

IR (film) ν_{max} 2973, 2935, 1729, 1556, 1458, 1382, 1292, 1254, 1143, 1109, 986, 806, 693 cm^{-1}.

HRMS (ESI-TOF) *m/z* calculated for $C_{19}H_{28}BClN_3O_2^+$ ($[M+H]^+$) 376.1958, found 376.1958.

UPLC traces at 220 nm absorption of the crude reaction mixture

[Product (t_R = 4.001 min), Mesitylene (t_R = 4.164 min)]

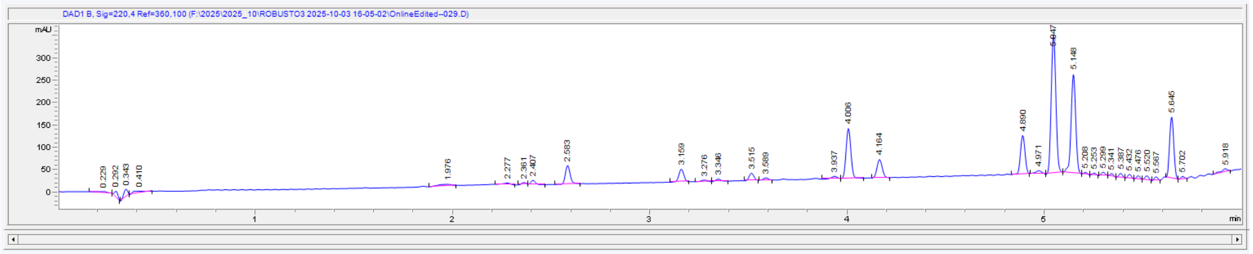

#	Time	Type	Area	Height	Width	Area%	Symmetry
10	3.159	BB	50.4	28.5	0.0276	2.631	1.004
11	3.276	BB	6.7	3.3	0.0316	0.352	1.107
12	3.346	BB	9	5.1	0.0276	0.468	0.882
13	3.515	BB	27.1	16.3	0.0263	1.413	0.946
14	3.589	BB	8.8	5.1	0.0273	0.459	1.068
15	3.937	BB	10.6	5.3	0.0324	0.555	1.322
16	4.006	BB	190.6	112.2	0.0268	9.952	0.905
17	4.164	BB	76.8	41.4	0.0296	4.007	0.907
18	4.89	BB	137	86.7	0.0254	7.152	0.961
19	4.971	BB	16.4	7.2	0.0364	0.854	0.968

UPLC calibration curve and calculation of reaction assay yield

AUC (product)	AUC (standard)	AUC (pdt/std)	Yield
40.9	56	0.730	13%
105.2	78.5	1.340	28%
94.2	49.6	1.899	47%
Yield % = 22.957 × AUC(pdt/std), R^2 = 0.9425			
**Reaction assay yield based on UPLC traces at 220 nm absorption:**			
190.6	76.8	2.482	**57%**

2-chloro-5-(((1*R*,2*R*,4*R*)-1-methyl-4-(prop-1-en-2-yl)-2-(1*H*-1,2,4-triazol-1-yl)cyclohexyl)-methyl)pyridine (60):

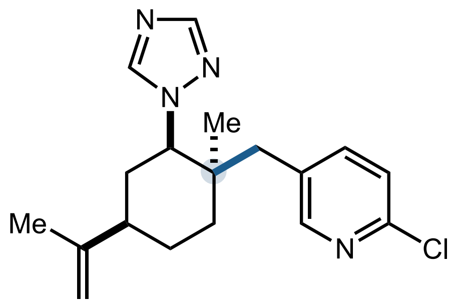

Prepared according to **General Procedure B** using (4*R*)-1-methyl-4-(prop-1-en-2-yl)-2-(1*H*-1,2,4-triazol-1-yl)cyclohexan-1-ol (221.3 mg, 1.0 mmol, 2.0 equiv.), **NHC** (563.1 mg, 1.10 mmol, 2.2 equiv.), pyridine (87 μL, 1.08 mmol, 2.15 equiv.), $PhCF_3$ (10.0 mL), 1-((6-chloropyridin-3-yl)methyl)-2,4,6-triphenylpyridin-1-ium tetrafluoroborate (260.4 mg, 0.5 mmol, 1.0 equiv.), KOPiv (280.4 mg, 2.0 mmol, 4.0 equiv.), $Ir[(dFCF_3ppy)_2(dtbbpy)]PF_6$ (8.4 mg, 7.5 μmol, 1.5 mol%), Fe(OEP)Cl (15.6 mg, 12.5 μmol, 2.0 mol%), acetone (5.0 mL), and *i*-PrOH (5.0 mL).

After irradiation, the reaction was concentrated under reduced pressure, and the resulting crude residue was purified by automated flash column chromatography (3 × 10 g silica column, 5-75% EtOAc/hexanes) followed by automated reverse-phase column chromatography (30 g C18 column, 45-75% $MeCN/H_2O$ with 0.1% NH_4OH) to afford the title compound as an off-white solid (78.1 mg, 47% yield, >20:1 d.r.).

^{1}H NMR (500 MHz, $CDCl_3$) δ 8.14 (d, *J* = 2.5 Hz, 1H), 8.05 (s, 1H), 7.94 (s, 1H), 7.44 (dd, *J* = 8.2, 2.5 Hz, 1H), 7.24 (d, *J* = 8.1 Hz, 1H), 4.78 (s, 1H), 4.31 (t, *J* = 5.9 Hz, 1H), 2.85 – 2.76 (m, 1H), 2.72 (d, *J* = 13.5 Hz, 1H), 2.54 (d, *J* = 13.5 Hz, 1H), 2.18 – 2.11 (m, 2H), 1.88 – 1.56 (m, 6H), 1.46 – 1.36 (m, 1H), 0.66 (s, 3H).

^{13}C NMR (126 MHz, Acetone-d_6) δ 152.35, 152.15, 150.12, 145.61, 142.34, 133.73, 124.42, 110.12, 62.76, 40.93, 39.47, 38.72, 32.70, 32.55, 26.30, 22.23, 21.67.

IR (film) ν_{max} 3084, 2957, 2862, 1642, 1584, 1563, 1502, 1459, 1385, 1274, 1203, 1142, 1107, 1017, 957, 896, 735, 672 cm^{-1}.

HRMS (ESI-TOF) *m/z* calculated for $C_{18}H_{24}ClN_4^+$ ($[M+H]^+$) 331.1684, found 331.1683.

tert-butyl 6-((1-((benzyloxy)carbonyl)-4-((6-chloropyridin-3-yl)methyl)piperidin-4-yl)methoxy)-2-azaspiro[3.3]heptane-2-carboxylate (61):

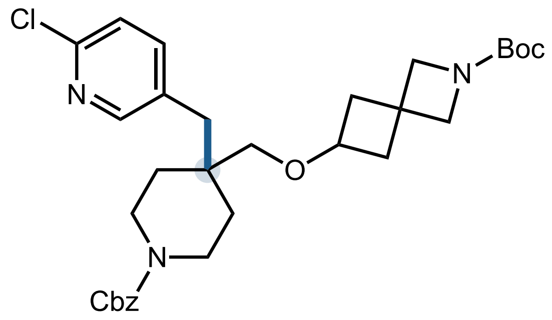

Prepared according to **General Procedure B** using *tert*-butyl 6-((1-((benzyloxy)carbonyl)-4-hydroxypiperidin-4-yl)methoxy)-2-azaspiro[3.3]heptane-2-carboxylate (460.6 mg, 1.0 mmol, 2.0 equiv.), **NHC** (563.1 mg, 1.10 mmol, 2.2 equiv.), pyridine (87 μL, 1.08 mmol, 2.15 equiv.), $PhCF_3$ (10.0 mL), 1-((6-chloropyridin-3-yl)methyl)-2,4,6-triphenylpyridin-1-ium tetrafluoroborate (260.4 mg, 0.5 mmol, 1.0 equiv.), KOPiv (280.4 mg, 2.0 mmol, 4.0 equiv.), $Ir[(dFCF_3ppy)_2(dtbbpy)]PF_6$ (8.4 mg, 7.5 μmol, 1.5 mol%), Fe(OEP)Cl (15.6 mg, 12.5 μmol, 2.0 mol%), acetone (5.0 mL), and *i*-PrOH (5.0 mL).

After irradiation, the reaction was concentrated under reduced pressure, and the resulting crude residue was purified by automated flash column chromatography (3 × 10 g silica column, 5-50% EtOAc/hexanes) followed by automated reverse-phase column chromatography (30 g C18 column, 65-85% $MeCN/H_2O$ with 0.1% NH_4OH) to afford the title compound as a light-yellow oil (141.6 mg, 50% yield).

^{1}H NMR (500 MHz, $CDCl_3$) δ 8.15 (d, J = 2.5 Hz, 1H), 7.43 – 7.06 (m, 7H), 5.11 (s, 2H), 3.87 (d, J = 2.2 Hz, 4H), 3.74 (p, J = 6.9 Hz, 1H), 3.68 – 3.60 (m, 2H), 3.32 (ddd, J = 13.3, 8.5, 4.1 Hz, 2H) [3.20 (ddd, J = 13.3, 8.5, 4.1 Hz, 2H)], 2.93 (s, 2H) [3.11 (s, 2H)], 2.64 (s, 2H) [2.59 (s, 2H)], 2.44 (ddd, J = 9.8, 6.7, 3.1 Hz, 2H) [2.58 (ddd, J = 9.8, 6.7, 3.1 Hz, 2H)], 2.08 – 2.00 (m, 2H) [2.25 – 2.13 (m, 2H)], 1.42 (s, 13H). **(minor rotamer in brackets)**

^{13}C NMR (126 MHz, $CDCl_3$) δ 156.22, 155.39, 154.69, 151.20, 149.54, 140.77, 138.03, 136.83, 132.12, 128.59, 128.12, 127.99, 127.94, 127.75, 123.71, 123.67, 79.48, 73.24, 71.65, 69.64, 68.82, 67.21, 64.86, 61.71, 60.53, 41.19, 41.00, 39.79, 38.33, 38.14, 36.78, 36.37, 31.98, 30.91, 30.39, 28.48. **(summary of rotamers)**

IR (film) ν_{max} 2970, 2870, 2246, 1694, 1584, 1563, 1392, 1278, 1242, 1171, 1078, 1025, 914, 732 cm^{-1}.

HRMS (ESI-TOF) *m/z* calculated for $C_{31}H_{40}ClN_3NaO_5^+$ ($[M+Na]^+$) 592.2549, found 592.2548.

***tert*-butyl 4-((2-(2,6-dioxopiperidin-3-yl)-1-oxoisoindolin-5-yl)methyl)-4-methylpiperidine-1-carboxylate (62):**

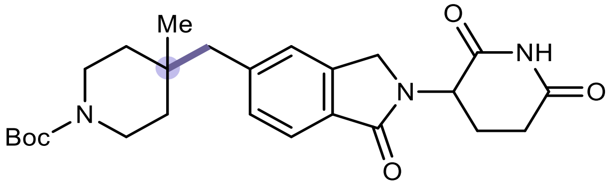

Prepared according to **General Procedure A** using 1-(*tert*-butoxycarbonyl)-4-methylpiperidine-4-carboxylic acid (25.1 mg, 0.1 mmol, 1.0 equiv.), 1-((2-(2,6-dioxopiperidin-3-yl)-1-oxoisoindolin-5-yl)methyl)-2,4,6-triphenylpyridin-1-ium tetrafluoroborate (97.7 mg, 0.15 mmol, 1.5 equiv.), K_2CO_3 (41.5 mg, 0.3 mmol, 3 equiv.), 4CzIPN (3.9 mg, 5.0 μmol, 5.0 mol%), Fe(OEP)Cl (3.1 mg, 5.0 μmol, 5.0 mol%), DMA (2 mL), and *i*-PrOH (2 mL)*.

**Note: Two-fold dilution was needed to prevent precipitation of dibenzyl byproducts.*

After work-up according to **General Procedure A**, the crude residue was purified by automated flash column chromatography (25 g silica column, 0-90% EtOAc/hexanes) followed by trituration with 33% Et_2O/hexanes and filtered of the solid to afford the title compound as an off-white solid (31.2 mg, 68% yield).

^{1}H NMR (500 MHz, DMSO-d$_6$) δ 10.98 (s, 1H), 7.63 (d, *J* = 7.7 Hz, 1H), 7.38 (s, 1H), 7.29 (dd, *J* = 7.7, 1.3 Hz, 1H), 5.10 (dd, *J* = 13.3, 5.2 Hz, 1H), 4.43 (d, *J* = 17.2 Hz, 1H), 4.30 (d, *J* = 17.1 Hz, 1H), 3.59 (dt, *J* = 13.6, 4.8 Hz, 2H), 3.21 – 3.01 (m, 2H), 2.91 (ddd, *J* = 17.3, 13.6, 5.4 Hz, 1H), 2.68 (s, 2H), 2.60 (ddd, *J* = 17.4, 4.5, 2.2 Hz, 1H), 2.39 (qd, *J* = 13.2, 4.5 Hz, 1H), 1.99 (dtd, *J* = 12.7, 5.3, 2.3 Hz, 1H), 1.45 – 1.32 (m, 2H), 1.38 (s, 9H), 1.28 – 1.16 (m, 2H), 0.87 (s, 3H).

^{13}C NMR (126 MHz, DMSO-d$_6$) δ 172.88, 171.10, 168.02, 153.92, 142.28, 141.81, 130.51, 129.65, 125.39, 122.18, 78.41, 51.51, 47.31, 47.01, 39.73 (2C *overlapped with solvent peak*), 36.06, 32.57, 31.22, 28.10, 22.81, 22.51.

IR (film) ν_{max} 3186, 3087, 2967, 2917, 2863, 1682, 1422, 1376, 1365, 1352, 1335, 1246, 1232, 1204, 1175, 1156, 1127, 1090, 992, 856, 839, 775, 757 cm^{-1}.

HRMS (ESI-TOF) *m/z* calculated for $C_{25}H_{33}N_3NaO_5^+$ ($[M+Na]^+$) 478.2312, found 478.2310.

4-chloro-*N*-(4-((1-(6-chloropyridin-3-yl)-2-methylpropan-2-yl)oxy)phenethyl)benzamide (63):

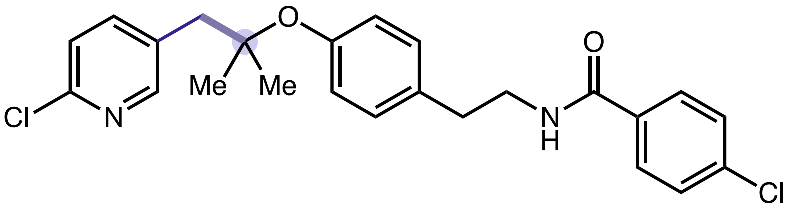

*Prepared according to **General Procedure A** with the following modifications: **NMP** instead of **DMA** was used as the optimal solvent.*

Prepared according to **General Procedure A** using Bezafibrate (180.9 mg, 0.5 mmol, 1.0 equiv.), 1-((6-chloropyridin-3-yl)methyl)-2,4,6-triphenylpyridin-1-ium tetrafluoroborate (390.6 mg, 0.75 mmol, 1.5 equiv.), K_2CO_3 (172.8 mg, 1.25 mmol, 2.5 equiv.), 4CzIPN (19.7 mg, 25.0 μmol, 5.0 mol%), Fe(OEP)Cl (15.6 mg, 25.0 μmol, 5.0 mol%), NMP (5.0 mL), and *i*-PrOH (5.0 mL).

After work-up according to **General Procedure A**, the crude residue was purified by automated flash column chromatography (2 × 25 g silica column, 0-60% EtOAc/hexanes) followed by automated reverse-phase column chromatography (30 g C18 column, 55-75% MeCN/H_2O with 0.1% NH_4OH) to afford the title compound as an off-white solid (152.8 mg, 69% yield).

^{1}H NMR (500 MHz, CDCl$_3$) δ 8.26 (s, 1H), 7.66 – 7.59 (m, 3H), 7.34 (d, *J* = 8.5 Hz, 2H), 7.26 (d, *J* = 8.2 Hz, 1H), 7.08 (d, *J* = 8.4 Hz, 2H), 6.82 (d, *J* = 8.4 Hz, 2H), 6.32 (t, *J* = 5.8 Hz, 1H), 3.64 (q, *J* = 6.7 Hz, 2H), 2.92 (s, 2H), 2.86 (t, *J* = 7.0 Hz, 2H), 1.22 (s, 6H).

^{13}C NMR (126 MHz, CDCl$_3$) δ 166.53, 153.55, 151.30, 149.72, 141.16, 137.71, 134.09, 133.09, 132.59, 129.45, 128.88, 128.37, 124.22, 123.59, 79.73, 45.47, 41.37, 34.99, 26.27.

IR (film) ν_{max} 3310, 3257, 2976, 2391, 1637, 1540, 1506, 1486, 1457, 1383, 1314, 1233, 1207, 1116, 1094, 1015, 901, 846, 750, 733, 587 cm^{-1}.

HRMS (ESI-TOF) *m/z* calculated for $C_{24}H_{25}Cl_2N_2O_2^+$ ($[M+H]^+$) 443.1288, found 443.1288.

(3*S*,4a*R*,6a*R*,6b*S*,8a*R*,11*R*,12*S*,12a*R*,14a*R*,14b*R*)-8a-((6-chloropyridin-3-yl)methyl)-4,4,6a,6b,11,12,14b-heptamethyl-1,2,3,4,4a,5,6,6a,6b,7,8,8a,9,10,11,12,12a,14,14a,14b-icosahydropicen-3-ol (64):

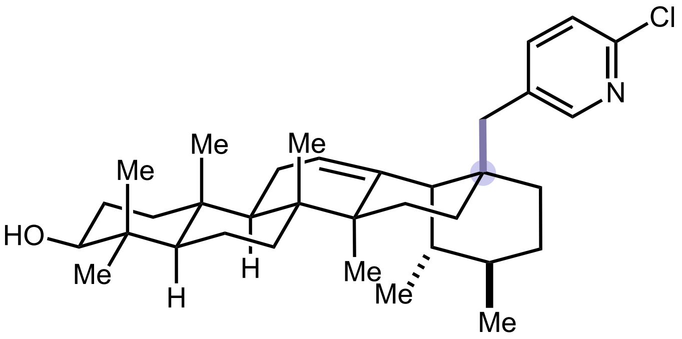

*Prepared according to **General Procedure A** with the following modifications: **NMP** instead of **DMA** was used as the optimal solvent.*

Prepared according to **General Procedure A** using ursolic acid (228.4 mg, 0.5 mmol, 1.0 equiv.), 1-((6-chloropyridin-3-yl)methyl)-2,4,6-triphenylpyridin-1-ium tetrafluoroborate (390.6 mg, 0.75 mmol, 1.5 equiv.), K_2CO_3 (172.8 mg, 1.25 mmol, 2.5 equiv.), 4CzIPN (19.7 mg, 25.0 μmol, 5.0 mol%), Fe(OEP)Cl (15.6 mg, 25.0 μmol, 5.0 mol%), NMP (5.0 mL), and *i*-PrOH (5.0 mL).

After work-up according to **General Procedure A**, the crude residue was purified by automated flash column chromatography (3 × 10 g silica column, 0-50% Et_2O/hexanes) followed by automated flash column chromatography (3 × 10 g silica column, 0-30% Et_2O/DCM) to afford the title compound as a crystalline off-white solid (365.2 mg, 73% yield, >20:1 d.r.).

^{1}H NMR (500 MHz, $CDCl_3$) δ 8.14 (s, 1H), 7.39 (dd, *J* = 8.2, 2.4 Hz, 1H), 7.19 (d, *J* = 8.1 Hz, 1H), 5.23 (t, *J* = 3.6 Hz, 1H), 3.23 (dd, *J* = 11.1, 5.1 Hz, 1H), 2.50 (q, *J* = 13.5 Hz, 2H), 2.13 – 1.90 (m, 4H), 1.71 – 1.53 (m, 6H), 1.50 – 1.27 (m, 6H), 1.27 – 0.65 (m, 30H).

^{13}C NMR (126 MHz, $CDCl_3$) δ 151.38, 149.02, 140.90, 139.14, 133.96, 126.06, 123.33, 79.07, 56.60, 55.28, 47.77, 42.00, 41.43, 40.26, 39.79, 39.22, 38.91, 38.89, 37.58, 37.01, 36.60, 32.87, 30.73, 28.26, 27.37, 26.28, 26.23, 23.80, 23.61, 21.36, 18.44, 17.60, 17.30, 15.80, 15.76.

IR (film) ν_{max} 3409, 2923, 2866, 2229, 1584, 1563, 1459, 1383, 1138, 1110, 1028, 996, 908, 829, 733 cm^{-1}.

HRMS (ESI-TOF) *m/z* calculated for $C_{35}H_{53}ClNO_2^+$ ($[M+H]^+$) 538.3810, found 538.3810.

4-(((3^3*R*,9^1*R*,9^2*R*,5*S*)-5-(*tert*-butyl)-17-methoxy-4,7-dioxo-2,8-dioxa-6-aza-1(2,3)-quinoxalina-3(3,1)-pyrrolidina-9(1,2)-cyclopropanacyclotetradecaphane-35-yl)methyl)benzonitrile (65):

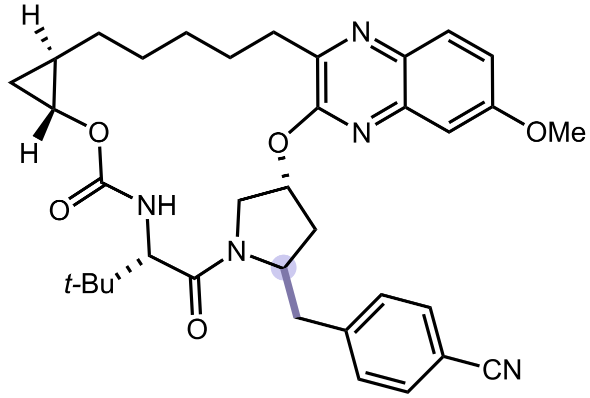

Prepared according to ***General Procedure A*** *with the following modifications:* ***NMP*** *instead of* ***DMA*** *was used as the optimal solvent.*

Prepared according to **General Procedure A** using (3^3*R*,3^5*S*,9^1*R*,9^2*R*,5*S*)-5-(*tert*-butyl)-17-methoxy-4,7-dioxo-2,8-dioxa-6-aza-1(2,3)-quinoxalina-3(3,1)-pyrrolidina-9(1,2)-cyclopropanacyclotetradecaphane-35-carboxylic acid (138.7 mg, 0.25 mmol, 1.0 equiv.), 1-(4-cyanobenzyl)-2,4,6-triphenylpyridin-1-ium tetrafluoroborate (191.4 mg, 0.375 mmol, 1.5 equiv.), K_2CO_3 (86.4 mg, 0.625 mmol, 2.5 equiv.), 4CzIPN (9.9 mg, 12.5 μmol, 5.0 mol%), Fe(OEP)Cl (7.8 mg, 12.5 μmol, 5.0 mol%), NMP (2.5 mL), and *i*-PrOH (2.5 mL).

After work-up according to **General Procedure A**, the crude residue was purified by automated flash column chromatography (3 × 10 g silica column, 0-40% EtOAc/hexanes) followed by automated reverse-phase column chromatography (30 g C18 column, 65-90% MeCN/H_2O with 0.1% NH_4OH) to afford the title compound as an off-white solid (mixture of diastereomers, 4:1 d.r., 59.0 mg, 38% yield).

^{1}H NMR (500 MHz, CDCl$_3$) δ 7.78 (d, *J* = 9.0 Hz, 1H) [7.86 (d, *J* = 9.1 Hz, 1H)], 7.55 (d, *J* = 8.1 Hz, 2H) [7.44 (d, *J* = 8.0 Hz, 1H)], 7.35 (d, *J* = 8.2 Hz, 2H), 7.14 (dd, *J* = 9.0, 2.6 Hz, 1H) [7.22 (dd, *J* = 9.1, 2.8 Hz, 1H)], 7.05 (d, *J* = 2.8 Hz, 1H), 5.83 (t, *J* = 4.3 Hz, 1H) [6.06 (t, *J* = 4.3 Hz, 1H)], 5.35 (d, *J* = 9.9 Hz, 1H) [5.26 (d, *J* = 10.1 Hz, 1H)], 4.48 – 4.33 (m, 3H) [4.63 (d, *J* = 12.0 Hz, 1H)], 3.92 – 3.80 (m, 6H) [4.03 – 3.98 (m, 6H)], 3.09 – 2.81 (m, 1H), 2.70 (ddd, *J* = 14.1, 12.0, 4.8 Hz, 1H), 2.43 (dd, *J* = 12.8, 10.0 Hz, 1H), 2.25 (dd, *J* = 14.6, 7.1 Hz, 1H), 2.16 (s, 1H) [2.14 (s, 1H)] 2.02 (ddd, *J* = 14.3, 9.5, 4.6 Hz, 1H), 1.82 – 1.36 (m, 7H), 1.06 (s, 9H), 1.04 – 0.88 (m, 2H), 0.73 – 0.63 (m, 1H), 0.54 – 0.44 (m, 1H) **(minor diastereomer in brackets).**

^{13}C NMR (126 MHz, CDCl$_3$) δ 171.63, 171.28, 160.56, 160.28, 157.78, 157.31, 155.04, 154.92, 148.65, 148.13, 144.66, 143.94, 141.17, 140.83, 134.49, 134.38, 132.49, 130.15, 130.05, 129.35, 129.28, 118.92, 118.91, 118.88, 118.48, 110.68, 110.37, 106.11, 106.03, 74.89, 73.49, 59.79, 59.59, 58.80, 57.82, 55.80, 55.75, 55.65, 55.41, 54.70, 54.64, 40.38, 38.83, 37.68, 35.22, 34.18, 34.15, 33.76, 33.60, 31.11, 31.06, 30.65, 29.55, 29.51, 29.21, 28.79, 28.21, 28.16, 26.61, 26.56, 26.45, 19.42, 18.77, 11.35, 11.15. **(summary of diastereomers)**

IR (film) ν_{max} 3383, 2931, 2855, 2229, 1720, 1638, 1581, 1502, 1417, 1319, 1224, 1129, 1071, 1030, 910, 829, 731, 600, 562 cm^{-1}.

HRMS (ESI-TOF) *m/z* calculated for $C_{36}H_{43}N_5NaO_5^+$ ([M+H]$^+$) 648.3156, found 648.3154.

tert-butyl 2-(4-bromobenzyl)-4-hydroxy-4-methylpyrrolidine-1-carboxylate ((±)-66):

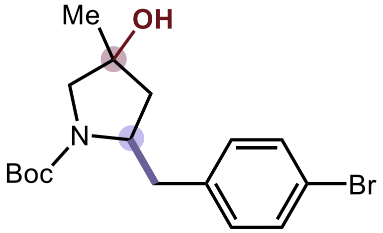

Prepared according to ***General Procedure A*** *with the following modifications:* ***NMP*** *instead of* ***DMA*** *was used as the optimal solvent.*

The carboxylic acid starting material was prepared according to a literature procedure.[15] The title compound was prepared according to **General Procedure A** *on 0.5 mmol scale in 2 × 40-mL vials* using 1-(*tert*-butoxycarbonyl)-4-hydroxy-4-methylpyrrolidine-2-carboxylic acid (123.9 mg, 0.5 mmol, 1.0 equiv.), 1-(4-bromobenzyl)-2,4,6-triphenylpyridin-1-ium tetrafluoroborate (423.2 mg, 0.75 mmol, 1.5 equiv.), K_2CO_3 (172.8 mg, 1.25 mmol, 2.5 equiv.), 4CzIPN (19.7 mg, 25 μmol, 5.0 mol%), Fe(OEP)Cl (15.6 mg, 25 μmol, 5.0 mol%), NMP (5 mL), and *i*-PrOH (5 mL). Contents in the two vials were combined for work-up.

After work-up according to **General Procedure A**, the crude residue was purified by automated flash column chromatography (25 g silica column, 0-10-40% EtOAc/hexanes) followed by automated reverse-phase column chromatography (30 g C18 column, 10-50% MeCN/H_2O with 0.1% NH_4OH) to afford the title compound as a white amorphous solid (195.4 mg, 53% yield).

^{1}H NMR (500 MHz, $CDCl_3$) δ 7.46 – 7.30 (m, 2H), 7.18 – 6.92 (m, 2H), 4.37 – 3.84 (m, 1H), 3.78 – 3.07 (m, 2H), 3.08 – 2.84 (m, 1H), 2.85 – 2.46 (m, 1H), 2.02 – 1.71 (m, 1H), 1.63 – 1.43 (m, 10H), 1.33 (m, 3H). **(summary of diastereomers)**

[15] Daiichi Sankyo Co., Ltd. Imidazothiazole Derivatives Having a 4,7-Diazaspiro[2.5]octane Ring Structure. EP Patent 2298778, 2011.

^{13}C NMR (126 MHz, $CDCl_3$) δ 155.19, 154.69, 137.33, 131.51, 131.25, 120.27, 120.10, 79.94, 79.69, 75.56, 60.18, 59.90, 59.56, 59.12, 58.05, 57.67, 45.36, 44.83, 43.01, 42.46, 40.38, 40.11, 39.15, 38.81, 28.66, 24.85. **(summary of diastereomers)**

IR (film) ν_{max} 3413, 2973, 2932, 2874, 1667, 1487, 1454, 1406, 1365, 1256, 1157, 1097, 1071, , 1011, 908, 802, 773, 730, 646 cm^{-1}.

HRMS (ESI-TOF) *m/z* calculated for $C_{13}H_{17}BrNO_3^+$ ($[M+H–isobutylene]^+$) 314.0386, found 314.0385.

***tert*-butyl 8-bromo-1-methyl-1,2,4,5-tetrahydro-3*H*-1,4-methanobenzo[*d*]azepine-3-carboxylate ((±)-67):**

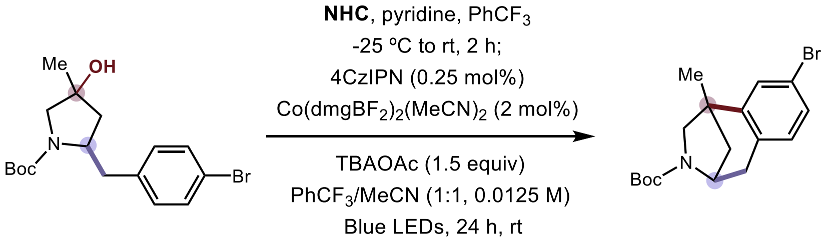

Stock solution 1: A 4-mL vial was charged with 4CzIPN (1.5 mg, 1.875 μmol), and dry acetonitrile (3 mL) under air. The mixture was sonicated until dissolved to form a light-yellow solution.

Reaction vial: A 40-mL vial was charged with tetrabutylammonium acetate (113.2 mg, 0.375 mmol, 1.5 equiv), $Co(dmgBF_2)_2(MeCN)_2$ (2.3 mg, 5 μmol, 2 mol%) and an X-shaped stir bar. The vessel was capped and sealed with electrical tape before dry acetonitrile (9 mL) and 1 mL of **Stock solution 1** [containing 4CzIPN (0.5 mg, 0.625 μmol, 0.25 mol%)] were sequentially added via syringe.

NHC Condensation vial: A 40-mL vial was charged with *tert*-butyl 2-(4-bromobenzyl)-4-hydroxy-4-methylpyrrolidine-1-carboxylate (92.6 mg, 0.25 mmol, 1.0 equiv.) and an X-shaped stir bar. The vessel was capped, evacuated, and backfilled with N_2 three times before $PhCF_3$ (5 mL) and pyridine (40 μL, 0.5 mmol, 2 equiv) was added via syringe. The reaction mixture was stirred for 10 minutes at room temperature and subsequently cooled down to -25 ˚C with a dry ice/*i*-PrOH bath (temperature monitored with a thermometer), and NHC (256 mg, 0.5 mmol, 2 equiv) was added portion-wise. The mixture was stirred at -25 ˚C and allowed to gradually warm up to 0 ˚C over the course of 2 hours, during which a white solid precipitated out, and the mixture usually turned from pale yellow to red.

Upon completion of NHC condensation, the resulting heterogeneous mixture was transferred into **Reaction vial** via syringe filter. PhCF3 (2.5 mL, two times) was added to rinse the **NHC Condensation vial** and subsequently transferred into **Reaction vial** through the same syringe filter. The homogeneous solution in **Reaction vial** was then sparged with N_2 for 15 minutes before it was sealed on top with melted parafilm. Reaction vial was irradiated with 450 nm LED modules at 100% light intensity with maximum fan speed and 500 rpm stir rate in a PennPhD Integrated Photoreactor for 24 hours.

The reaction mixture was concentrated and the resulting residue was purified by automated column chromatography (25 g silica column, 0-10% Et_2O/hexanes) to afford the title compound as a white amorphous solid (67.3 mg, 76% yield).

^{1}H NMR (500 MHz, $CDCl_3$) δ 7.40 – 7.37 (m, 1H), 7.31 – 7.27 (m, 1H), 7.00 – 6.94 (m, 1H), 4.48 – 4.27 (m, 1H), 3.40 – 3.27 (m, 1H), 3.25 – 3.18 (m, 1H), 3.18 – 2.97 (m, 1H), 2.95 – 2.82 (m, 1H), 2.03 – 1.92 (m, 1H), 1.89 – 1.81 (m, 1H), 1.53 – 1.49 (m, 3H), 1.48 – 1.38 (m, 9H). **(summary of rotamers)**

^{13}C NMR (126 MHz, $CDCl_3$) δ 154.02, 154.00, 147.19, 147.04, 133.24, 132.84, 131.58, 131.28, 129.73, 126.93, 126.75, 119.94, 119.77, 79.42, 79.35, 61.50, 61.09, 54.39, 53.97, 42.03, 41.89, 41.40, 41.00, 36.99, 36.48, 28.71, 28.59, 20.96, 20.92. **(summary of rotamers)**

IR (film) ν_{max} 2966, 2929, 2866, 1689, 1481, 1456, 1392, 1365, 1326, 1248, 1179, 1160, 1144, 1122, 1098, 935, 893, 801, 770, 732 cm^{-1}.

HRMS (ESI-TOF) *m/z* calculated for $C_{13}H_{15}BrNO_2^+$ ($[M+H–isobutylene]^+$) 296.0281, found 296.0283.

***tert*-butyl (6-bromo-2,2-dimethyl-3,4-dihydro-2*H*-pyrano[2,3-*b*]pyridin-3-yl)carbamate ((±)-68):**

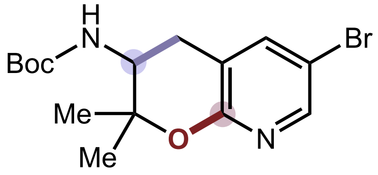

The acylic intermediate was prepared according to **General Procedure A** using (*S*)-2-((*tert*-butoxycarbonyl)amino)-3-hydroxy-3-methylbutanoic acid (58.3 mg, 0.25 mmol, 1.0 equiv.), 1-((5-bromo-2-fluoropyridin-3-yl)methyl)-2,4,6-triphenylpyridin-1-ium tetrafluoroborate (218.7 mg, 0.375 mmol, 1.5 equiv.), K_2CO_3 (86.4 mg, 0.625 mmol, 2.5 equiv.), 4CzIPN (9.9 mg, 12.5 μmol, 5.0 mol%), Fe(OEP)Cl (7.8 mg, 12.5 μmol, 5.0 mol%), DMA (2.5 mL), and *i*-PrOH (2.5 mL).

After work-up according to **General Procedure A**, the crude residue was dissolved in THF (3.0 mL), and KO*t*-Bu (30.9 mg, 0.275 mmol, 1.1 equiv.) was added in one portion. The reaction was stirred at room temperature for 24 hours. After complete conversion to the desired cyclized product, the reaction mixture was concentrated *in vacuo*, and the crude residue was purified by automated flash column chromatography (3 × 10 g silica column, 0-40% EtOAc/hexanes) to afford the title compound as a tan solid (29.8 mg, 33% yield).

^{1}H NMR (500 MHz, $CDCl_3$) δ 8.18 (s, 1H), 7.55 (s, 1H), 4.68 (d, *J* = 9.5 Hz, 1H), 3.99 – 3.91 (m, 1H), 3.13 (dd, *J* = 17.3, 5.2 Hz, 1H), 2.75 (dd, *J* = 17.4, 3.6 Hz, 1H), 1.44 (s, 3H), 1.42 (s, 9H), 1.36 (s, 3H).

^{13}C NMR (126 MHz, $CDCl_3$) δ 158.66, 155.53, 148.05, 141.80, 115.94, 112.28, 80.10, 79.14, 49.12, 29.52, 28.45, 25.30, 24.44.

IR (film) ν_{max} 3281, 2979, 2933, 1709, 1565, 1531, 1440, 1400, 1366, 1286, 1158, 1051, 751 cm^{-1}.

HRMS (ESI-TOF) *m/z* calculated for $C_{11}H_{14}BrN_2O_3^+$ ($[M+H–isobutylene]^+$) 301.0182, found 301.0182.

***tert*-butyl (2*R*,3*S*)-3-hydroxy-2-(4-(methoxycarbonyl)benzyl)pyrrolidine-1-carboxylate (69):**

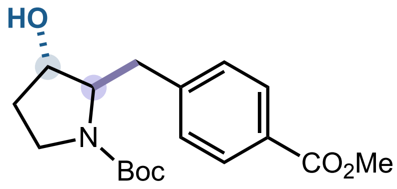

Prepared according to **General Procedure A** *on 1.0 mmol scale in 2 × 40-mL vials* using (2*S*,3*S*)-1-(*tert*-butoxycarbonyl)-3-hydroxypyrrolidine-2-carboxylate (231.3 mg, 1.0 mmol, 1.0 equiv.), 1-(4-(methoxycarbonyl)benzyl)-2,4,6-triphenylpyridin-1-ium tetrafluoroborate (815.0 mg, 1.5 mmol, 1.5 equiv.), K_2CO_3 (345.5 mg, 2.50mmol, 2.5 equiv.), 4CzIPN (39.4 mg, 50.0 μmol, 5.0 mol%), Fe(OEP)Cl (31.2 mg, 50.0 μmol, 5.0 mol%), NMP (10.0 mL), and *i*-PrOH (10.0 mL).

After work-up according to **General Procedure A**, the crude residue was purified by automated flash column chromatography (100 g silica column, 10-100% EtOAc/hexanes) followed by automated reverse-phase column chromatography (30 g C18 column, 35-60% MeCN/H_2O with 0.1% NH_4OH) to afford the title compound as an off-white gum (237.8 mg, 71% yield, >20:1 d.r.).

^{1}H NMR (500 MHz, $CDCl_3$) δ 7.95 (d, *J* = 7.5 Hz, 2H), 7.26 (d, *J* = 7.6 Hz, 2H), 4.10 (s 1H), 3.98 – 3.82 (m, 1H), 3.89 (s, 3H), 3.60 – 3.28 (m, 2H), 3.23 – 3.00 (m, 1H), 2.61 – 2.49 (m, 1H), 2.02 – 1.69 (m, 1H), 1.48 (s, 9H).

^{13}C NMR (126 MHz, $CDCl_3$) δ 167.31, 167.12, 154.81, 154.72, 143.99, 143.91, 130.04, 129.93, 129.86, 129.70, 129.57, 129.41, 128.62, 128.50, 79.93, 79.66, 76.91, 74.43, 73.59, 71.72, 67.45, 52.21, 52.14, 44.70, 44.23, 39.53, 38.58, 31.71, 31.21, 28.66, 28.58. **(summary of rotamers)**

IR (film) ν_{max} 3422, 2976, 1719, 1667, 1398, 1277, 1177, 1105, 1032, 986, 908, 964, 729, 646, 540 cm^{-1}.

HRMS (ESI-TOF) *m/z* calculated for $C_{18}H_{25}NNaO_5^+$ ([M+Na]$^+$) 358.1625, found 358.1627.

tert-butyl (_S_)-2-(4-(methoxycarbonyl)benzyl)pyrrolidine-1-carboxylate (70):

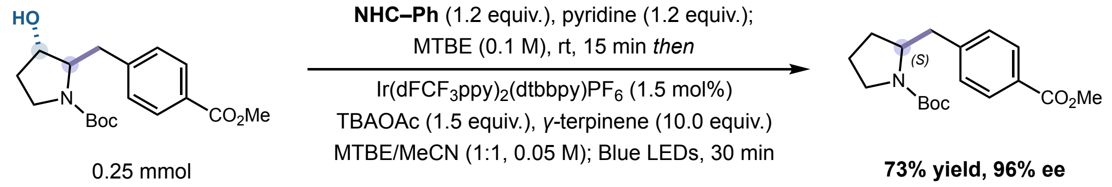

Catalysts and reagents. To an oven-dried 40 mL vial equipped with a cross stir bar was added Ir[(dFCF$_3$ppy)$_2$(dtbbpy)]PF$_6$ (4.2 mg, 3.75 µmol, 1.5 mol%) and TBAOAc (113.1 mg, 0.375 mmol, 1.5 equiv.). MeCN (2.5 mL) was then added via syringe while stirring to form a homogenous solution, after which γ-terpinene (400 µL, 2.5 mmol, 10.0 equiv.) was added via syringe in one portion.

Alcohol activation and reaction set-up. To an oven-dried 8 mL vial equipped with a stir bar was added _tert_-butyl (2_R_,3_S_)-3-hydroxy-2-(4-(methoxycarbonyl)benzyl)pyrrolidine-1-carboxylate (83.9 mg, 0.25 mmol, 1.0 equiv.) and **NHC–Ph** (5,7-di-_tert_-butyl-3-phenylbenzo[_d_]oxazol-3-ium tetrafluoroborate, 118.6 mg, 0.3 mmol, 1.2 equiv.). Next, the vial was evacuated and back-filled three times with N_2, after which MTBE (2.5 mL) was added via syringe. At this point, pyridine (24 µL, 0.3 mmol 1.2 equiv.) was added dropwise. The resulting suspension was then stirred at room temperature for 15 min, after which it was syringe-filtered to remove pyridinium salts and unreacted **NHC**. The resultant solution was then injected into the reaction vial containing the catalysts and reagents. The septum was then punctured with three 16 G needles, and the vial was placed in a PennPhD m1 integrated photoreactor. The reaction was irradiated with 450 nm blue LEDs (100% light intensity, 5200 rpm fans, 1000 rpm stirring) for 30 min, after which the reaction mixture was concentrated _in vacuo_. The residue was purified by automated normal-phase flash column chromatography (2 × 25 g silica column, 5-60% Et_2O/hexanes) to afford the title compound as a colourless oil (58.4 mg, 73% yield).

^{1}H NMR (500 MHz, CDCl$_3$) δ 7.95 (d, J = 8.9 Hz, 2H), 7.25 (d, J = 8.9 Hz, 2H), 4.10 – 3.93 (m, 1H), 3.90 (s, 3H), 3.43 – 3.00 (m, 3H), 2.71 – 2.56 (m, 1H), 1.82 – 1.58 (m, 4H), 1.49 (s, 9H). Spectroscopic data are consistent with previous report.[16]

Chiral HPLC. ChiralCEL AS–H column, 3% isopropanol/hexanes, 1.0 mL/min, 96% ee, t_S = 9.74 min (major (*S*)-enantiomer), t_R = 8.42 min (minor (*R*)-enantiomer).

The racemate was prepared according to **General Procedure A** using Boc-Pro-OH (107.6 mg, 0.5 mmol, 1.0 equiv.), 1-(4-(methoxycarbonyl)benzyl)-2,4,6-triphenylpyridin-1-ium tetrafluoroborate (407.5 mg, 0.75 mmol, 1.5 equiv.), K_2CO_3 (172.8 mg, 1.25 mmol, 2.5 equiv.), 4CzIPN (19.7 mg, 25.0 μmol, 5.0 mol%), Fe(OEP)Cl (15.6 mg, 25.0 μmol, 5.0 mol%), DMA (5.0 mL), and *i*-PrOH (5.0 mL).

After work-up according to **General Procedure A**, the crude residue was purified by automated flash column chromatography (2 × 25 g silica column, 0-25% EtOAc/hexanes) followed by automated flash column chromatography (2 × 10 g silica column, 0-25% Et_2O/DCM) to afford the racemate as a colourless oil.

Chiral HPLC traces of racemate (±)-70

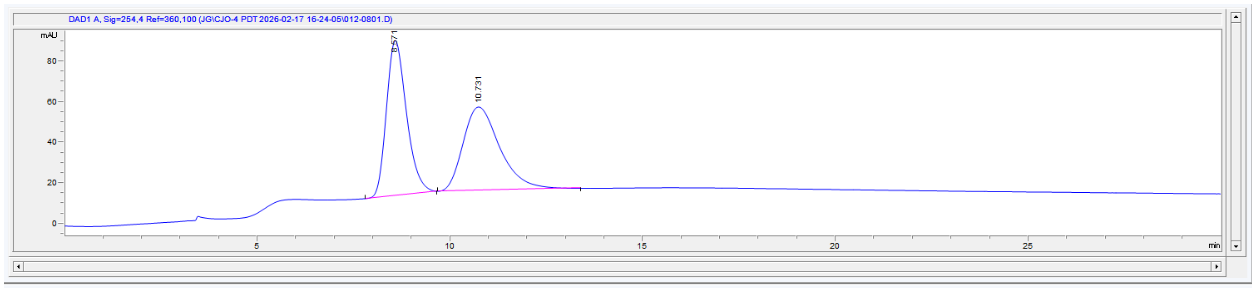

#	Time	Area	Height	Width	Area%	Symmetry
1	8.571	2769.5	76.5	0.5523	50.296	0.746
2	10.731	2736.9	41.2	1.0113	49.704	0.677

[16] Massah, A.R; Ross, A.J.; Jackson, R.F.W. In Situ trapping of Boc-2-pyrrolidinylmethylzinc Iodide with Aryl Iodides: Direct Synthesis of 2-Benzylpyrrolidines. *J. Org. Chem.* **2010,** *75* (23), 8275–8278. DOI: 10.1021/jo101503p.

Chiral HPLC traces of isolated, enantioenriched 70

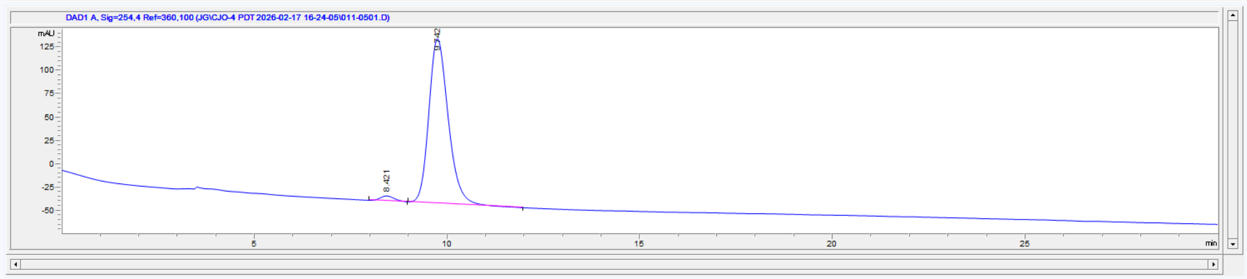

#	Time	Area	Height	Width	Area%	Symmetry
1	8.421	136.2	5.4	0.3795	2.156	0.804
2	9.742	6182.5	176.6	0.5384	97.844	0.752

benzyl 2-benzylpyrrolidine-1-carboxylate:

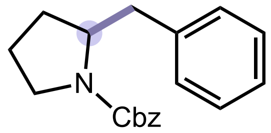

Prepared according to **General Procedure A** using Z-Pro-OH (124.6 mg, 0.5 mmol, 1.0 equiv.), 1-benzyl-2,4,6-triphenylpyridin-1-ium tetrafluoroborate (485.3 mg, 0.75 mmol, 1.5 equiv.), K_2CO_3 (172.8 mg, 1.25 mmol, 2.5 equiv.), 4CzIPN (19.7 mg, 25.0 μmol, 5.0 mol%), Fe(OEP)Cl (15.6 mg, 25.0 μmol, 5.0 mol%), DMA (5.0 mL), and *i*-PrOH (5.0 mL).

After work-up according to **General Procedure A**, the crude residue was purified by automated reverse-phase column chromatography (60 g C18 column, 60-80% MeCN/H_2O with 0.1% NH_4OH) to afford the title compound as a light-yellow oil (80.5 mg, 55% yield).

^{1}H NMR (500 MHz, CDCl$_3$) δ 7.48 – 6.97 (m, 10H), 5.19 (s, 2H), 4.09 (d, *J* = 27.4 Hz, 1H), 3.64 – 3.31 (m, 2H), 3.12 (dd, *J* = 83.4, 10.9 Hz, 1H), 2.66 – 2.53 (m, 1H), 1.85 – 1.69 (m, 4H).

^{13}C NMR (126 MHz, CDCl$_3$) δ 155.07, 154.95, 139.07, 138.99, 137.29, 137.03, 129.70, 129.49, 128.61, 128.49, 128.45, 128.18, 128.11, 128.00, 127.93, 126.38, 126.30, 67.06, 66.61, 59.40, 58.93, 46.97, 46.72, 40.73, 39.55, 29.84, 29.00, 23.57, 22.77. **(summary of rotamers)**

IR (film) ν_{max} 3028, 2952, 2876, 1697, 1497, 1453, 1410, 1357, 1337, 1188, 1094, 769, 699 cm^{-1}.

HRMS (ESI-TOF) *m/z* calculated for $C_{19}H_{22}NO_2^+$ ([M+H]$^+$) 296.1645, found 296.1647.

1,2-bis(6-chloropyridin-3-yl)ethane :

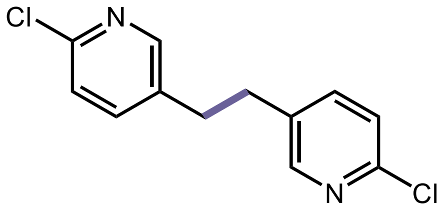

^{1}H NMR (500 MHz, CDCl$_3$) δ 8.17 (d, *J* = 2.5 Hz, 1H), 7.38 (dd, *J* = 8.1, 2.5 Hz, 1H), 7.24 (d, *J* = 8.1 Hz, 1H), 2.91 (s, 2H).

^{13}C NMR (126 MHz, CDCl$_3$) δ 149.73, 138.94, 134.59, 124.21, 33.68.

IR (film) ν_{max} 3066, 2918, 2854, 1586, 1564, 1464, 1443, 1377, 1308, 1182, 1139, 1110, 1096, 1079, 1025, 918, 835, 814, 747, 645, 633 cm^{-1}.

HRMS (ESI-TOF) *m/z* calculated for $C_{12}H_{11}Cl_2N_2^+$ ([M+H]$^+$) 253.0294, found 253.0298.

9. NMR Spectral Data

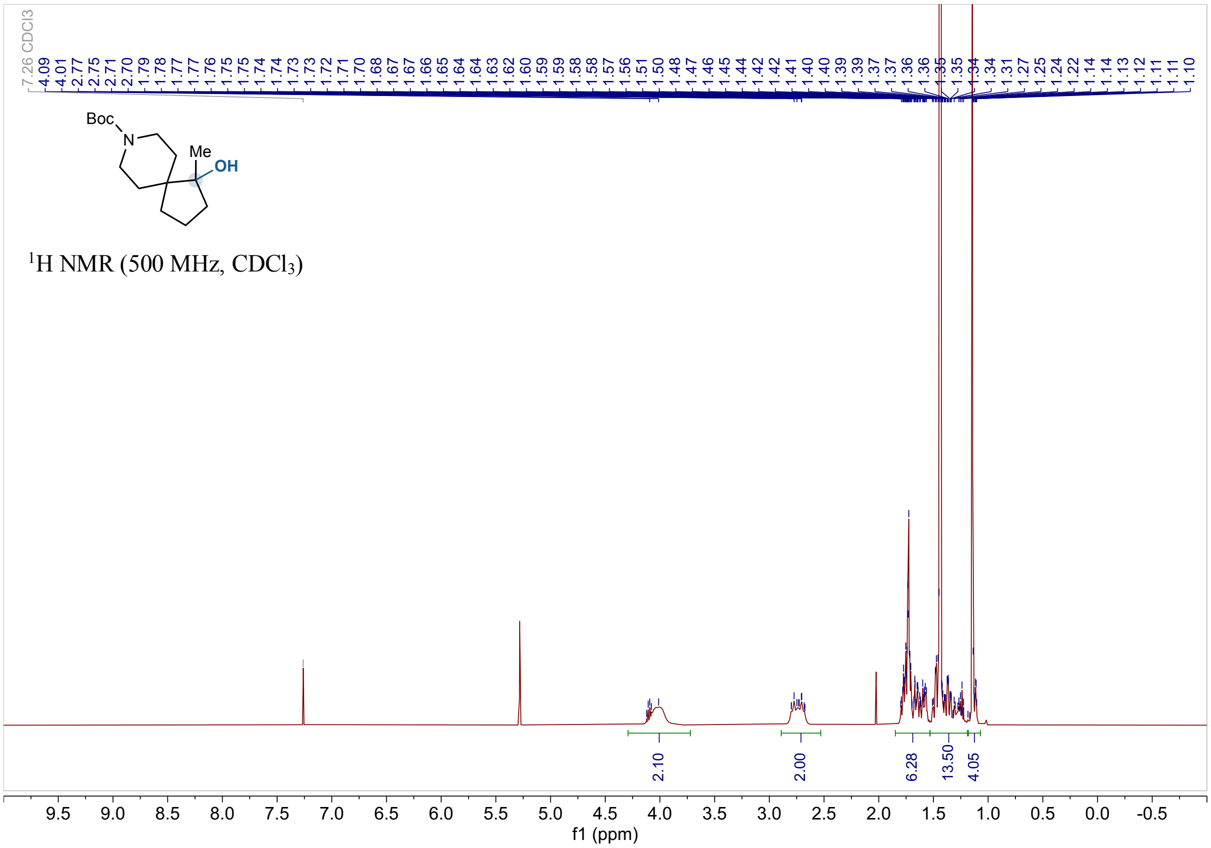

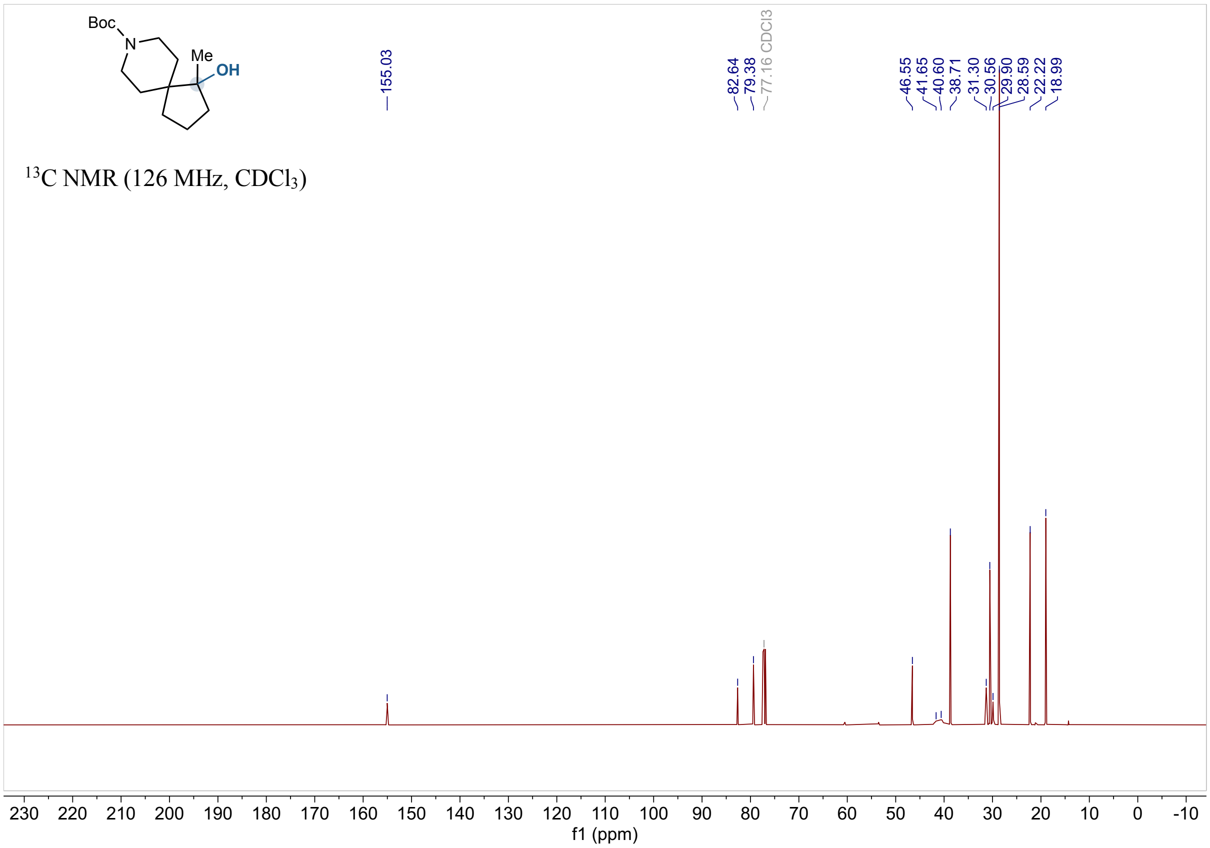

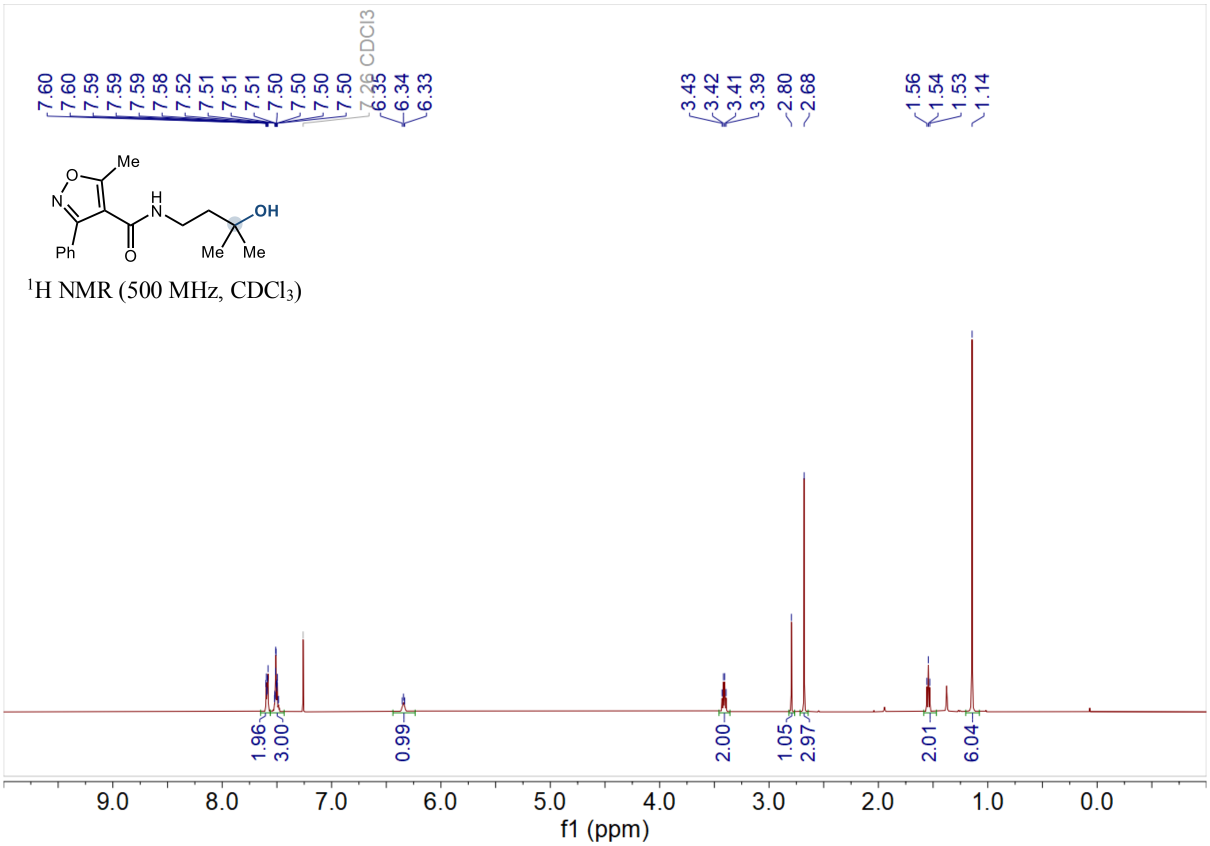

^{1}H NMR (500 MHz, $CDCl_3$)

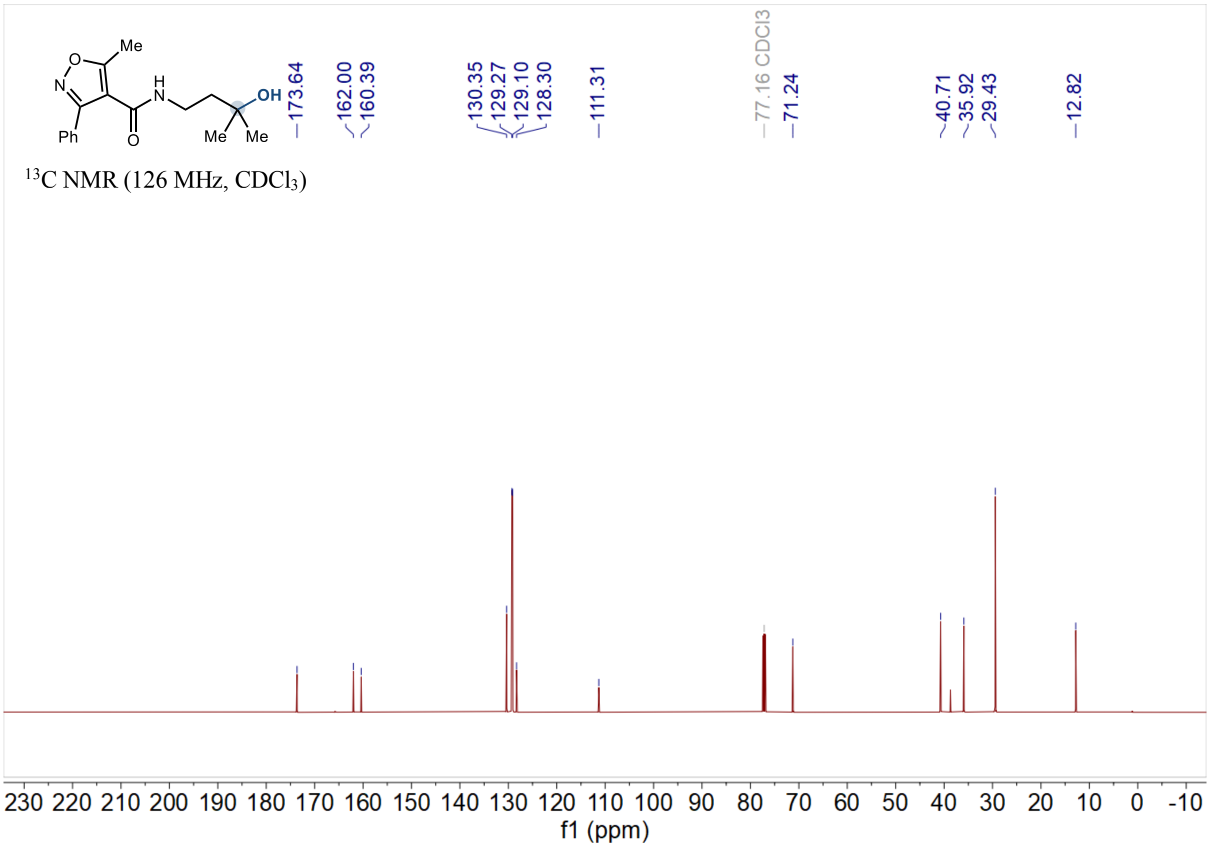

^{13}C NMR (126 MHz, $CDCl_3$)

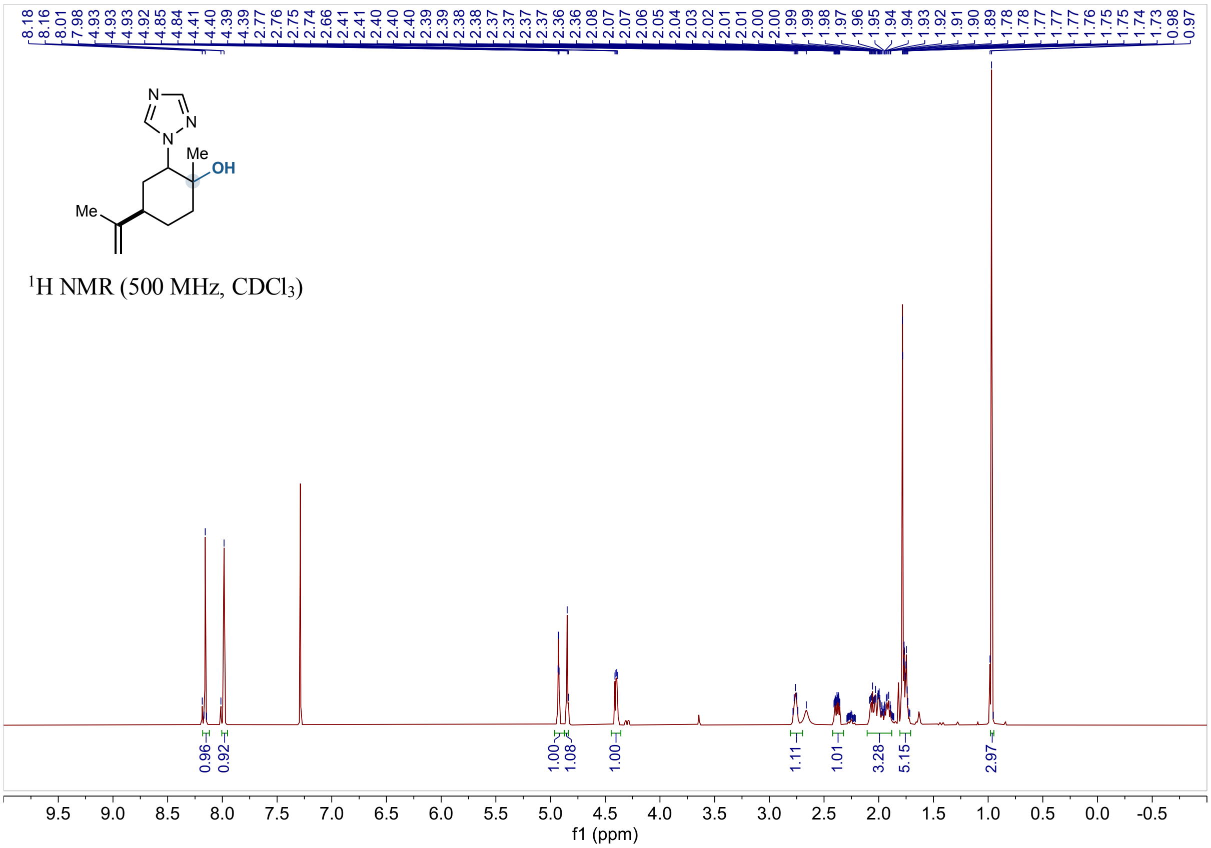
8.18 8.16 8.01 7.98 4.93 4.93 4.93 4.92 4.85 4.84 4.41 4.40 4.39 4.39 2.77 2.76 2.75 2.74 2.66 2.41 2.41 2.40 2.40 2.40 2.39 2.39 2.38 2.38 2.37 2.37 2.37 2.37 2.36 2.36 2.08 2.07 2.07 2.06 2.05 2.04 2.03 2.02 2.01 2.01 2.00 2.00 1.99 1.99 1.98 1.97 1.96 1.95 1.94 1.94 1.93 1.92 1.91 1.90 1.89 1.78 1.78 1.77 1.77 1.77 1.76 1.75 1.75 1.74 1.73 0.98 0.97
N
N
N
Me
OH
Me
^{1}H NMR (500 MHz, $CDCl_3$)
0.96 0.92 1.00 1.08 1.00 1.11 1.01 3.28 5.15 2.97
9.5 9.0 8.5 8.0 7.5 7.0 6.5 6.0 5.5 5.0 4.5 4.0 3.5 3.0 2.5 2.0 1.5 1.0 0.5 0.0 -0.5
f1 (ppm)

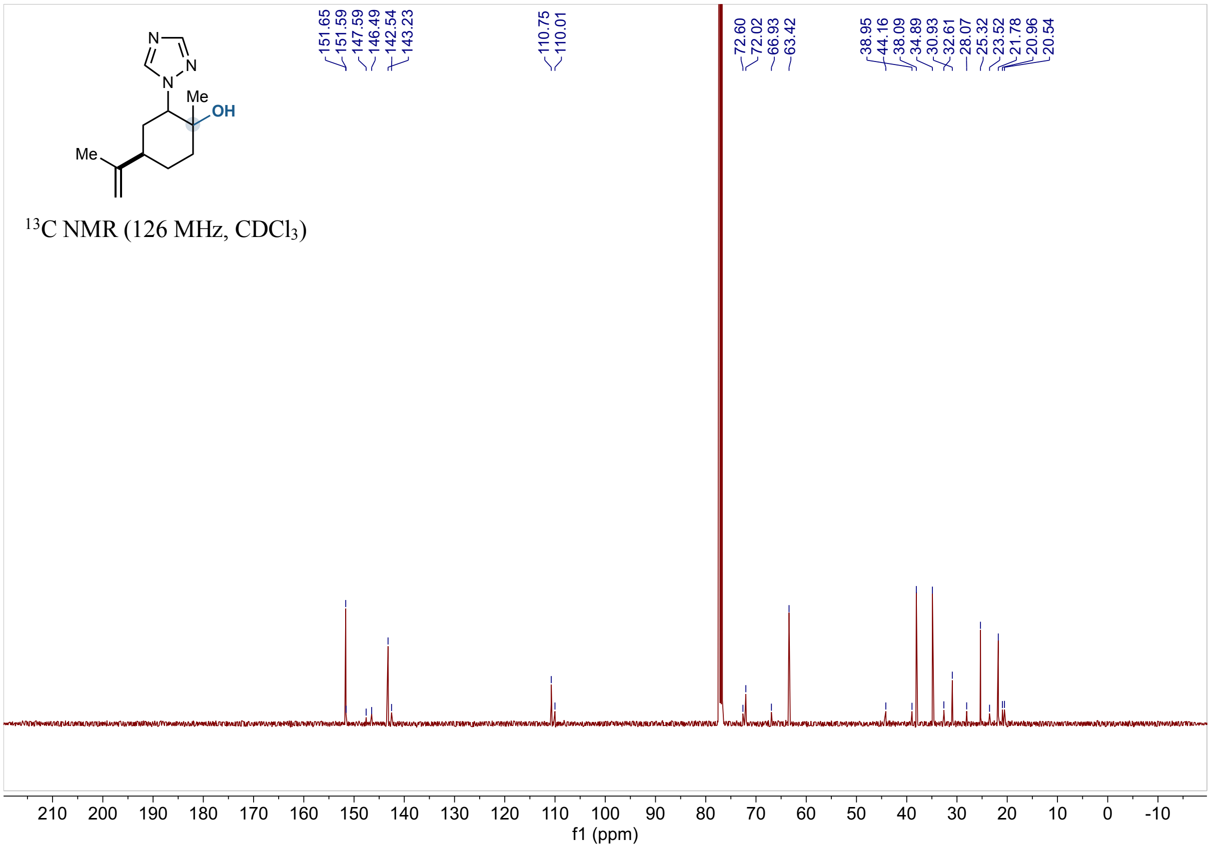
151.65 151.59 147.59 146.49 142.54 143.23
110.75 110.01
72.60 72.02 66.93 63.42
38.95 44.16 38.09 34.89 30.93 32.61 28.07 25.32 23.52 21.78 20.96 20.54
N
N
N
Me
OH
Me
^{13}C NMR (126 MHz, $CDCl_3$)
210 200 190 180 170 160 150 140 130 120 110 100 90 80 70 60 50 40 30 20 10 0 -10
f1 (ppm)

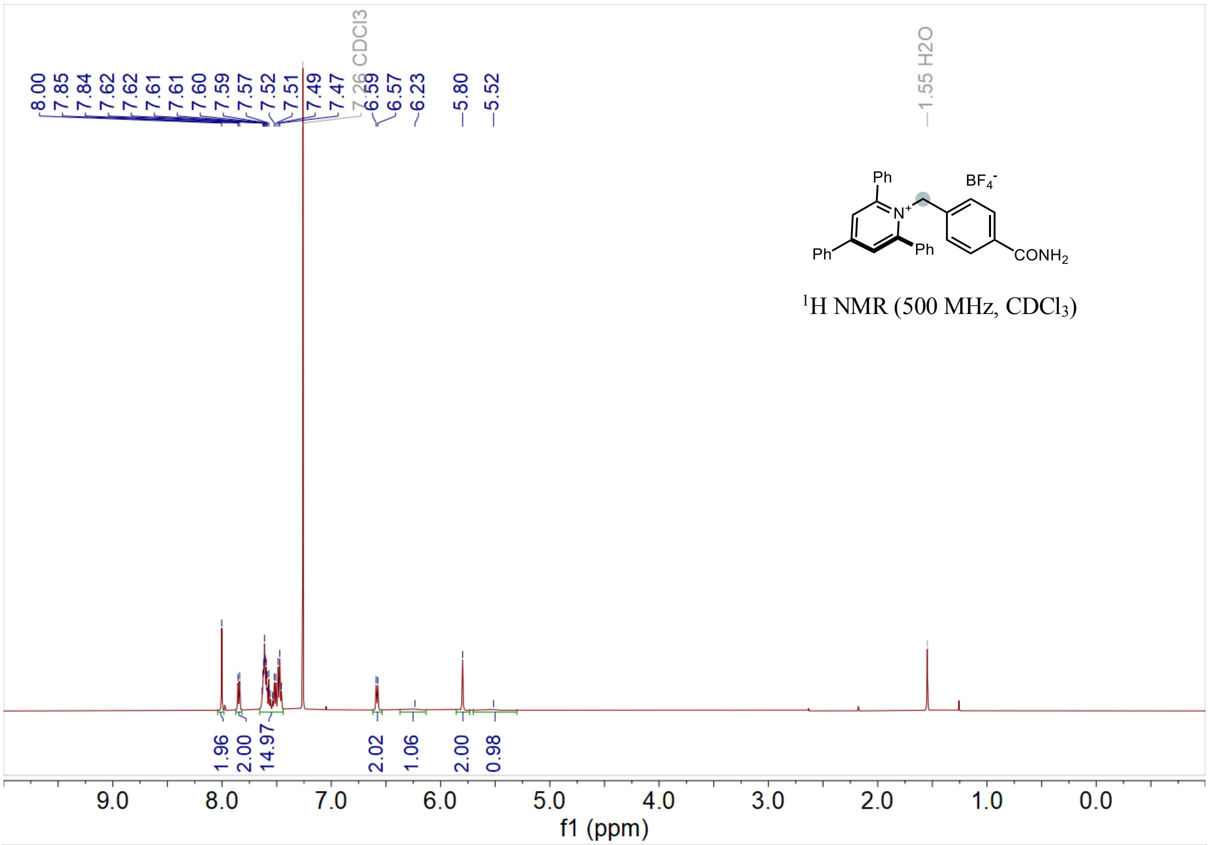

8.00
7.85
7.84
7.62
7.62
7.61
7.61
7.60
7.59
7.57
7.52
7.51
7.49
7.47
7.26 CDCl3
6.59
6.57
6.23
5.80
5.52
1.55 H2O
Ph
BF4-
N+
Ph
Ph
CONH2
1H NMR (500 MHz, CDCl3)
1.96
2.00
14.97
2.02
1.06
2.00
0.98
9.0
8.0
7.0
6.0
5.0
4.0
3.0
2.0
1.0
0.0
f1 (ppm)

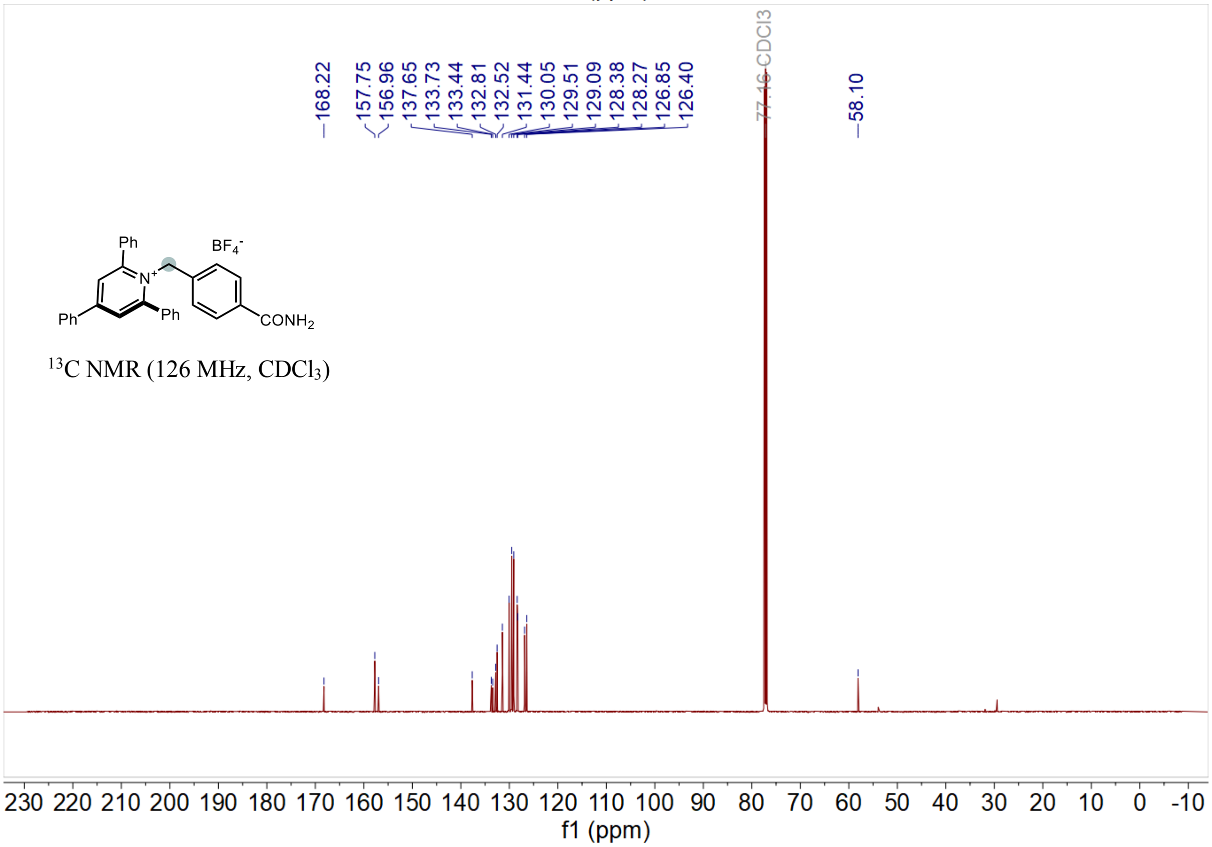

168.22
157.75
156.96
137.65
133.73
133.44
132.81
132.52
131.44
130.05
129.51
129.09
128.38
128.27
126.85
126.40
77.16 CDCl3
58.10
Ph
BF4-
N+
Ph
Ph
CONH2
13C NMR (126 MHz, CDCl3)
230 220 210 200 190 180 170 160 150 140 130 120 110 100 90 80 70 60 50 40 30 20 10 0 -10
f1 (ppm)

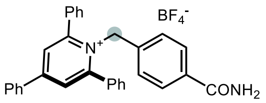

^{19}F NMR (376 MHz, $CDCl_3$)

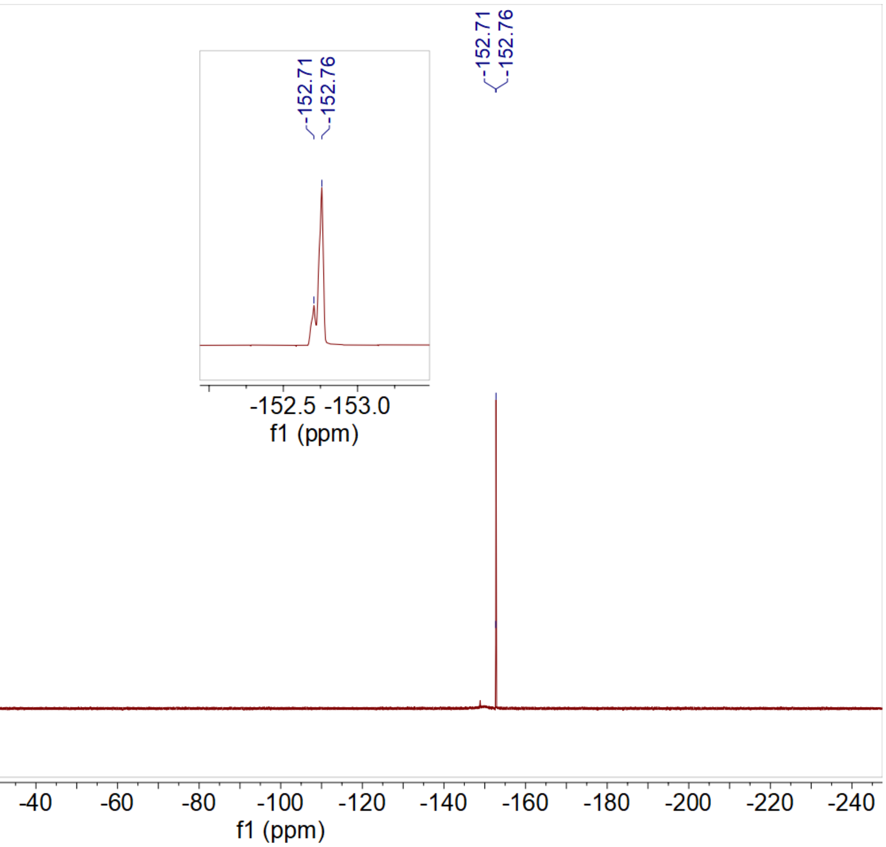

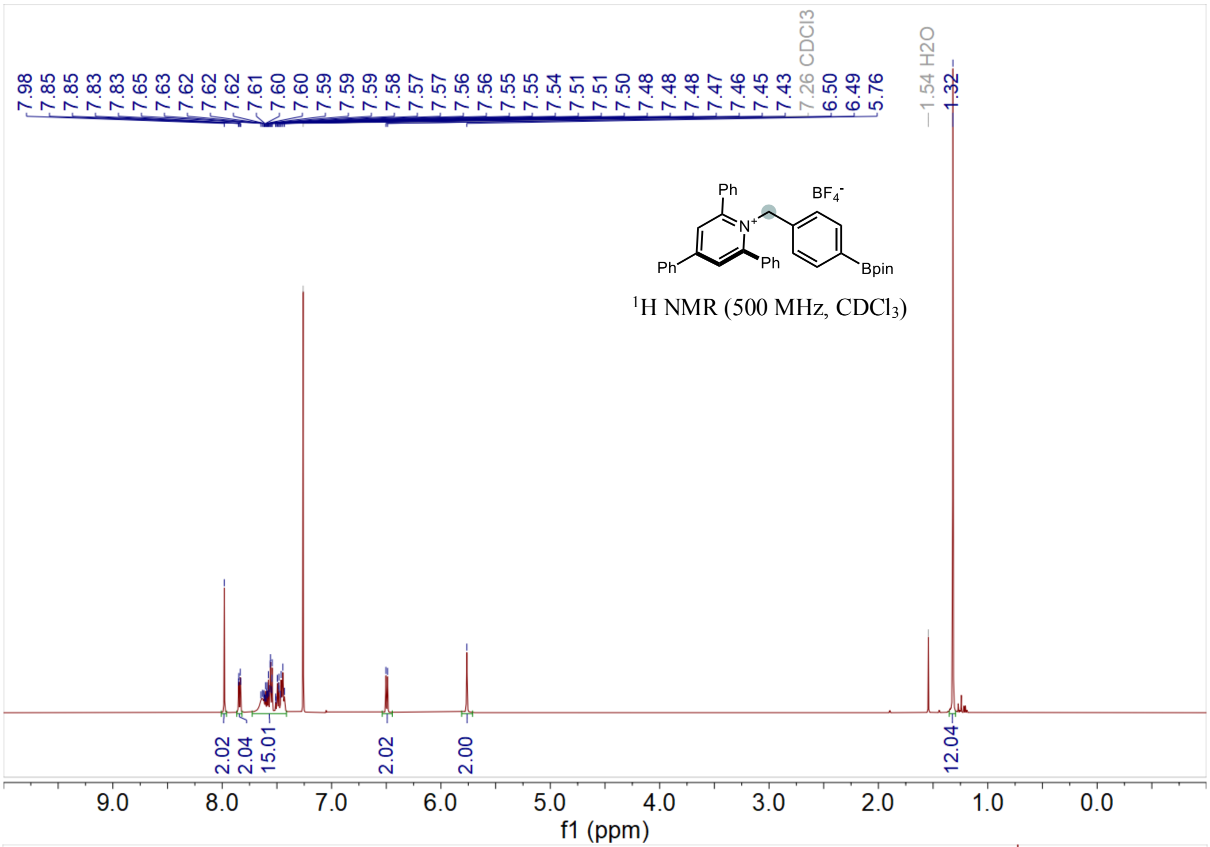
7.98 7.85 7.85 7.83 7.83 7.65 7.63 7.62 7.62 7.62 7.61 7.60 7.60 7.59 7.59 7.59 7.58 7.57 7.57 7.56 7.56 7.55 7.55 7.54 7.51 7.51 7.50 7.48 7.48 7.48 7.47 7.46 7.45 7.43 7.26 CDCl3 6.50 6.49 5.76
1.54 H2O
1.32
Ph
BF4-
N+
Ph
Ph
Bpin
1H NMR (500 MHz, CDCl3)
2.02 2.04 15.01 2.02 2.00 12.04
9.0 8.0 7.0 6.0 5.0 4.0 3.0 2.0 1.0 0.0
f1 (ppm)

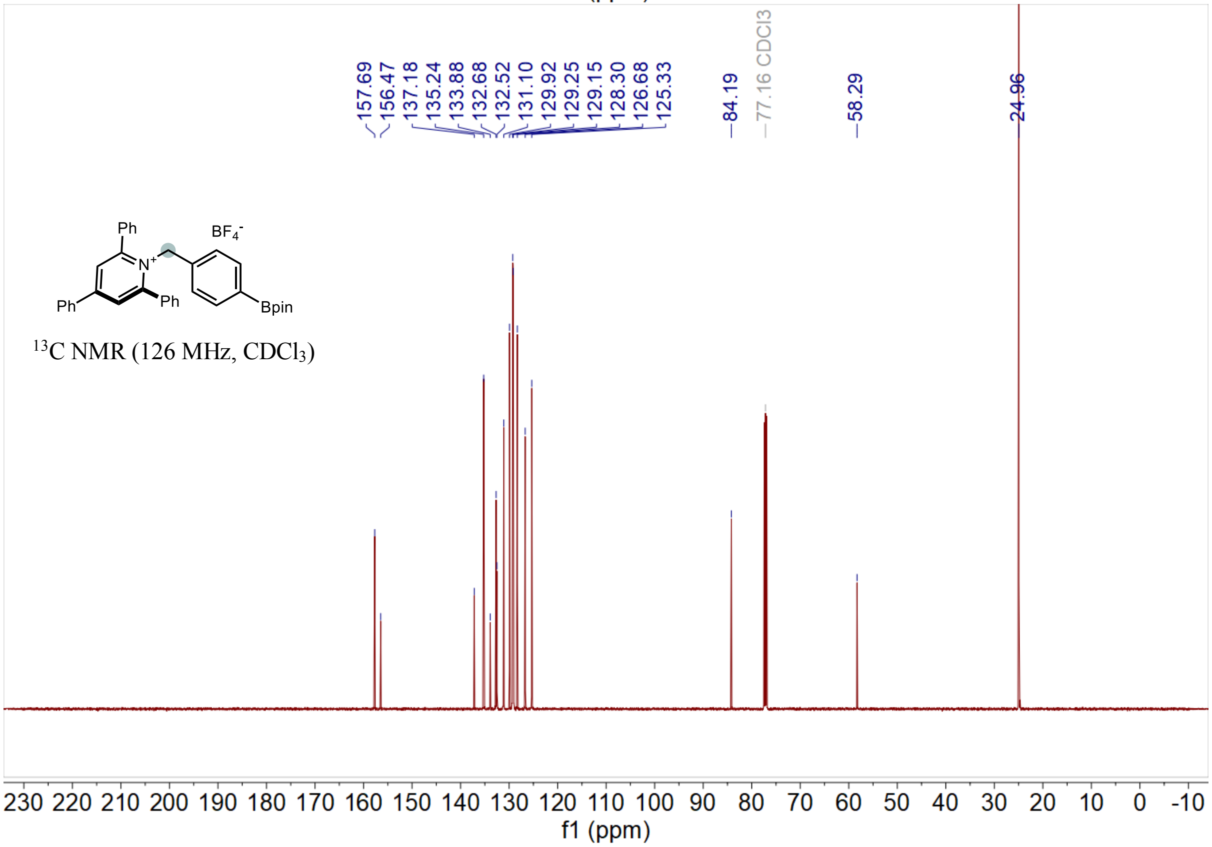
157.69 156.47 137.18 135.24 133.88 132.68 132.52 131.10 129.92 129.25 129.15 128.30 126.68 125.33
84.19
77.16 CDCl3
58.29
24.96
Ph
BF4-
N+
Ph
Ph
Bpin
13C NMR (126 MHz, CDCl3)
230 220 210 200 190 180 170 160 150 140 130 120 110 100 90 80 70 60 50 40 30 20 10 0 -10
f1 (ppm)

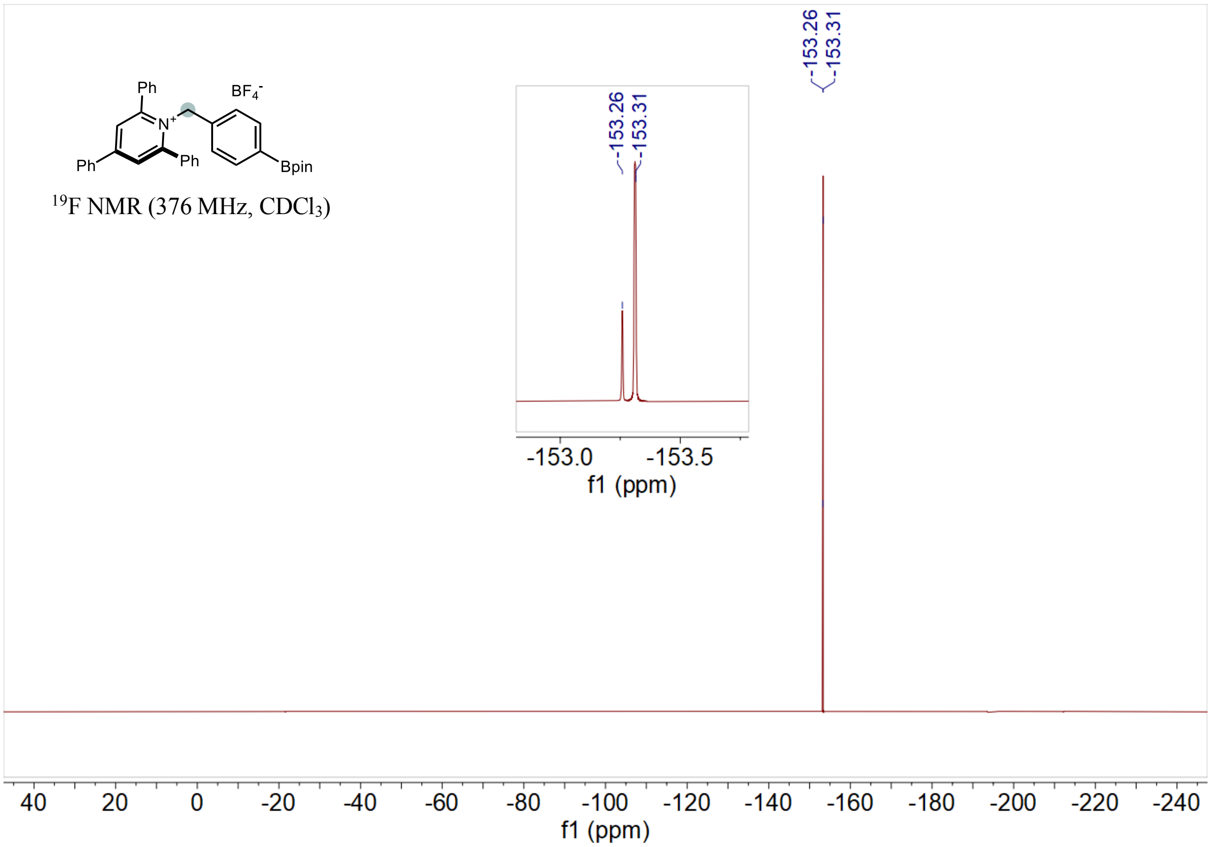
Ph
BF4-
N+
Ph
Ph
Bpin
19F NMR (376 MHz, CDCl3)
-153.26
-153.31
-153.0
-153.5
f1 (ppm)
40
20
0
-20
-40
-60
-80
-100
-120
-140
-160
-180
-200
-220
-240
f1 (ppm)

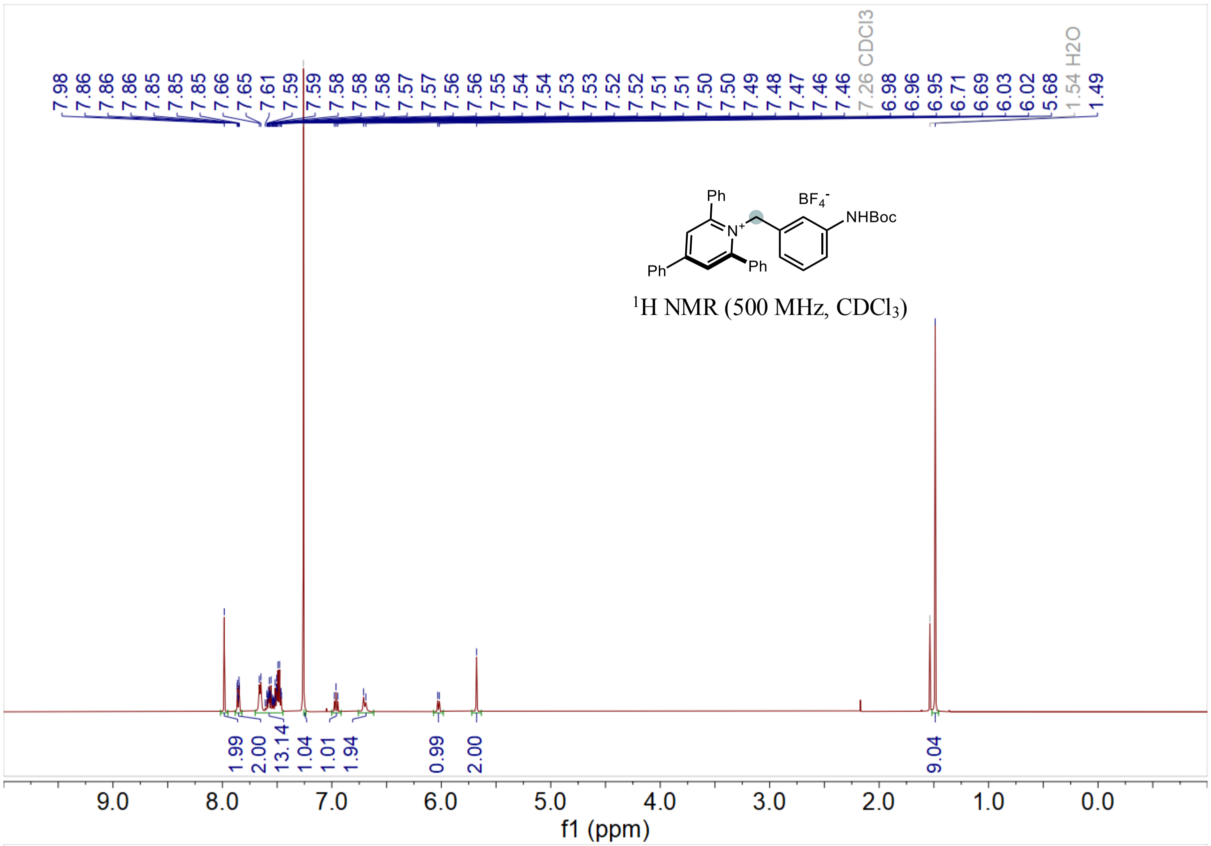

7.98
7.86
7.86
7.86
7.85
7.85
7.85
7.66
7.65
7.61
7.59
7.59
7.58
7.58
7.58
7.57
7.57
7.56
7.56
7.55
7.54
7.54
7.53
7.53
7.52
7.52
7.51
7.51
7.50
7.50
7.49
7.48
7.47
7.46
7.46
7.26 CDCl3
6.98
6.96
6.95
6.71
6.69
6.03
6.02
5.68
1.54 H2O
1.49
Ph
BF4-
NHBoc
N+
Ph
Ph
1H NMR (500 MHz, CDCl3)
1.99
2.00
13.14
1.04
1.01
1.94
0.99
2.00
9.04
9.0
8.0
7.0
6.0
5.0
4.0
3.0
2.0
1.0
0.0
f1 (ppm)

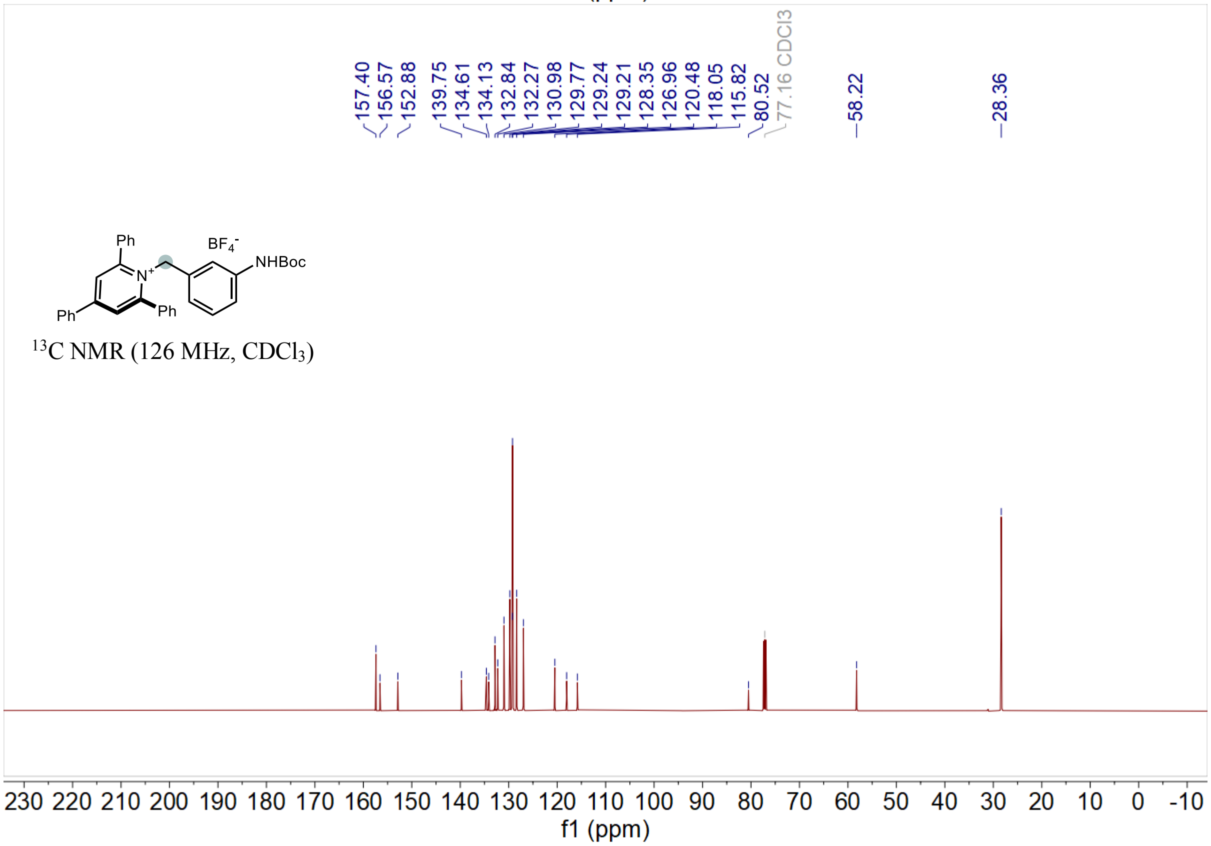

157.40
156.57
152.88
139.75
134.61
134.13
132.84
132.27
130.98
129.77
129.24
129.21
128.35
126.96
120.48
118.05
115.82
80.52
77.16 CDCl3
58.22
28.36
Ph
BF4-
NHBoc
N+
Ph
Ph
13C NMR (126 MHz, CDCl3)
230
220
210
200
190
180
170
160
150
140
130
120
110
100
90
80
70
60
50
40
30
20
10
0
-10
f1 (ppm)

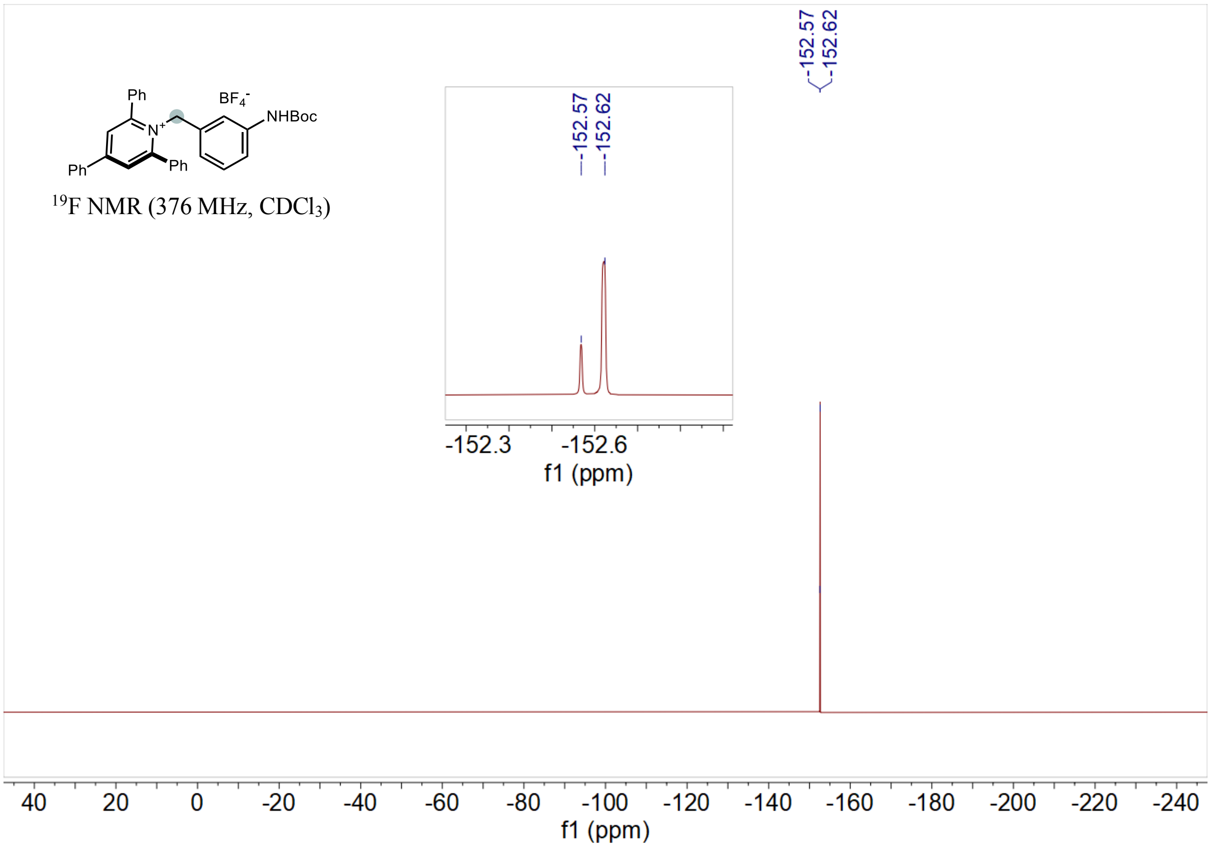
Ph
BF4-
NHBoc
N+
Ph
Ph
19F NMR (376 MHz, CDCl3)
-152.57
-152.62
-152.3
-152.6
f1 (ppm)
40
20
0
-20
-40
-60
-80
-100
-120
-140
-160
-180
-200
-220
-240
f1 (ppm)

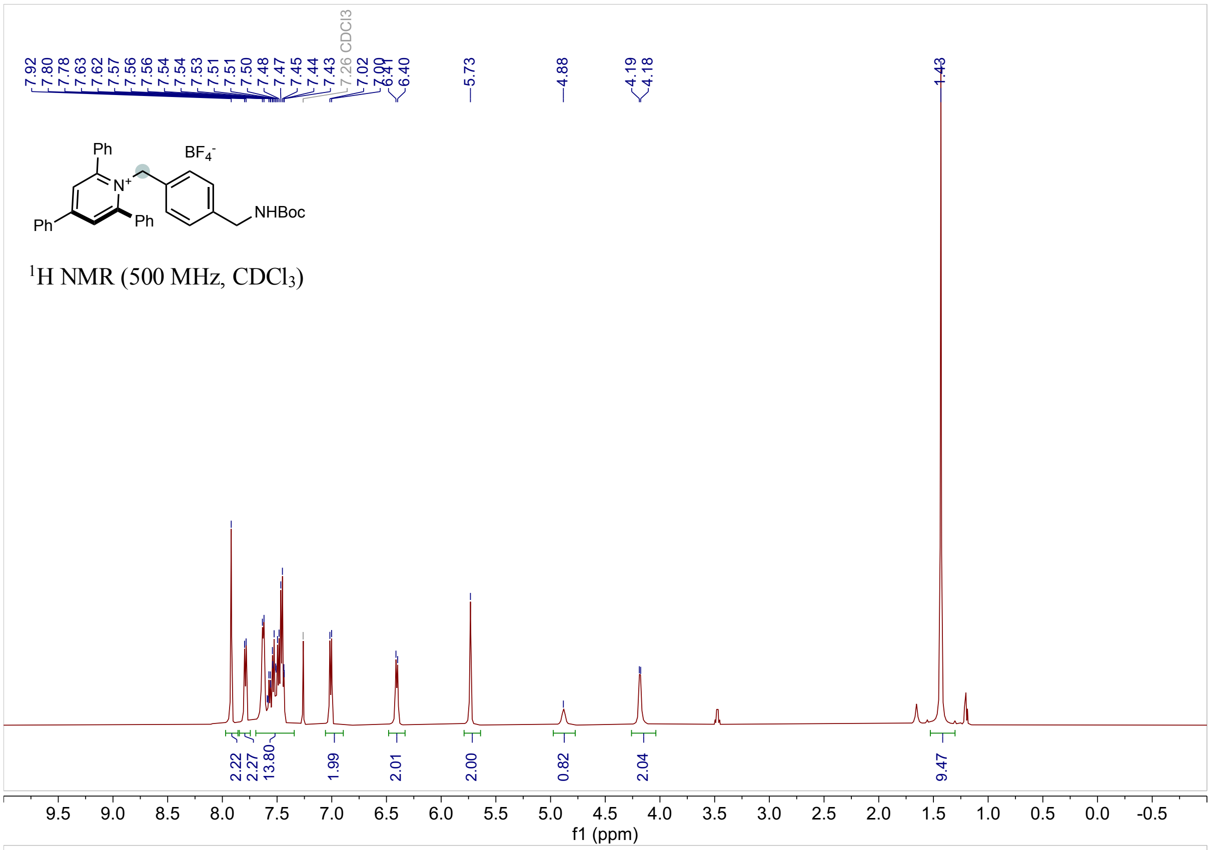

^{1}H NMR (500 MHz, $CDCl_3$)

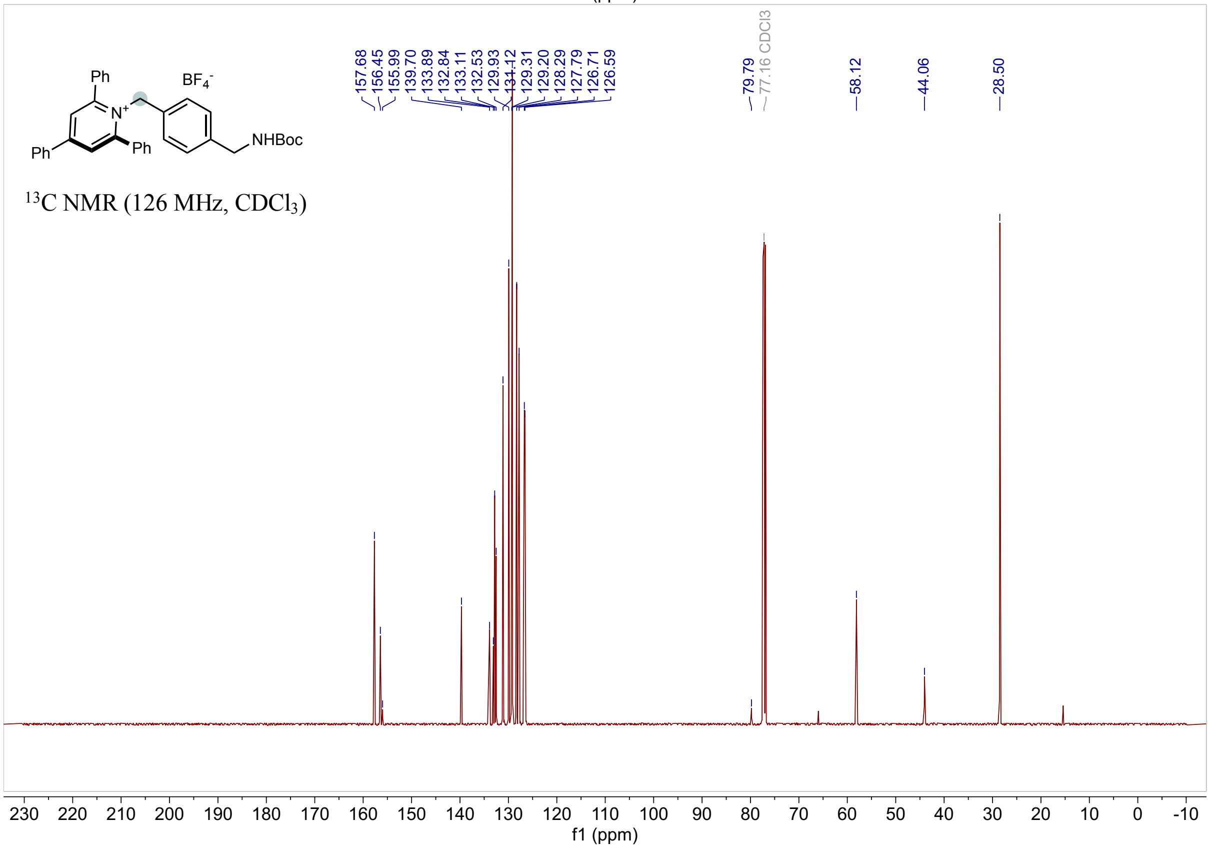

^{13}C NMR (126 MHz, $CDCl_3$)

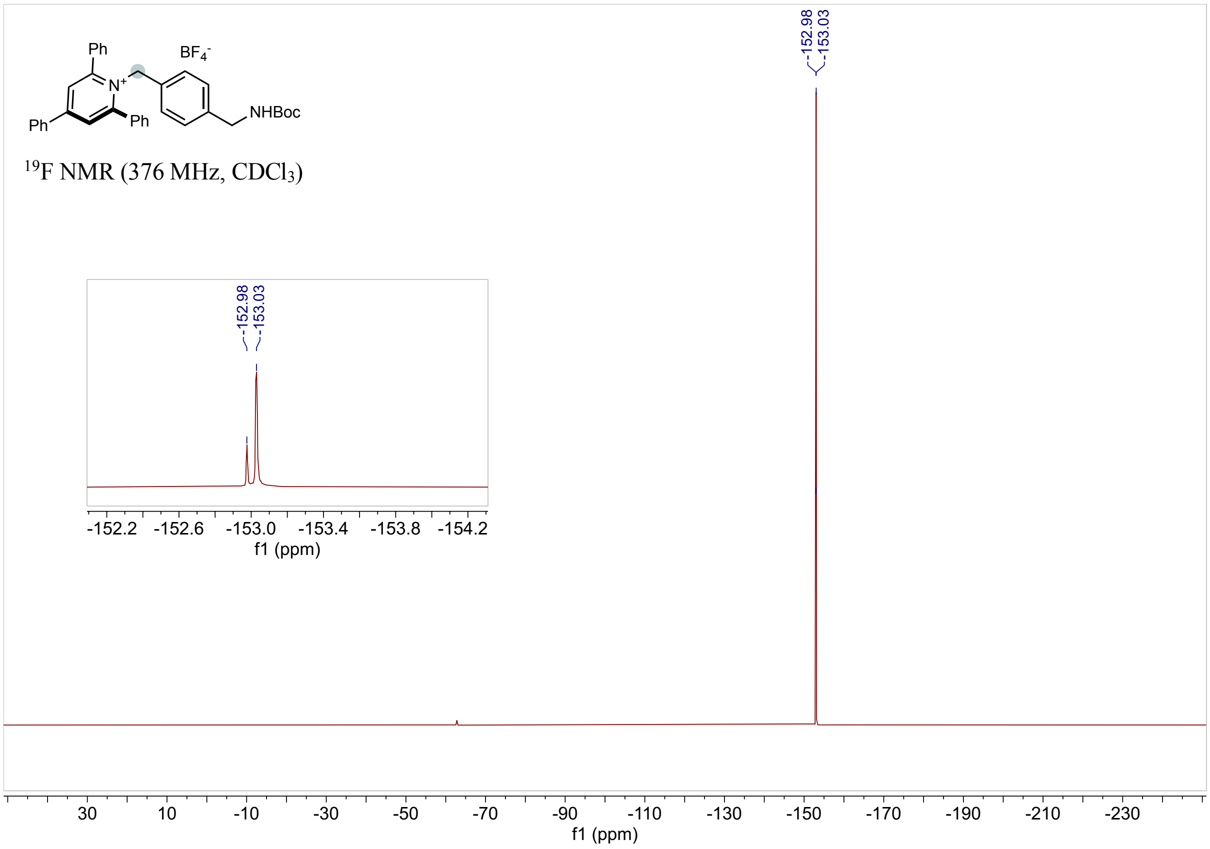

Ph
BF4-
N+
Ph
Ph
NHBoc
19F NMR (376 MHz, CDCl3)
-152.98
-153.03
-152.2 -152.6 -153.0 -153.4 -153.8 -154.2
f1 (ppm)
30 10 -10 -30 -50 -70 -90 -110 -130 -150 -170 -190 -210 -230
f1 (ppm)

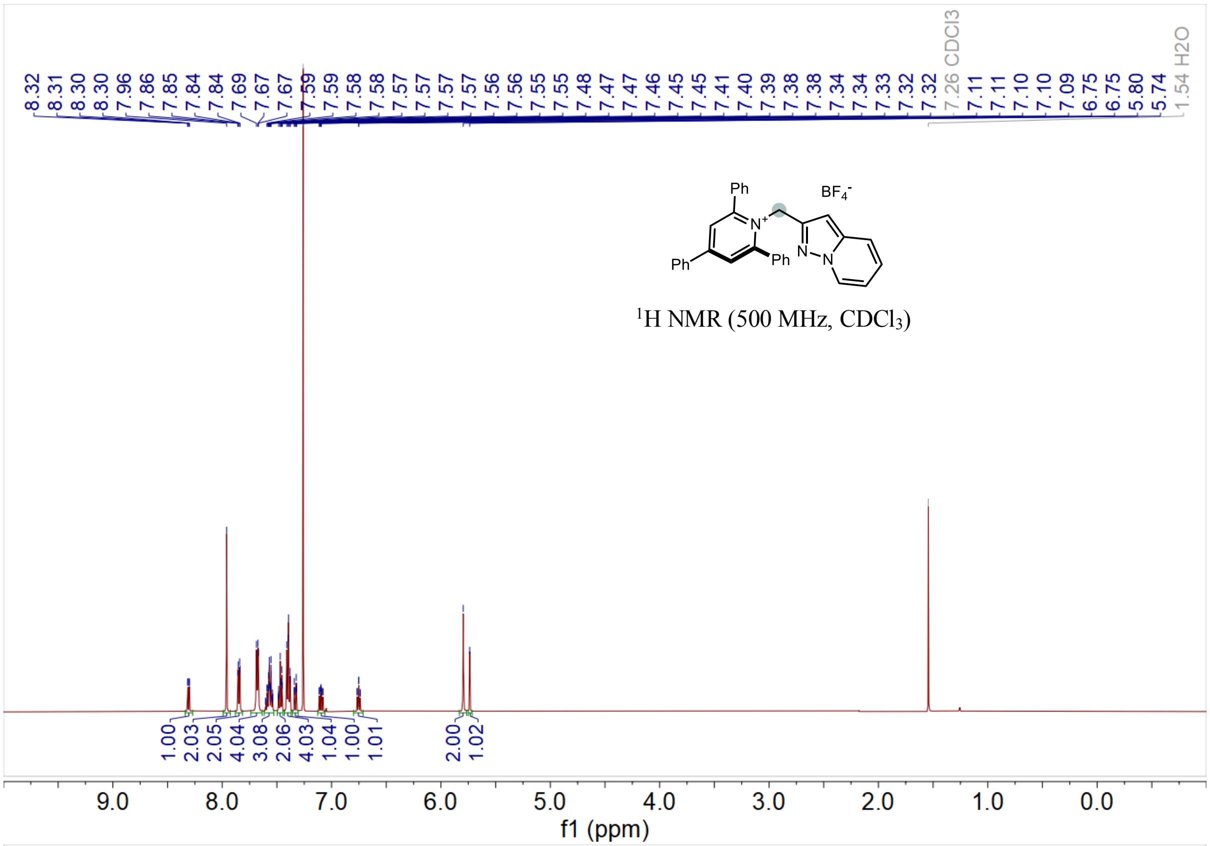
8.32 8.31 8.30 8.30 7.96 7.86 7.85 7.84 7.84 7.69 7.67 7.67 7.59 7.59 7.58 7.58 7.57 7.57 7.57 7.57 7.56 7.56 7.55 7.55 7.48 7.47 7.47 7.46 7.45 7.45 7.41 7.40 7.39 7.38 7.38 7.34 7.33 7.32 7.32 7.26 CDCl3 7.11 7.11 7.10 7.10 7.09 6.75 6.75 5.80 5.74 1.54 H2O
Ph
BF4-
N+
Ph
Ph
N
N
1H NMR (500 MHz, CDCl3)
1.00 2.03 2.05 4.04 3.08 2.06 4.03 1.04 1.00 1.01 2.00 1.02
9.0 8.0 7.0 6.0 5.0 4.0 3.0 2.0 1.0 0.0
f1 (ppm)

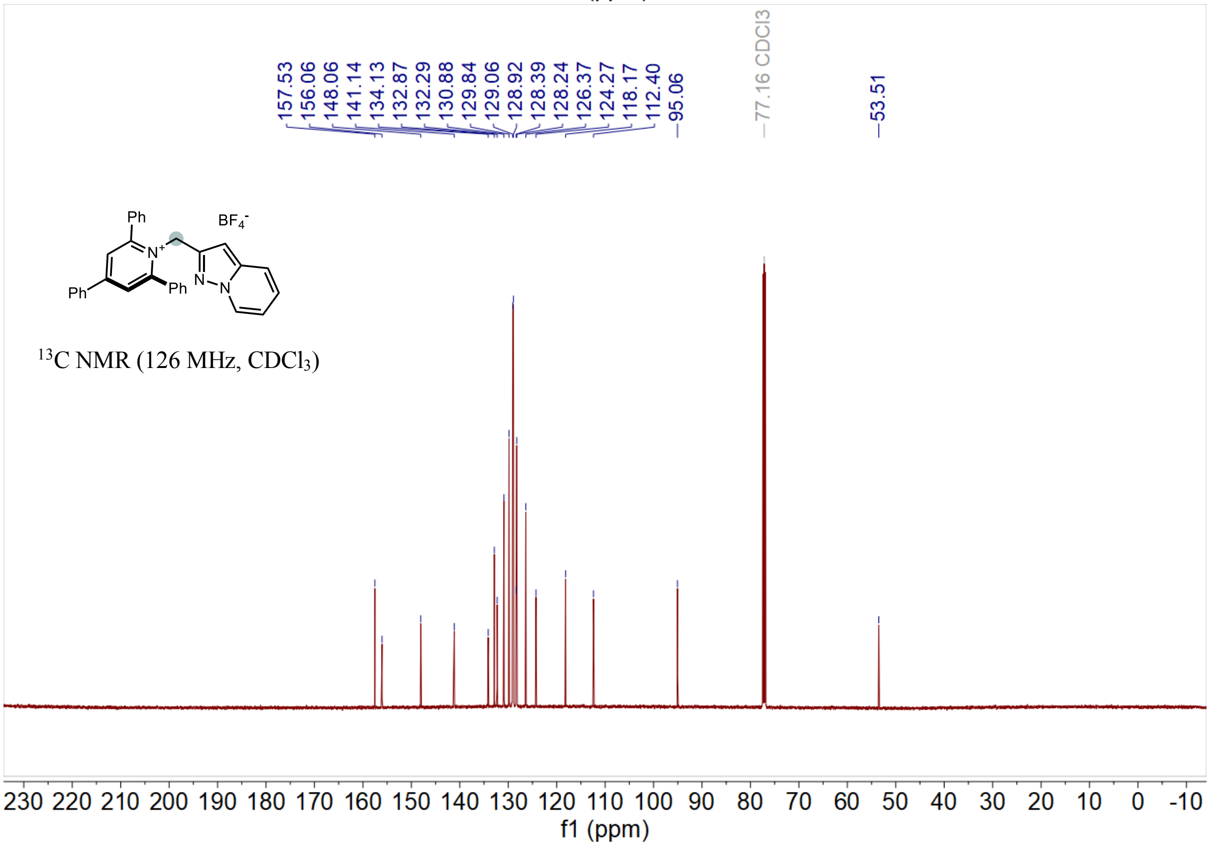
157.53 156.06 148.06 141.14 134.13 132.87 132.29 130.88 129.84 129.06 128.92 128.39 128.24 126.37 124.27 118.17 112.40 95.06 77.16 CDCl3 53.51
Ph
BF4-
N+
Ph
Ph
N
N
13C NMR (126 MHz, CDCl3)
230 220 210 200 190 180 170 160 150 140 130 120 110 100 90 80 70 60 50 40 30 20 10 0 -10
f1 (ppm)

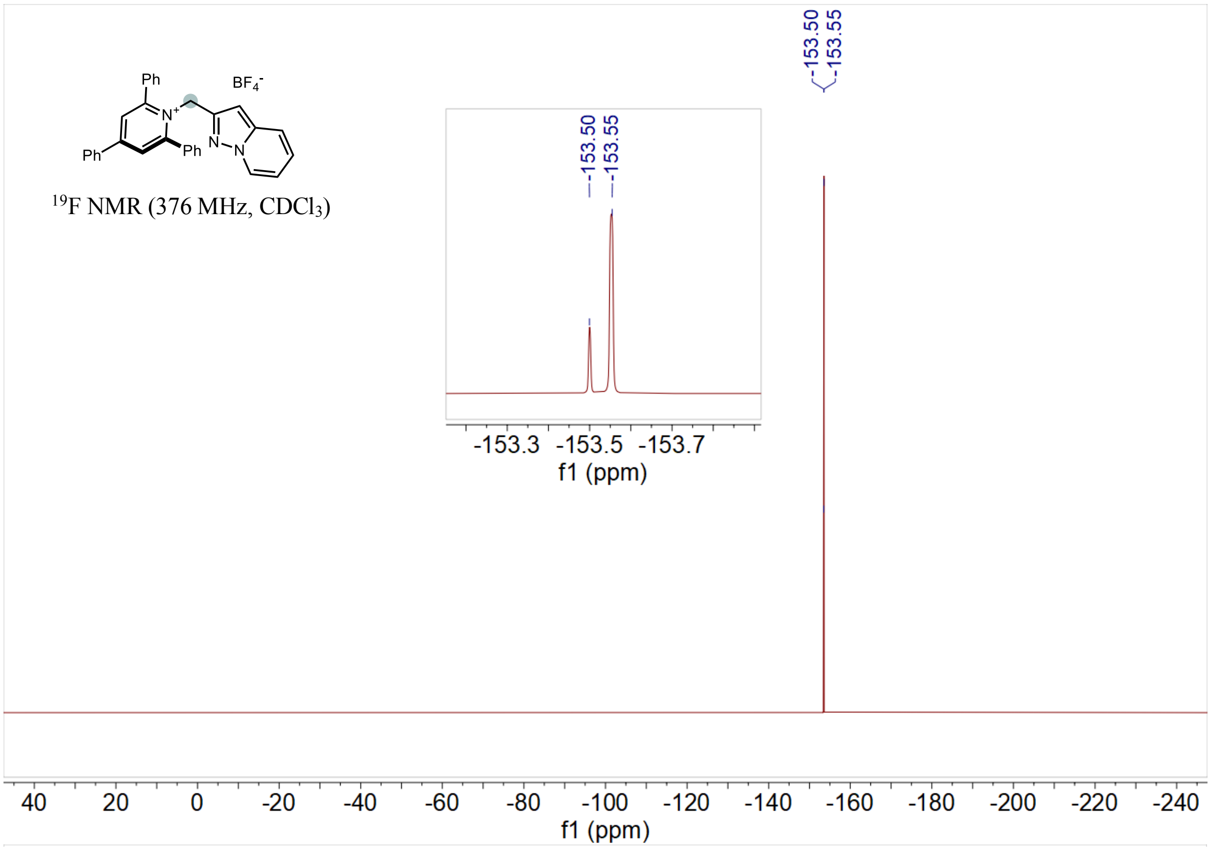
Ph
BF4-
N+
N
N
Ph
Ph
19F NMR (376 MHz, CDCl3)
-153.50
-153.55
-153.3 -153.5 -153.7
f1 (ppm)
40 20 0 -20 -40 -60 -80 -100 -120 -140 -160 -180 -200 -220 -240
f1 (ppm)

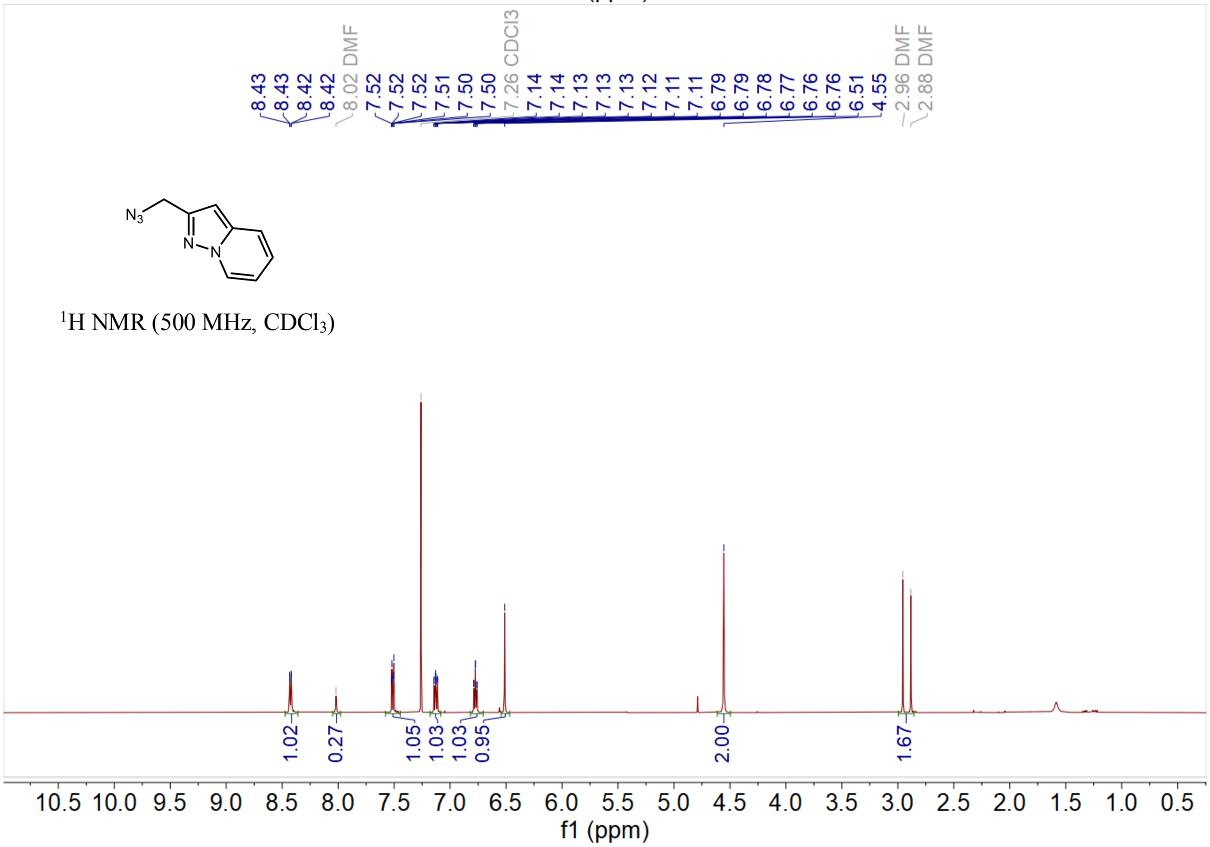
8.43
8.43
8.42
8.42
8.02 DMF
7.52
7.52
7.52
7.51
7.50
7.50
7.26 CDCl3
7.14
7.14
7.13
7.13
7.13
7.12
7.11
7.11
6.79
6.79
6.78
6.77
6.76
6.76
6.51
4.55
2.96 DMF
2.88 DMF
N3
N
N
1H NMR (500 MHz, CDCl3)
1.02
0.27
1.05
1.03
1.03
0.95
2.00
1.67
10.5 10.0 9.5 9.0 8.5 8.0 7.5 7.0 6.5 6.0 5.5 5.0 4.5 4.0 3.5 3.0 2.5 2.0 1.5 1.0 0.5
f1 (ppm)

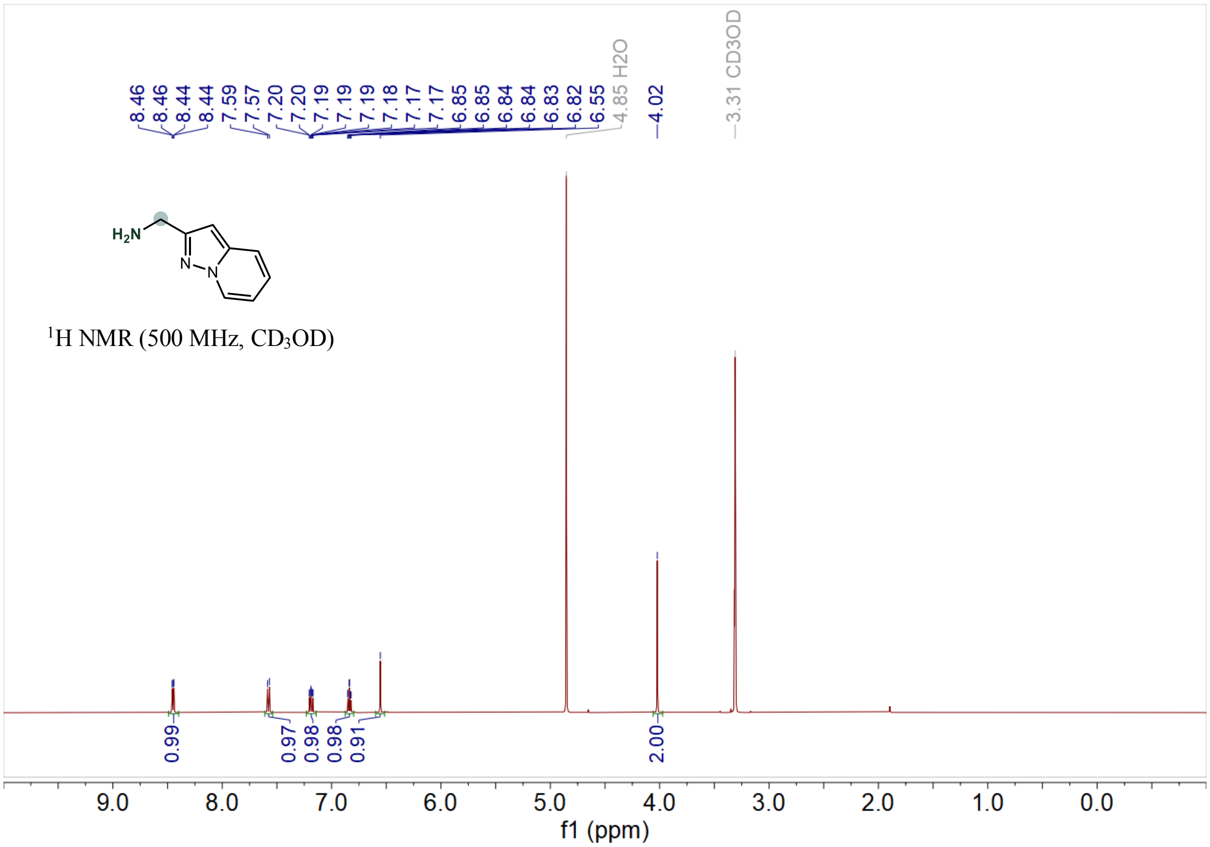

^{1}H NMR (500 MHz, CD_3OD)

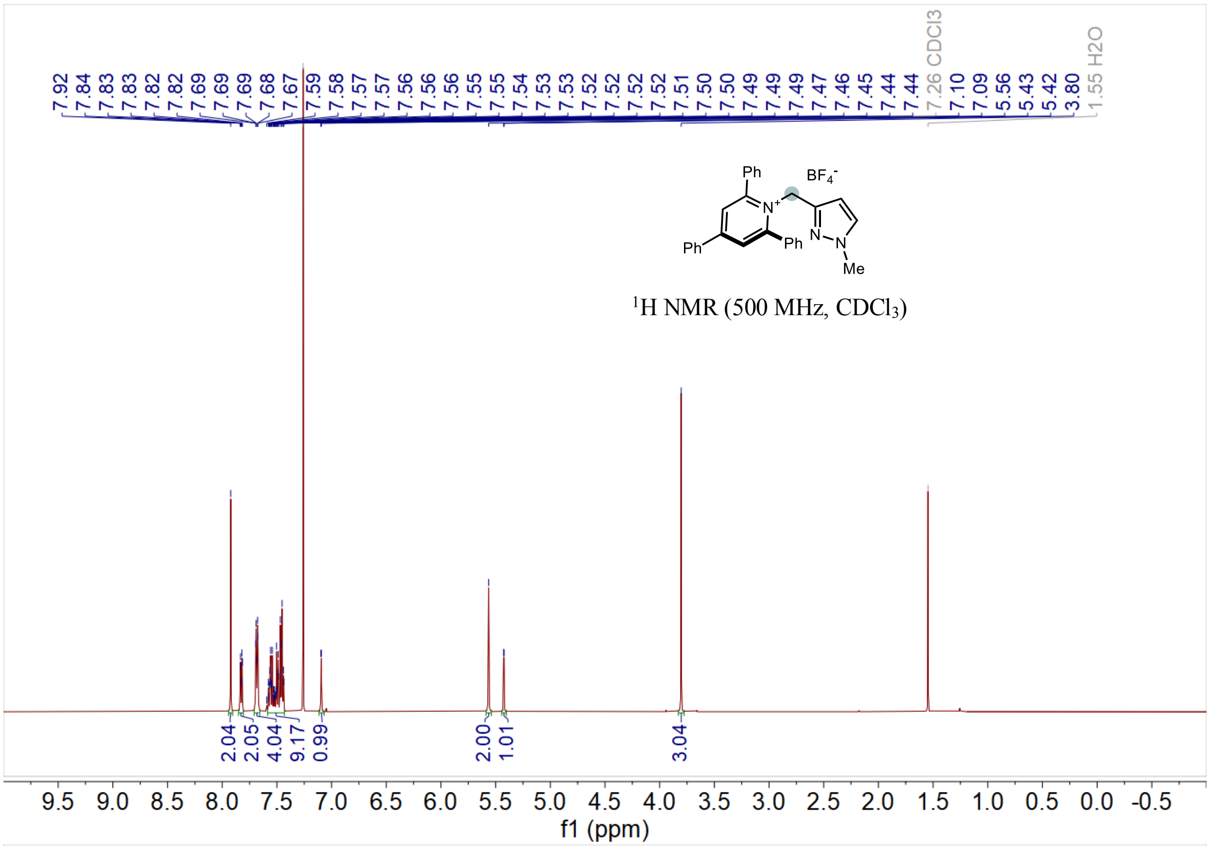

7.92
7.84
7.83
7.83
7.82
7.82
7.69
7.69
7.69
7.68
7.67
7.59
7.58
7.57
7.57
7.56
7.56
7.56
7.55
7.55
7.54
7.53
7.53
7.52
7.52
7.52
7.52
7.52
7.51
7.50
7.50
7.49
7.49
7.49
7.47
7.46
7.45
7.44
7.44
7.26 CDCl3
7.10
7.09
5.56
5.43
5.42
3.80
1.55 H2O
Ph
BF4-
N+
Ph
Ph
N
N
Me
1H NMR (500 MHz, CDCl3)
2.04
2.05
4.04
9.17
0.99
2.00
1.01
3.04
9.5 9.0 8.5 8.0 7.5 7.0 6.5 6.0 5.5 5.0 4.5 4.0 3.5 3.0 2.5 2.0 1.5 1.0 0.5 0.0 -0.5
f1 (ppm)

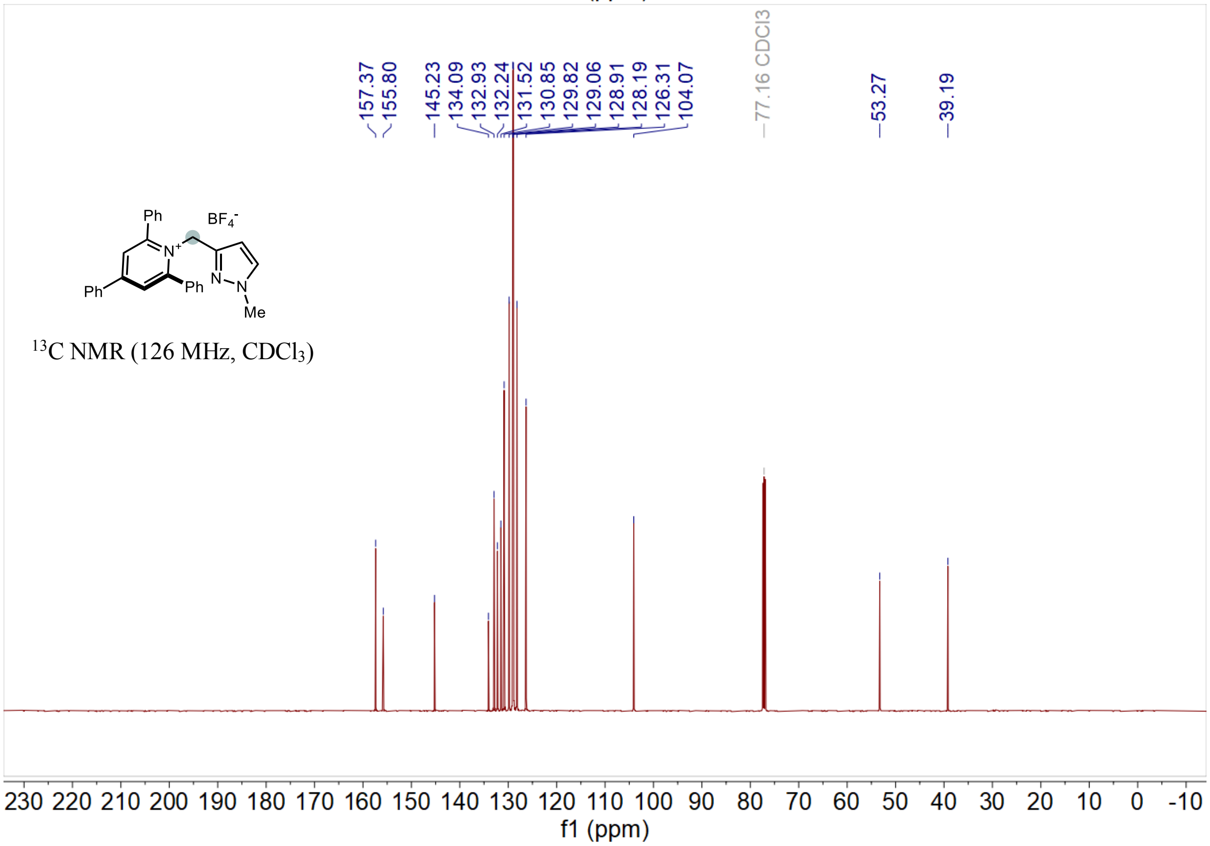

157.37
155.80
145.23
134.09
132.93
132.24
131.52
130.85
129.82
129.06
128.91
128.19
126.31
104.07
77.16 CDCl3
53.27
39.19
Ph
BF4-
N+
Ph
Ph
N
N
Me
13C NMR (126 MHz, CDCl3)
230 220 210 200 190 180 170 160 150 140 130 120 110 100 90 80 70 60 50 40 30 20 10 0 -10
f1 (ppm)

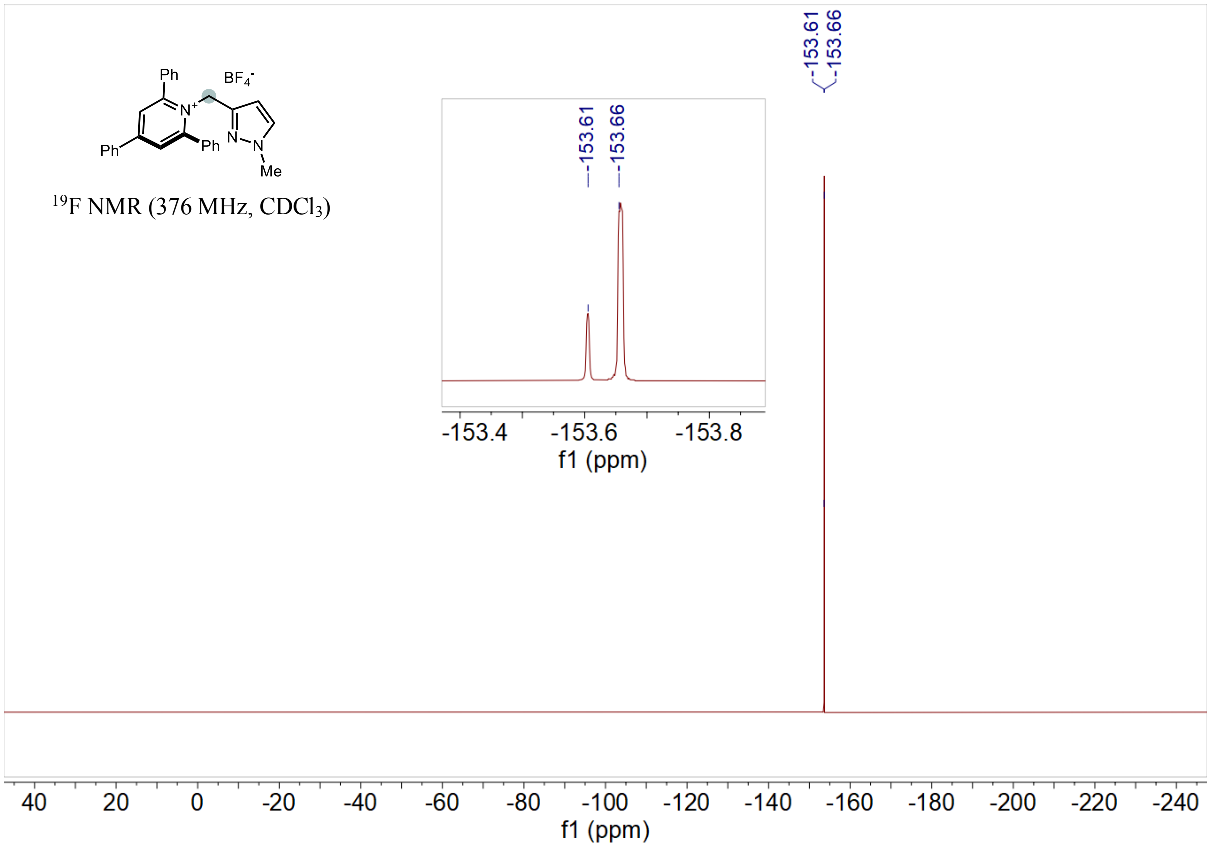

Ph
BF4-
N+
Ph
Ph
N
N
Me
19F NMR (376 MHz, CDCl3)
-153.61
-153.66
-153.4
-153.6
-153.8
f1 (ppm)
40
20
0
-20
-40
-60
-80
-100
-120
-140
-160
-180
-200
-220
-240
f1 (ppm)

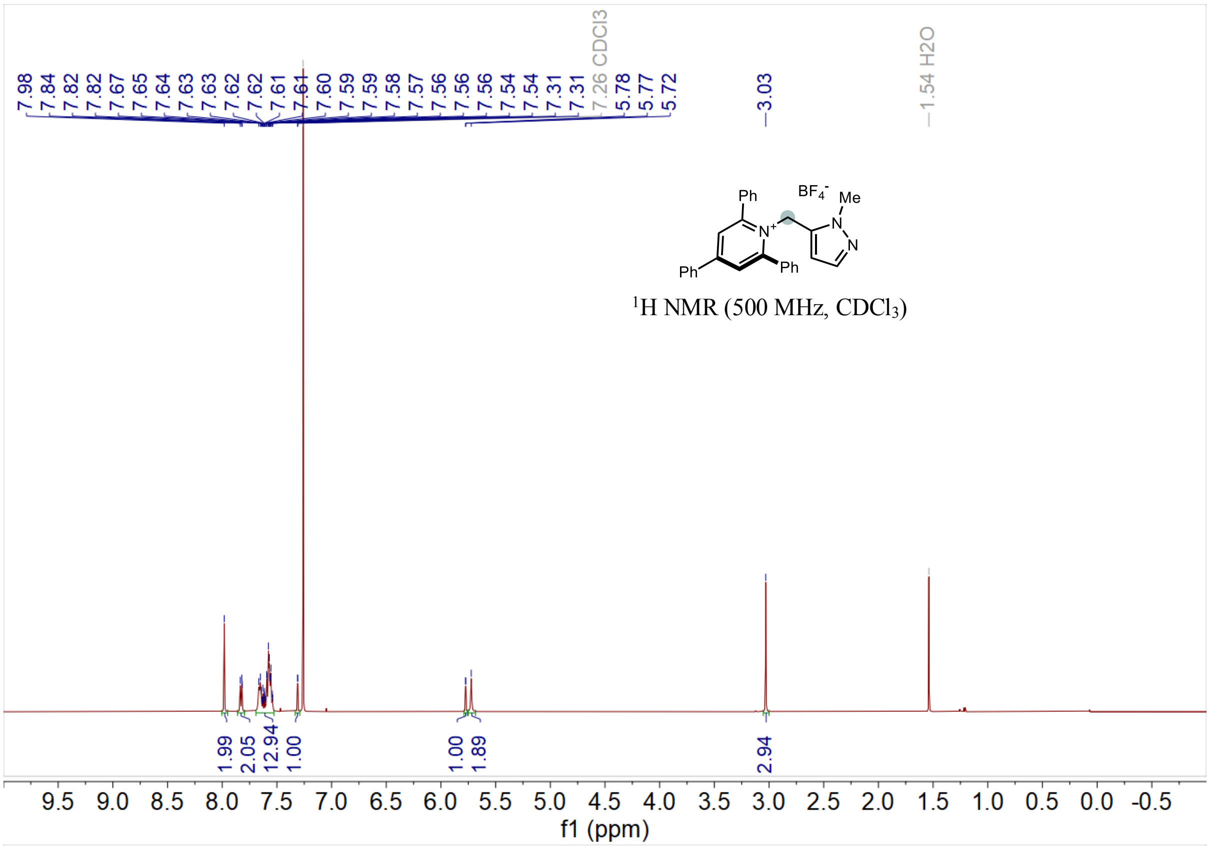
7.98
7.84
7.82
7.82
7.67
7.65
7.64
7.63
7.63
7.62
7.62
7.61
7.61
7.60
7.59
7.59
7.58
7.57
7.56
7.56
7.56
7.54
7.54
7.31
7.31
7.26 CDCl3
5.78
5.77
5.72
3.03
1.54 H2O
Ph
BF4-
Me
N+
N
N
Ph
Ph
1H NMR (500 MHz, CDCl3)
1.99
2.05
12.94
1.00
1.00
1.89
2.94
9.5 9.0 8.5 8.0 7.5 7.0 6.5 6.0 5.5 5.0 4.5 4.0 3.5 3.0 2.5 2.0 1.5 1.0 0.5 0.0 -0.5
f1 (ppm)

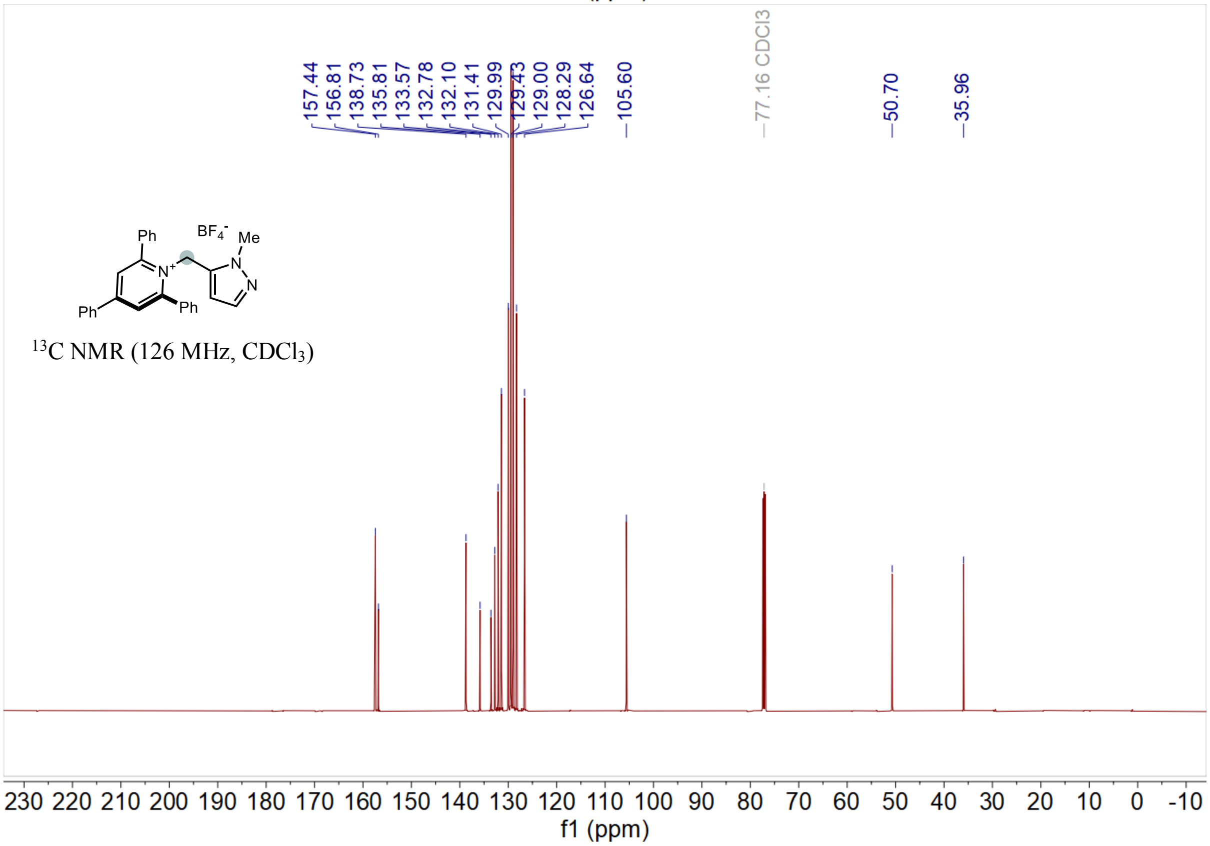
157.44
156.81
138.73
135.81
133.57
132.78
132.10
131.41
129.99
129.43
129.00
128.29
126.64
105.60
77.16 CDCl3
50.70
35.96
Ph
BF4-
Me
N+
N
N
Ph
Ph
13C NMR (126 MHz, CDCl3)
230 220 210 200 190 180 170 160 150 140 130 120 110 100 90 80 70 60 50 40 30 20 10 0 -10
f1 (ppm)

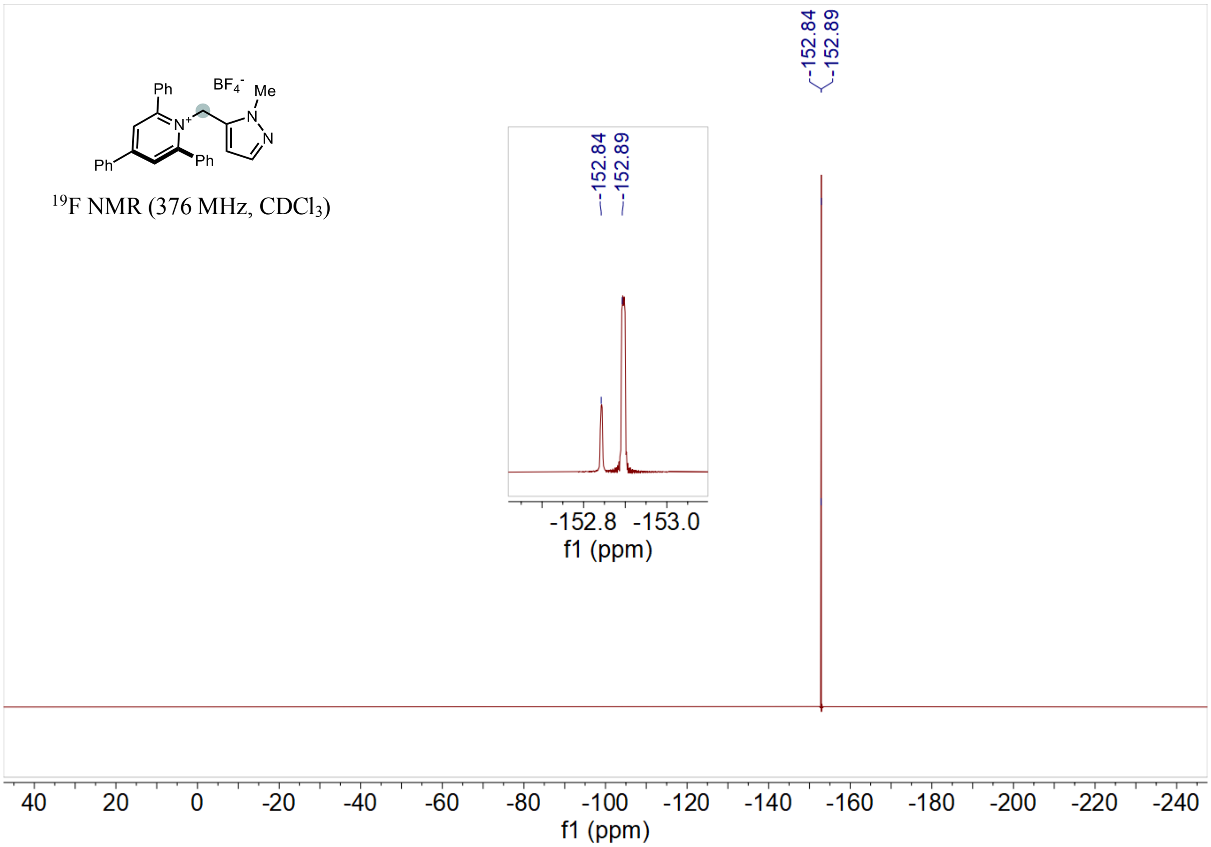

Ph
BF4-
Me
N+
N
N
Ph
Ph
19F NMR (376 MHz, CDCl3)
-152.84
-152.89
-152.8
-153.0
f1 (ppm)
40
20
0
-20
-40
-60
-80
-100
-120
-140
-160
-180
-200
-220
-240
f1 (ppm)

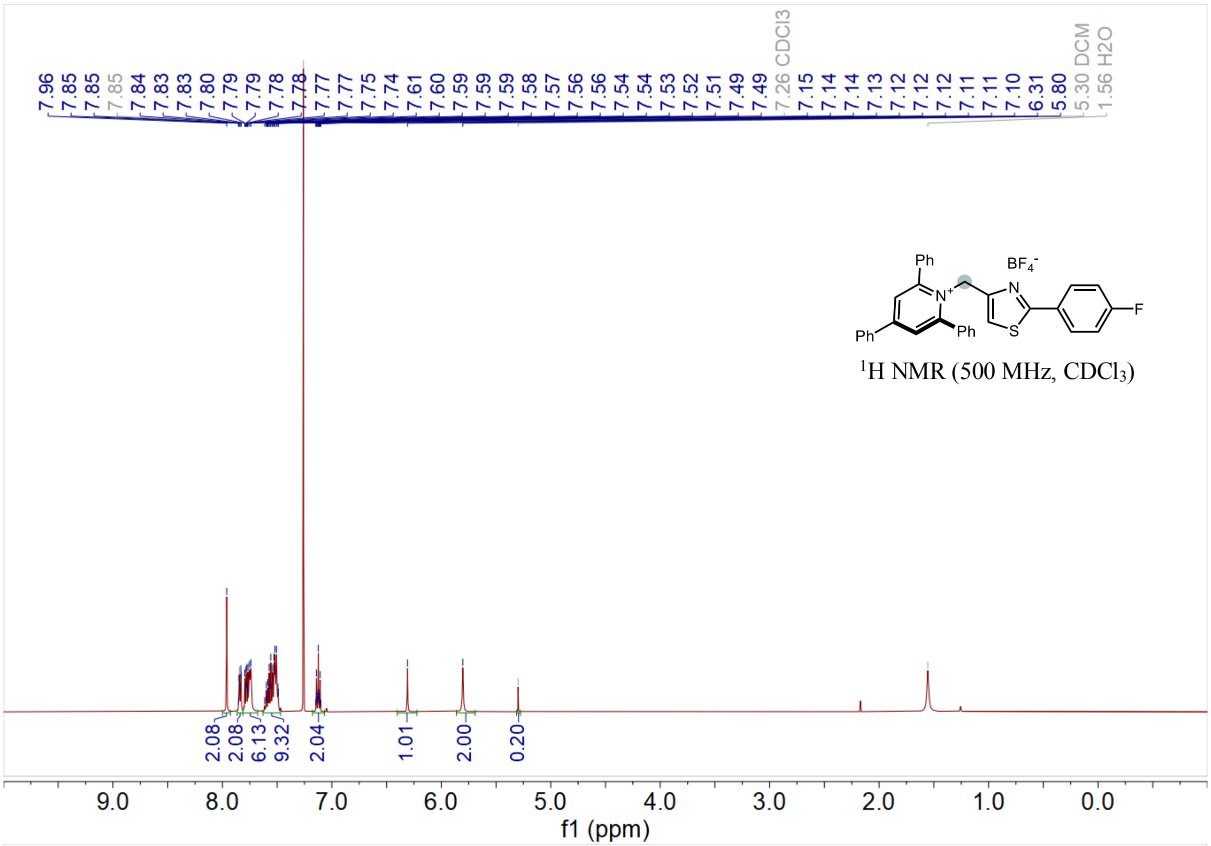
7.96
7.85
7.85
7.85
7.84
7.83
7.83
7.80
7.79
7.79
7.78
7.78
7.77
7.77
7.75
7.74
7.61
7.60
7.59
7.59
7.59
7.58
7.57
7.56
7.56
7.54
7.54
7.53
7.52
7.51
7.49
7.49
7.26 CDCl3
7.15
7.14
7.14
7.13
7.12
7.12
7.11
7.11
7.10
6.31
5.80
5.30 DCM
1.56 H2O
Ph
BF4-
N+
N
S
F
Ph
Ph
1H NMR (500 MHz, CDCl3)
2.08
2.08
6.13
9.32
2.04
1.01
2.00
0.20
9.0
8.0
7.0
6.0
5.0
4.0
3.0
2.0
1.0
0.0
f1 (ppm)

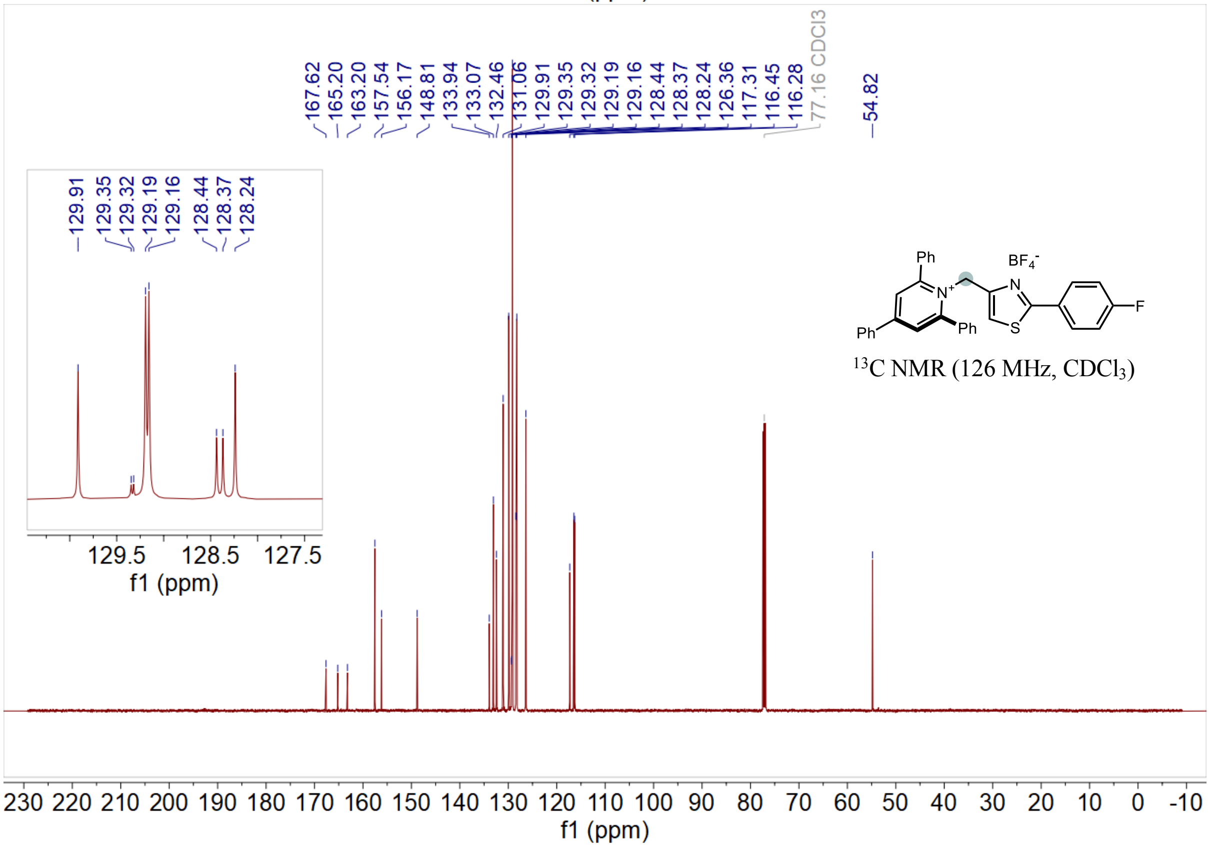
167.62
165.20
163.20
157.54
156.17
148.81
133.94
133.07
132.46
131.06
129.91
129.35
129.32
129.19
129.16
128.44
128.37
128.24
126.36
117.31
116.45
116.28
77.16 CDCl3
54.82
129.91
129.35
129.32
129.19
129.16
128.44
128.37
128.24
129.5
128.5
127.5
f1 (ppm)
Ph
BF4-
N+
N
S
F
Ph
Ph
13C NMR (126 MHz, CDCl3)
230
220
210
200
190
180
170
160
150
140
130
120
110
100
90
80
70
60
50
40
30
20
10
0
-10
f1 (ppm)

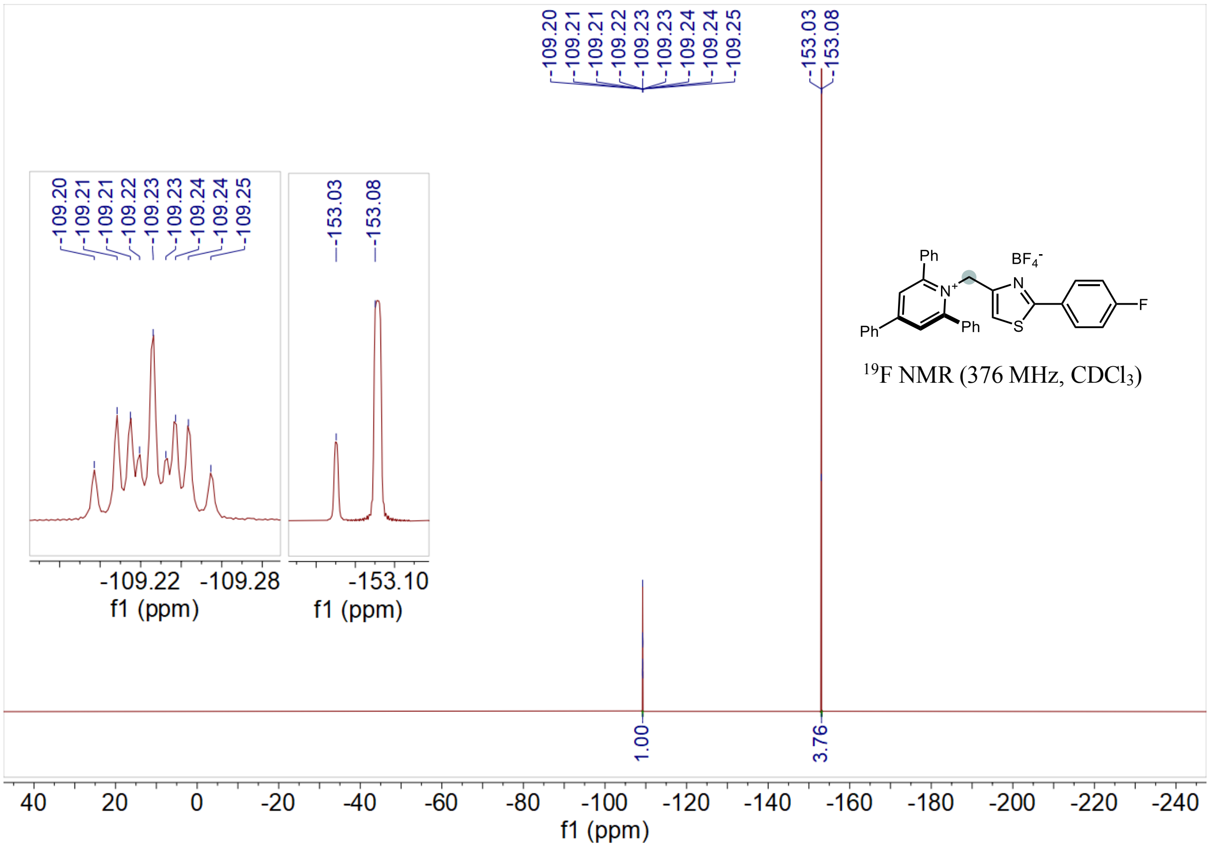

-109.20
-109.21
-109.21
-109.22
-109.23
-109.23
-109.24
-109.24
-109.25
-153.03
-153.08
-109.22 -109.28
f1 (ppm)
-153.10
f1 (ppm)
Ph
BF4-
N+
N
F
Ph
Ph
S
19F NMR (376 MHz, CDCl3)
1.00
3.76
40 20 0 -20 -40 -60 -80 -100 -120 -140 -160 -180 -200 -220 -240
f1 (ppm)

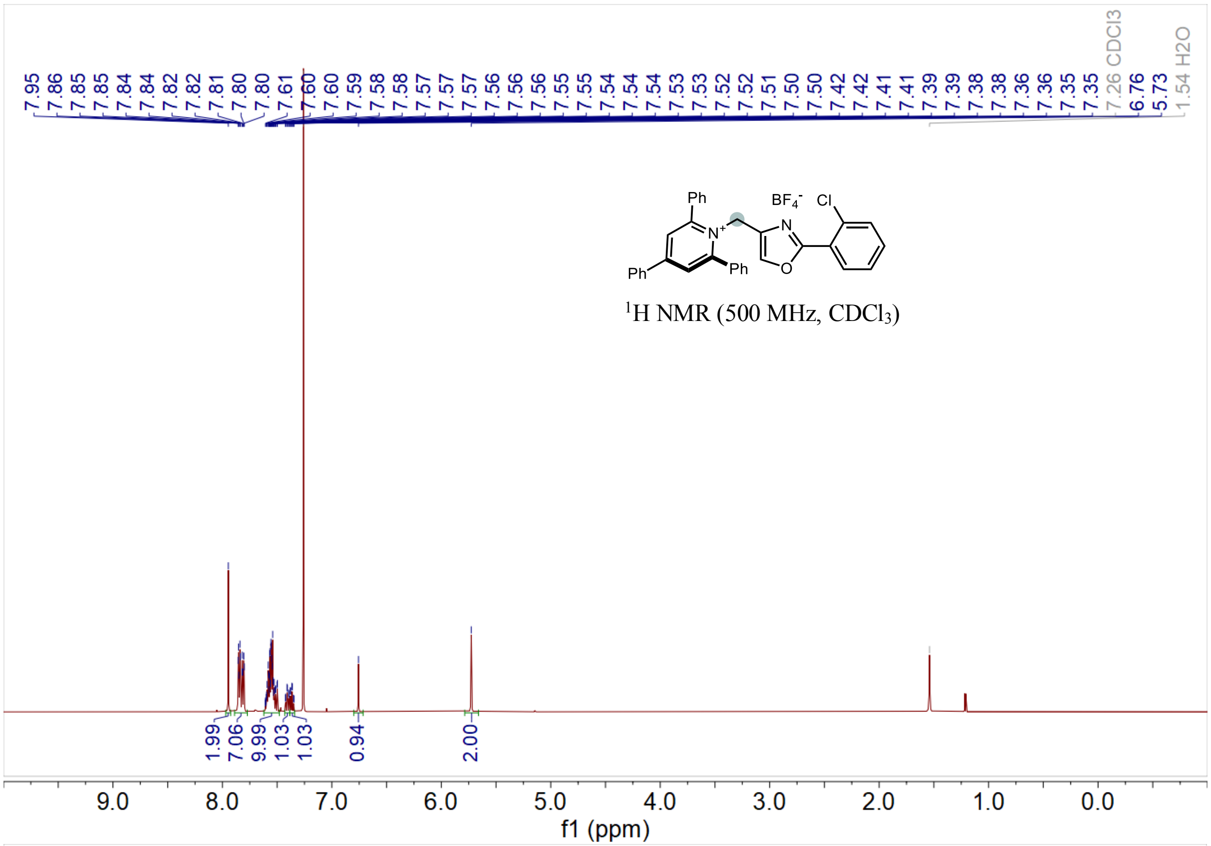
7.95
7.86
7.85
7.85
7.84
7.84
7.82
7.82
7.81
7.80
7.80
7.61
7.60
7.60
7.59
7.58
7.58
7.57
7.57
7.57
7.56
7.56
7.56
7.55
7.55
7.54
7.54
7.54
7.53
7.53
7.52
7.52
7.51
7.50
7.50
7.42
7.42
7.41
7.41
7.39
7.39
7.38
7.38
7.36
7.36
7.35
7.35
7.26 CDCl3
6.76
5.73
1.54 H2O
Ph
BF4-
Cl
N+
N
O
Ph
Ph
1H NMR (500 MHz, CDCl3)
1.99
7.06
9.99
1.03
1.03
0.94
2.00
9.0
8.0
7.0
6.0
5.0
4.0
3.0
2.0
1.0
0.0
f1 (ppm)

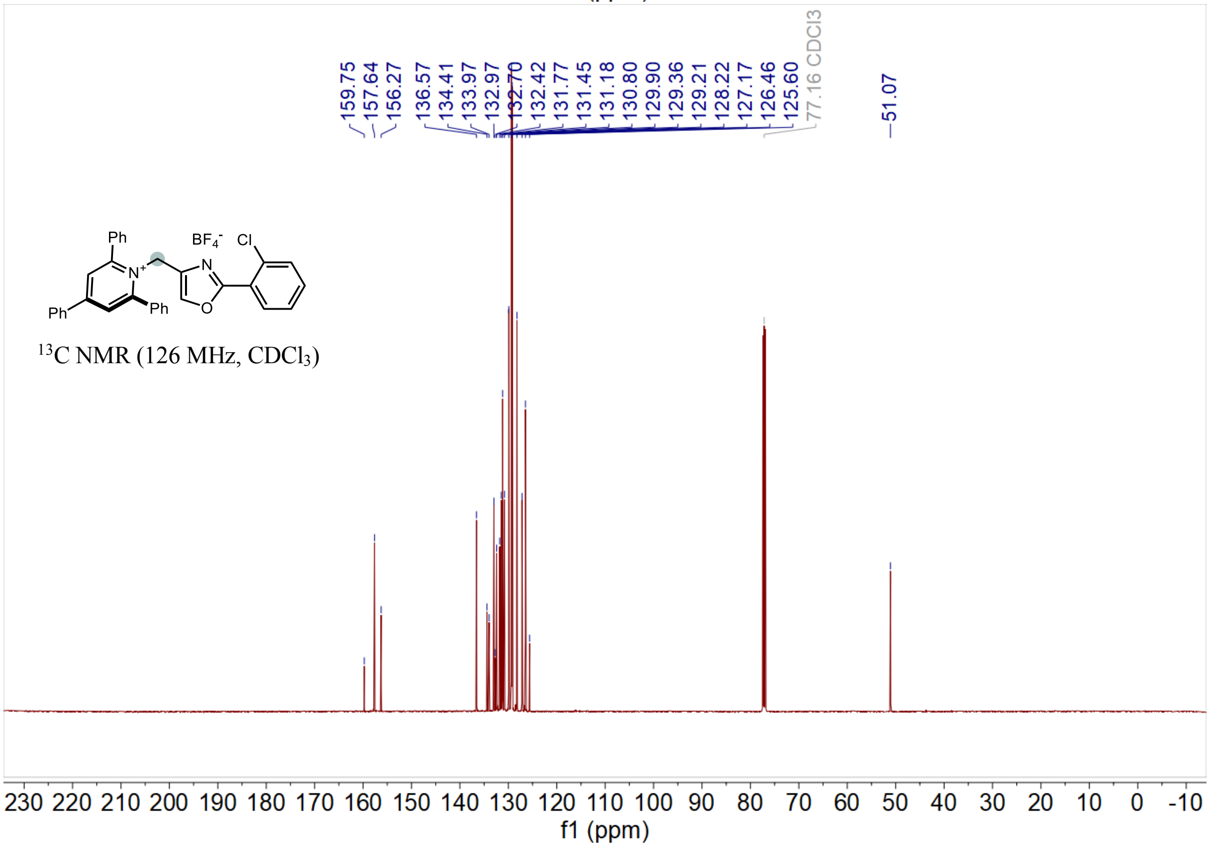
159.75
157.64
156.27
136.57
134.41
133.97
132.97
132.70
132.42
131.77
131.45
131.18
130.80
129.90
129.36
129.21
128.22
127.17
126.46
125.60
77.16 CDCl3
51.07
Ph
BF4-
Cl
N+
N
O
Ph
Ph
13C NMR (126 MHz, CDCl3)
230 220 210 200 190 180 170 160 150 140 130 120 110 100 90 80 70 60 50 40 30 20 10 0 -10
f1 (ppm)

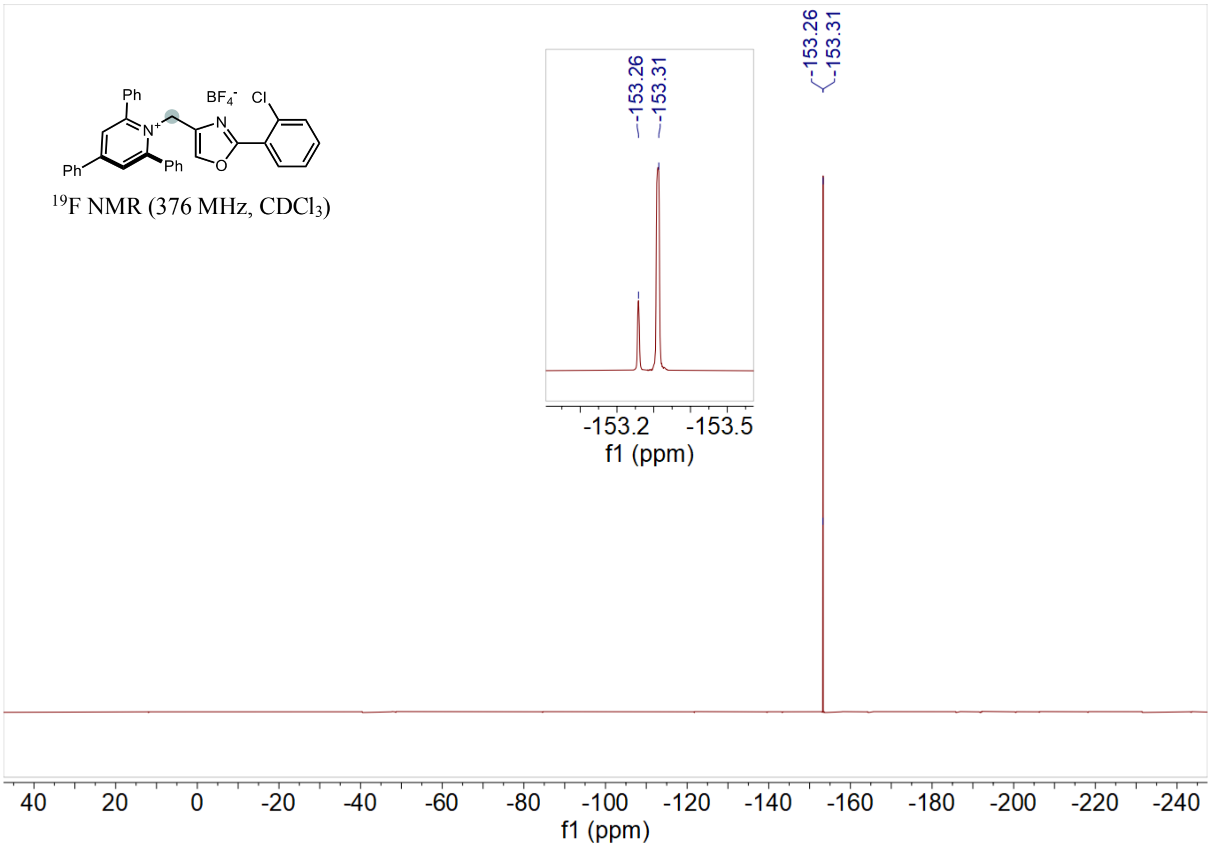

Ph
BF4-
Cl
N+
N
O
Ph
Ph
19F NMR (376 MHz, CDCl3)
-153.26
-153.31
-153.2
-153.5
f1 (ppm)
40
20
0
-20
-40
-60
-80
-100
-120
-140
-160
-180
-200
-220
-240
f1 (ppm)

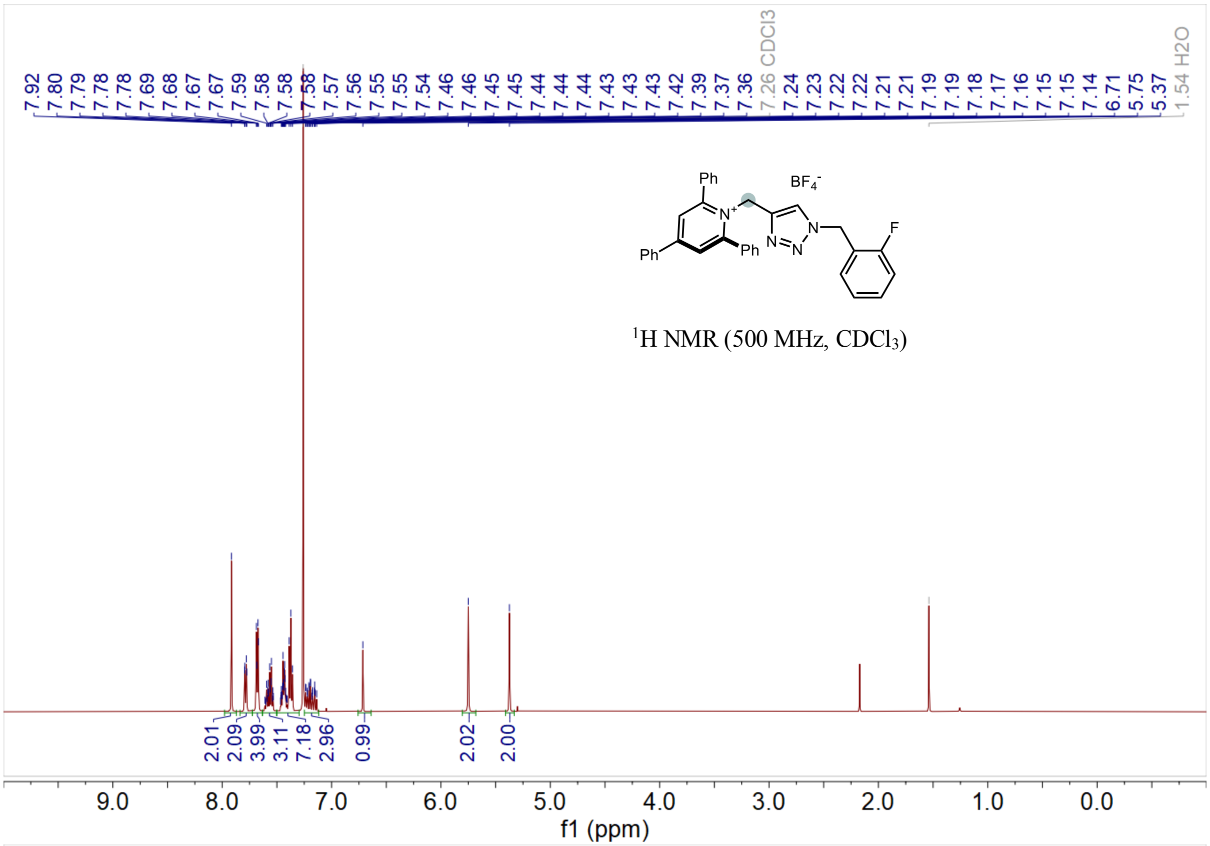

7.92 7.80 7.79 7.78 7.78 7.69 7.68 7.67 7.67 7.59 7.58 7.58 7.58 7.57 7.56 7.55 7.55 7.54 7.46 7.46 7.45 7.45 7.44 7.44 7.44 7.43 7.43 7.43 7.42 7.39 7.37 7.36 7.26 CDCl3 7.24 7.23 7.22 7.22 7.21 7.21 7.19 7.19 7.18 7.17 7.16 7.15 7.15 7.14 6.71 5.75 5.37 1.54 H2O
Ph
BF4-
N+
N
N=N
Ph
Ph
F
1H NMR (500 MHz, CDCl3)
2.01 2.09 3.99 3.11 7.18 2.96 0.99 2.02 2.00
9.0 8.0 7.0 6.0 5.0 4.0 3.0 2.0 1.0 0.0
f1 (ppm)

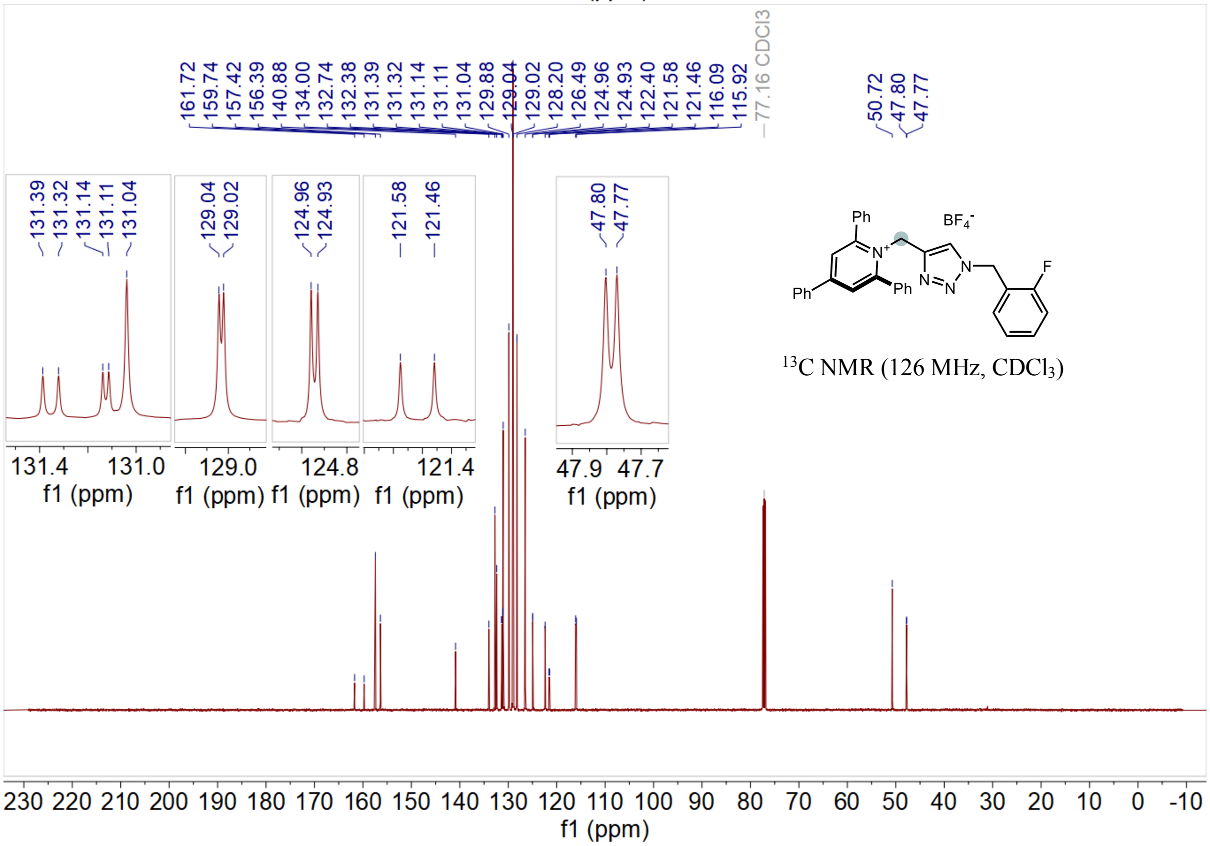

161.72 159.74 157.42 156.39 140.88 134.00 132.74 132.38 131.39 131.32 131.14 131.11 131.04 129.88 129.04 129.02 128.20 126.49 124.96 124.93 122.40 121.58 121.46 116.09 115.92 77.16 CDCl3
50.72 47.80 47.77
131.39 131.32 131.14 131.11 131.04
129.04 129.02
124.96 124.93
121.58 121.46
47.80 47.77
131.4 131.0
f1 (ppm)
129.0
f1 (ppm)
124.8
f1 (ppm)
121.4
f1 (ppm)
47.9 47.7
f1 (ppm)
Ph
BF4-
N+
N
N=N
Ph
Ph
F
13C NMR (126 MHz, CDCl3)
230 220 210 200 190 180 170 160 150 140 130 120 110 100 90 80 70 60 50 40 30 20 10 0 -10
f1 (ppm)

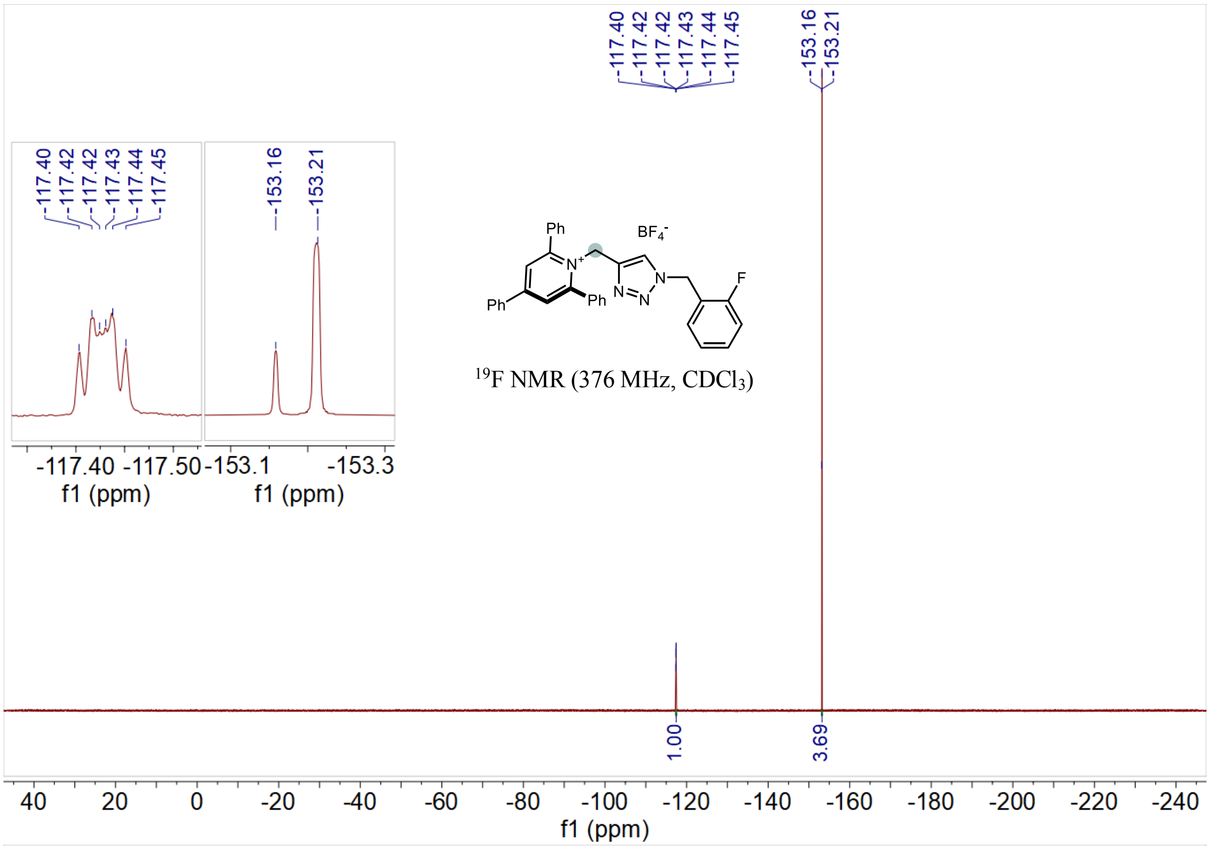

-117.40
-117.42
-117.42
-117.43
-117.44
-117.45
-153.16
-153.21
-117.40 -117.50
f1 (ppm)
-153.1 -153.3
f1 (ppm)
Ph
BF4-
N+
N
N
N
F
Ph
Ph
19F NMR (376 MHz, CDCl3)
1.00
3.69
40 20 0 -20 -40 -60 -80 -100 -120 -140 -160 -180 -200 -220 -240
f1 (ppm)

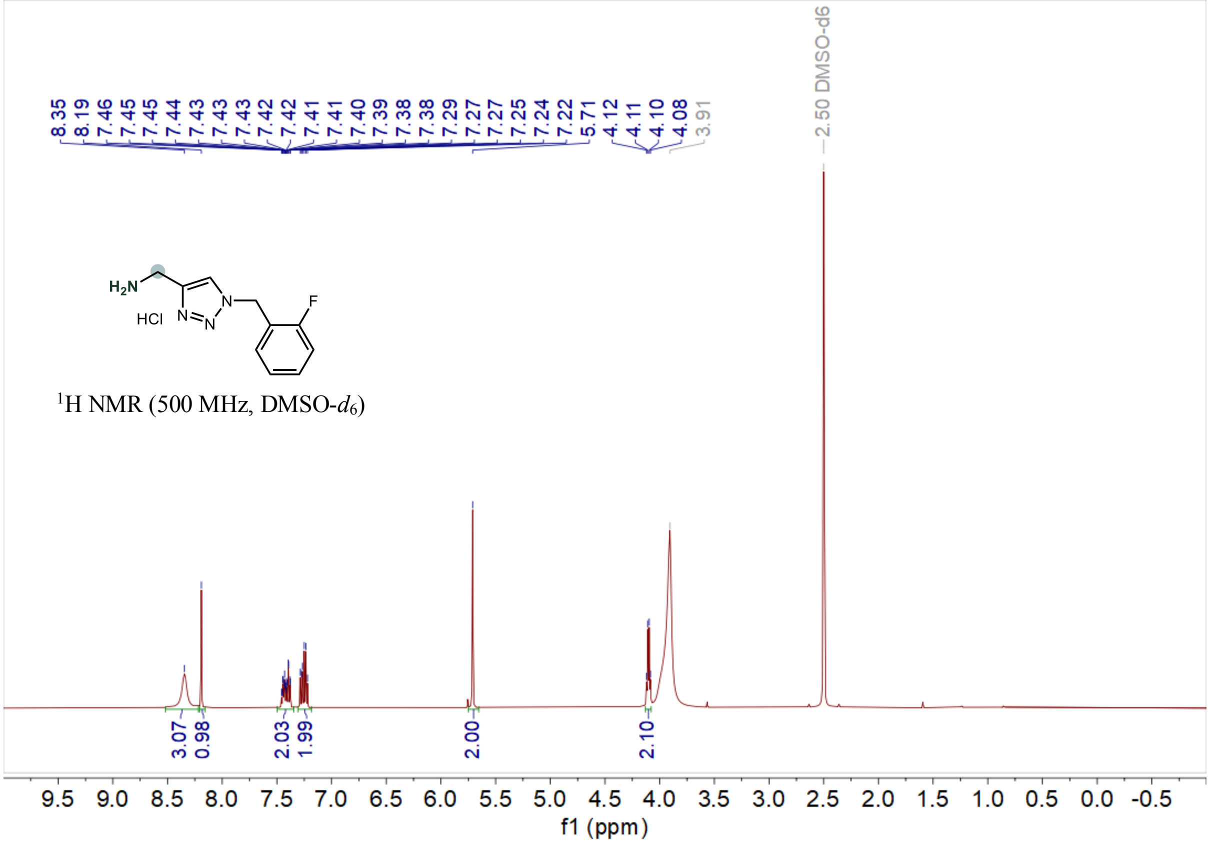

8.35
8.19
7.46
7.45
7.45
7.44
7.43
7.43
7.43
7.42
7.42
7.41
7.41
7.40
7.39
7.38
7.38
7.29
7.27
7.27
7.25
7.24
7.22
5.71
4.12
4.11
4.10
4.08
3.91
2.50 DMSO-d6
H2N
HCl
N
N
N
F
1H NMR (500 MHz, DMSO-d6)
3.07
0.98
2.03
1.99
2.00
2.10
9.5 9.0 8.5 8.0 7.5 7.0 6.5 6.0 5.5 5.0 4.5 4.0 3.5 3.0 2.5 2.0 1.5 1.0 0.5 0.0 -0.5
f1 (ppm)

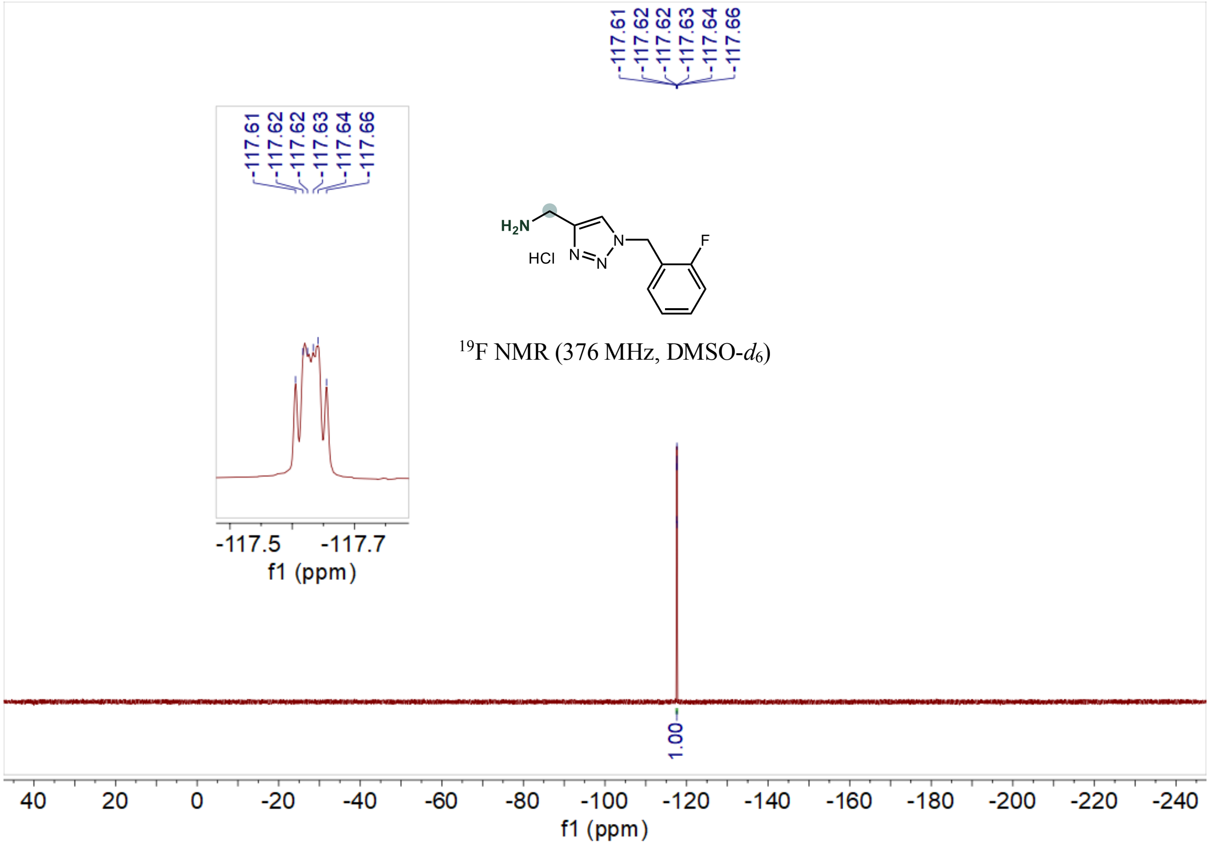

^{19}F NMR (376 MHz, DMSO-d_6)

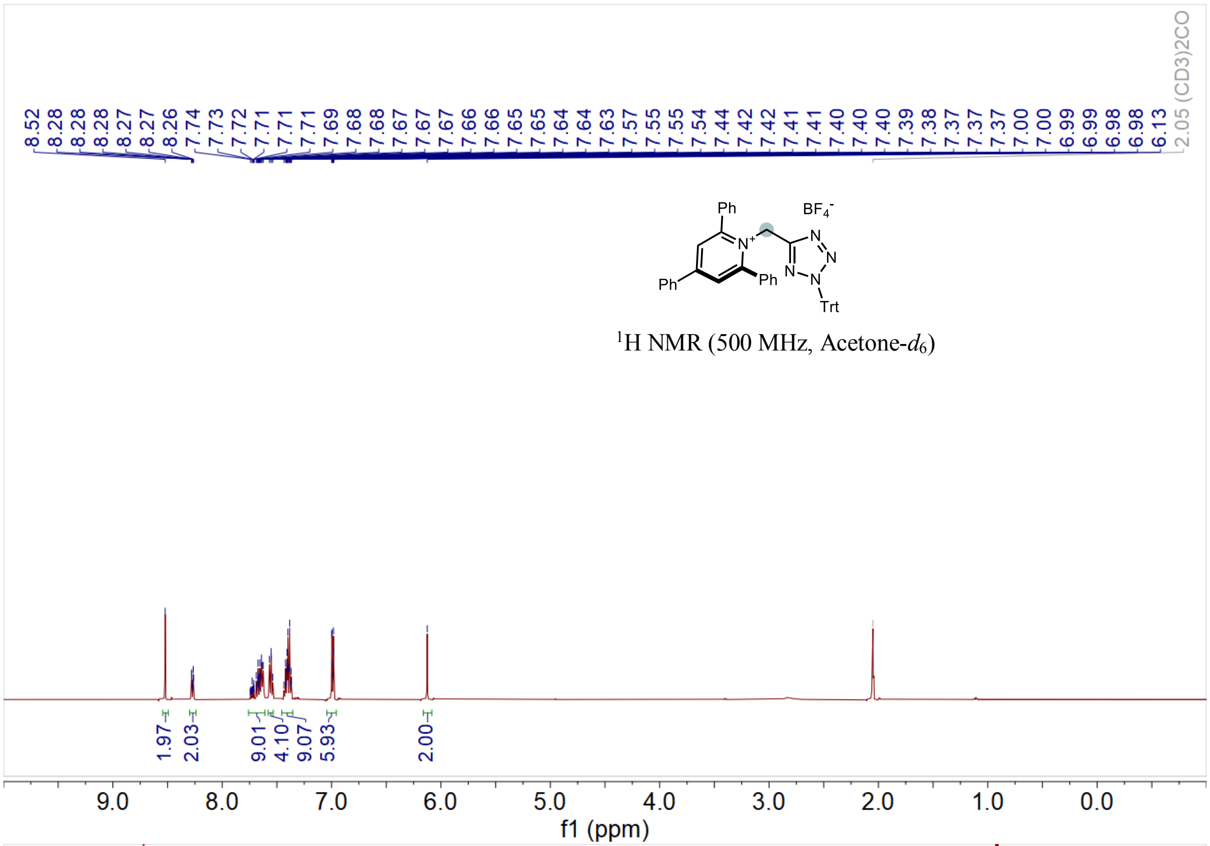

^{1}H NMR (500 MHz, Acetone-d_6)

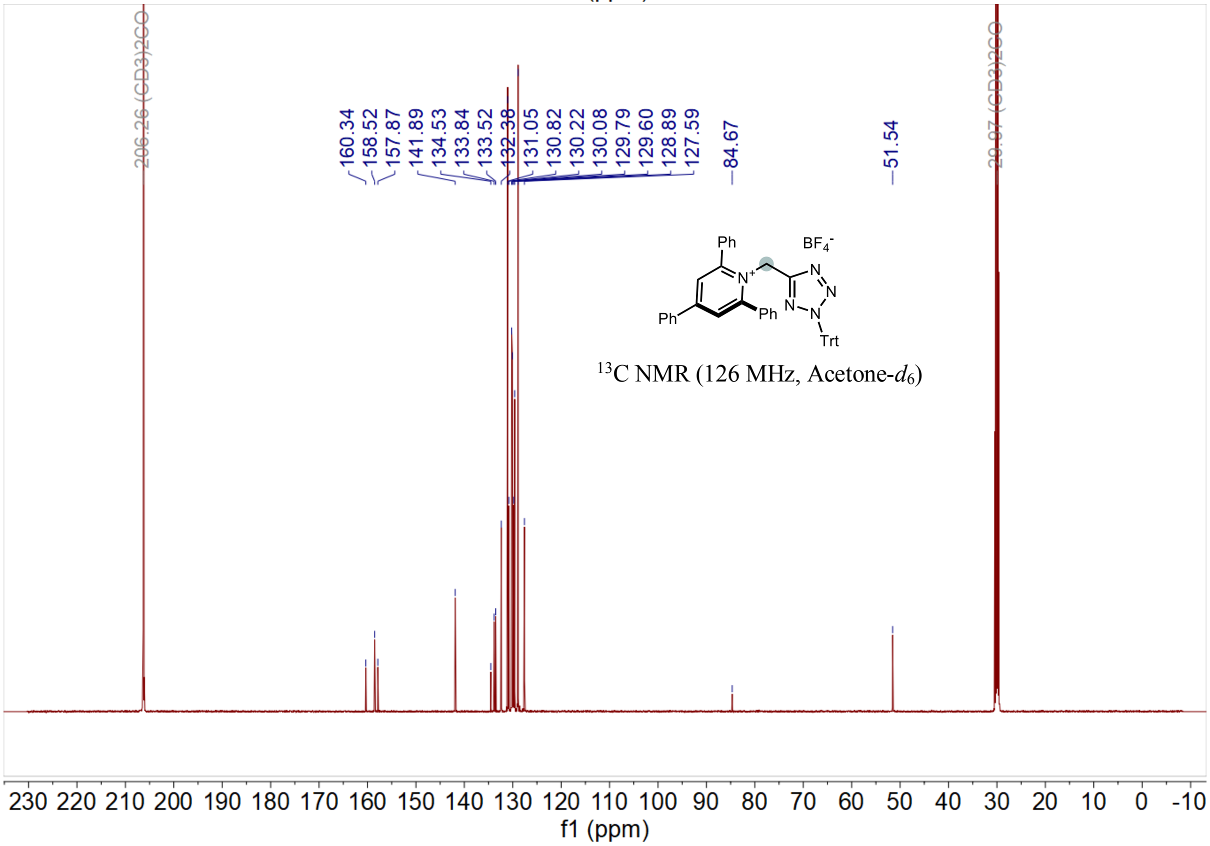

^{13}C NMR (126 MHz, Acetone-d_6)

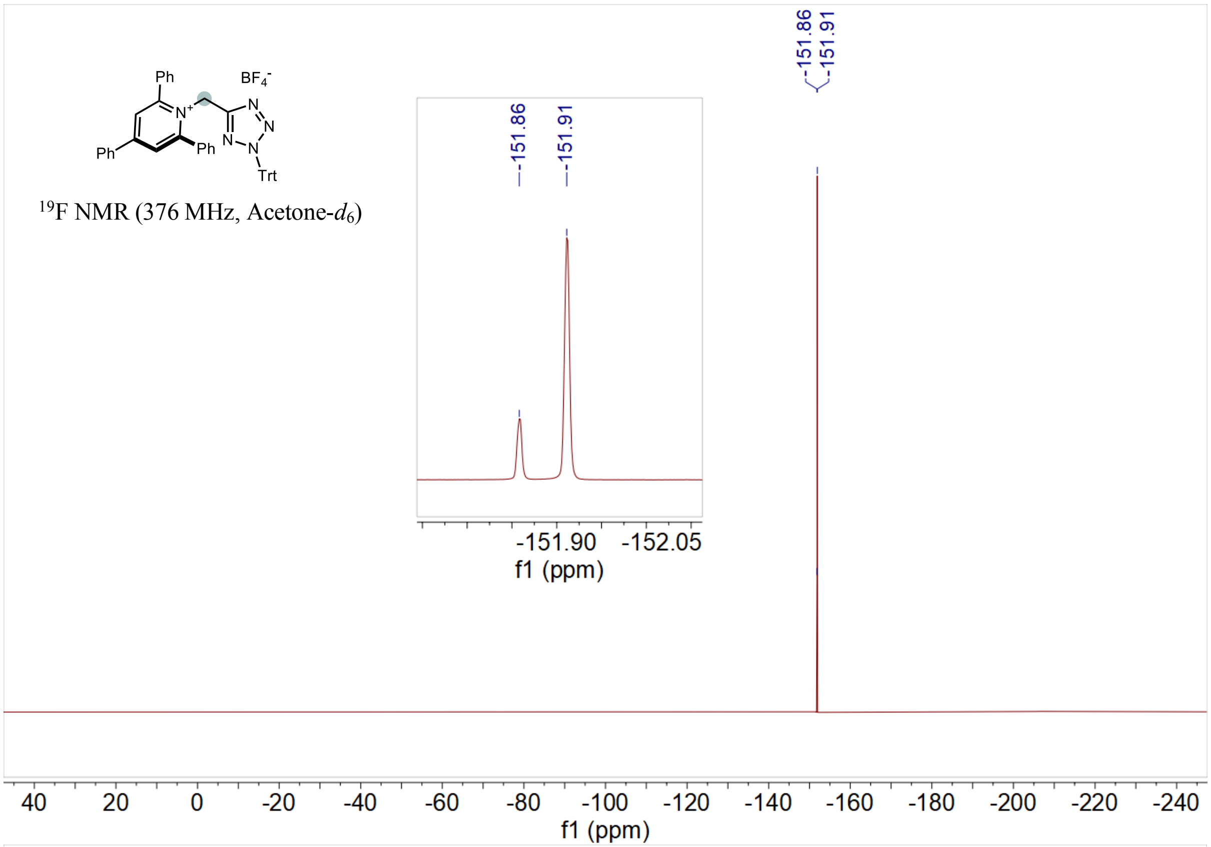

Ph
BF4-
N+
N
N
N
N
Ph
Ph
Trt
19F NMR (376 MHz, Acetone-d6)
-151.86
-151.91
-151.90
-152.05
f1 (ppm)
40
20
0
-20
-40
-60
-80
-100
-120
-140
-160
-180
-200
-220
-240
f1 (ppm)

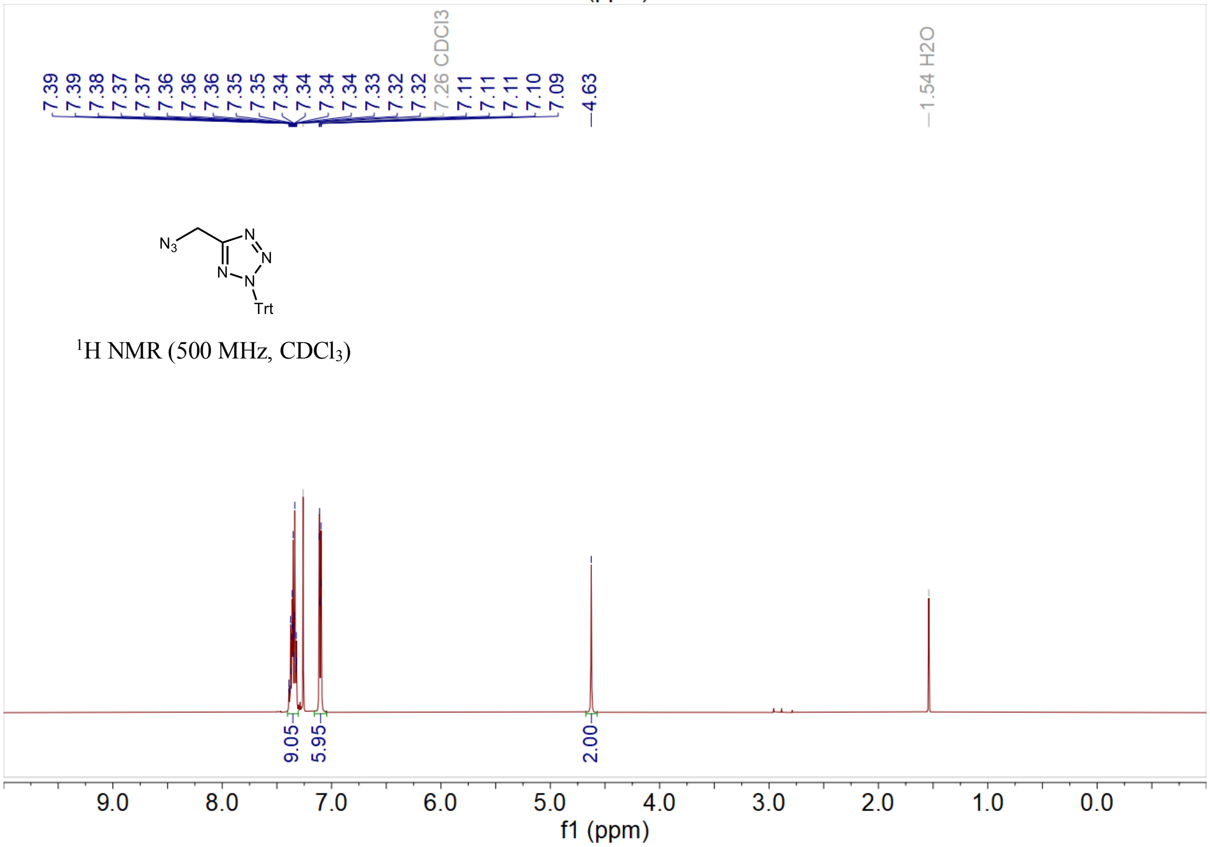

7.39
7.39
7.38
7.37
7.37
7.36
7.36
7.36
7.35
7.35
7.34
7.34
7.34
7.34
7.33
7.32
7.32
7.26 CDCl3
7.11
7.11
7.11
7.11
7.10
7.09
4.63
1.54 H2O
N3
N
N
N
N
Trt
1H NMR (500 MHz, CDCl3)
9.05
5.95
2.00
9.0
8.0
7.0
6.0
5.0
4.0
3.0
2.0
1.0
0.0
f1 (ppm)

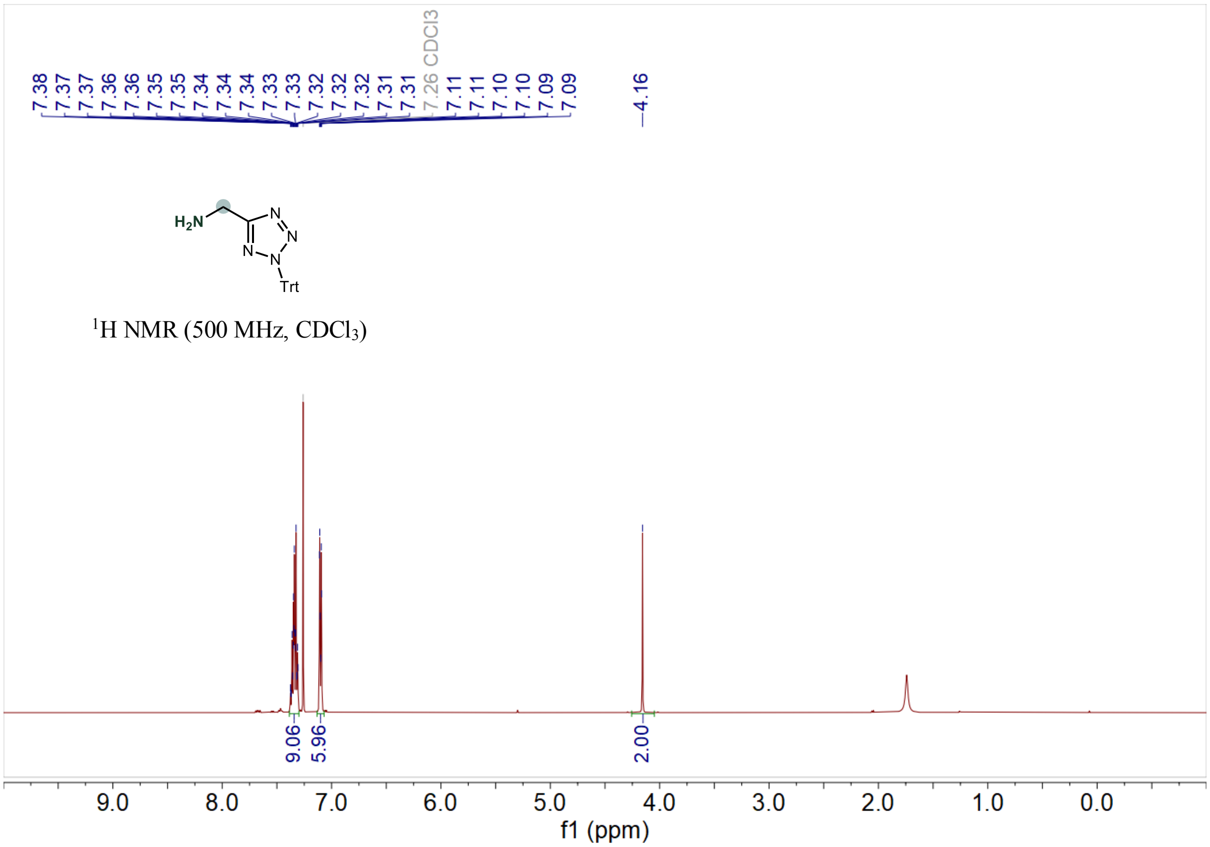
7.38
7.37
7.37
7.36
7.36
7.35
7.35
7.34
7.34
7.34
7.33
7.33
7.32
7.32
7.32
7.31
7.31
7.26 CDCl3
7.11
7.11
7.10
7.10
7.09
7.09
4.16
H2N
N
N
N
N
Trt
1H NMR (500 MHz, CDCl3)
9.06
5.96
2.00
9.0
8.0
7.0
6.0
5.0
4.0
3.0
2.0
1.0
0.0
f1 (ppm)

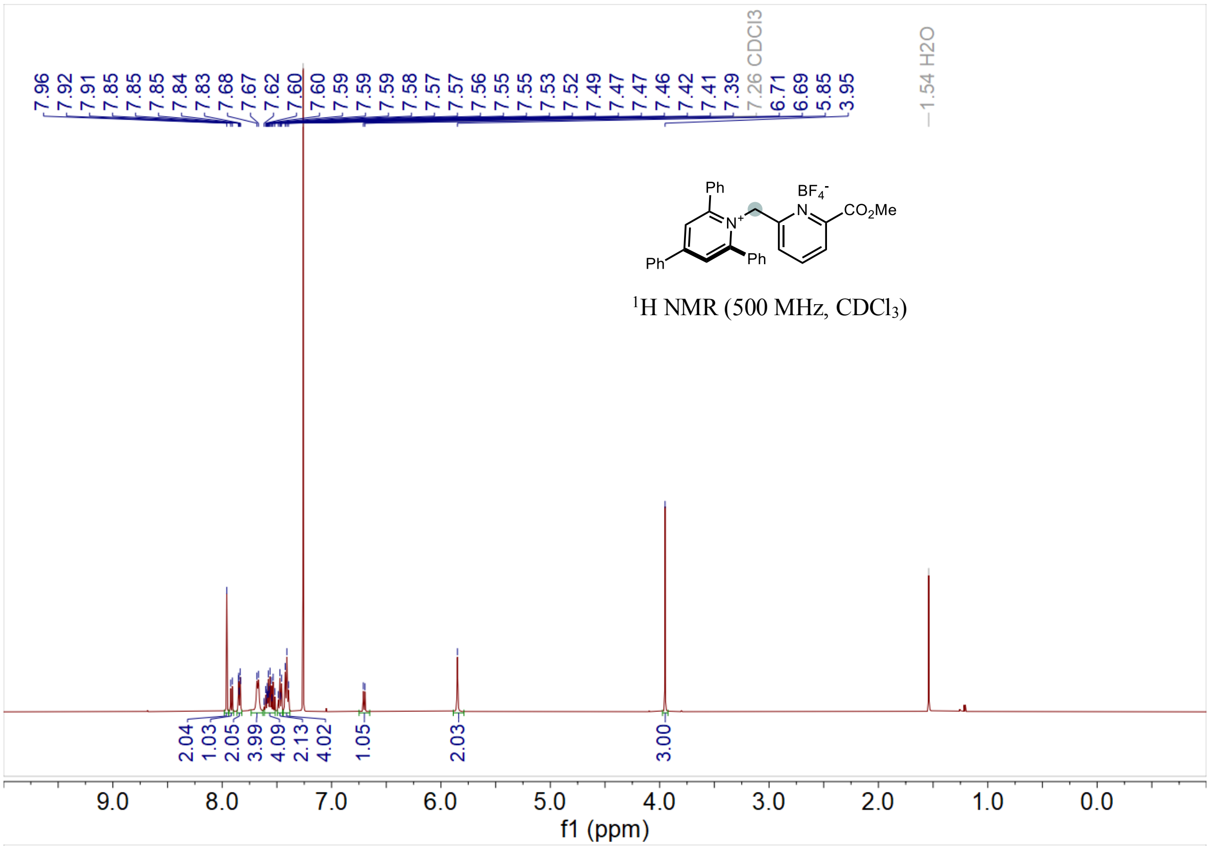
7.96
7.92
7.91
7.85
7.85
7.84
7.83
7.68
7.67
7.62
7.60
7.60
7.59
7.59
7.59
7.58
7.57
7.57
7.56
7.55
7.55
7.53
7.52
7.49
7.47
7.47
7.46
7.42
7.41
7.39
7.26 CDCl3
6.71
6.69
5.85
3.95
1.54 H2O
Ph
BF_4^-
N
CO_2Me
N^+
Ph
Ph
^{1}H NMR (500 MHz, $CDCl_3$)
2.04
1.03
2.05
3.99
4.09
2.13
4.02
1.05
2.03
3.00
9.0
8.0
7.0
6.0
5.0
4.0
3.0
2.0
1.0
0.0
f1 (ppm)

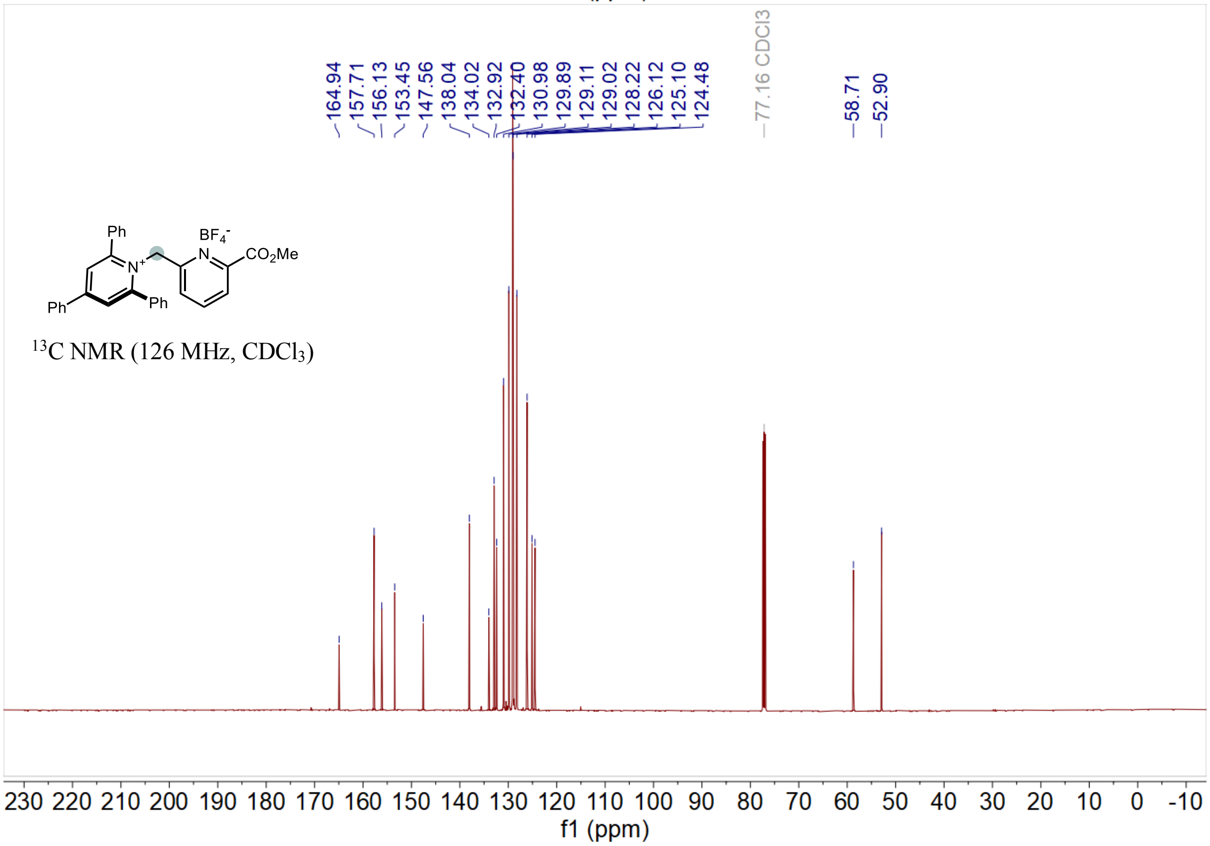
164.94
157.71
156.13
153.45
147.56
138.04
134.02
132.92
132.40
130.98
129.89
129.11
129.02
128.22
126.12
125.10
124.48
77.16 CDCl3
58.71
52.90
Ph
BF_4^-
N
CO_2Me
N^+
Ph
Ph
^{13}C NMR (126 MHz, $CDCl_3$)
230 220 210 200 190 180 170 160 150 140 130 120 110 100 90 80 70 60 50 40 30 20 10 0 -10
f1 (ppm)

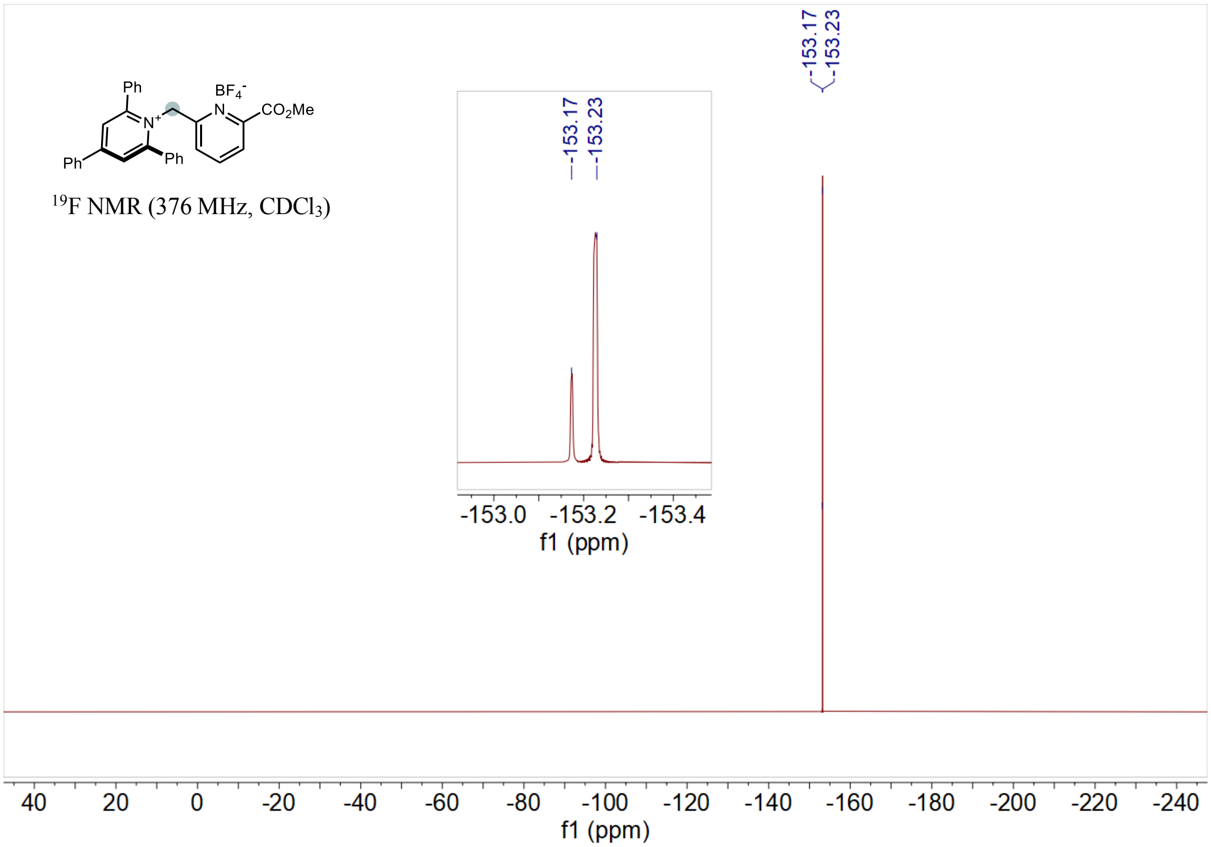

Ph
BF4-
N+
N
CO2Me
Ph
Ph
19F NMR (376 MHz, CDCl3)
-153.17
-153.23
-153.0
-153.2
-153.4
f1 (ppm)
40
20
0
-20
-40
-60
-80
-100
-120
-140
-160
-180
-200
-220
-240
f1 (ppm)

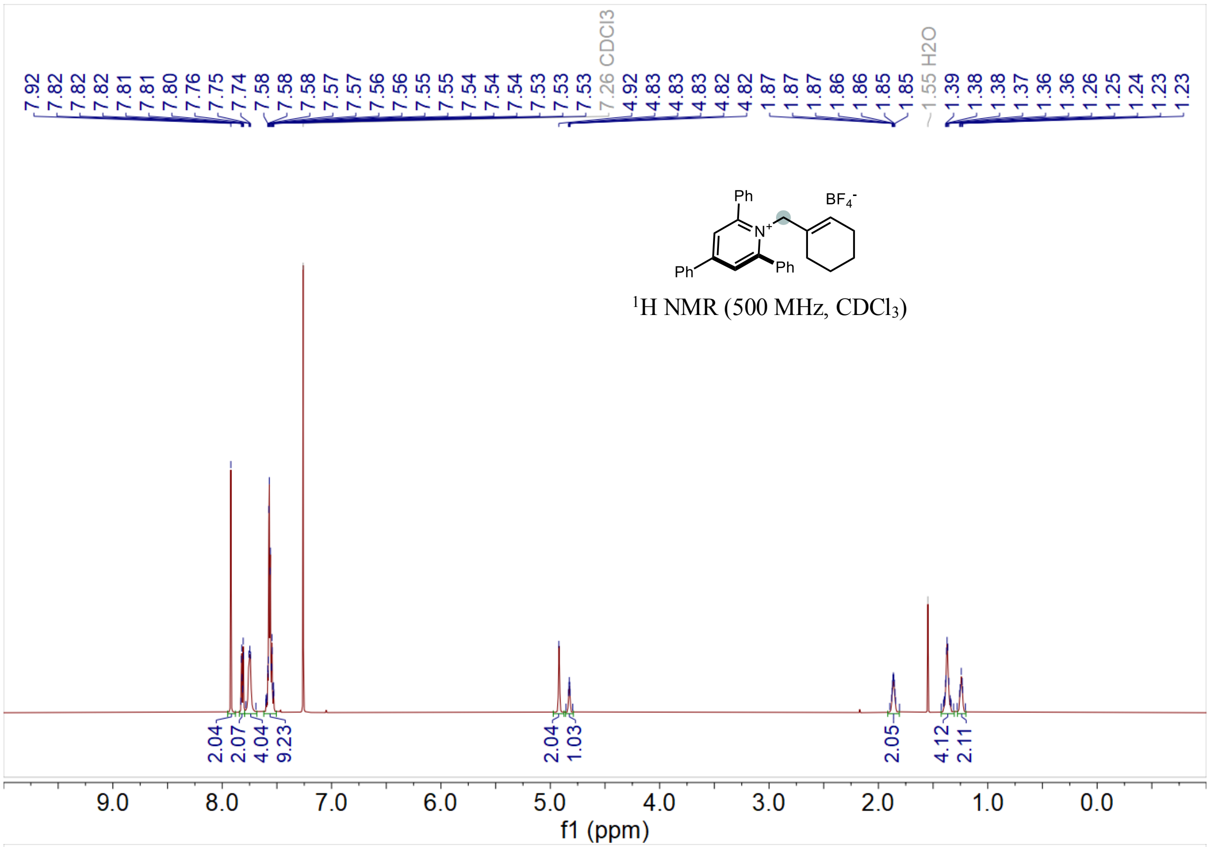
7.92 7.82 7.82 7.82 7.81 7.81 7.80 7.76 7.75 7.74 7.58 7.58 7.58 7.57 7.57 7.56 7.56 7.55 7.55 7.54 7.54 7.54 7.53 7.53 7.53
7.26 CDCl3
4.92 4.83 4.83 4.83 4.82 4.82
1.87 1.87 1.86 1.86 1.85 1.85
1.55 H2O
1.39 1.38 1.38 1.37 1.36 1.36 1.26 1.25 1.24 1.23 1.23
Ph
BF4-
N+
Ph
Ph
1H NMR (500 MHz, CDCl3)
2.04 2.07 4.04 9.23
2.04 1.03
2.05
4.12 2.11
9.0 8.0 7.0 6.0 5.0 4.0 3.0 2.0 1.0 0.0
f1 (ppm)

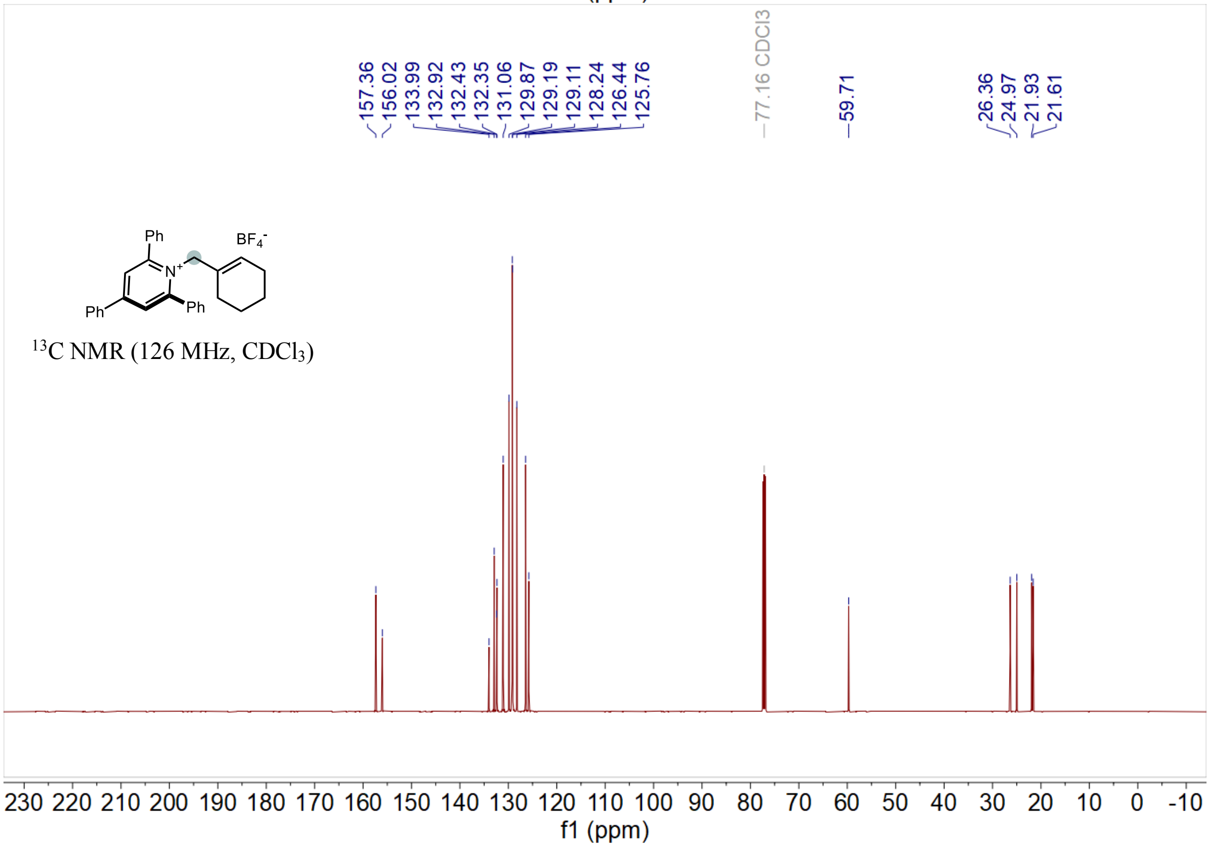
157.36 156.02 133.99 132.92 132.43 132.35 131.06 129.87 129.19 129.11 128.24 126.44 125.76
77.16 CDCl3
59.71
26.36 24.97 21.93 21.61
Ph
BF4-
N+
Ph
Ph
13C NMR (126 MHz, CDCl3)
230 220 210 200 190 180 170 160 150 140 130 120 110 100 90 80 70 60 50 40 30 20 10 0 -10
f1 (ppm)

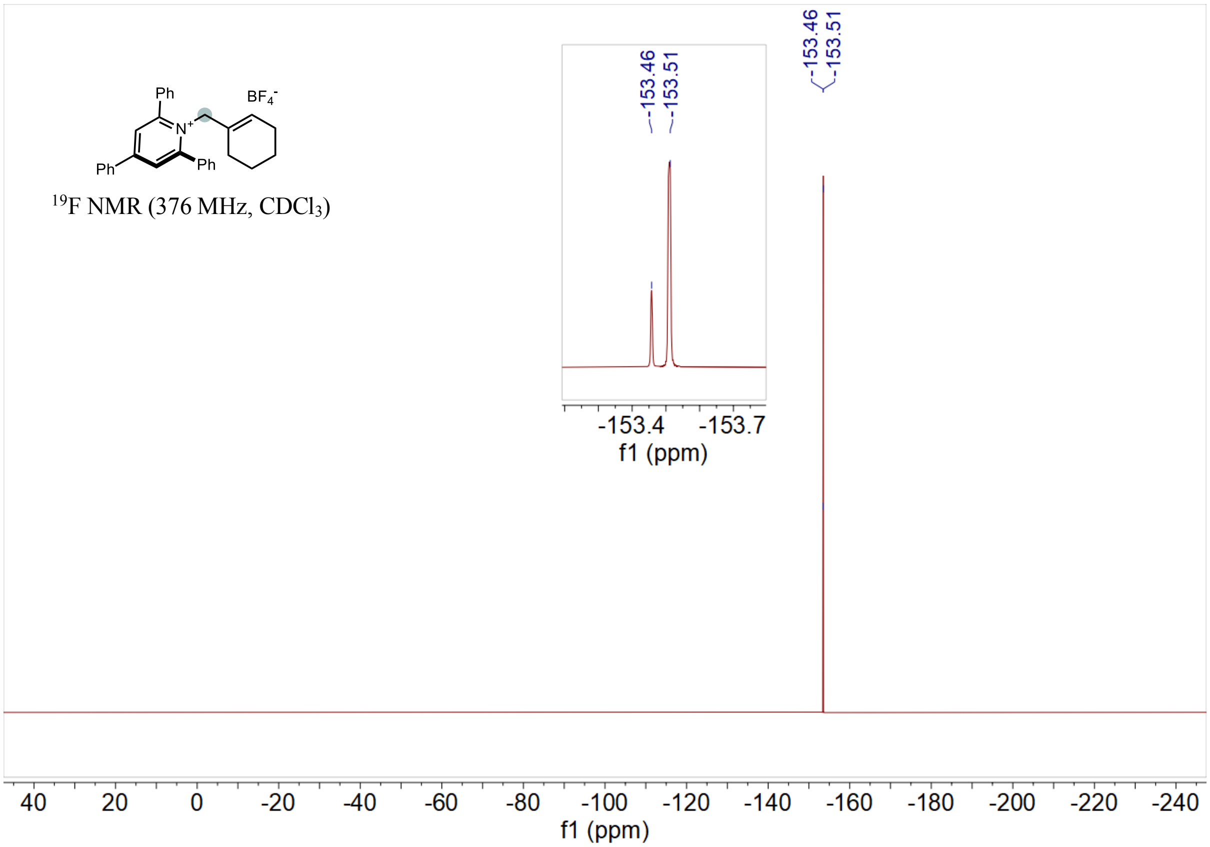

Ph
BF4-
Ph
Ph
19F NMR (376 MHz, CDCl3)
-153.46
-153.51
-153.4
-153.7
f1 (ppm)
40
20
0
-20
-40
-60
-80
-100
-120
-140
-160
-180
-200
-220
-240
f1 (ppm)

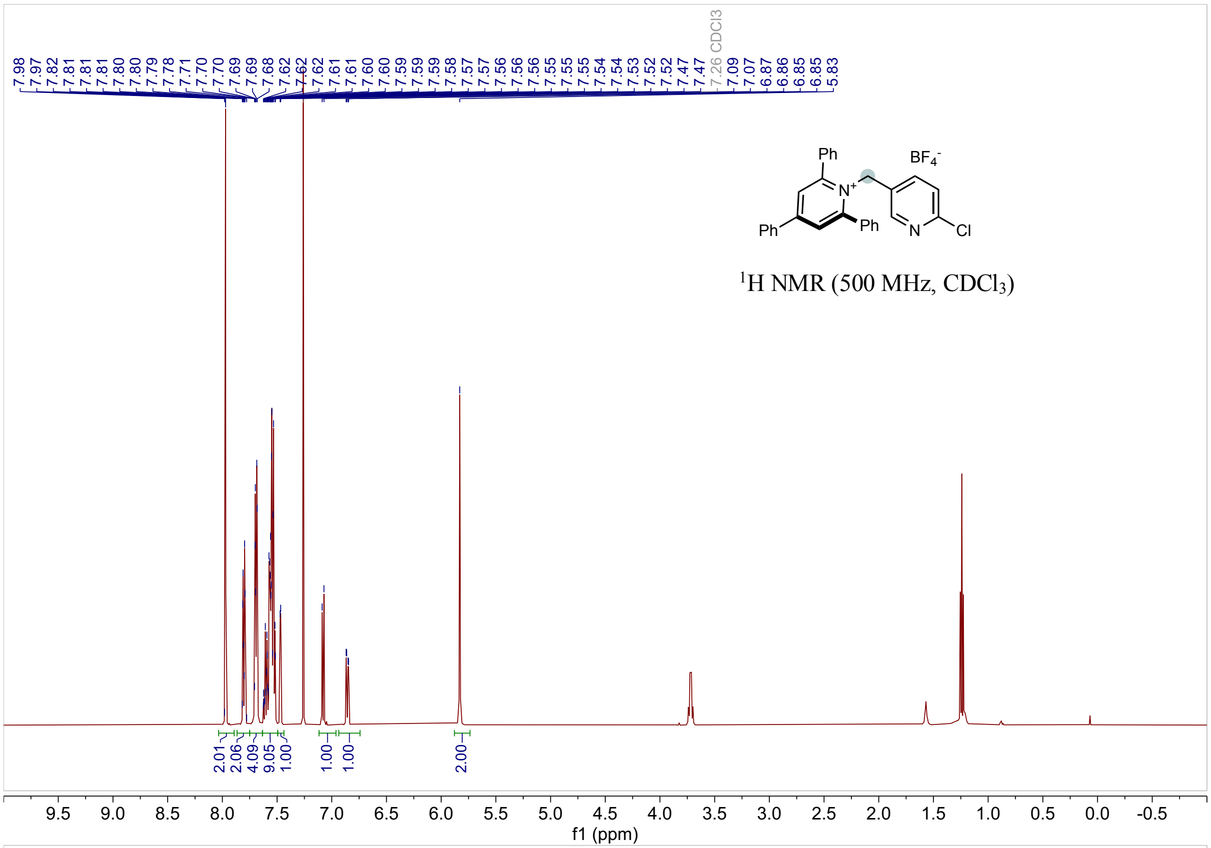
7.98
7.97
7.82
7.81
7.81
7.80
7.80
7.79
7.78
7.71
7.70
7.69
7.69
7.68
7.62
7.62
7.62
7.61
7.61
7.60
7.60
7.59
7.59
7.59
7.58
7.57
7.56
7.56
7.56
7.55
7.55
7.55
7.54
7.54
7.53
7.52
7.52
7.47
7.47
7.26 CDCl3
7.09
7.07
6.87
6.86
6.85
6.85
5.83
Ph
BF4-
N+
Ph
Ph
N
Cl
1H NMR (500 MHz, CDCl3)
2.01
2.06
4.09
9.05
1.00
1.00
1.00
2.00
f1 (ppm)

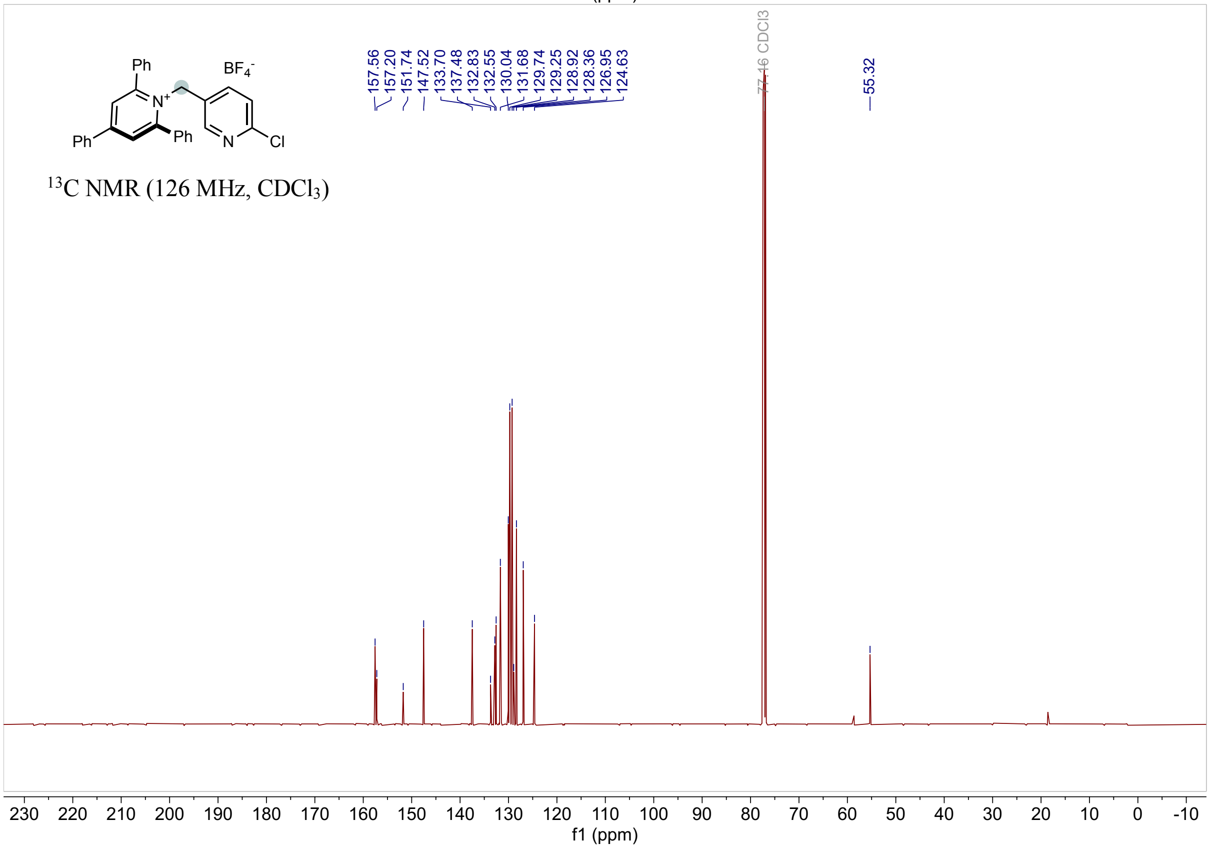
Ph
BF4-
N+
Ph
Ph
N
Cl
13C NMR (126 MHz, CDCl3)
157.56
157.20
151.74
147.52
133.70
137.48
132.83
132.55
130.04
131.68
129.74
129.25
128.92
128.36
126.95
124.63
77.16 CDCl3
55.32
f1 (ppm)

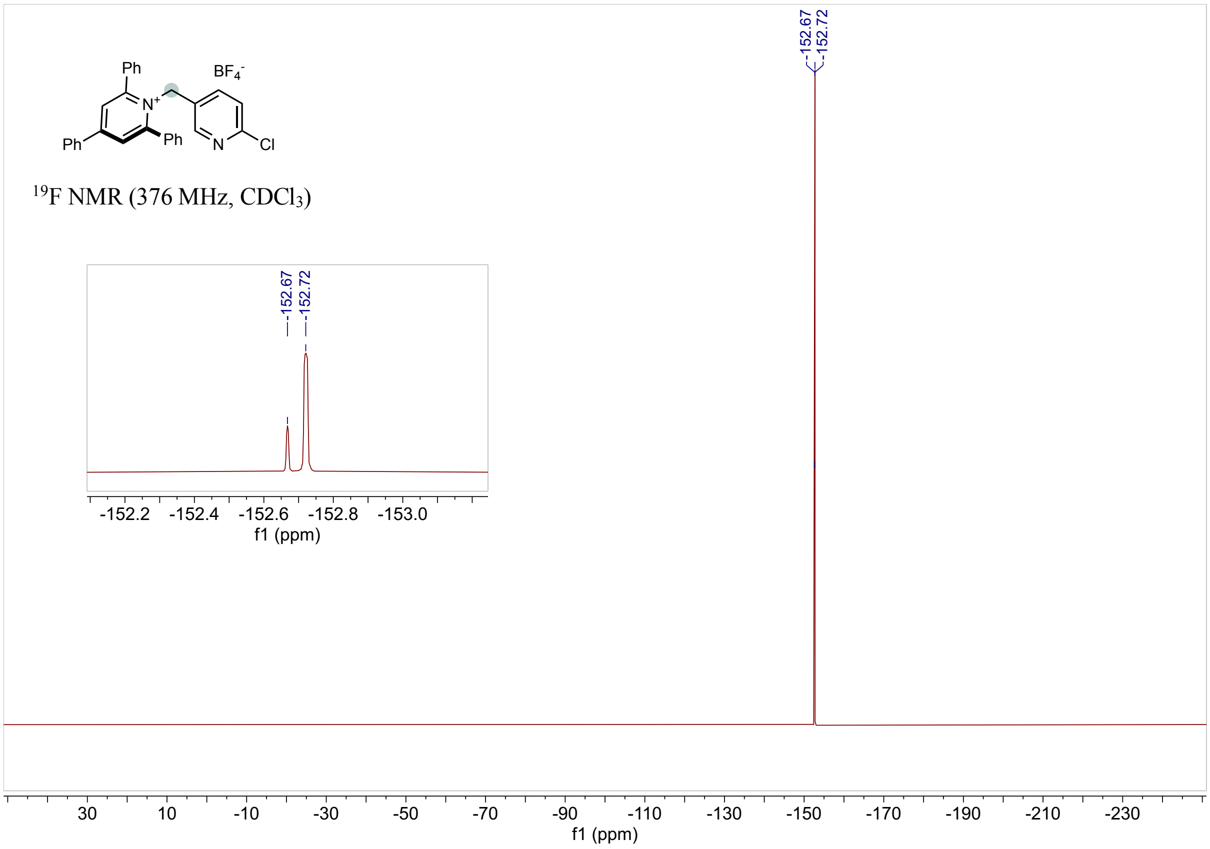

Ph
BF4-
N+
Ph
Ph
N
Cl
19F NMR (376 MHz, CDCl3)
-152.67
-152.72
-152.2
-152.4
-152.6
-152.8
-153.0
f1 (ppm)
30
10
-10
-30
-50
-70
-90
-110
-130
-150
-170
-190
-210
-230
f1 (ppm)

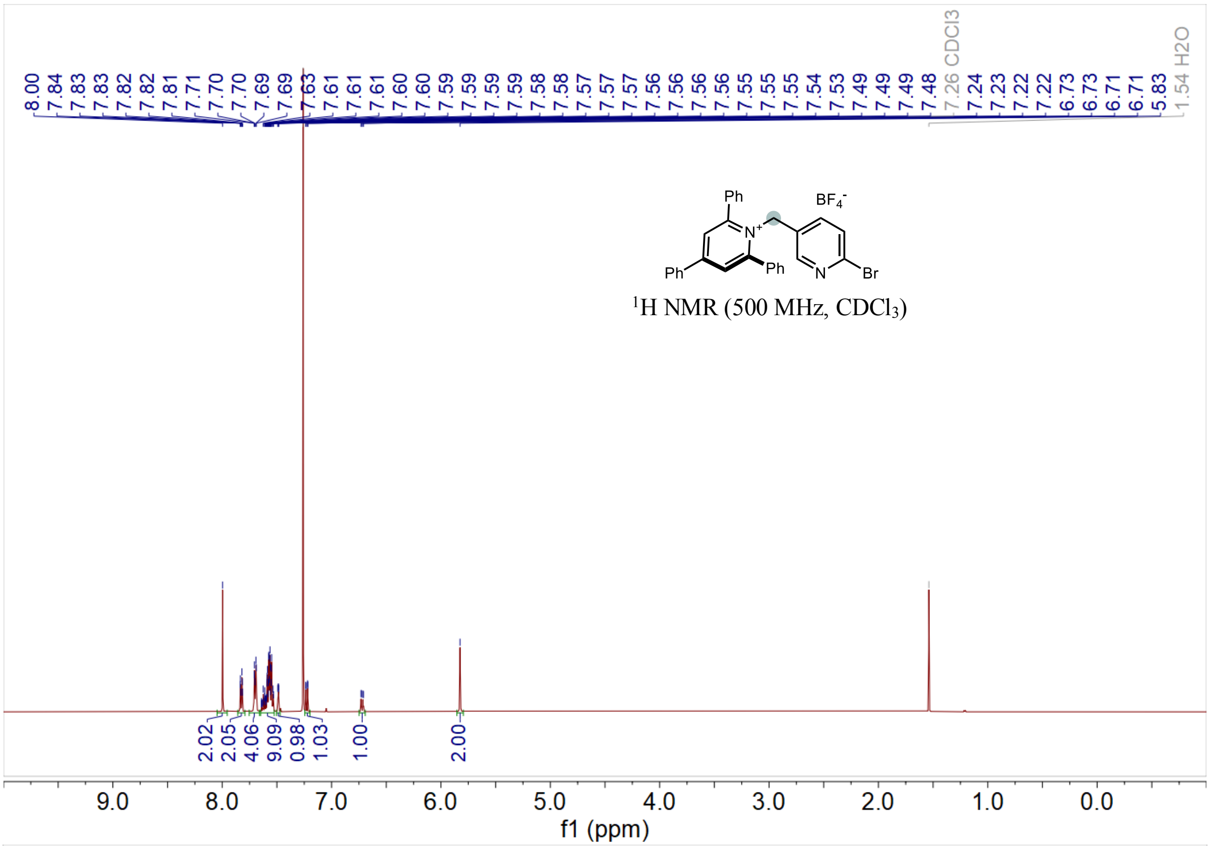
8.00 7.84 7.83 7.82 7.82 7.81 7.71 7.70 7.70 7.69 7.69 7.63 7.61 7.61 7.60 7.60 7.59 7.59 7.59 7.59 7.58 7.58 7.57 7.57 7.57 7.56 7.56 7.56 7.56 7.55 7.55 7.55 7.54 7.53 7.49 7.49 7.49 7.48 7.26 CDCl3 7.24 7.23 7.22 7.22 6.73 6.73 6.71 6.71 5.83 1.54 H2O
Ph
BF4-
N+
Ph
Ph
N
Br
1H NMR (500 MHz, CDCl3)
2.02 2.05 4.06 9.09 0.98 1.03 1.00 2.00
9.0 8.0 7.0 6.0 5.0 4.0 3.0 2.0 1.0 0.0
f1 (ppm)

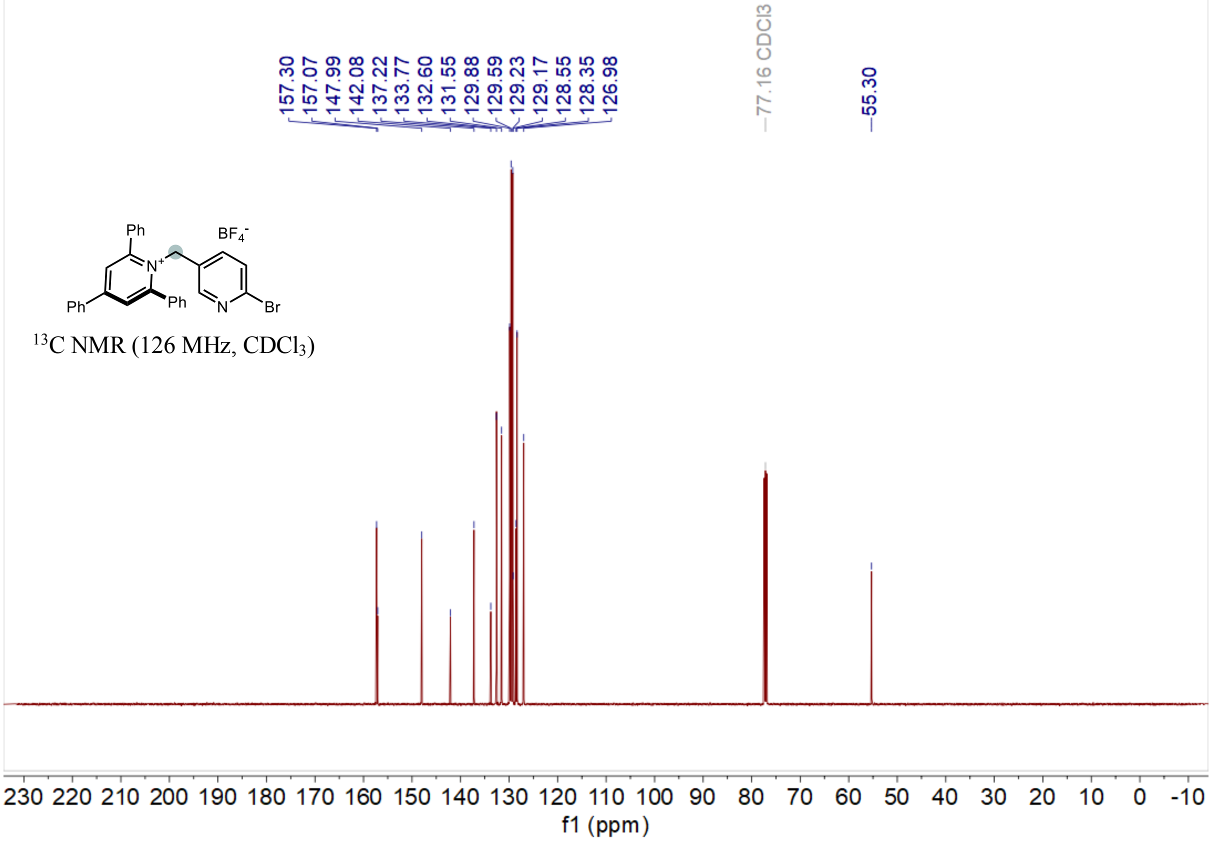
157.30 157.07 147.99 142.08 137.22 133.77 132.60 131.55 129.88 129.59 129.23 129.17 128.55 128.35 126.98
77.16 CDCl3
55.30
Ph
BF4-
N+
Ph
Ph
N
Br
13C NMR (126 MHz, CDCl3)
230 220 210 200 190 180 170 160 150 140 130 120 110 100 90 80 70 60 50 40 30 20 10 0 -10
f1 (ppm)

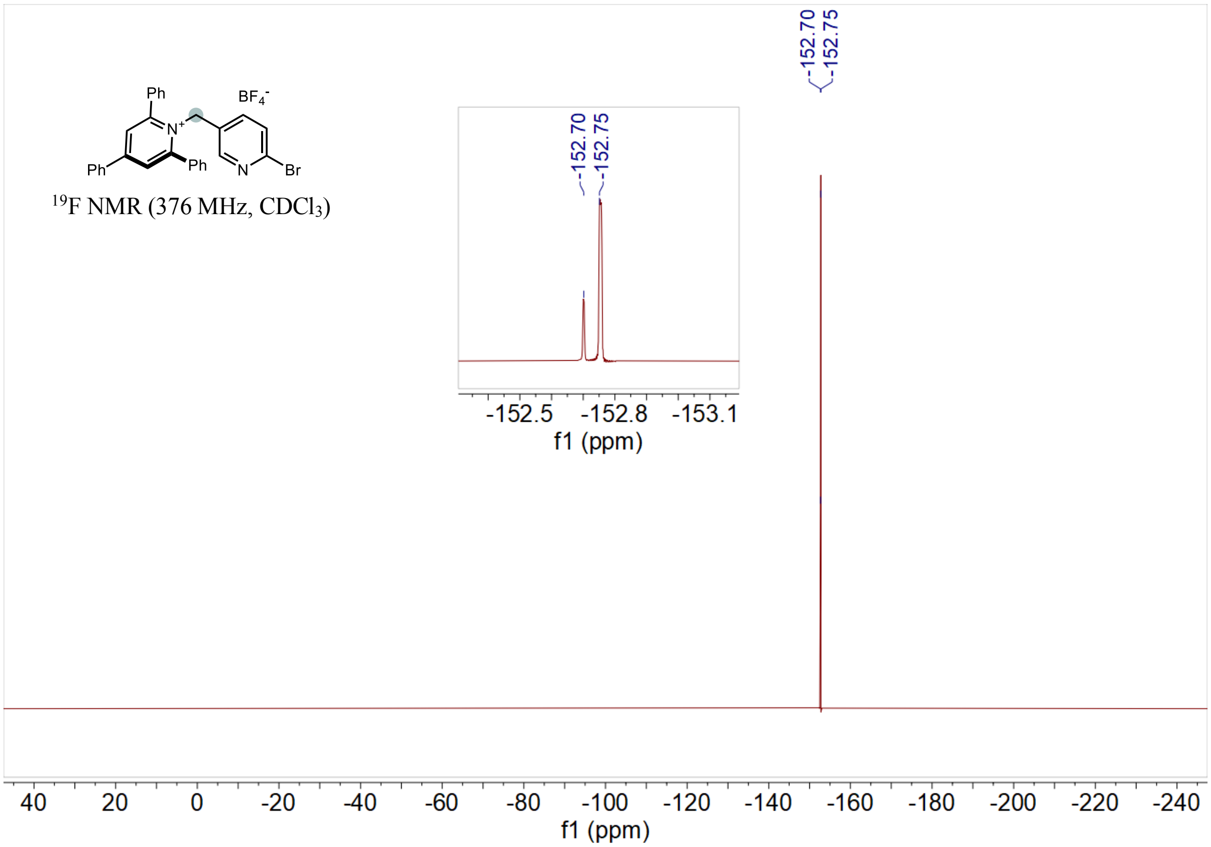

Ph
BF4-
N+
Ph
Ph
N
Br
19F NMR (376 MHz, CDCl3)
-152.70
-152.75
-152.5
-152.8
-153.1
f1 (ppm)
40
20
0
-20
-40
-60
-80
-100
-120
-140
-160
-180
-200
-220
-240
f1 (ppm)

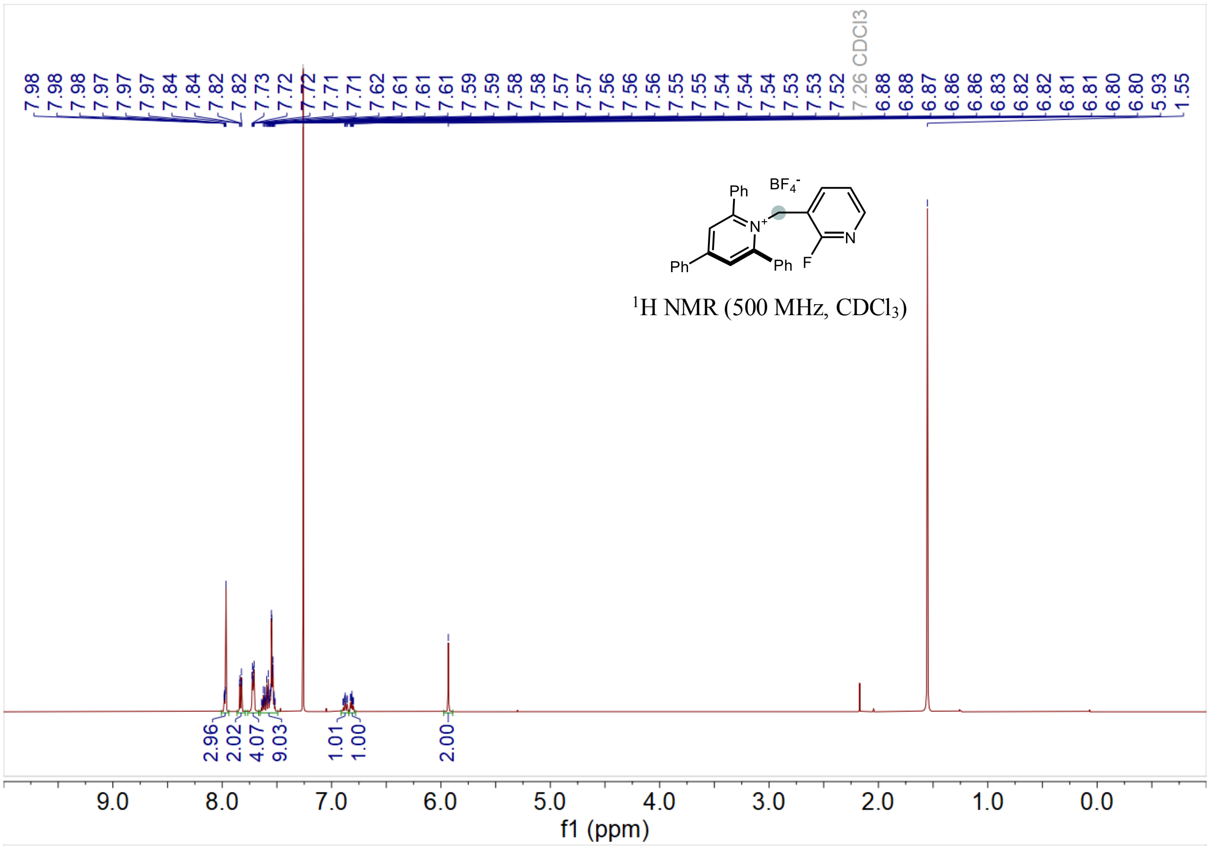

7.98 7.98 7.98 7.97 7.97 7.97 7.84 7.84 7.82 7.82 7.73 7.72 7.72 7.71 7.71 7.62 7.61 7.61 7.61 7.59 7.59 7.58 7.58 7.57 7.57 7.56 7.56 7.56 7.55 7.55 7.54 7.54 7.54 7.53 7.53 7.52 7.26 CDCl3 6.88 6.88 6.87 6.86 6.86 6.83 6.82 6.82 6.81 6.81 6.80 6.80 5.93 1.55
Ph
BF4-
N+
N
Ph
Ph
F
1H NMR (500 MHz, CDCl3)
2.96 2.02 4.07 9.03 1.01 1.00 2.00
9.0 8.0 7.0 6.0 5.0 4.0 3.0 2.0 1.0 0.0
f1 (ppm)

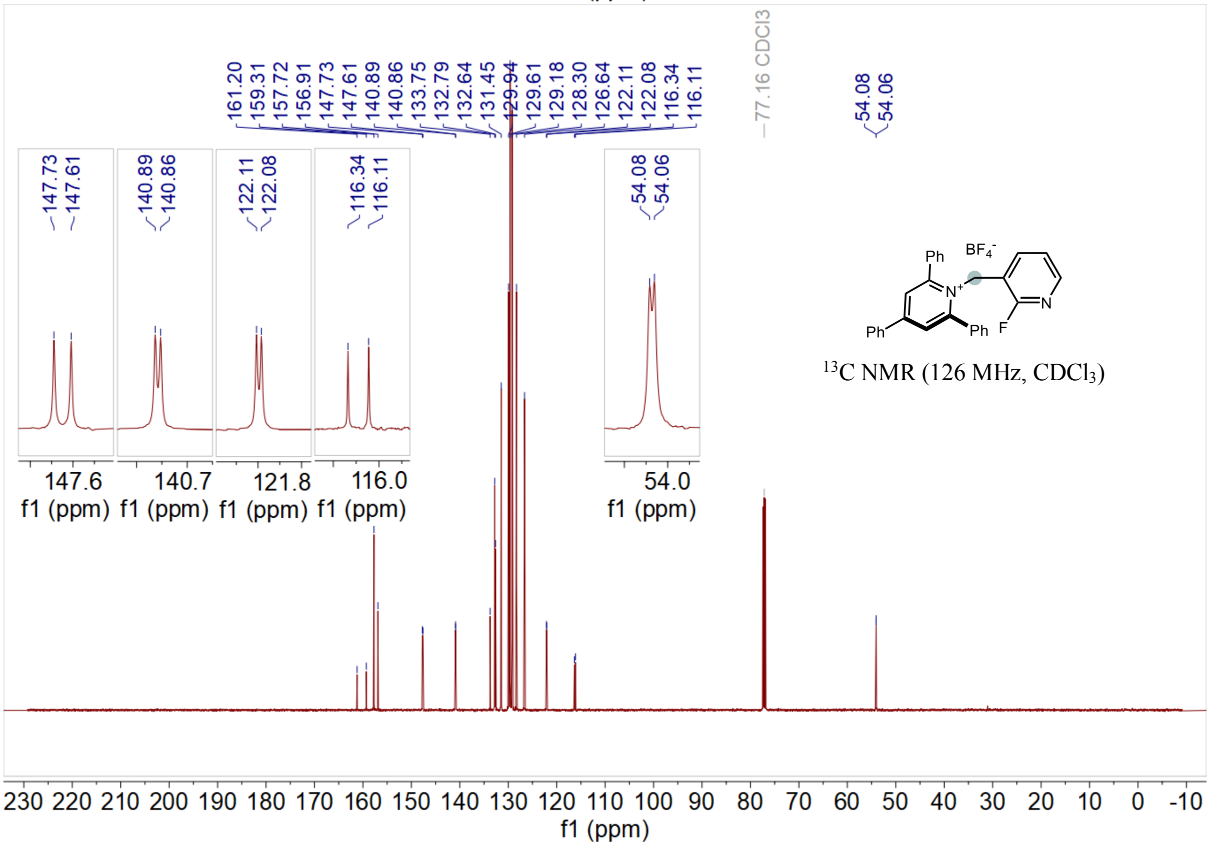

161.20 159.31 157.72 156.91 147.73 147.61 140.89 140.86 133.75 132.79 132.64 131.45 129.94 129.61 129.18 128.30 126.64 122.11 122.08 116.34 116.11
77.16 CDCl3
54.08 54.06
147.73 147.61
140.89 140.86
122.11 122.08
116.34 116.11
54.08 54.06
147.6 140.7 121.8 116.0 54.0
f1 (ppm) f1 (ppm) f1 (ppm) f1 (ppm) f1 (ppm)
Ph
BF4-
N+
N
Ph
Ph
F
13C NMR (126 MHz, CDCl3)
230 220 210 200 190 180 170 160 150 140 130 120 110 100 90 80 70 60 50 40 30 20 10 0 -10
f1 (ppm)

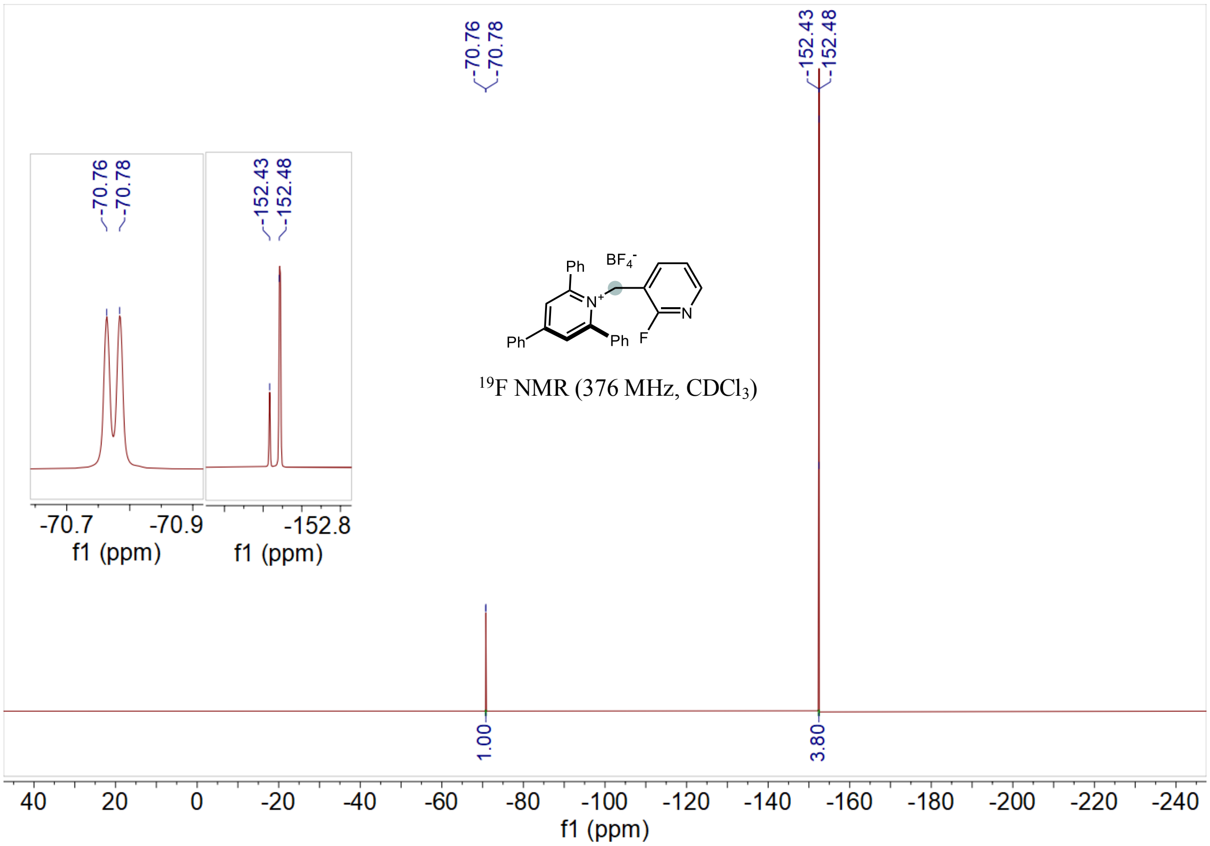
-70.76
-70.78
-152.43
-152.48
BF4-
Ph
N+
Ph
Ph
F
N
19F NMR (376 MHz, CDCl3)
-70.7
-70.9
f1 (ppm)
-152.8
f1 (ppm)
1.00
3.80
40 20 0 -20 -40 -60 -80 -100 -120 -140 -160 -180 -200 -220 -240
f1 (ppm)

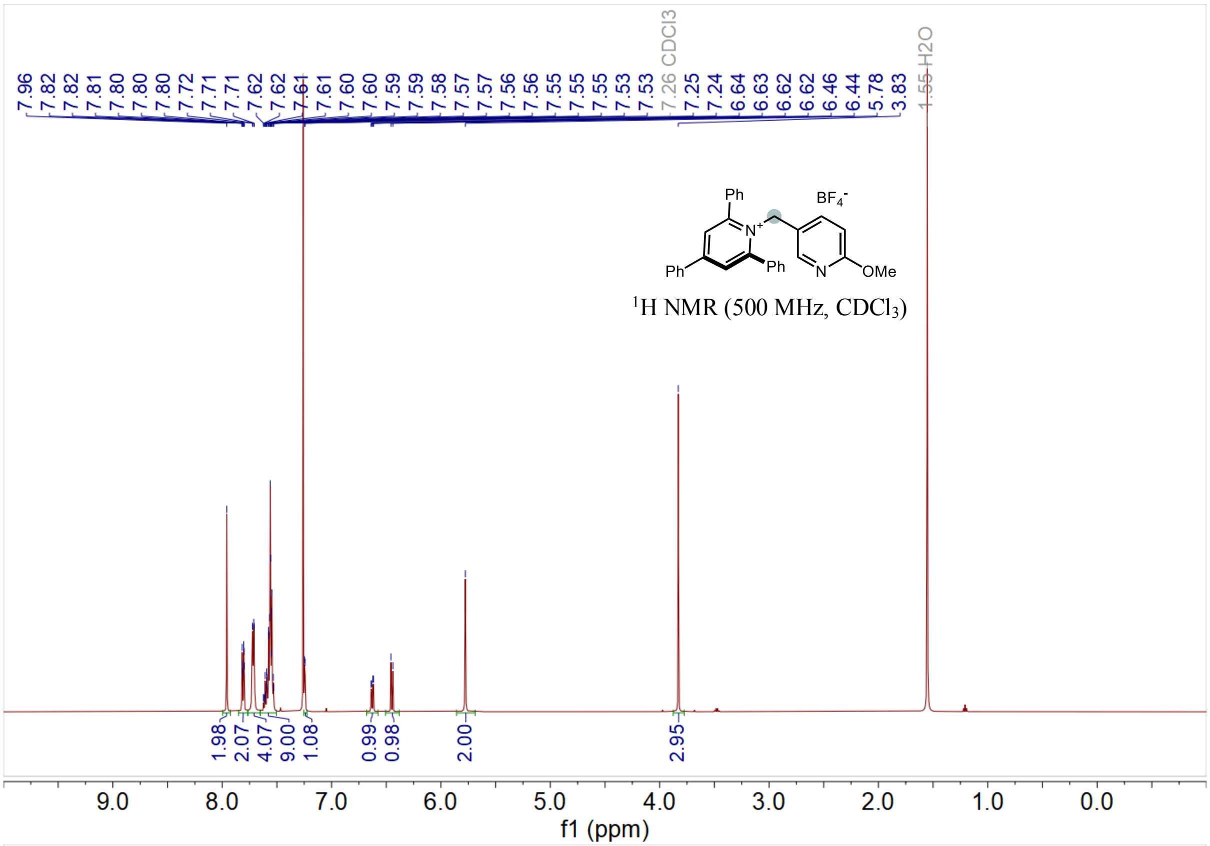
7.96
7.82
7.82
7.81
7.80
7.80
7.80
7.72
7.71
7.71
7.62
7.62
7.61
7.61
7.60
7.60
7.59
7.59
7.58
7.57
7.57
7.56
7.56
7.55
7.55
7.55
7.53
7.53
7.26 CDCl3
7.25
7.24
6.64
6.63
6.62
6.62
6.46
6.44
5.78
3.83
1.55 H2O
Ph
BF_4^-
N^+
Ph
Ph
N
OMe
^1H NMR (500 MHz, $CDCl_3$)
1.98
2.07
4.07
9.00
1.08
0.99
0.98
2.00
2.95
9.0
8.0
7.0
6.0
5.0
4.0
3.0
2.0
1.0
0.0
f1 (ppm)

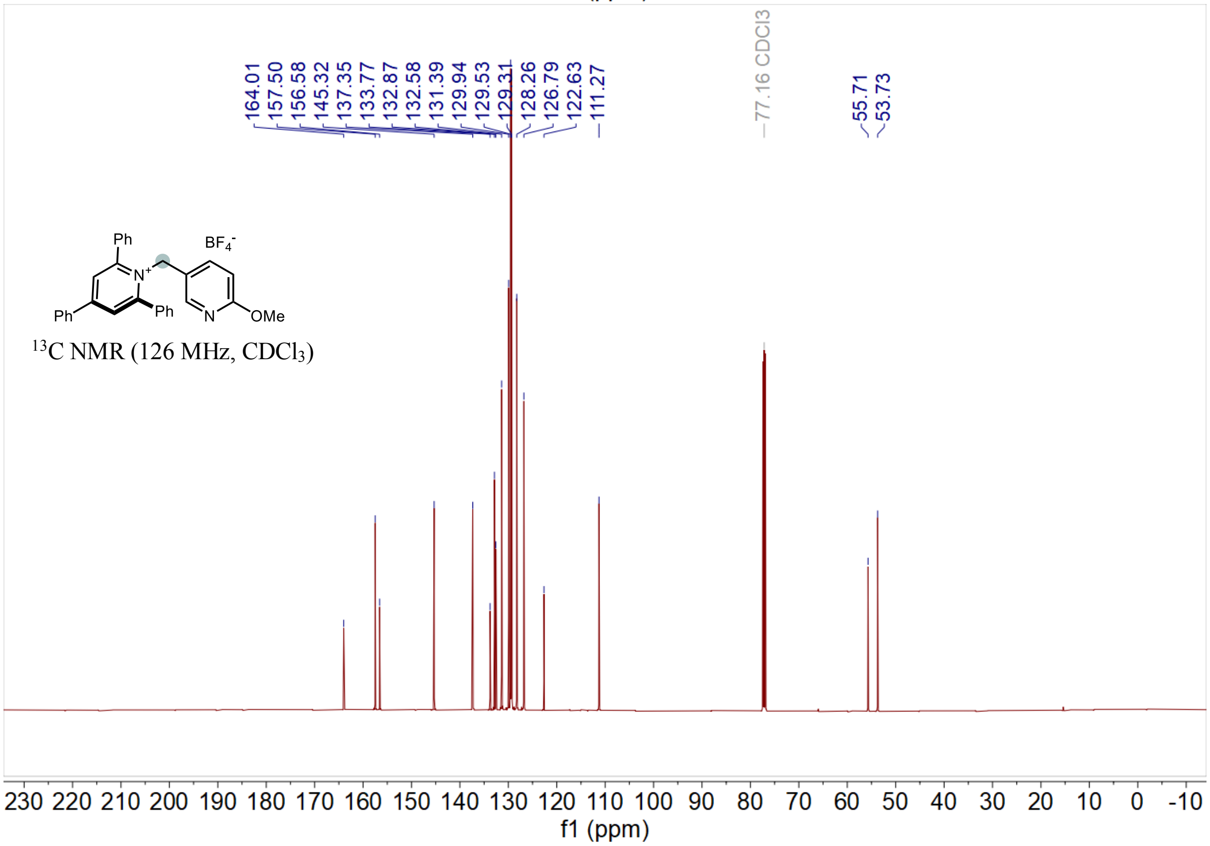
164.01
157.50
156.58
145.32
137.35
133.77
132.87
132.58
131.39
129.94
129.53
129.31
128.26
126.79
122.63
111.27
77.16 CDCl3
55.71
53.73
Ph
BF_4^-
N^+
Ph
Ph
N
OMe
^{13}C NMR (126 MHz, $CDCl_3$)
230
220
210
200
190
180
170
160
150
140
130
120
110
100
90
80
70
60
50
40
30
20
10
0
-10
f1 (ppm)

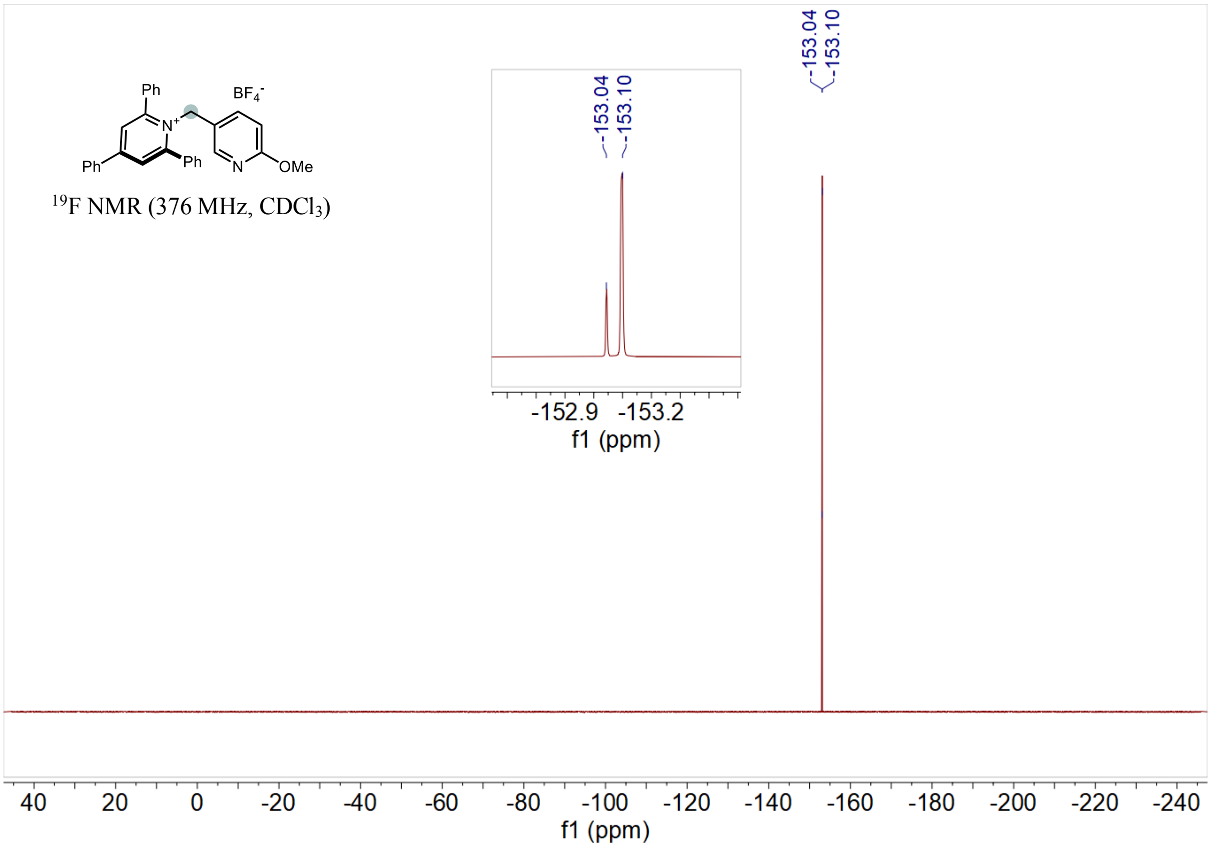
Ph
BF4-
N+
Ph
Ph
N
OMe
19F NMR (376 MHz, CDCl3)
-153.04
-153.10
-152.9
-153.2
f1 (ppm)
-153.04
-153.10
40
20
0
-20
-40
-60
-80
-100
-120
-140
-160
-180
-200
-220
-240
f1 (ppm)

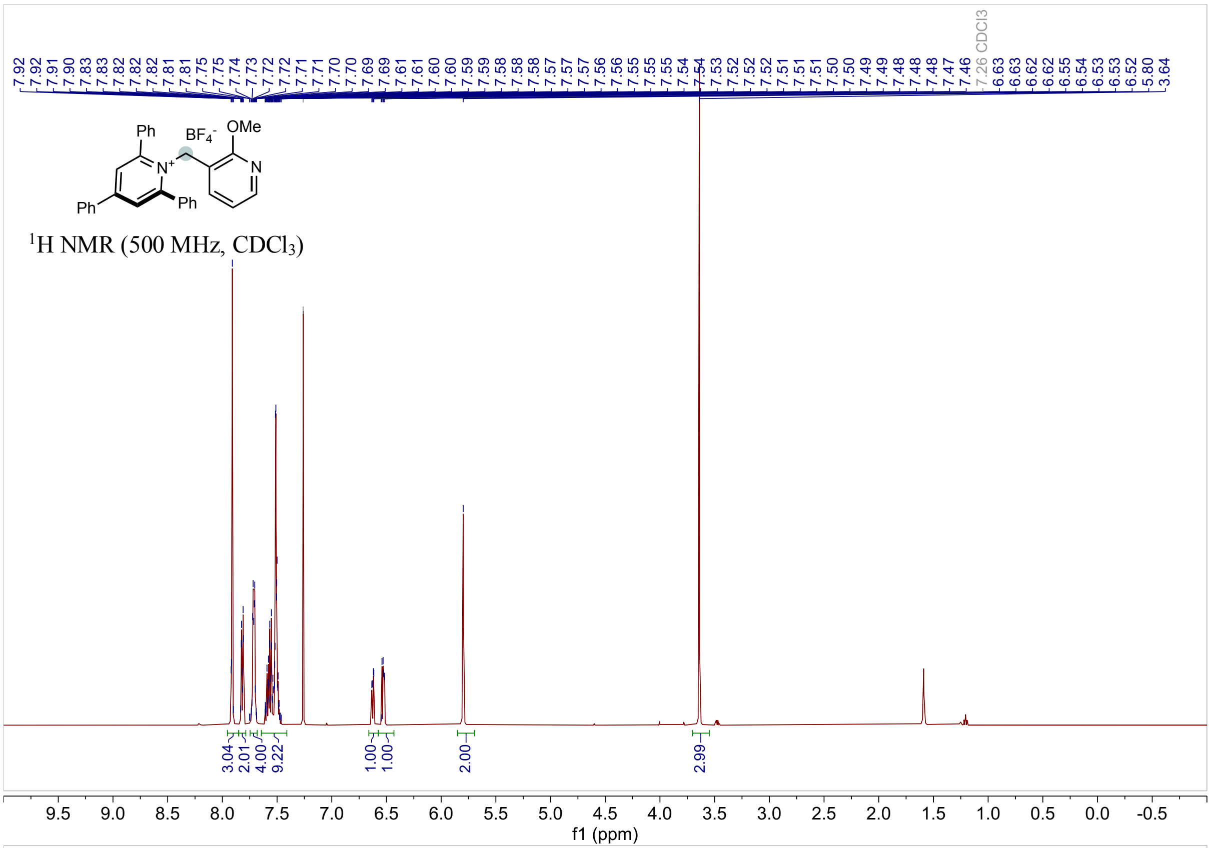

7.92
7.92
7.91
7.90
7.83
7.83
7.82
7.82
7.82
7.81
7.75
7.74
7.73
7.72
7.72
7.71
7.70
7.69
7.69
7.61
7.61
7.60
7.59
7.59
7.58
7.58
7.58
7.57
7.57
7.56
7.56
7.55
7.55
7.55
7.54
7.54
7.53
7.52
7.52
7.52
7.51
7.51
7.51
7.50
7.50
7.49
7.49
7.48
7.48
7.48
7.47
7.46
7.26 CDCl3
6.63
6.63
6.62
6.62
6.55
6.54
6.53
6.53
6.52
5.80
3.64
Ph
BF4-
OMe
N+
N
Ph
Ph
1H NMR (500 MHz, CDCl3)
3.04
2.01
4.00
9.22
1.00
1.00
2.00
2.99
9.5 9.0 8.5 8.0 7.5 7.0 6.5 6.0 5.5 5.0 4.5 4.0 3.5 3.0 2.5 2.0 1.5 1.0 0.5 0.0 -0.5
f1 (ppm)

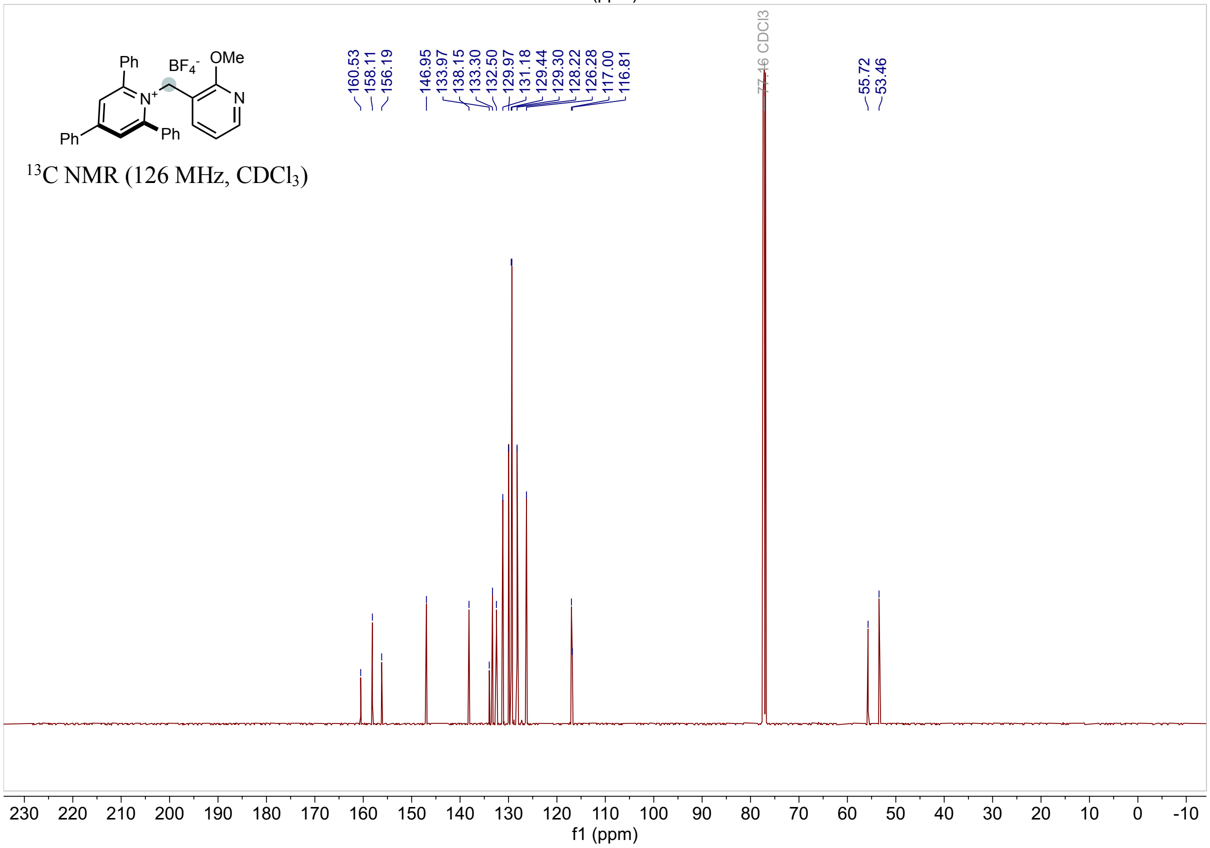

160.53
158.11
156.19
146.95
133.97
138.15
133.30
132.50
129.97
131.18
129.44
129.30
128.22
126.28
117.00
116.81
77.16 CDCl3
55.72
53.46
Ph
BF4-
OMe
N+
N
Ph
Ph
13C NMR (126 MHz, CDCl3)
230 220 210 200 190 180 170 160 150 140 130 120 110 100 90 80 70 60 50 40 30 20 10 0 -10
f1 (ppm)

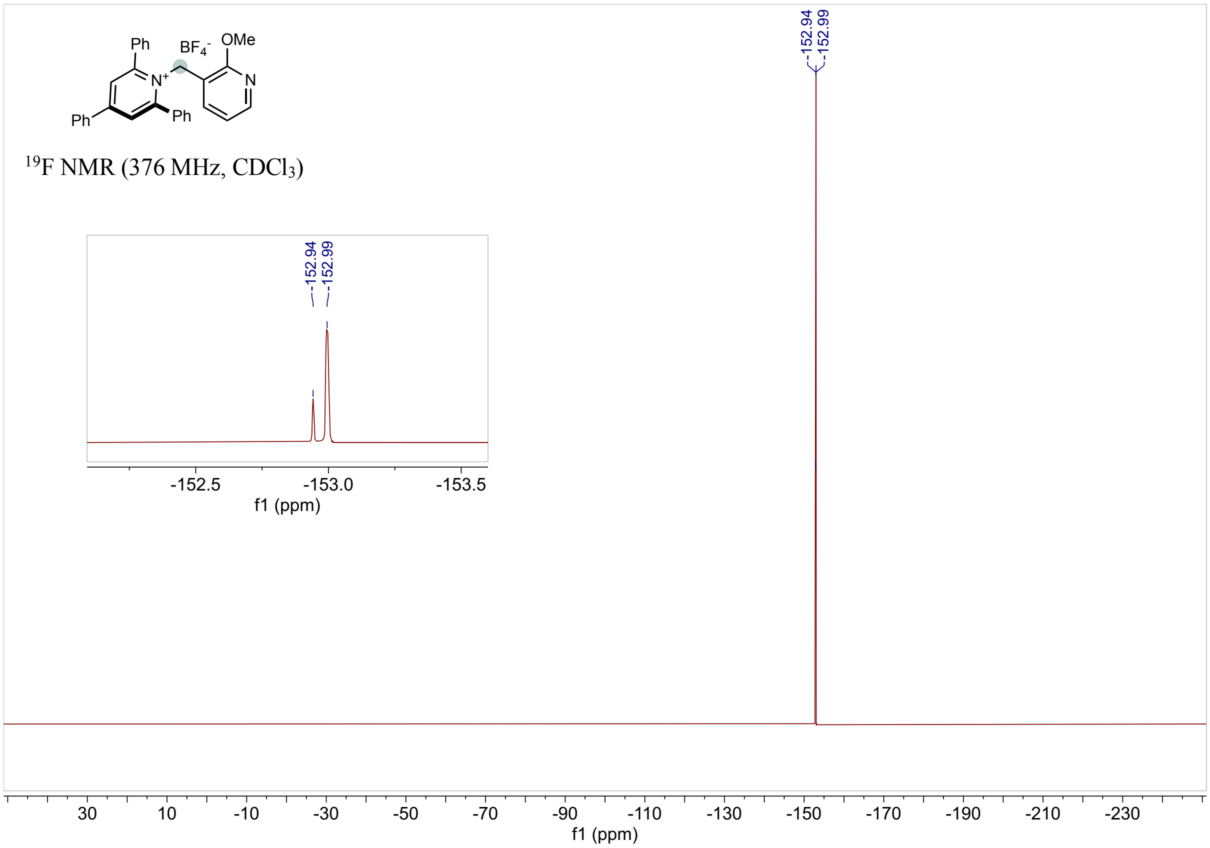

Ph
BF4-
OMe
N+
N
Ph
Ph
19F NMR (376 MHz, CDCl3)
-152.94
-152.99
-152.5
-153.0
-153.5
f1 (ppm)
30
10
-10
-30
-50
-70
-90
-110
-130
-150
-170
-190
-210
-230
f1 (ppm)

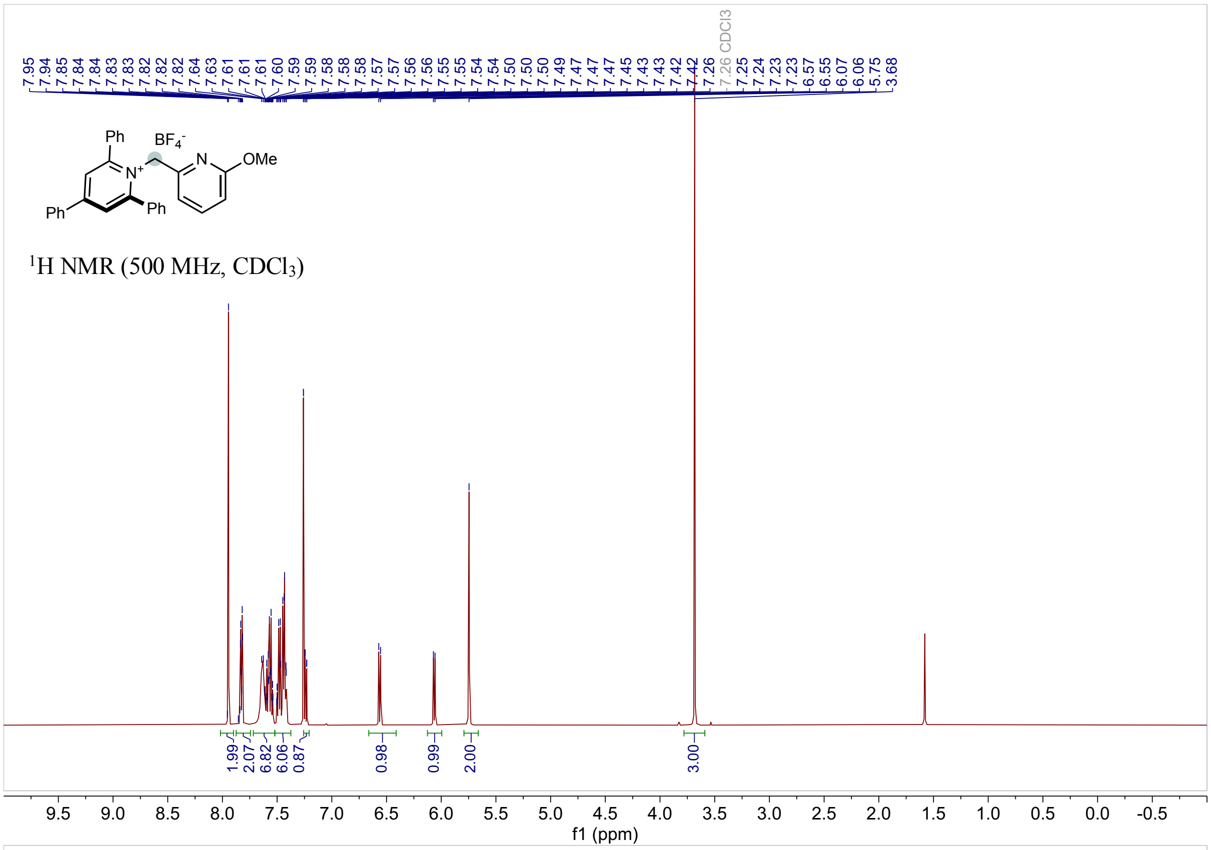

7.95
7.94
7.85
7.84
7.84
7.83
7.83
7.82
7.82
7.82
7.64
7.63
7.61
7.61
7.61
7.60
7.59
7.59
7.58
7.58
7.58
7.57
7.57
7.56
7.56
7.55
7.55
7.54
7.54
7.50
7.50
7.50
7.49
7.47
7.47
7.47
7.45
7.43
7.43
7.42
7.42
7.26
7.26 CDCl3
7.25
7.24
7.23
7.23
6.57
6.55
6.07
6.06
5.75
3.68
Ph
BF4-
N+
N
OMe
Ph
Ph
1H NMR (500 MHz, CDCl3)
1.99
2.07
6.82
6.06
0.87
0.98
0.99
2.00
3.00
9.5
9.0
8.5
8.0
7.5
7.0
6.5
6.0
5.5
5.0
4.5
4.0
3.5
3.0
2.5
2.0
1.5
1.0
0.5
0.0
-0.5
f1 (ppm)

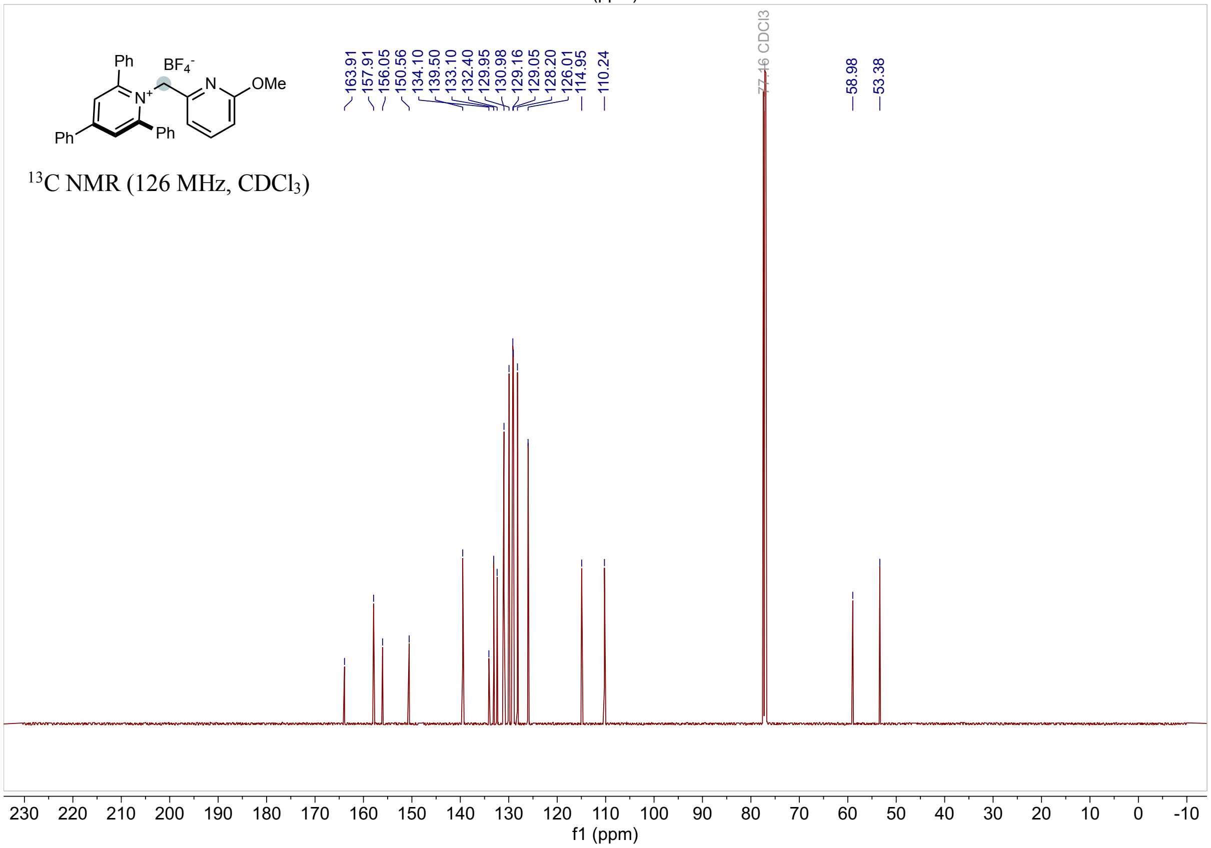

Ph
BF4-
N+
N
OMe
Ph
Ph
13C NMR (126 MHz, CDCl3)
163.91
157.91
156.05
150.56
134.10
139.50
133.10
132.40
129.95
130.98
129.16
129.05
128.20
126.01
114.95
110.24
77.16 CDCl3
58.98
53.38
230
220
210
200
190
180
170
160
150
140
130
120
110
100
90
80
70
60
50
40
30
20
10
0
-10
f1 (ppm)

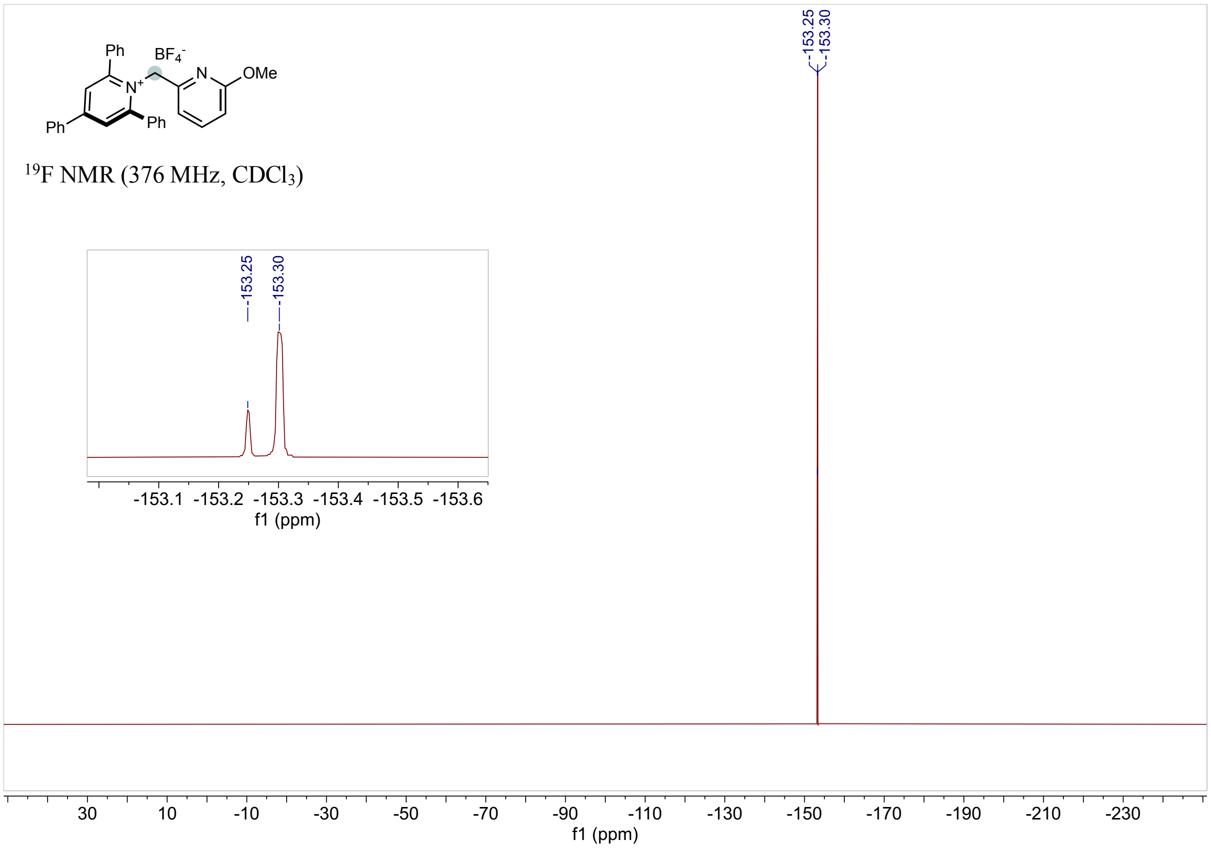
-153.25
-153.30
Ph
BF4-
N
OMe
N+
Ph
Ph
19F NMR (376 MHz, CDCl3)
-153.1 -153.2 -153.3 -153.4 -153.5 -153.6
f1 (ppm)
30 10 -10 -30 -50 -70 -90 -110 -130 -150 -170 -190 -210 -230
f1 (ppm)

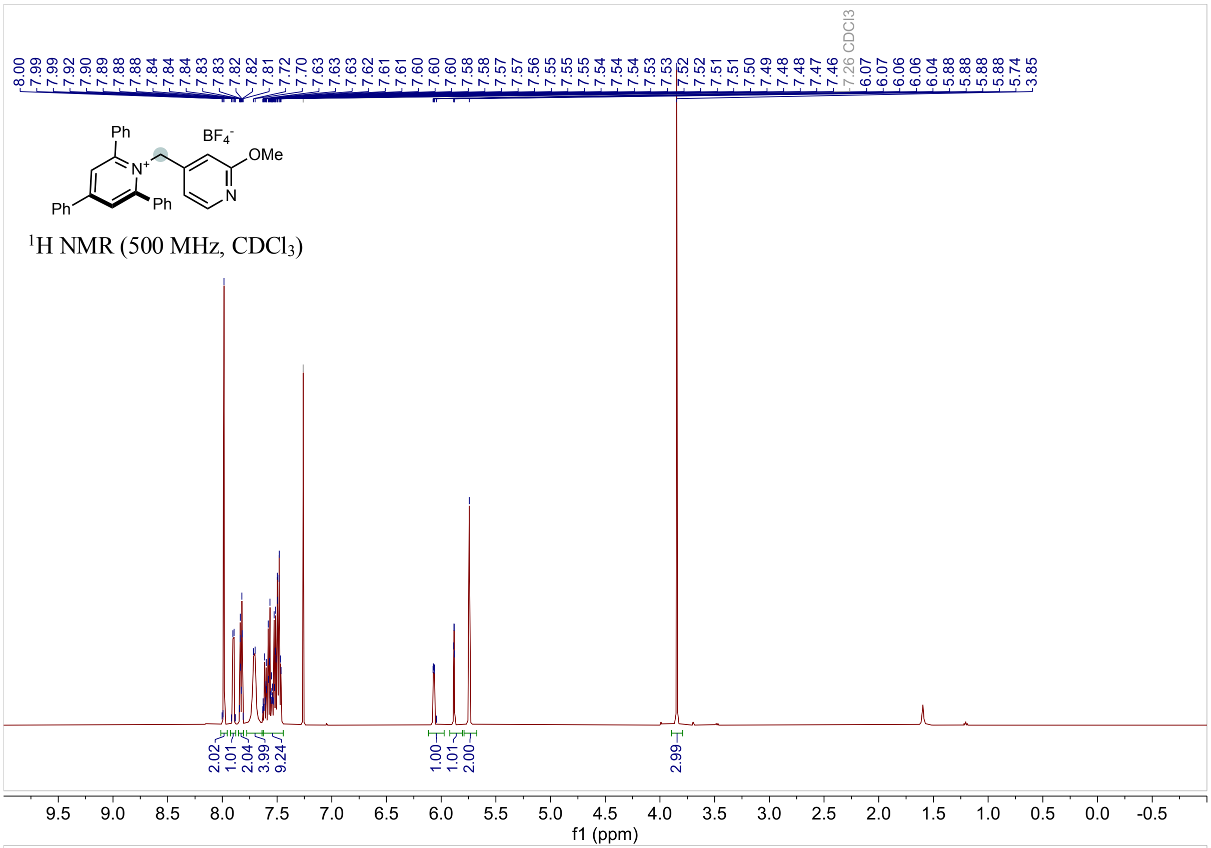

8.00 7.99 7.99 7.92 7.90 7.89 7.88 7.84 7.84 7.84 7.83 7.83 7.82 7.82 7.81 7.72 7.70 7.63 7.63 7.62 7.61 7.61 7.60 7.60 7.60 7.58 7.57 7.57 7.56 7.55 7.55 7.54 7.54 7.53 7.53 7.52 7.52 7.51 7.51 7.50 7.49 7.48 7.48 7.47 7.46
7.26 CDCl3
6.07 6.07 6.06 6.06 6.04 5.88 5.88 5.88 5.88 5.74 3.85
Ph
BF4-
OMe
N+
N
Ph
Ph
1H NMR (500 MHz, CDCl3)
2.02 1.01 2.04 3.99 9.24
1.00 1.01 2.00
2.99
9.5 9.0 8.5 8.0 7.5 7.0 6.5 6.0 5.5 5.0 4.5 4.0 3.5 3.0 2.5 2.0 1.5 1.0 0.5 0.0 -0.5
f1 (ppm)

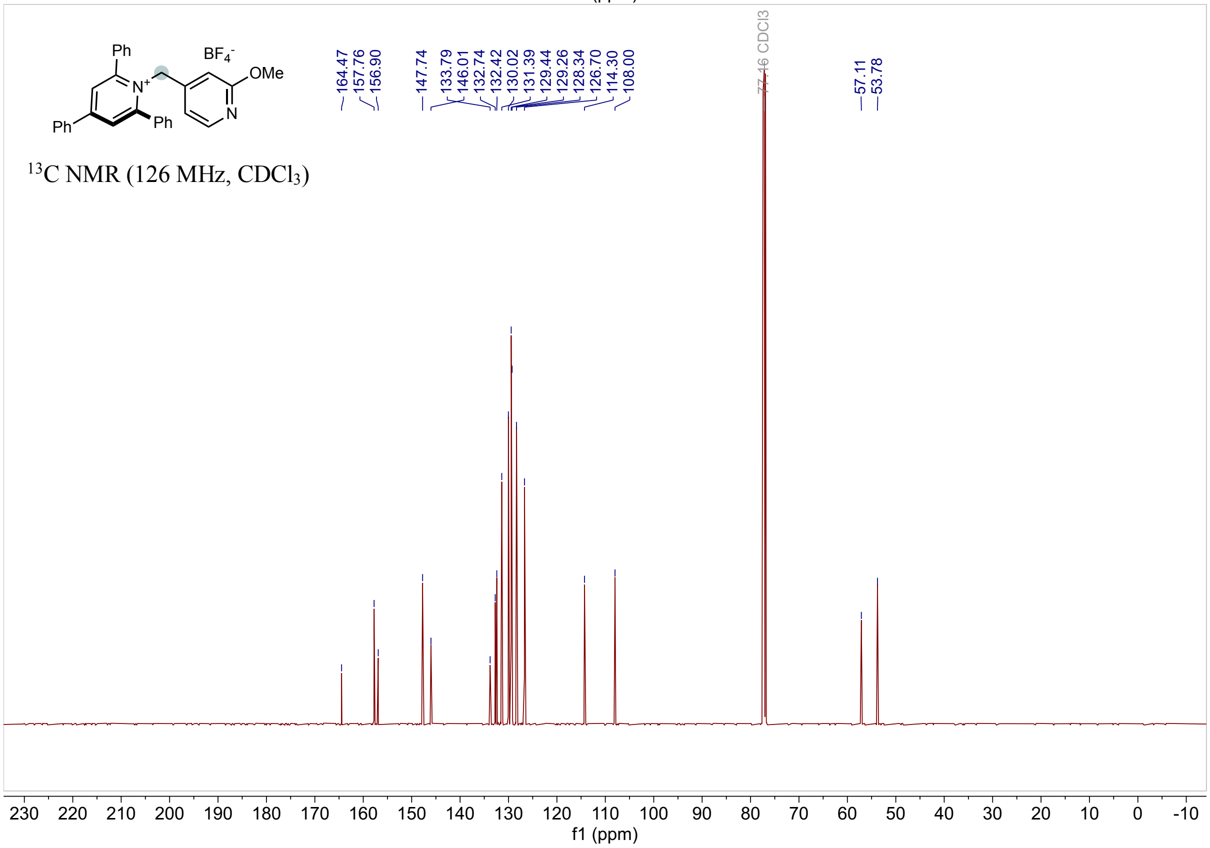

Ph
BF4-
OMe
N+
N
Ph
Ph
13C NMR (126 MHz, CDCl3)
164.47 157.76 156.90
147.74 133.79 146.01 132.74 132.42 130.02 131.39 129.44 129.26 128.34 126.70 114.30 108.00
77.16 CDCl3
57.11 53.78
230 220 210 200 190 180 170 160 150 140 130 120 110 100 90 80 70 60 50 40 30 20 10 0 -10
f1 (ppm)

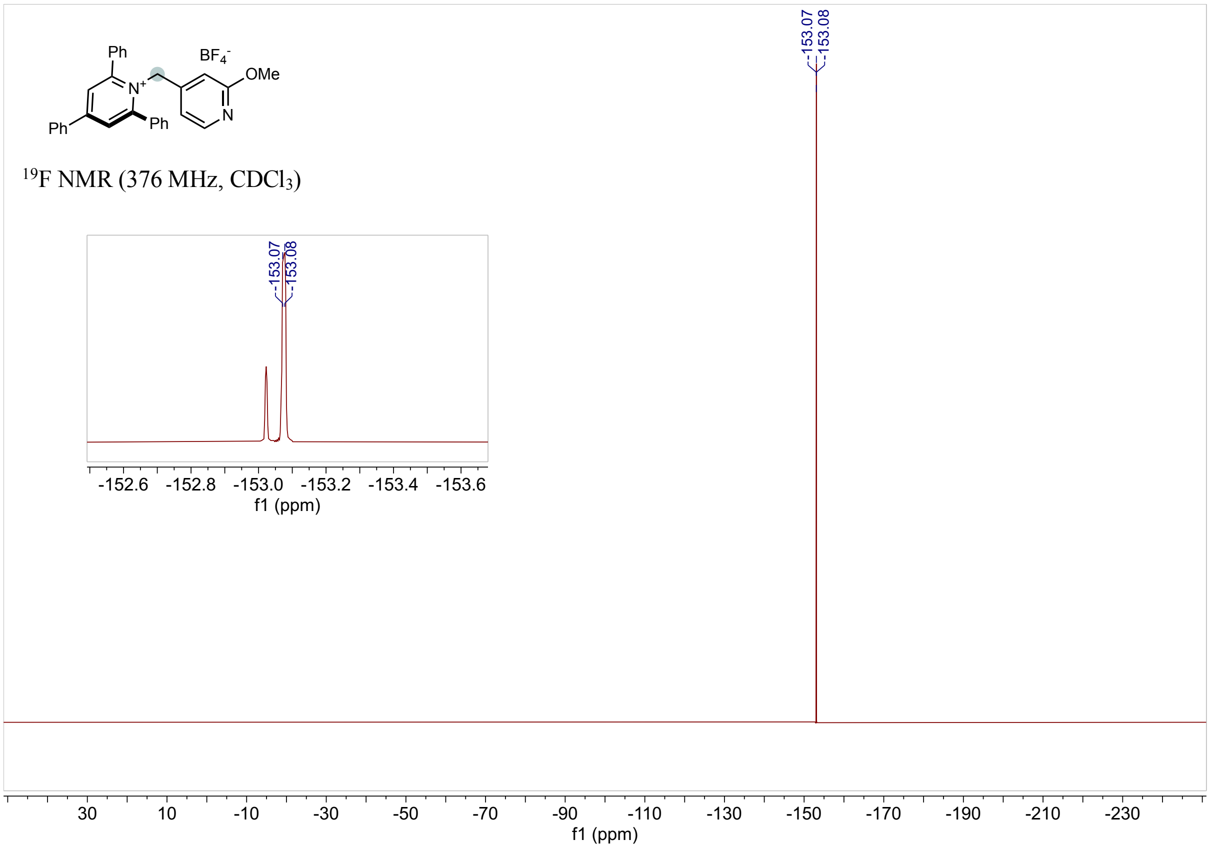

Ph
BF4-
OMe
N+
Ph
Ph
N
19F NMR (376 MHz, CDCl3)
-153.07
-153.08
-152.6
-152.8
-153.0
-153.2
-153.4
-153.6
f1 (ppm)
30
10
-10
-30
-50
-70
-90
-110
-130
-150
-170
-190
-210
-230
f1 (ppm)

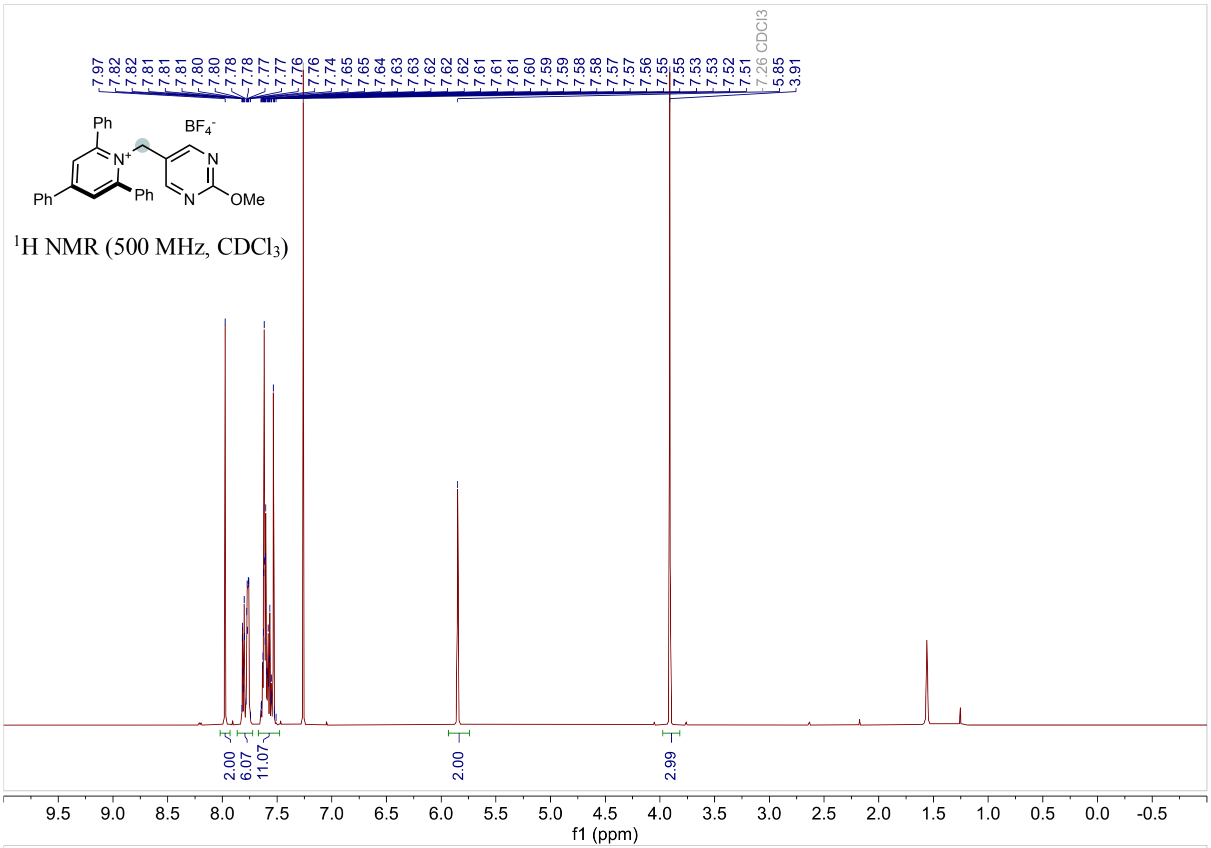
7.97
7.82
7.81
7.81
7.81
7.80
7.78
7.78
7.77
7.77
7.76
7.76
7.74
7.65
7.65
7.64
7.63
7.63
7.62
7.62
7.62
7.61
7.61
7.61
7.60
7.59
7.59
7.58
7.58
7.57
7.57
7.56
7.55
7.55
7.53
7.53
7.52
7.51
7.26 CDCl3
5.85
3.91
Ph
BF4-
N+
N
Ph
Ph
N
OMe
1H NMR (500 MHz, CDCl3)
2.00
6.07
11.07
2.00
2.99
f1 (ppm)
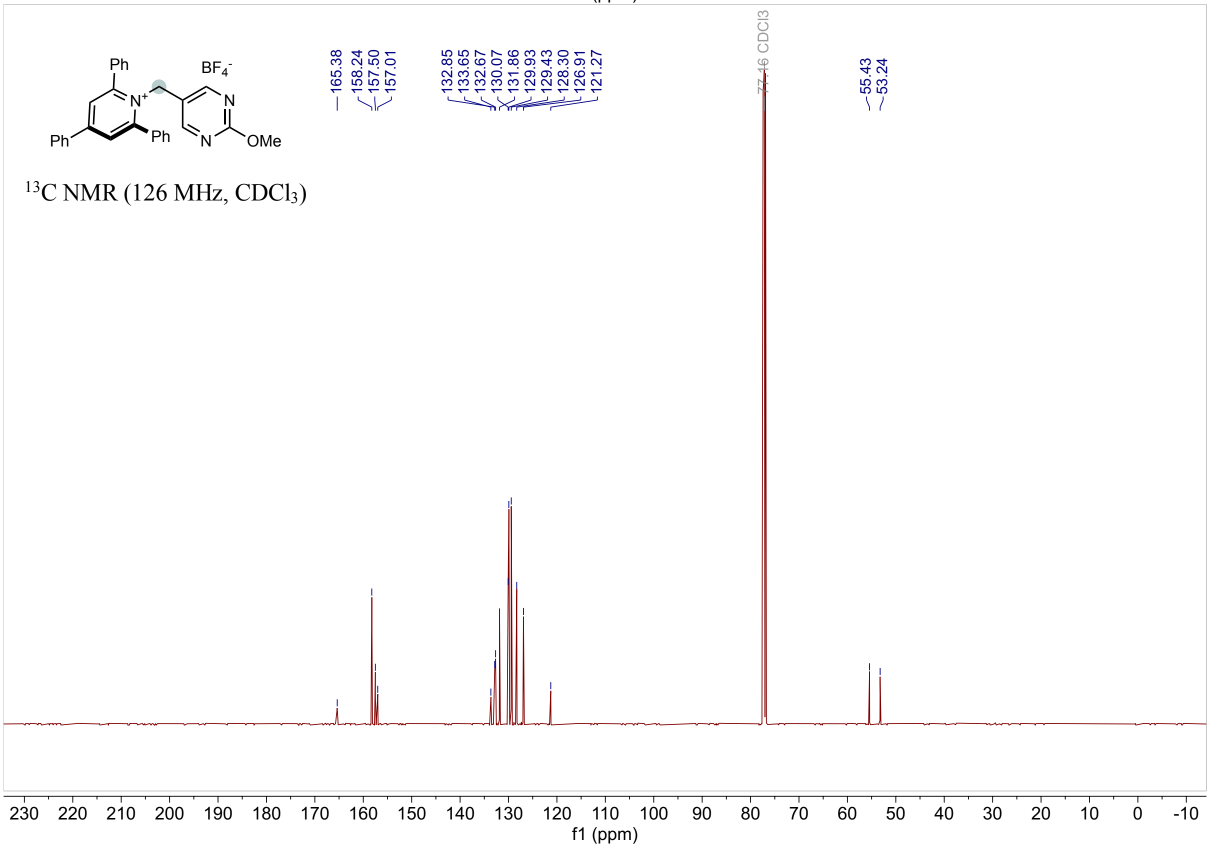
165.38
158.24
157.50
157.01
132.85
133.65
132.67
130.07
131.86
129.93
129.43
128.30
126.91
121.27
77.16 CDCl3
55.43
53.24
Ph
BF4-
N+
N
Ph
Ph
N
OMe
13C NMR (126 MHz, CDCl3)
f1 (ppm)

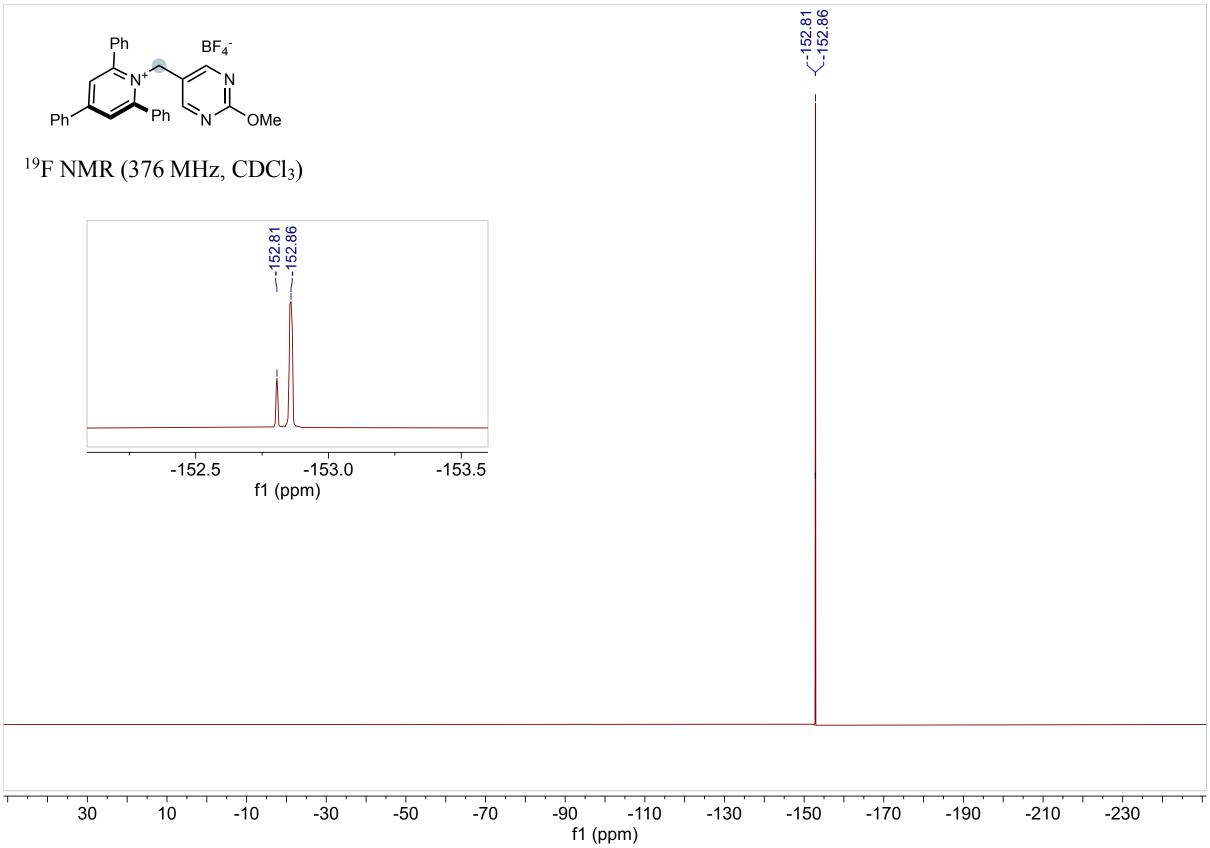

^{19}F NMR (376 MHz, $CDCl_3$)

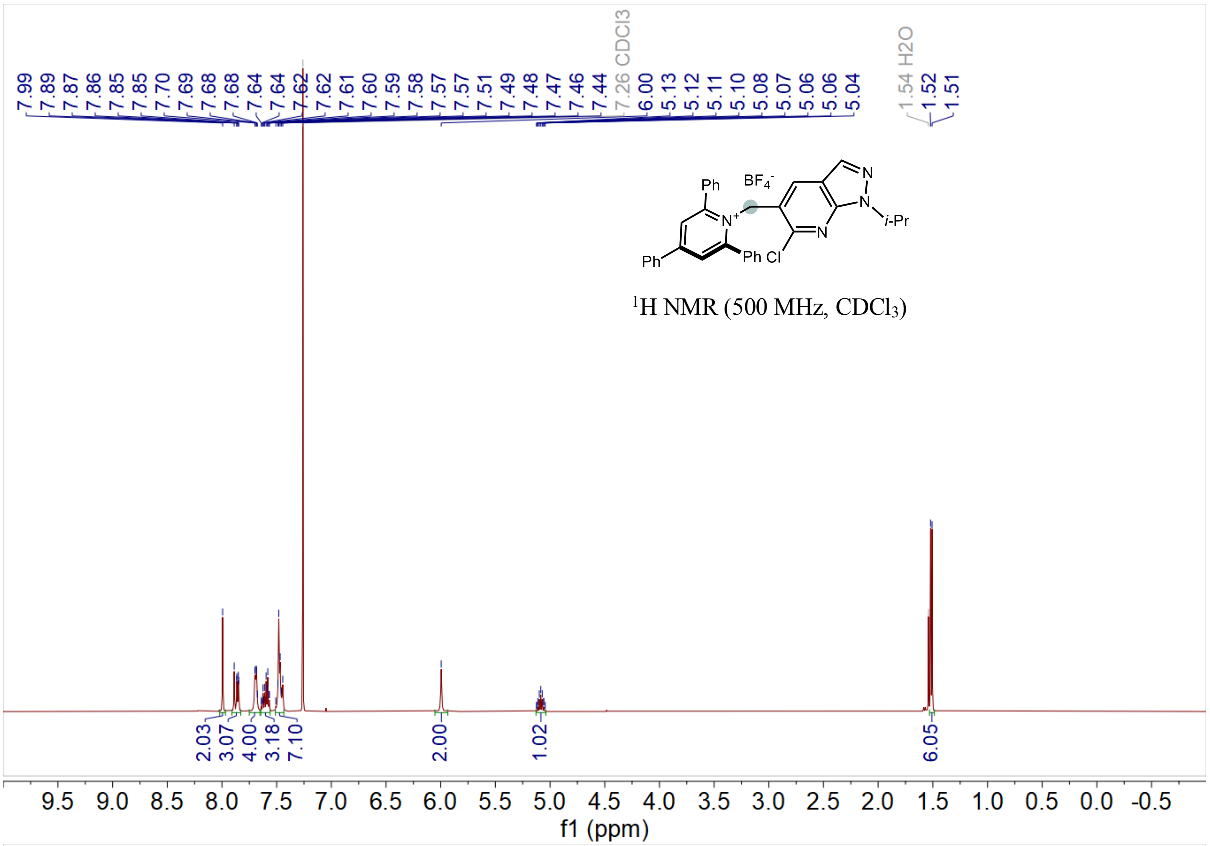
7.99
7.89
7.87
7.86
7.85
7.85
7.70
7.69
7.68
7.68
7.64
7.64
7.62
7.62
7.61
7.60
7.59
7.58
7.57
7.57
7.51
7.49
7.48
7.47
7.46
7.44
7.26 CDCl3
6.00
5.13
5.12
5.11
5.10
5.08
5.07
5.06
5.04
1.54 H2O
1.52
1.51
BF4-
Ph
N+
Ph
Ph
Cl
N
N
N
i-Pr
1H NMR (500 MHz, CDCl3)
2.03
3.07
4.00
3.18
7.10
2.00
1.02
6.05
9.5 9.0 8.5 8.0 7.5 7.0 6.5 6.0 5.5 5.0 4.5 4.0 3.5 3.0 2.5 2.0 1.5 1.0 0.5 0.0 -0.5
f1 (ppm)

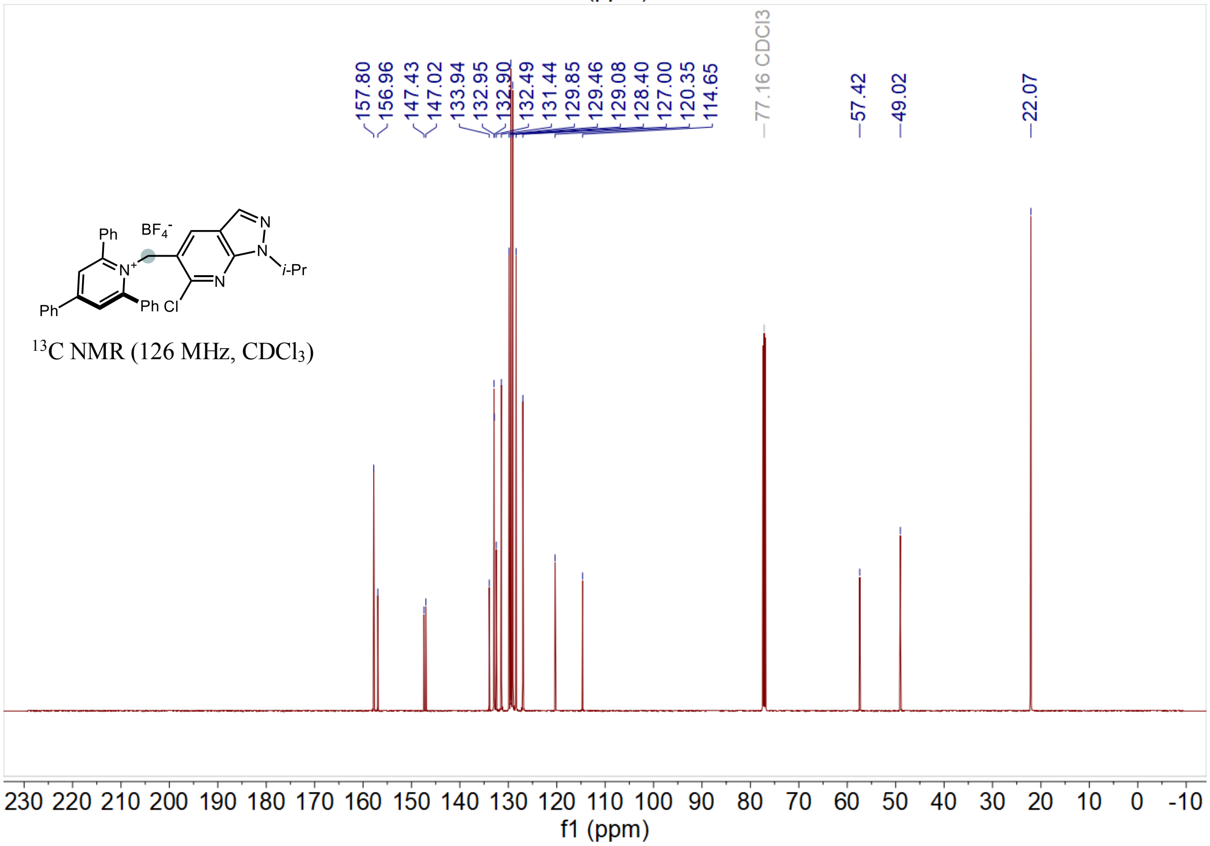
157.80
156.96
147.43
147.02
133.94
132.95
132.90
132.49
131.44
129.85
129.46
129.08
128.40
127.00
120.35
114.65
77.16 CDCl3
57.42
49.02
22.07
BF4-
Ph
N+
Ph
Ph
Cl
N
N
N
i-Pr
13C NMR (126 MHz, CDCl3)
230 220 210 200 190 180 170 160 150 140 130 120 110 100 90 80 70 60 50 40 30 20 10 0 -10
f1 (ppm)

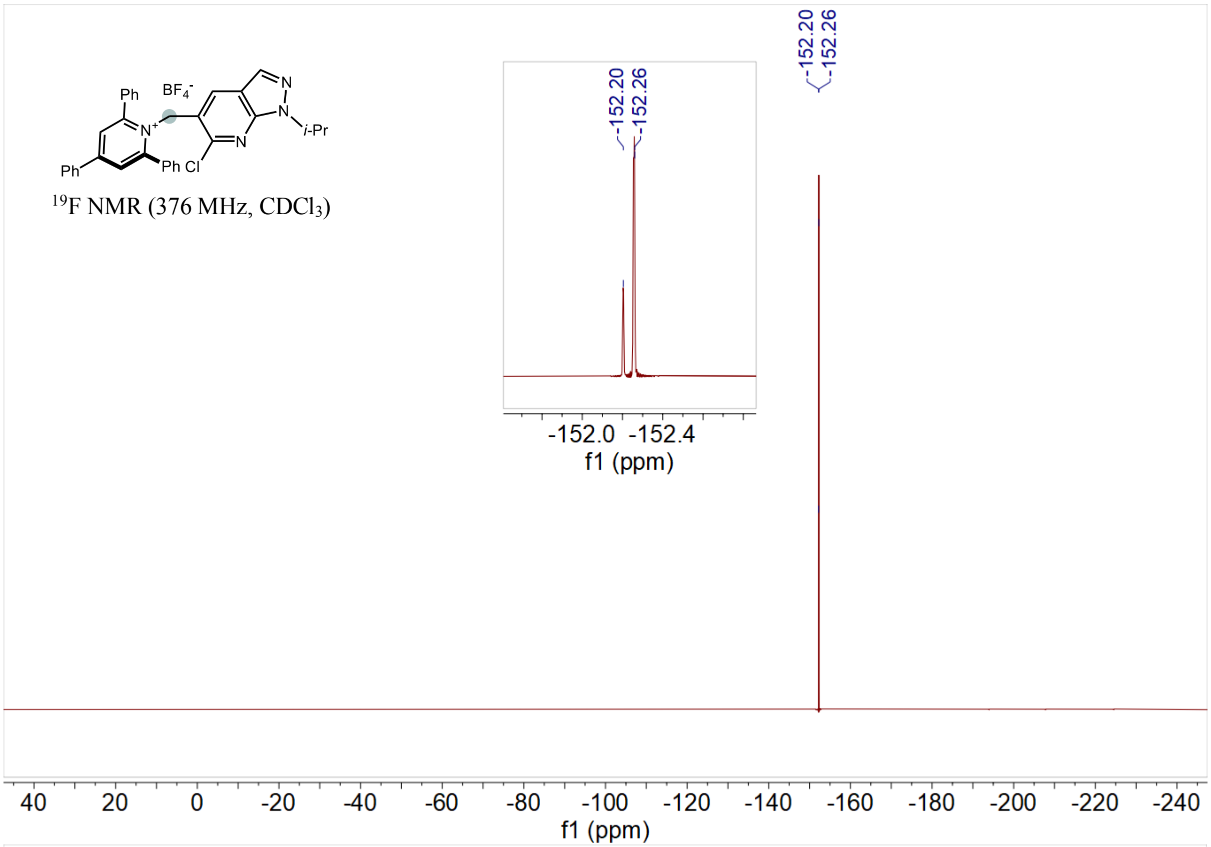

Ph
BF4-
N+
N
N
i-Pr
Ph Cl
Ph
19F NMR (376 MHz, CDCl3)
-152.20
-152.26
-152.0 -152.4
f1 (ppm)
40 20 0 -20 -40 -60 -80 -100 -120 -140 -160 -180 -200 -220 -240

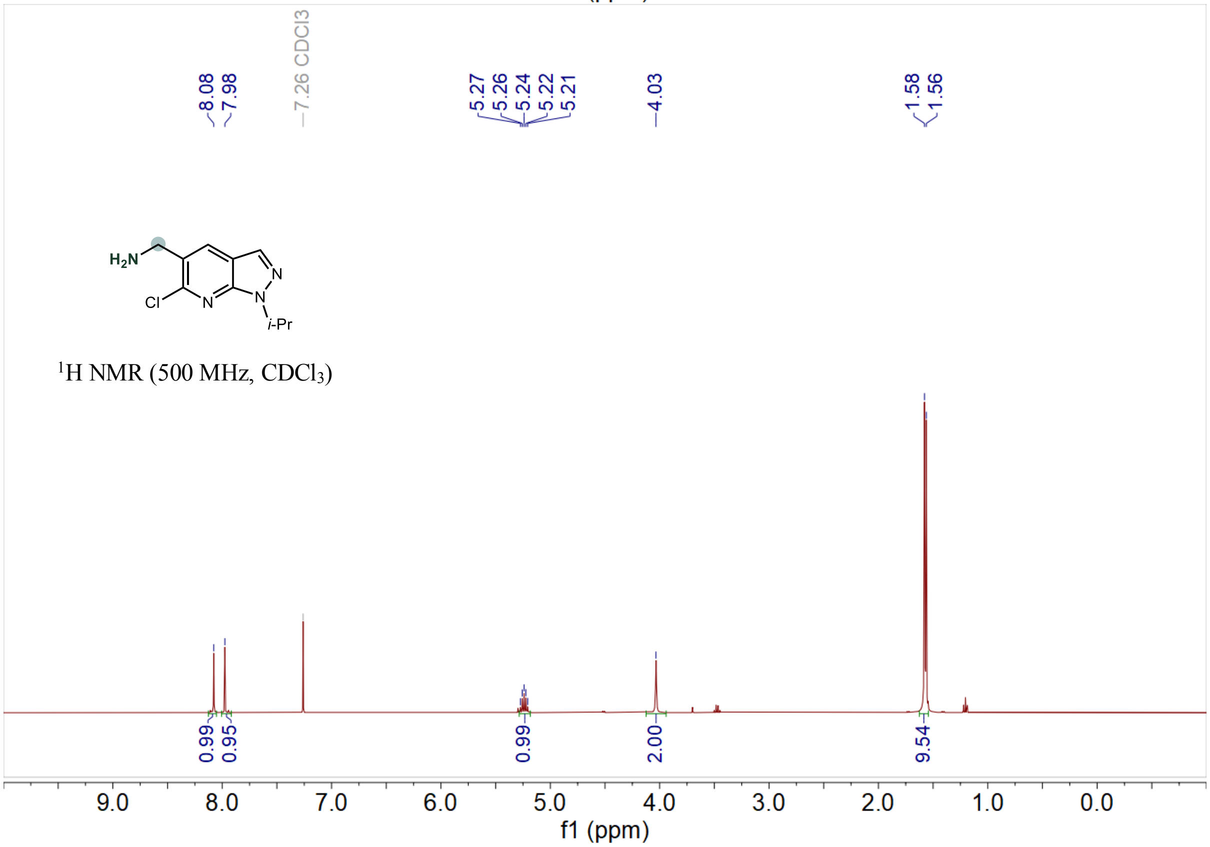

8.08
7.98
7.26 CDCl3
5.27
5.26
5.24
5.22
5.21
4.03
1.58
1.56
H2N
Cl
N
N
N
i-Pr
1H NMR (500 MHz, CDCl3)
0.99
0.95
0.99
2.00
9.54
9.0 8.0 7.0 6.0 5.0 4.0 3.0 2.0 1.0 0.0
f1 (ppm)

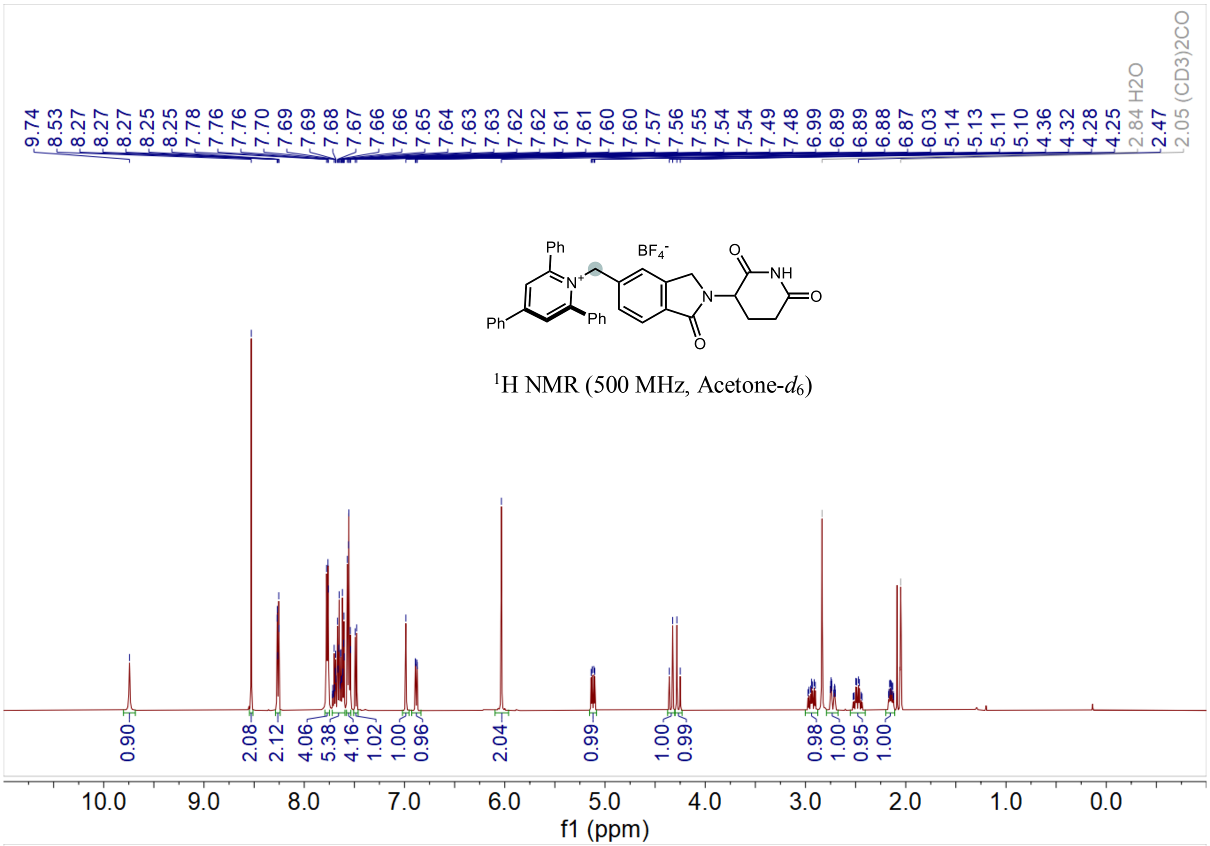
9.74 8.53 8.27 8.27 8.27 8.25 8.25 7.78 7.76 7.76 7.70 7.69 7.69 7.68 7.67 7.66 7.66 7.65 7.64 7.63 7.63 7.62 7.62 7.61 7.61 7.60 7.60 7.57 7.56 7.55 7.54 7.54 7.49 7.48 6.99 6.89 6.88 6.87 6.03 5.14 5.13 5.11 5.10 4.36 4.32 4.28 4.25 2.84 H2O 2.47 2.05 (CD3)2CO
Ph
BF_4^-
O
NH
N$^+$
N
O
Ph
Ph
O
^{1}H NMR (500 MHz, Acetone-d_6)
0.90 2.08 2.12 4.06 5.38 4.16 1.02 1.00 0.96 2.04 0.99 1.00 0.99 0.98 1.00 0.95 1.00
10.0 9.0 8.0 7.0 6.0 5.0 4.0 3.0 2.0 1.0 0.0
f1 (ppm)

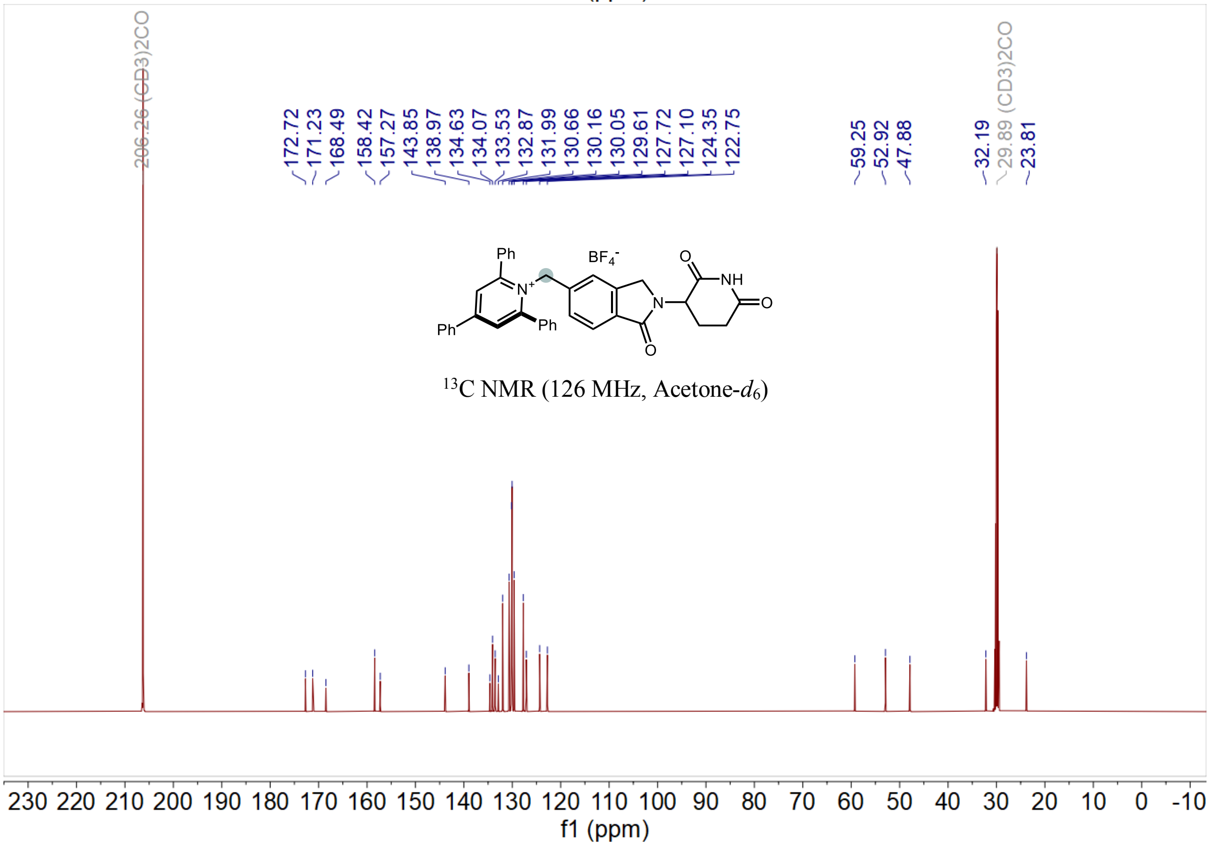
206.26 (CD3)2CO
172.72 171.23 168.49 158.42 157.27 143.85 138.97 134.63 134.07 133.53 132.87 131.99 130.66 130.16 130.05 129.61 127.72 127.10 124.35 122.75
59.25 52.92 47.88
32.19 29.89 (CD3)2CO 23.81
Ph
BF_4^-
O
NH
N$^+$
N
O
Ph
Ph
O
^{13}C NMR (126 MHz, Acetone-d_6)
230 220 210 200 190 180 170 160 150 140 130 120 110 100 90 80 70 60 50 40 30 20 10 0 -10
f1 (ppm)

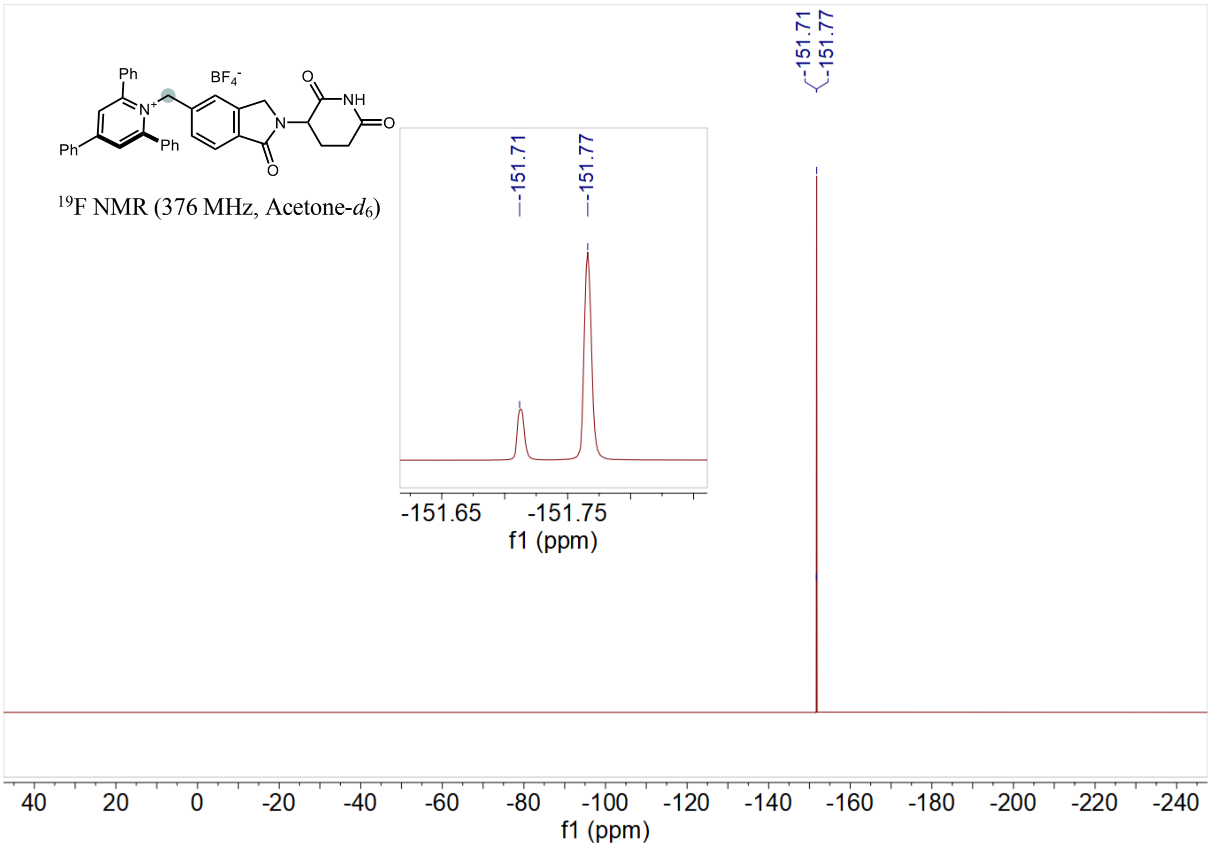

-151.71
-151.77
Ph
BF4-
O
NH
N+
N
O
Ph
Ph
O
19F NMR (376 MHz, Acetone-d6)
-151.65
-151.75
f1 (ppm)
40
20
0
-20
-40
-60
-80
-100
-120
-140
-160
-180
-200
-220
-240

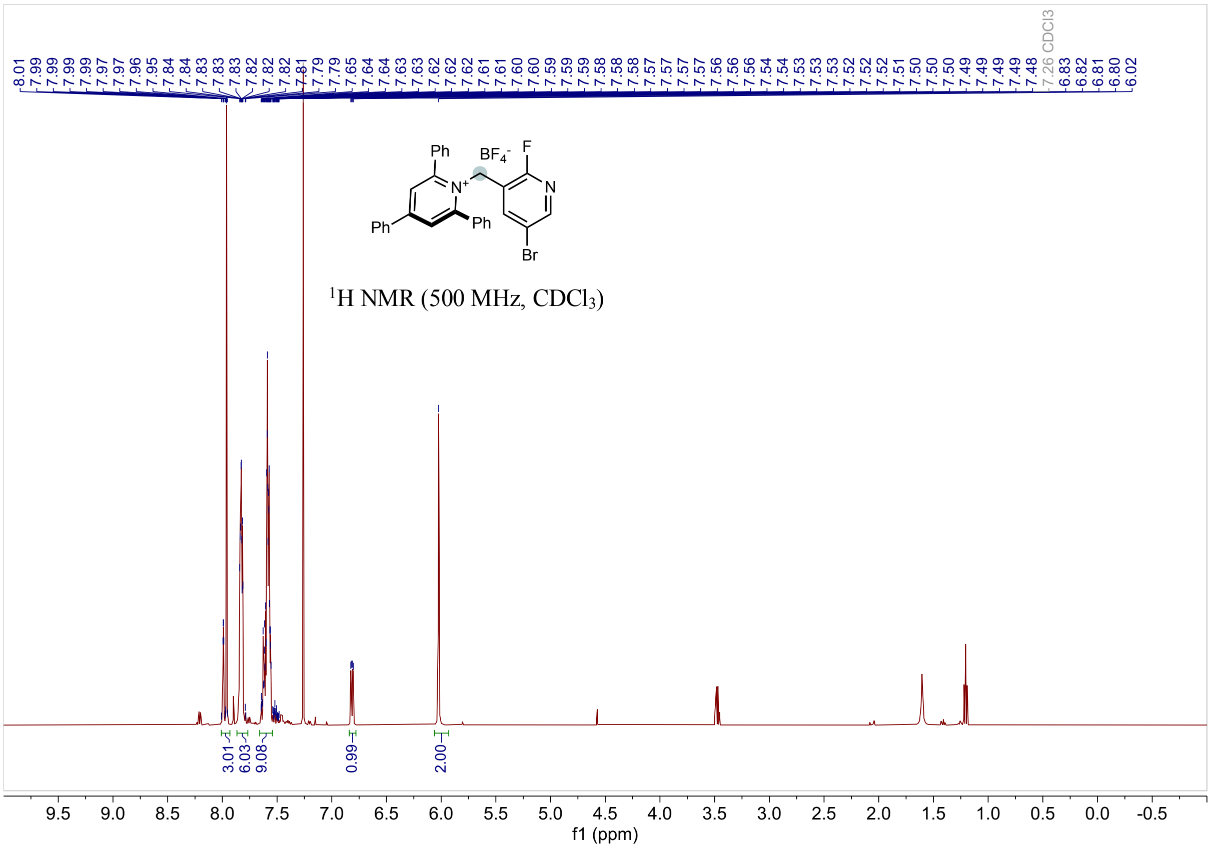

8.01
7.99
7.99
7.99
7.97
7.97
7.96
7.95
7.84
7.83
7.83
7.82
7.82
7.82
7.81
7.79
7.65
7.64
7.64
7.63
7.63
7.62
7.62
7.62
7.61
7.61
7.60
7.60
7.59
7.59
7.59
7.58
7.58
7.58
7.57
7.57
7.57
7.56
7.56
7.56
7.54
7.54
7.53
7.53
7.53
7.52
7.52
7.52
7.51
7.50
7.50
7.49
7.49
7.49
7.48
7.48
7.26 CDCl3
6.83
6.82
6.81
6.80
6.02
Ph
BF4-
F
N+
N
Ph
Ph
Br
1H NMR (500 MHz, CDCl3)
3.01
6.03
9.08
0.99
2.00
9.5
9.0
8.5
8.0
7.5
7.0
6.5
6.0
5.5
5.0
4.5
4.0
3.5
3.0
2.5
2.0
1.5
1.0
0.5
0.0
-0.5
f1 (ppm)

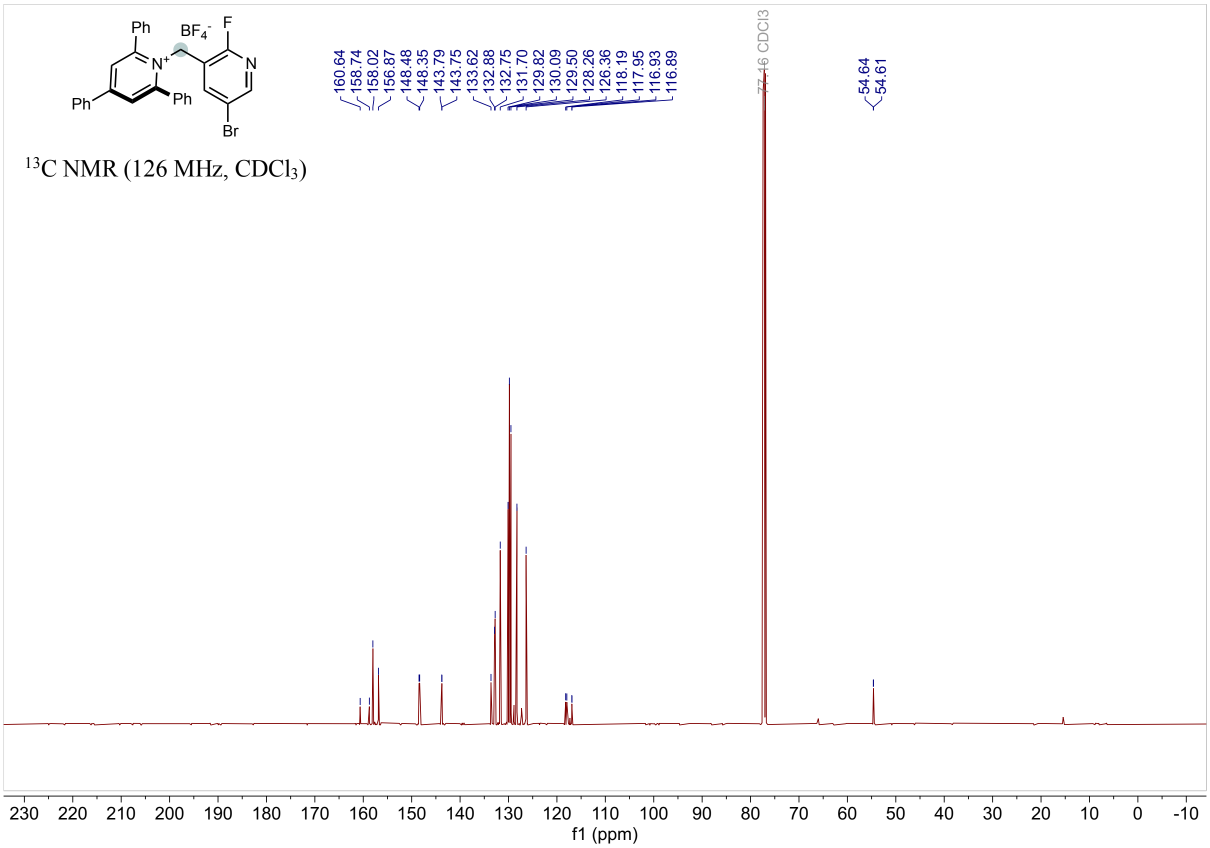

Ph
BF4-
F
N+
N
Ph
Ph
Br
13C NMR (126 MHz, CDCl3)
160.64
158.74
158.02
156.87
148.48
148.35
143.79
143.75
133.62
132.88
132.75
131.70
129.82
130.09
129.50
128.26
126.36
118.19
117.95
116.93
116.89
77.16 CDCl3
54.64
54.61
230
220
210
200
190
180
170
160
150
140
130
120
110
100
90
80
70
60
50
40
30
20
10
0
-10
f1 (ppm)

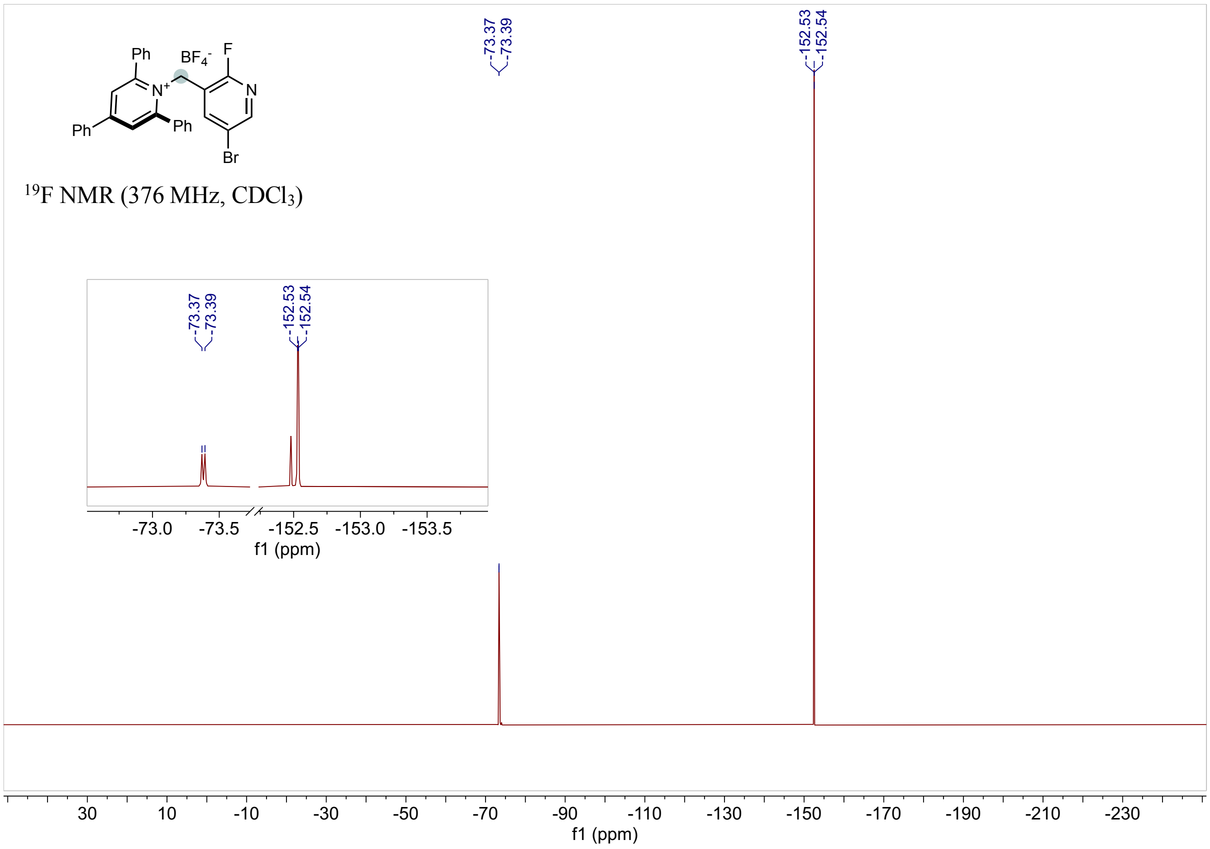

Ph
BF4-
F
N+
N
Ph
Ph
Br
19F NMR (376 MHz, CDCl3)
-73.37
-73.39
-152.53
-152.54
-73.0
-73.5
-152.5
-153.0
-153.5
f1 (ppm)
30
10
-10
-30
-50
-70
-90
-110
-130
-150
-170
-190
-210
-230
f1 (ppm)

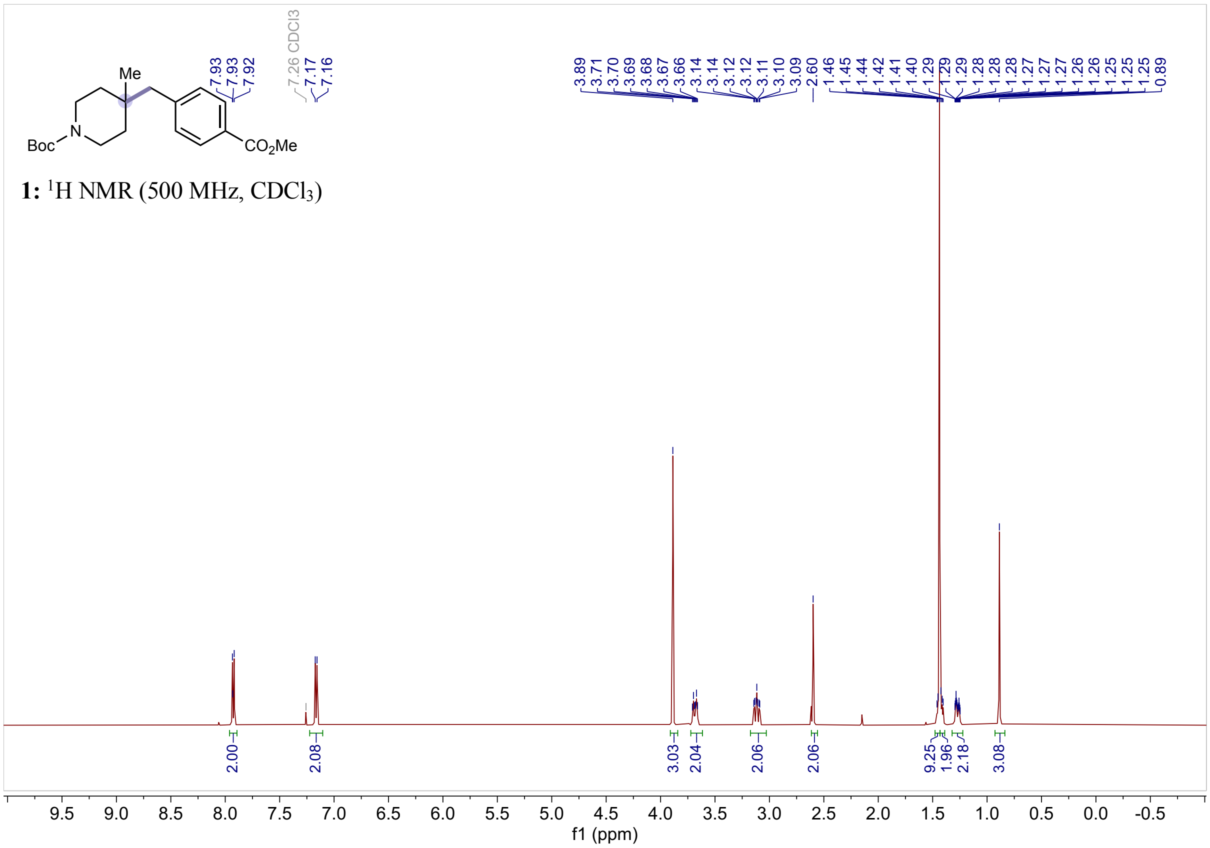

1: ^{1}H NMR (500 MHz, $CDCl_3$)

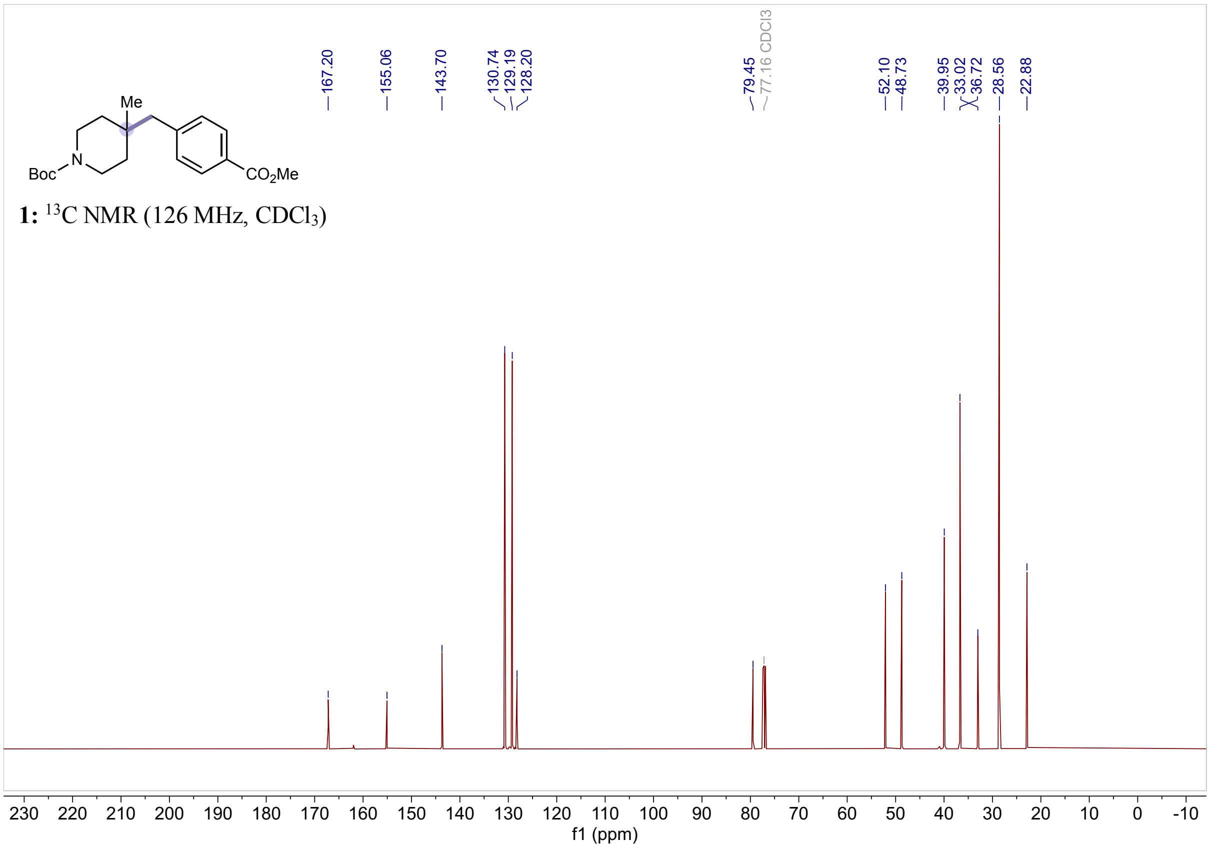

1: ^{13}C NMR (126 MHz, $CDCl_3$)

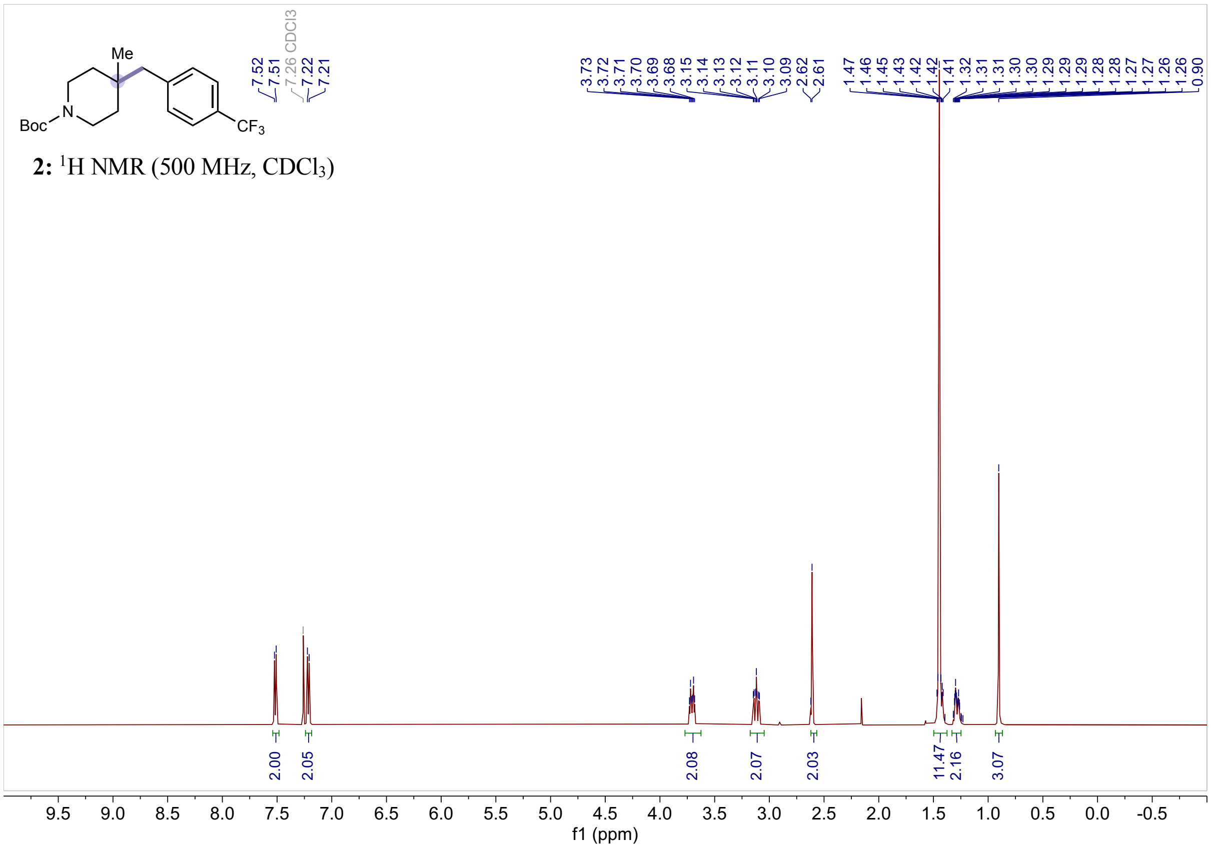

2: ^{1}H NMR (500 MHz, $CDCl_3$)

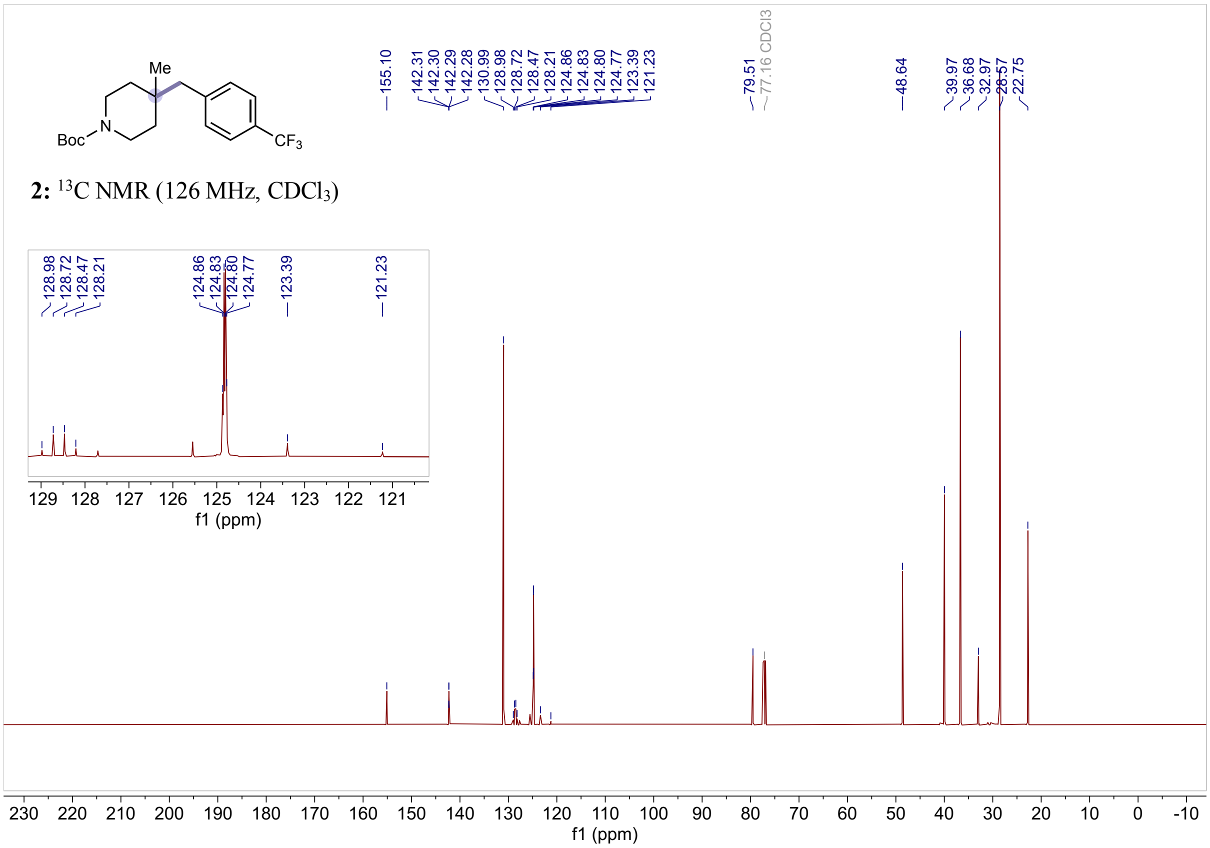

2: ^{13}C NMR (126 MHz, $CDCl_3$)

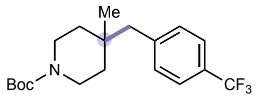

2: ^{19}F NMR (471 MHz, CDCl$_3$)

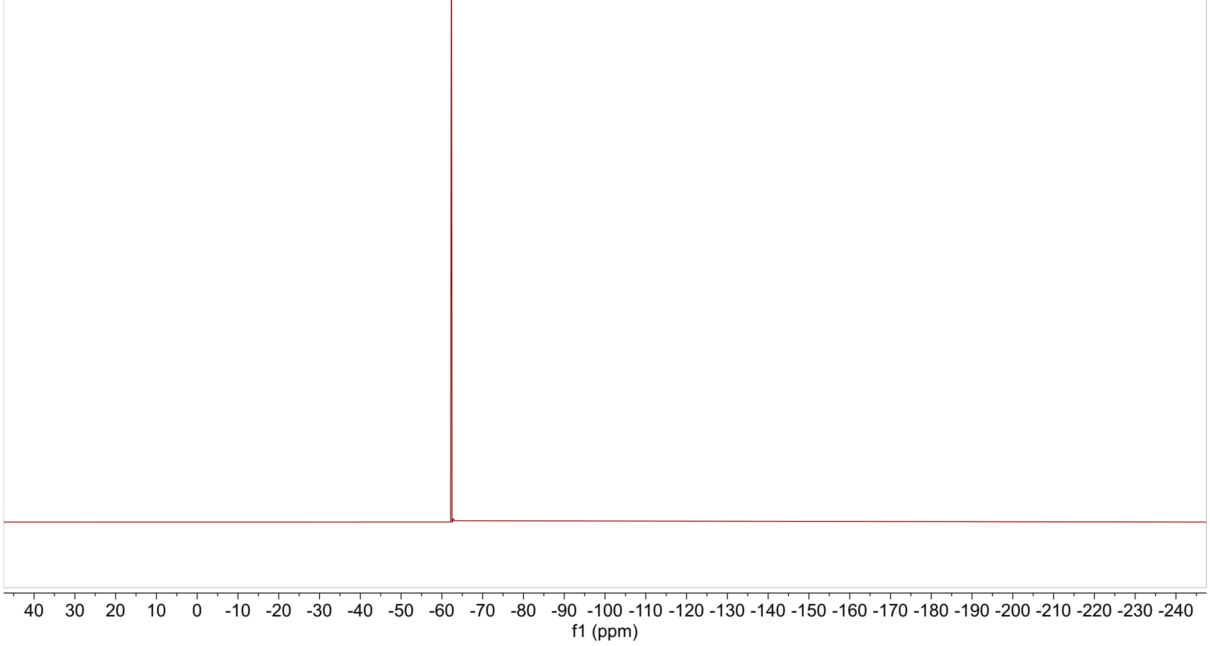

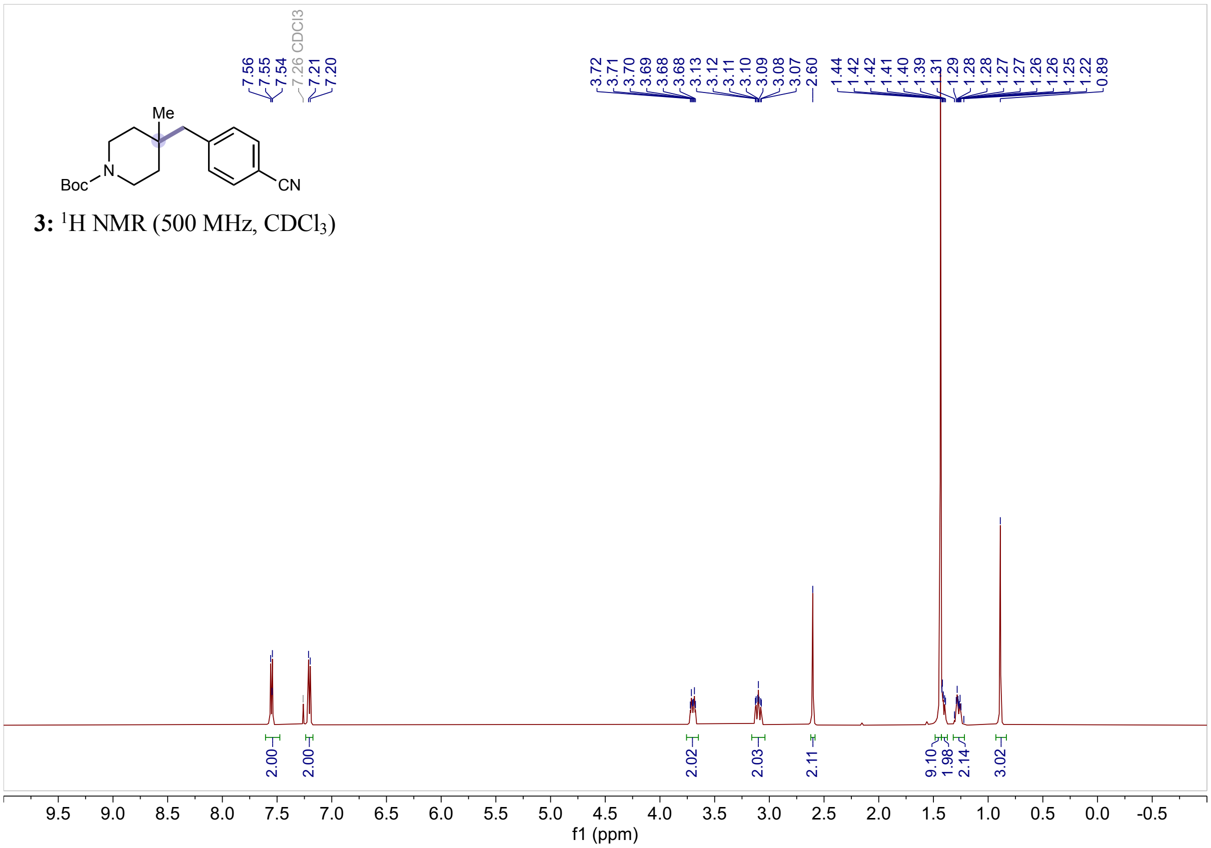

3: ^{1}H NMR (500 MHz, $CDCl_3$)

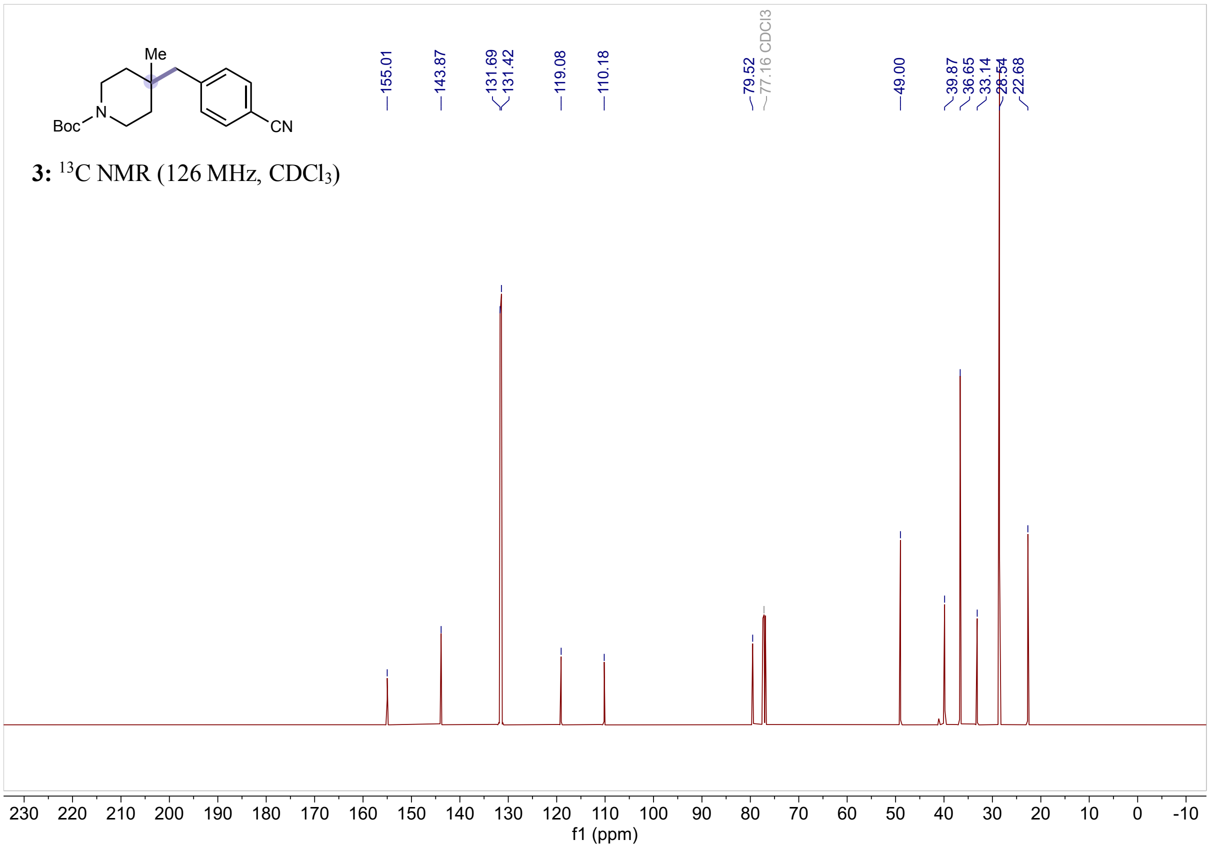

3: ^{13}C NMR (126 MHz, $CDCl_3$)

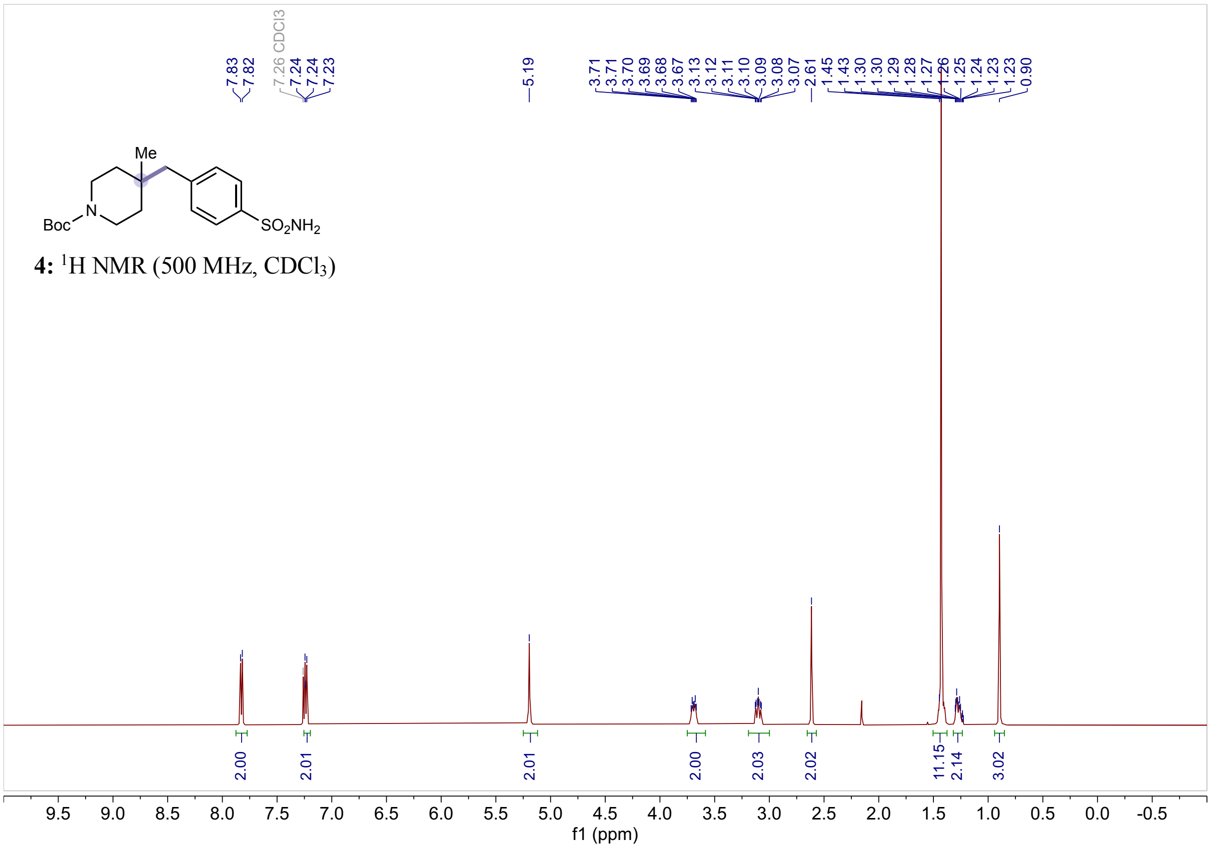

4: ^{1}H NMR (500 MHz, $CDCl_3$)

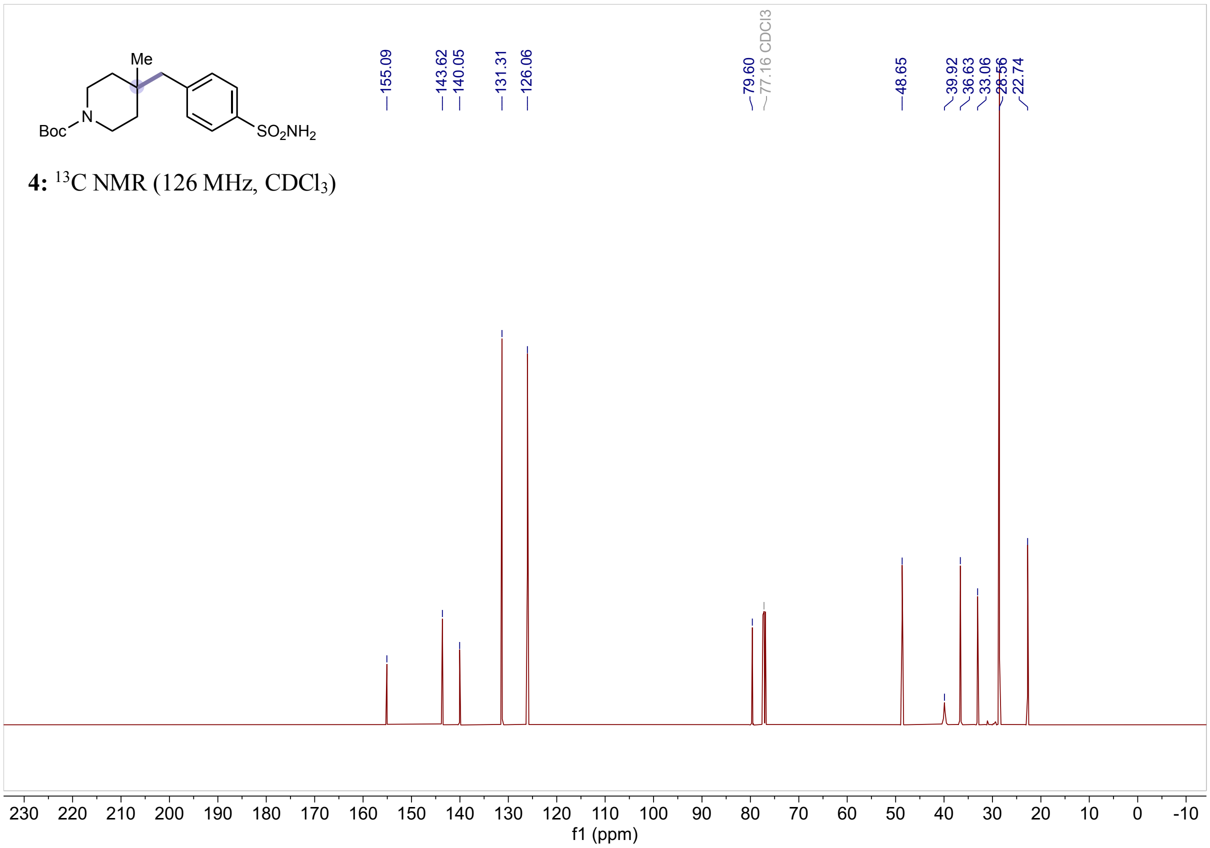

4: ^{13}C NMR (126 MHz, $CDCl_3$)

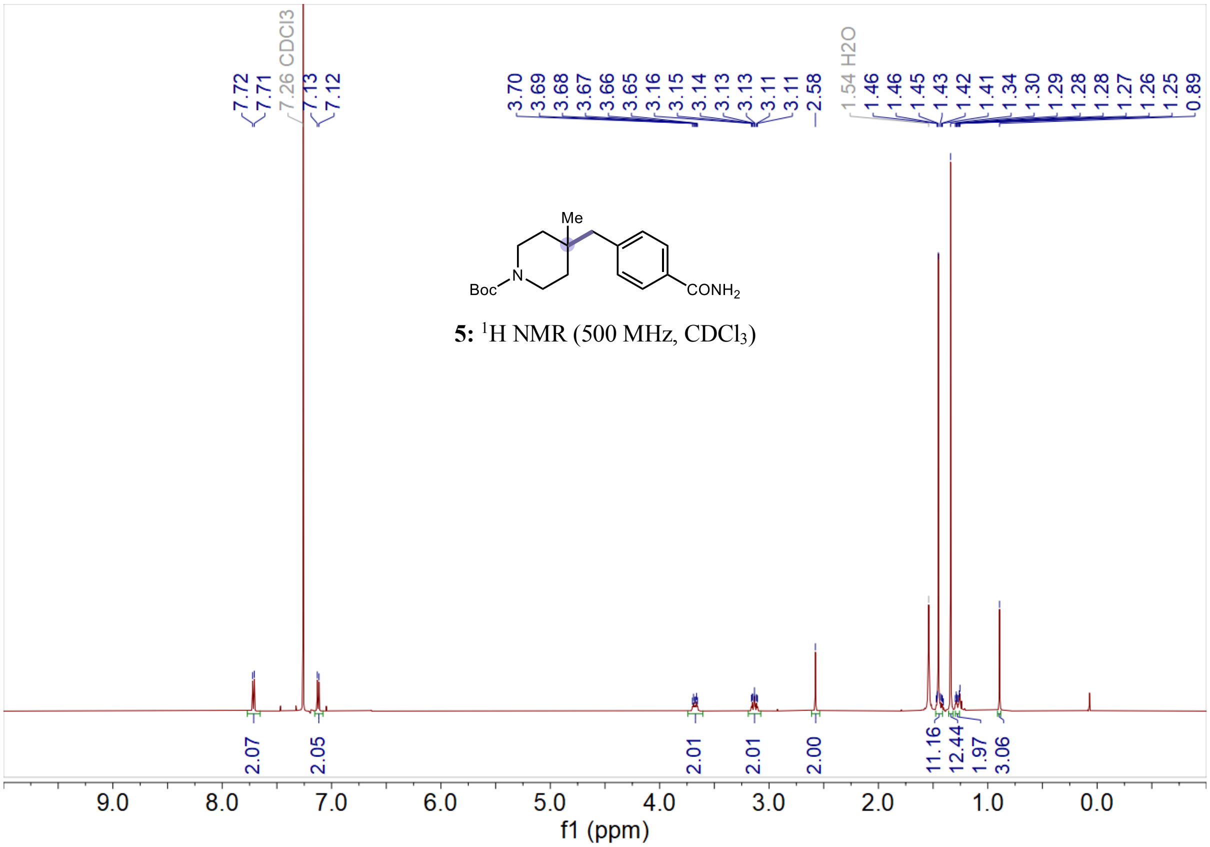

5: ^{1}H NMR (500 MHz, $CDCl_3$)

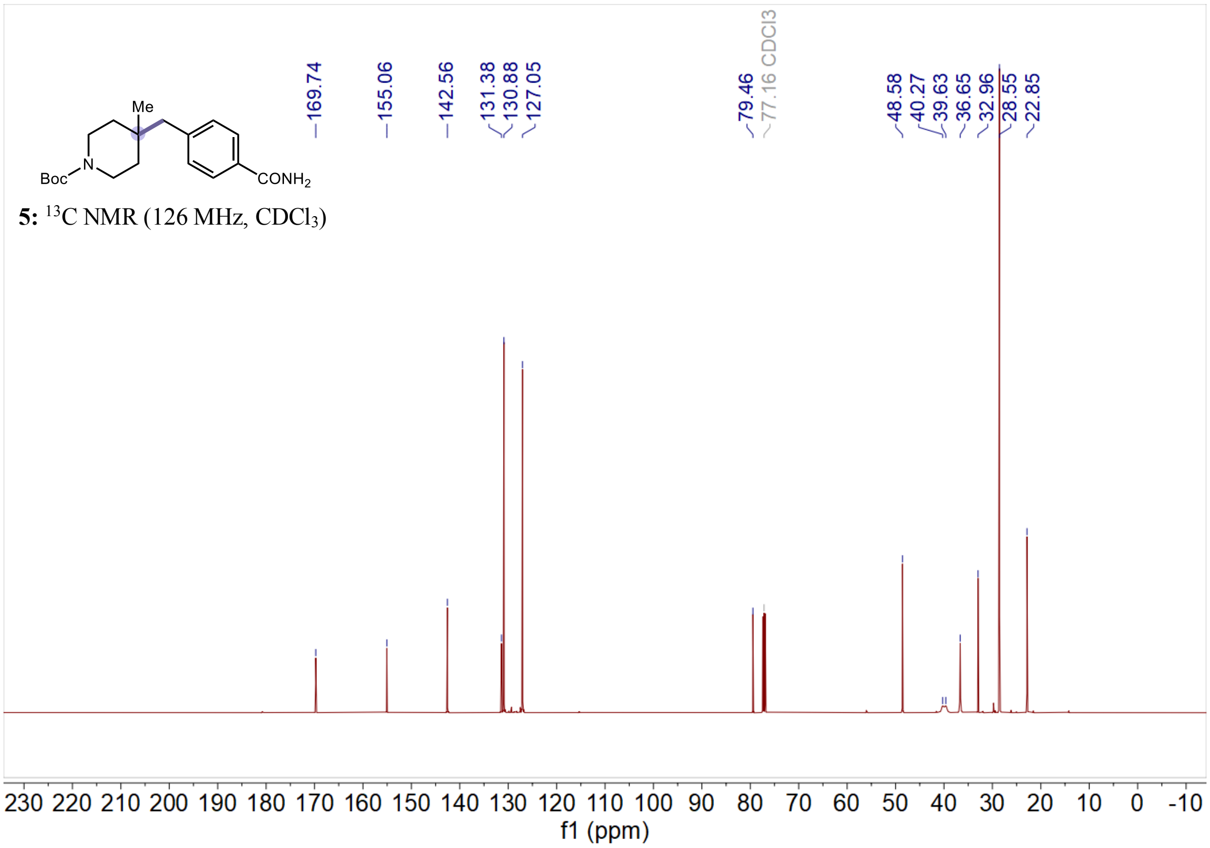

5: ^{13}C NMR (126 MHz, $CDCl_3$)

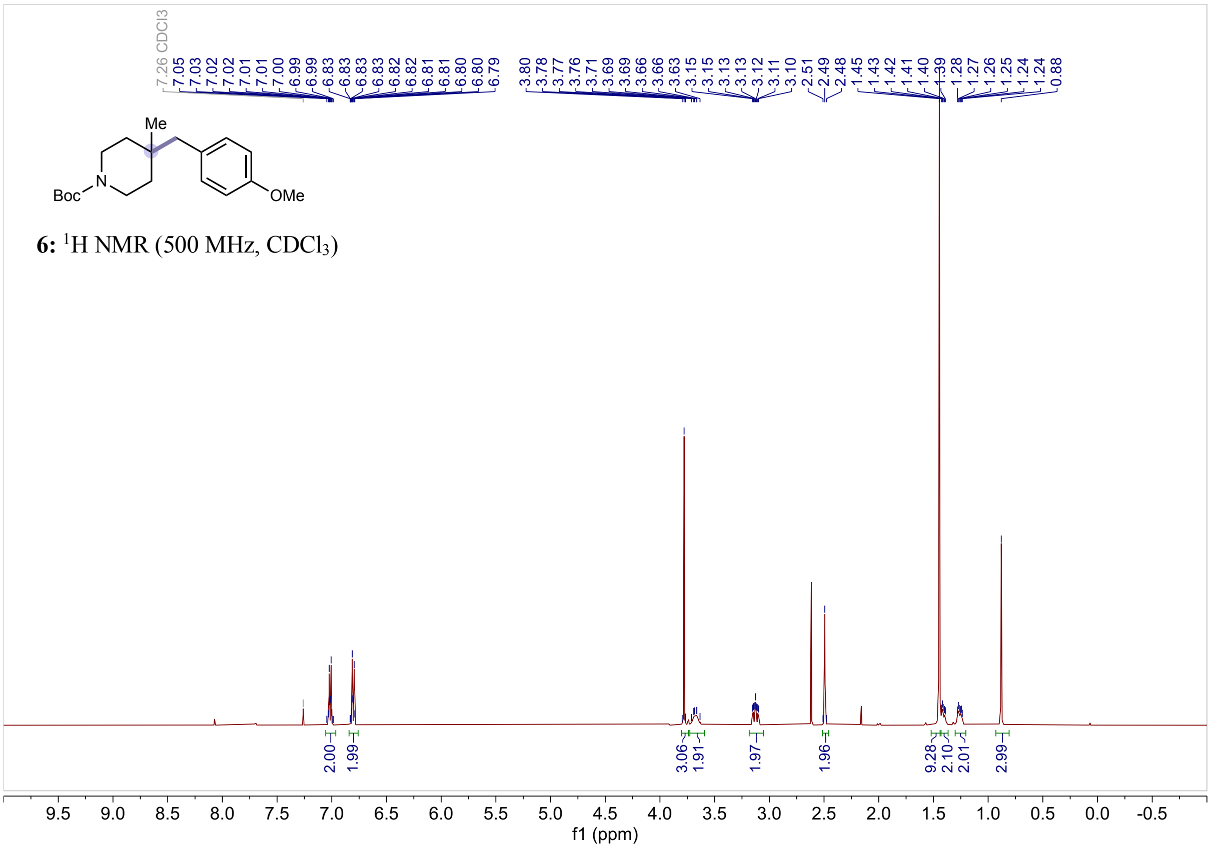

Me
Boc
N
OMe
6: ¹H NMR (500 MHz, CDCl3)
7.26 CDCl3
2.00
1.99
3.06
1.91
1.97
1.96
9.28
2.10
2.01
2.99
f1 (ppm)

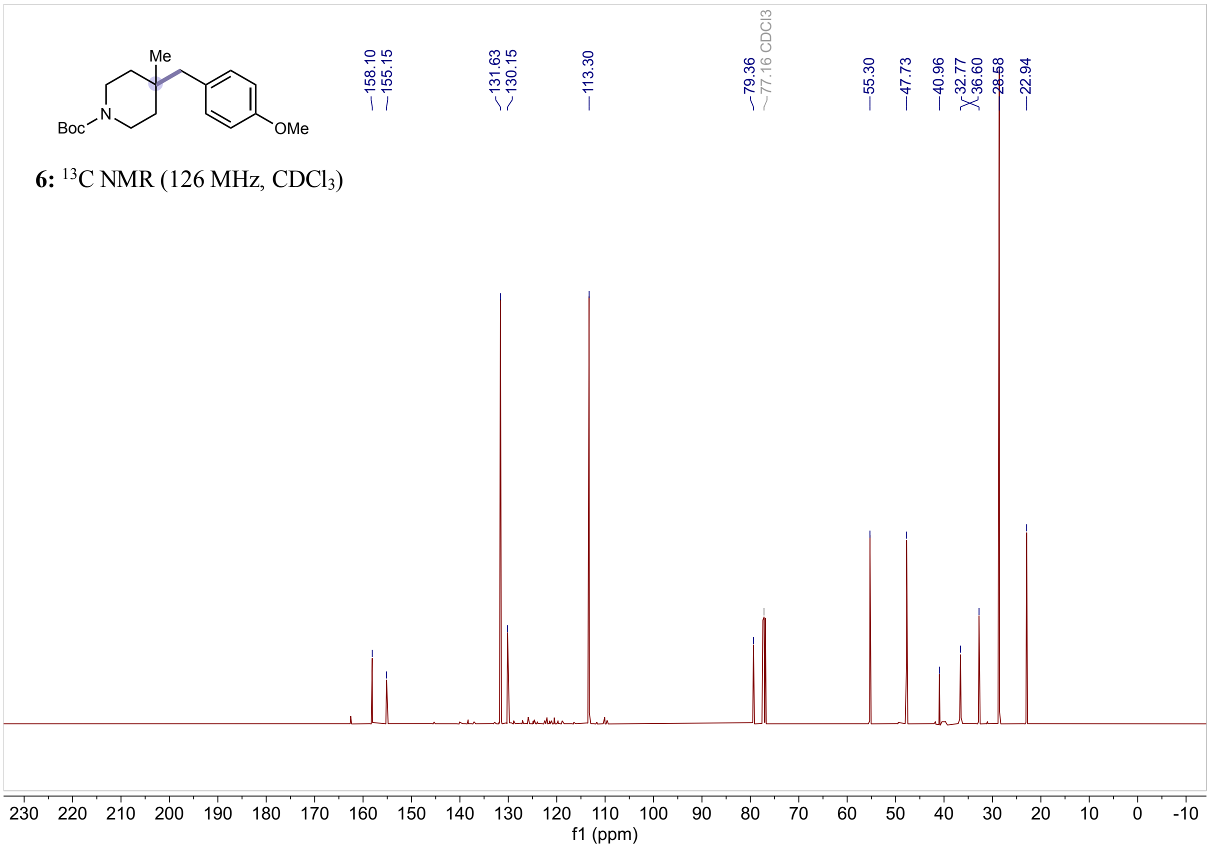

Me
Boc
N
OMe
6: ¹³C NMR (126 MHz, CDCl3)
158.10
155.15
131.63
130.15
113.30
79.36
77.16 CDCl3
55.30
47.73
40.96
36.60
32.77
28.58
22.94
f1 (ppm)

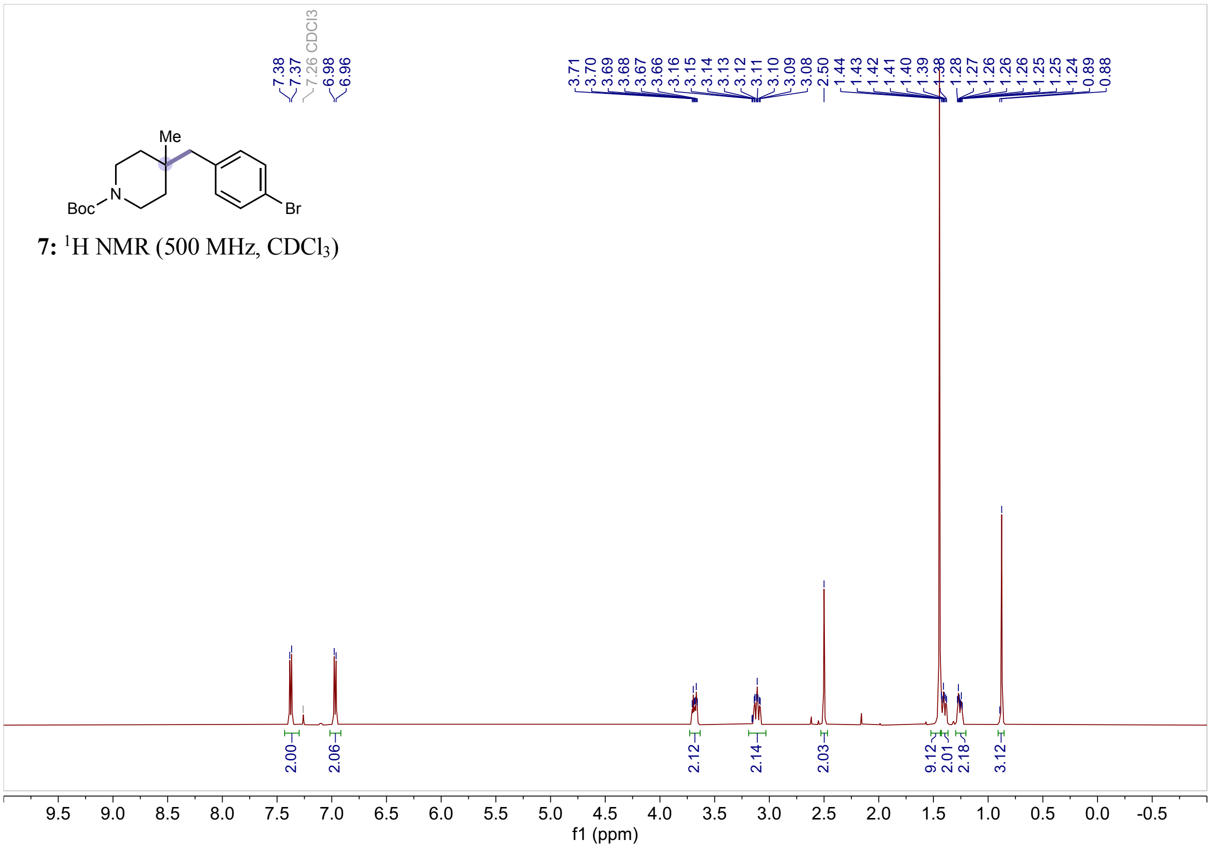

7: ^{1}H NMR (500 MHz, $CDCl_3$)

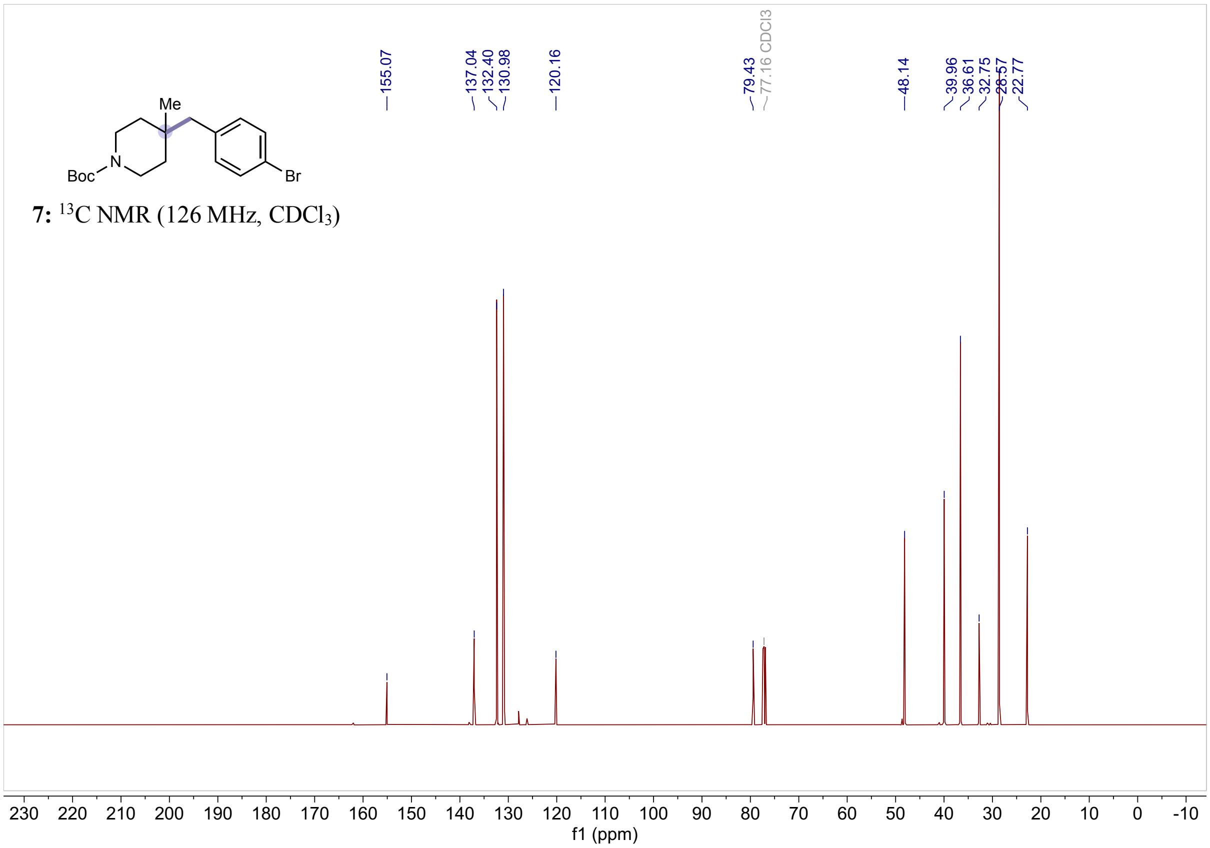

7: ^{13}C NMR (126 MHz, $CDCl_3$)

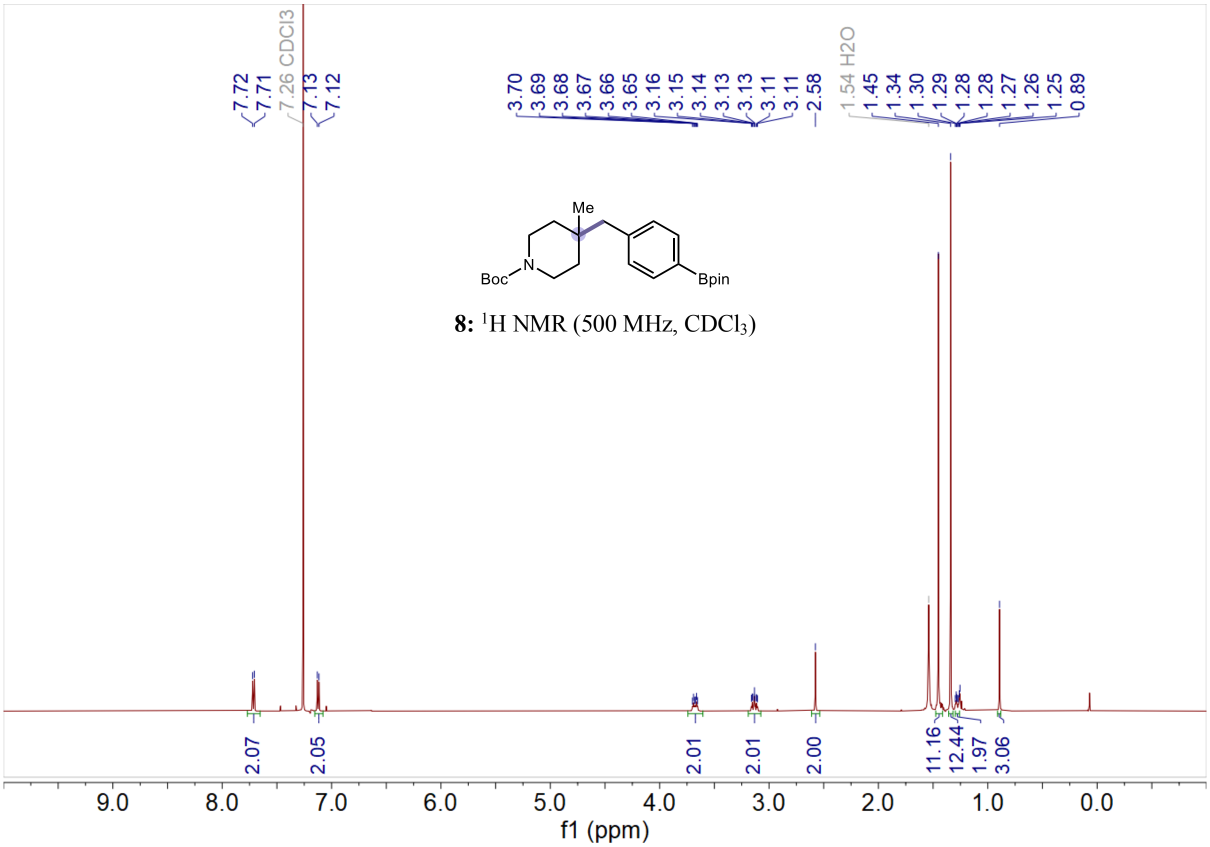
7.72
7.71
7.26 CDCl3
7.13
7.12
3.70
3.69
3.68
3.67
3.66
3.65
3.16
3.15
3.14
3.13
3.13
3.11
3.11
2.58
1.54 H2O
1.45
1.34
1.30
1.29
1.28
1.28
1.27
1.26
1.25
0.89
Me
Boc
N
Bpin
8: 1H NMR (500 MHz, CDCl3)
2.07
2.05
2.01
2.01
2.00
11.16
12.44
1.97
3.06
9.0
8.0
7.0
6.0
5.0
4.0
3.0
2.0
1.0
0.0
f1 (ppm)

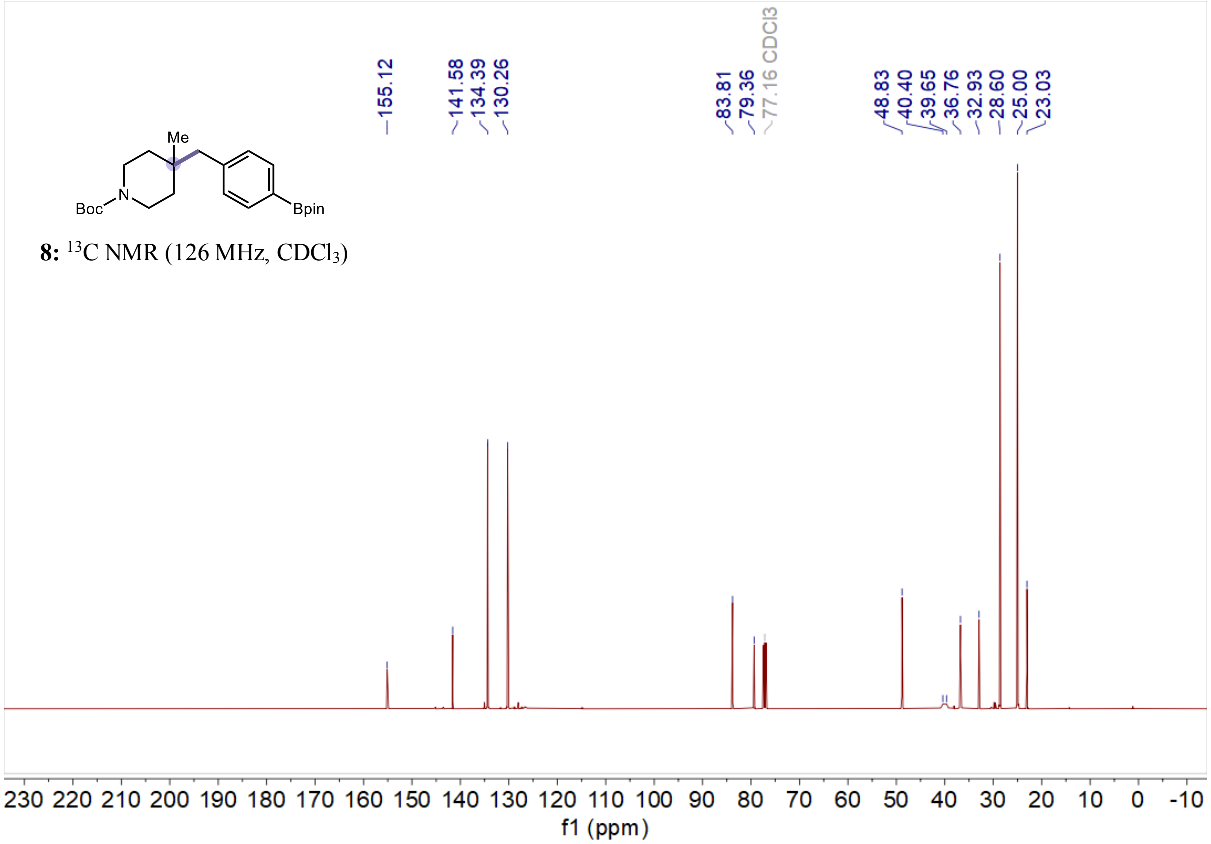
155.12
141.58
134.39
130.26
83.81
79.36
77.16 CDCl3
48.83
40.40
39.65
36.76
32.93
28.60
25.00
23.03
Me
Boc
N
Bpin
8: 13C NMR (126 MHz, CDCl3)
230 220 210 200 190 180 170 160 150 140 130 120 110 100 90 80 70 60 50 40 30 20 10 0 -10
f1 (ppm)

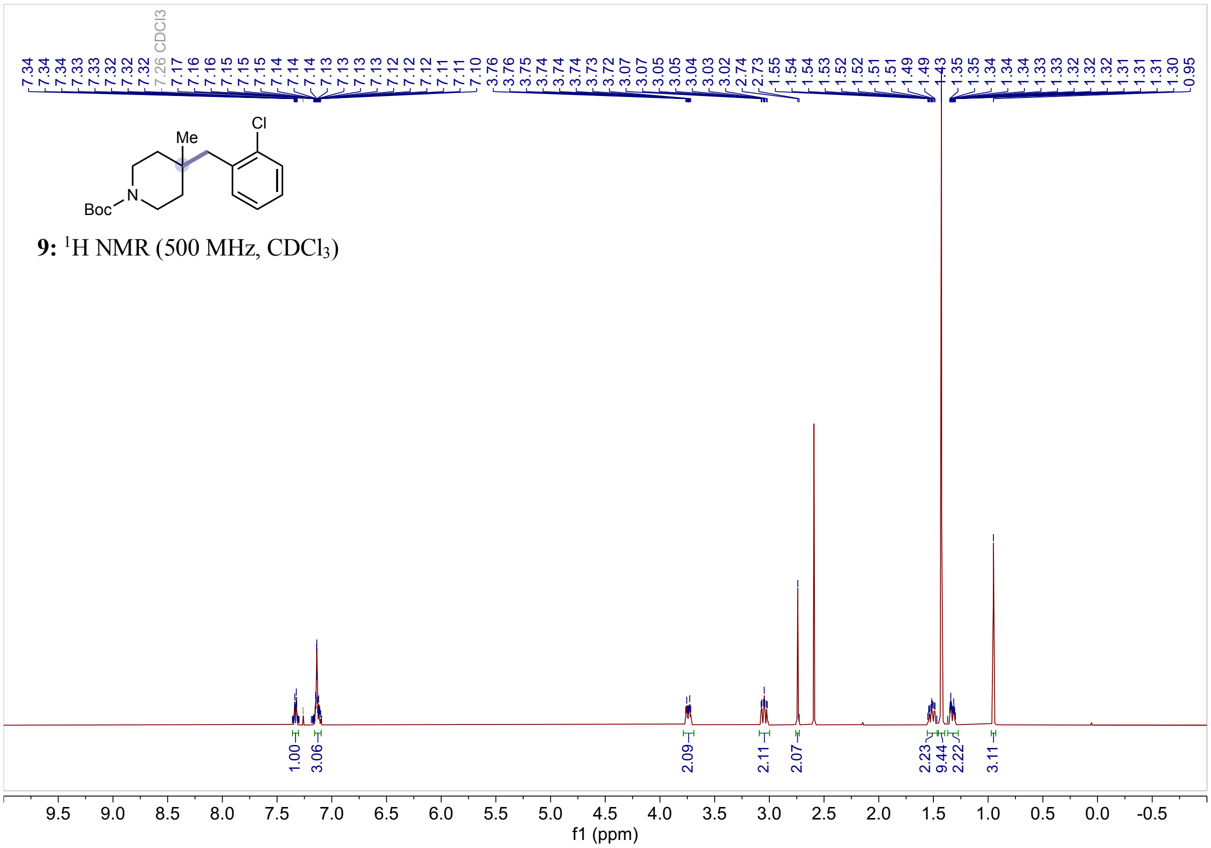

9: ^{1}H NMR (500 MHz, $CDCl_3$)

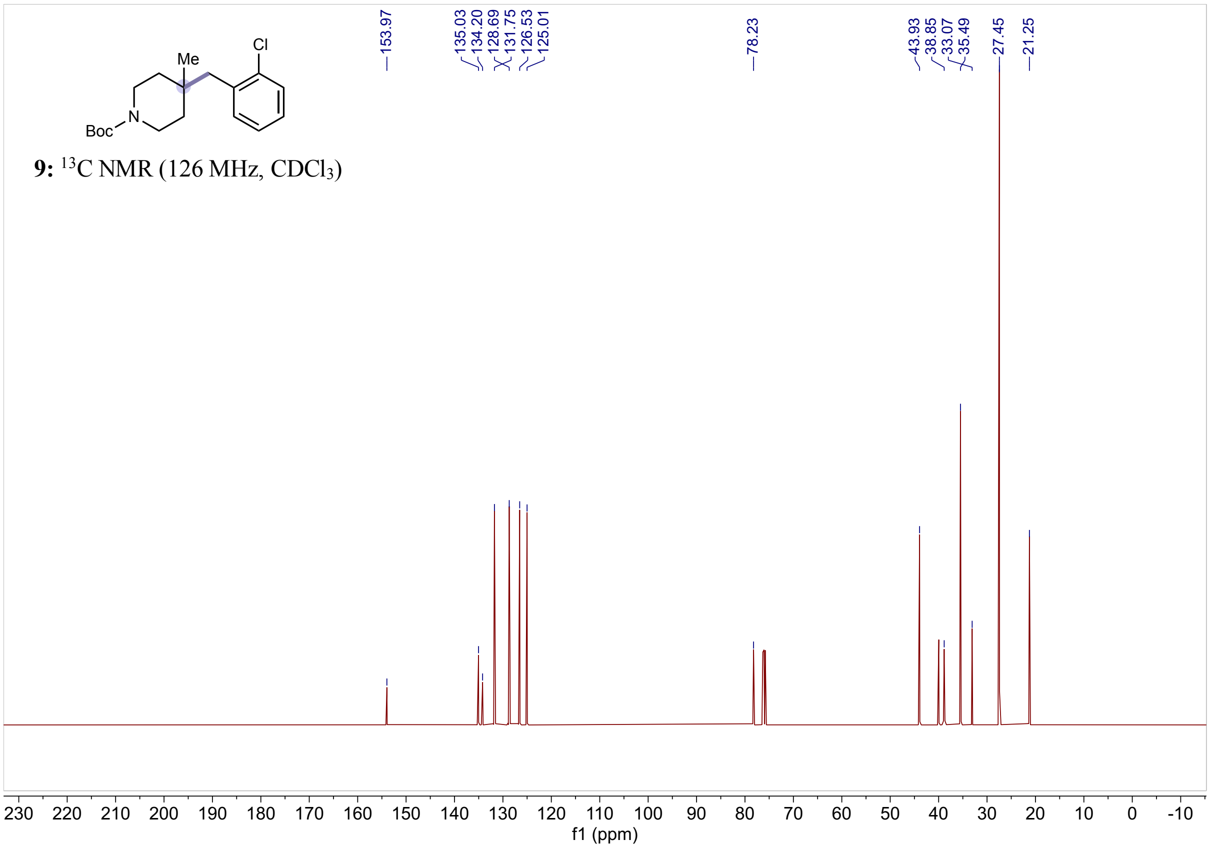

9: ^{13}C NMR (126 MHz, $CDCl_3$)

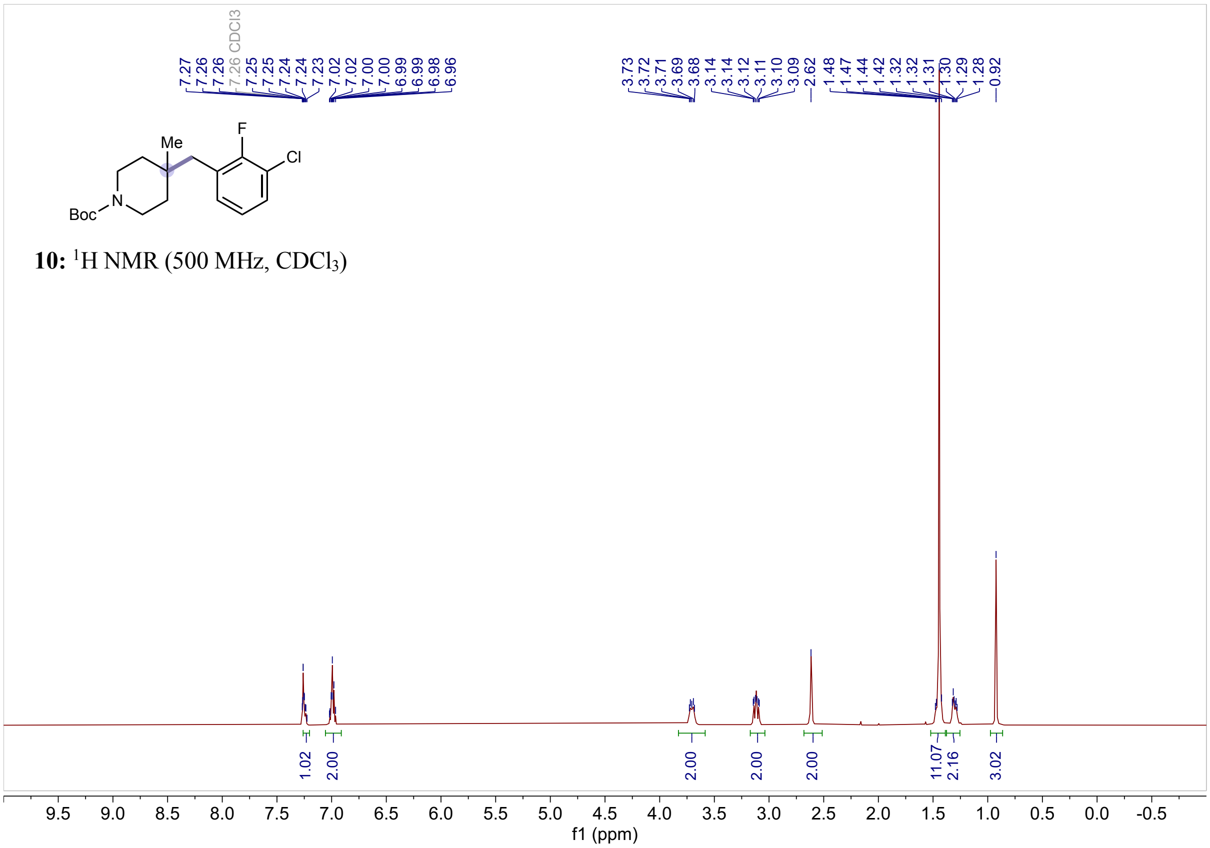

10: ¹H NMR (500 MHz, CDCl₃)
7.27
7.26
7.26 CDCl3
7.25
7.25
7.24
7.24
7.23
7.02
7.02
7.00
7.00
6.99
6.99
6.98
6.96
3.73
3.72
3.71
3.69
3.68
3.14
3.14
3.12
3.11
3.10
3.09
2.62
1.48
1.47
1.44
1.42
1.32
1.32
1.31
1.30
1.29
1.28
0.92
1.02
2.00
2.00
2.00
2.00
11.07
2.16
3.02
f1 (ppm)

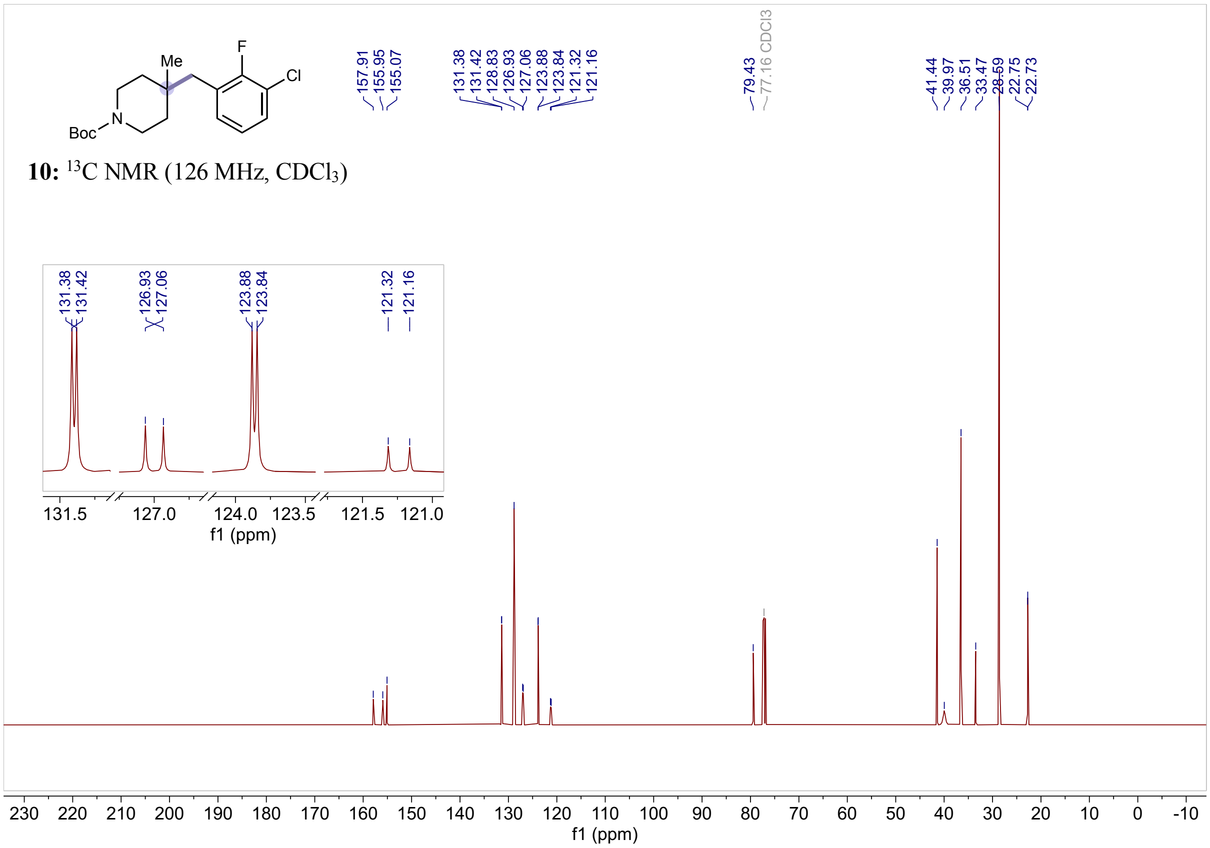

10: ¹³C NMR (126 MHz, CDCl₃)
157.91
155.95
155.07
131.38
131.42
128.83
126.93
127.06
123.88
123.84
121.32
121.16
79.43
77.16 CDCl3
41.44
39.97
36.51
33.47
28.59
22.75
22.73
f1 (ppm)

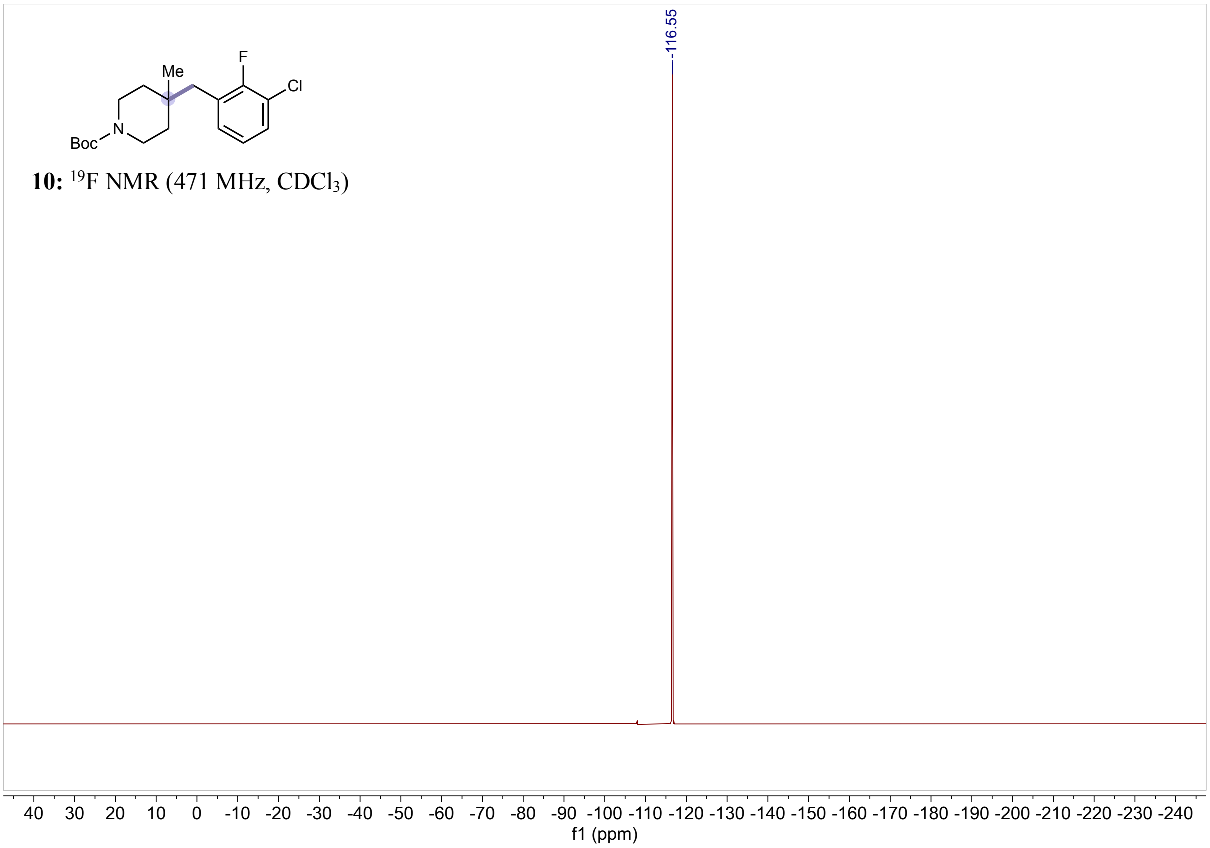

10: ^{19}F NMR (471 MHz, $CDCl_3$)

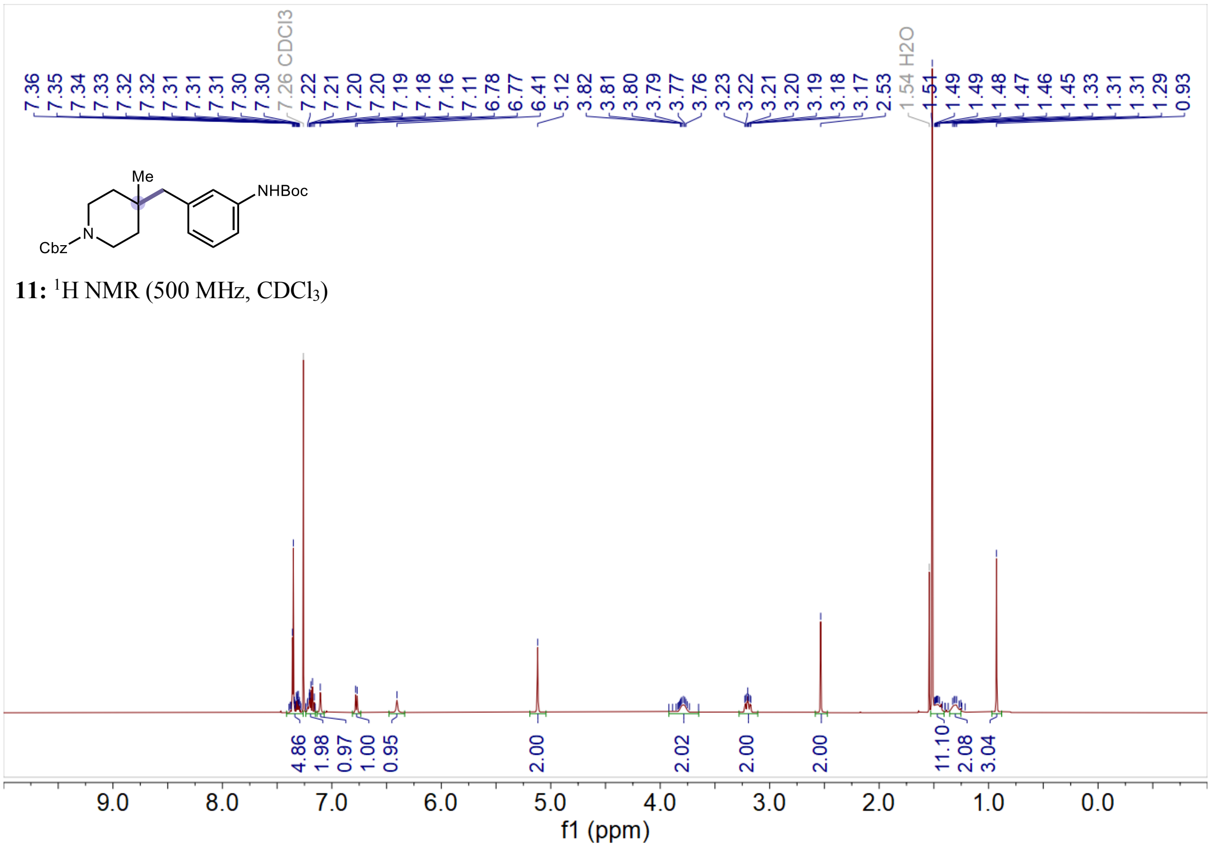

11: ^{1}H NMR (500 MHz, $CDCl_3$)

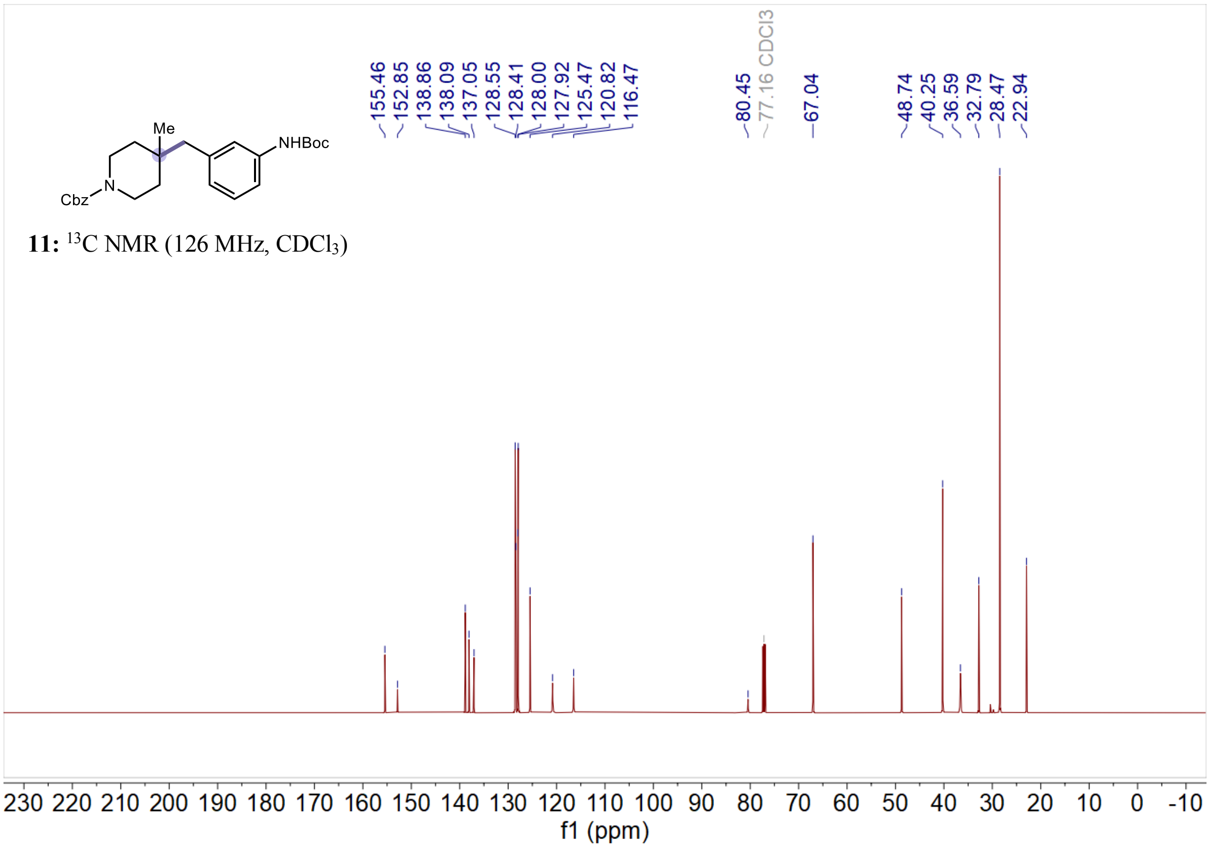

11: ^{13}C NMR (126 MHz, $CDCl_3$)

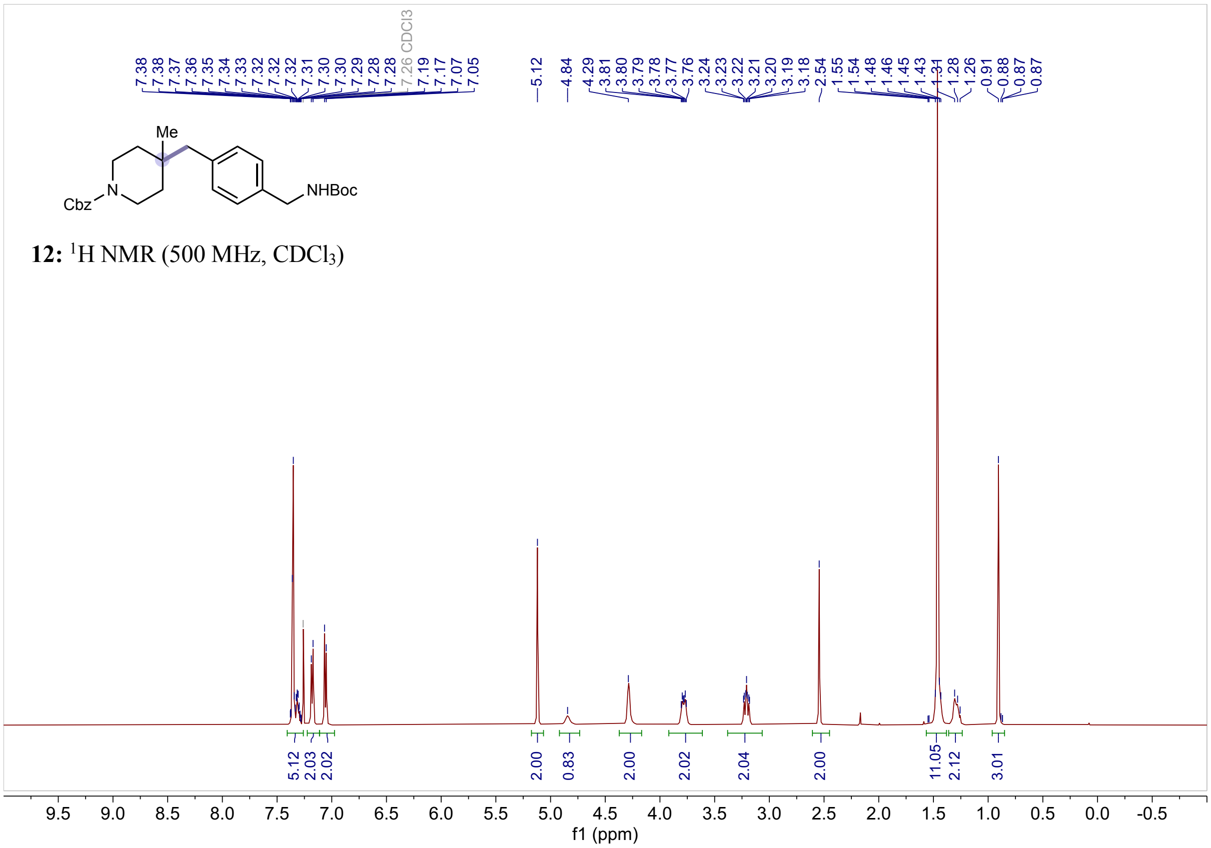

12: ^{1}H NMR (500 MHz, $CDCl_3$)

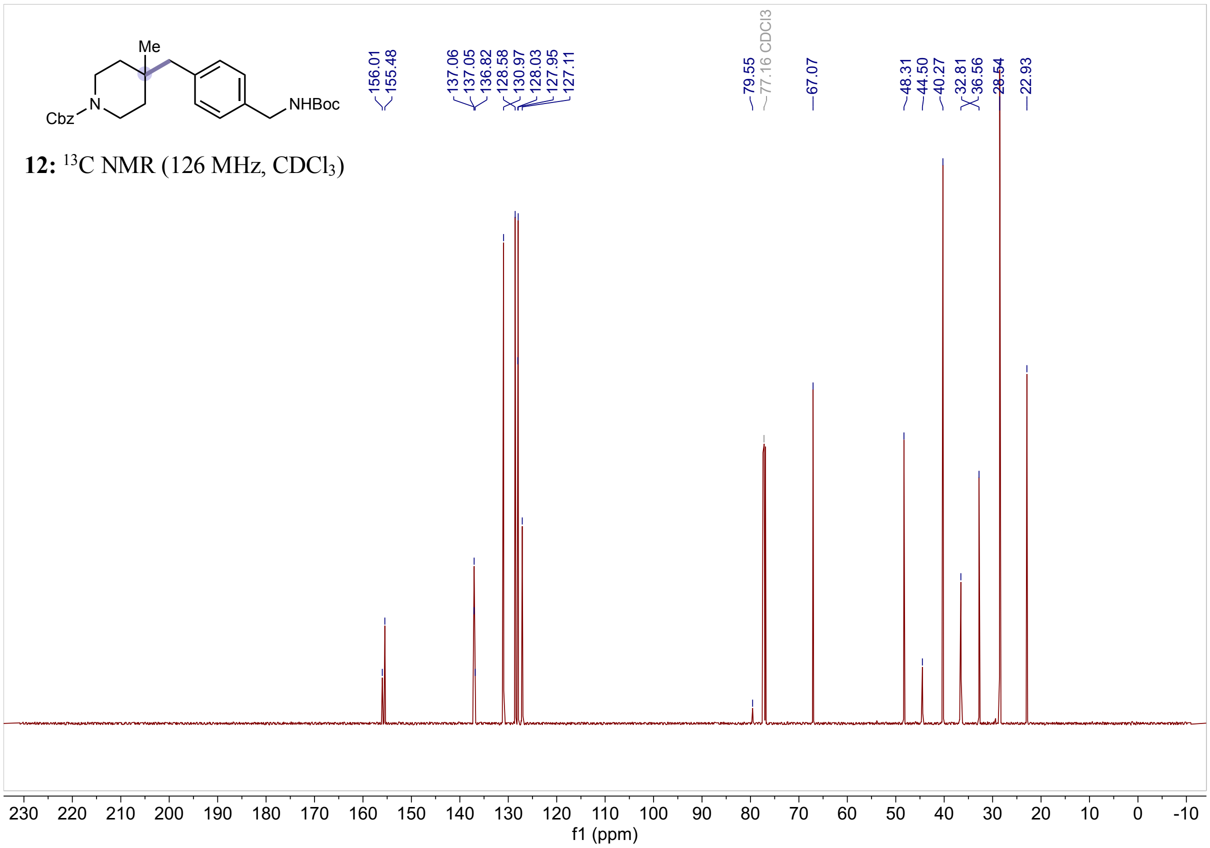

12: ^{13}C NMR (126 MHz, $CDCl_3$)

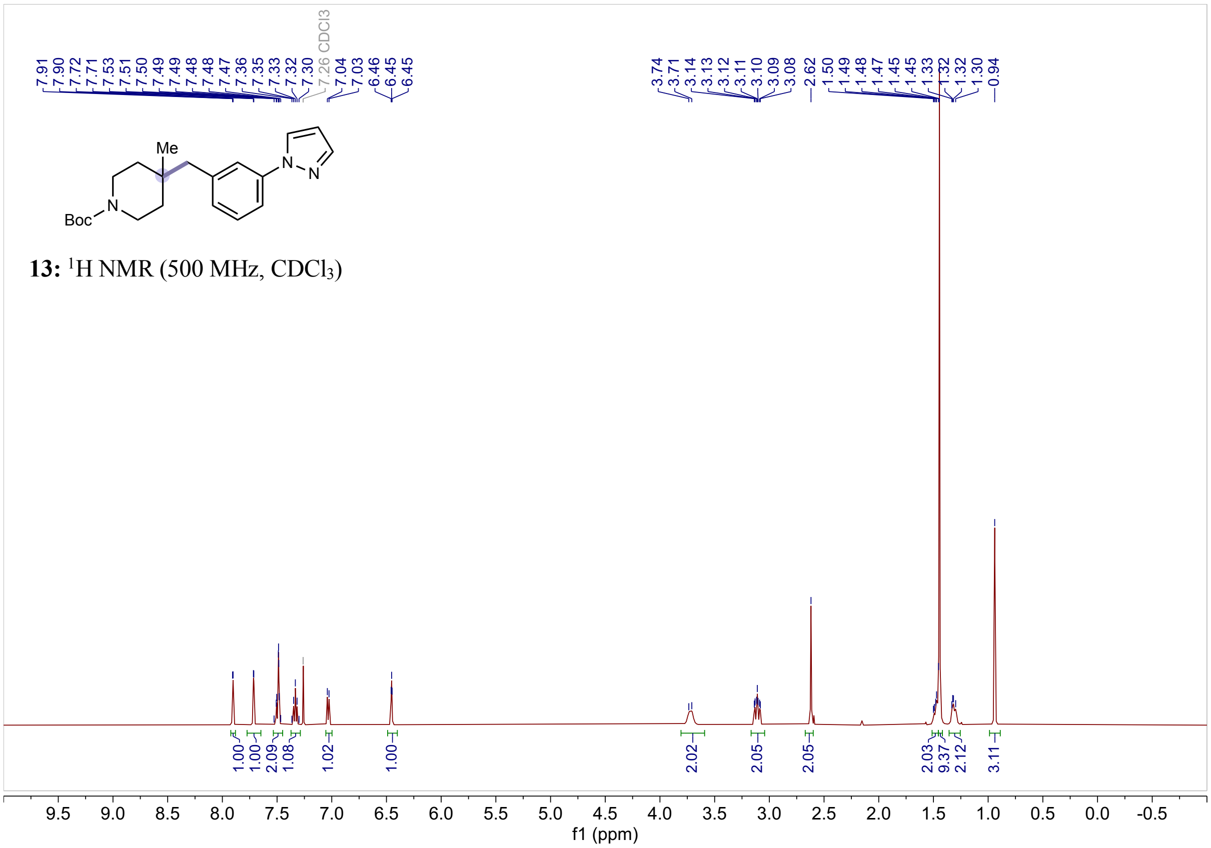

13: ^{1}H NMR (500 MHz, $CDCl_3$)

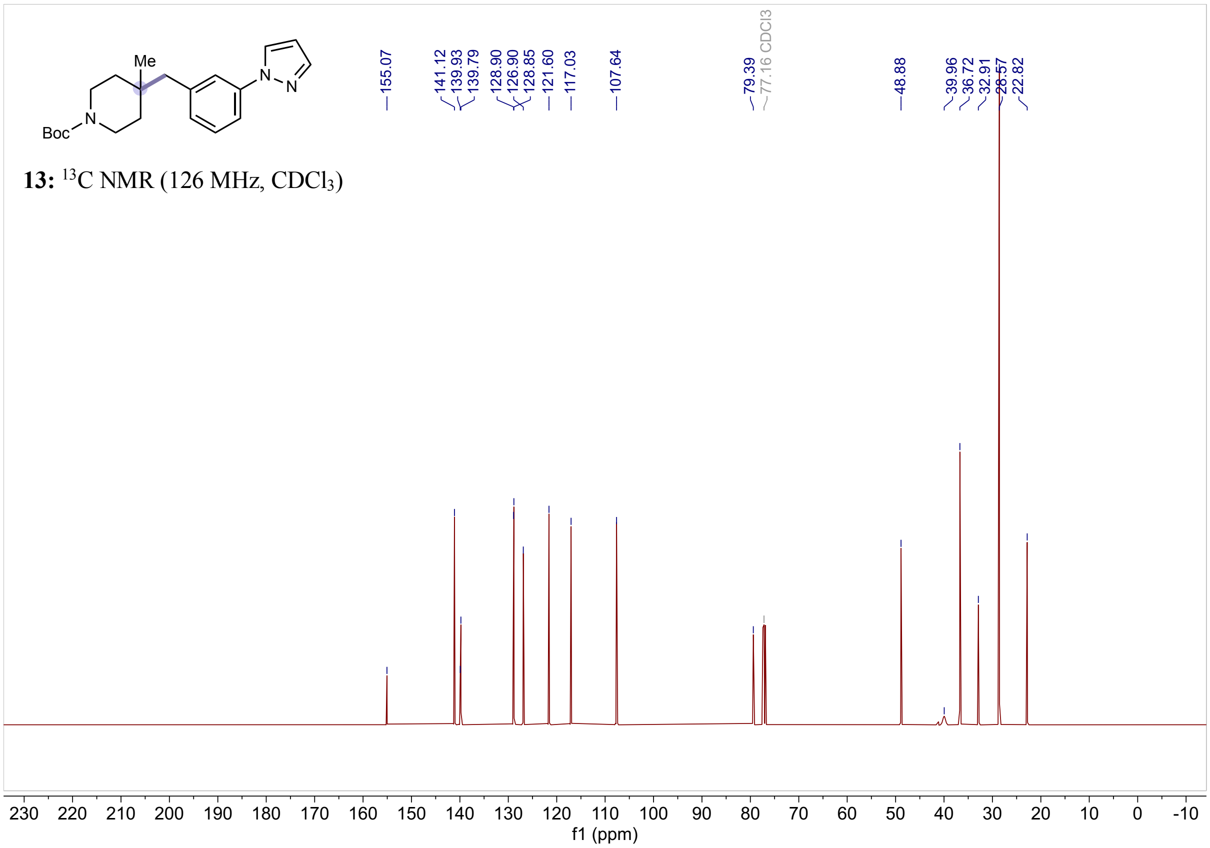

13: ^{13}C NMR (126 MHz, $CDCl_3$)

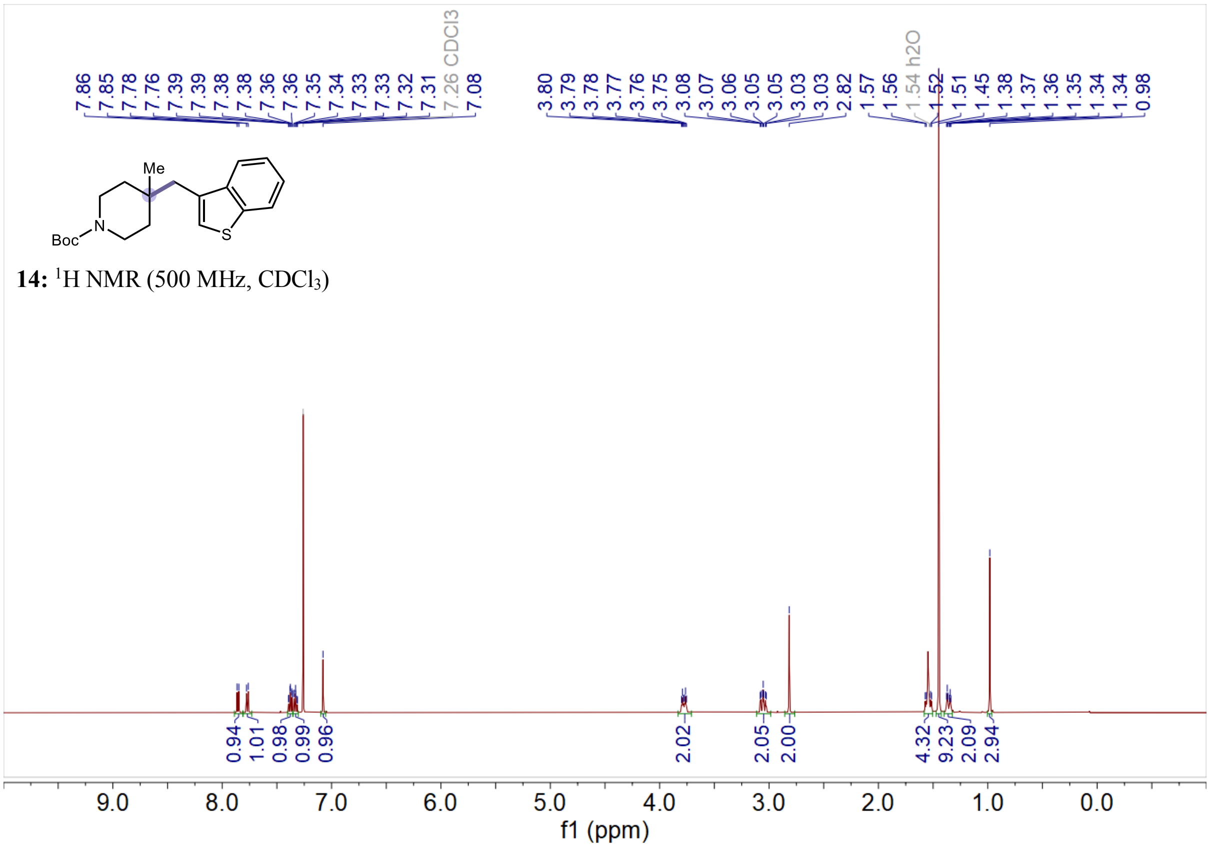

14: ^{1}H NMR (500 MHz, $CDCl_3$)

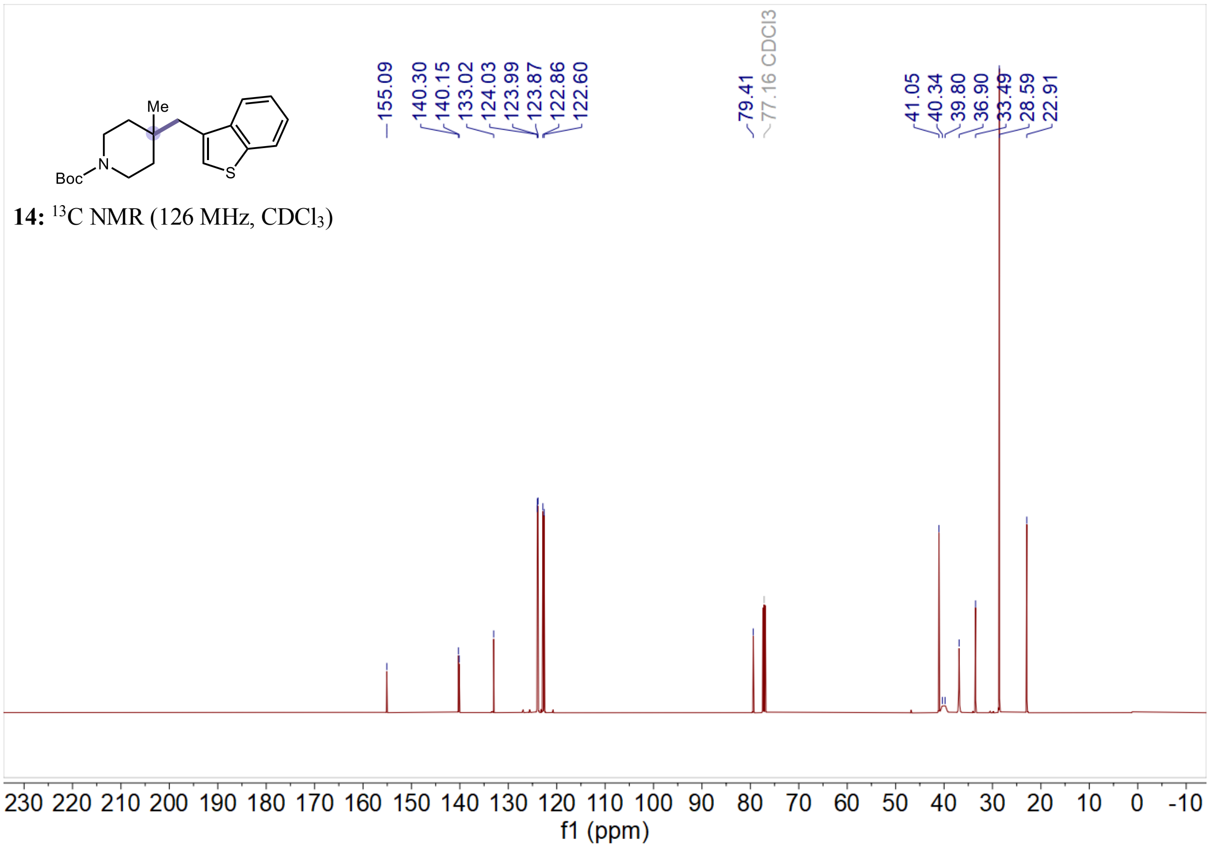

14: ^{13}C NMR (126 MHz, $CDCl_3$)

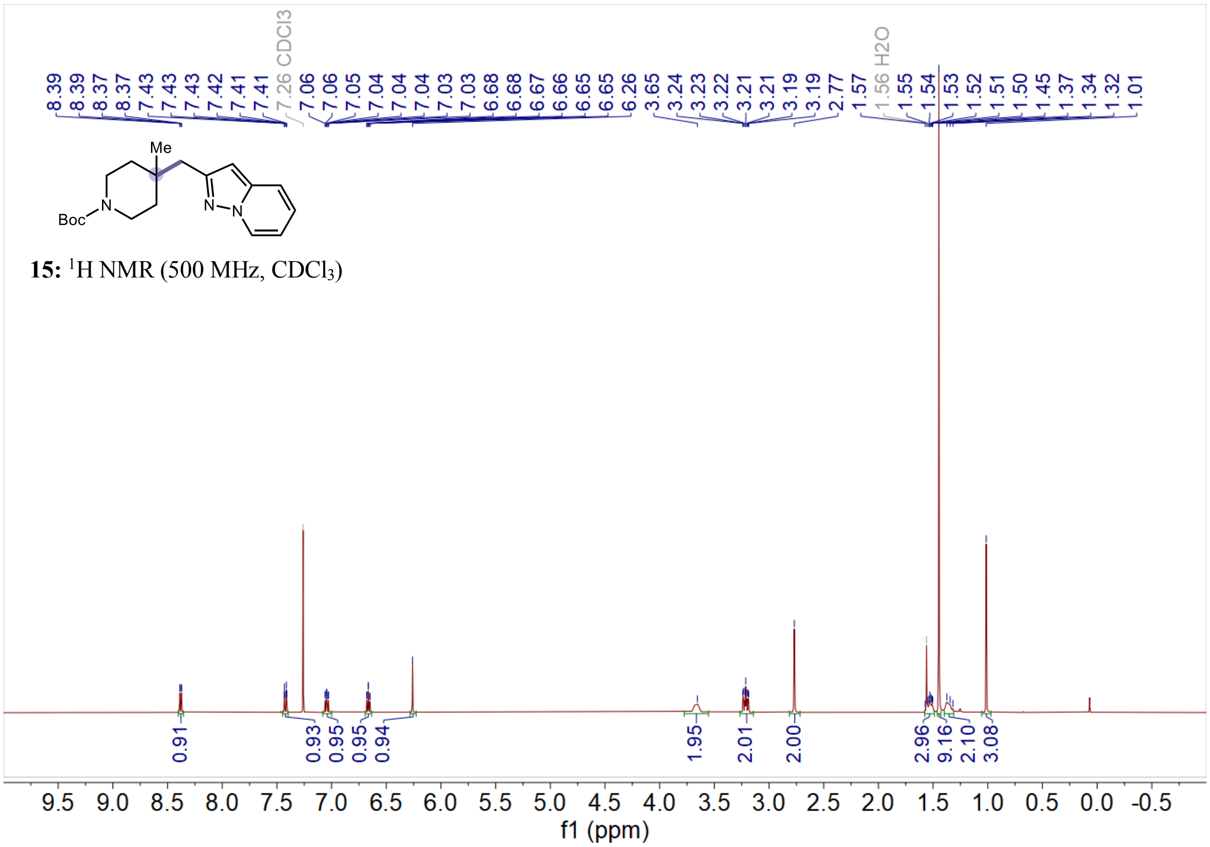

15: ^{1}H NMR (500 MHz, $CDCl_3$)

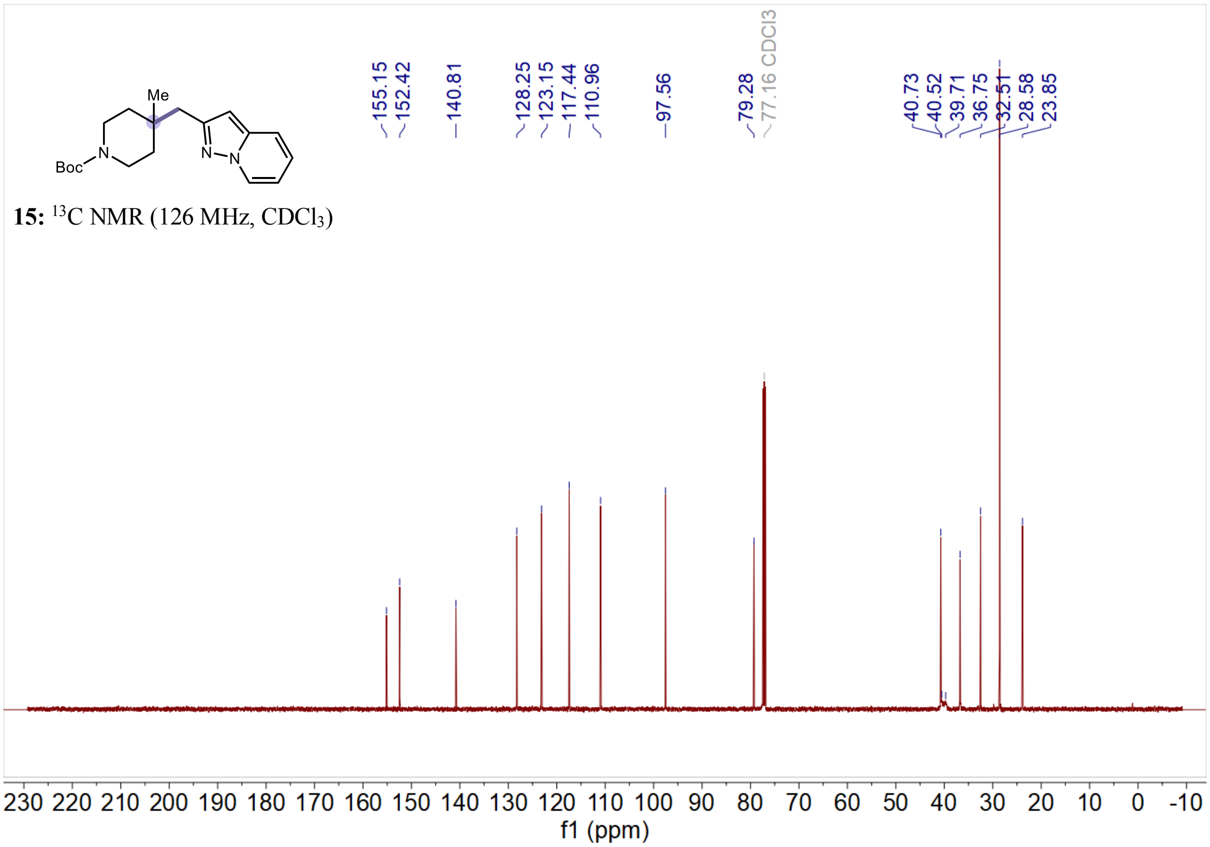

15: ^{13}C NMR (126 MHz, $CDCl_3$)

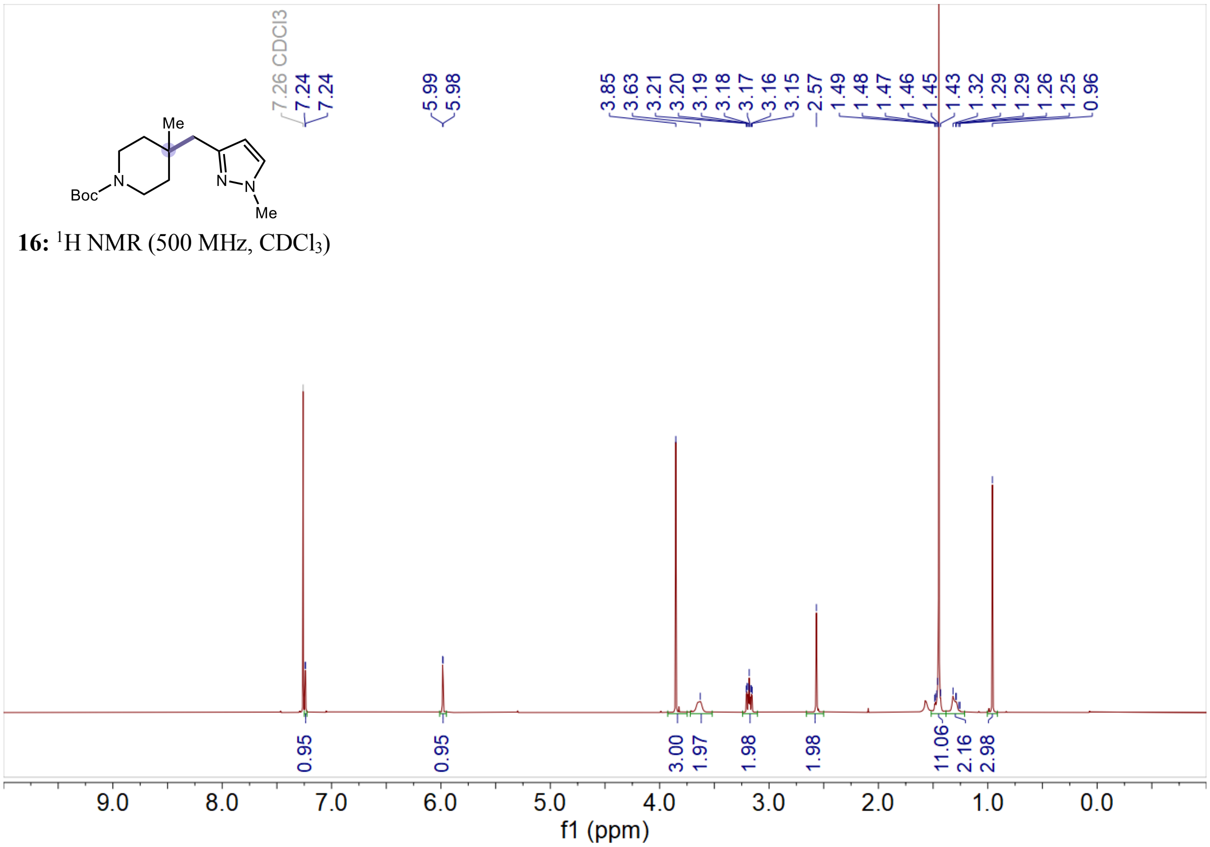

16: ^{1}H NMR (500 MHz, $CDCl_3$)

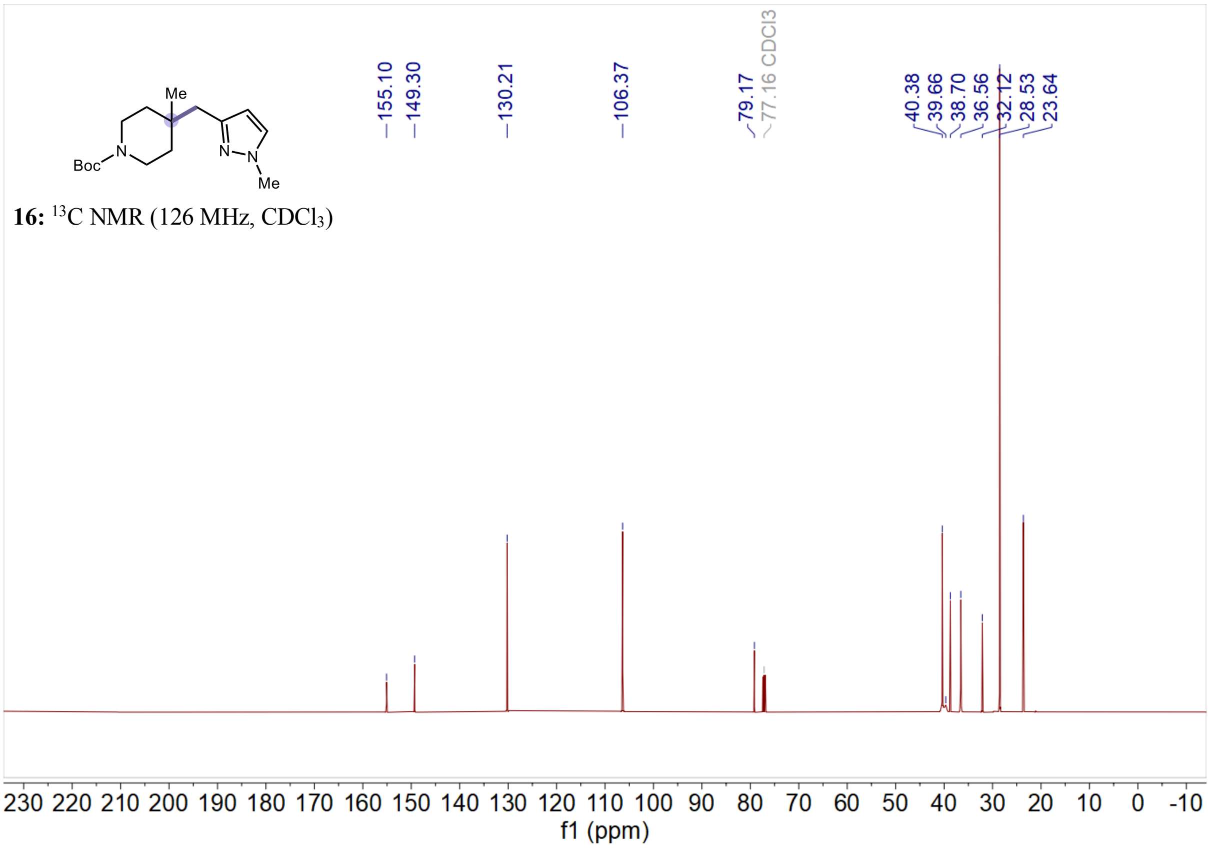

16: ^{13}C NMR (126 MHz, $CDCl_3$)

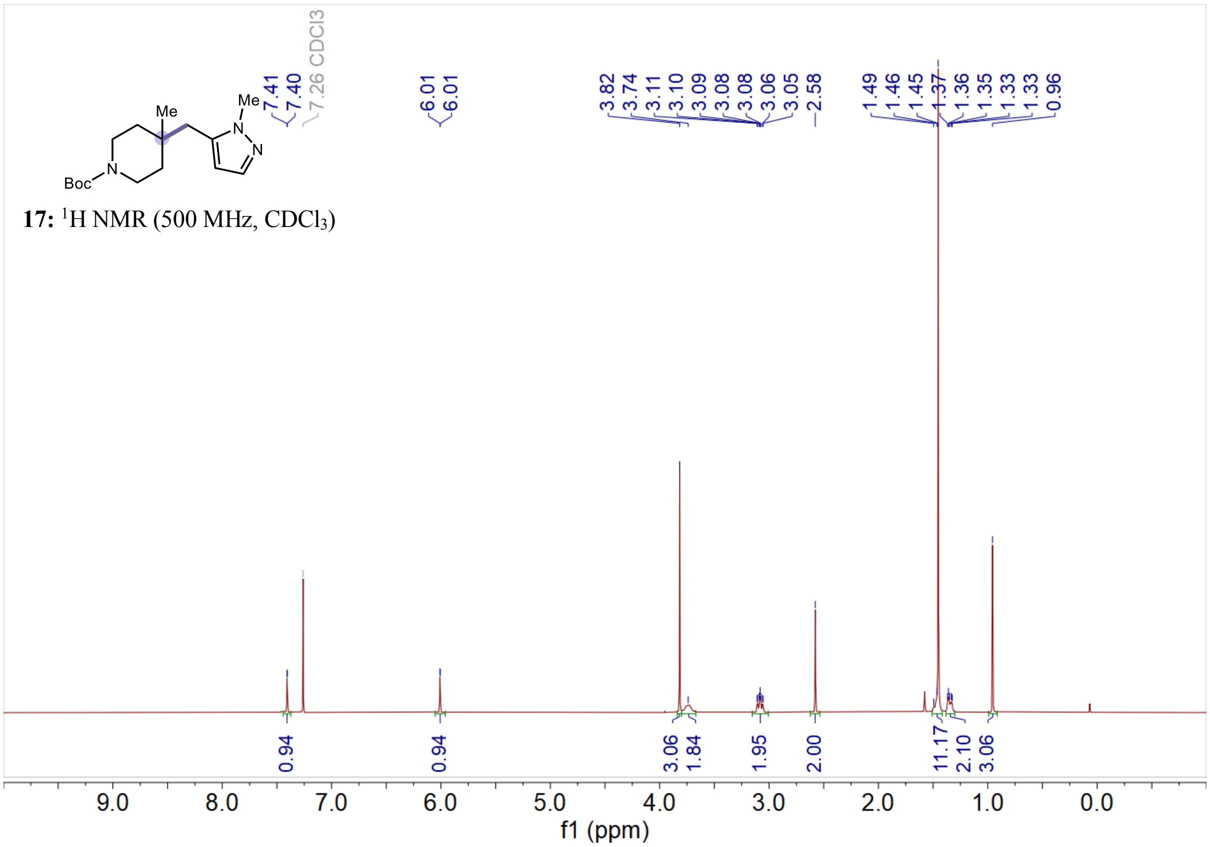

17: ^{1}H NMR (500 MHz, $CDCl_3$)

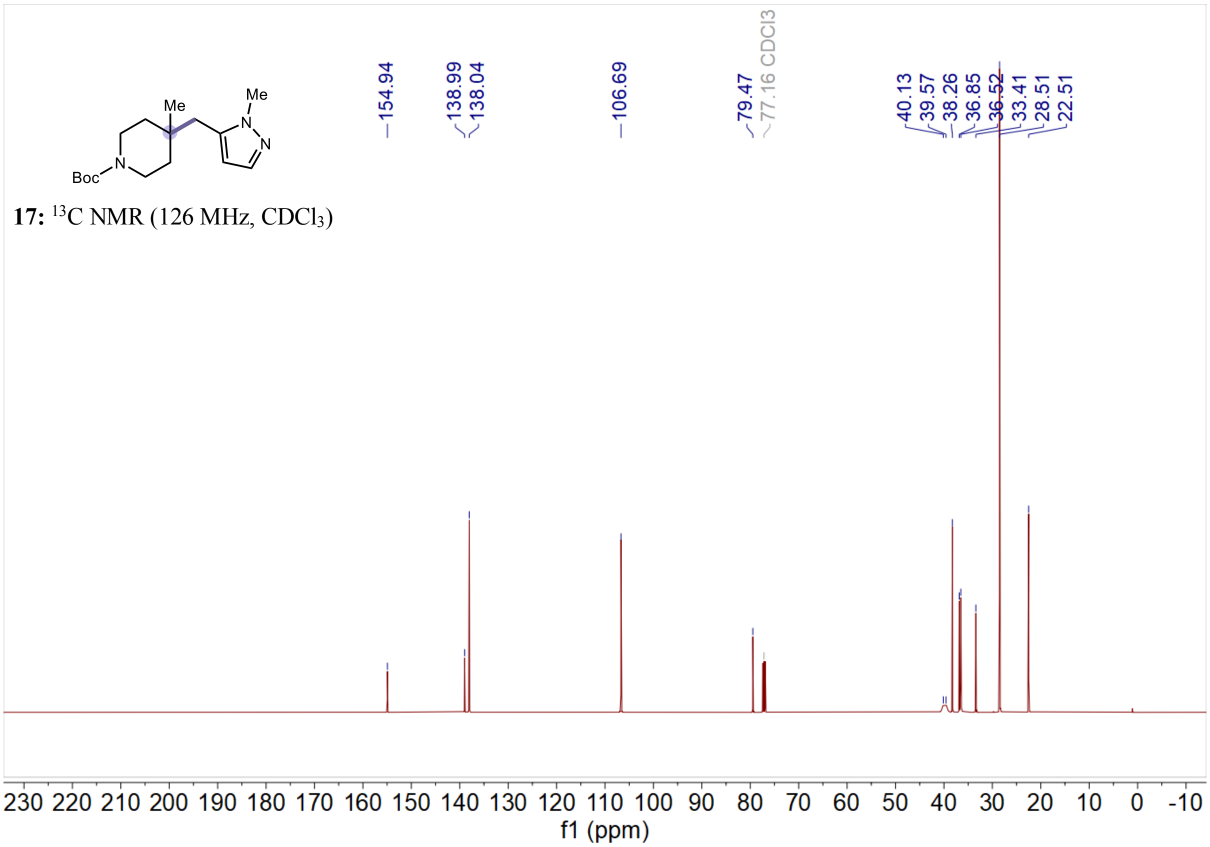

17: ^{13}C NMR (126 MHz, $CDCl_3$)

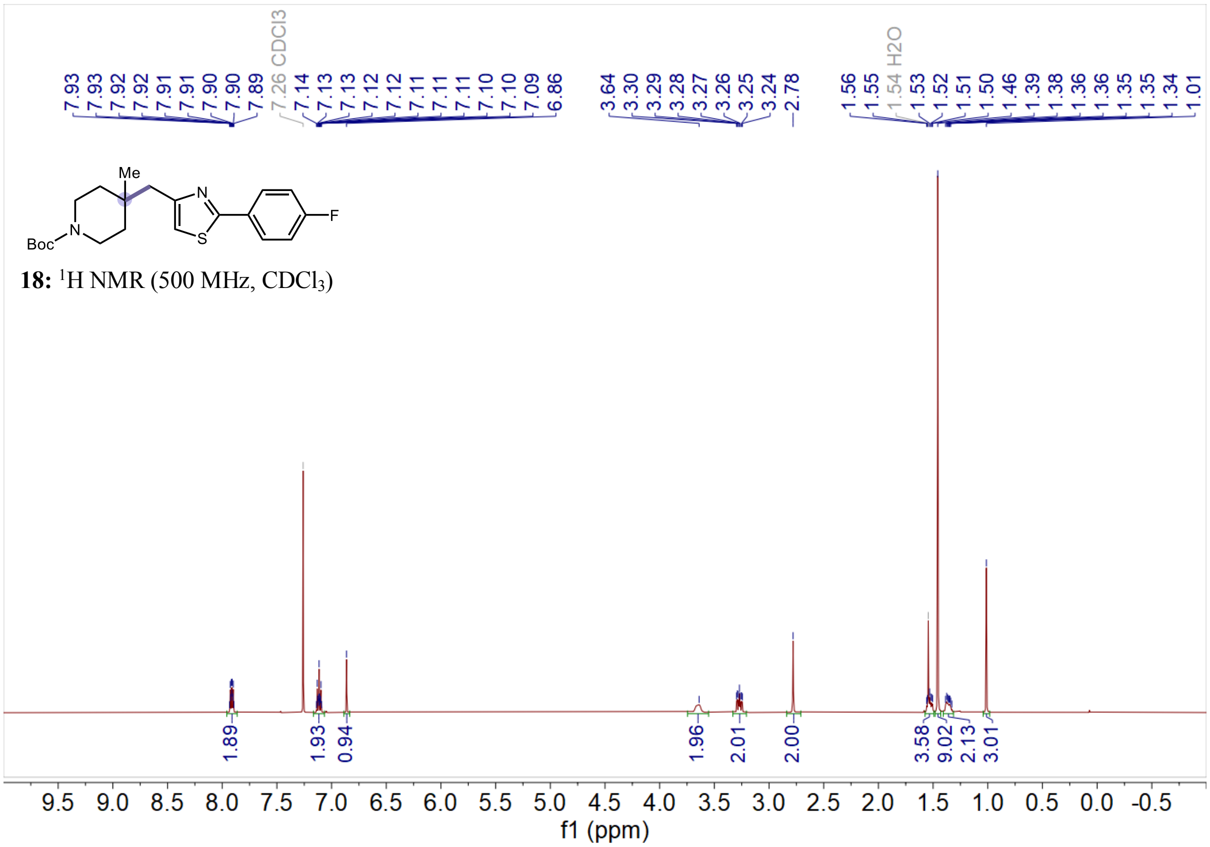
7.93 7.93 7.92 7.92 7.91 7.91 7.90 7.90 7.89
7.26 CDCl3
7.14 7.13 7.13 7.12 7.12 7.11 7.11 7.11 7.10 7.10 7.09 6.86
3.64 3.30 3.29 3.28 3.27 3.26 3.25 3.24 2.78
1.56 1.55
1.54 H2O
1.53 1.52 1.51 1.50 1.46 1.39 1.38 1.36 1.36 1.35 1.35 1.34 1.01
Me
N
S
F
Boc
N
18: ¹H NMR (500 MHz, CDCl3)
1.89 1.93 0.94 1.96 2.01 2.00 3.58 9.02 2.13 3.01
9.5 9.0 8.5 8.0 7.5 7.0 6.5 6.0 5.5 5.0 4.5 4.0 3.5 3.0 2.5 2.0 1.5 1.0 0.5 0.0 -0.5
f1 (ppm)

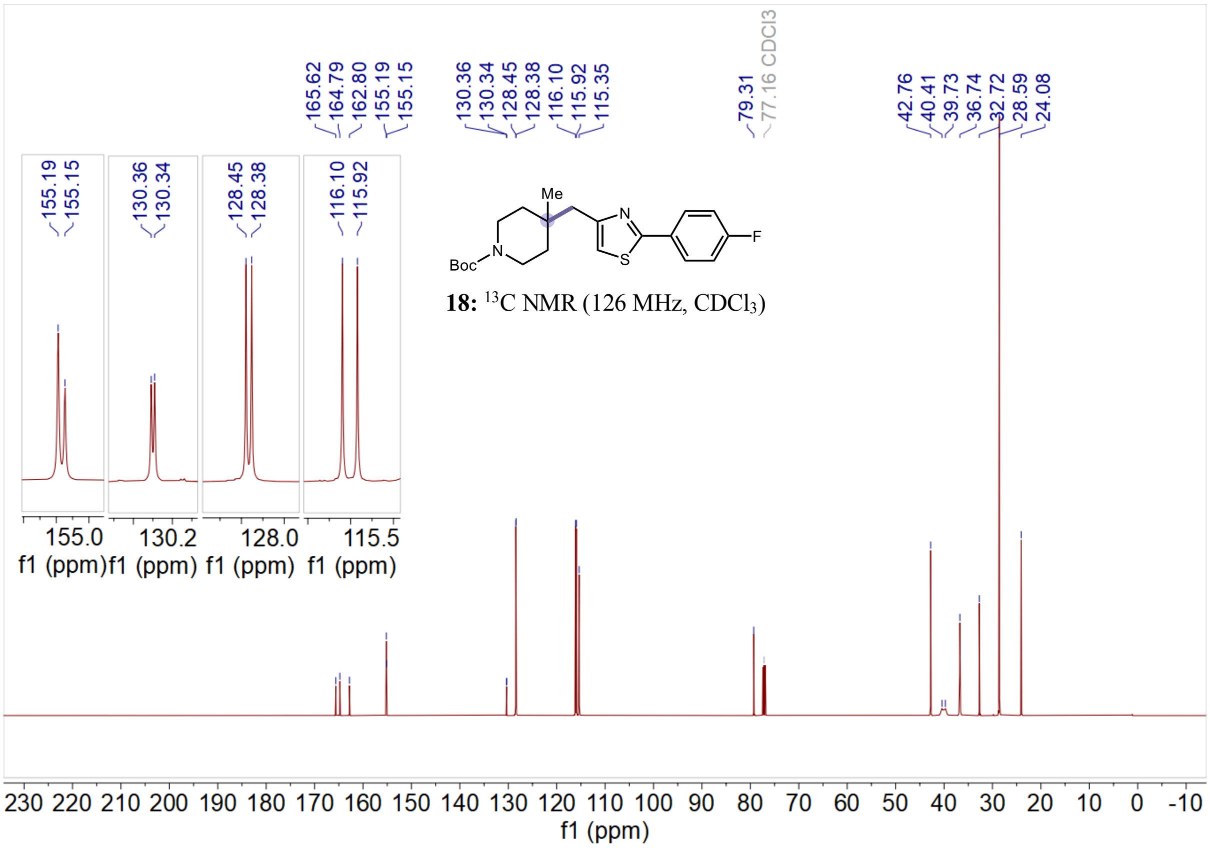
165.62 164.79 162.80 155.19 155.15
130.36 130.34 128.45 128.38 116.10 115.92 115.35
79.31
77.16 CDCl3
42.76 40.41 39.73 36.74 32.72 28.59 24.08
155.19 155.15
130.36 130.34
128.45 128.38
116.10 115.92
155.0 130.2 128.0 115.5
f1 (ppm) f1 (ppm) f1 (ppm) f1 (ppm)
Me
N
S
F
Boc
N
18: ¹³C NMR (126 MHz, CDCl3)
230 220 210 200 190 180 170 160 150 140 130 120 110 100 90 80 70 60 50 40 30 20 10 0 -10
f1 (ppm)

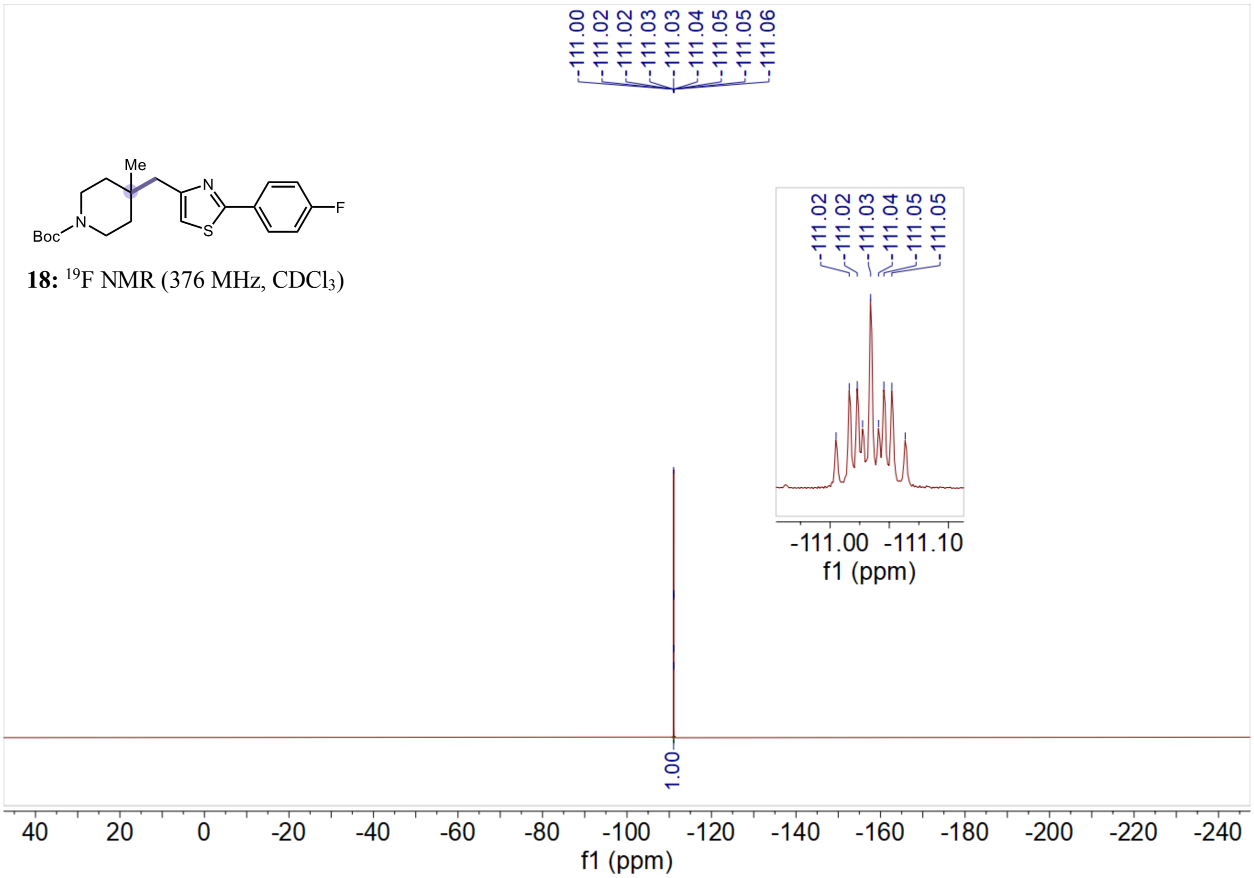

18: ^{19}F NMR (376 MHz, $CDCl_3$)

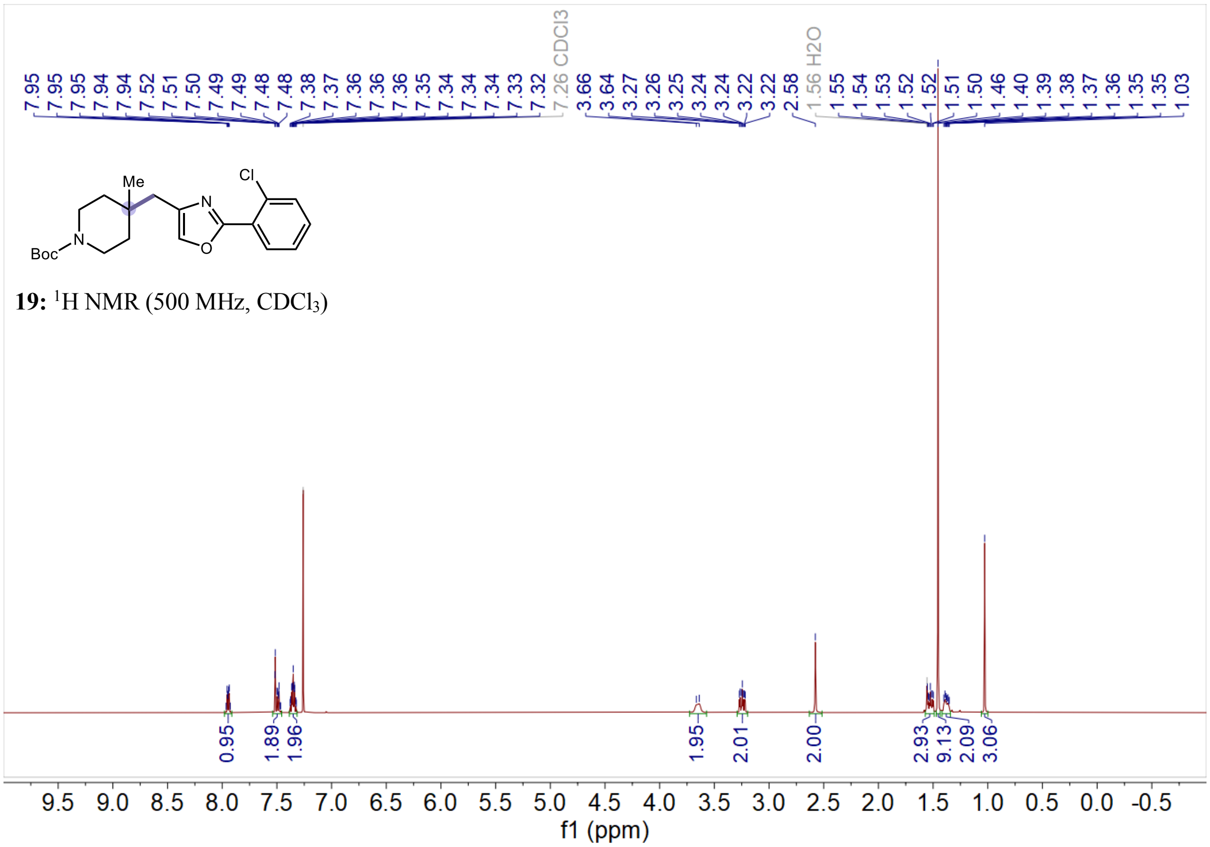

19: ^{1}H NMR (500 MHz, $CDCl_3$)

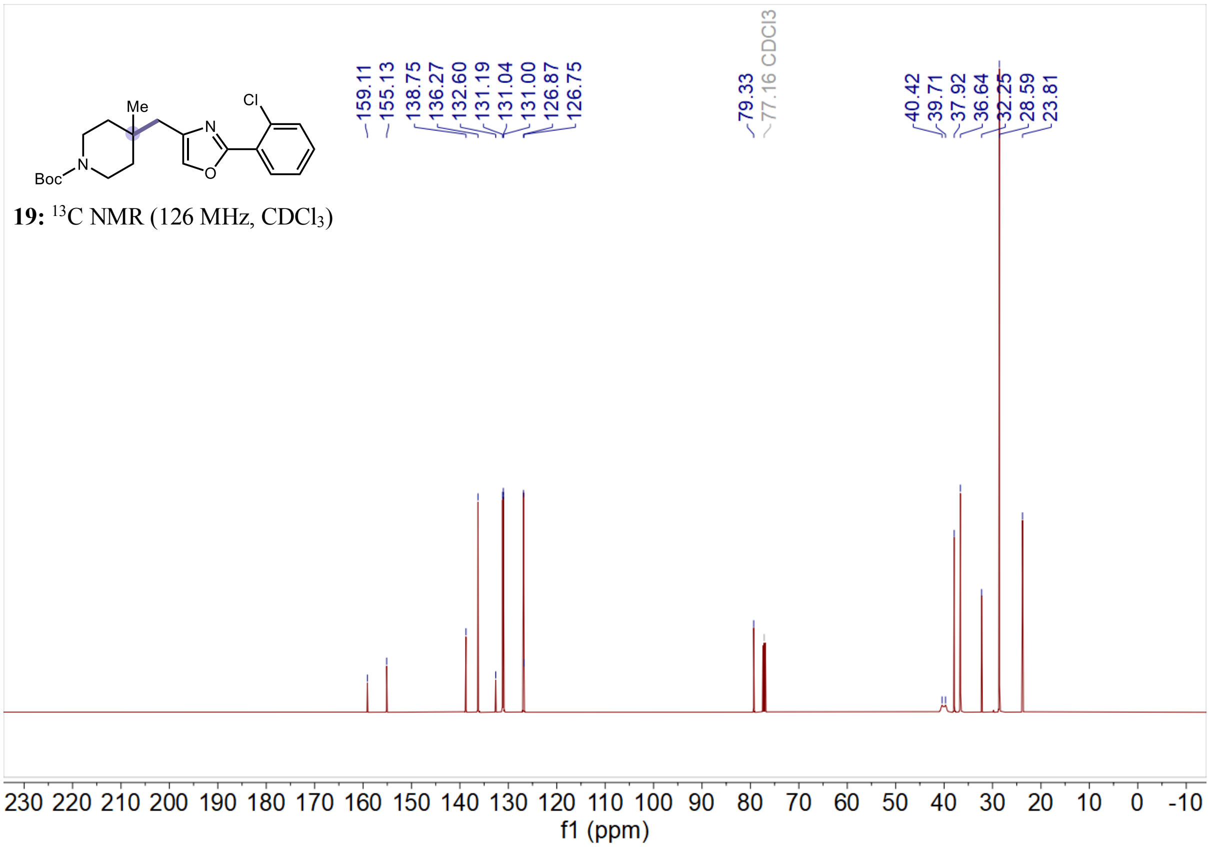

19: ^{13}C NMR (126 MHz, $CDCl_3$)

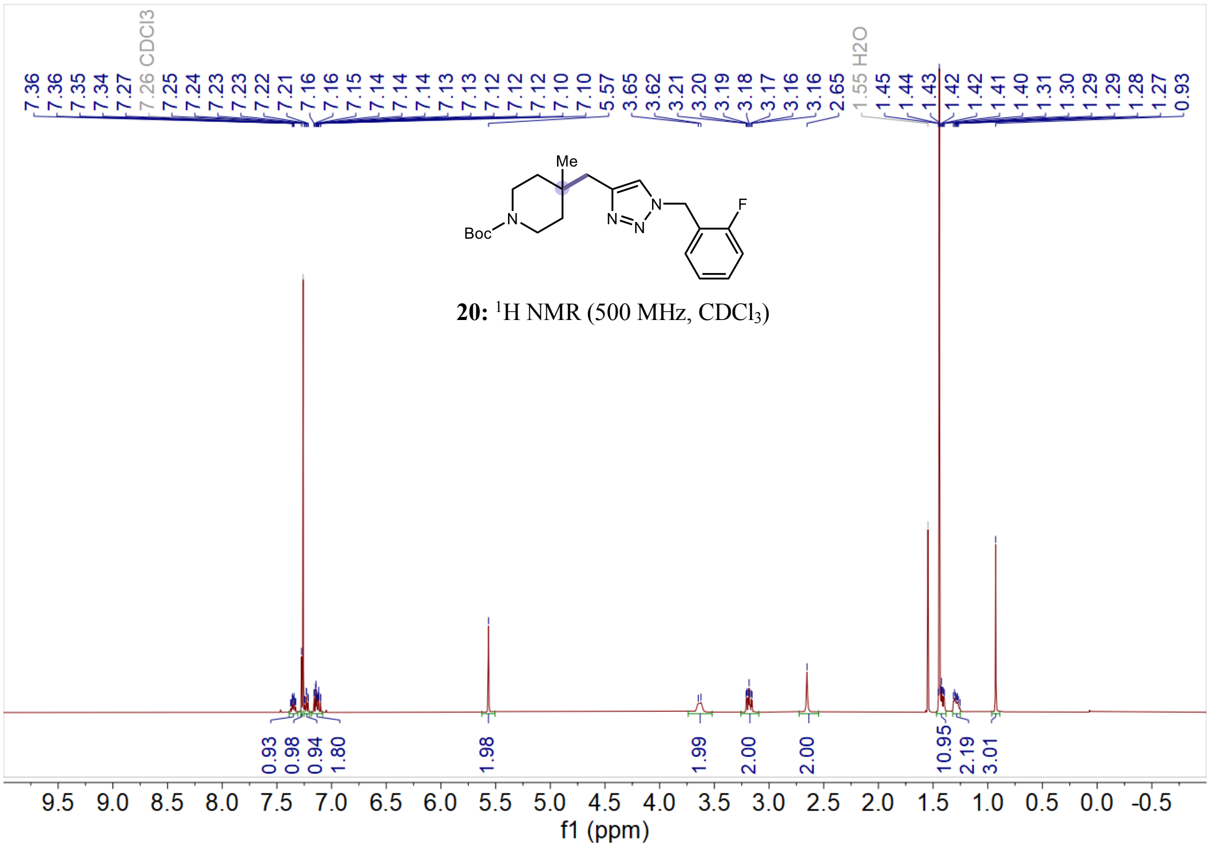

20: ^{1}H NMR (500 MHz, $CDCl_3$)

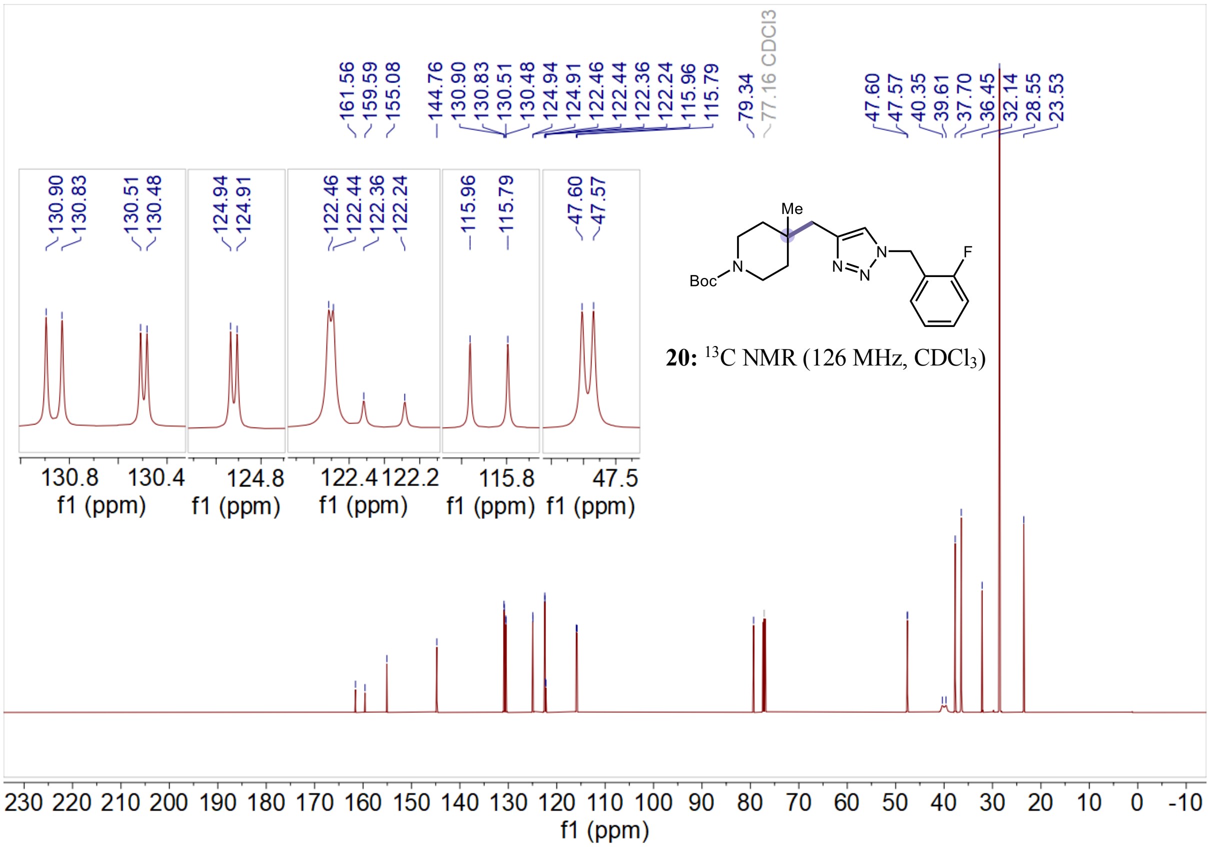

20: ^{13}C NMR (126 MHz, $CDCl_3$)

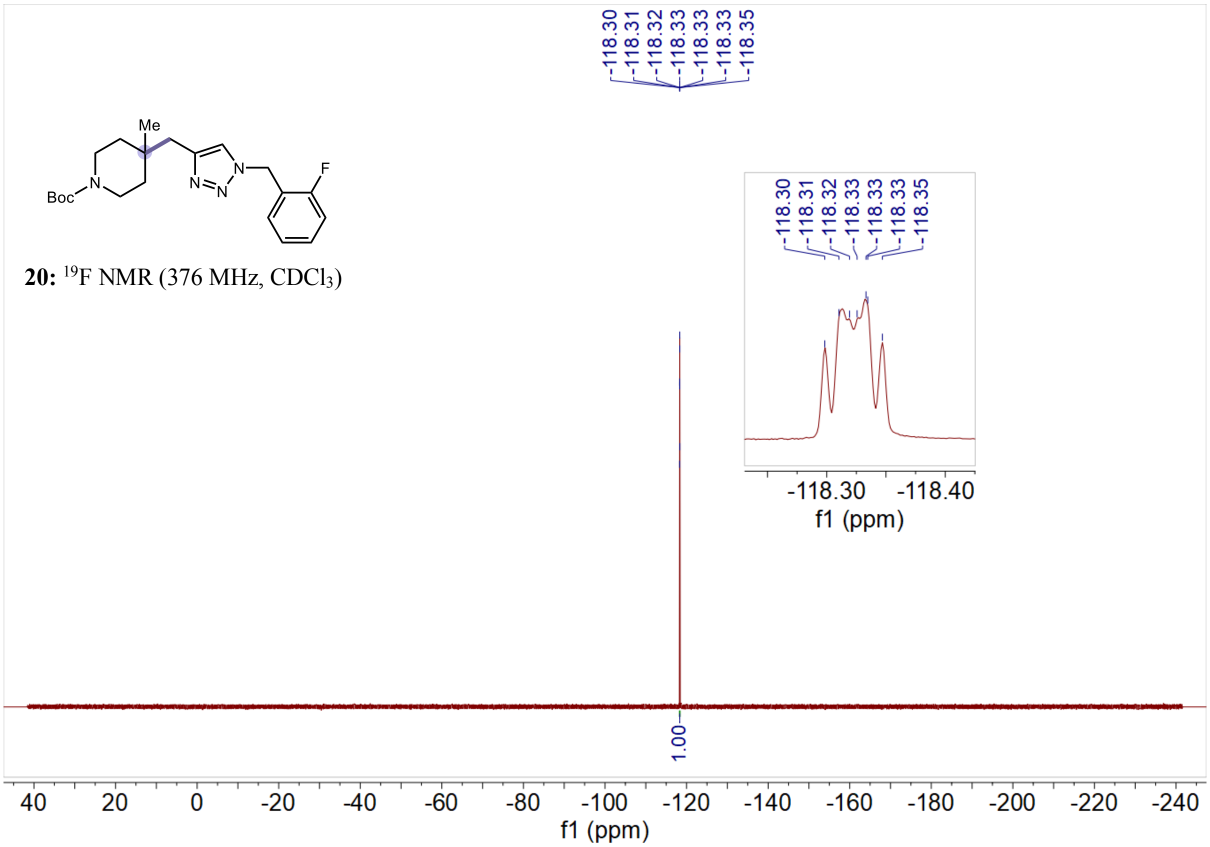

20: ^{19}F NMR (376 MHz, $CDCl_3$)

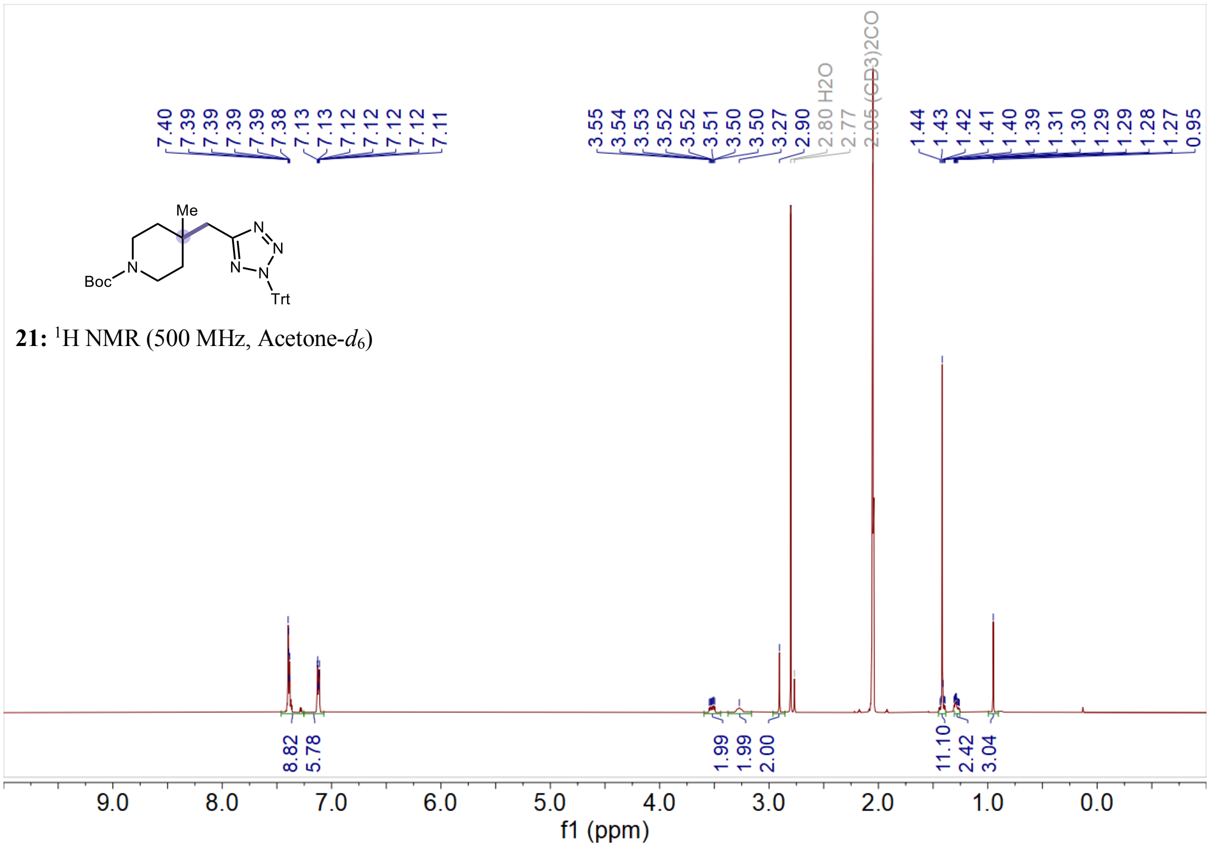

21: ^{1}H NMR (500 MHz, Acetone-d_6)

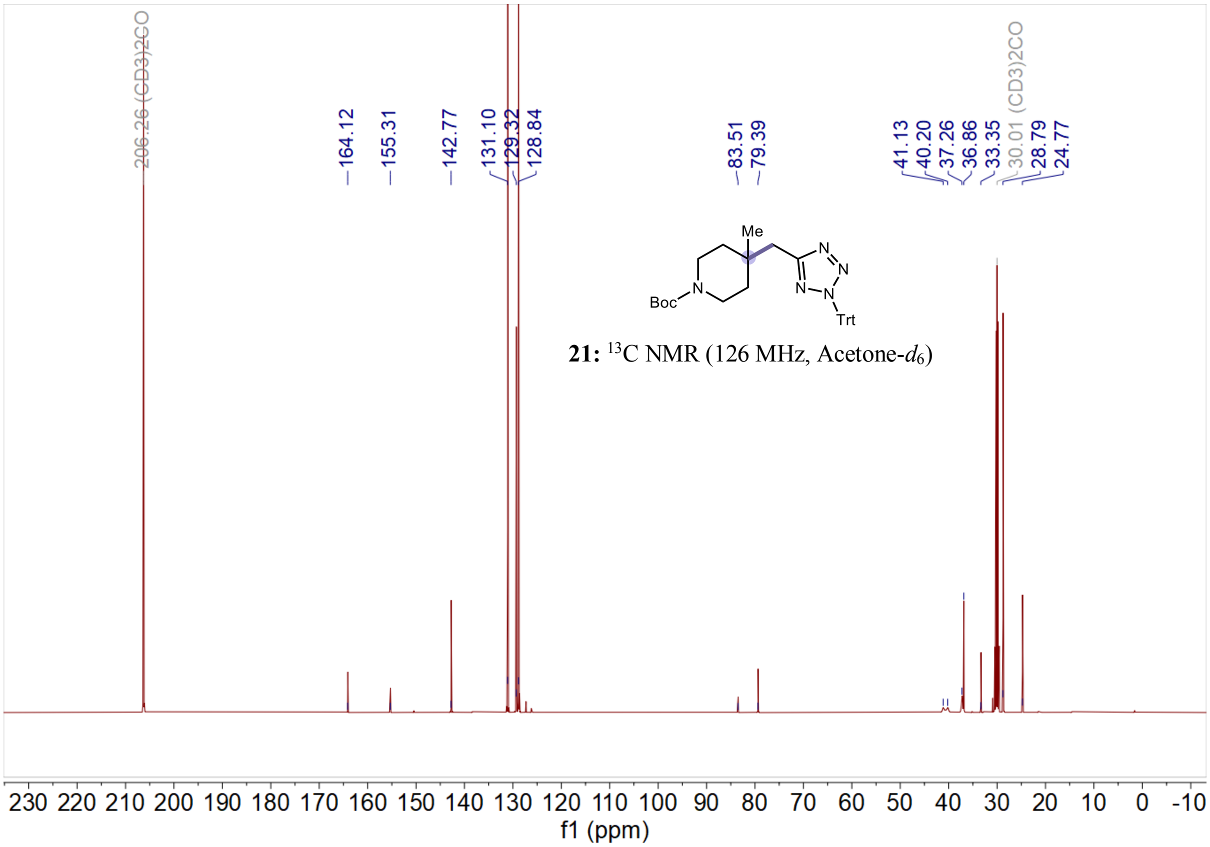

21: ^{13}C NMR (126 MHz, Acetone-d_6)

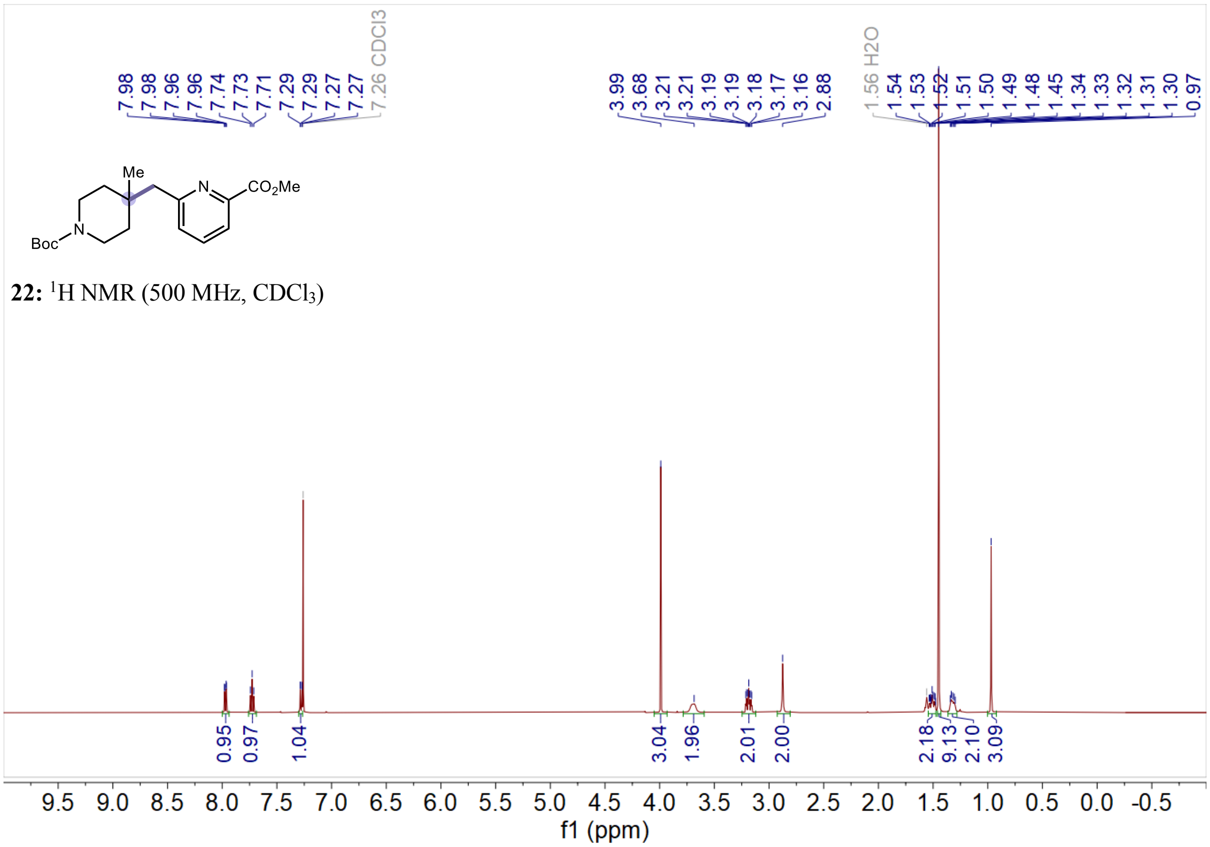

22: ^{1}H NMR (500 MHz, $CDCl_3$)

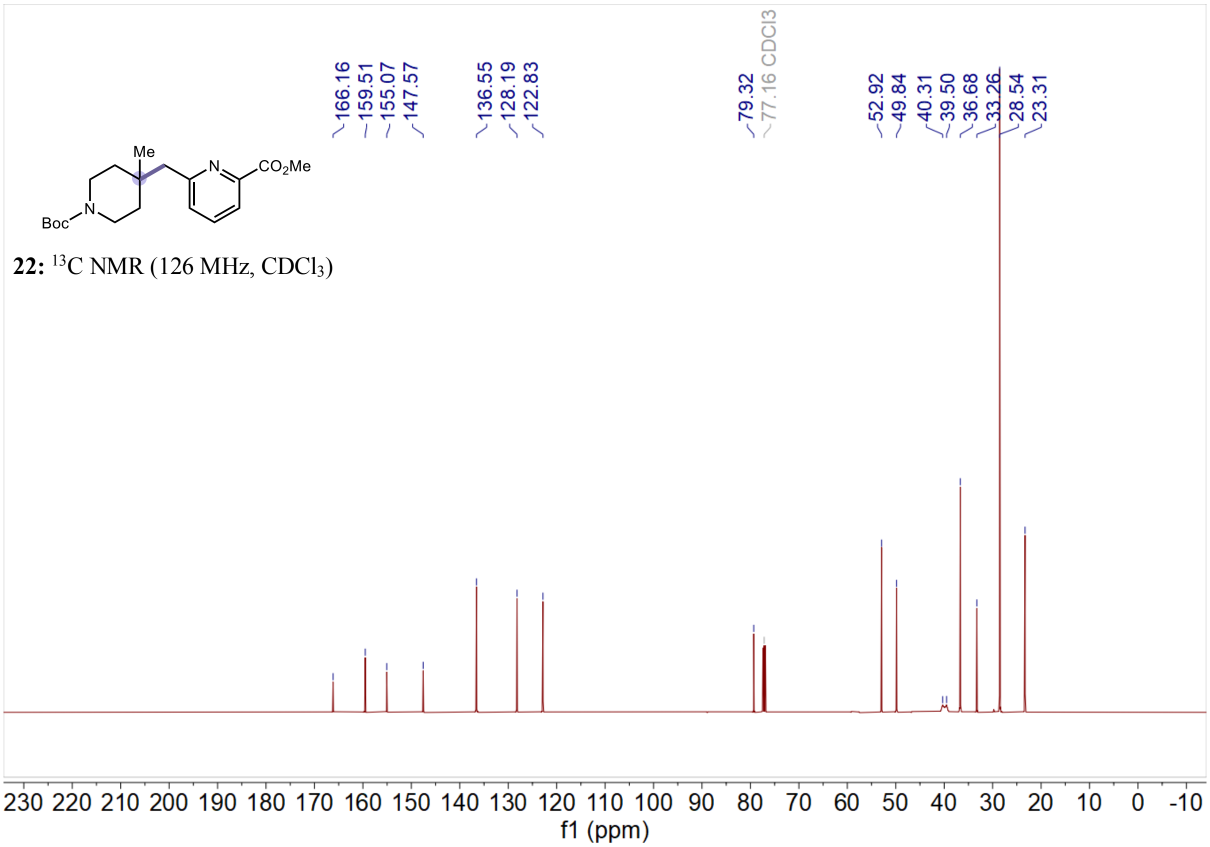

22: ^{13}C NMR (126 MHz, $CDCl_3$)

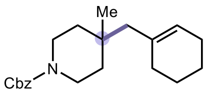

23: ^{1}H NMR (500 MHz, $CDCl_3$)

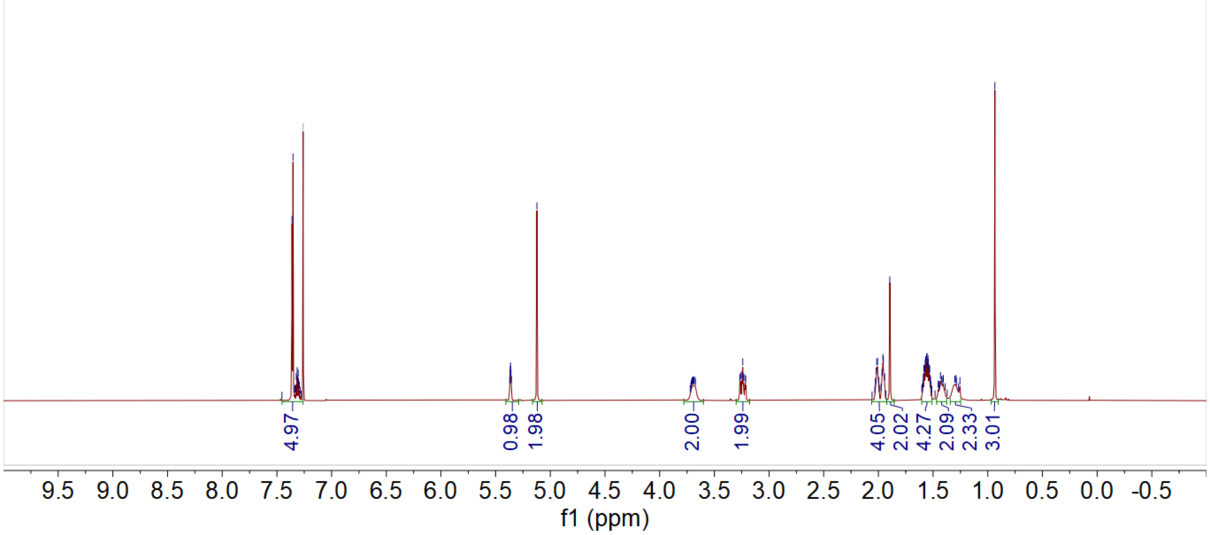

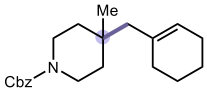

23: ^{13}C NMR (126 MHz, $CDCl_3$)

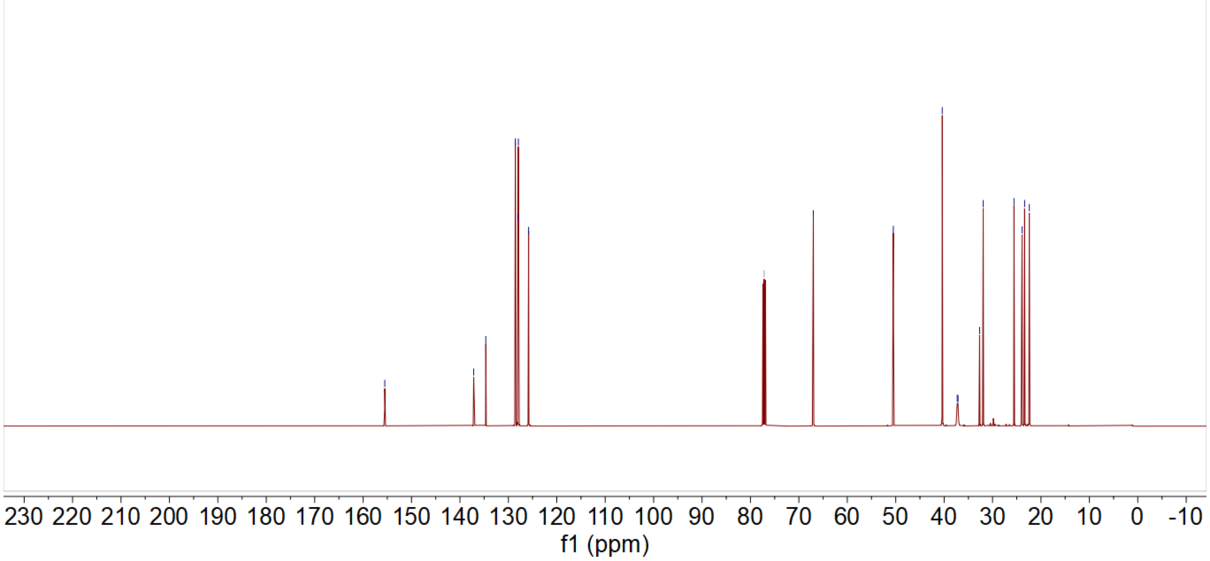

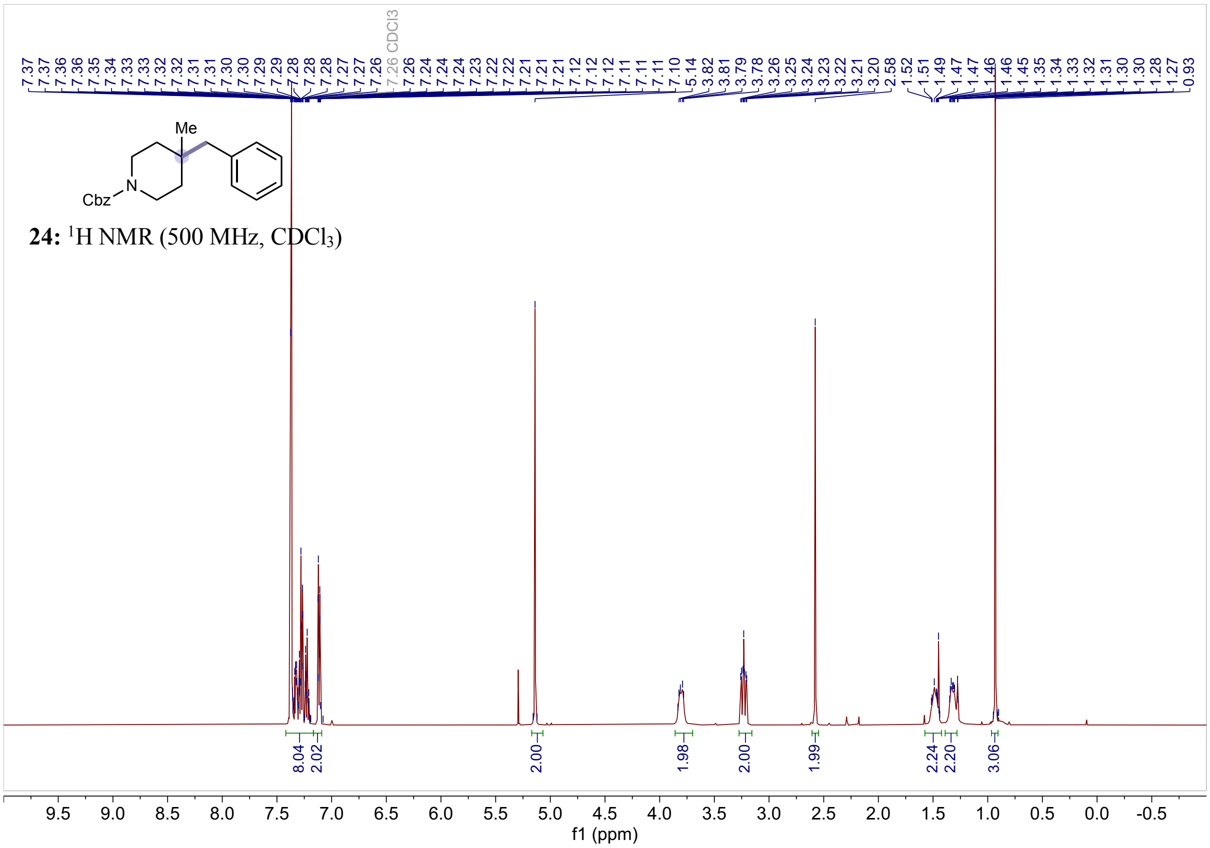

24: ^{1}H NMR (500 MHz, $CDCl_3$)

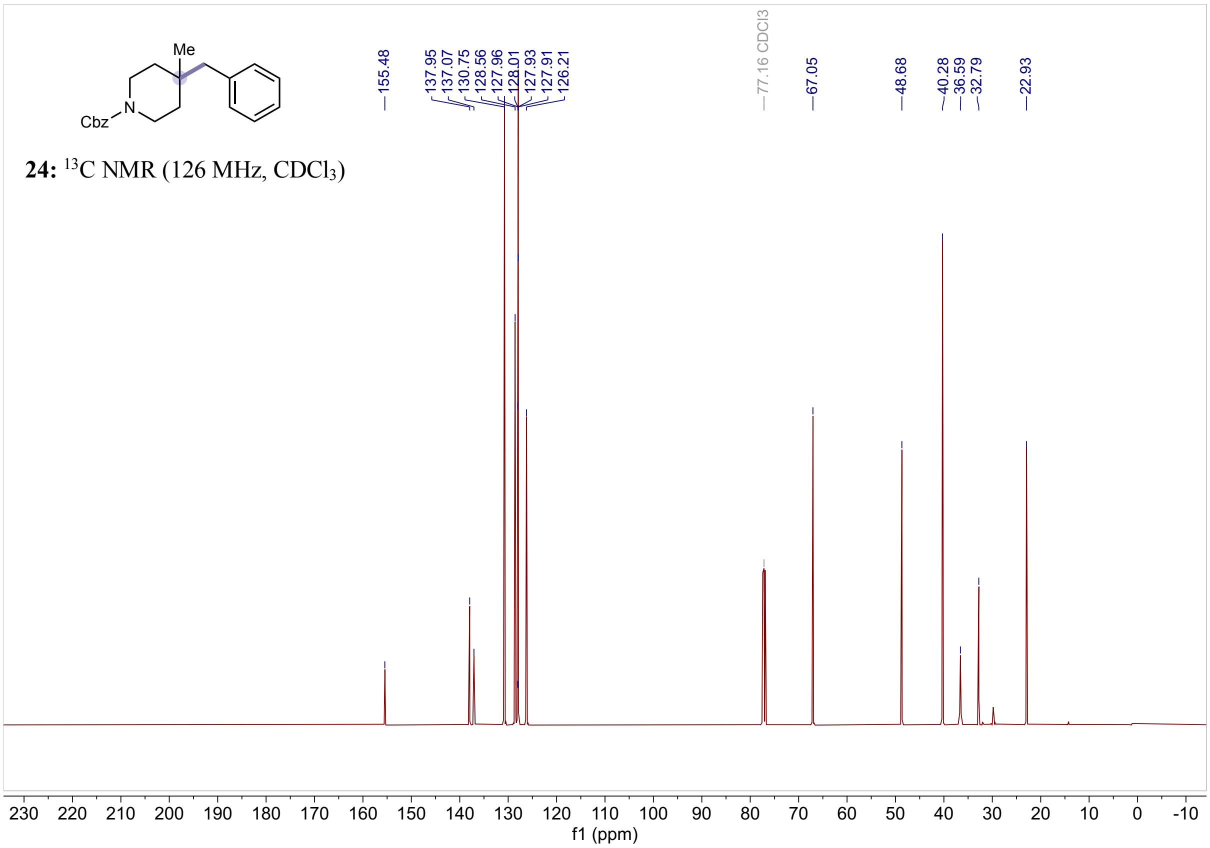

24: ^{13}C NMR (126 MHz, $CDCl_3$)

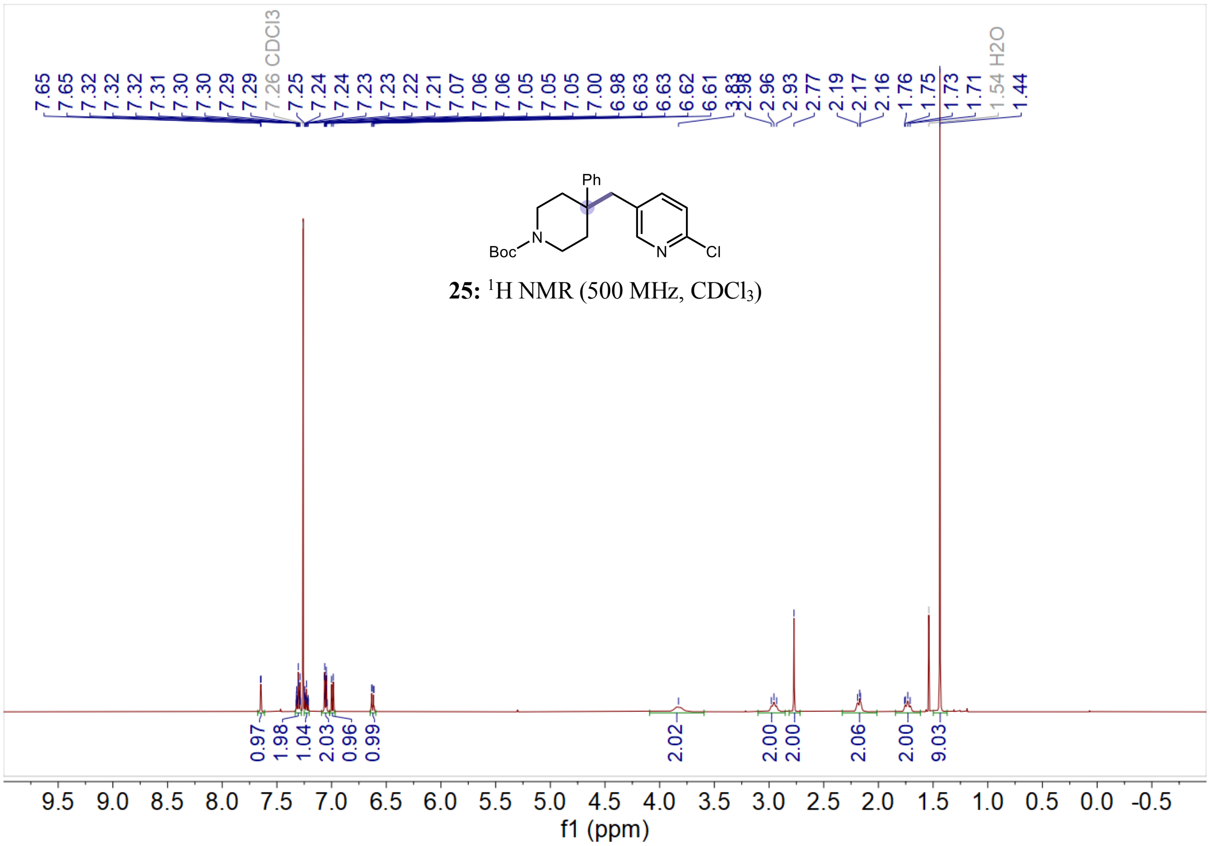

25: ^{1}H NMR (500 MHz, $CDCl_3$)

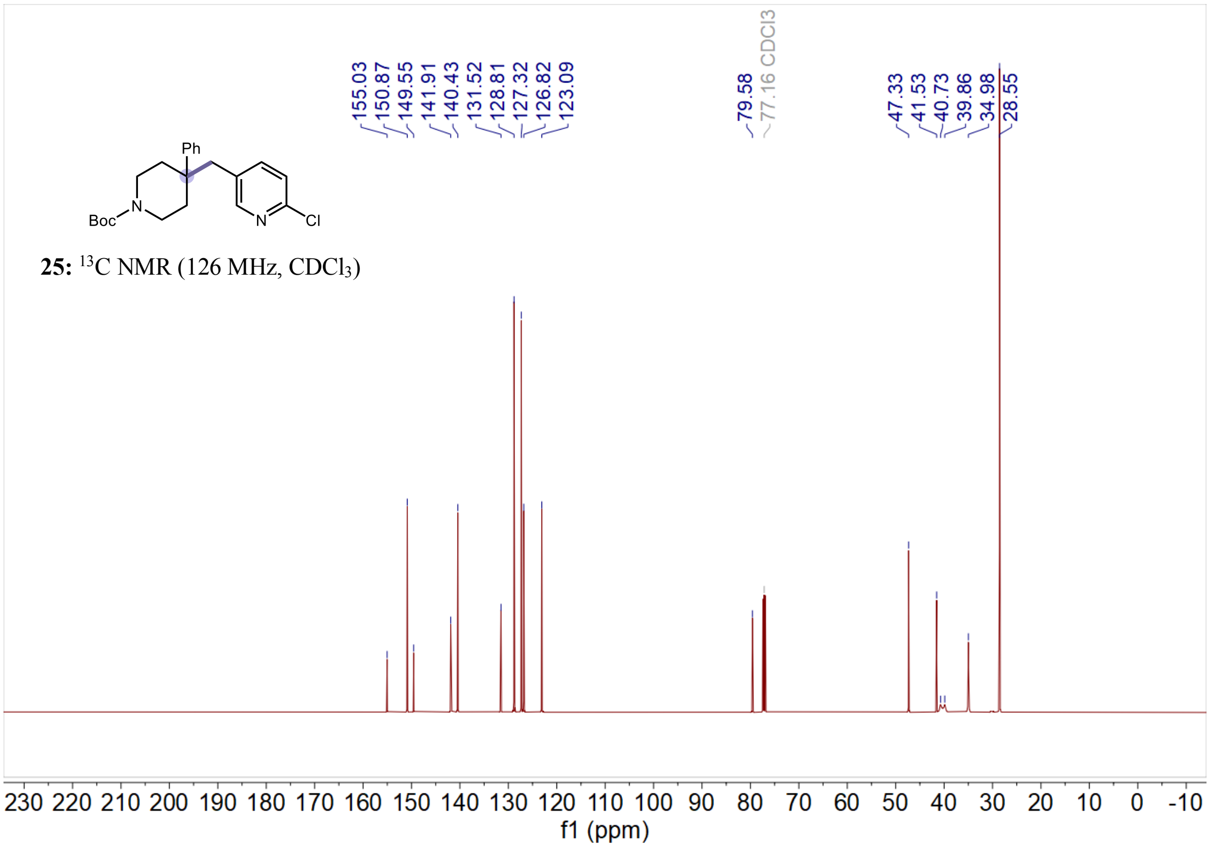

25: ^{13}C NMR (126 MHz, $CDCl_3$)

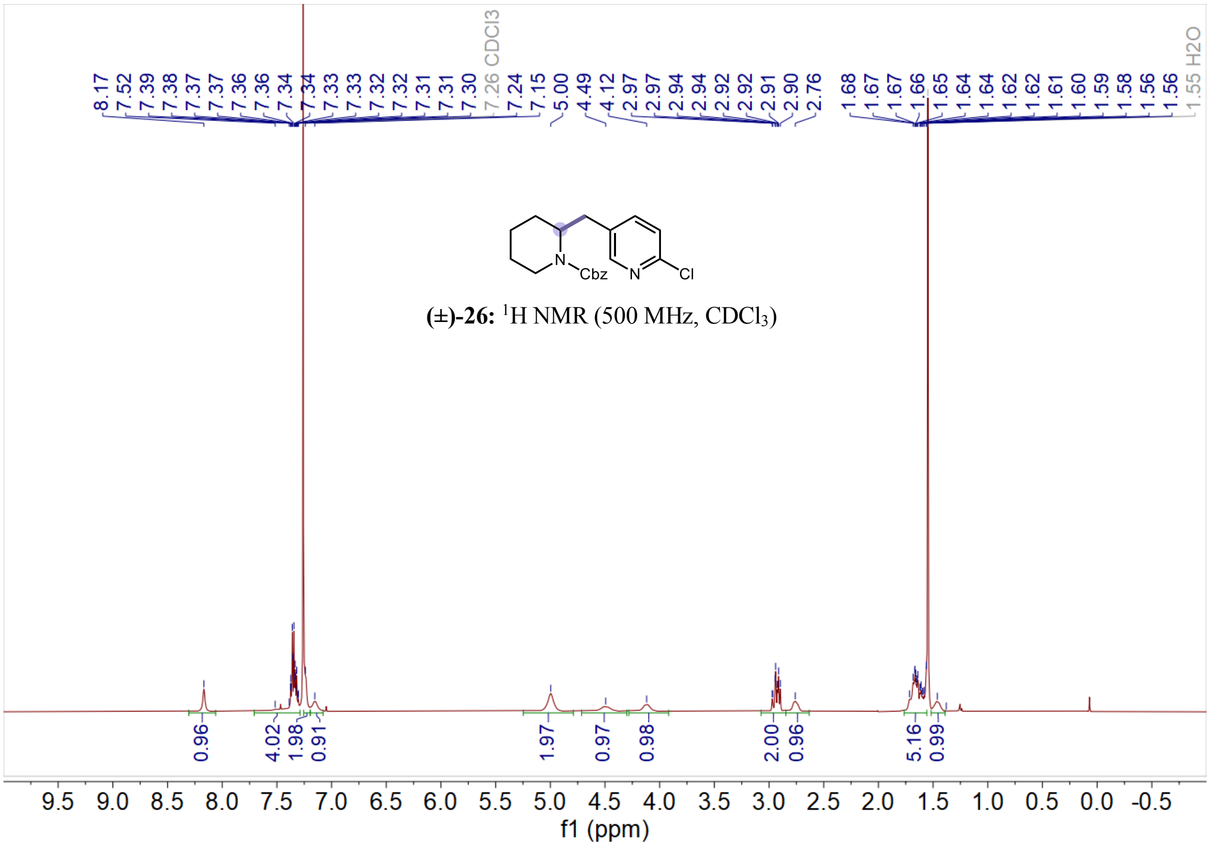

(±)-26: ^{1}H NMR (500 MHz, $CDCl_3$)

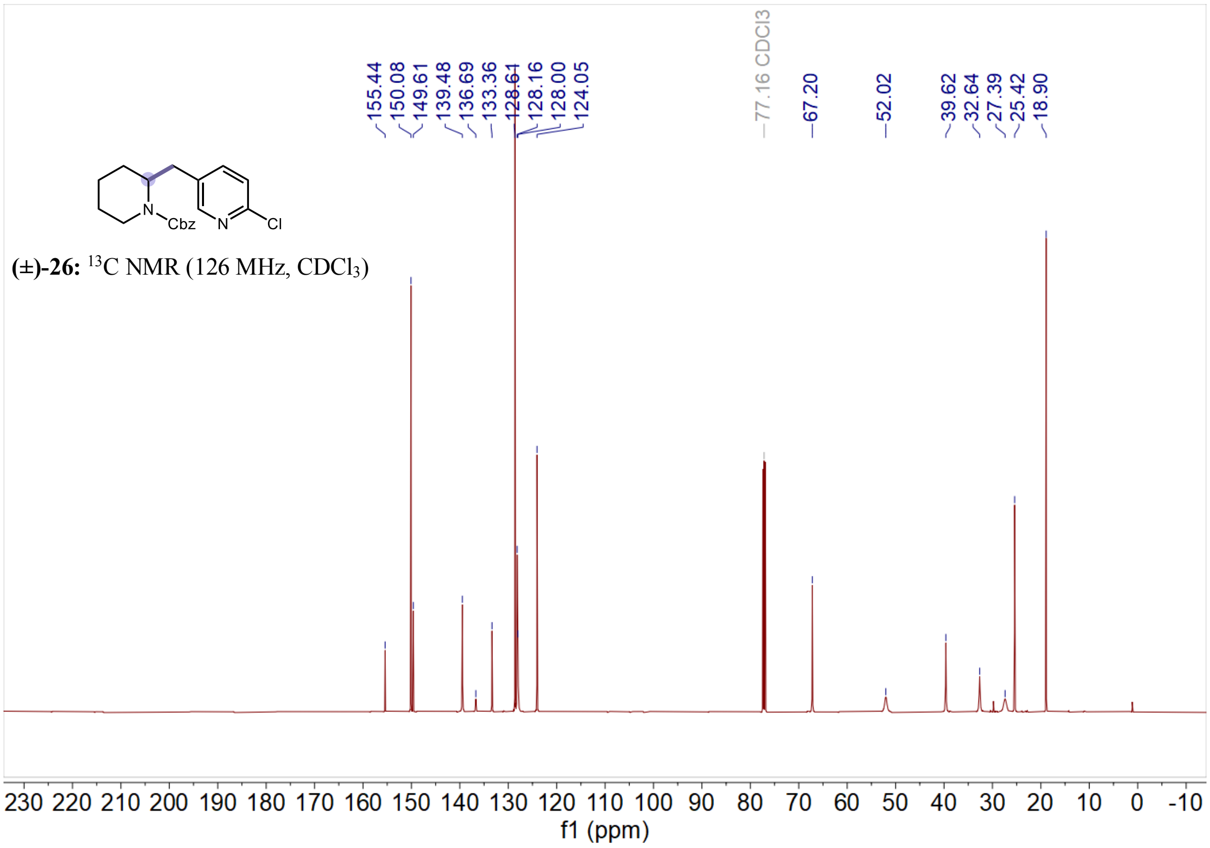

(±)-26: ^{13}C NMR (126 MHz, $CDCl_3$)

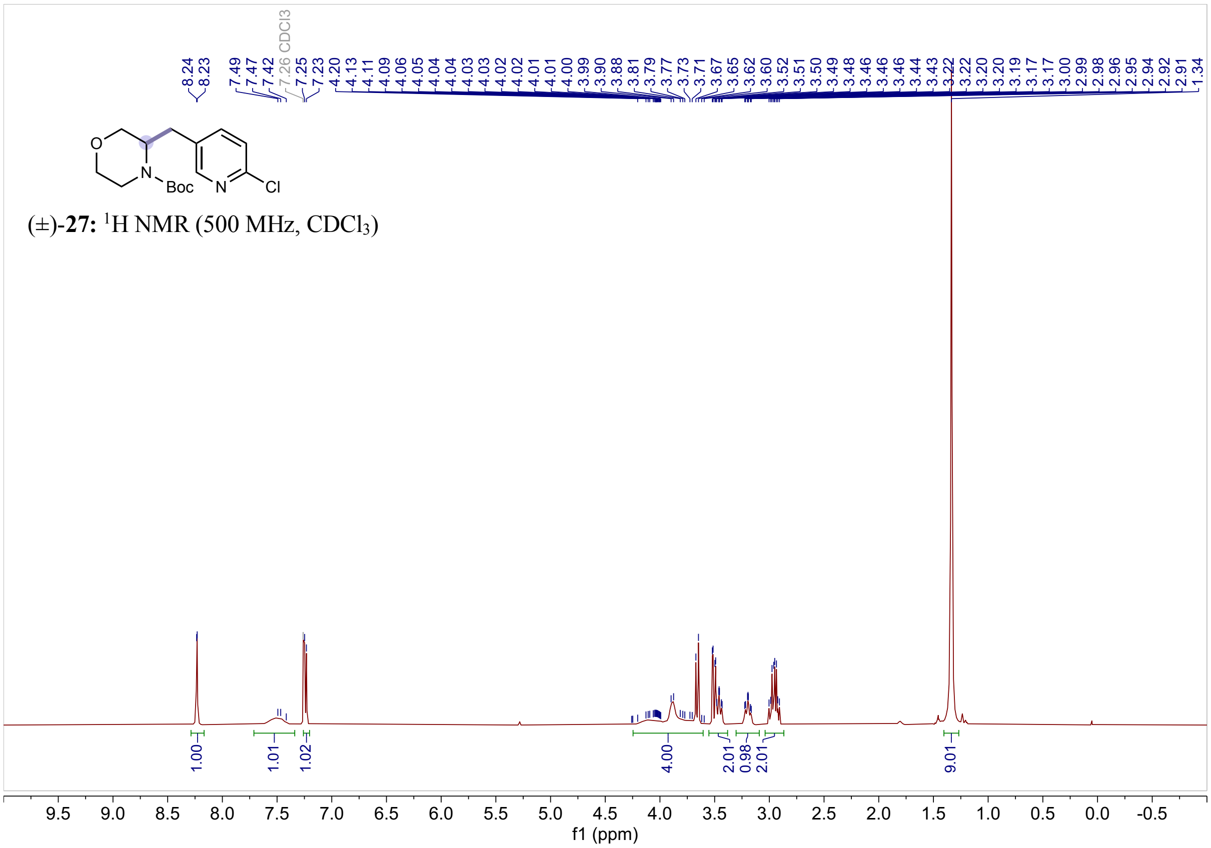

(±)-**27:** ^{1}H NMR (500 MHz, CDCl$_3$)

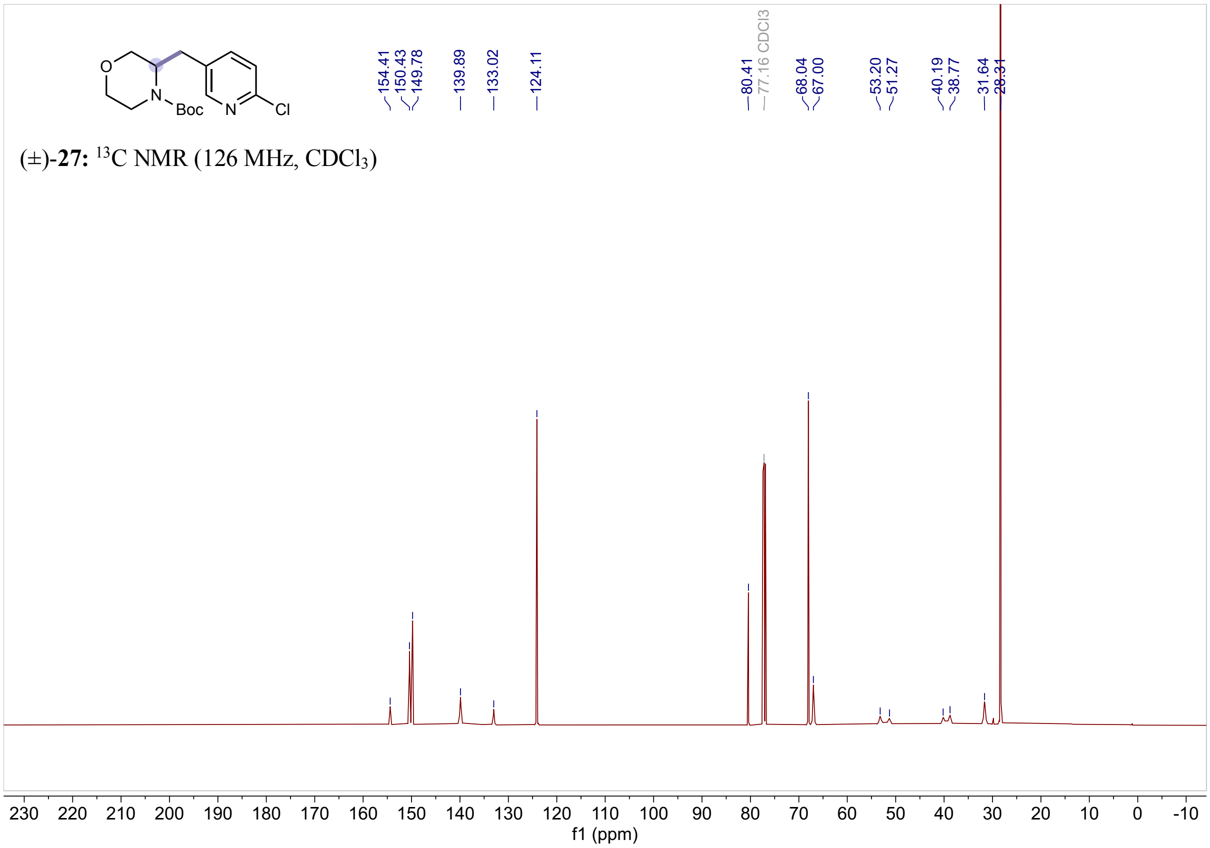

(±)-**27:** ^{13}C NMR (126 MHz, CDCl$_3$)

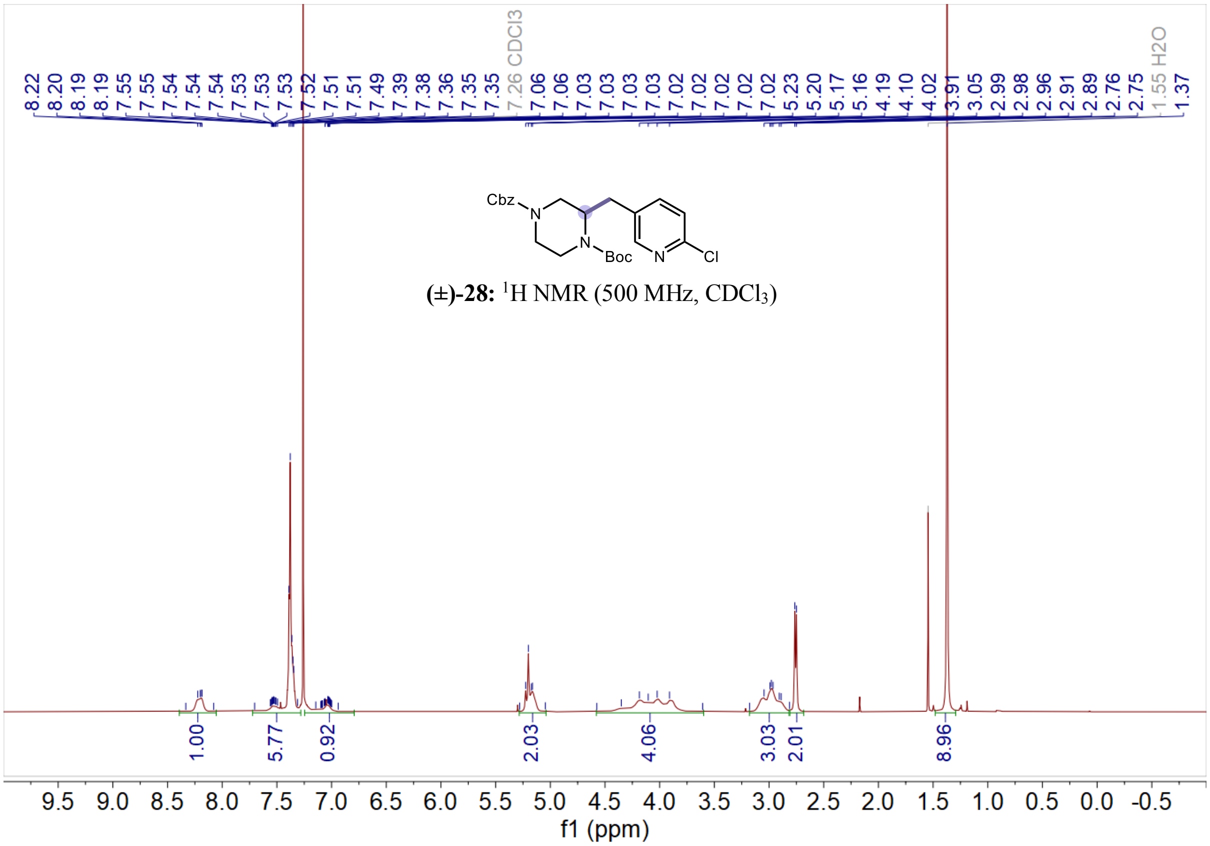

(±)-28: ^{1}H NMR (500 MHz, $CDCl_3$)

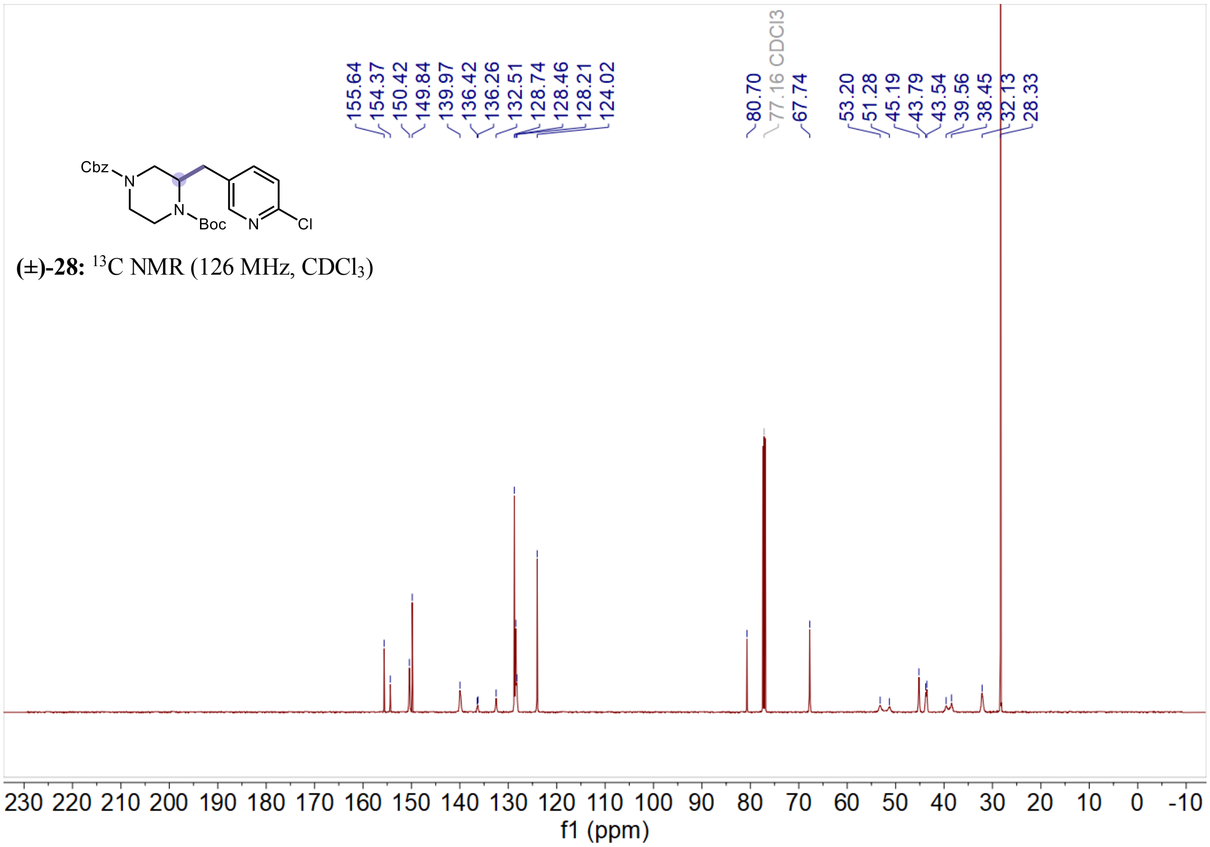

(±)-28: ^{13}C NMR (126 MHz, $CDCl_3$)

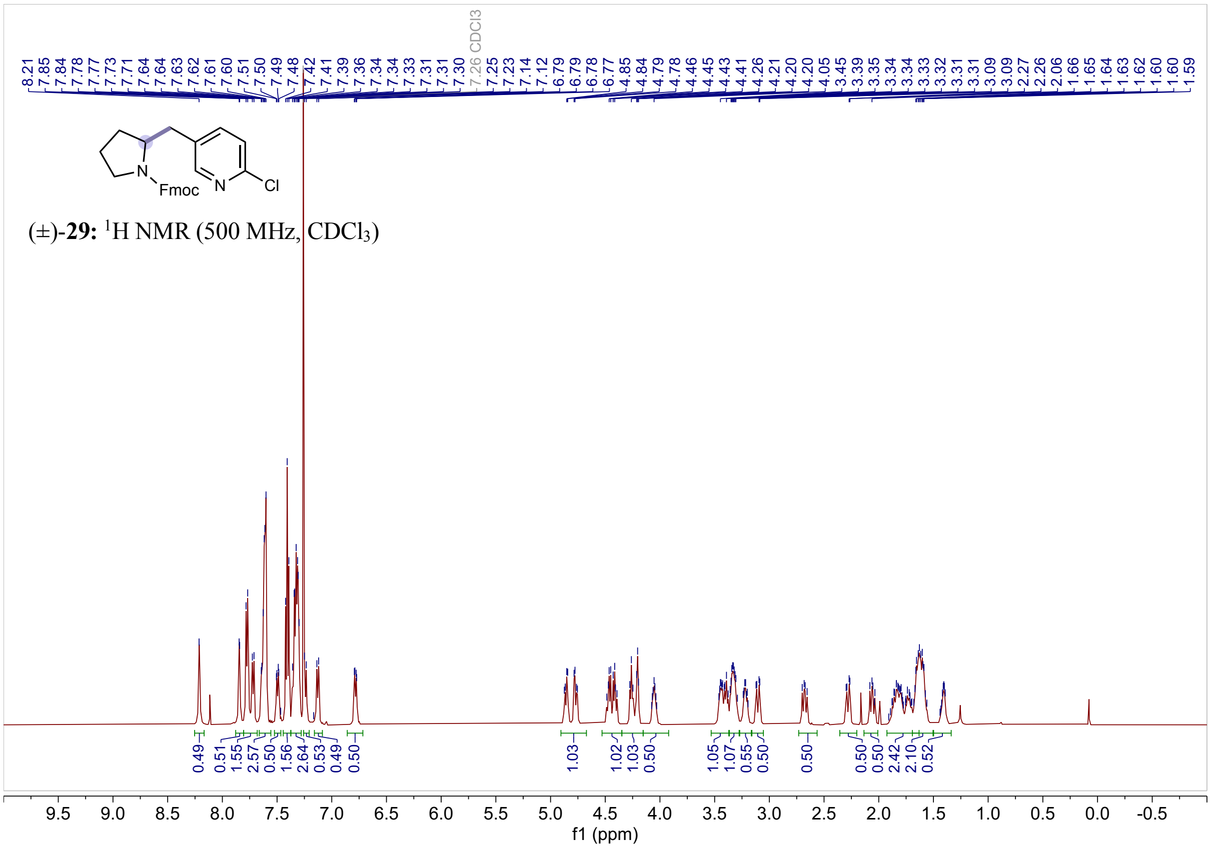
8.21 7.85 7.84 7.78 7.77 7.73 7.71 7.64 7.64 7.63 7.62 7.61 7.60 7.51 7.50 7.49 7.48 7.42 7.41 7.39 7.36 7.34 7.34 7.33 7.31 7.31 7.30 7.26 CDCl3 7.25 7.23 7.14 7.12 6.79 6.79 6.78 6.77 4.85 4.84 4.79 4.78 4.46 4.45 4.43 4.41 4.26 4.21 4.20 4.20 4.05 3.45 3.39 3.35 3.34 3.34 3.33 3.32 3.31 3.31 3.09 3.09 2.27 2.26 2.06 1.66 1.65 1.64 1.63 1.62 1.60 1.60 1.59
Fmoc
N
Cl
(±)-29: ¹H NMR (500 MHz, CDCl₃)
0.49 0.51 1.55 2.57 0.50 1.56 2.64 0.53 0.49 0.50 1.03 1.02 1.03 0.50 1.05 1.07 0.55 0.50 0.50 0.50 0.50 2.42 2.10 0.52
f1 (ppm)

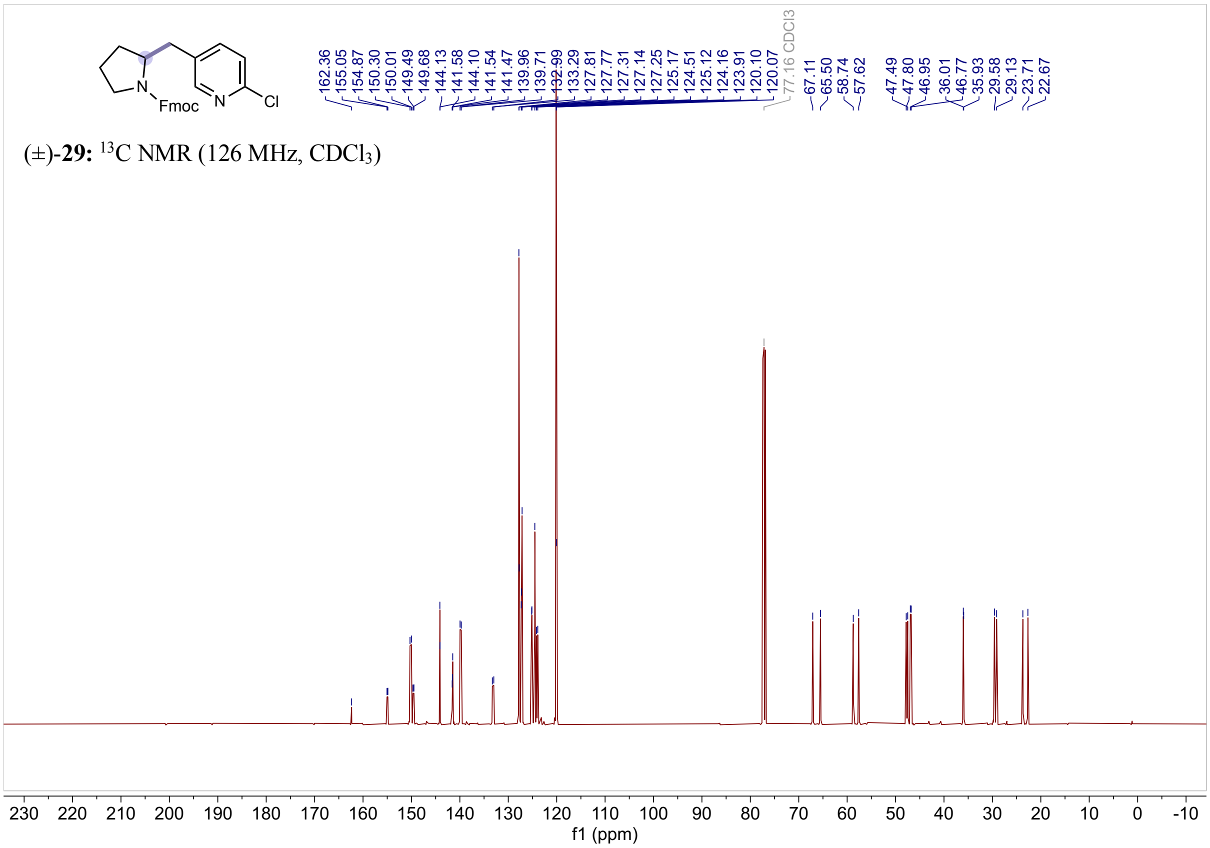
162.36 155.05 154.87 150.30 150.01 149.49 149.68 144.13 141.58 144.10 141.54 141.47 139.96 139.71 132.99 133.29 127.81 127.77 127.31 127.14 127.25 125.17 125.12 124.51 124.16 123.91 120.10 120.07 77.16 CDCl3 67.11 65.50 58.74 57.62 47.49 47.80 46.95 36.01 46.77 35.93 29.58 29.13 23.71 22.67
Fmoc
N
Cl
(±)-29: ¹³C NMR (126 MHz, CDCl₃)
f1 (ppm)

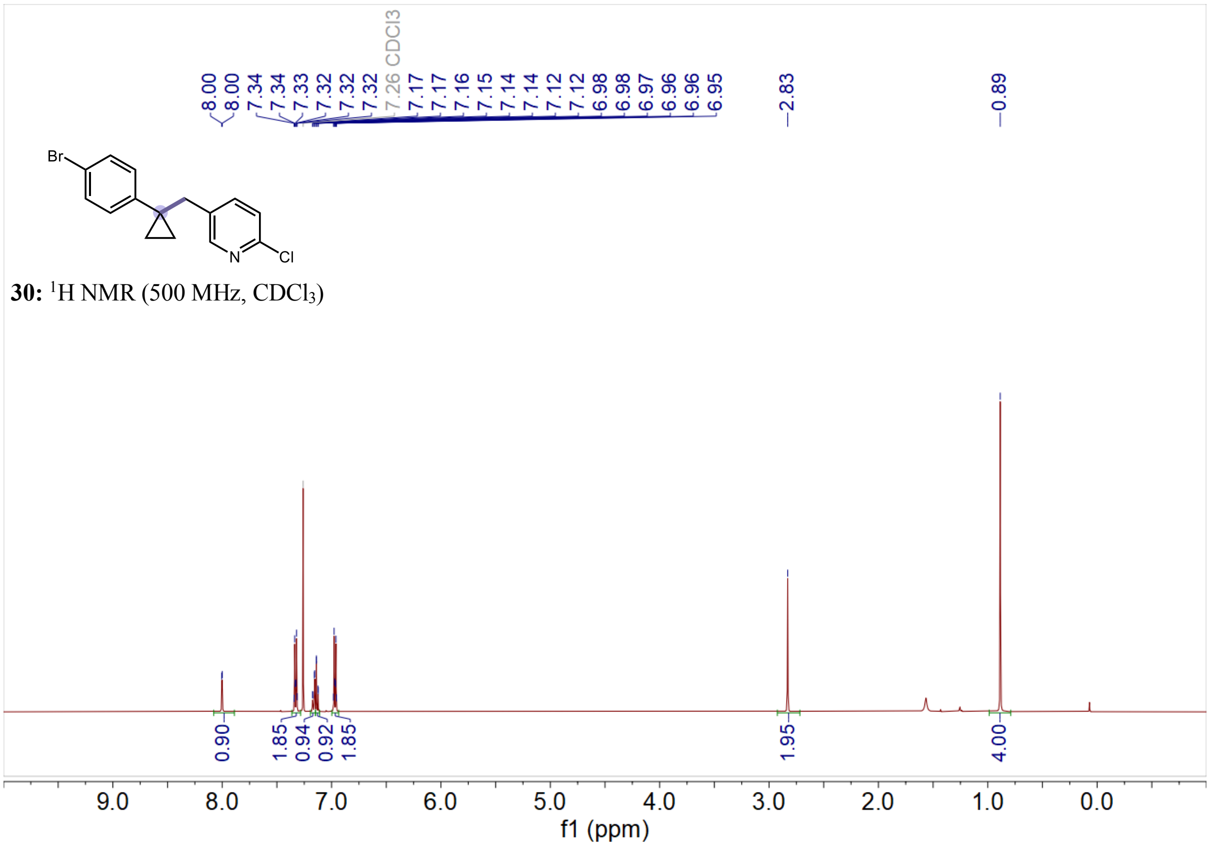

30: ^{1}H NMR (500 MHz, $CDCl_3$)

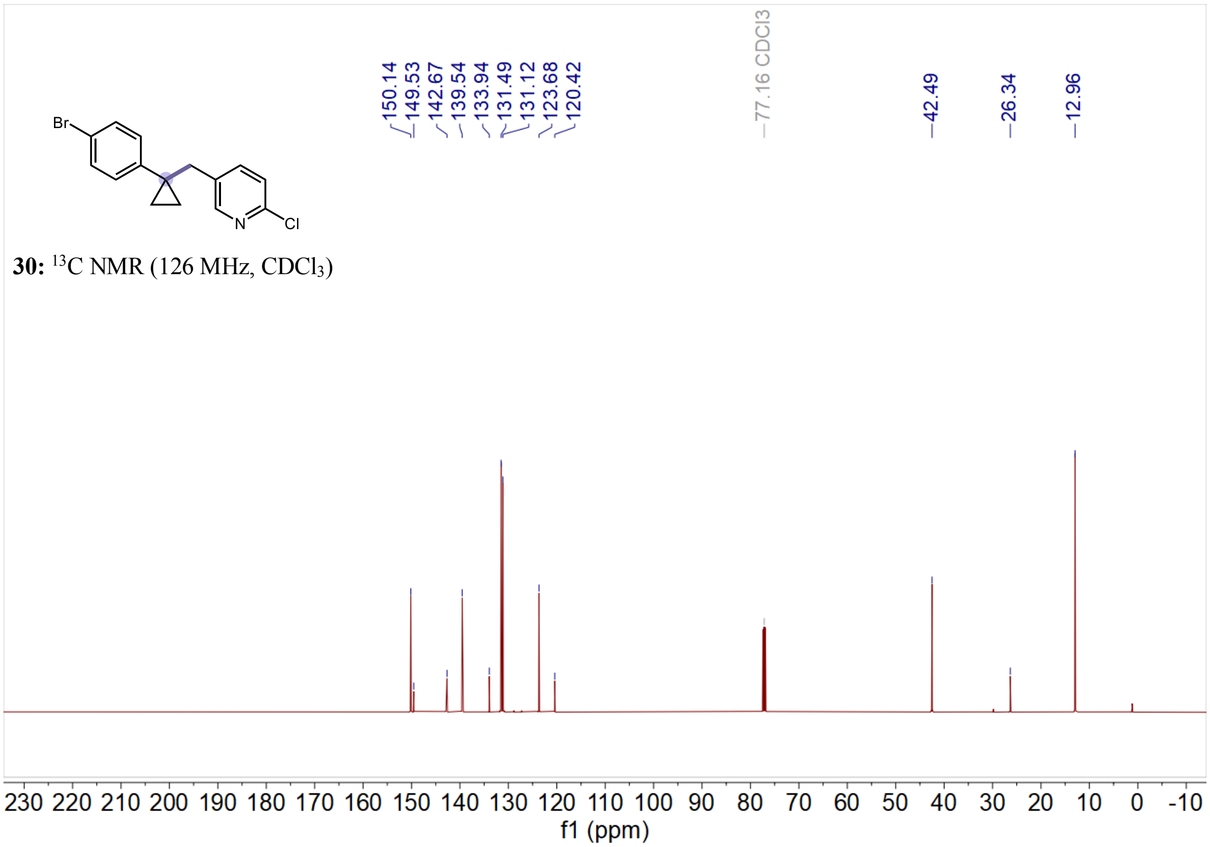

30: ^{13}C NMR (126 MHz, $CDCl_3$)

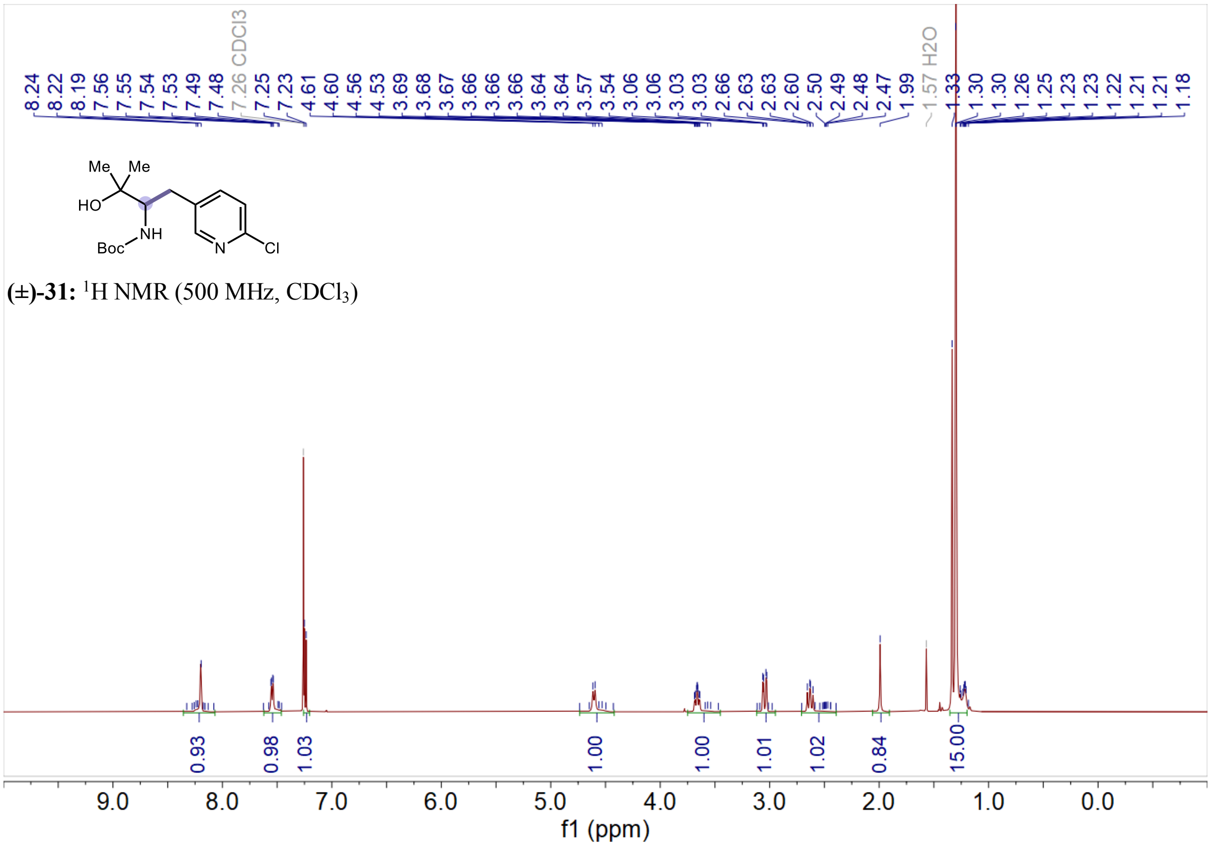

(±)-31: ^{1}H NMR (500 MHz, $CDCl_3$)

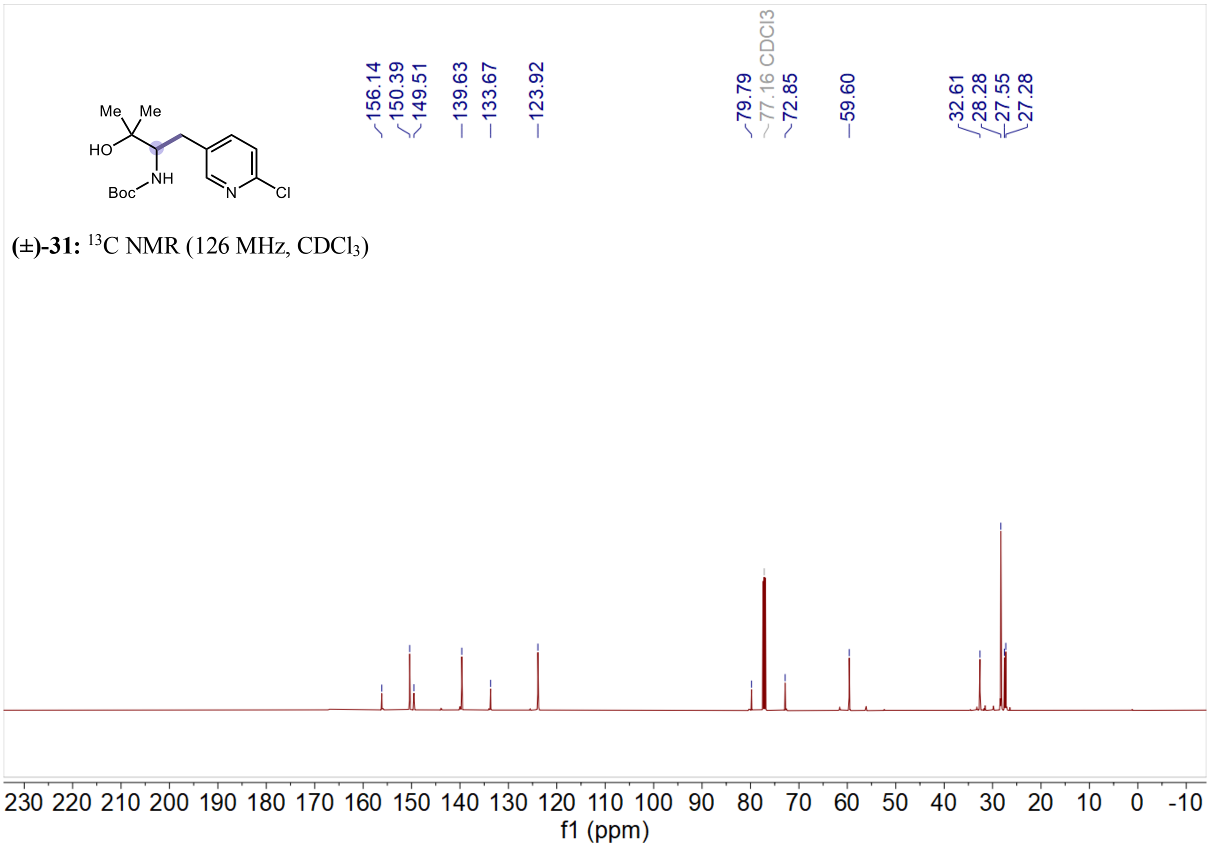

(±)-31: ^{13}C NMR (126 MHz, $CDCl_3$)

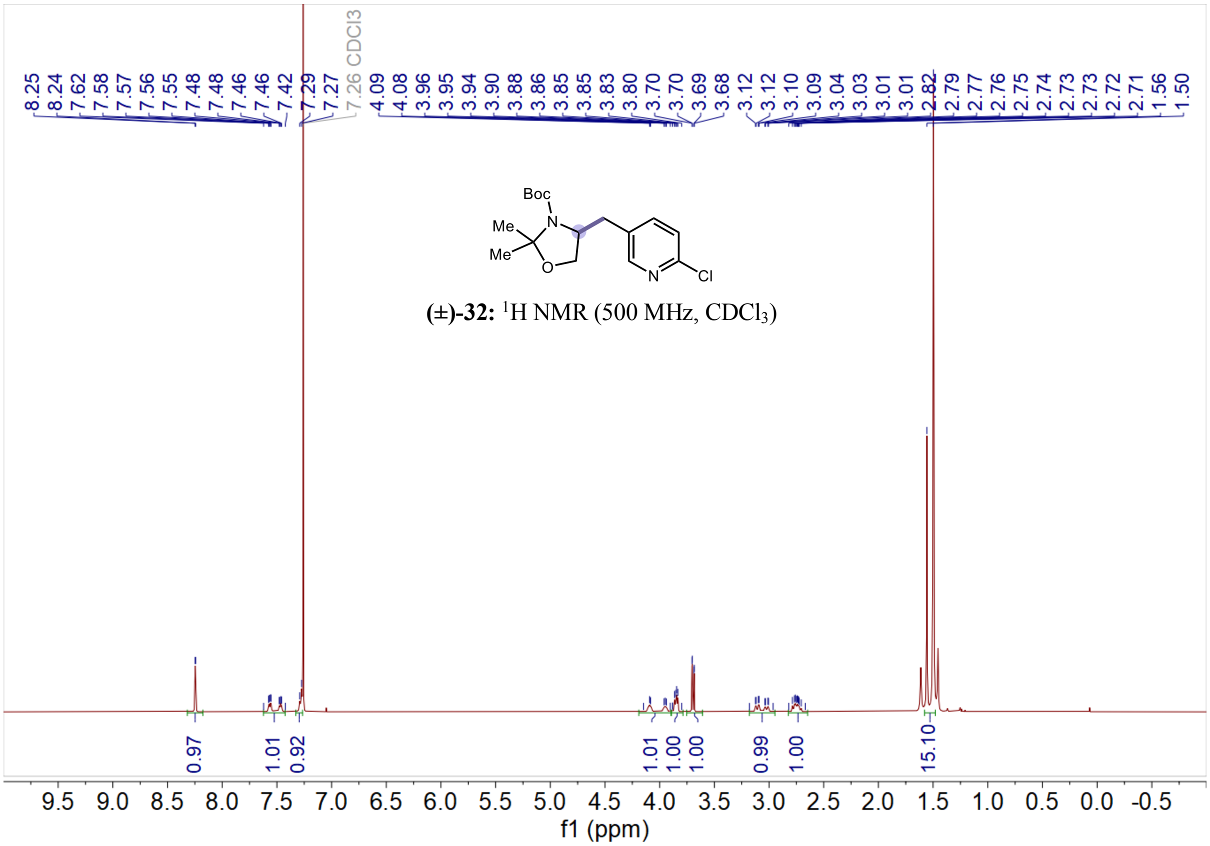
8.25
8.24
7.62
7.58
7.57
7.56
7.55
7.48
7.48
7.46
7.46
7.42
7.29
7.27
7.26 CDCl3
4.09
4.08
3.96
3.95
3.94
3.90
3.88
3.86
3.85
3.85
3.83
3.80
3.70
3.70
3.69
3.68
3.12
3.12
3.10
3.09
3.04
3.03
3.01
3.01
2.82
2.79
2.77
2.76
2.75
2.74
2.73
2.73
2.72
2.71
1.56
1.50
Boc
Me
Me
N
O
N
Cl
(±)-32: 1H NMR (500 MHz, CDCl3)
0.97
1.01
0.92
1.01
1.00
1.00
0.99
1.00
15.10
9.5 9.0 8.5 8.0 7.5 7.0 6.5 6.0 5.5 5.0 4.5 4.0 3.5 3.0 2.5 2.0 1.5 1.0 0.5 0.0 -0.5
f1 (ppm)

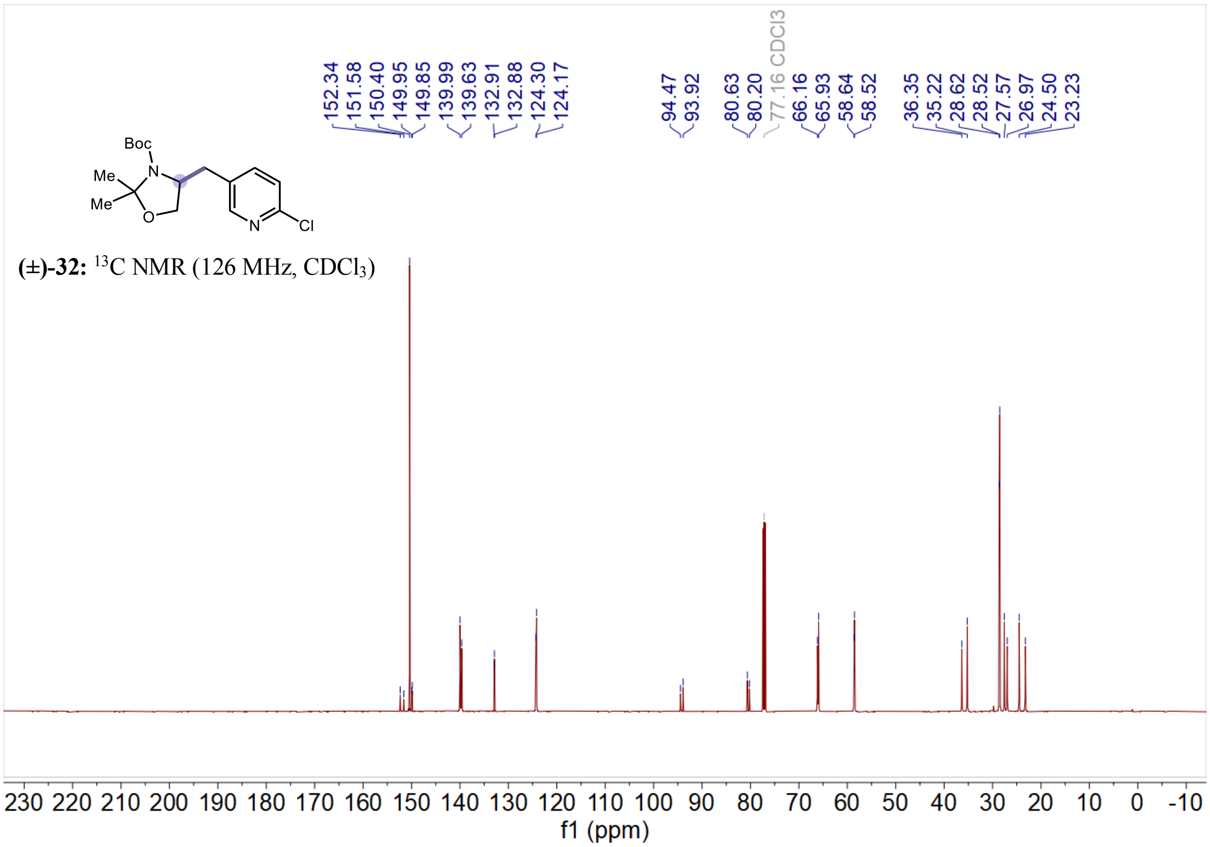
152.34
151.58
150.40
149.95
149.85
139.99
139.63
132.91
132.88
124.30
124.17
94.47
93.92
80.63
80.20
77.16 CDCl3
66.16
65.93
58.64
58.52
36.35
35.22
28.62
28.52
27.57
26.97
24.50
23.23
Boc
Me
Me
N
O
N
Cl
(±)-32: 13C NMR (126 MHz, CDCl3)
230 220 210 200 190 180 170 160 150 140 130 120 110 100 90 80 70 60 50 40 30 20 10 0 -10
f1 (ppm)

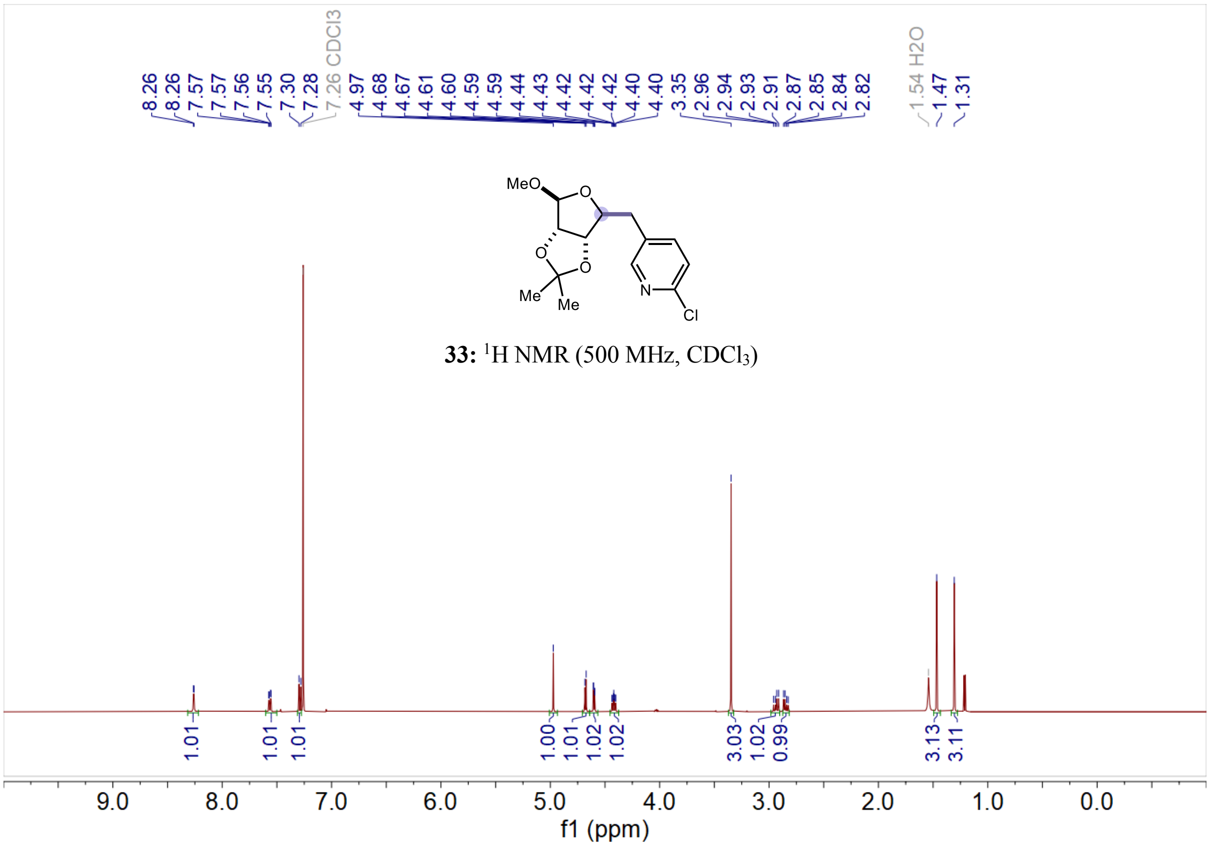

33: ^{1}H NMR (500 MHz, $CDCl_3$)

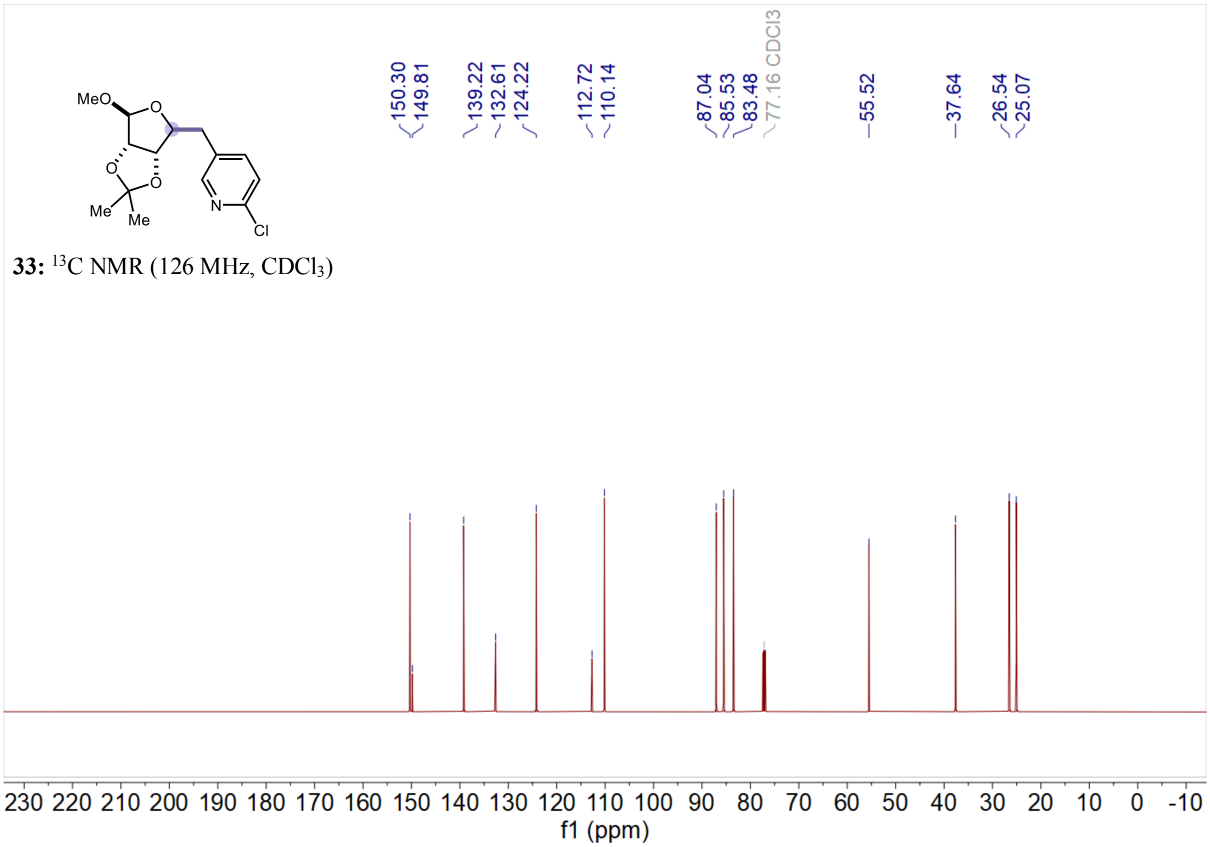

33: ^{13}C NMR (126 MHz, $CDCl_3$)

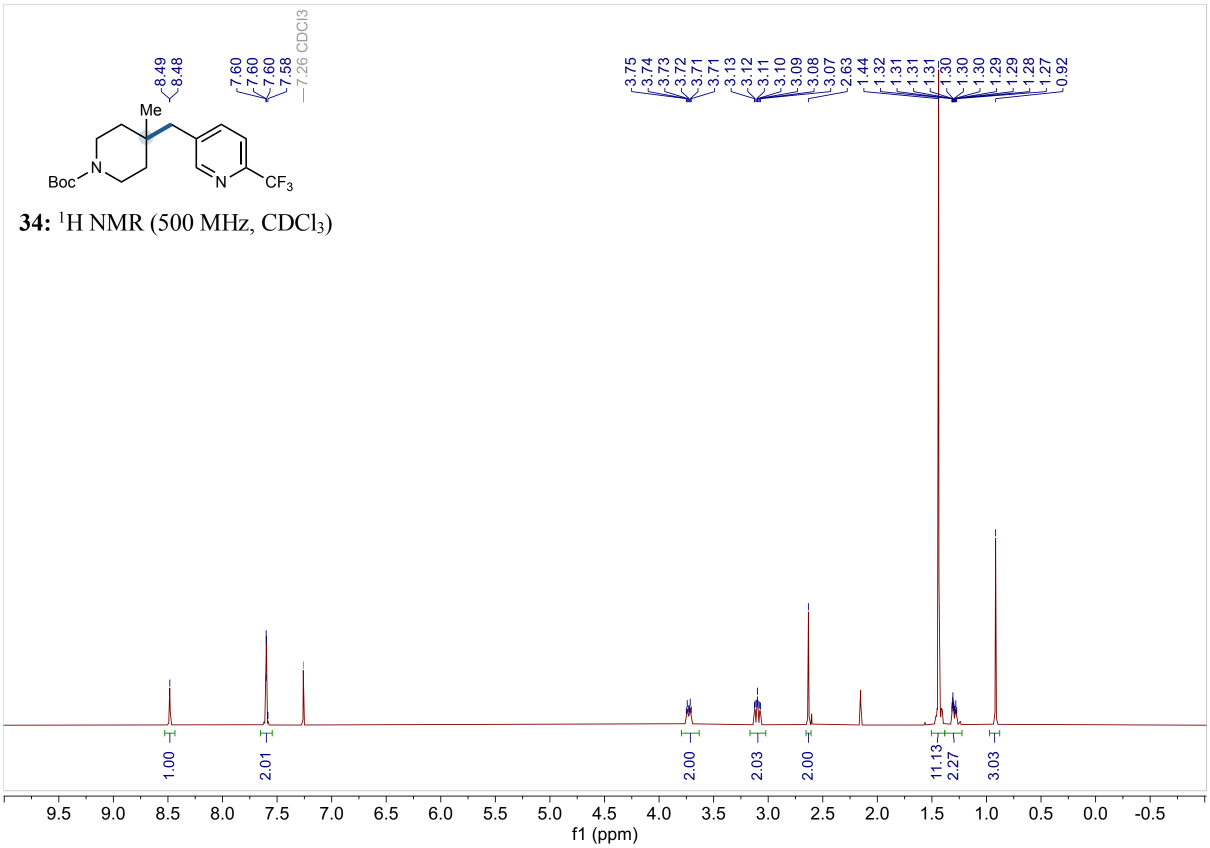
Me
Boc
N
N
CF3
34: 1H NMR (500 MHz, CDCl3)
8.49
8.48
7.60
7.60
7.60
7.58
7.26 CDCl3
3.75
3.74
3.73
3.72
3.71
3.71
3.13
3.12
3.11
3.10
3.09
3.08
3.07
2.63
1.44
1.32
1.31
1.31
1.31
1.30
1.30
1.30
1.29
1.29
1.28
1.27
0.92
1.00
2.01
2.00
2.03
2.00
11.13
2.27
3.03
f1 (ppm)

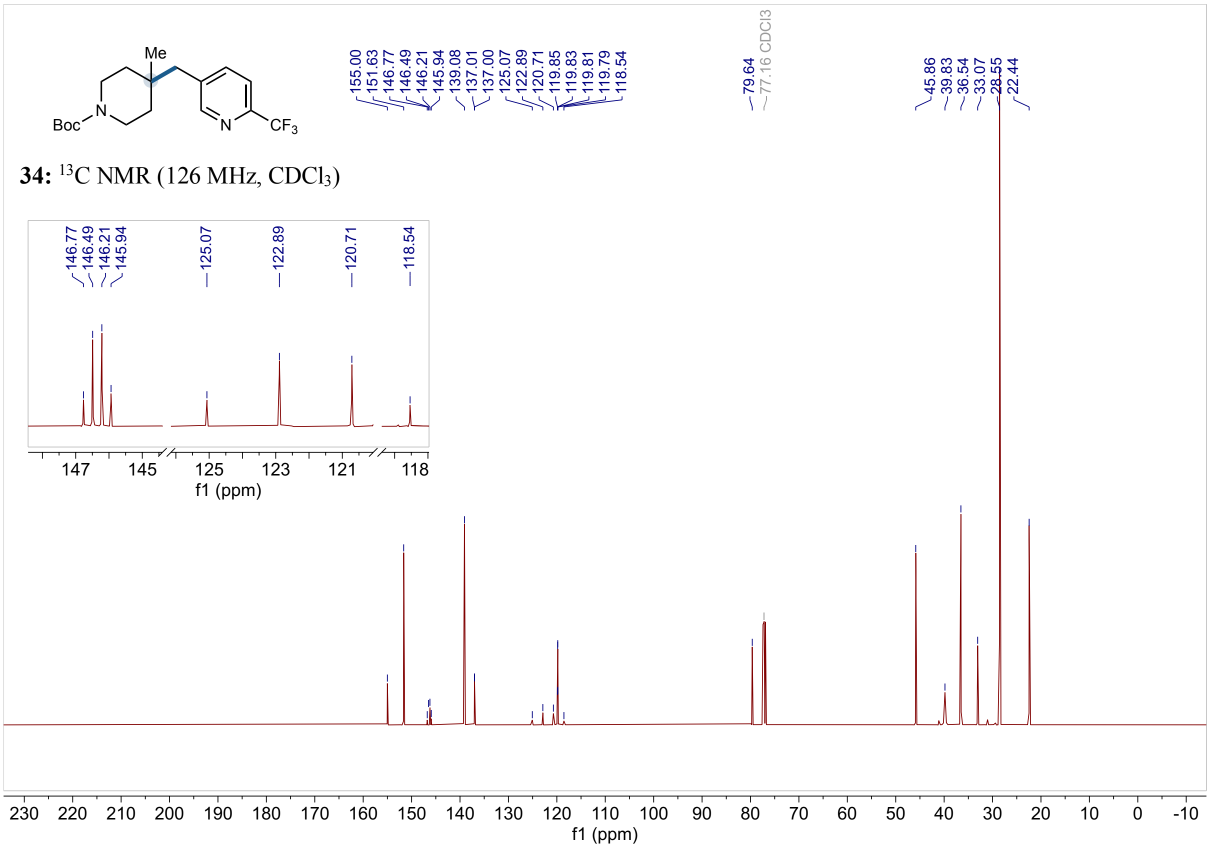
Me
Boc
N
N
CF3
34: 13C NMR (126 MHz, CDCl3)
155.00
151.63
146.77
146.49
146.21
145.94
139.08
137.01
137.00
125.07
122.89
120.71
119.85
119.83
119.81
119.79
118.54
79.64
77.16 CDCl3
45.86
39.83
36.54
33.07
28.55
22.44
f1 (ppm)

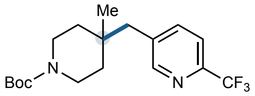

34: ^{19}F NMR (471 MHz, $CDCl_3$)

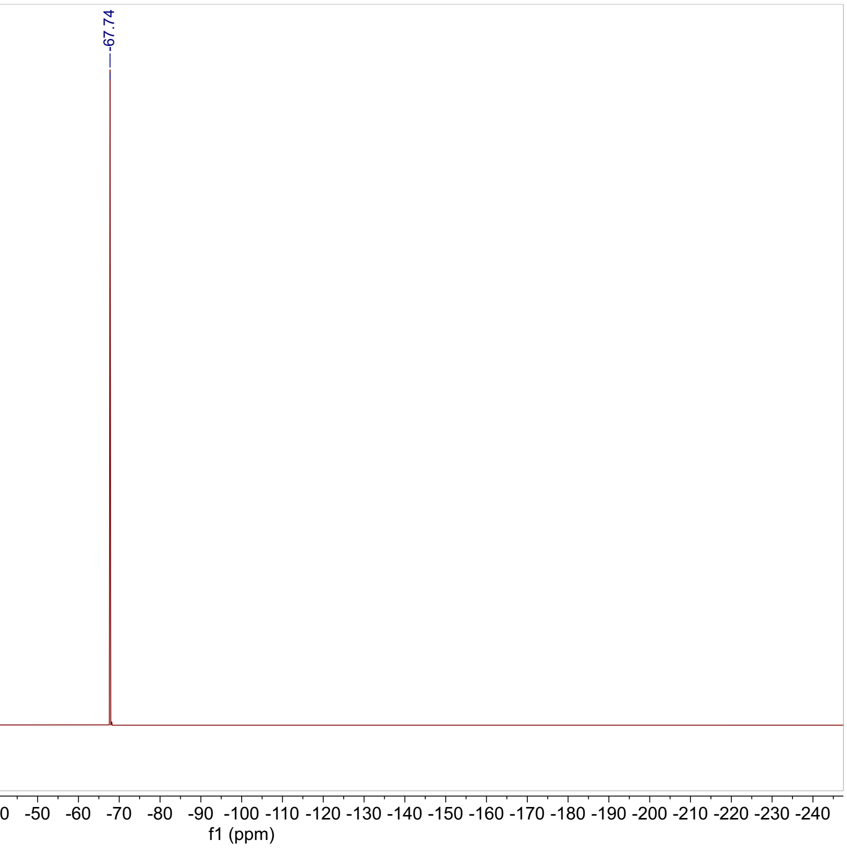

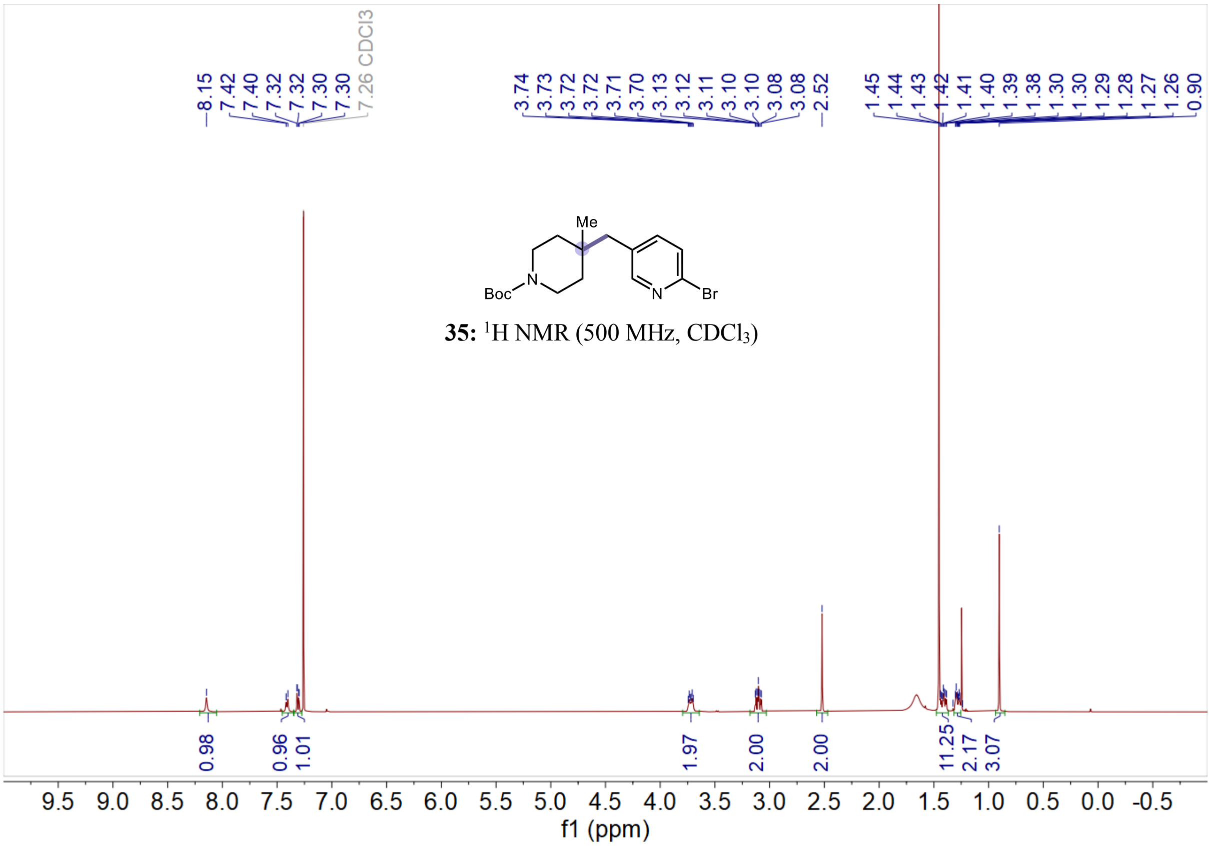

35: ^{1}H NMR (500 MHz, $CDCl_3$)

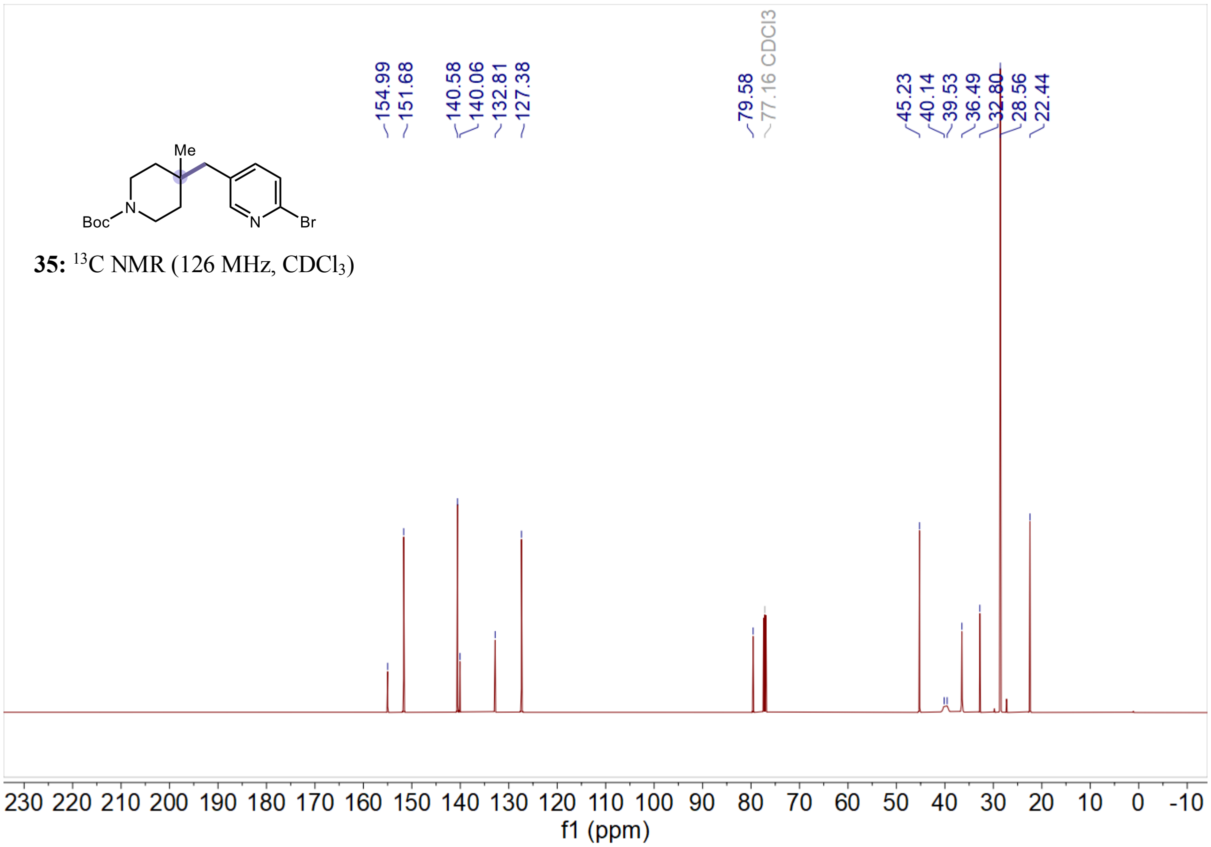

35: ^{13}C NMR (126 MHz, $CDCl_3$)

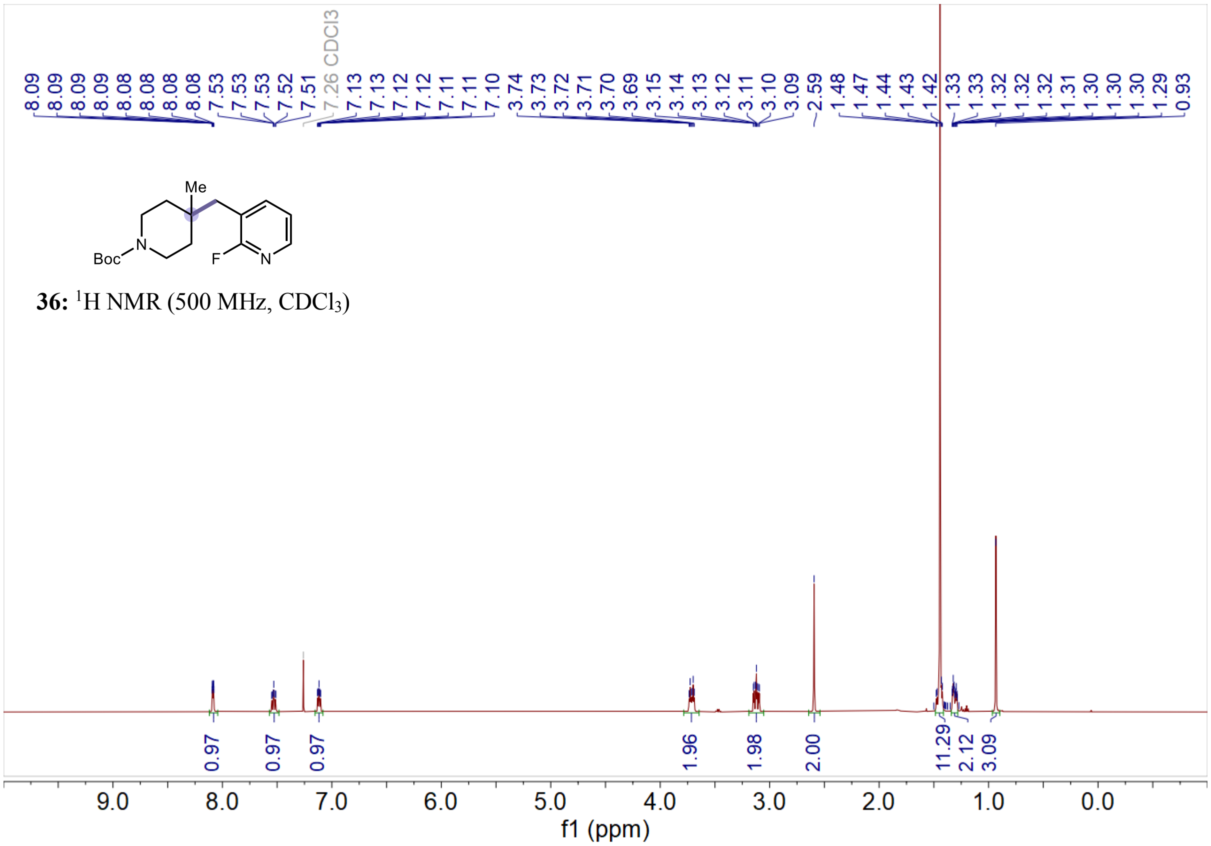

36: ^{1}H NMR (500 MHz, $CDCl_3$)

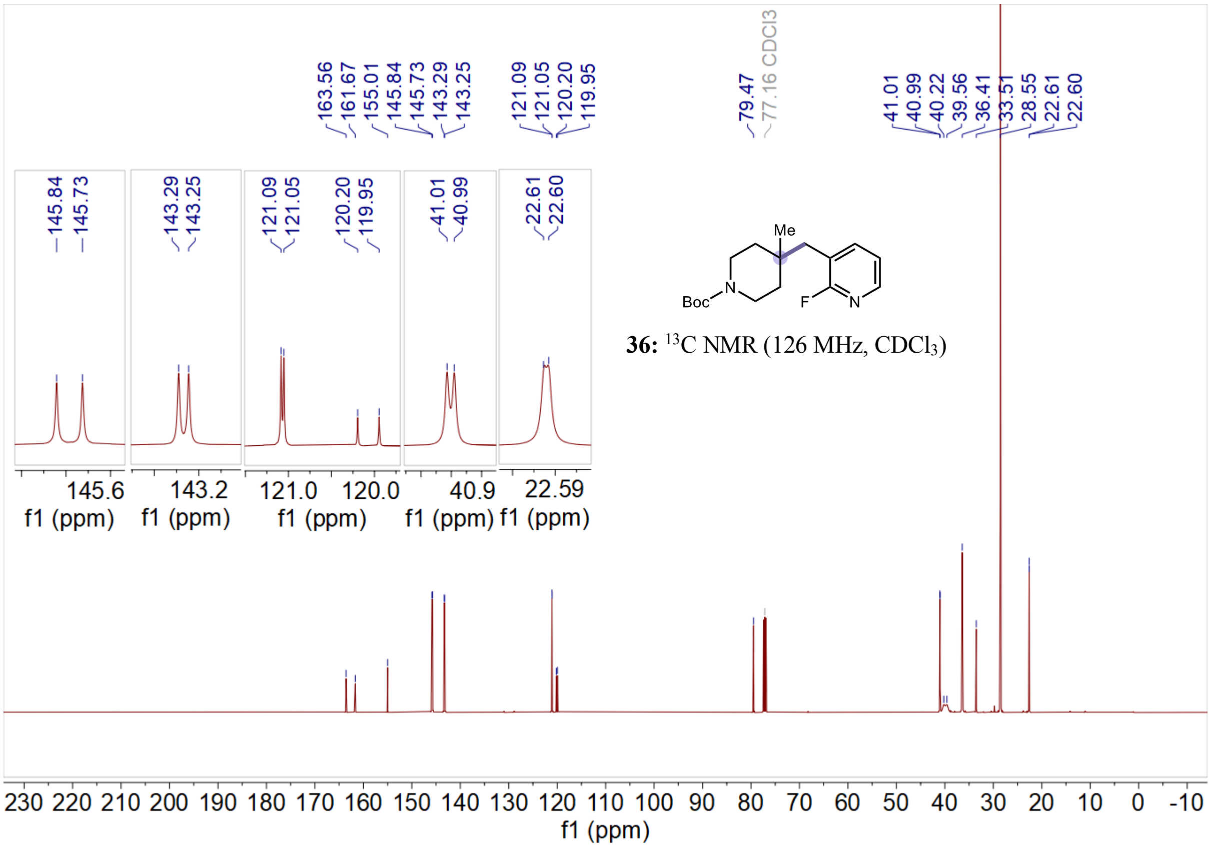

36: ^{13}C NMR (126 MHz, $CDCl_3$)

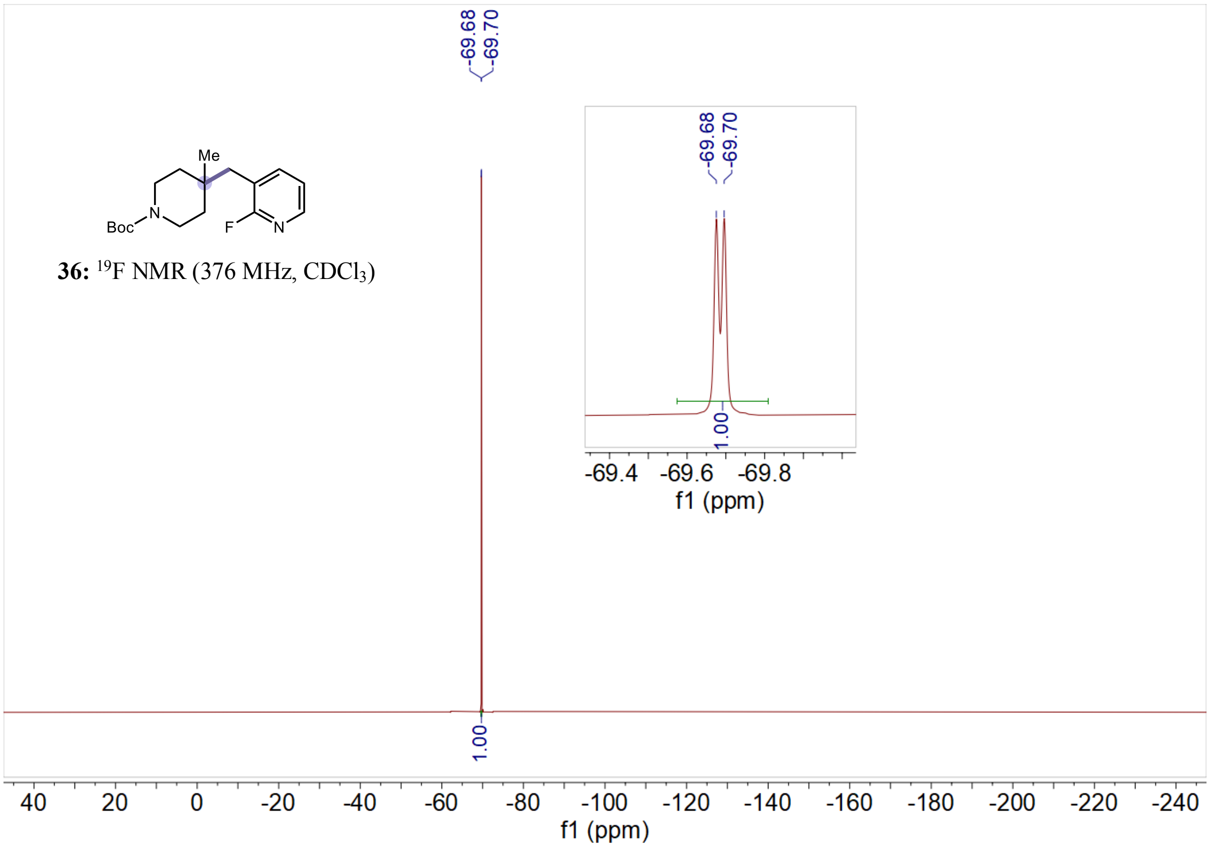

36: ^{19}F NMR (376 MHz, $CDCl_3$)

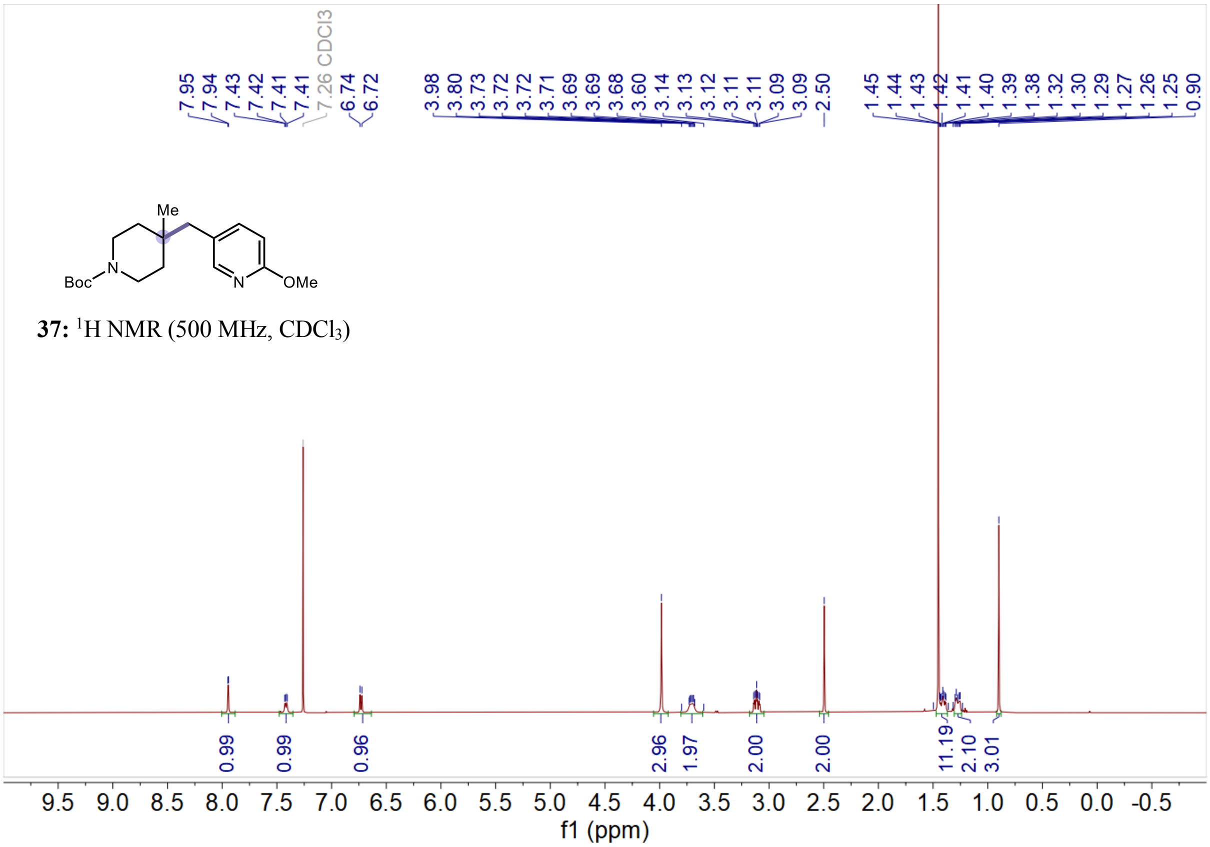

37: ^{1}H NMR (500 MHz, $CDCl_3$)

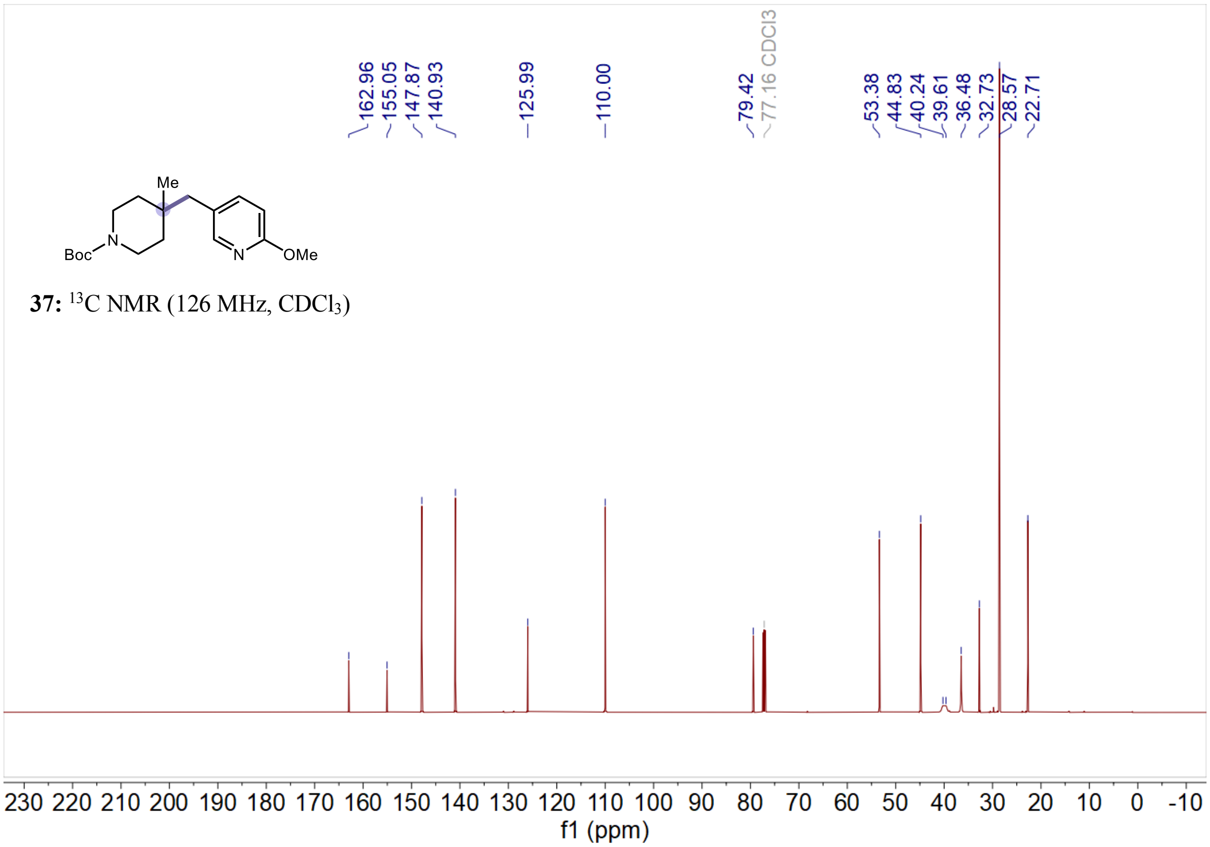

37: ^{13}C NMR (126 MHz, $CDCl_3$)

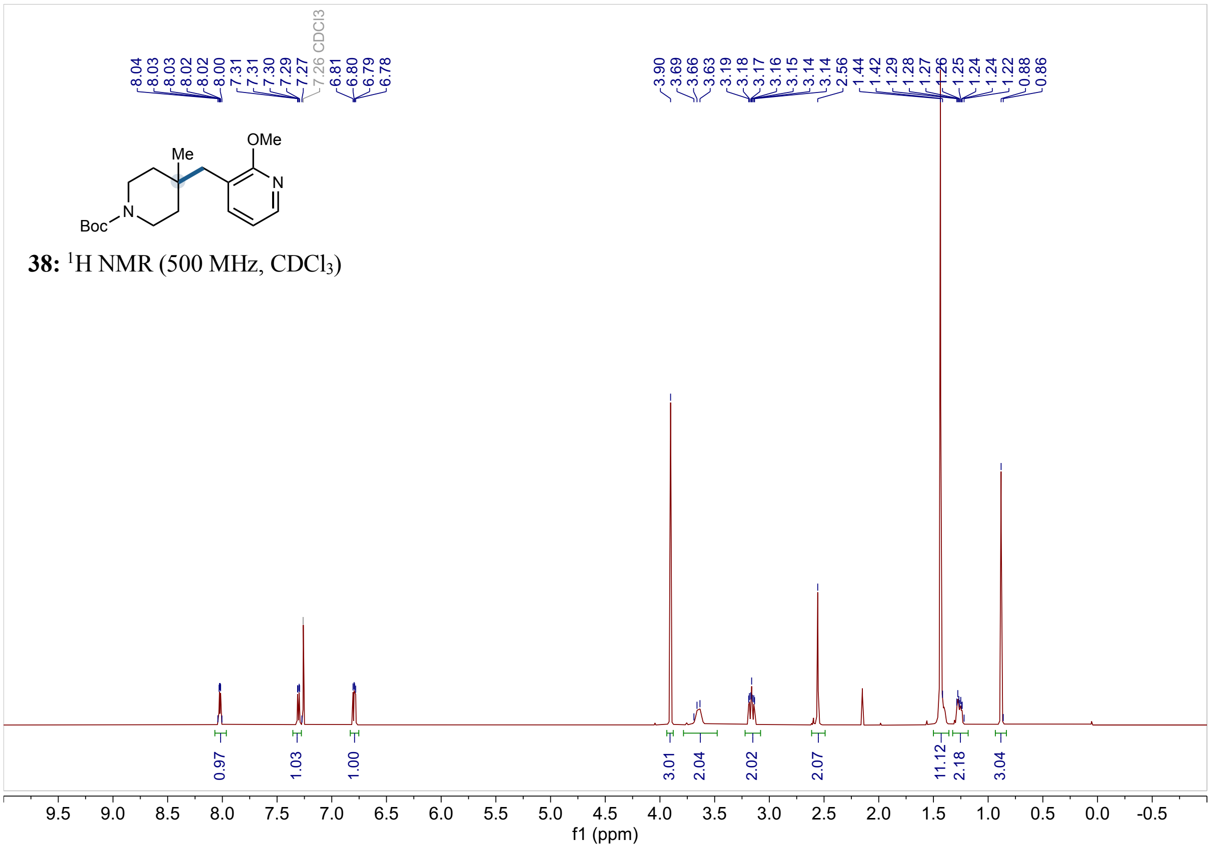

38: ^{1}H NMR (500 MHz, $CDCl_3$)

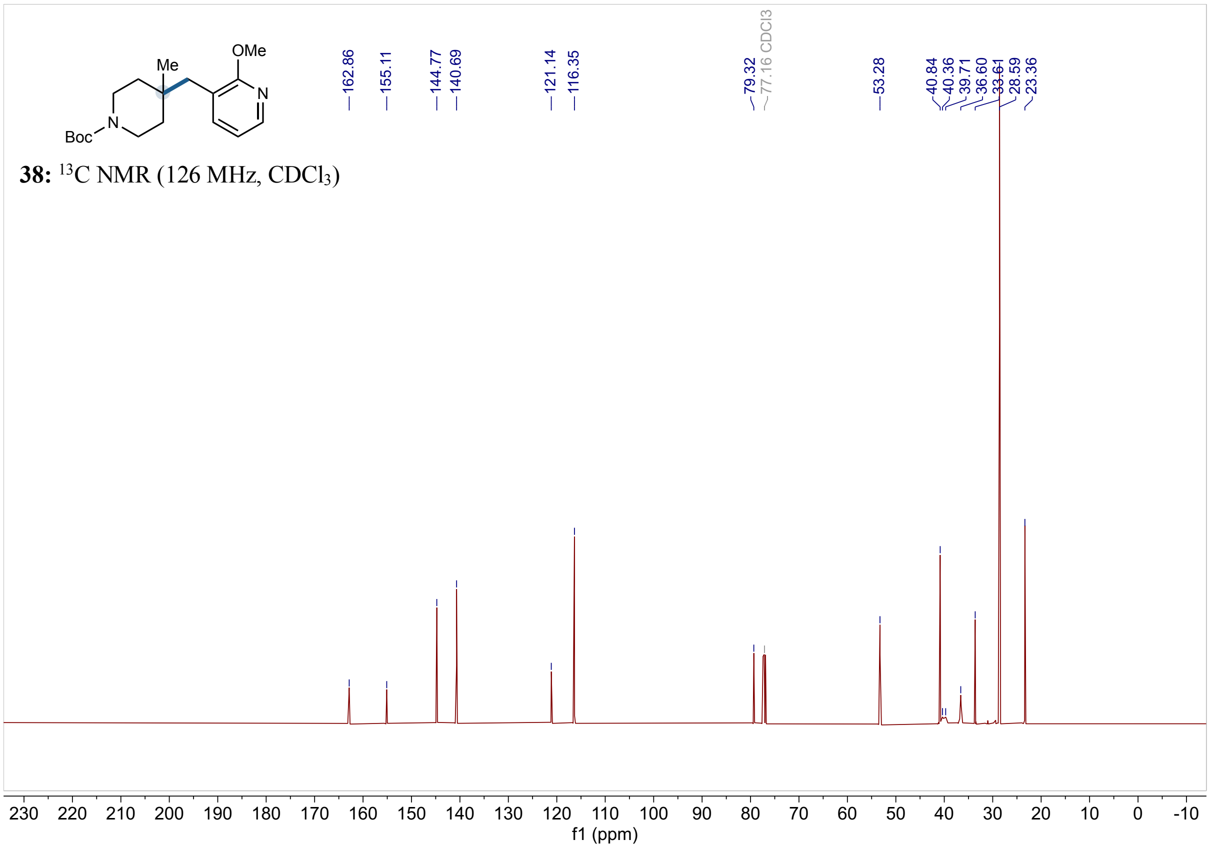

38: ^{13}C NMR (126 MHz, $CDCl_3$)

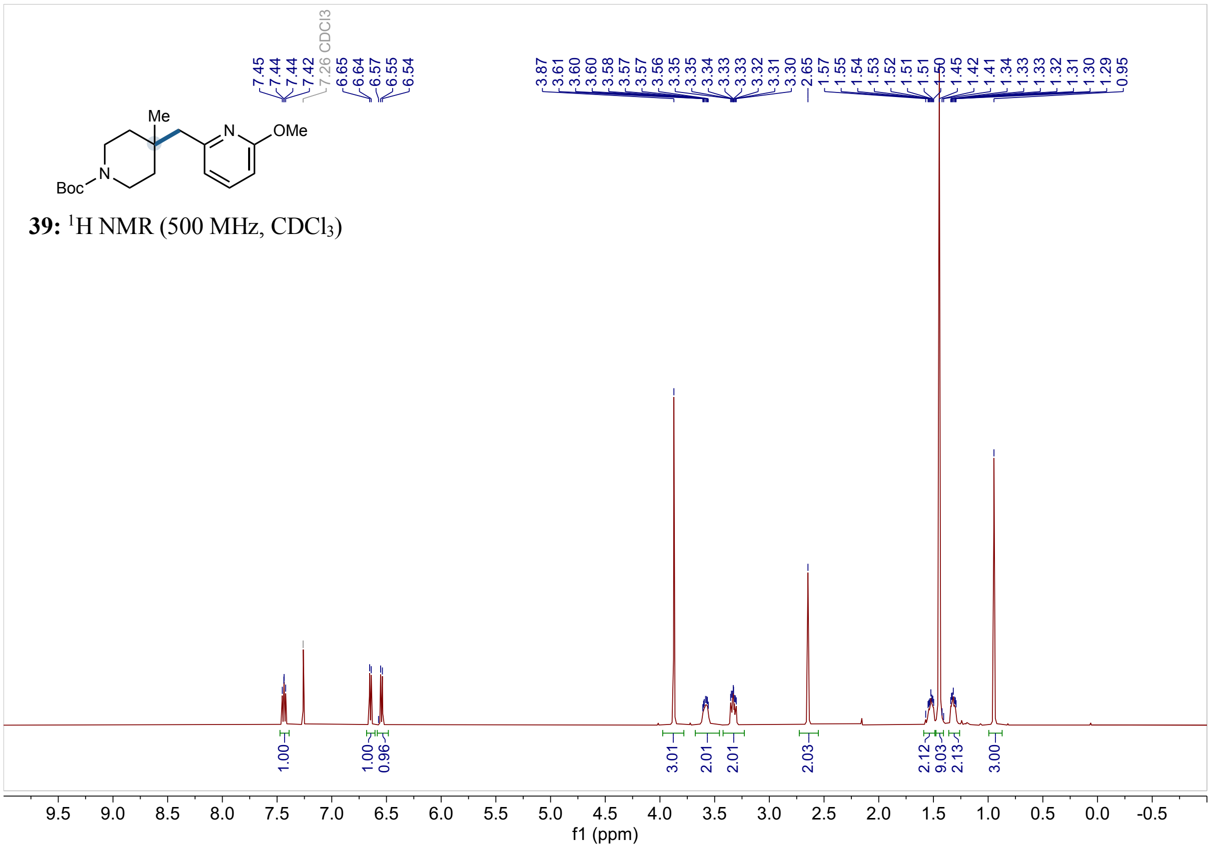

39: ^{1}H NMR (500 MHz, $CDCl_3$)

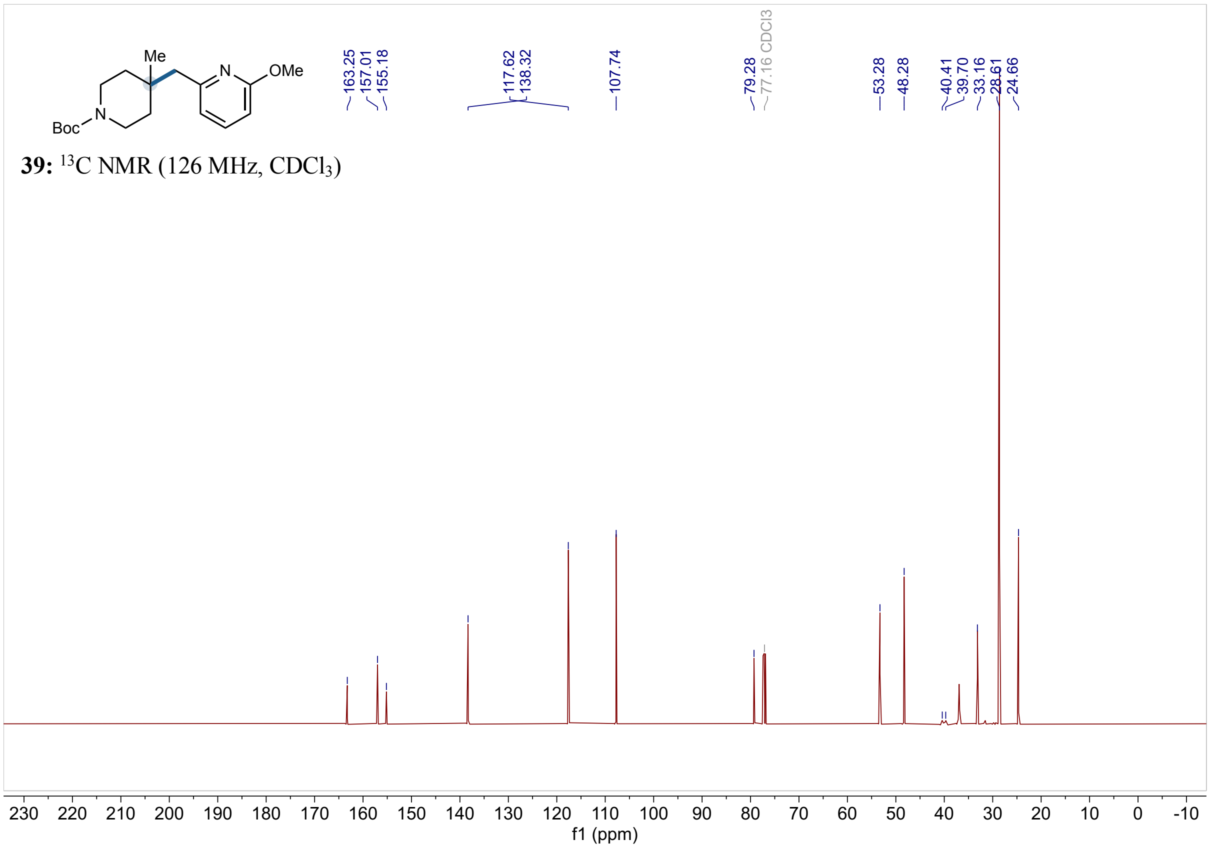

39: ^{13}C NMR (126 MHz, $CDCl_3$)

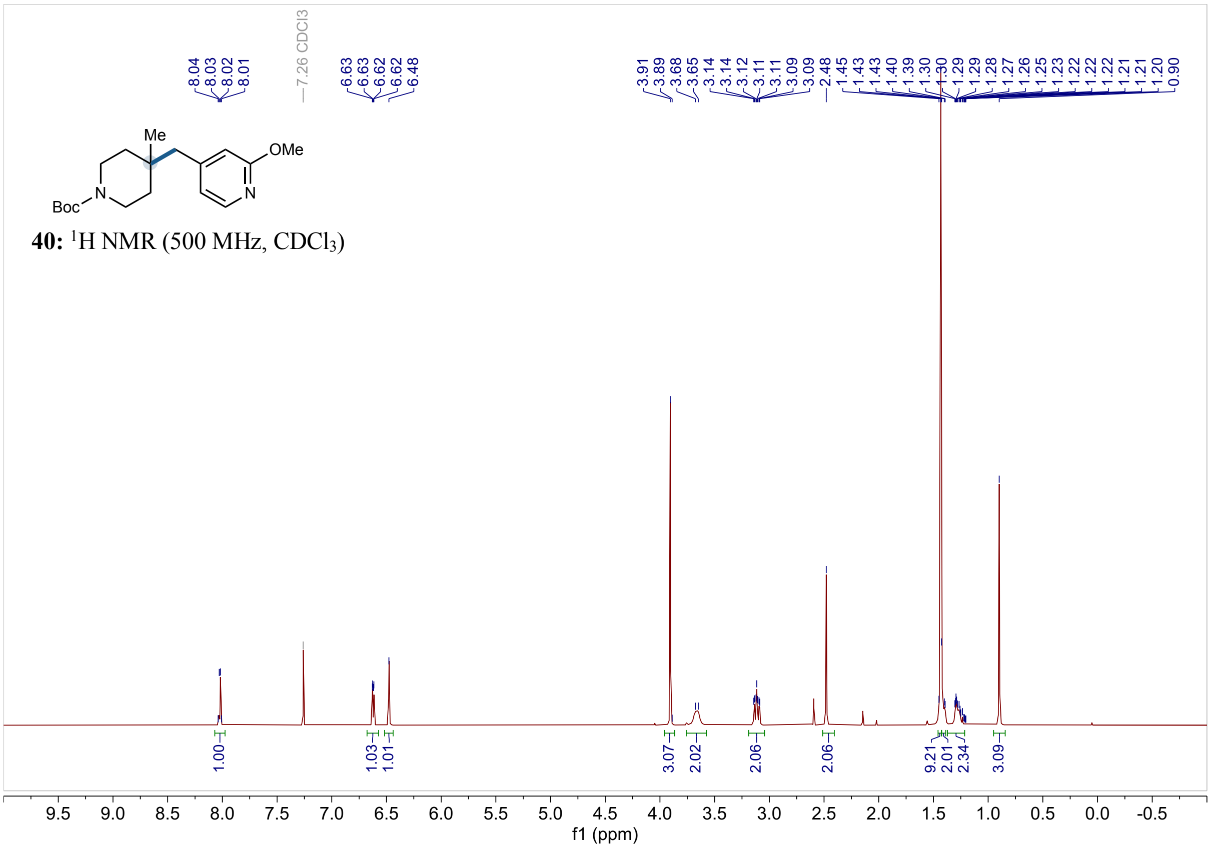
Me
OMe
Boc
N
N
40: ¹H NMR (500 MHz, CDCl3)
8.04
8.03
8.02
8.01
7.26 CDCl3
6.63
6.63
6.62
6.62
6.48
3.91
3.89
3.68
3.65
3.14
3.14
3.12
3.11
3.11
3.09
3.09
2.48
1.45
1.43
1.43
1.40
1.39
1.30
1.30
1.29
1.29
1.28
1.27
1.26
1.25
1.23
1.22
1.22
1.22
1.21
1.21
1.20
0.90
1.00
1.03
1.01
3.07
2.02
2.06
2.06
9.21
2.01
2.34
3.09
f1 (ppm)

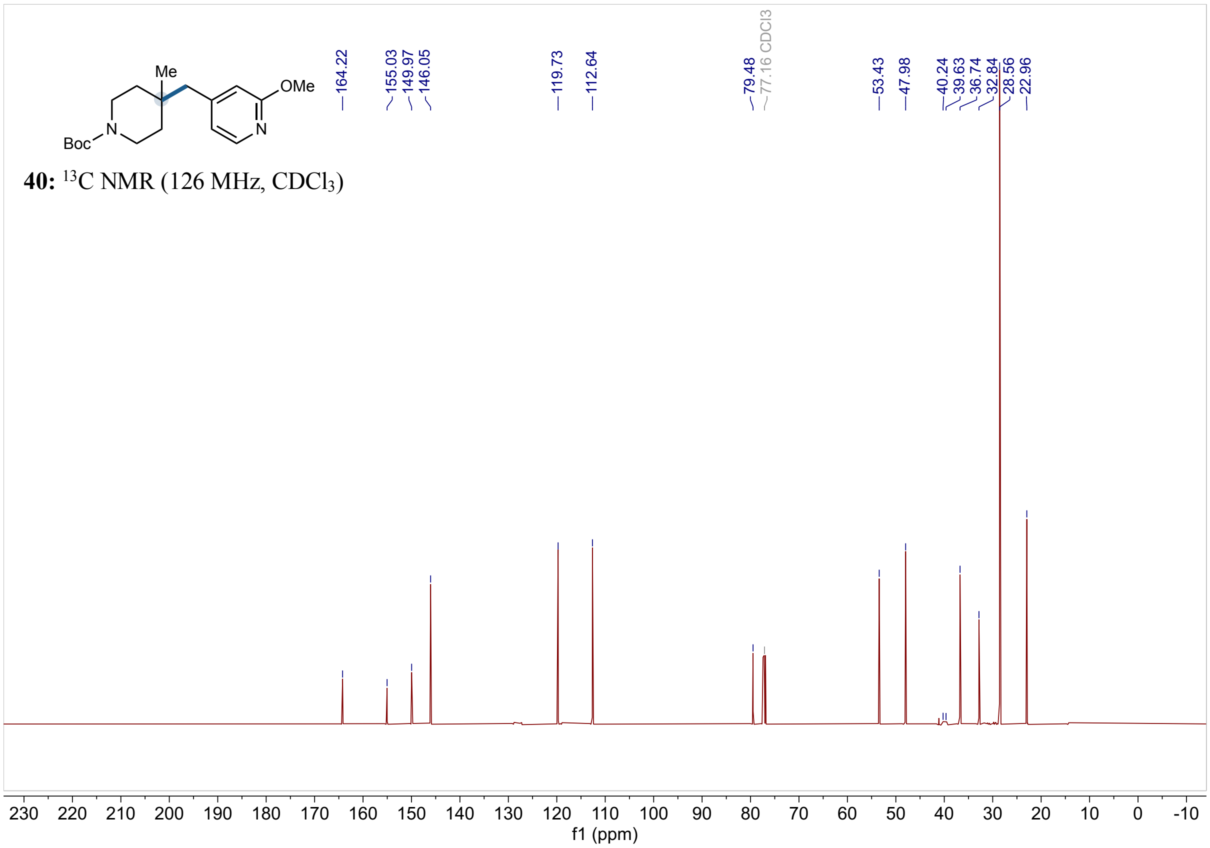
Me
OMe
Boc
N
N
40: ¹³C NMR (126 MHz, CDCl3)
164.22
155.03
149.97
146.05
119.73
112.64
79.48
77.16 CDCl3
53.43
47.98
40.24
39.63
36.74
32.84
28.56
22.96
f1 (ppm)

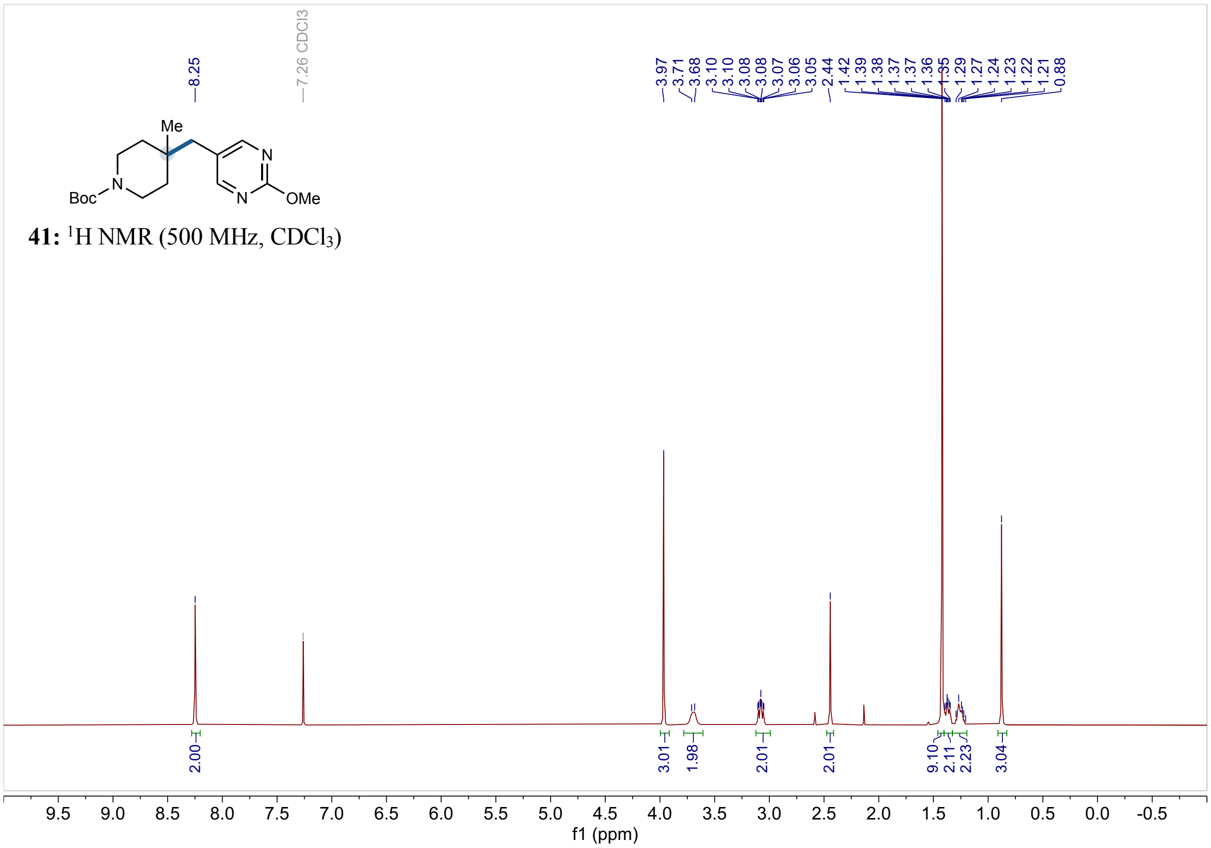

41: ^{1}H NMR (500 MHz, $CDCl_3$)

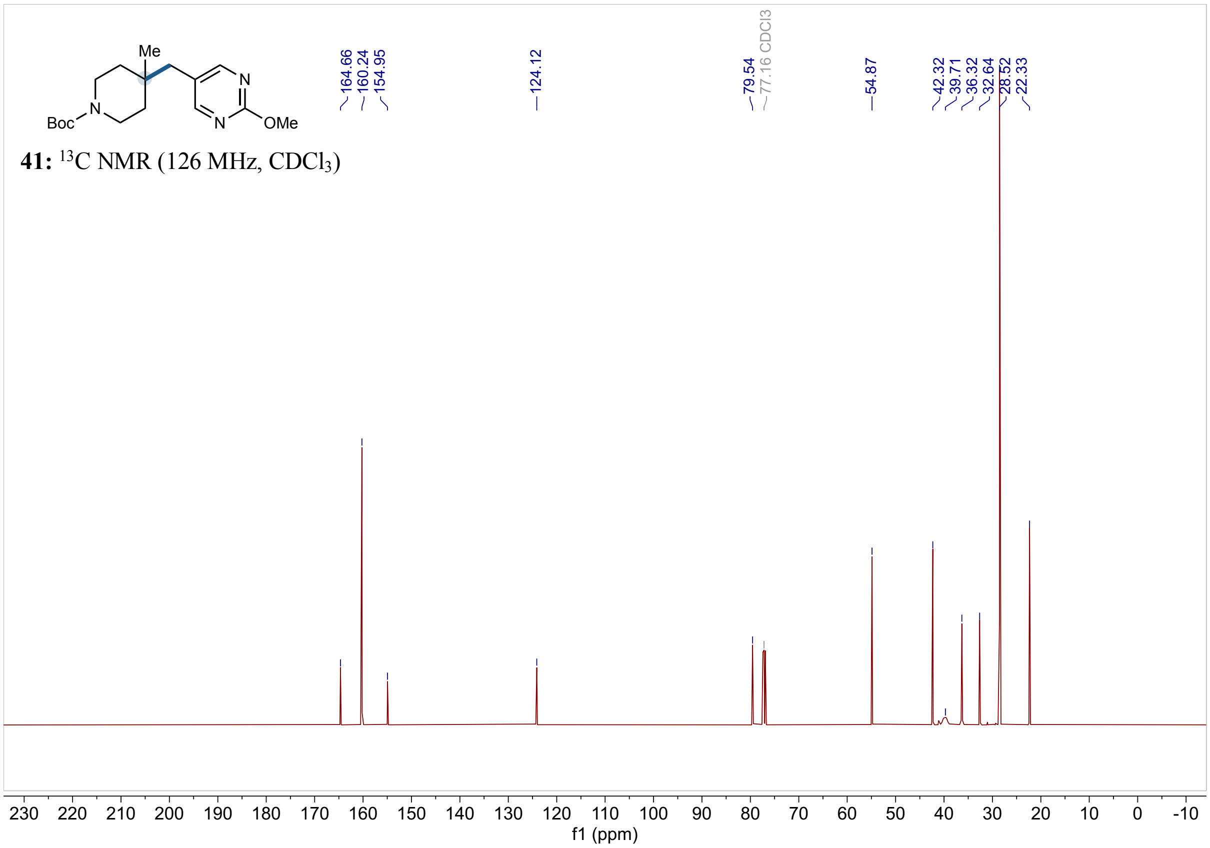

41: ^{13}C NMR (126 MHz, $CDCl_3$)

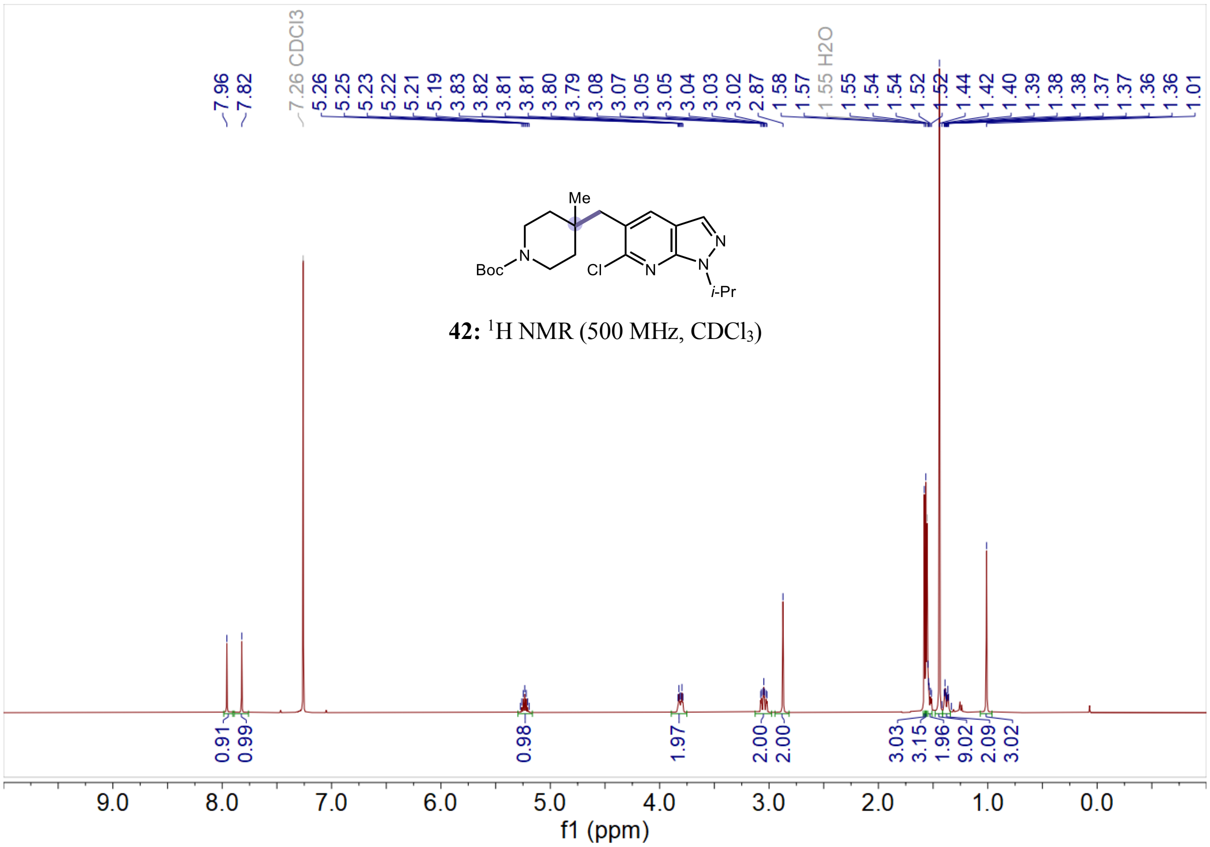

42: ^{1}H NMR (500 MHz, CDCl$_3$)

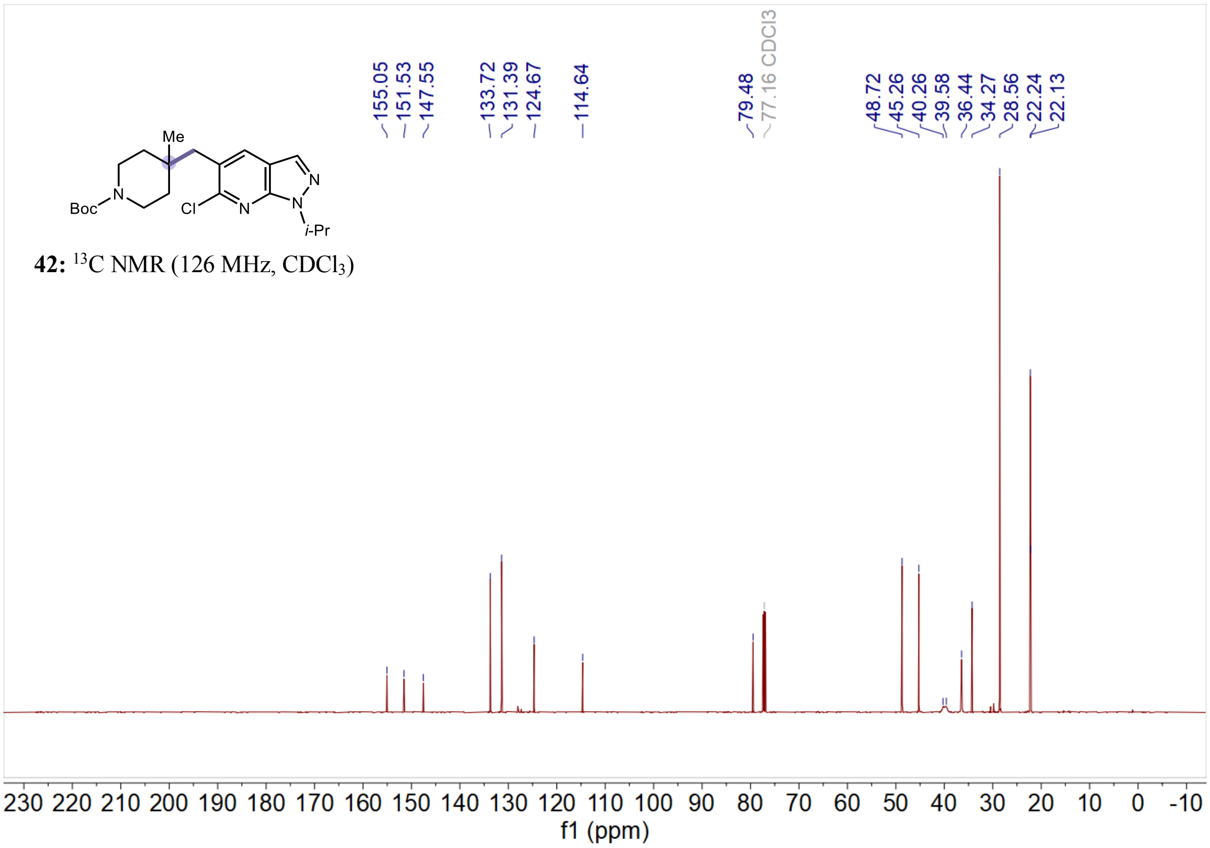

42: ^{13}C NMR (126 MHz, CDCl$_3$)

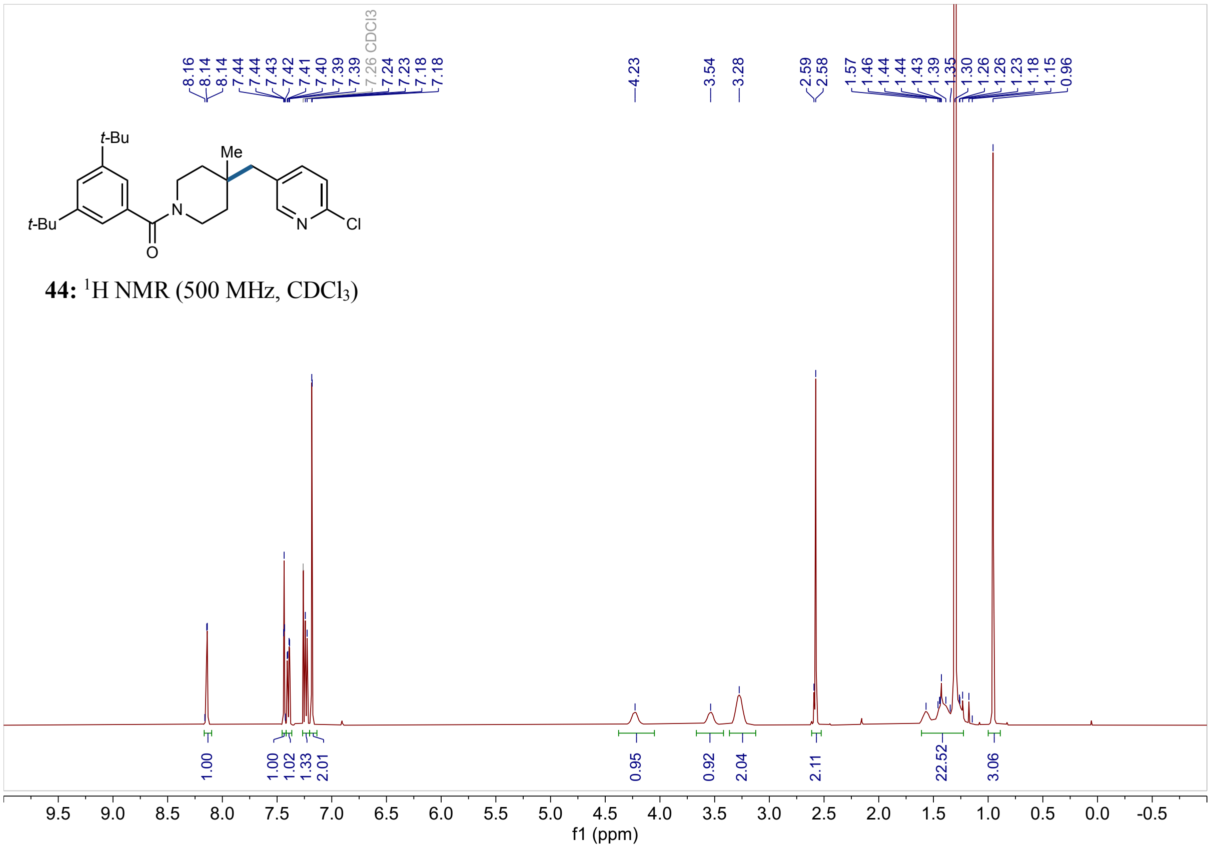

44: ^{1}H NMR (500 MHz, $CDCl_3$)

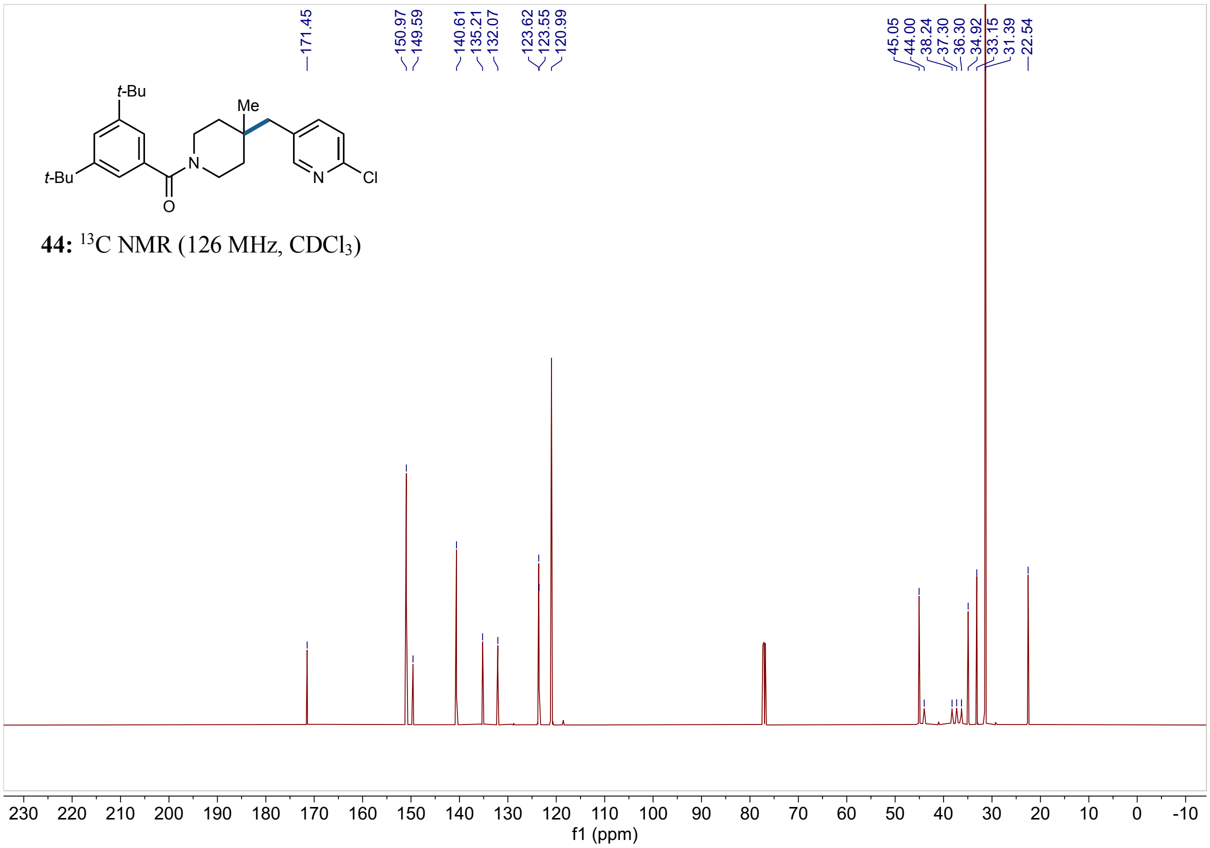

44: ^{13}C NMR (126 MHz, $CDCl_3$)

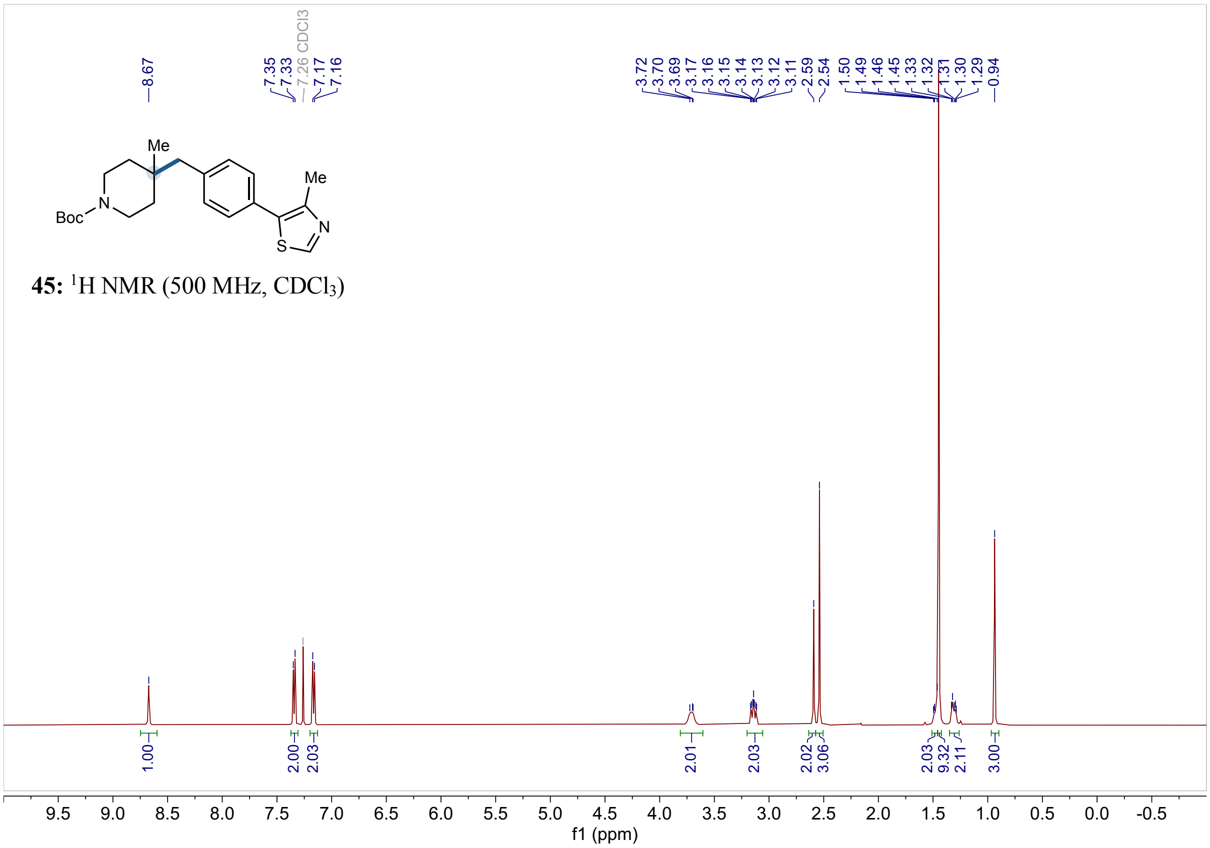

45: ^{1}H NMR (500 MHz, CDCl$_3$)
8.67
7.35
7.33
7.26 CDCl3
7.17
7.16
3.72
3.70
3.69
3.17
3.16
3.15
3.14
3.13
3.12
3.11
2.59
2.54
1.50
1.49
1.46
1.45
1.33
1.32
1.31
1.30
1.29
0.94
1.00
2.00
2.03
2.01
2.03
2.02
3.06
2.03
9.32
2.11
3.00
f1 (ppm)

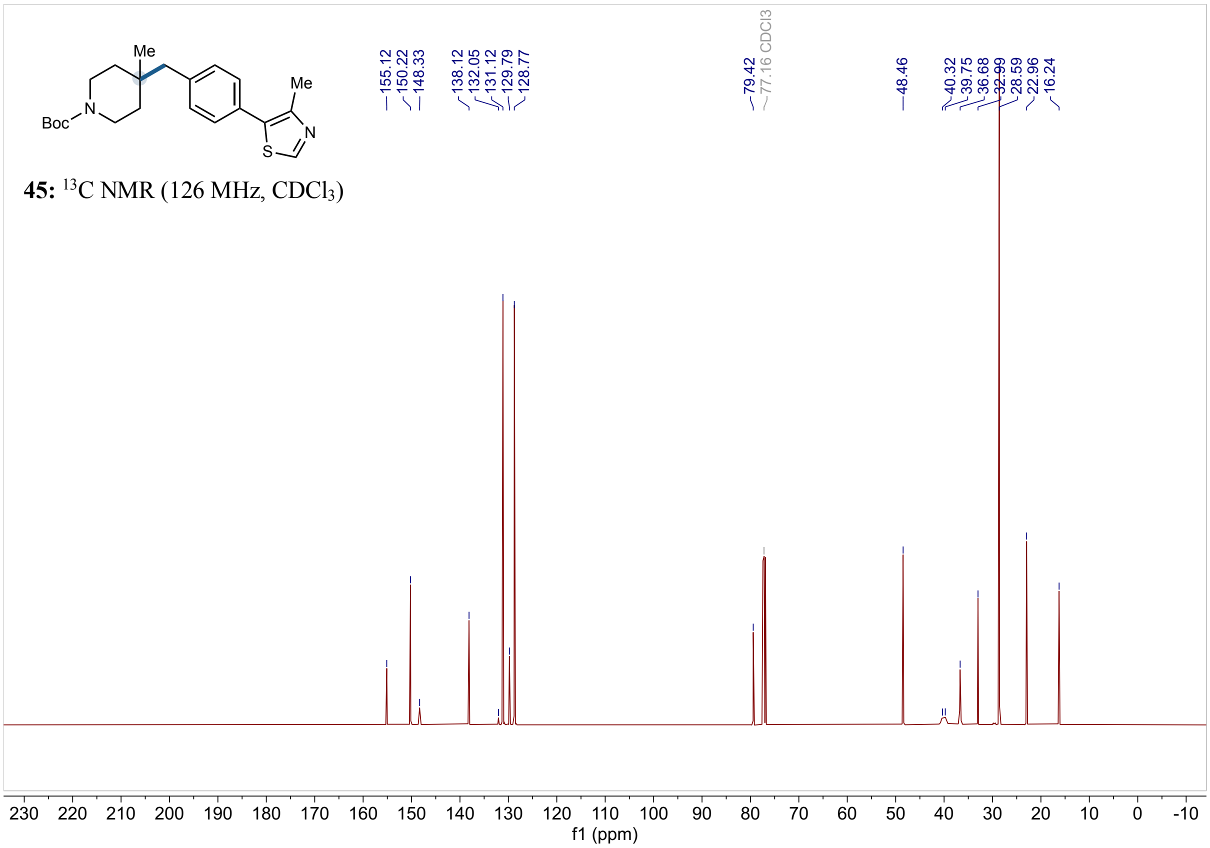

45: ^{13}C NMR (126 MHz, CDCl$_3$)
155.12
150.22
148.33
138.12
132.05
131.12
129.79
128.77
79.42
77.16 CDCl3
48.46
40.32
39.75
36.68
32.99
28.59
22.96
16.24
f1 (ppm)

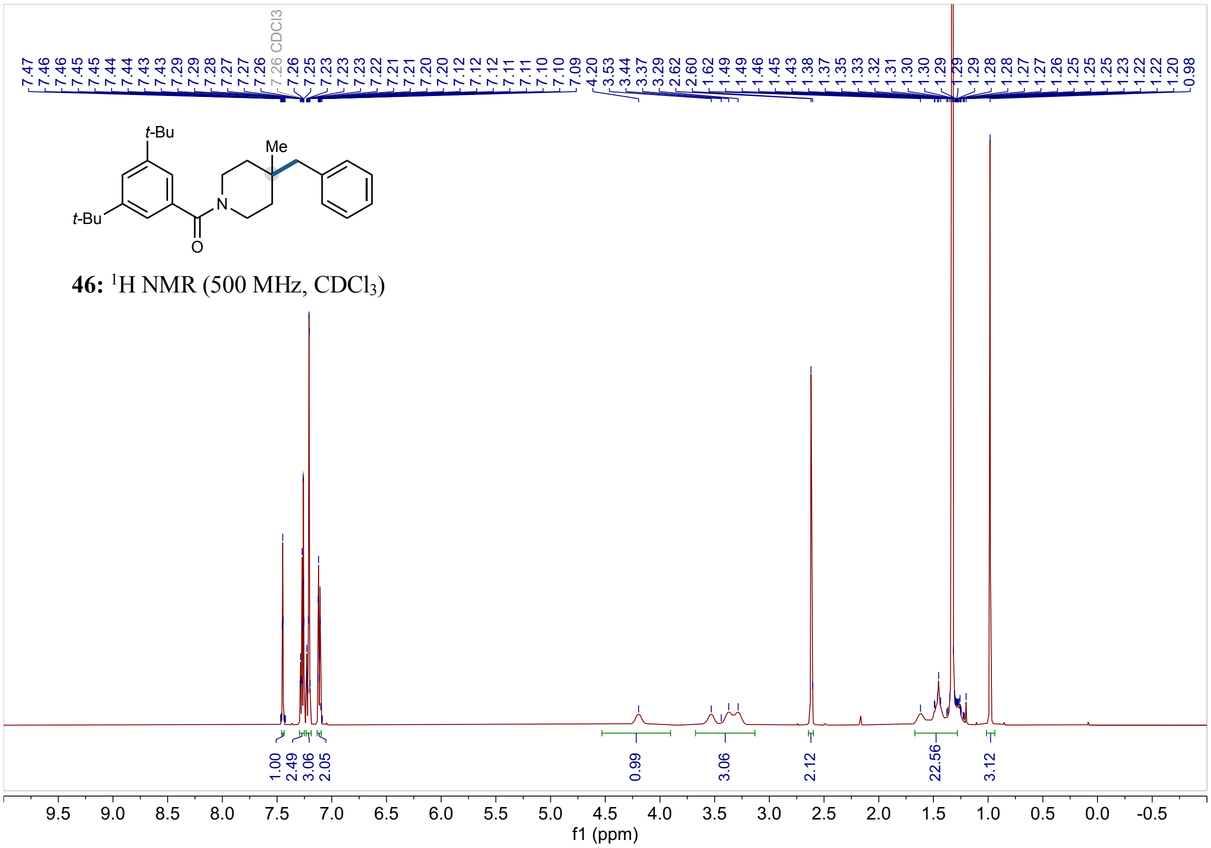
t-Bu
Me
t-Bu
N
O
46: ¹H NMR (500 MHz, CDCl₃)
f1 (ppm)

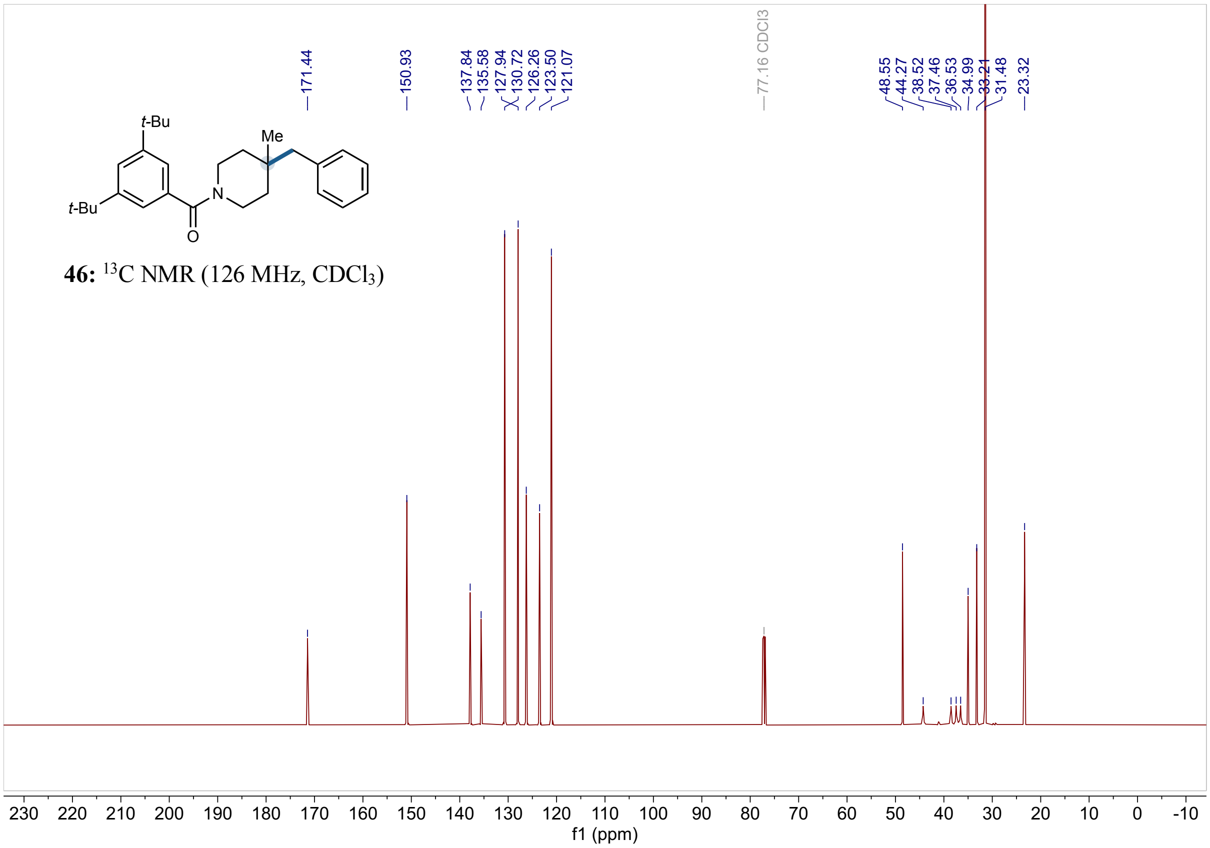
t-Bu
Me
t-Bu
N
O
46: ¹³C NMR (126 MHz, CDCl₃)
171.44
150.93
137.84
135.58
127.94
130.72
126.26
123.50
121.07
77.16 CDCl3
48.55
44.27
38.52
37.46
36.53
34.99
33.21
31.48
23.32
f1 (ppm)

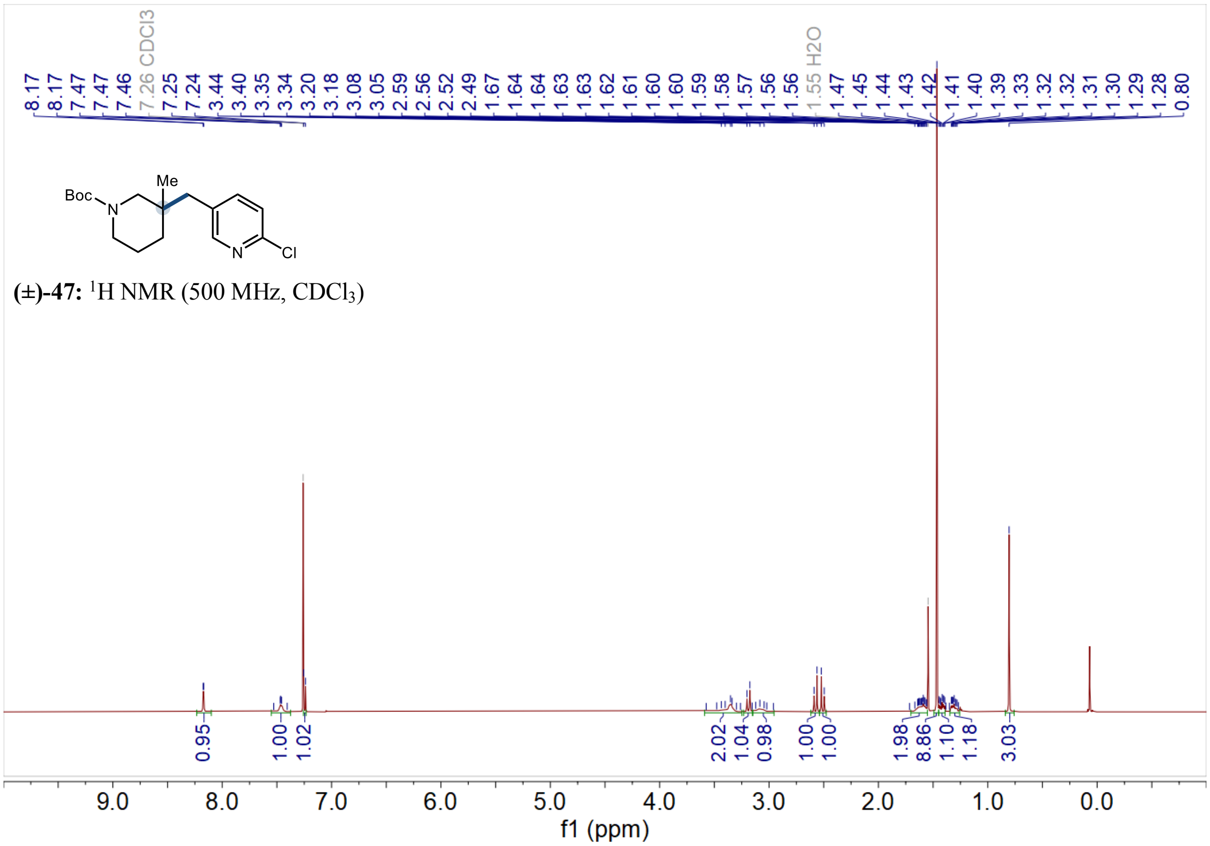

(±)-47: ^{1}H NMR (500 MHz, $CDCl_3$)

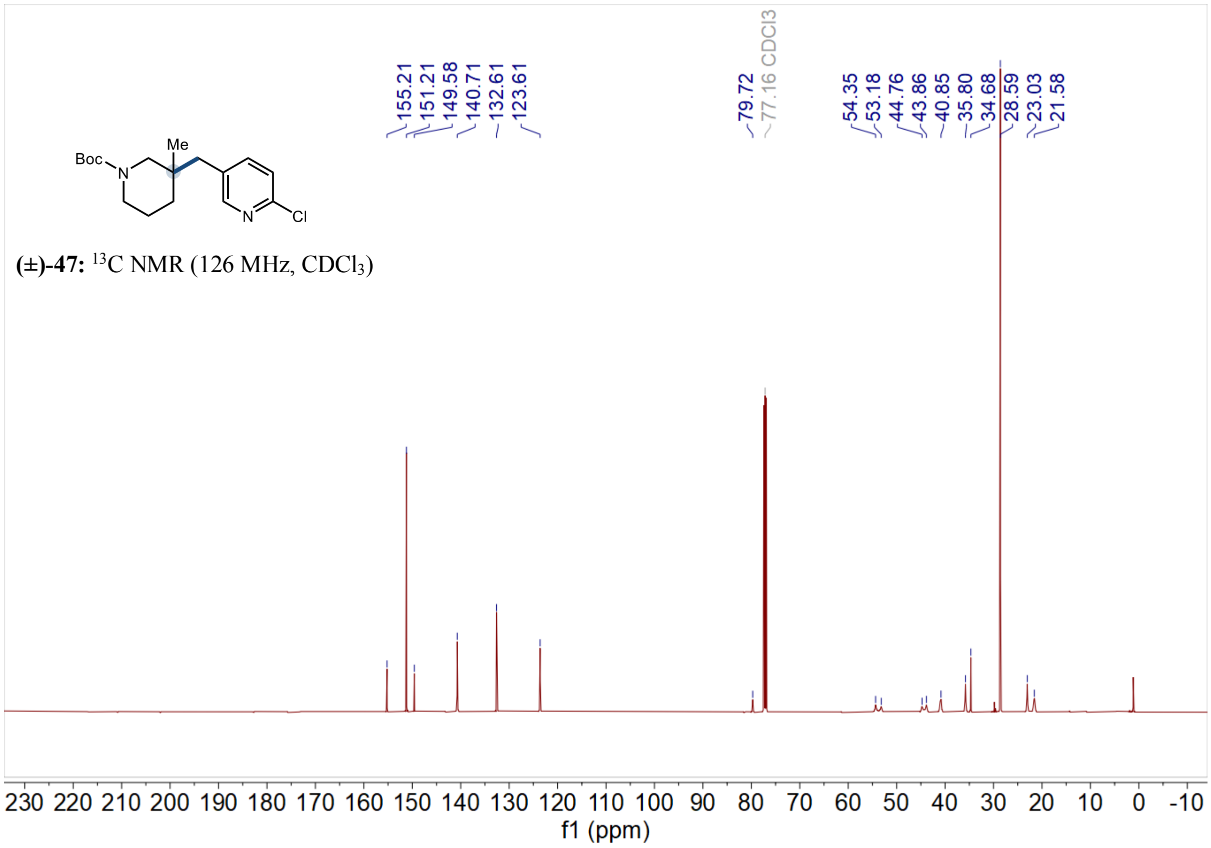

(±)-47: ^{13}C NMR (126 MHz, $CDCl_3$)

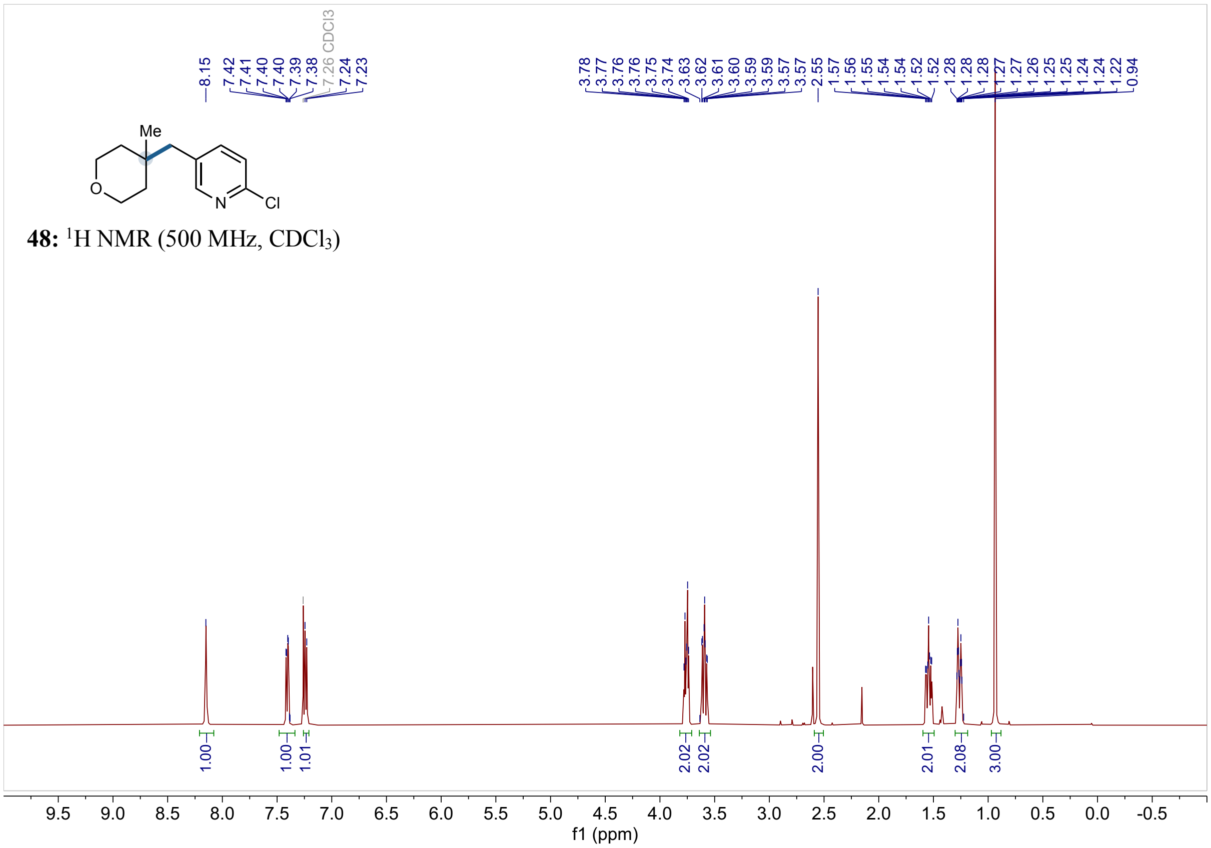

48: ^{1}H NMR (500 MHz, $CDCl_3$)

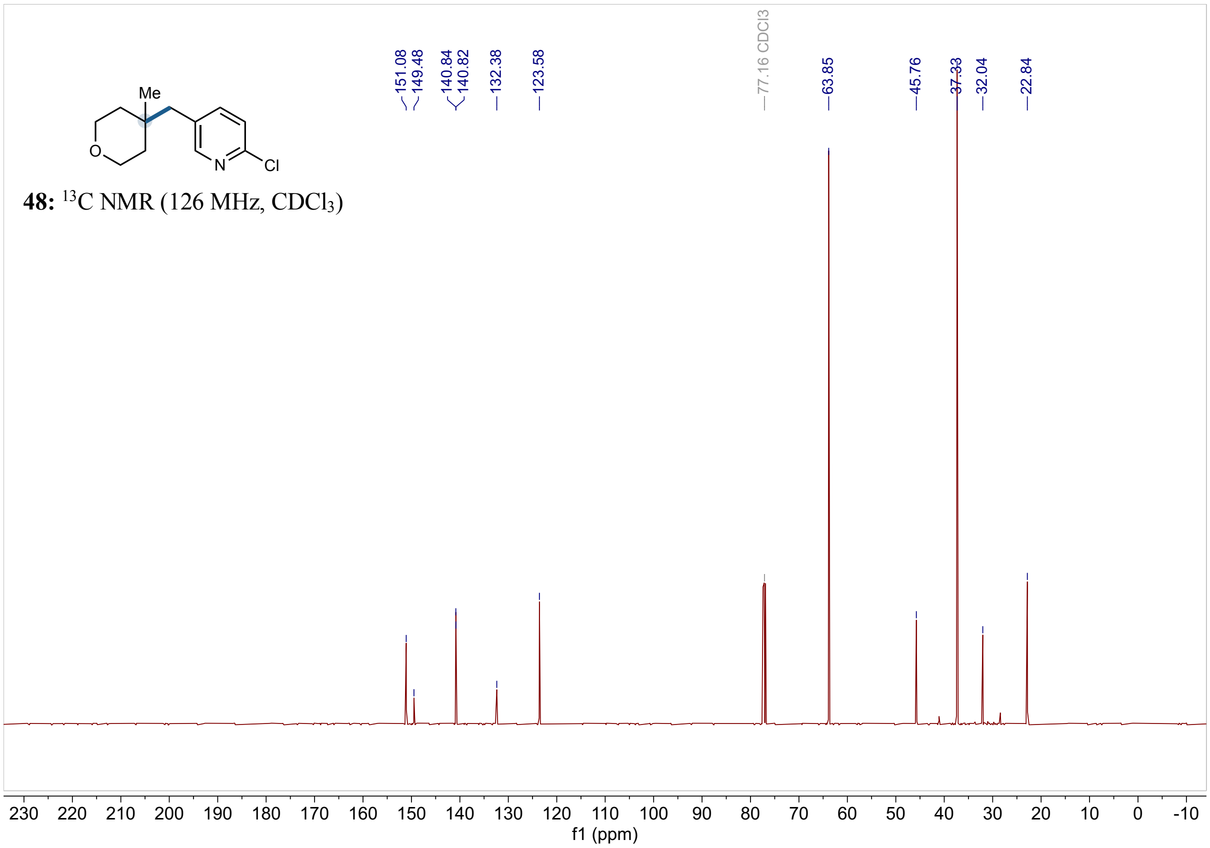

48: ^{13}C NMR (126 MHz, $CDCl_3$)

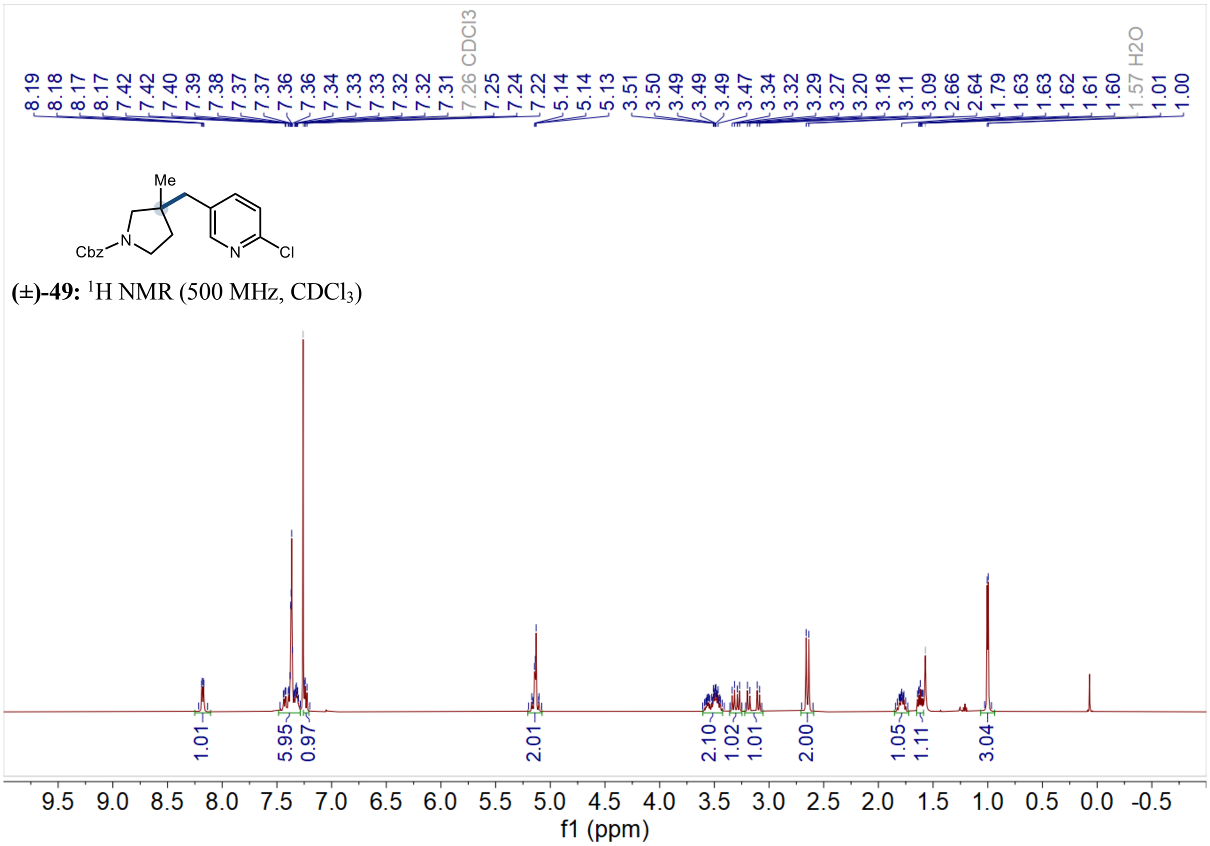

(±)-49: ^{1}H NMR (500 MHz, $CDCl_3$)

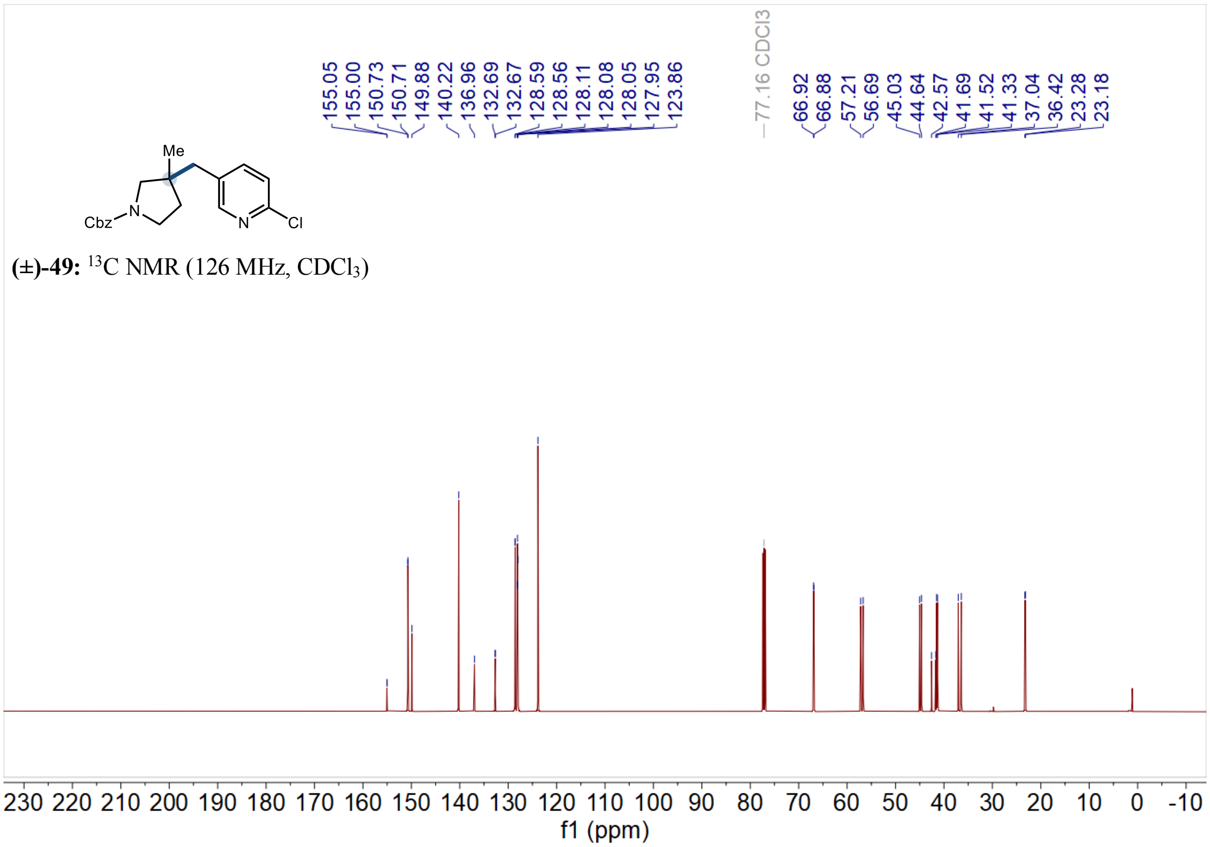

(±)-49: ^{13}C NMR (126 MHz, $CDCl_3$)

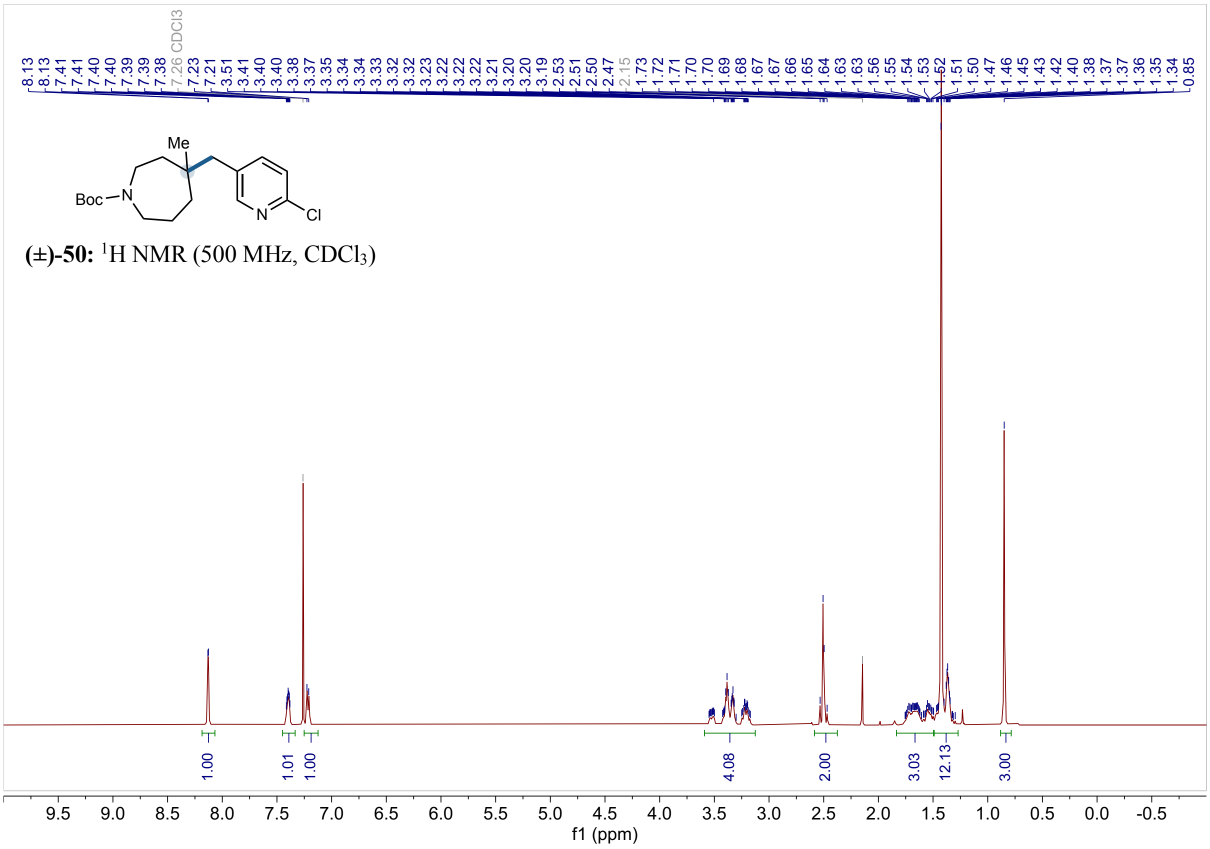
(±)-50: 1H NMR (500 MHz, CDCl3)
Me
Boc
N
Cl
8.13 8.13 7.41 7.40 7.40 7.39 7.39 7.38 7.26 CDCl3 7.23 7.21 3.51 3.41 3.40 3.38 3.37 3.35 3.34 3.34 3.33 3.32 3.32 3.23 3.22 3.22 3.22 3.21 3.20 3.19 2.53 2.51 2.50 2.47 2.15 1.73 1.72 1.71 1.70 1.69 1.68 1.67 1.67 1.66 1.65 1.64 1.63 1.63 1.56 1.55 1.54 1.53 1.52 1.51 1.50 1.47 1.46 1.45 1.43 1.42 1.40 1.38 1.37 1.37 1.36 1.35 1.34 0.85
1.00 1.01 1.00 4.08 2.00 3.03 12.13 3.00
9.5 9.0 8.5 8.0 7.5 7.0 6.5 6.0 5.5 5.0 4.5 4.0 3.5 3.0 2.5 2.0 1.5 1.0 0.5 0.0 -0.5
f1 (ppm)

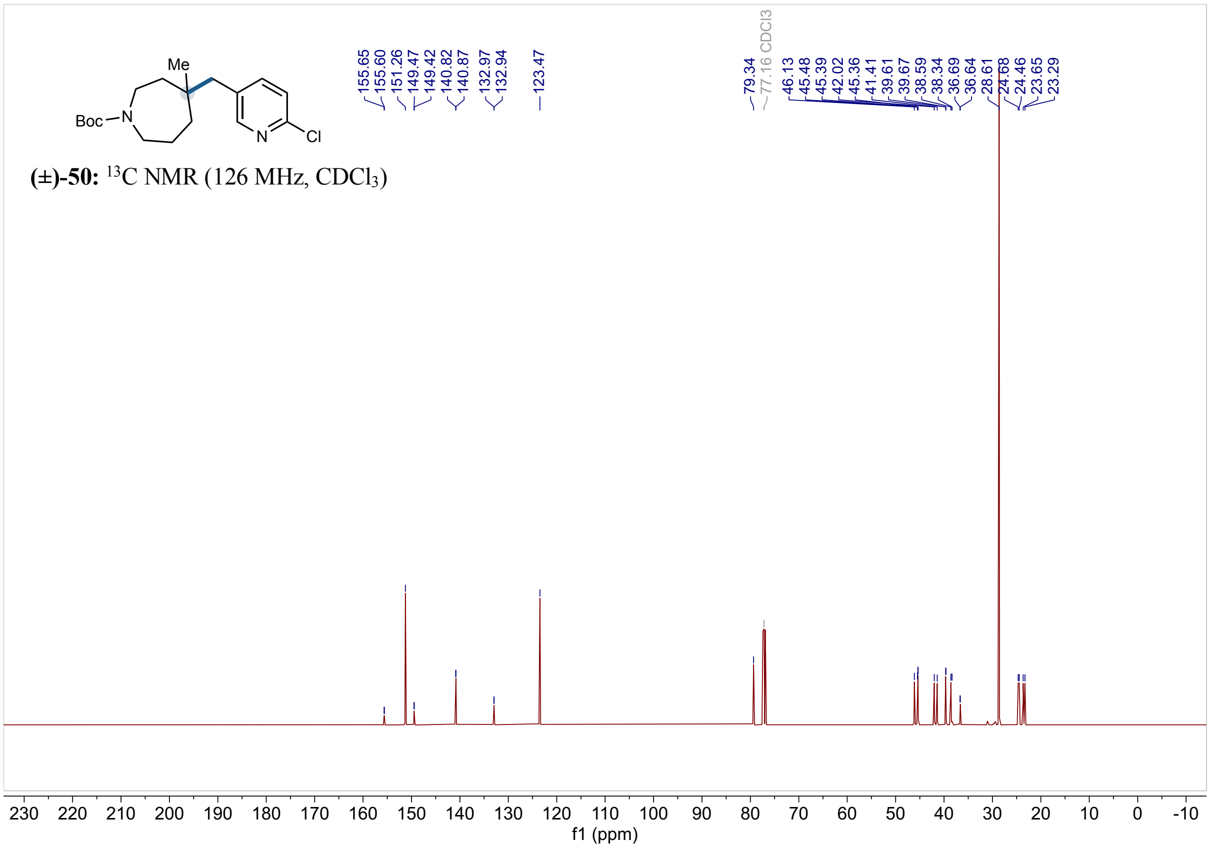
(±)-50: 13C NMR (126 MHz, CDCl3)
Me
Boc
N
Cl
155.65 155.60 151.26 149.47 149.42 140.82 140.87 132.97 132.94 123.47
79.34 77.16 CDCl3 46.13 45.48 45.39 42.02 45.36 41.41 39.67 39.61 38.59 38.34 36.69 36.64 28.61 24.68 24.46 23.65 23.29
230 220 210 200 190 180 170 160 150 140 130 120 110 100 90 80 70 60 50 40 30 20 10 0 -10
f1 (ppm)

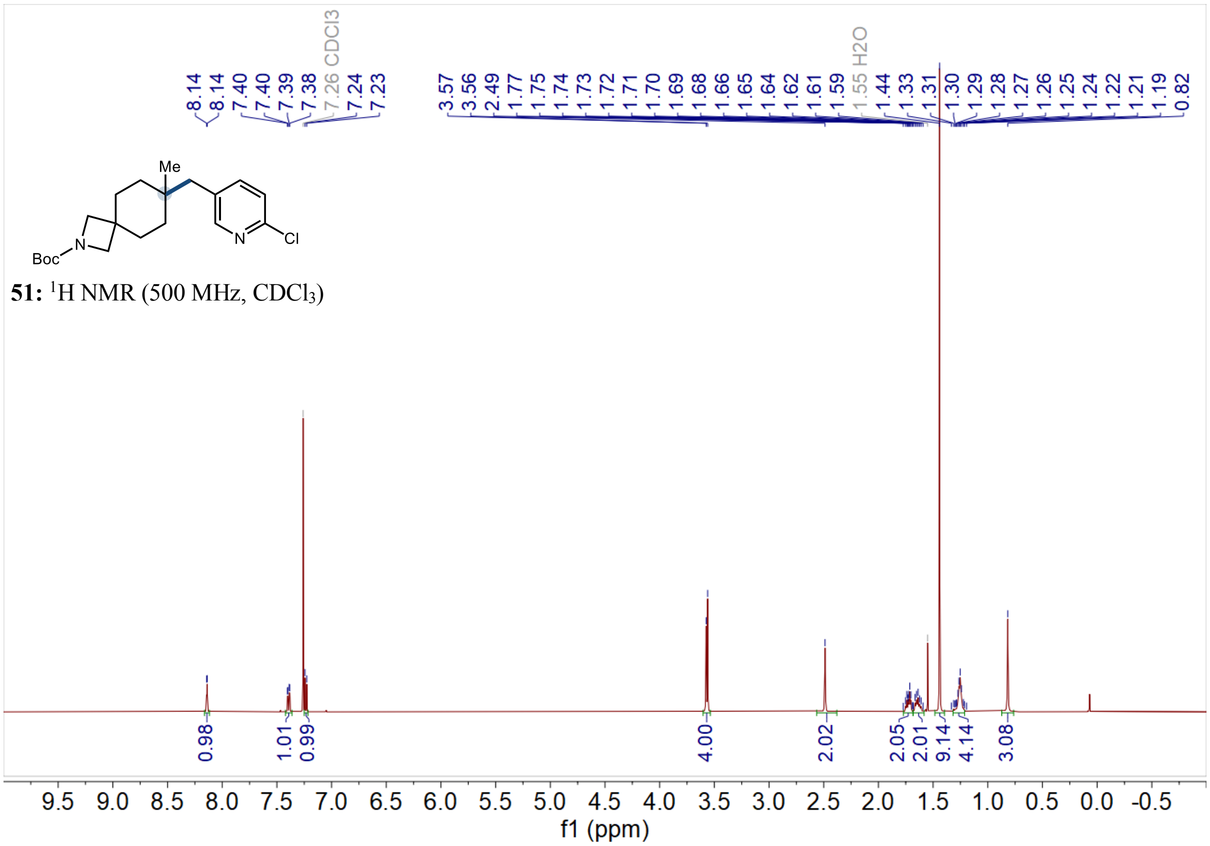

51: ^{1}H NMR (500 MHz, $CDCl_3$)

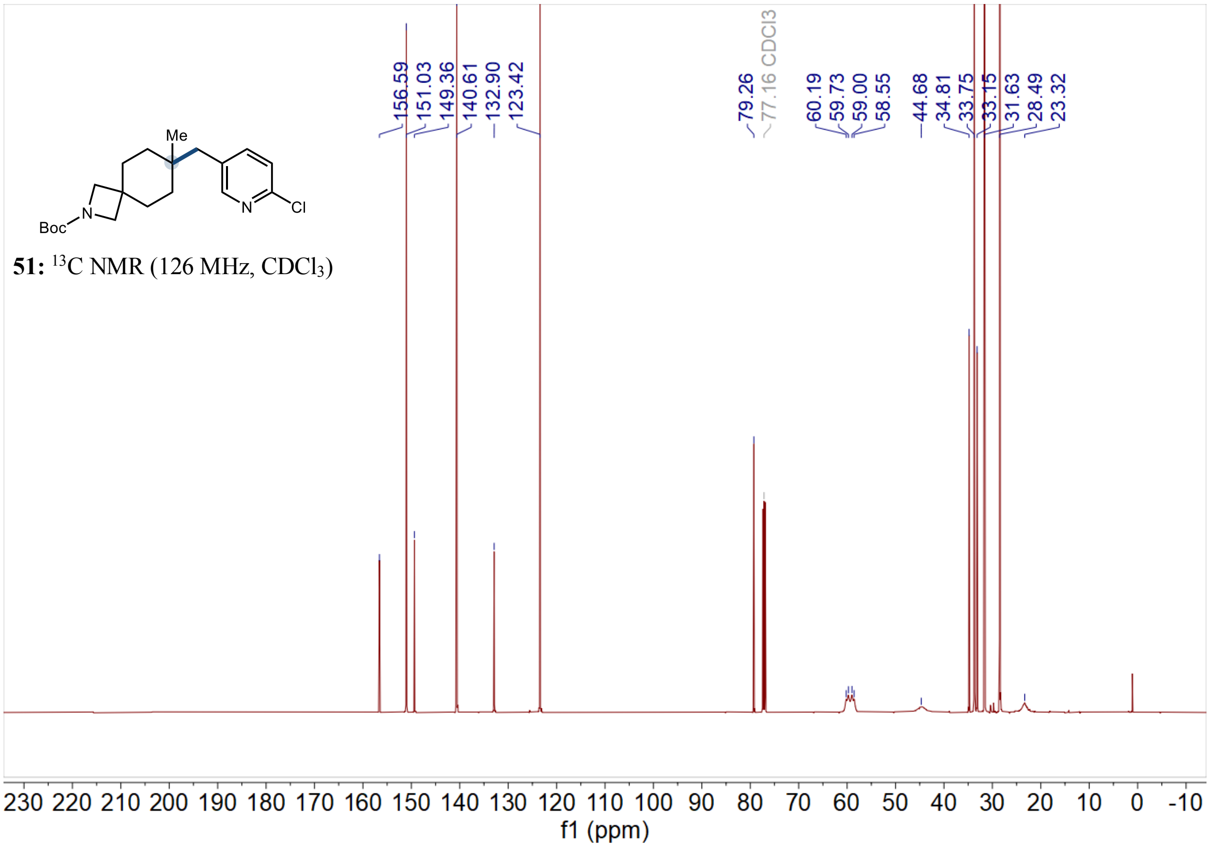

51: ^{13}C NMR (126 MHz, $CDCl_3$)

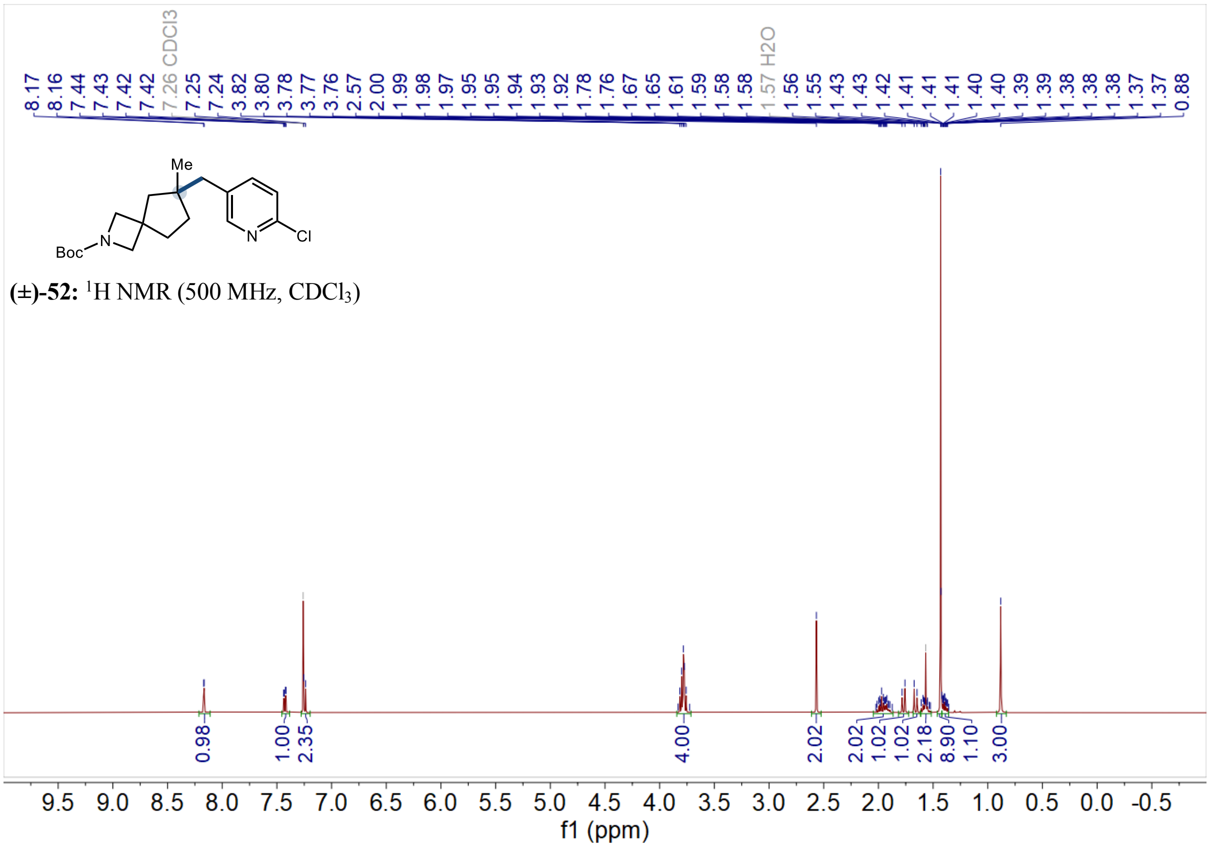

(±)-52: ^{1}H NMR (500 MHz, $CDCl_3$)

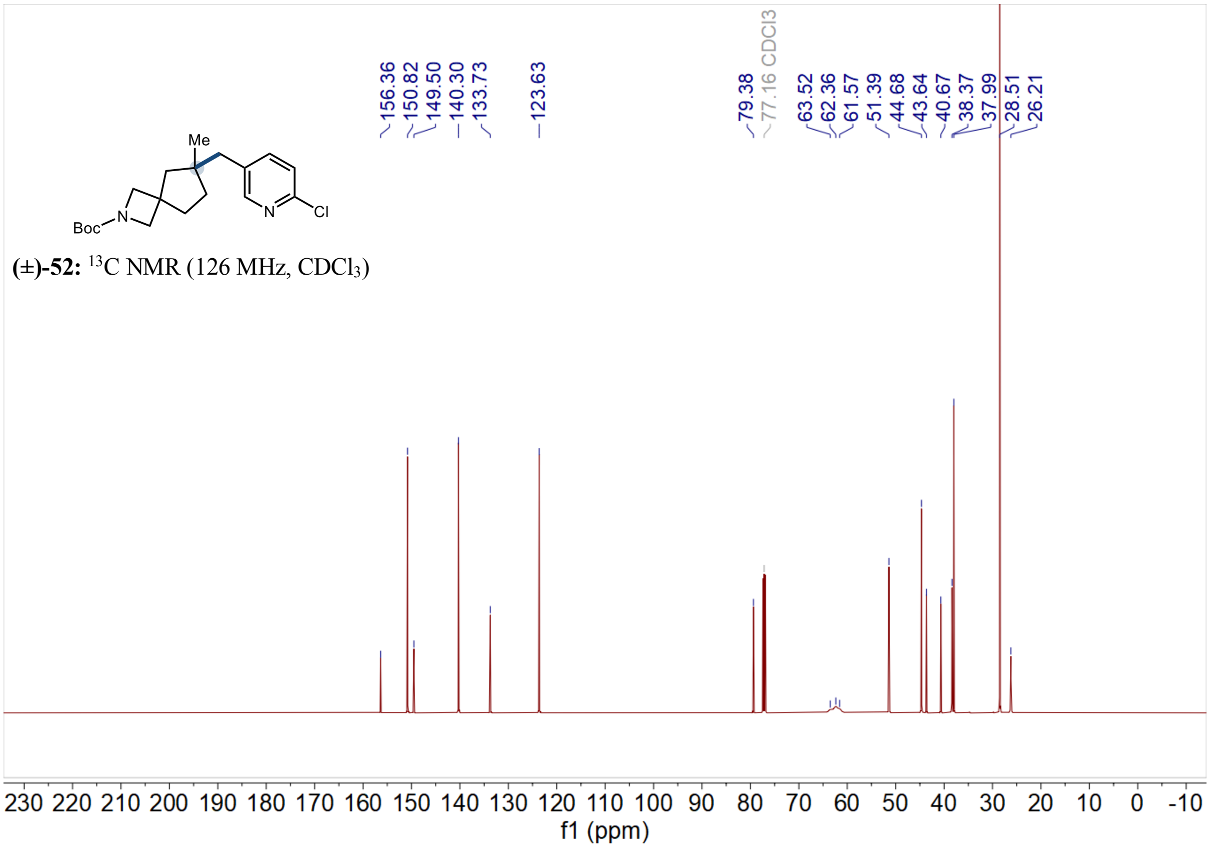

(±)-52: ^{13}C NMR (126 MHz, $CDCl_3$)

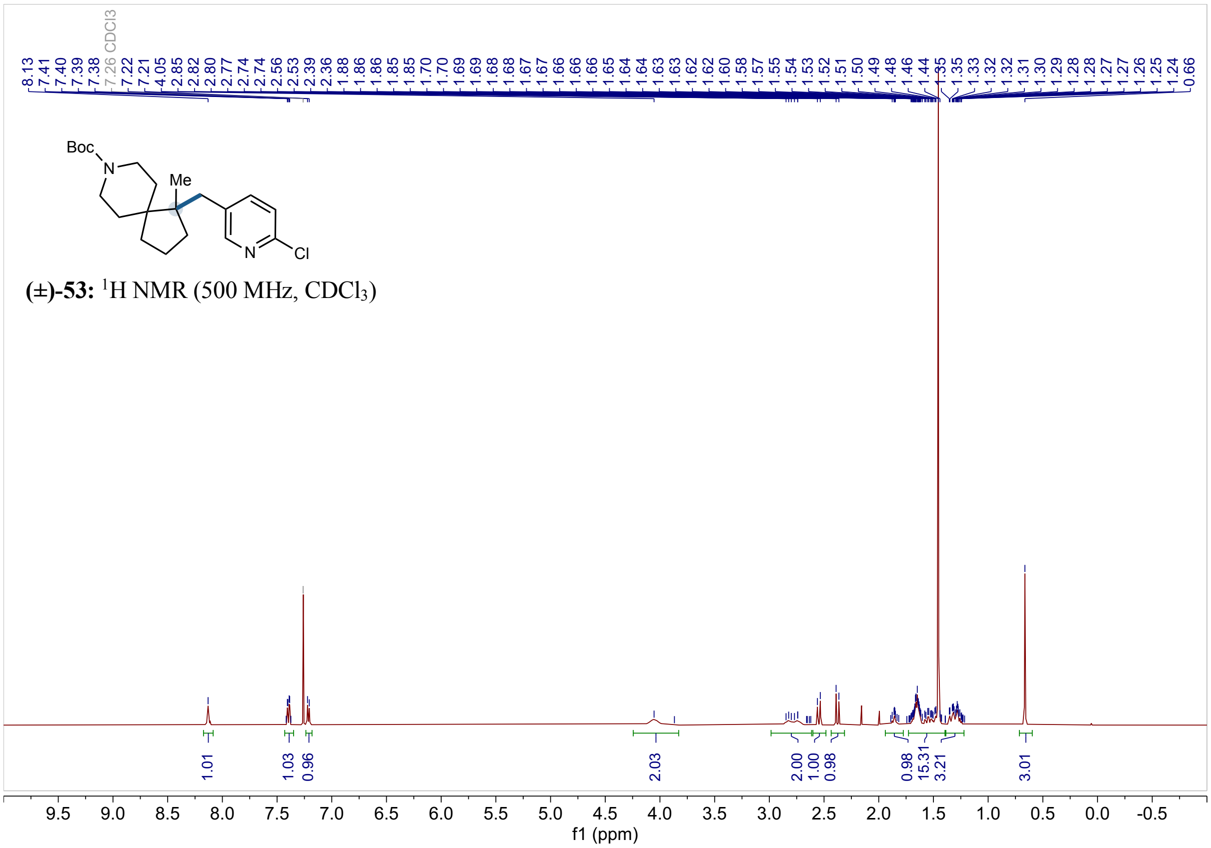

(±)-53: ^{1}H NMR (500 MHz, $CDCl_3$)

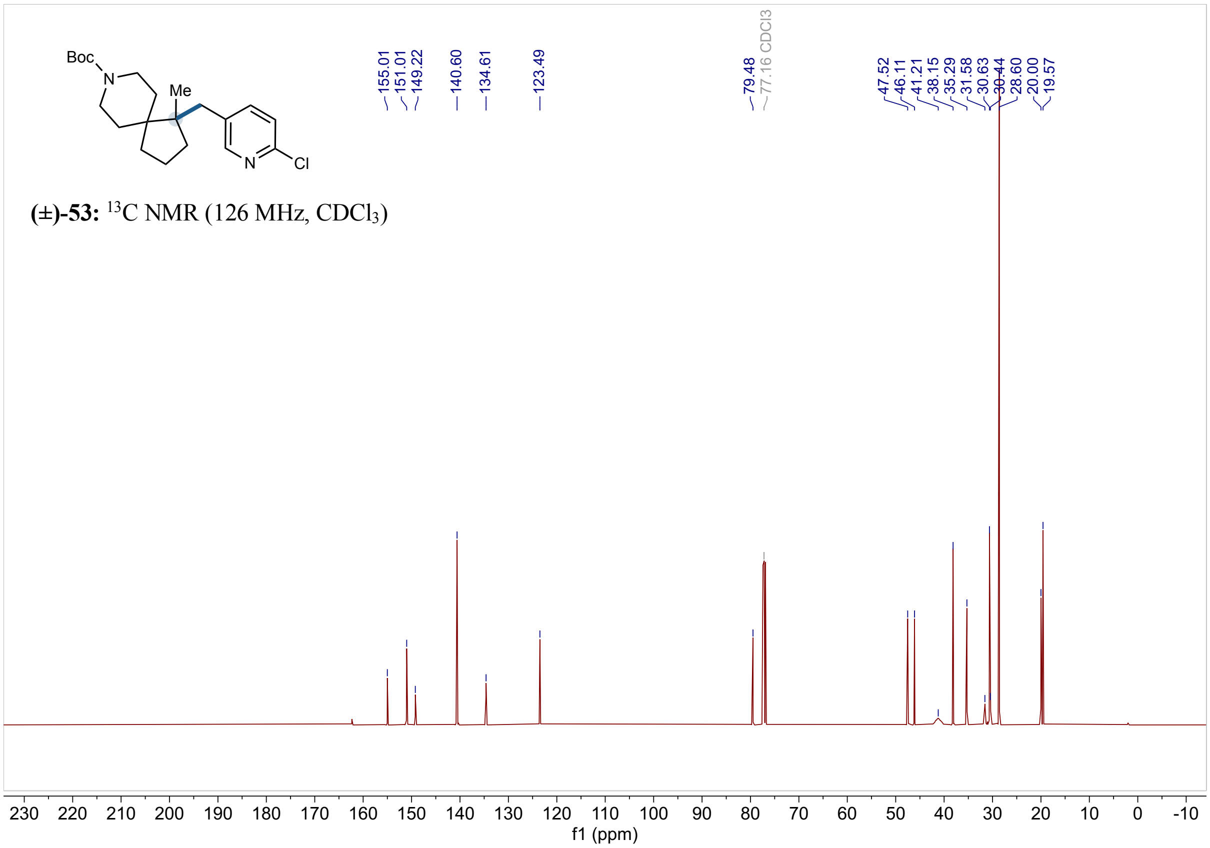

(±)-53: ^{13}C NMR (126 MHz, $CDCl_3$)

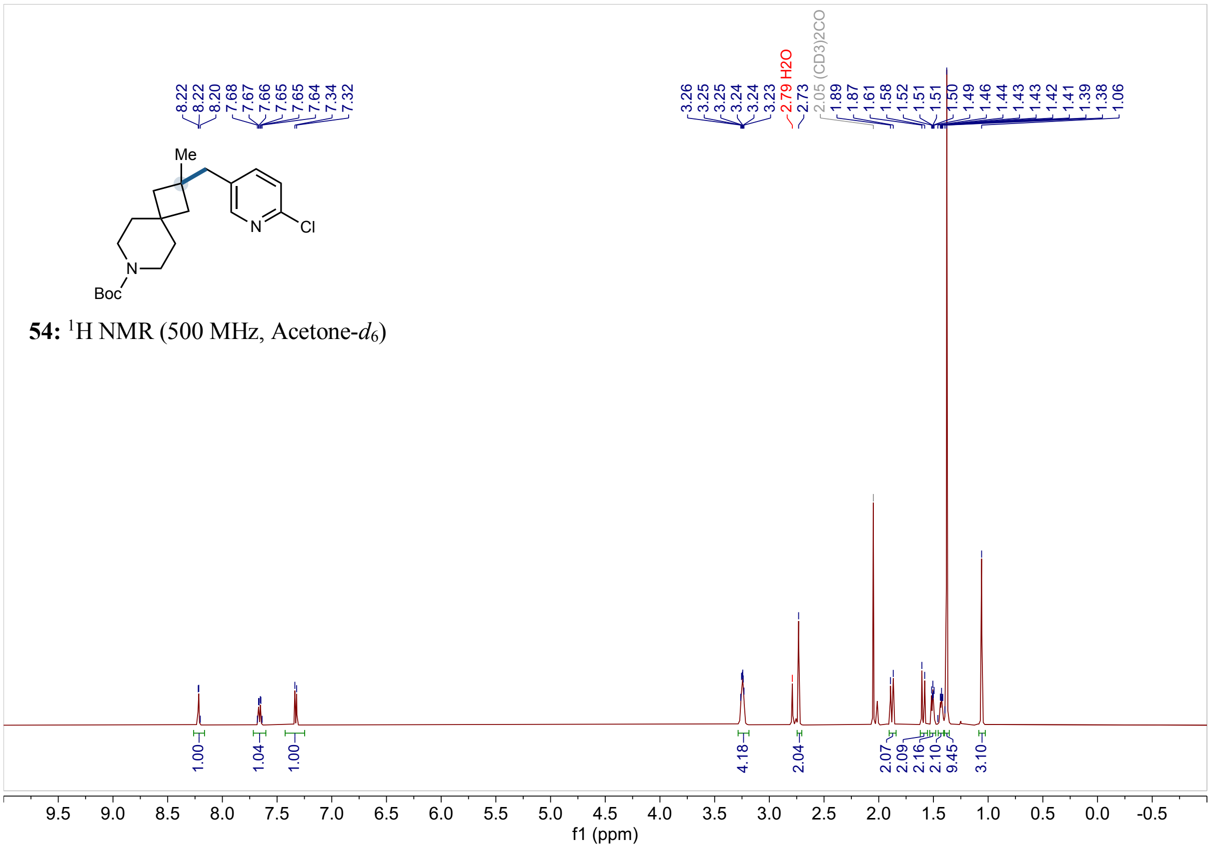

54: ^{1}H NMR (500 MHz, Acetone-d_6)

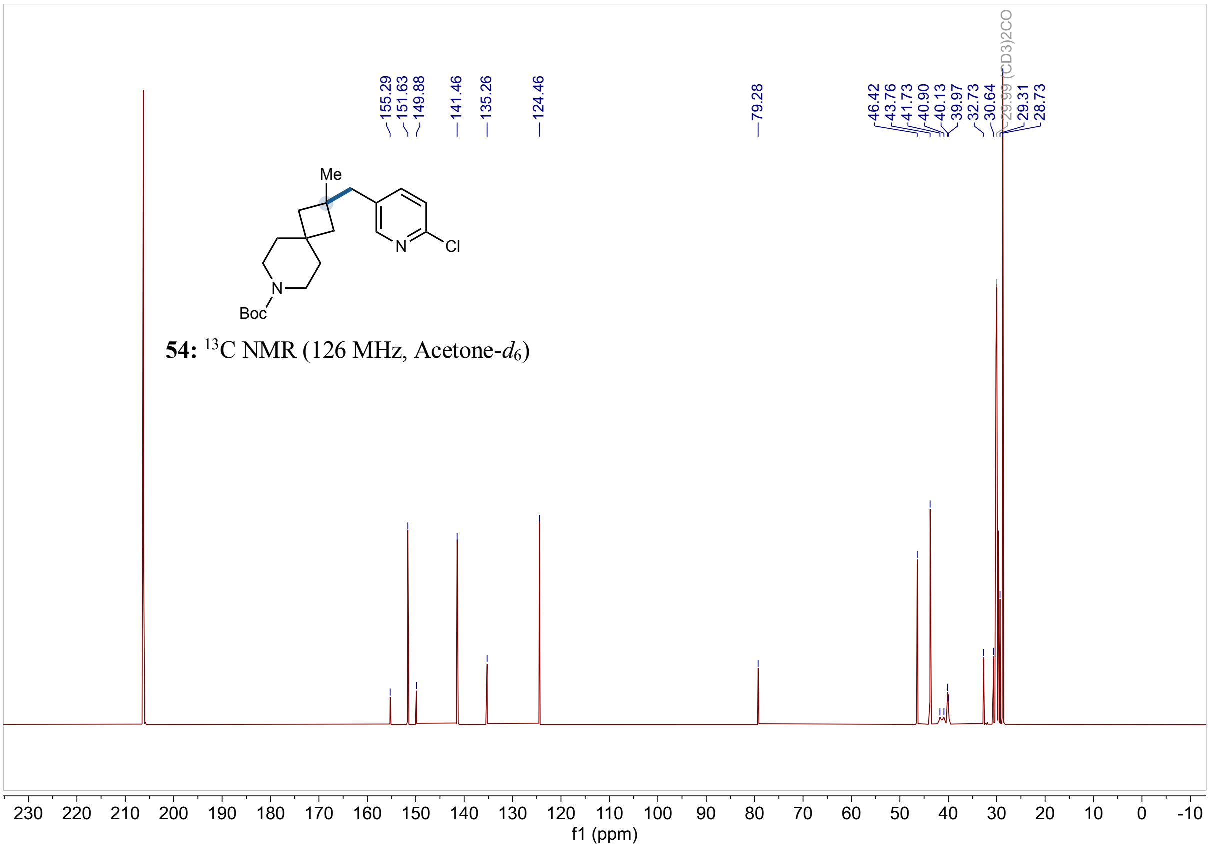

54: ^{13}C NMR (126 MHz, Acetone-d_6)

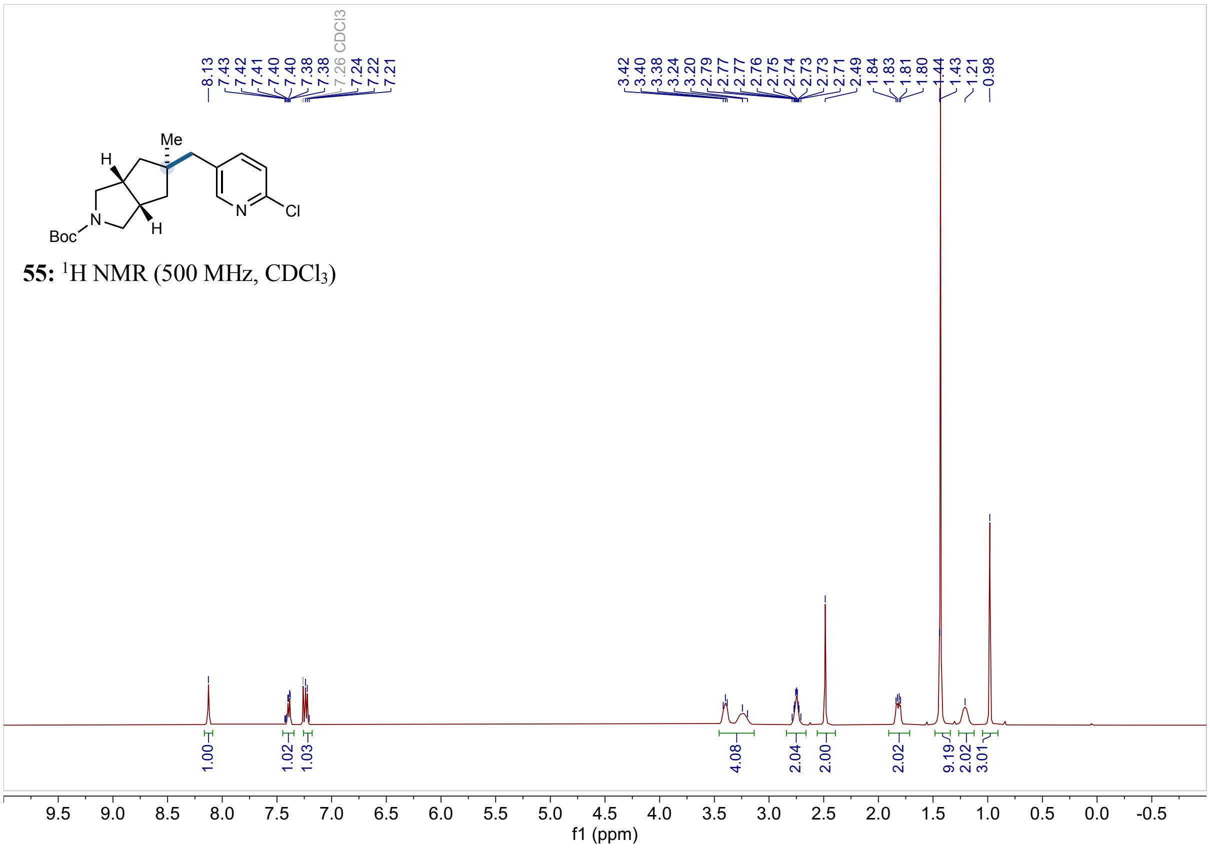

55: ^{1}H NMR (500 MHz, $CDCl_3$)

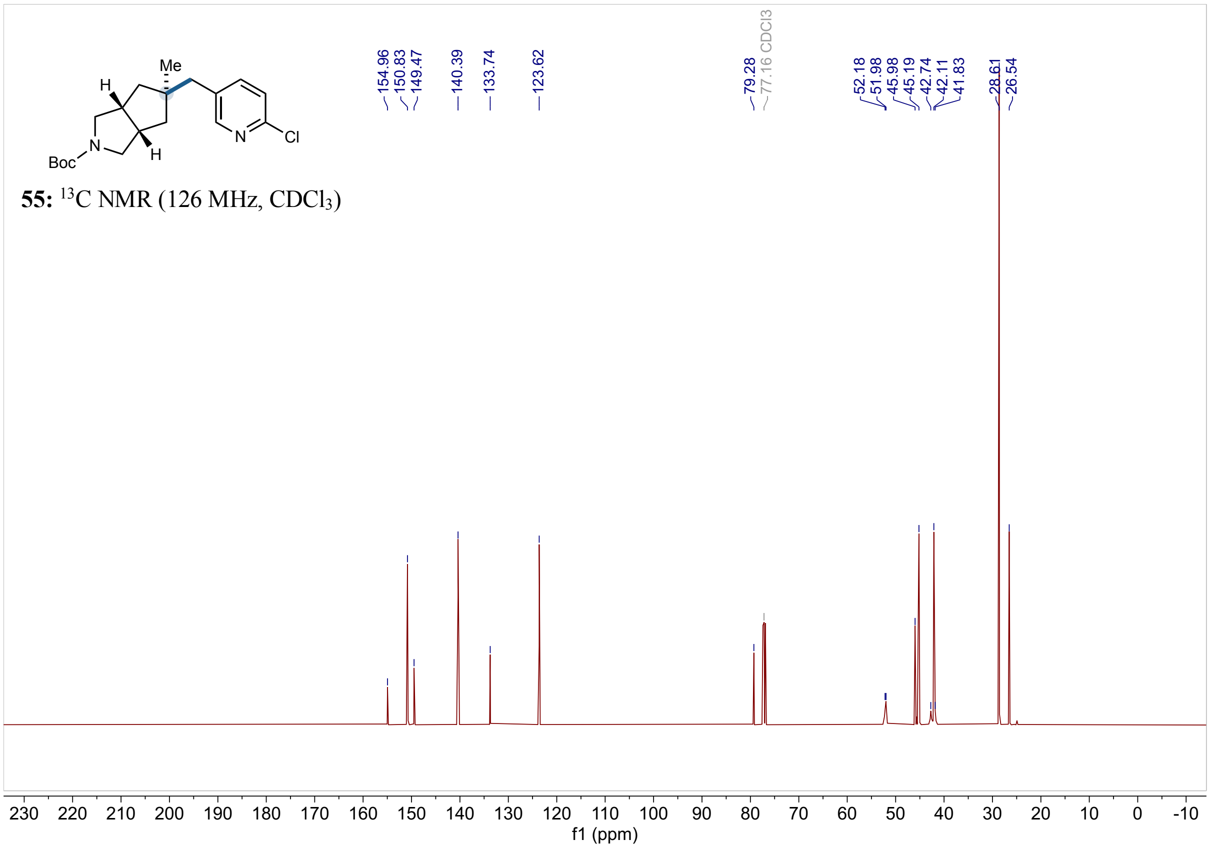

55: ^{13}C NMR (126 MHz, $CDCl_3$)

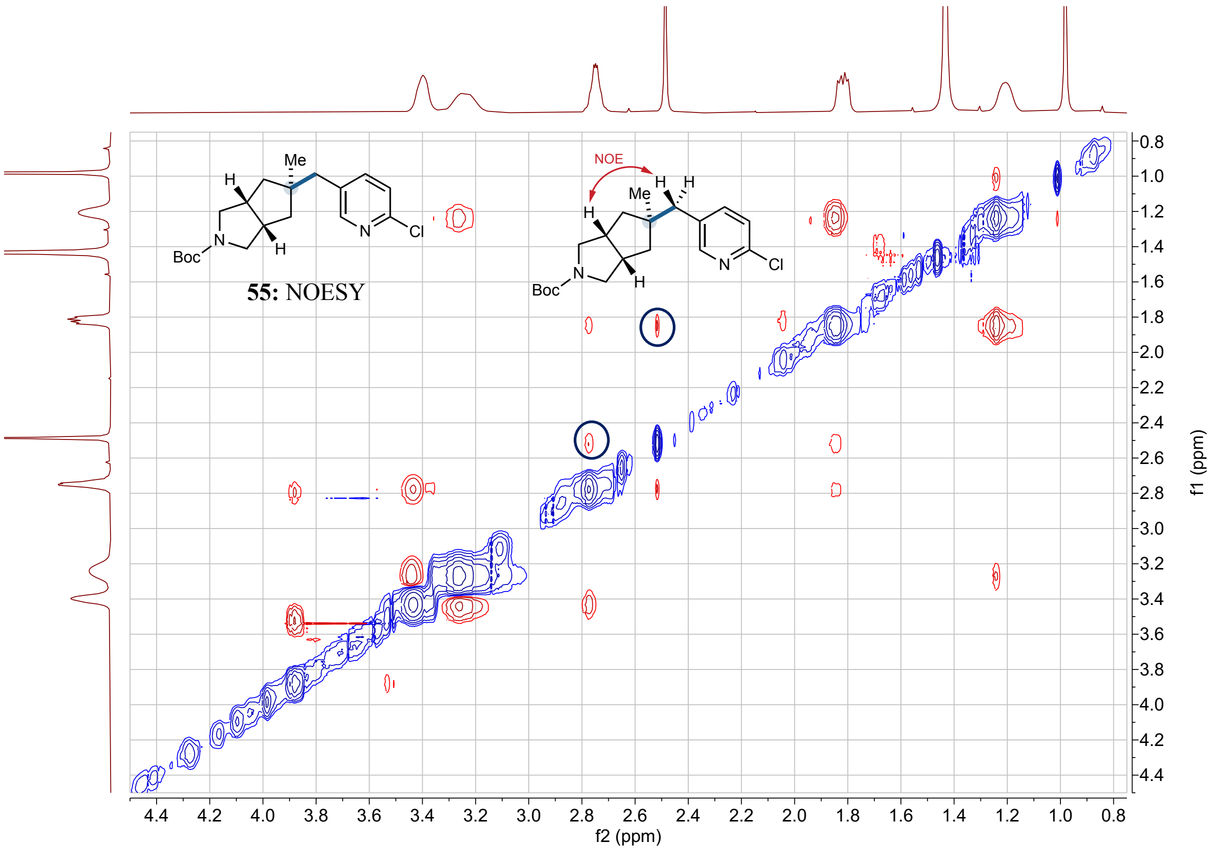
55: NOESY
NOE
Me
Boc
N
Cl
f2 (ppm)
f1 (ppm)

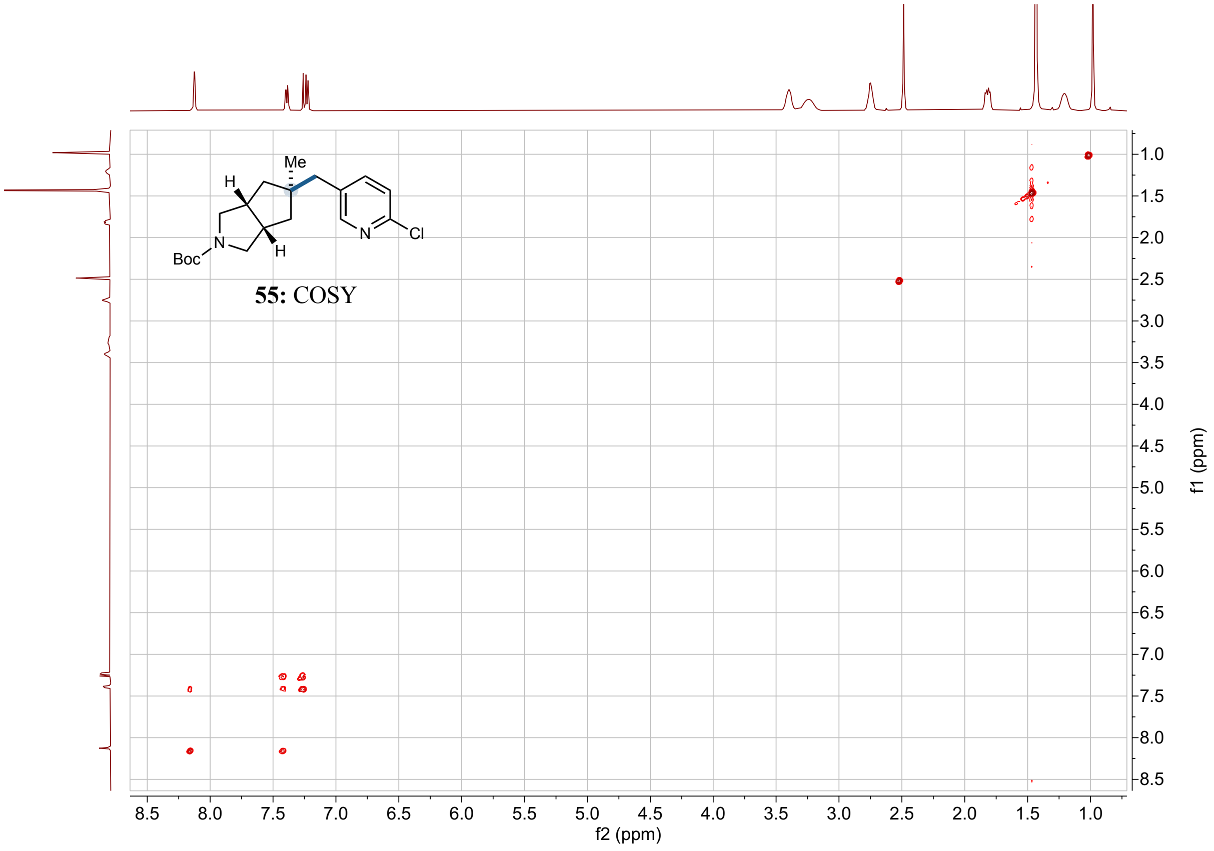
55: COSY
Me
Boc
N
Cl
f2 (ppm)
f1 (ppm)

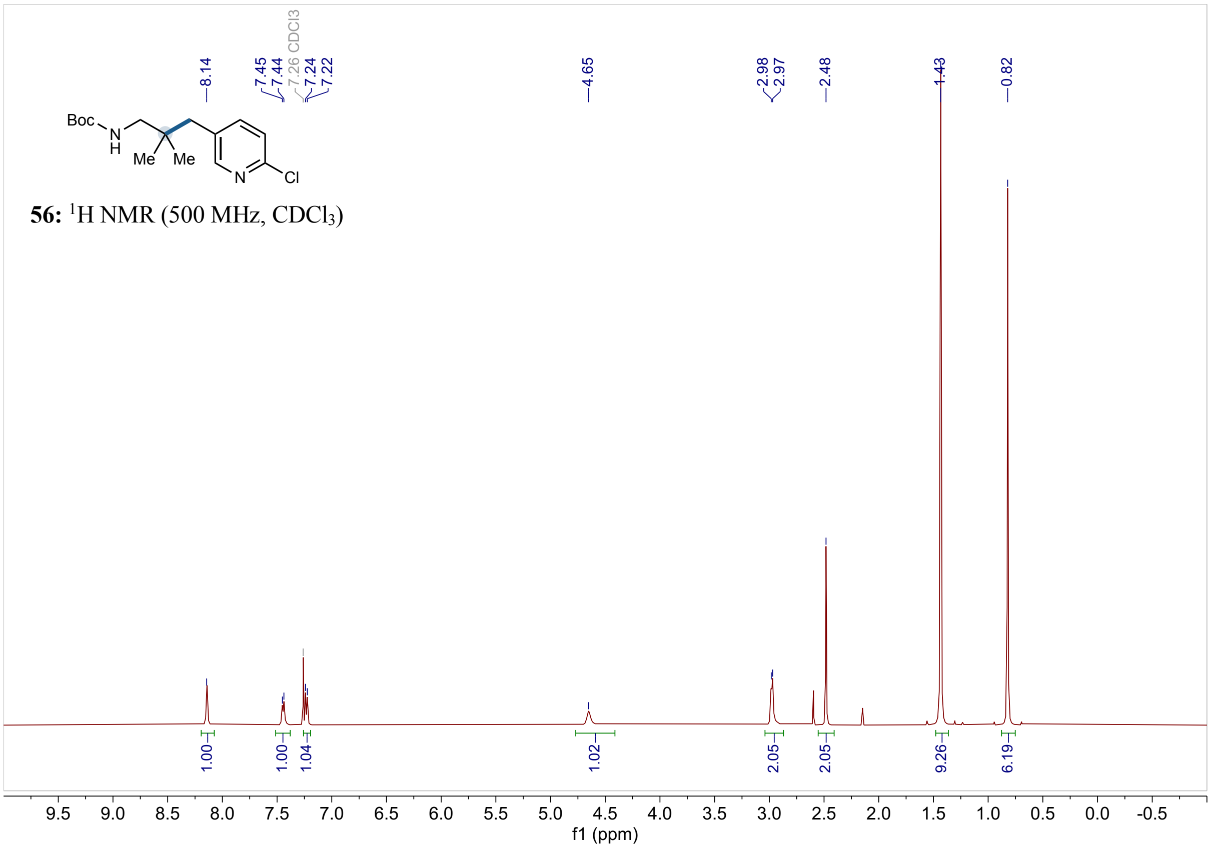

8.14
7.45
7.44
7.26 CDCl3
7.24
7.22
4.65
2.98
2.97
2.48
1.43
0.82
Boc
N
H
Me
Me
N
Cl
56: ¹H NMR (500 MHz, CDCl₃)
1.00
1.00
1.04
1.02
2.05
2.05
9.26
6.19
f1 (ppm)

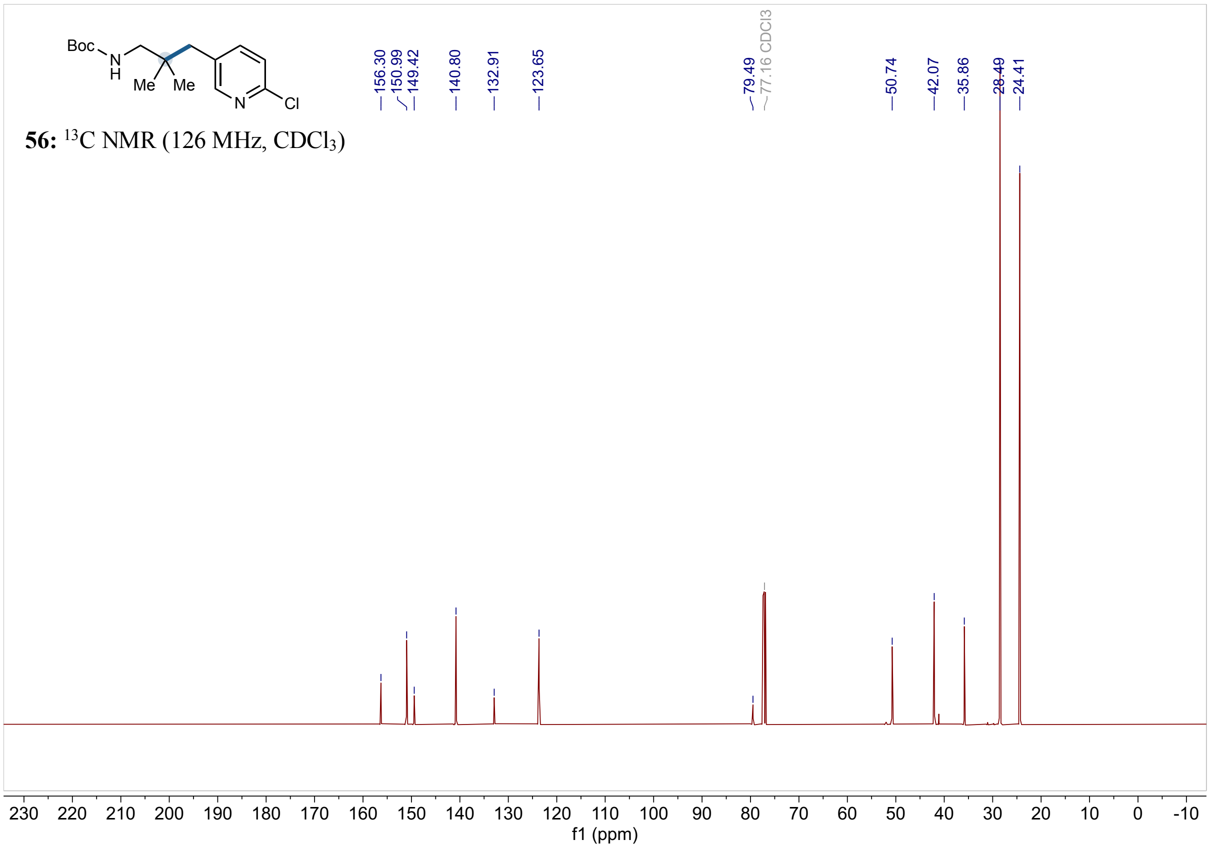

Boc
N
H
Me
Me
N
Cl
56: ¹³C NMR (126 MHz, CDCl₃)
156.30
150.99
149.42
140.80
132.91
123.65
79.49
77.16 CDCl3
50.74
42.07
35.86
28.49
24.41
f1 (ppm)

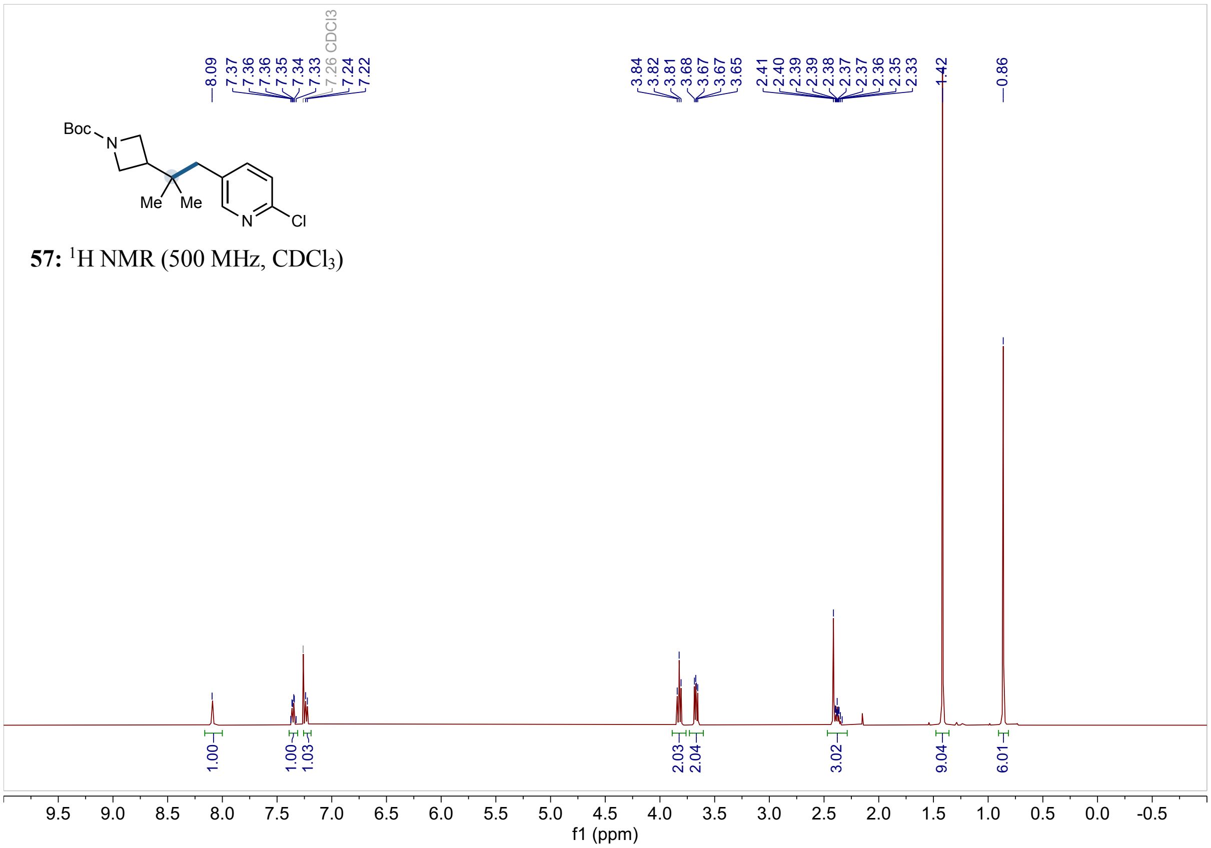

57: ^{1}H NMR (500 MHz, $CDCl_3$)

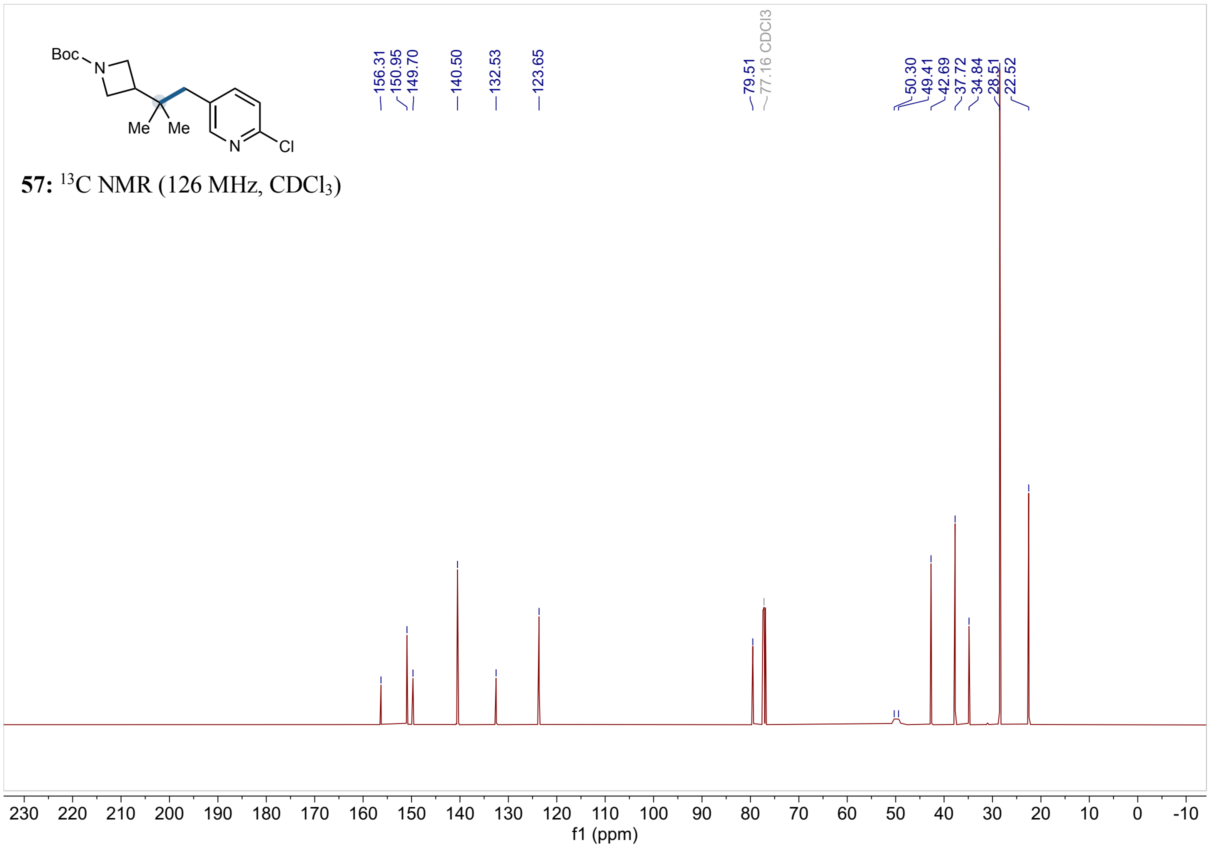

57: ^{13}C NMR (126 MHz, $CDCl_3$)

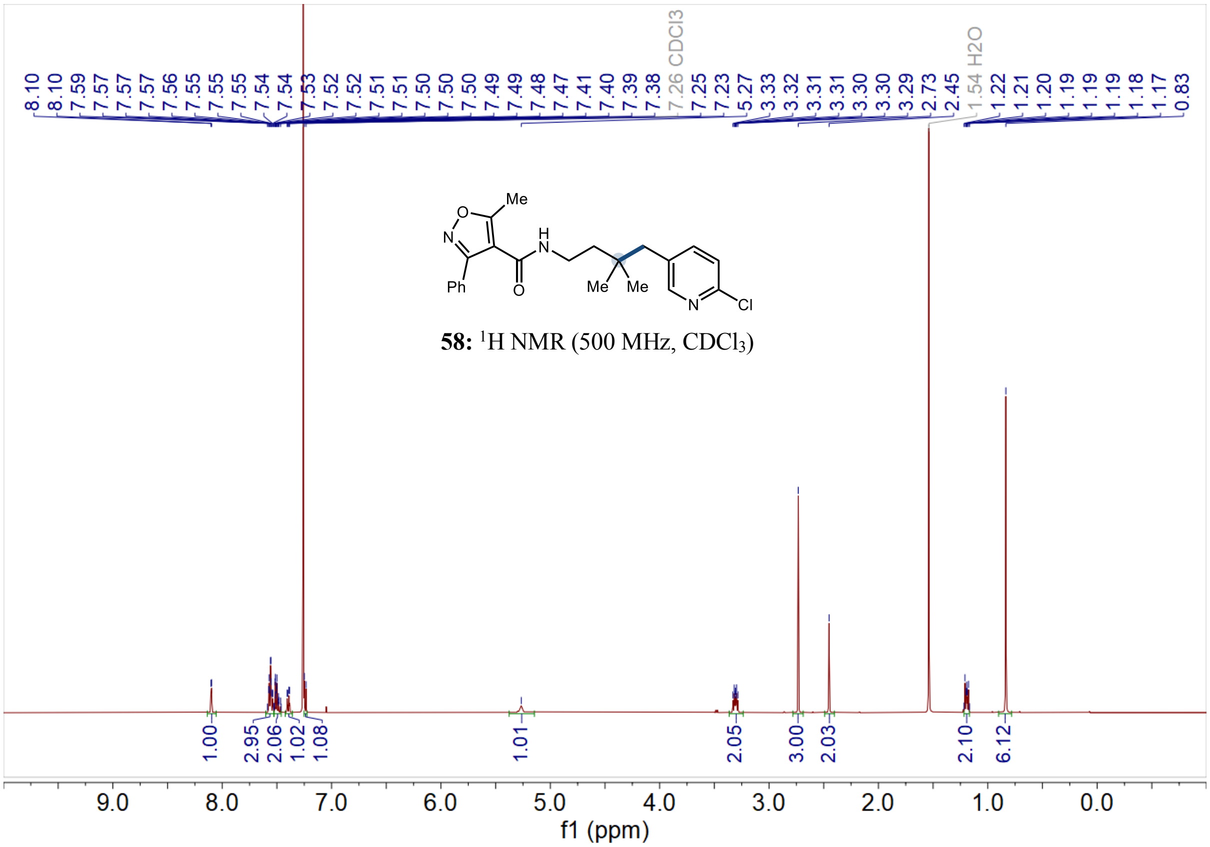

58: ^{1}H NMR (500 MHz, $CDCl_3$)

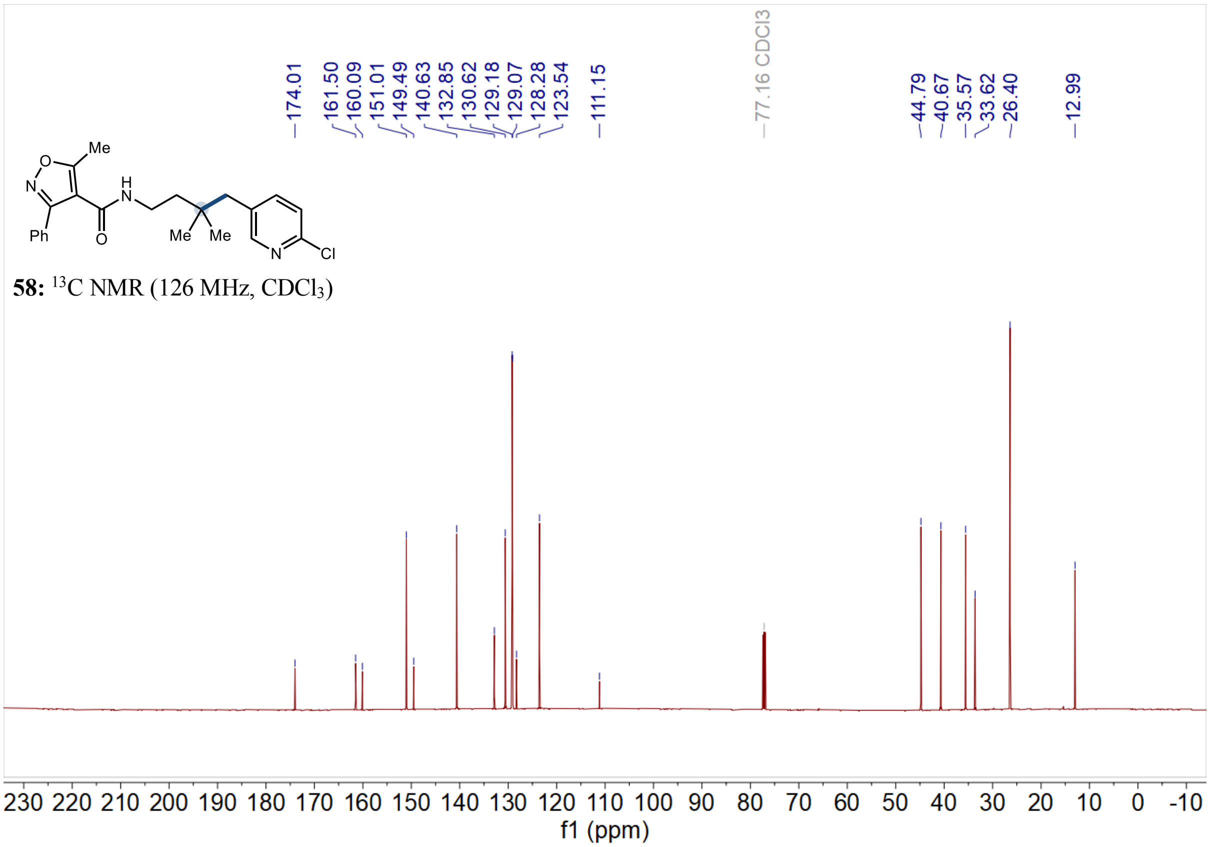

58: ^{13}C NMR (126 MHz, $CDCl_3$)

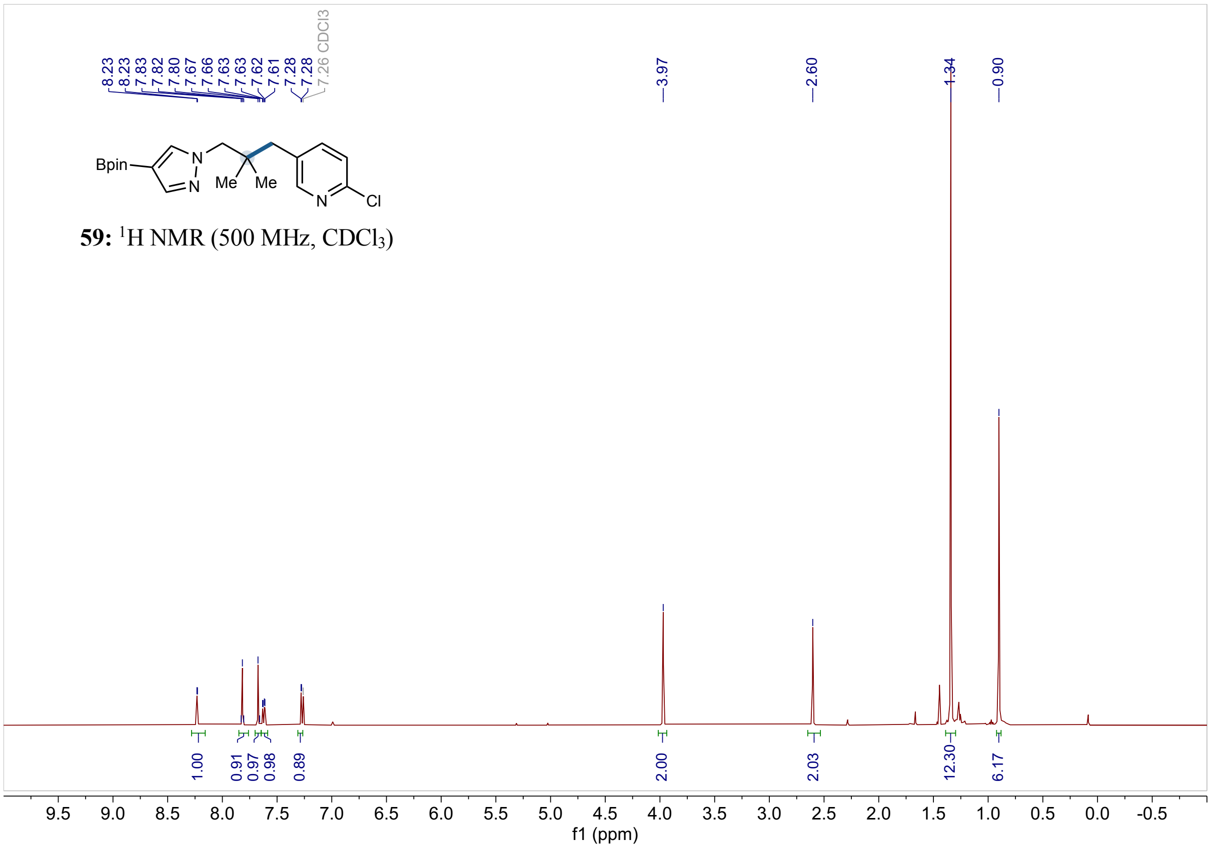

59: ^{1}H NMR (500 MHz, $CDCl_3$)
Bpin
N
N
Me
Me
N
Cl
8.23
8.23
7.83
7.82
7.80
7.67
7.66
7.63
7.63
7.62
7.61
7.28
7.28
7.26 CDCl3
3.97
2.60
1.34
0.90
1.00
0.91
0.97
0.98
0.89
2.00
2.03
12.30
6.17
f1 (ppm)

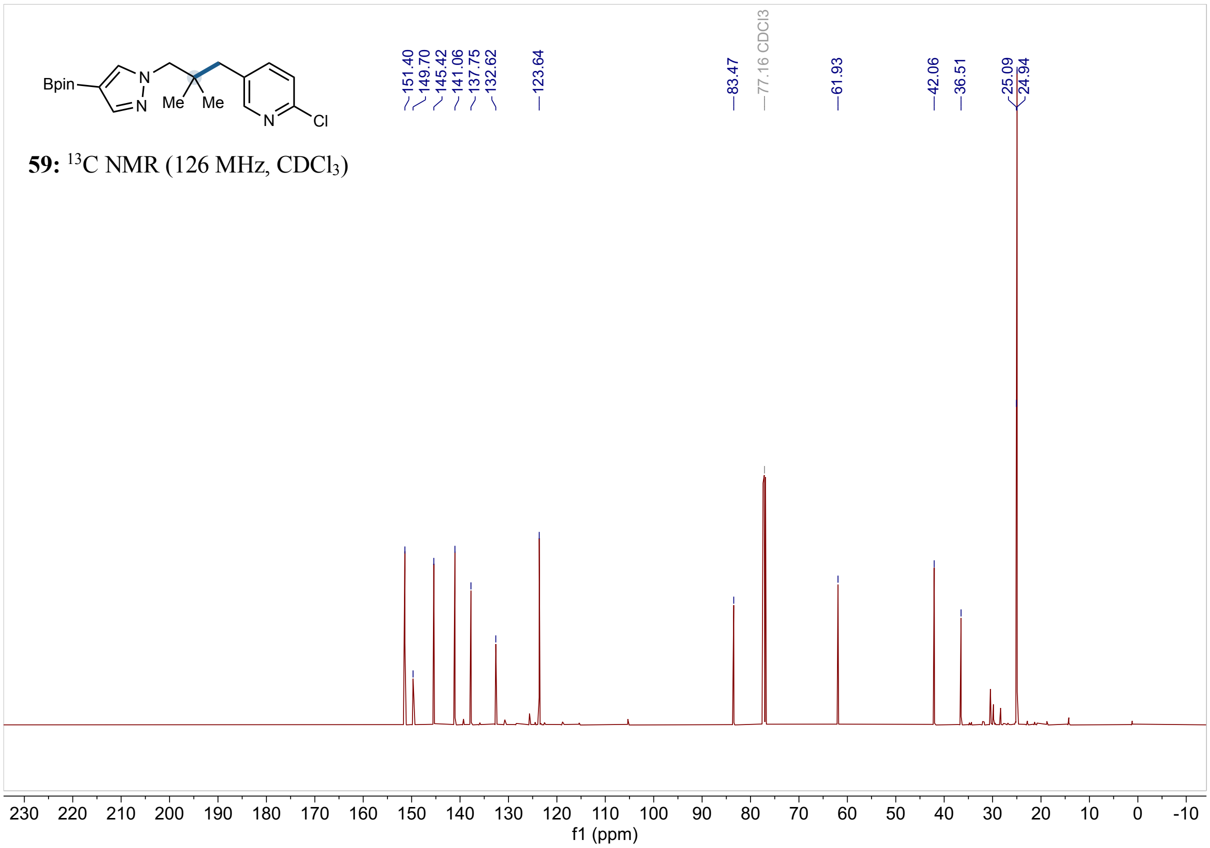

59: ^{13}C NMR (126 MHz, $CDCl_3$)
Bpin
N
N
Me
Me
N
Cl
151.40
149.70
145.42
141.06
137.75
132.62
123.64
83.47
77.16 CDCl3
61.93
42.06
36.51
25.09
24.94
f1 (ppm)

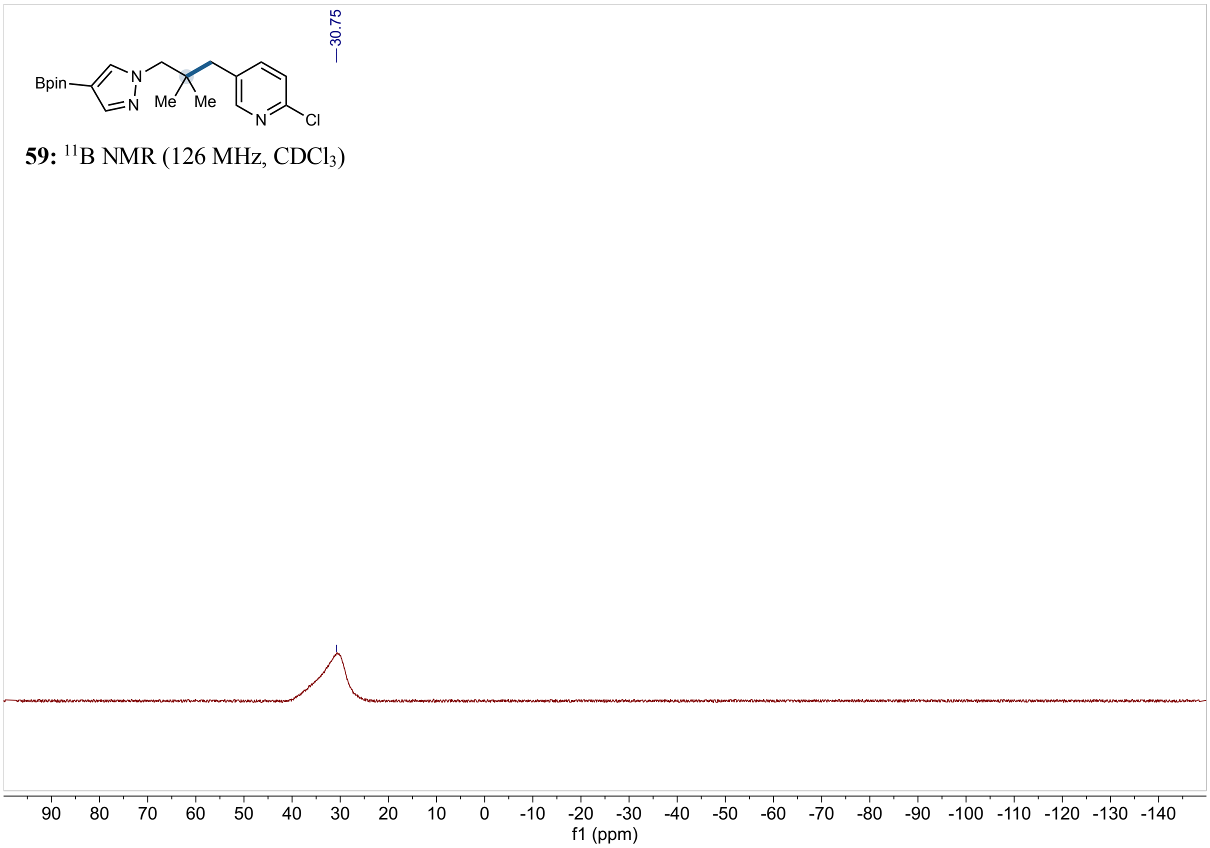

59: ^{11}B NMR (126 MHz, $CDCl_3$)

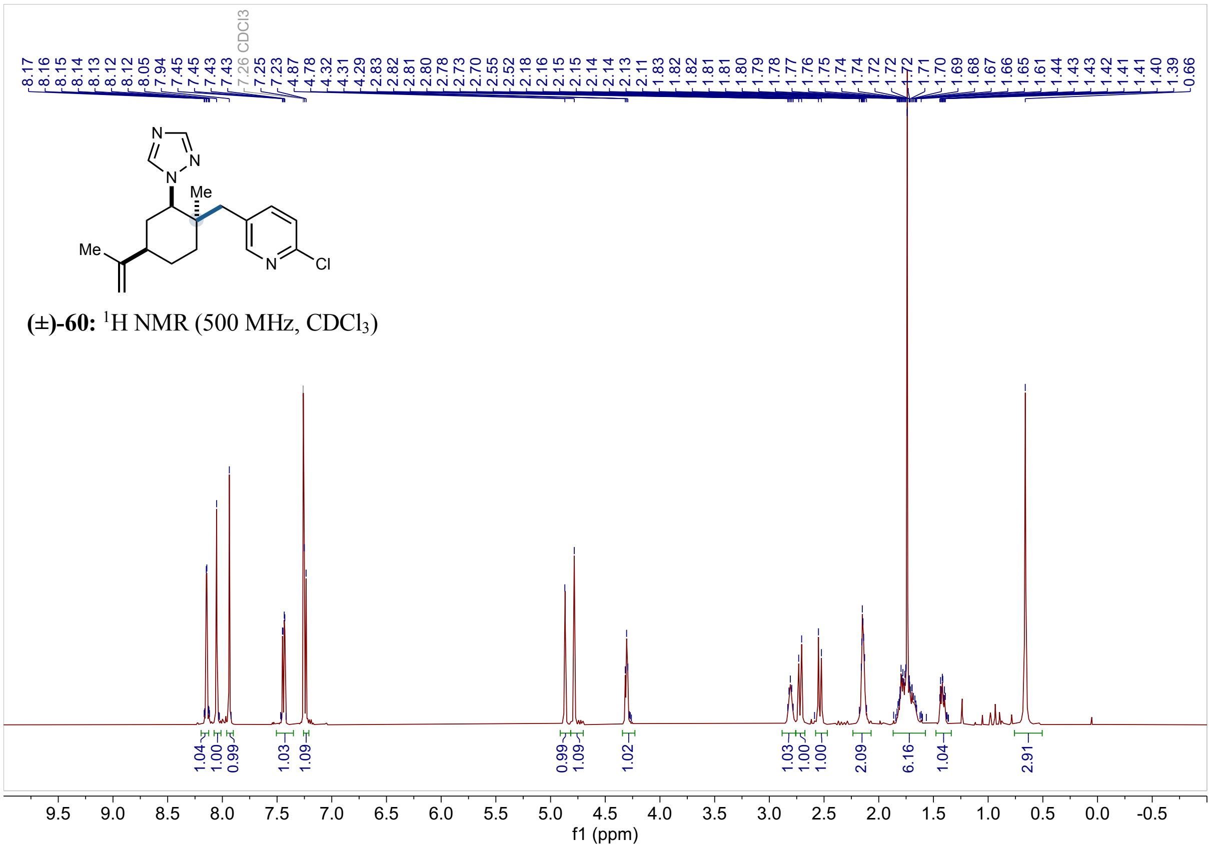
8.17 8.16 8.15 8.14 8.13 8.12 8.12 8.05 7.94 7.45 7.45 7.43 7.43 7.26 CDCl3 7.25 7.23 4.87 4.78 4.32 4.31 4.29 2.83 2.82 2.81 2.80 2.78 2.73 2.70 2.55 2.52 2.18 2.16 2.15 2.15 2.14 2.14 2.13 2.11 1.83 1.82 1.82 1.81 1.81 1.80 1.79 1.78 1.77 1.76 1.75 1.74 1.74 1.72 1.72 1.72 1.71 1.70 1.69 1.68 1.67 1.66 1.65 1.61 1.44 1.43 1.43 1.42 1.41 1.41 1.40 1.39 0.66
N
N
N
Me
Me
N
Cl
(±)-60: 1H NMR (500 MHz, CDCl3)
1.04 1.00 0.99 1.03 1.09 0.99 1.09 1.02 1.03 1.00 1.00 2.09 6.16 1.04 2.91
9.5 9.0 8.5 8.0 7.5 7.0 6.5 6.0 5.5 5.0 4.5 4.0 3.5 3.0 2.5 2.0 1.5 1.0 0.5 0.0 -0.5
f1 (ppm)

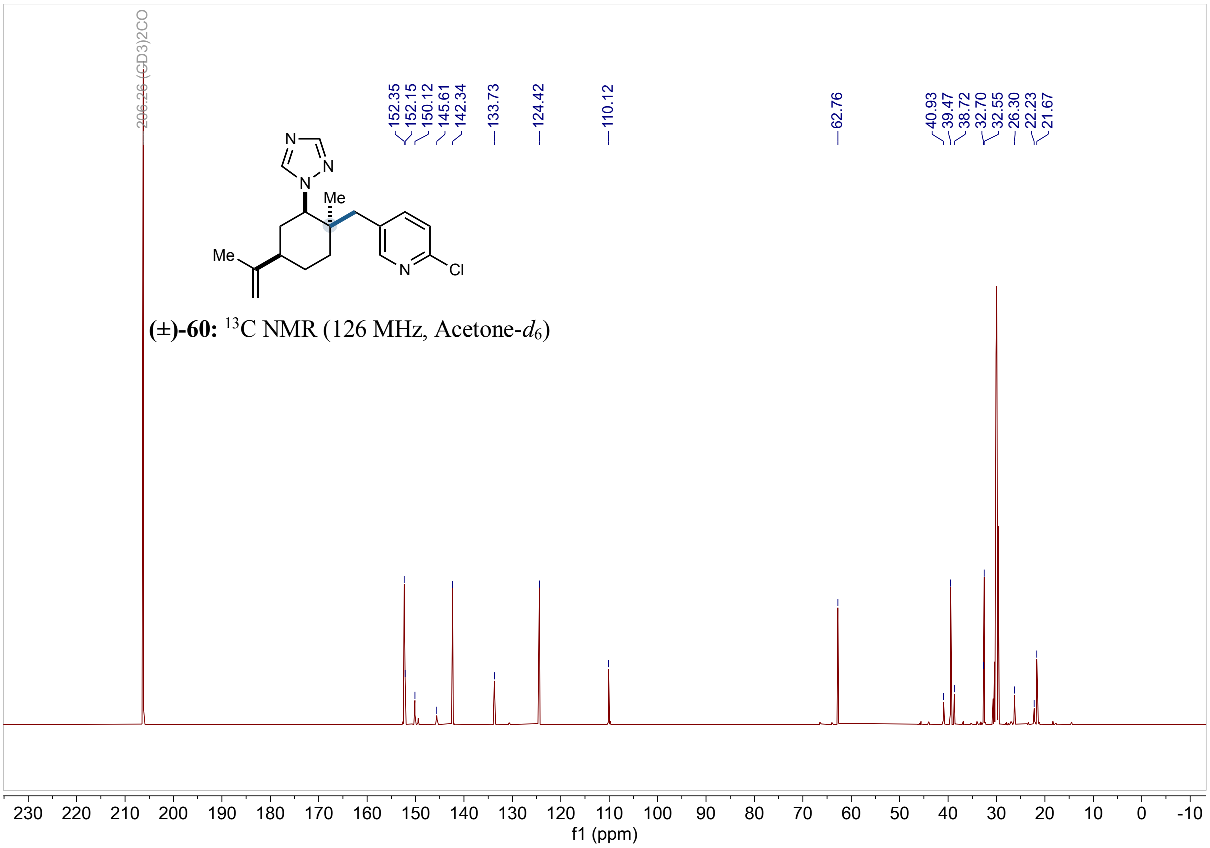
206.26 (CD3)2CO
152.35 152.15 150.12 145.61 142.34
133.73
124.42
110.12
62.76
40.93 39.47 38.72 32.70 32.55 26.30 22.23 21.67
N
N
N
Me
Me
N
Cl
(±)-60: 13C NMR (126 MHz, Acetone-d6)
230 220 210 200 190 180 170 160 150 140 130 120 110 100 90 80 70 60 50 40 30 20 10 0 -10
f1 (ppm)

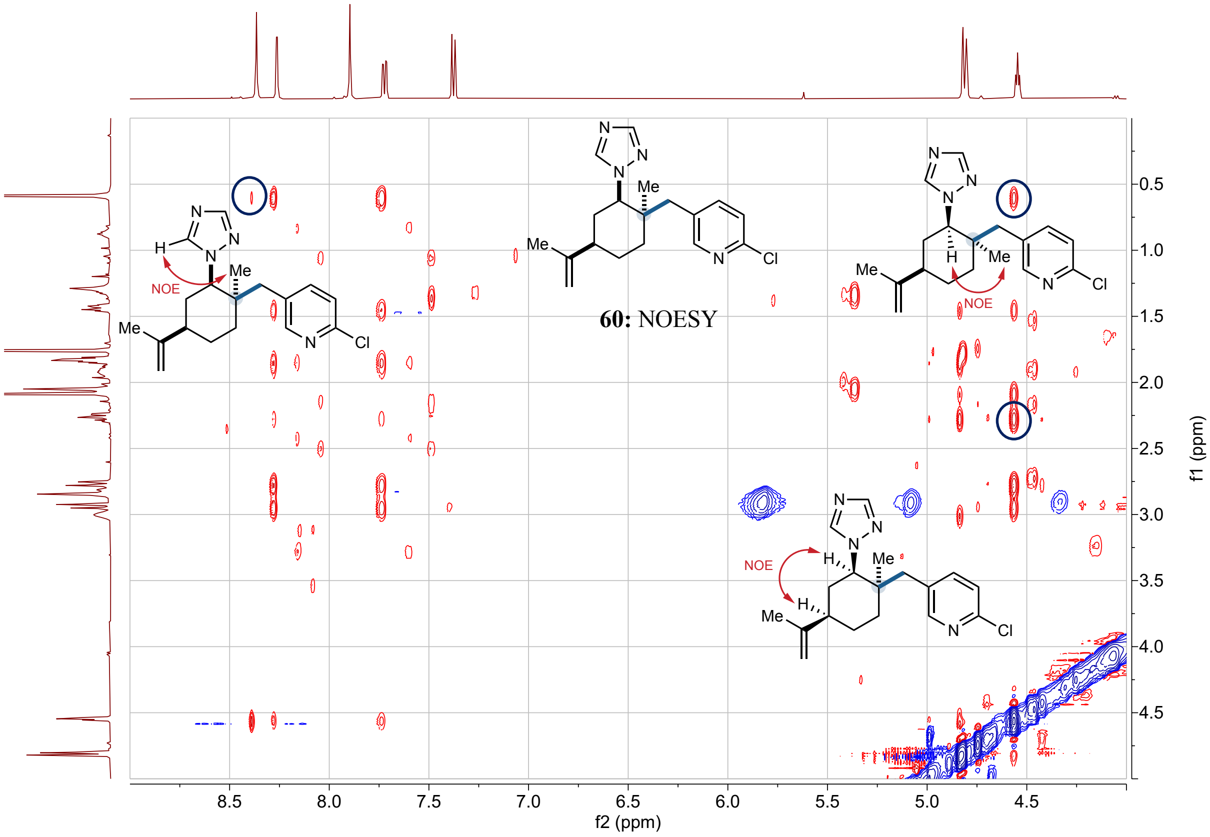

NOE
Me
60: NOESY
f2 (ppm)
f1 (ppm)

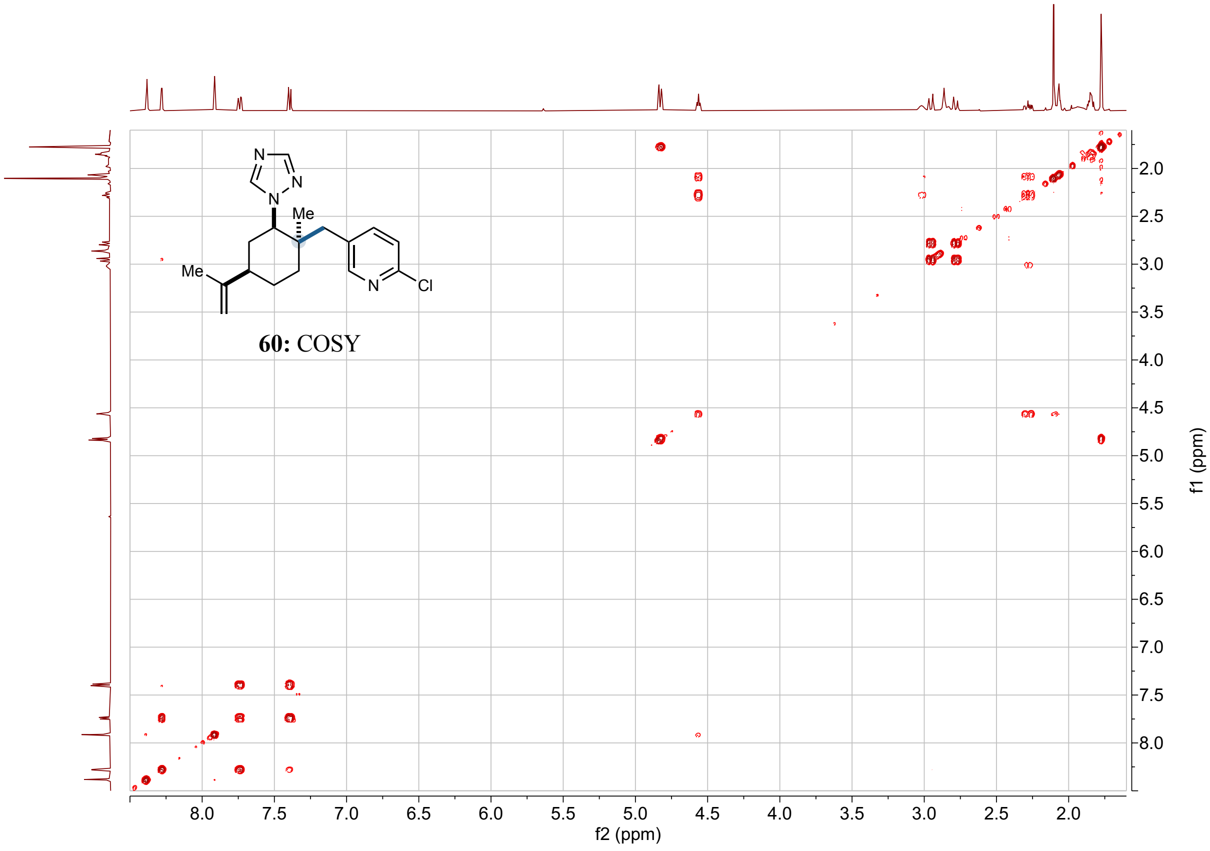

Me
60: COSY
f2 (ppm)
f1 (ppm)

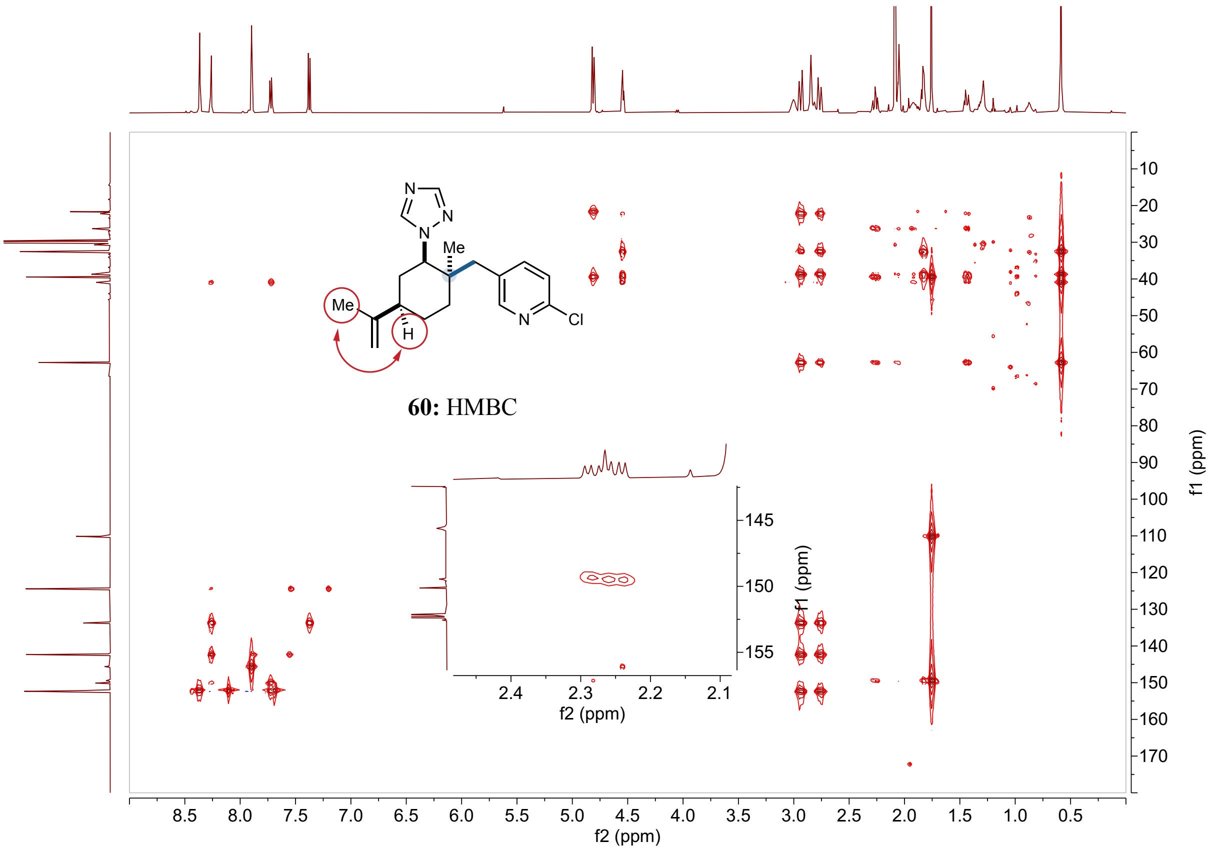
N
N
N
Me
Me
H
N
Cl
60: HMBC
2.4
2.3
2.2
2.1
f2 (ppm)
145
150
155
f1 (ppm)
8.5
8.0
7.5
7.0
6.5
6.0
5.5
5.0
4.5
4.0
3.5
3.0
2.5
2.0
1.5
1.0
0.5
f2 (ppm)
10
20
30
40
50
60
70
80
90
100
110
120
130
140
150
160
170
f1 (ppm)

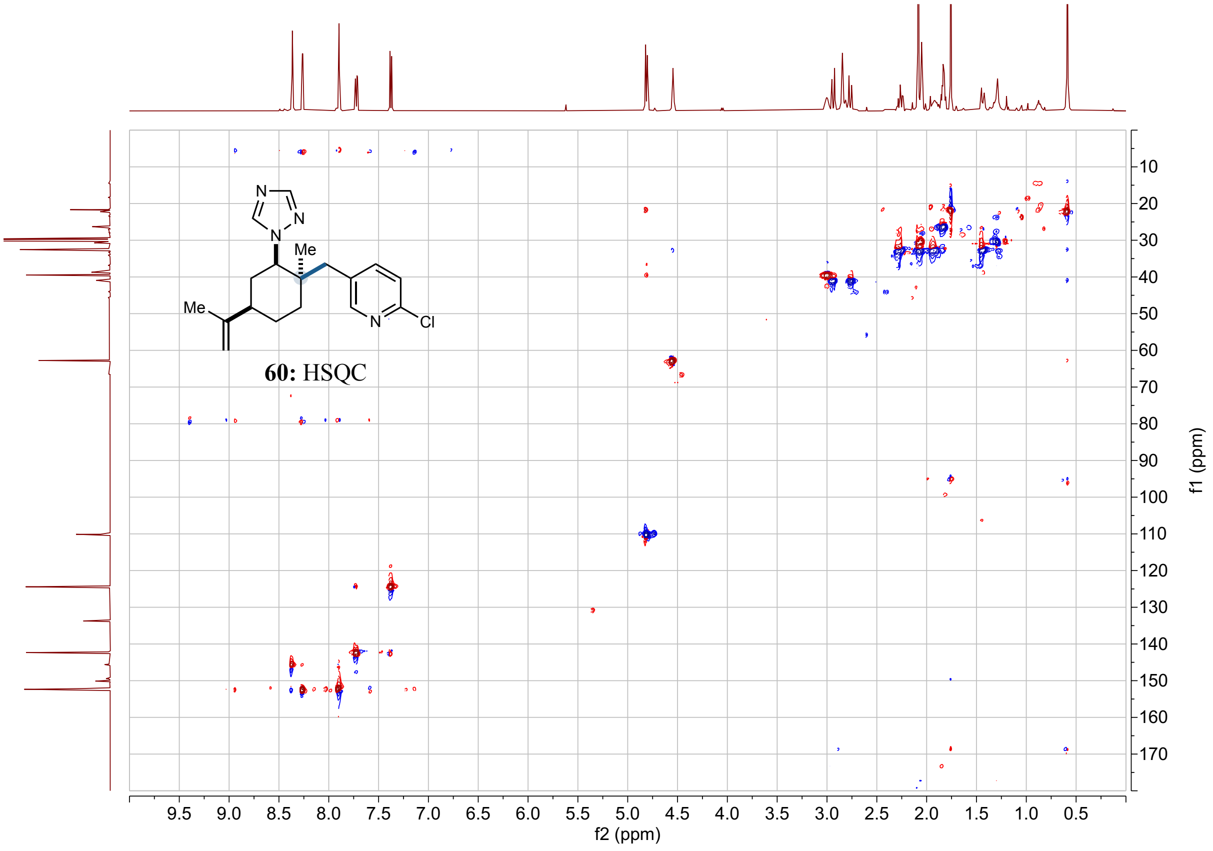
N
N
N
Me
Me
N
Cl
60: HSQC
9.5
9.0
8.5
8.0
7.5
7.0
6.5
6.0
5.5
5.0
4.5
4.0
3.5
3.0
2.5
2.0
1.5
1.0
0.5
f2 (ppm)
10
20
30
40
50
60
70
80
90
100
110
120
130
140
150
160
170
f1 (ppm)

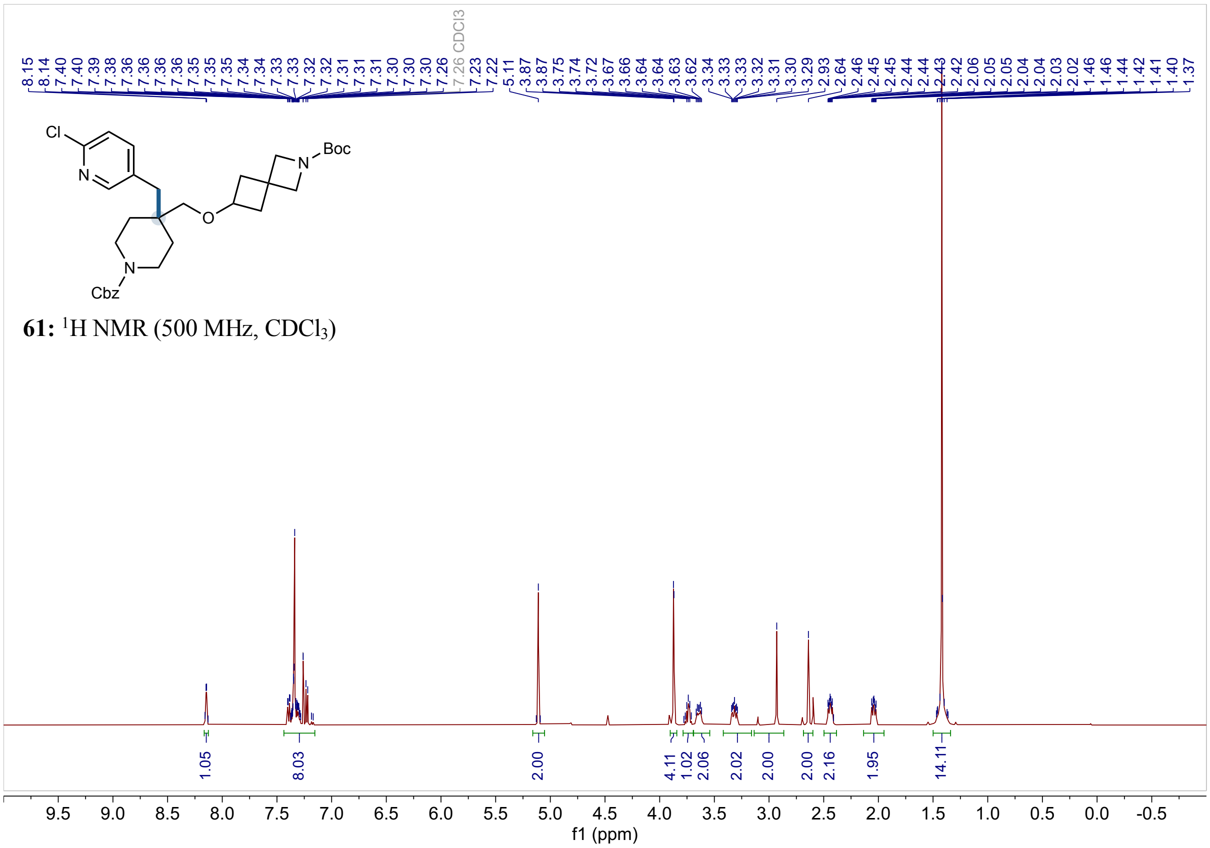

61: ^{1}H NMR (500 MHz, $CDCl_3$)

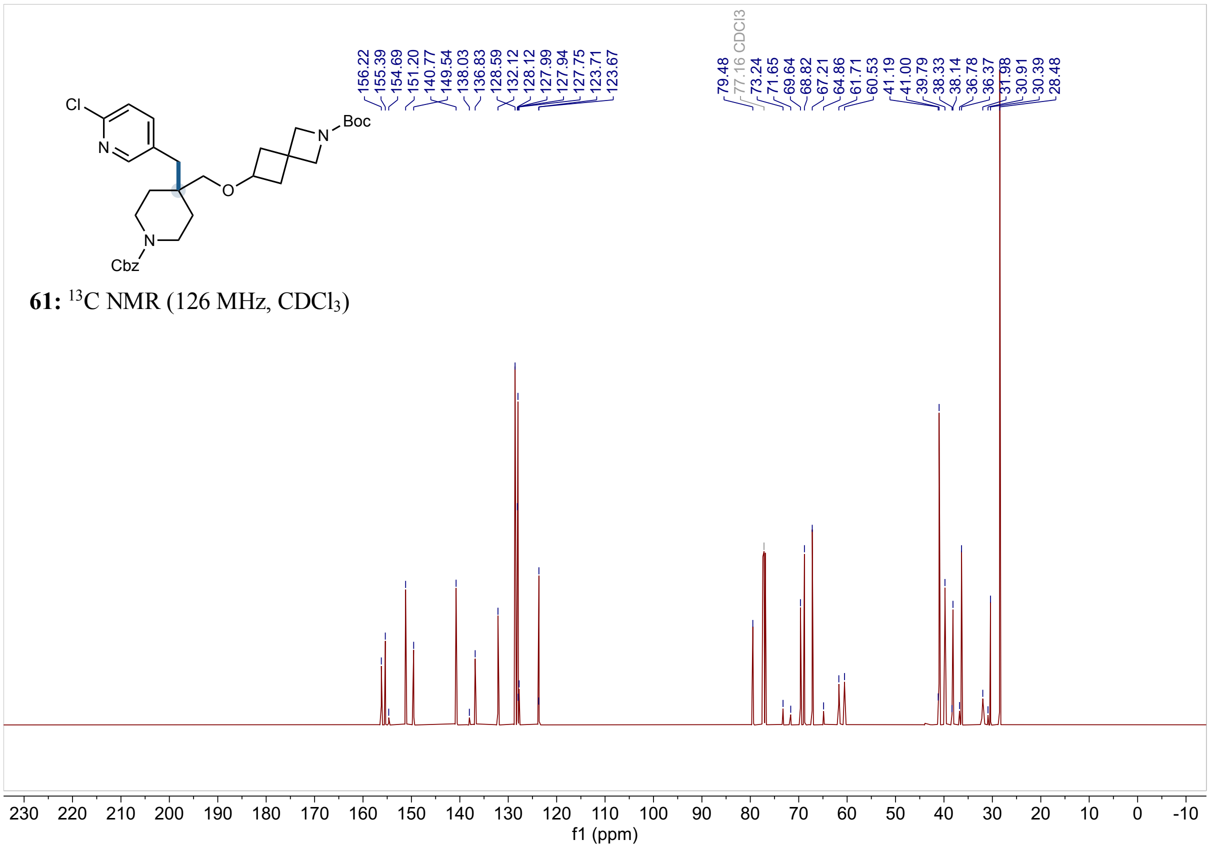

61: ^{13}C NMR (126 MHz, $CDCl_3$)

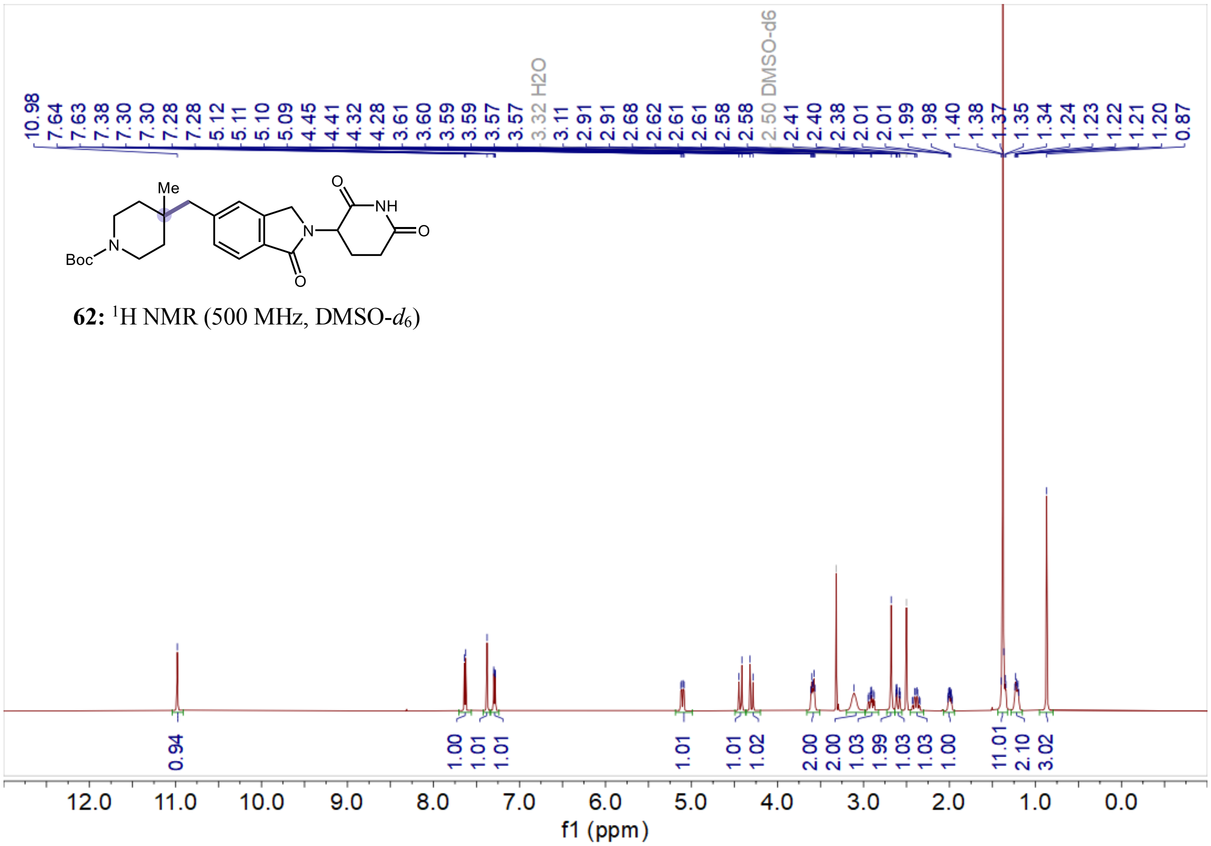
62: ¹H NMR (500 MHz, DMSO-d6)
10.98
7.64
7.63
7.38
7.30
7.30
7.28
7.28
5.12
5.11
5.10
5.09
4.45
4.41
4.32
4.28
3.61
3.60
3.59
3.59
3.57
3.57
3.32 H2O
3.11
2.91
2.91
2.68
2.62
2.61
2.61
2.58
2.58
2.50 DMSO-d6
2.41
2.40
2.38
2.01
2.01
1.99
1.98
1.40
1.38
1.37
1.35
1.34
1.24
1.23
1.22
1.21
1.20
0.87
0.94
1.00
1.01
1.01
1.01
1.01
1.02
2.00
2.00
1.03
1.99
1.03
1.03
1.00
11.01
2.10
3.02
f1 (ppm)

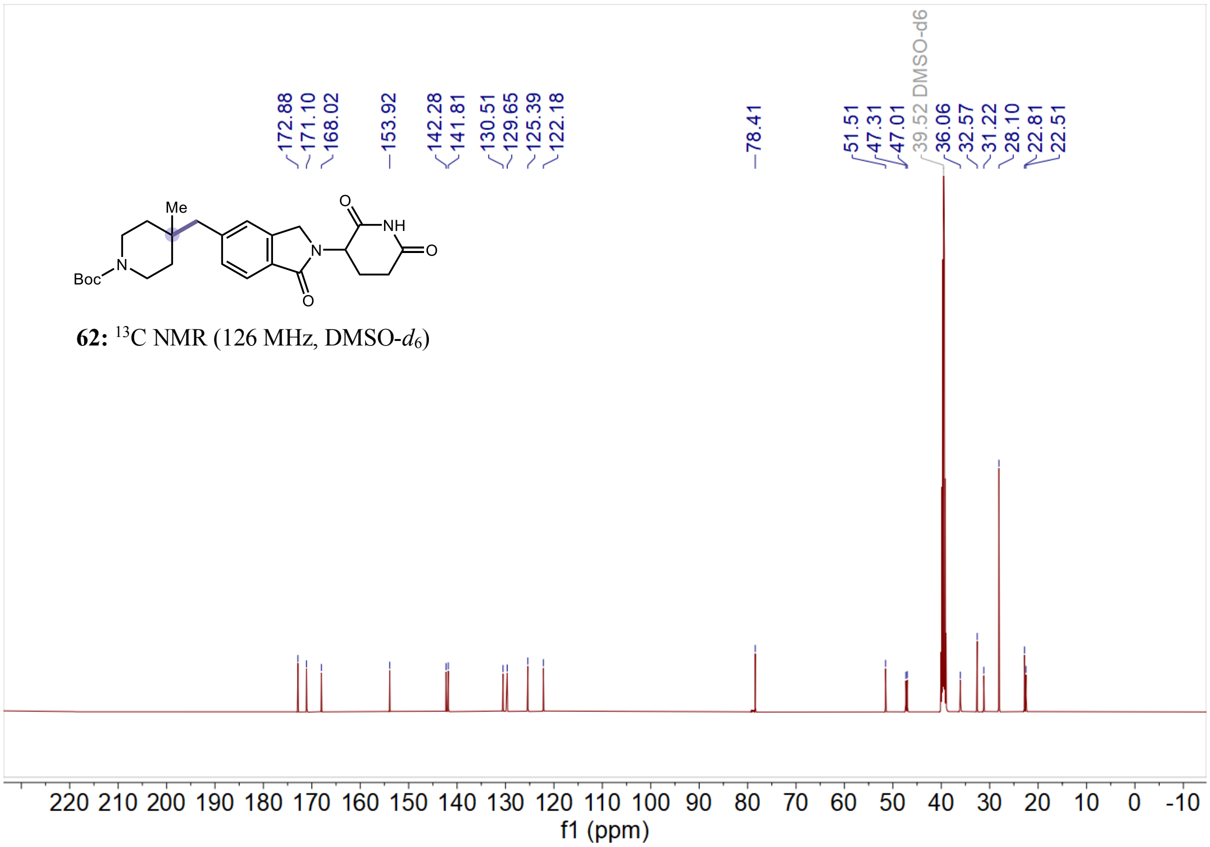
62: ¹³C NMR (126 MHz, DMSO-d6)
172.88
171.10
168.02
153.92
142.28
141.81
130.51
129.65
125.39
122.18
78.41
51.51
47.31
47.01
39.52 DMSO-d6
36.06
32.57
31.22
28.10
22.81
22.51
f1 (ppm)

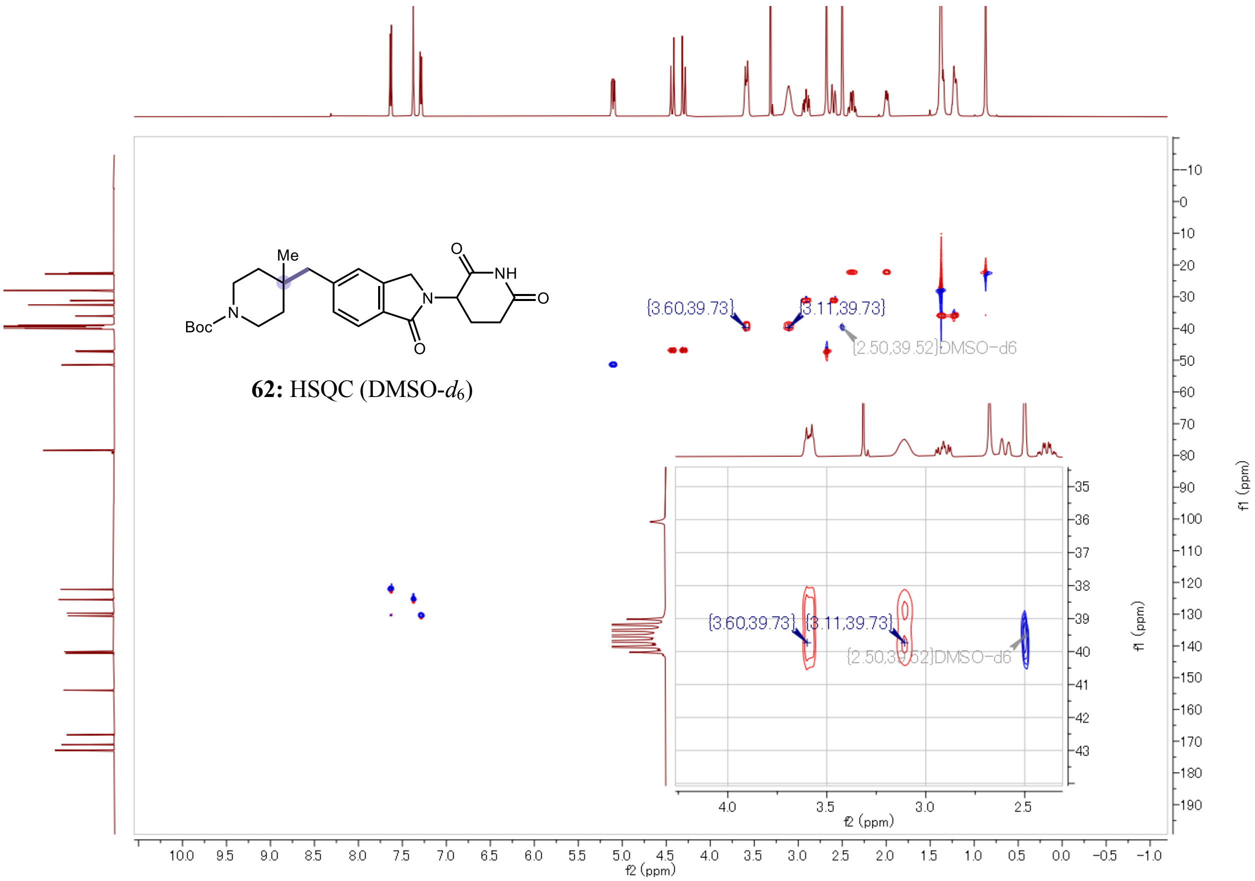

Me
NH
O
O
N
O
Boc
N
62: HSQC (DMSO-d6)
{3.60,39.73}
{3.11,39.73}
{2.50,39.52}DMSO-d6
f2 (ppm)
f1 (ppm)

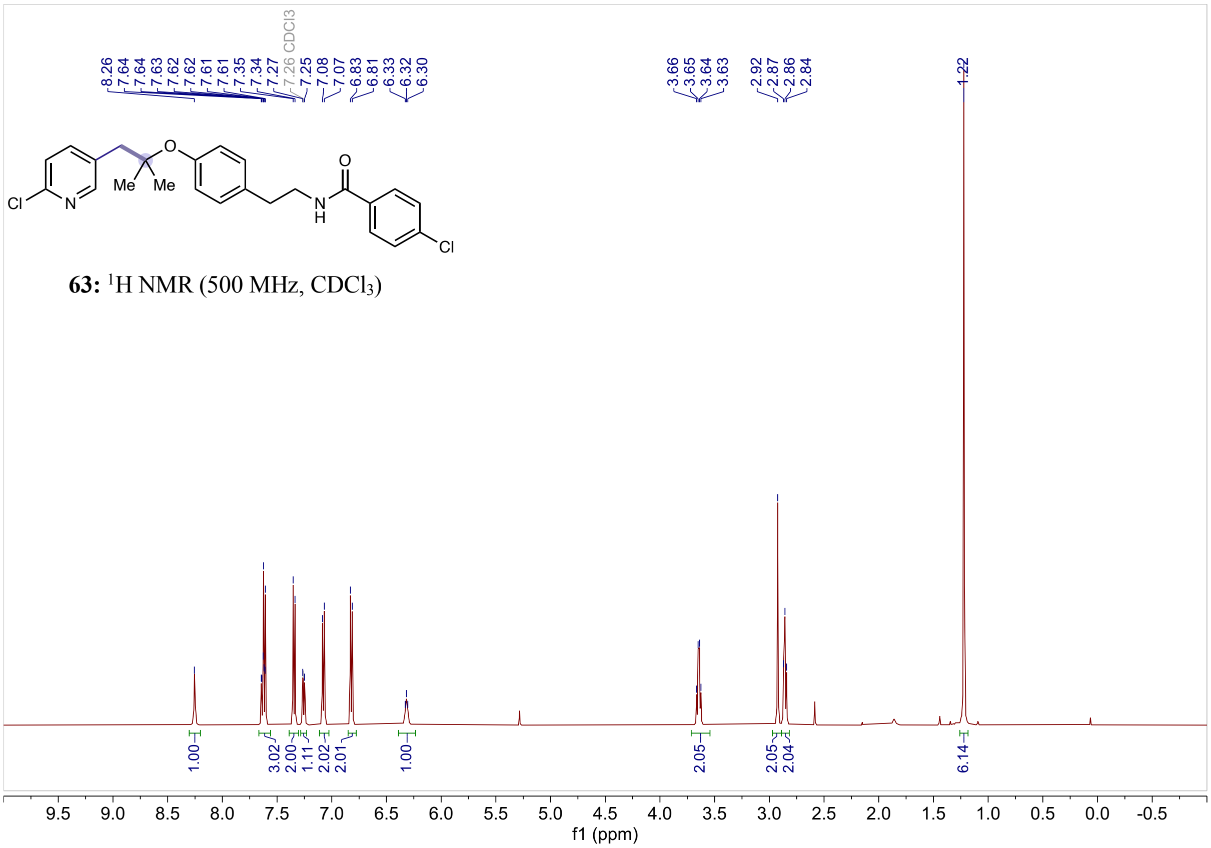

63: ^{1}H NMR (500 MHz, $CDCl_3$)

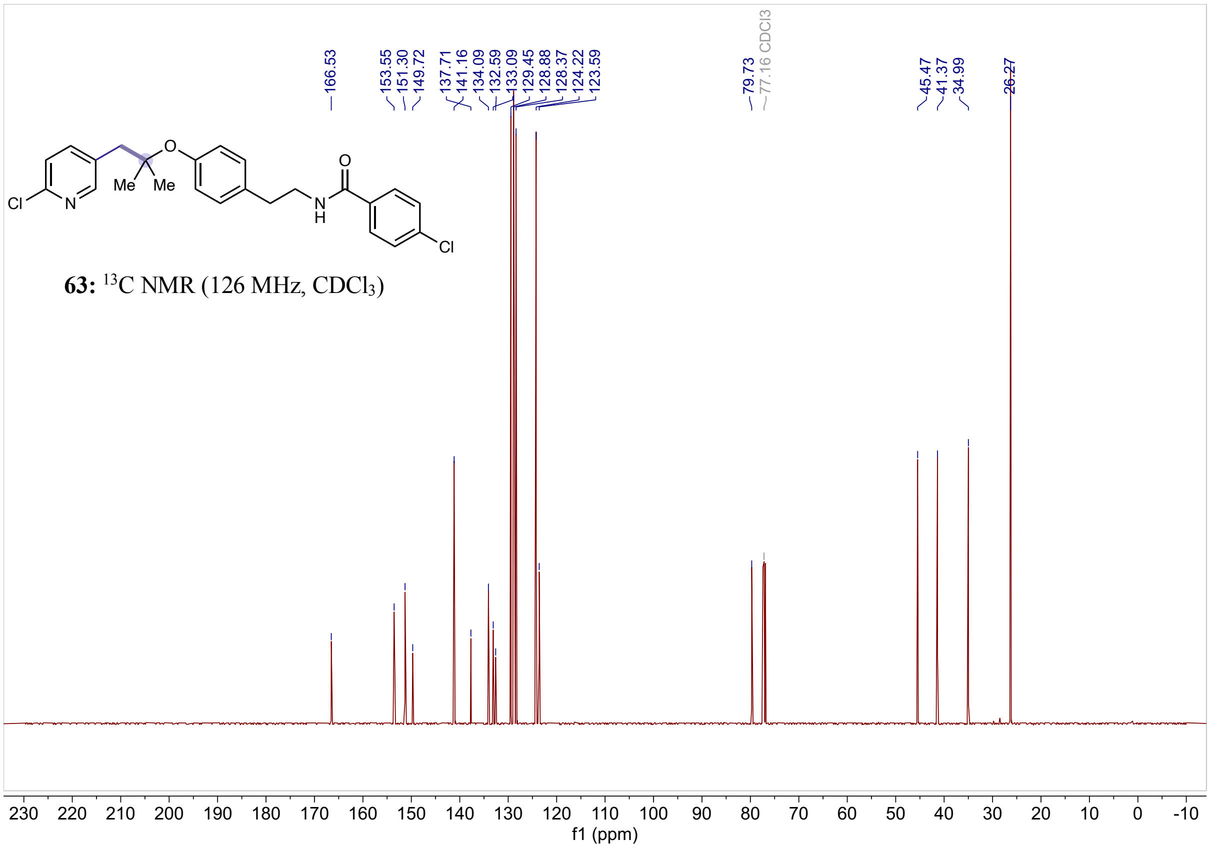

63: ^{13}C NMR (126 MHz, $CDCl_3$)

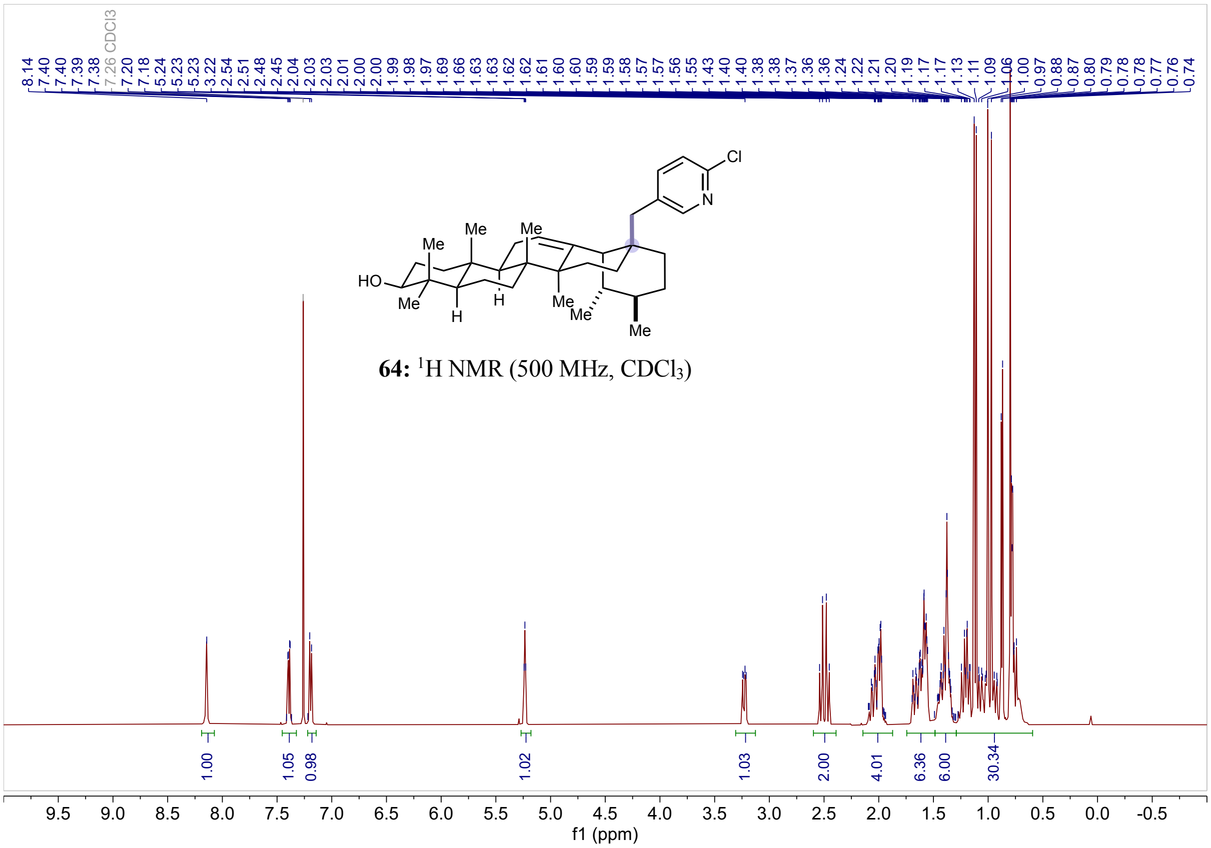

64: ^{1}H NMR (500 MHz, $CDCl_3$)

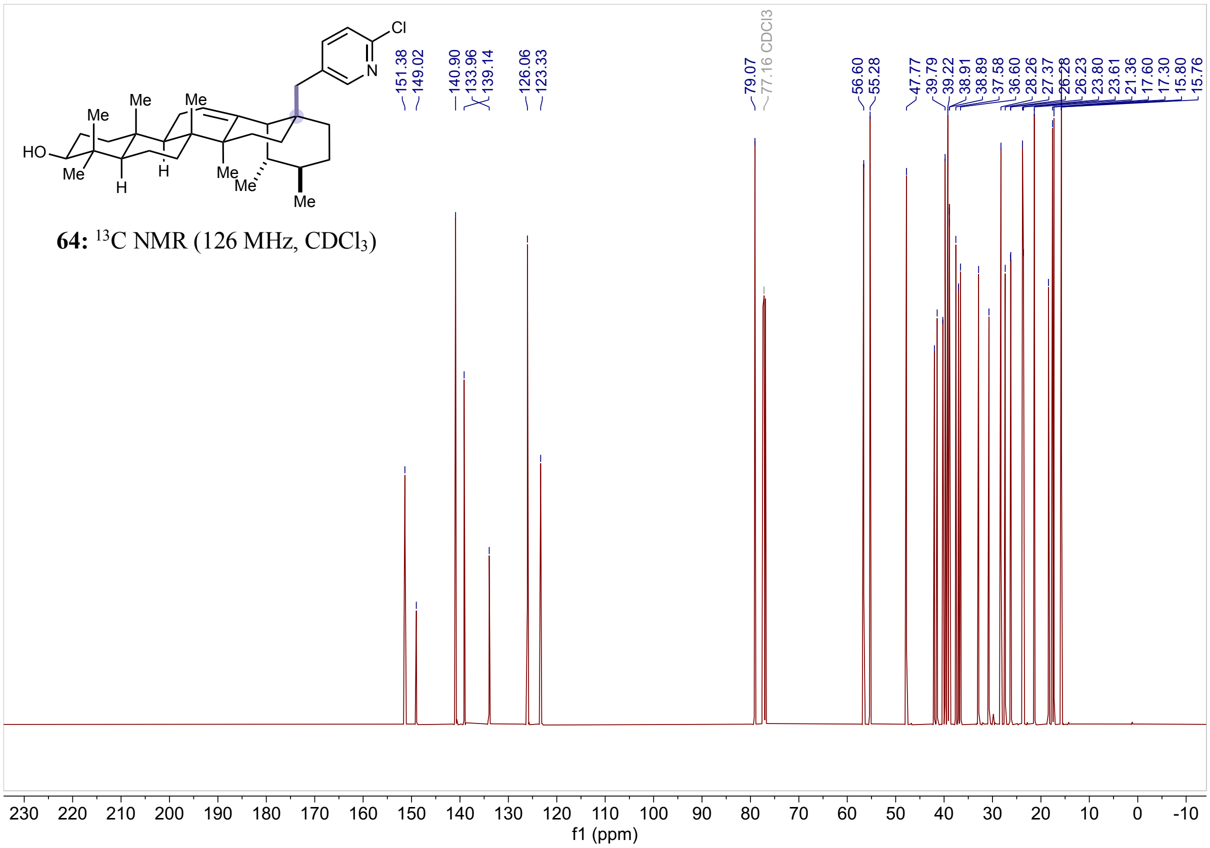

64: ^{13}C NMR (126 MHz, $CDCl_3$)

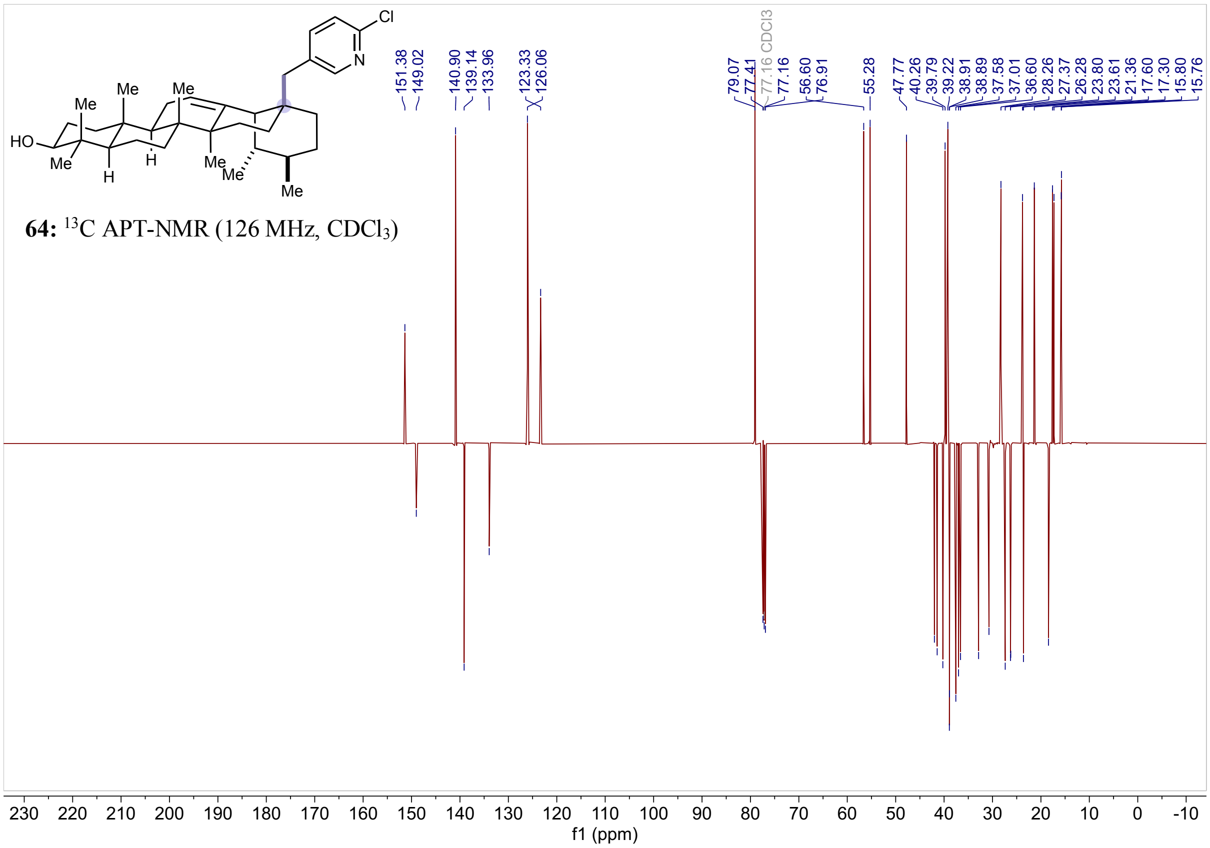
64: 13C APT-NMR (126 MHz, CDCl3)
151.38
149.02
140.90
139.14
133.96
123.33
126.06
79.07
77.41
77.16 CDCl3
77.16
56.60
76.91
55.28
47.77
40.26
39.79
39.22
38.91
38.89
37.58
37.01
36.60
28.26
27.37
26.28
23.80
23.61
21.36
17.60
17.30
15.80
15.76
f1 (ppm)

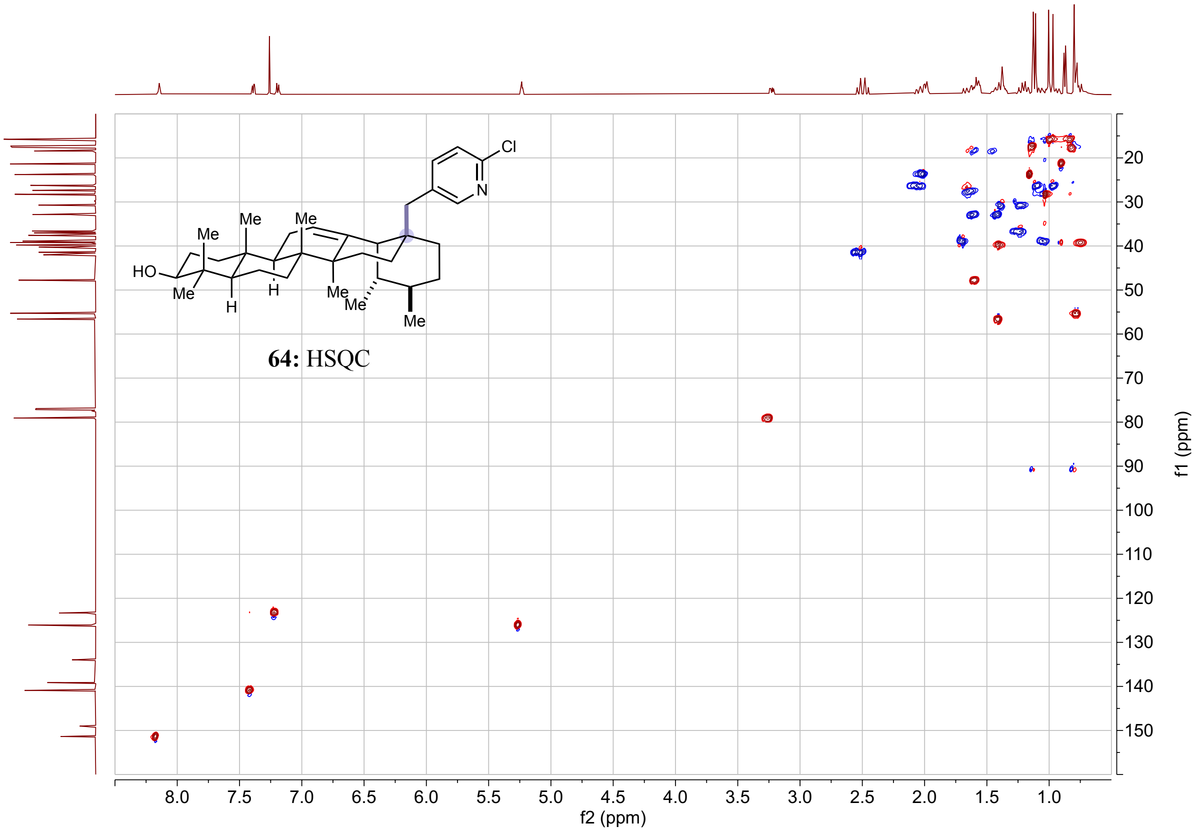
64: HSQC
f2 (ppm)
f1 (ppm)

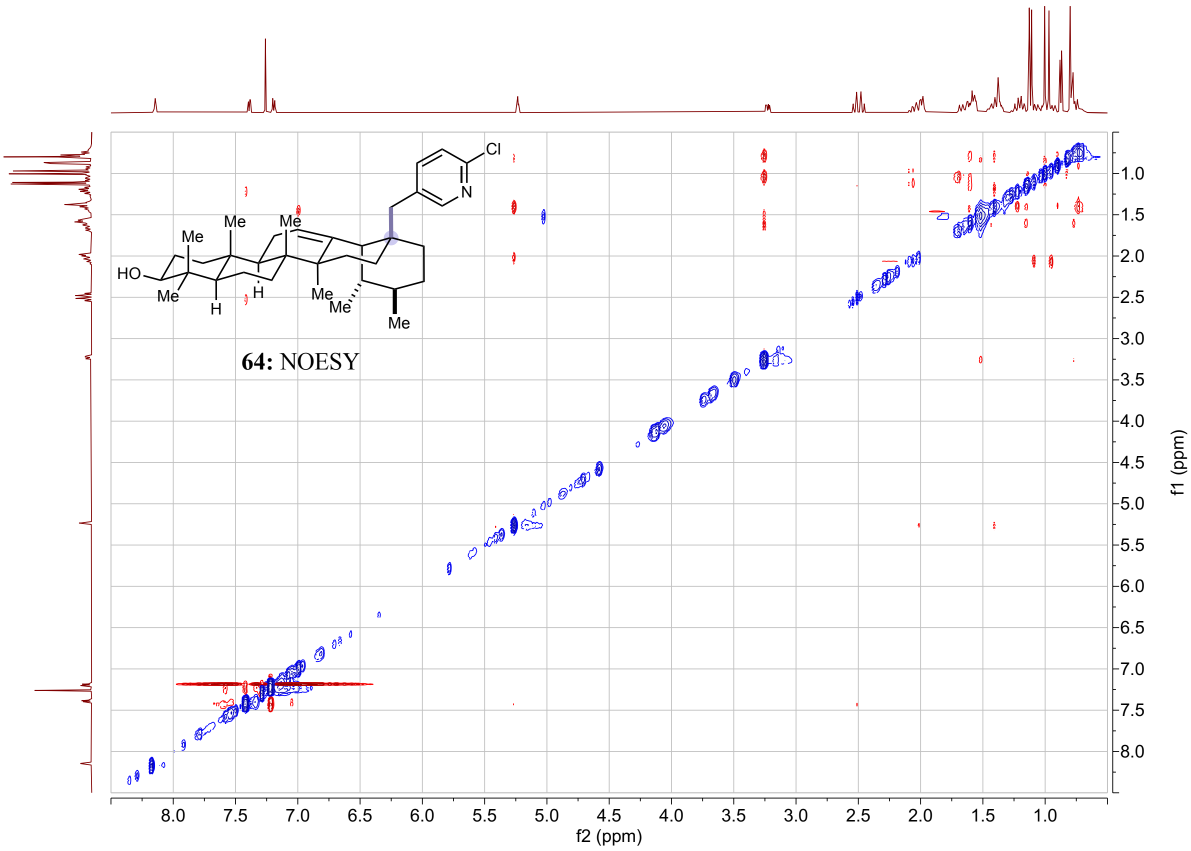
HO
Me
Me
H
Me
H
Me
Me
Me
Me
Cl
N
64: NOESY
f1 (ppm)
f2 (ppm)

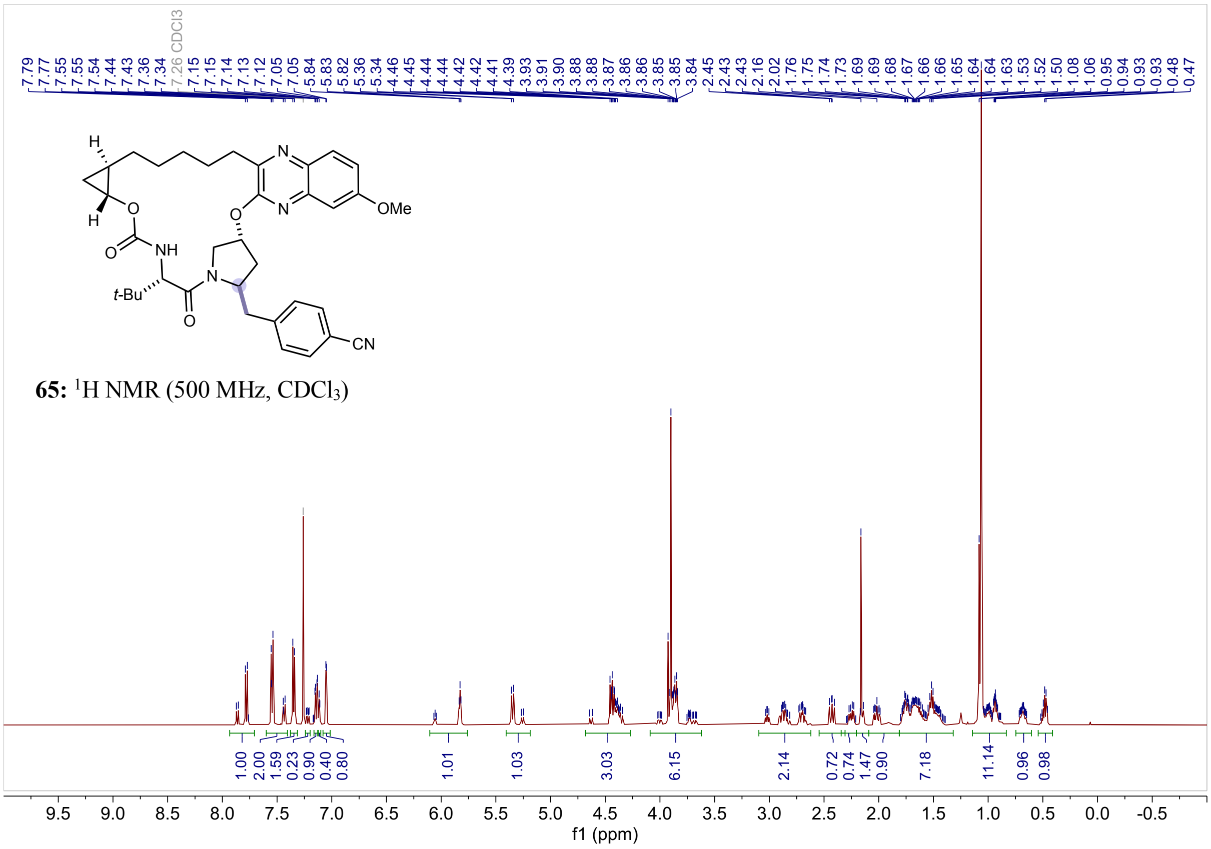

65: ^{1}H NMR (500 MHz, $CDCl_3$)

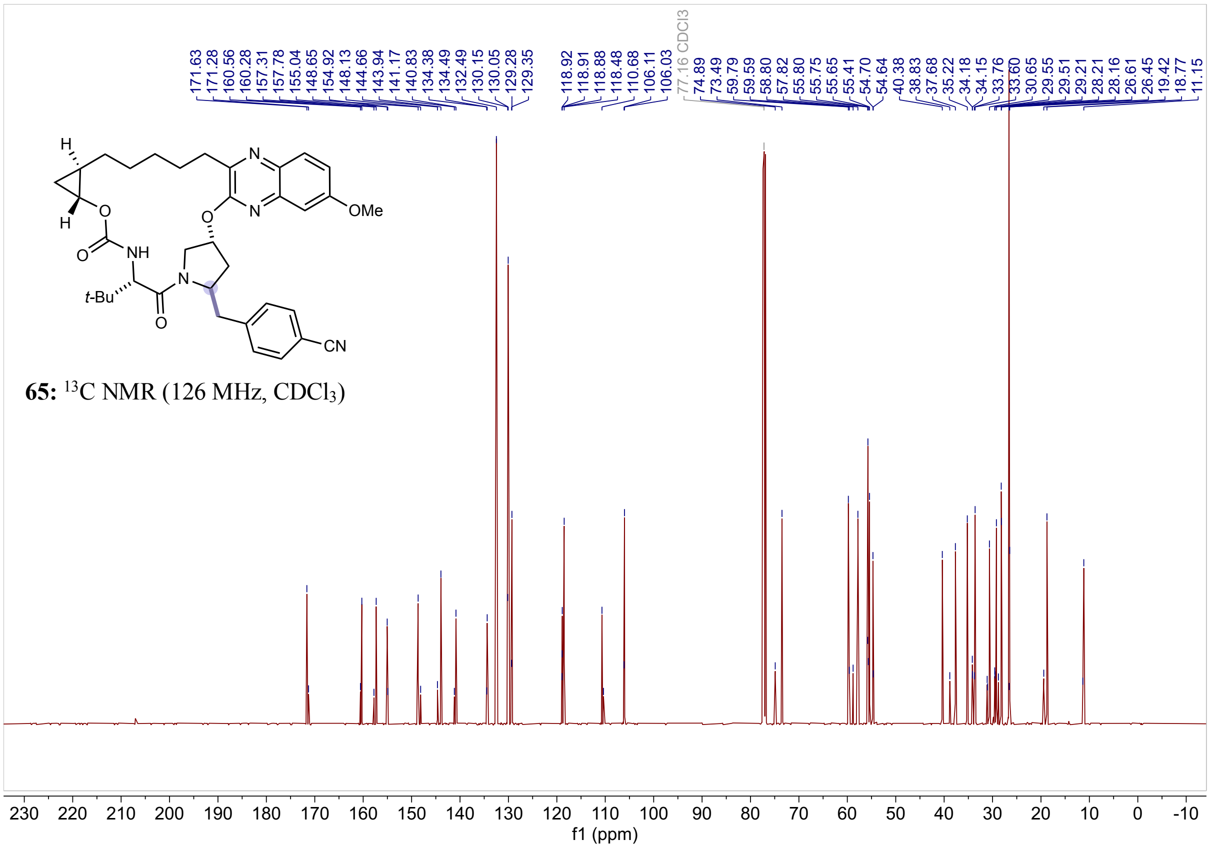

65: ^{13}C NMR (126 MHz, $CDCl_3$)

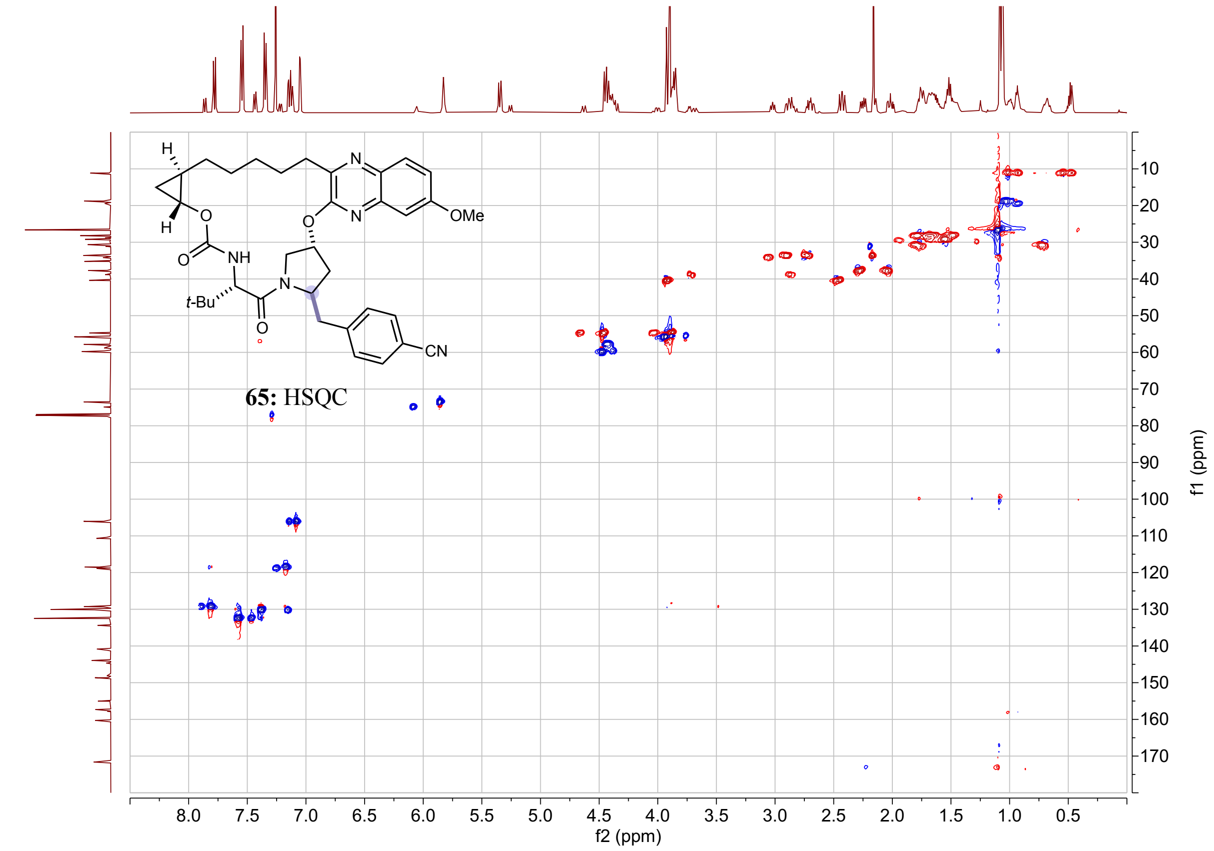
65: HSQC
OMe
NH
t-Bu
CN
f2 (ppm)
f1 (ppm)

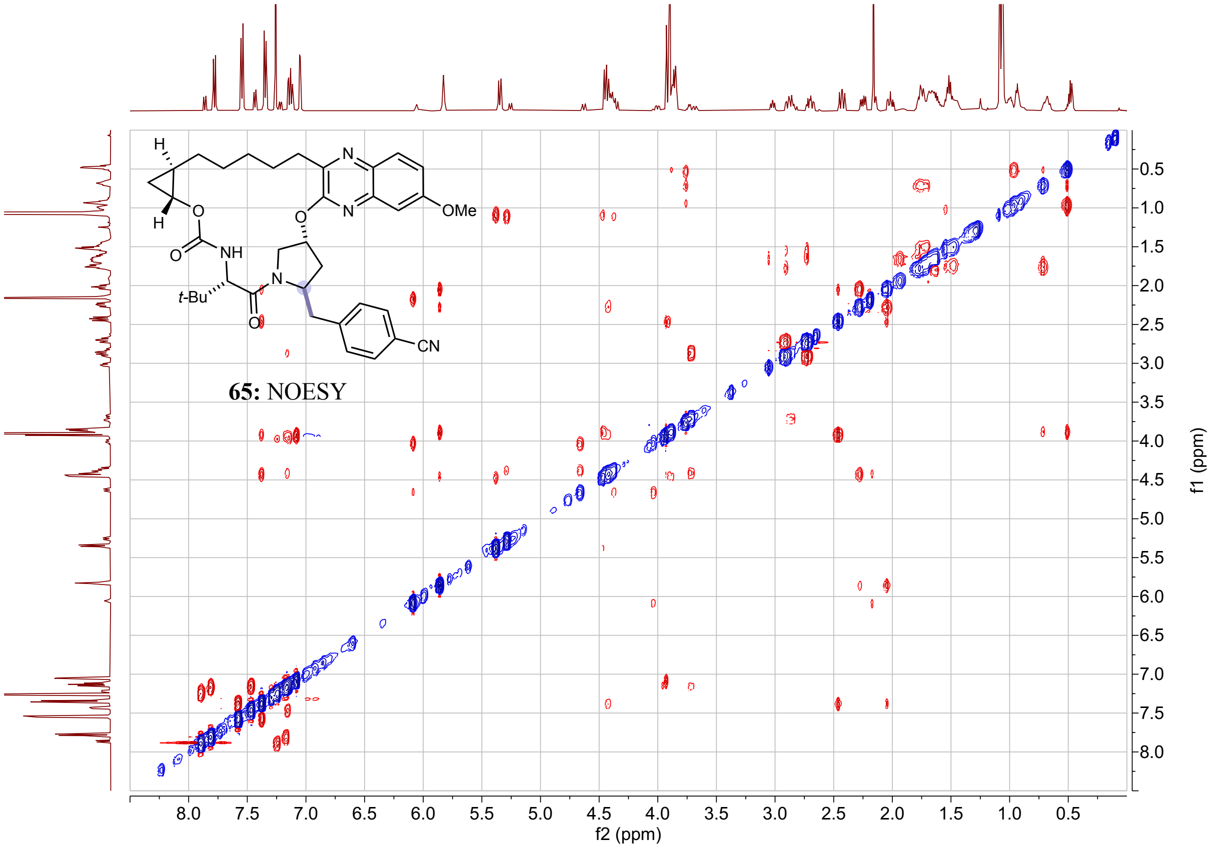
65: NOESY
OMe
NH
t-Bu
CN
f2 (ppm)
f1 (ppm)

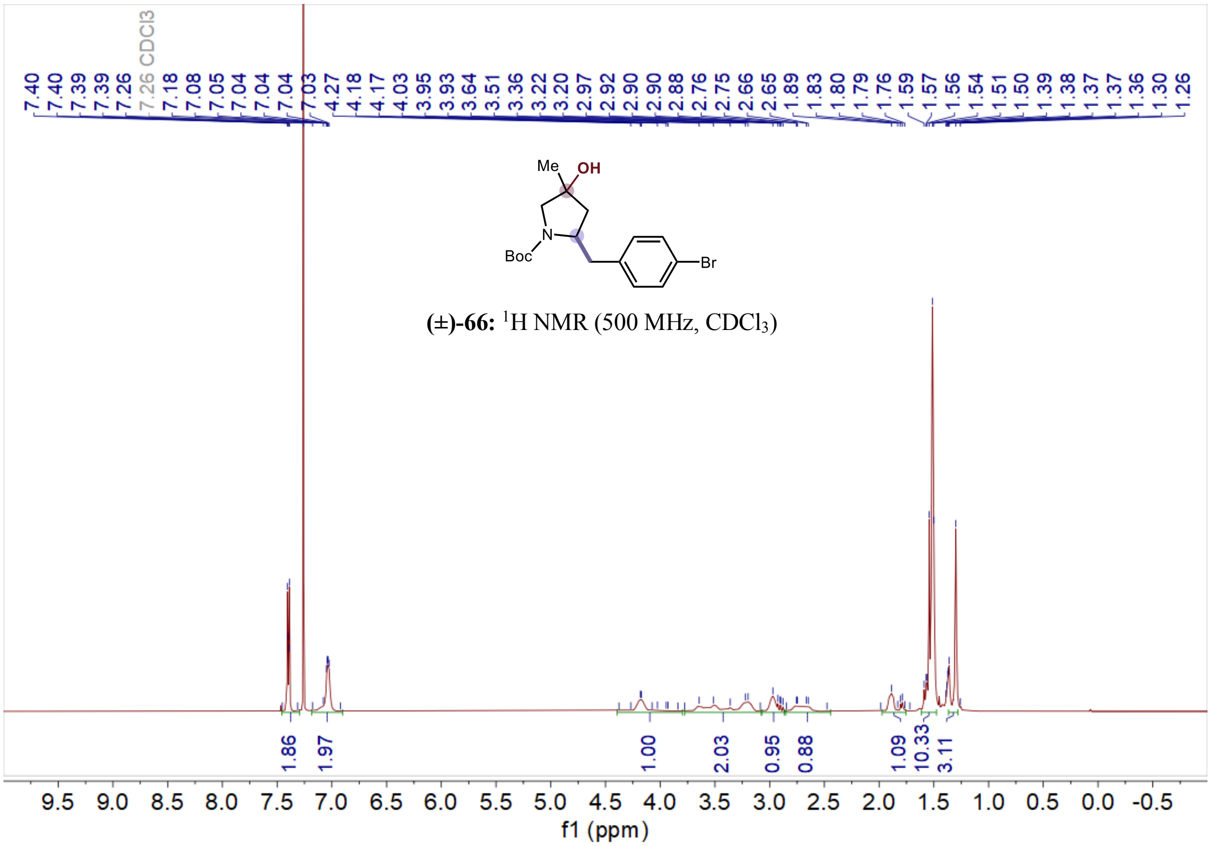
(±)-66: ¹H NMR (500 MHz, CDCl3)
Me
OH
N
Boc
Br
7.40 7.40 7.39 7.39 7.26 7.26 CDCl3 7.18 7.08 7.05 7.04 7.04 7.04 7.03 4.27 4.18 4.17 4.03 3.95 3.93 3.64 3.51 3.36 3.22 3.20 2.97 2.92 2.90 2.90 2.88 2.76 2.75 2.66 2.65 1.89 1.83 1.80 1.79 1.76 1.59 1.57 1.56 1.54 1.51 1.50 1.39 1.38 1.37 1.37 1.36 1.30 1.26
1.86 1.97 1.00 2.03 0.95 0.88 1.09 10.33 3.11
f1 (ppm)

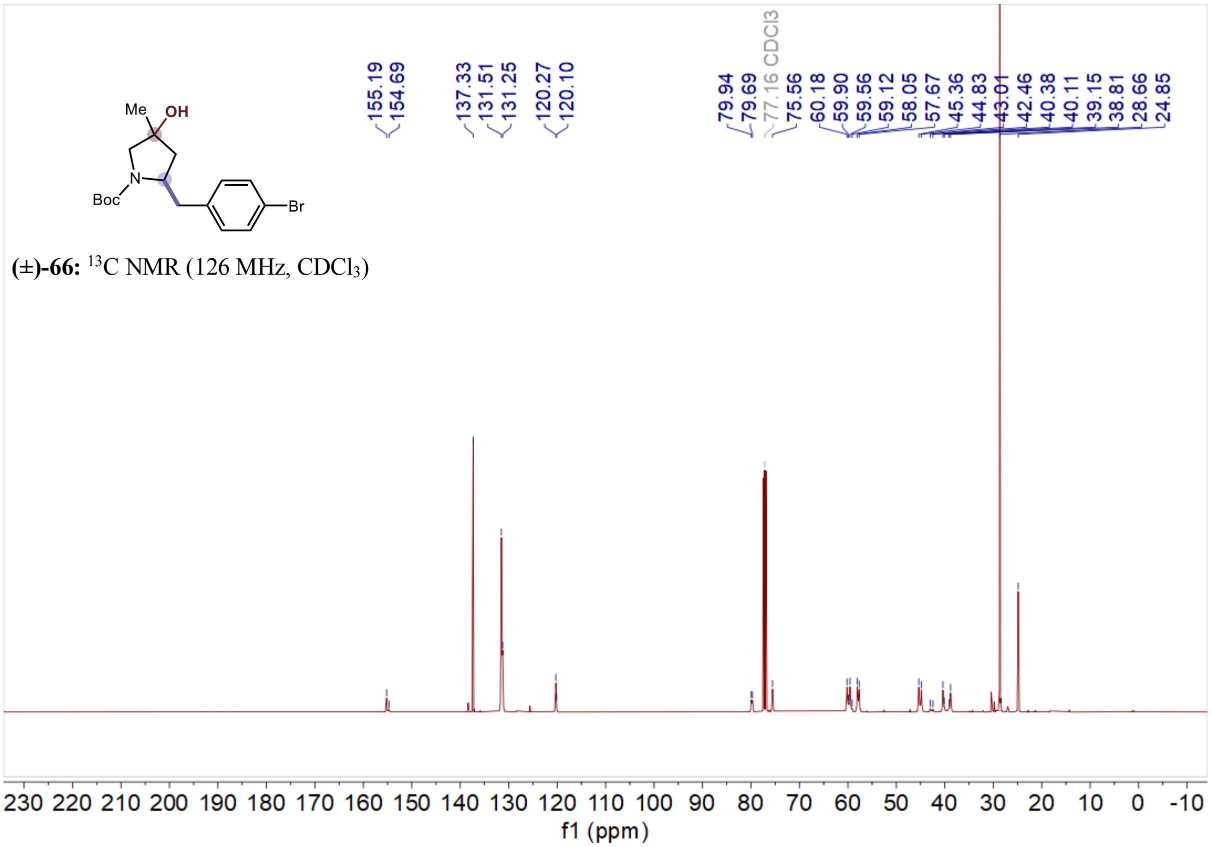
(±)-66: ¹³C NMR (126 MHz, CDCl3)
Me
OH
N
Boc
Br
155.19 154.69 137.33 131.51 131.25 120.27 120.10 79.94 79.69 77.16 CDCl3 75.56 60.18 59.90 59.56 59.12 58.05 57.67 45.36 44.83 43.01 42.46 40.38 40.11 39.15 38.81 28.66 24.85
f1 (ppm)

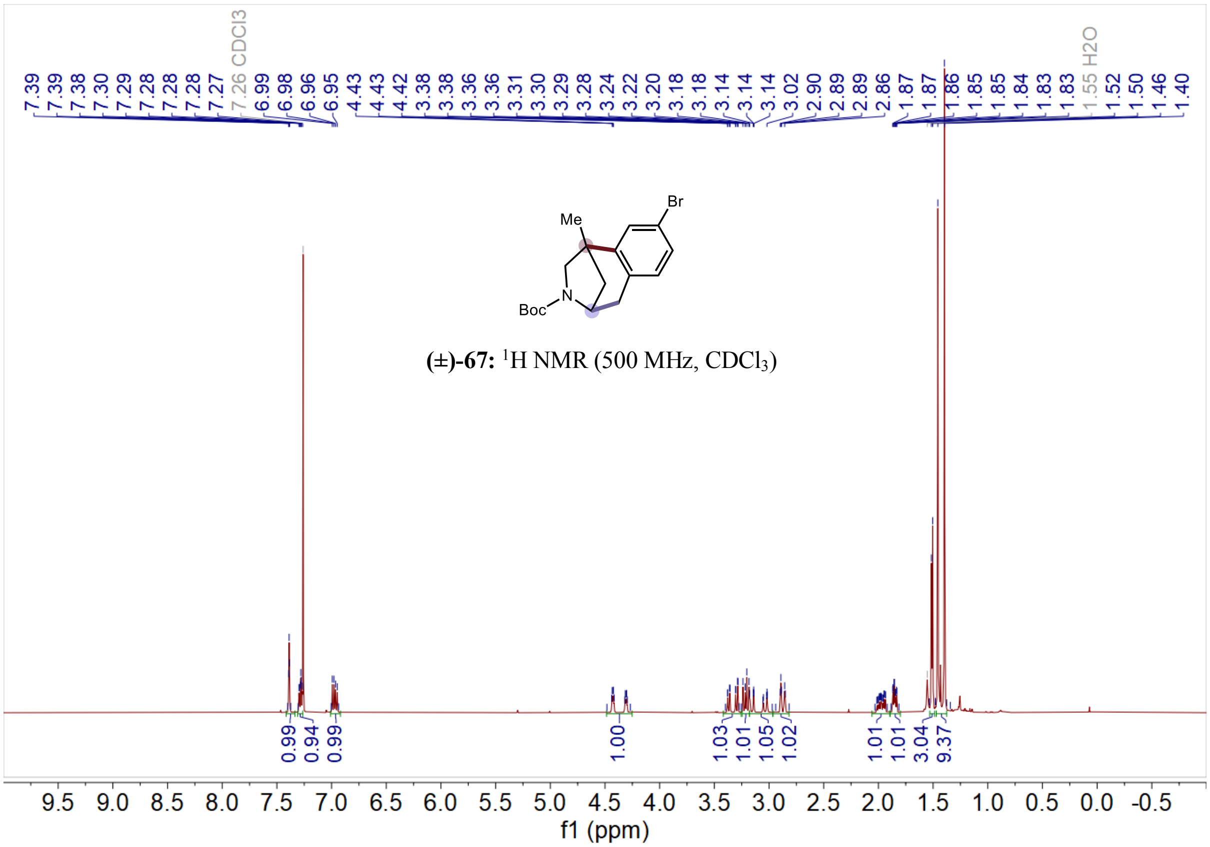

(±)-67: ^{1}H NMR (500 MHz, $CDCl_3$)

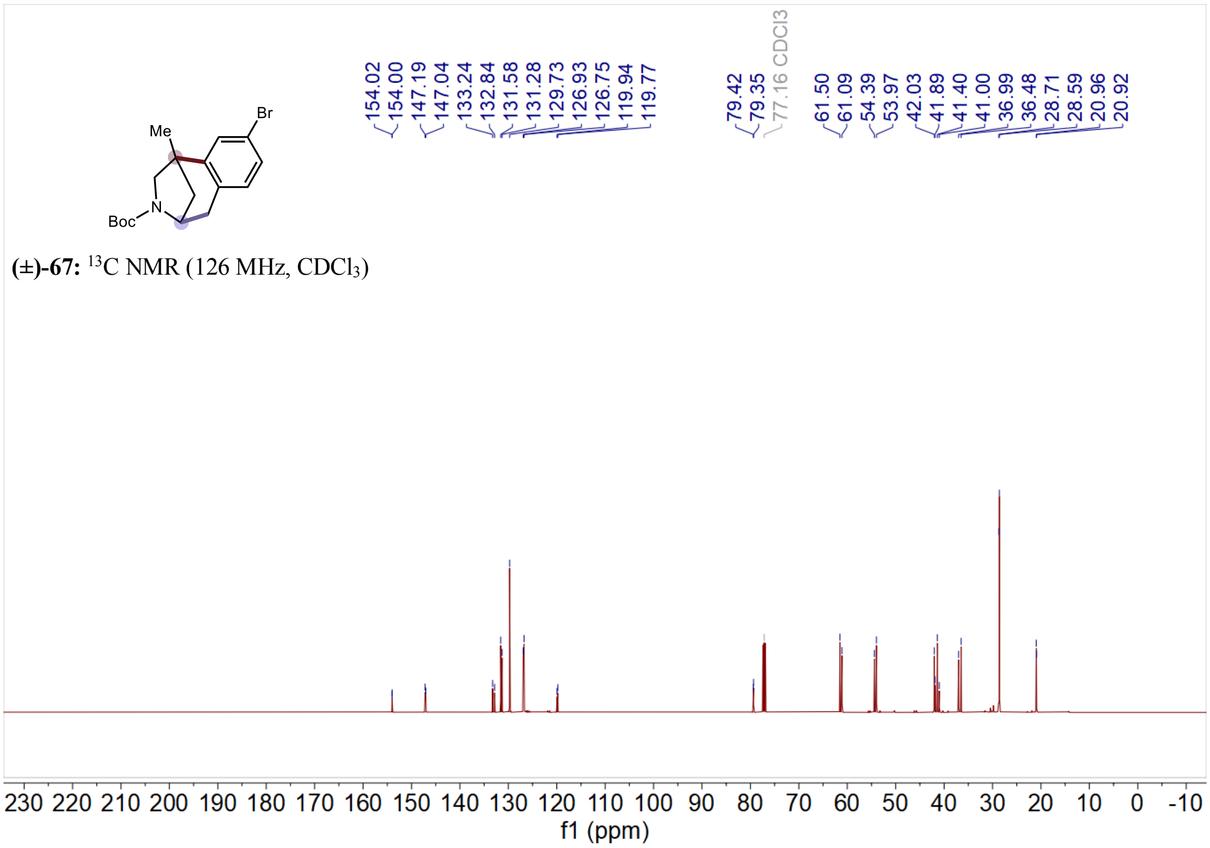

(±)-67: ^{13}C NMR (126 MHz, $CDCl_3$)

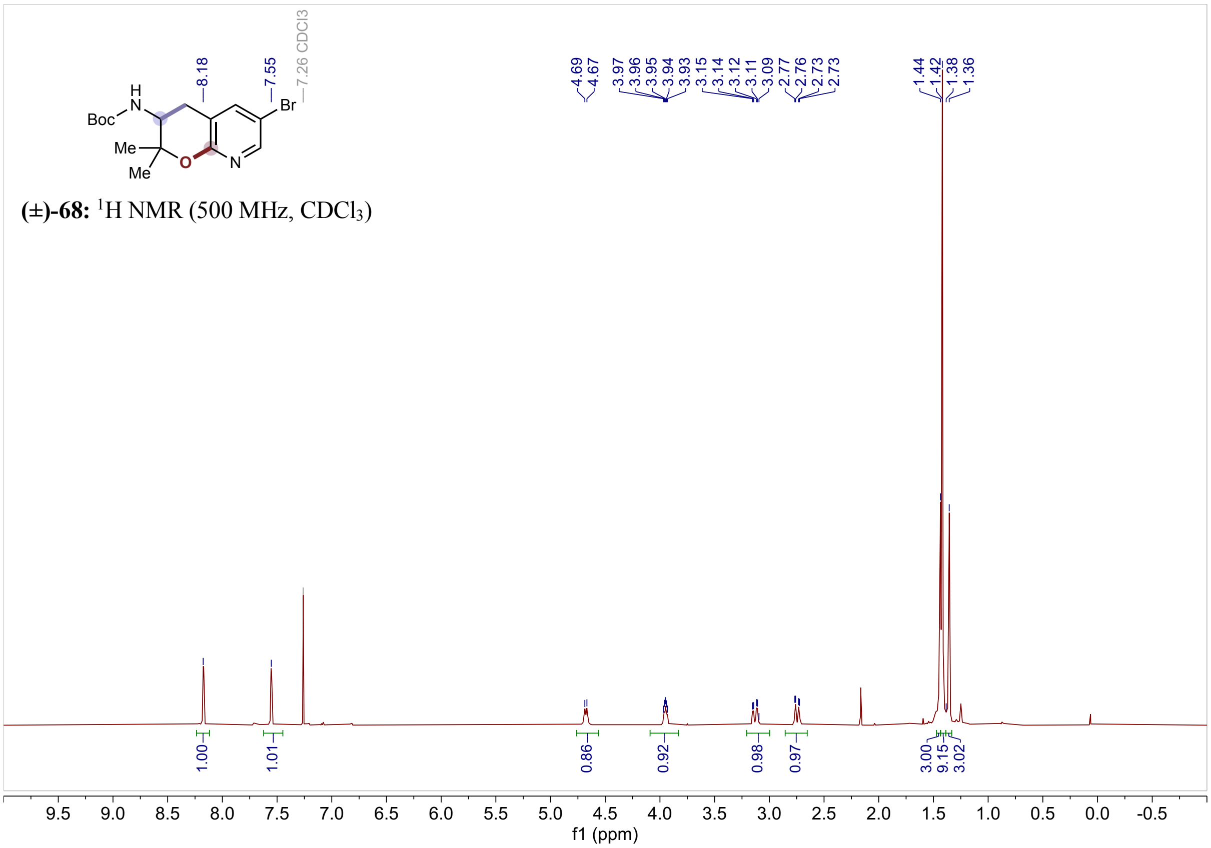
(±)-68: 1H NMR (500 MHz, CDCl3)
8.18
7.55
7.26 CDCl3
4.69
4.67
3.97
3.96
3.95
3.94
3.93
3.15
3.14
3.12
3.11
3.09
2.77
2.76
2.73
2.73
1.44
1.42
1.38
1.36
1.00
1.01
0.86
0.92
0.98
0.97
3.00
9.15
3.02
f1 (ppm)

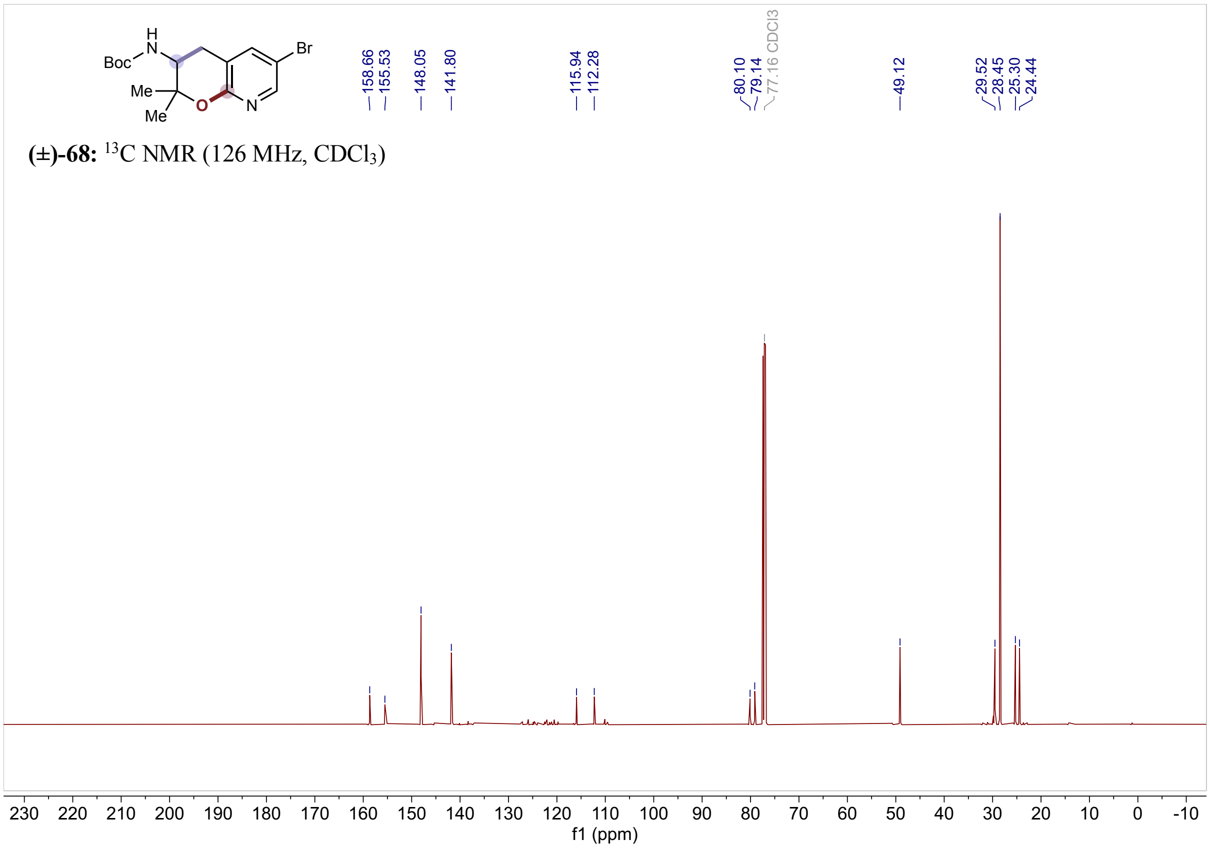
(±)-68: 13C NMR (126 MHz, CDCl3)
158.66
155.53
148.05
141.80
115.94
112.28
80.10
79.14
77.16 CDCl3
49.12
29.52
28.45
25.30
24.44
f1 (ppm)

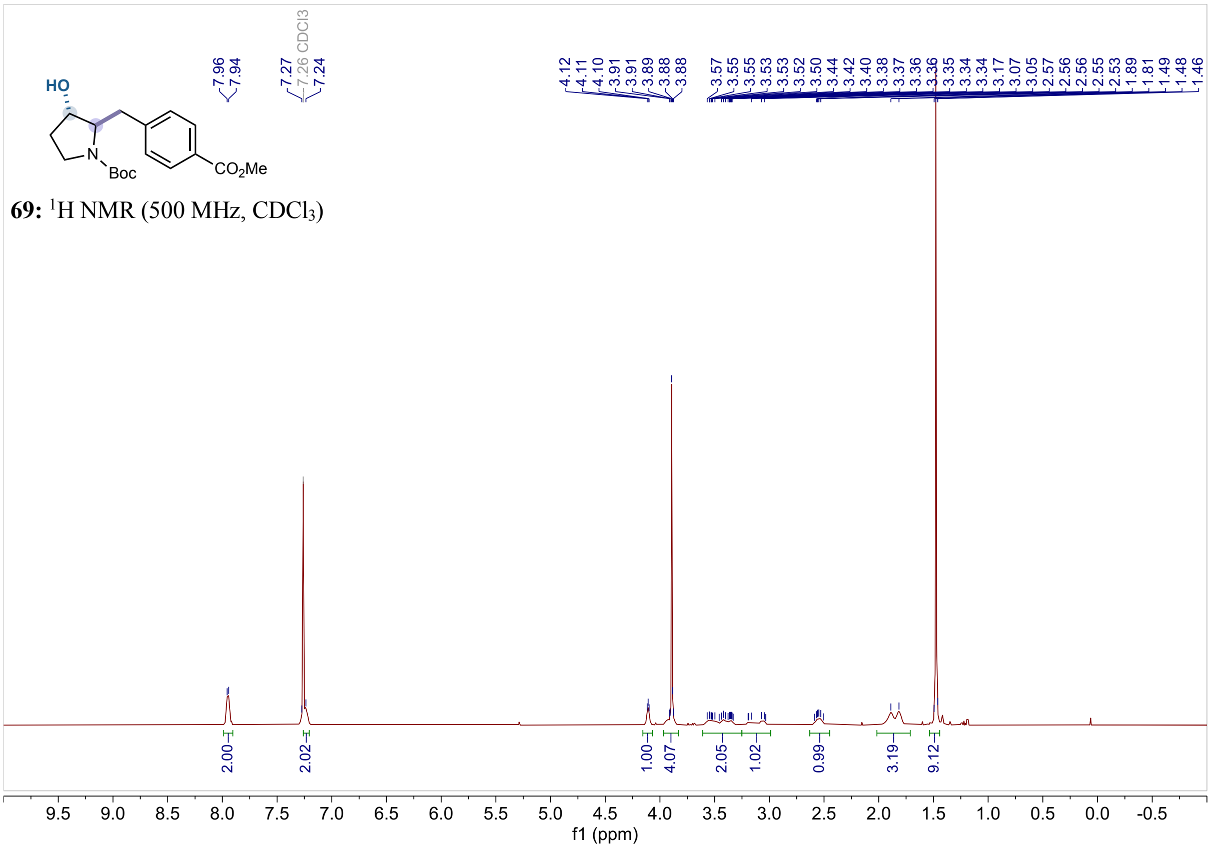

HO
N
Boc
CO2Me
69: 1H NMR (500 MHz, CDCl3)
7.96
7.94
7.27
7.26 CDCl3
7.24
4.12
4.11
4.10
3.91
3.91
3.89
3.88
3.88
3.57
3.55
3.55
3.53
3.53
3.52
3.50
3.44
3.42
3.40
3.38
3.37
3.36
3.36
3.35
3.34
3.34
3.17
3.07
3.05
2.57
2.56
2.56
2.55
2.53
1.89
1.81
1.49
1.48
1.46
2.00
2.02
1.00
4.07
2.05
1.02
0.99
3.19
9.12
9.5 9.0 8.5 8.0 7.5 7.0 6.5 6.0 5.5 5.0 4.5 4.0 3.5 3.0 2.5 2.0 1.5 1.0 0.5 0.0 -0.5
f1 (ppm)

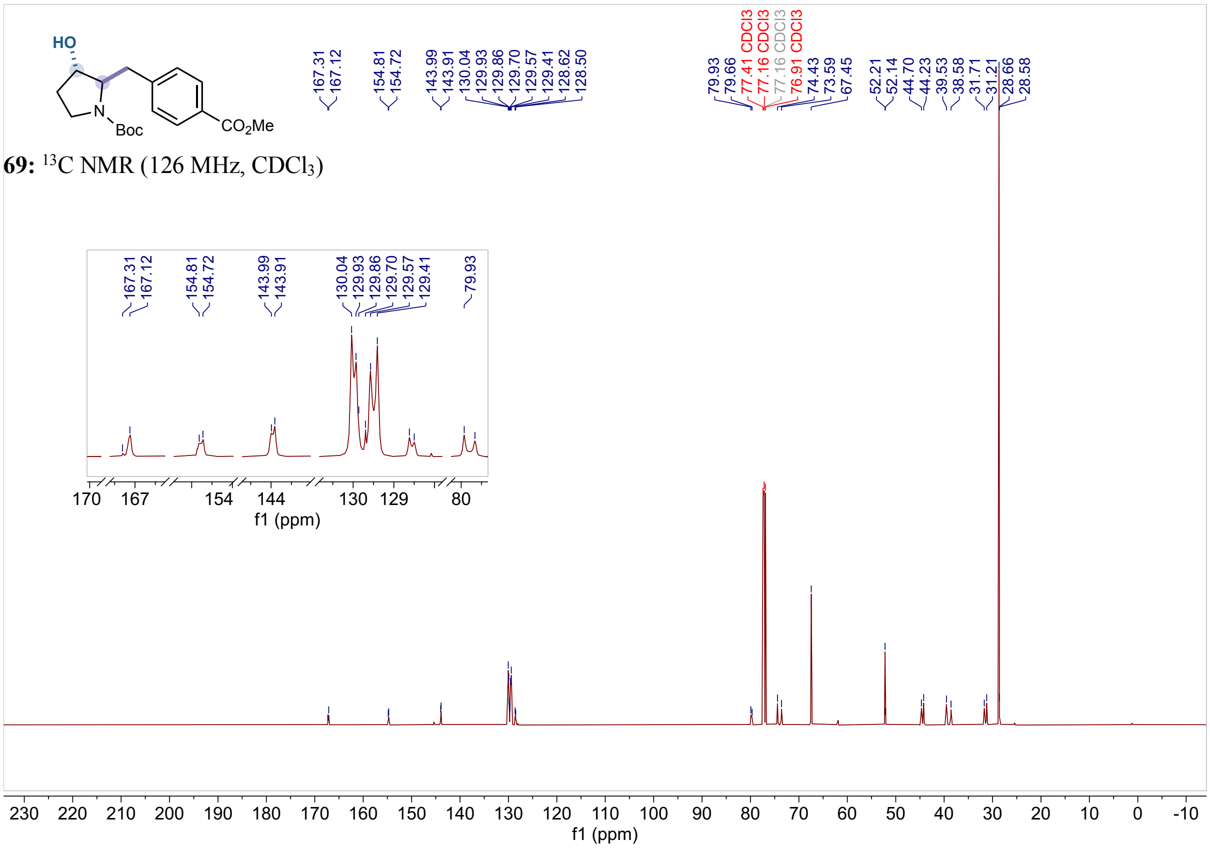

HO
N
Boc
CO2Me
69: 13C NMR (126 MHz, CDCl3)
167.31
167.12
154.81
154.72
143.99
143.91
130.04
129.93
129.86
129.70
129.57
129.41
128.62
128.50
79.93
79.66
77.41 CDCl3
77.16 CDCl3
77.16 CDCl3
76.91 CDCl3
74.43
73.59
67.45
52.21
52.14
44.70
44.23
39.53
38.58
31.71
31.21
28.66
28.58
170 167 154 144 130 129 80
f1 (ppm)
230 220 210 200 190 180 170 160 150 140 130 120 110 100 90 80 70 60 50 40 30 20 10 0 -10
f1 (ppm)

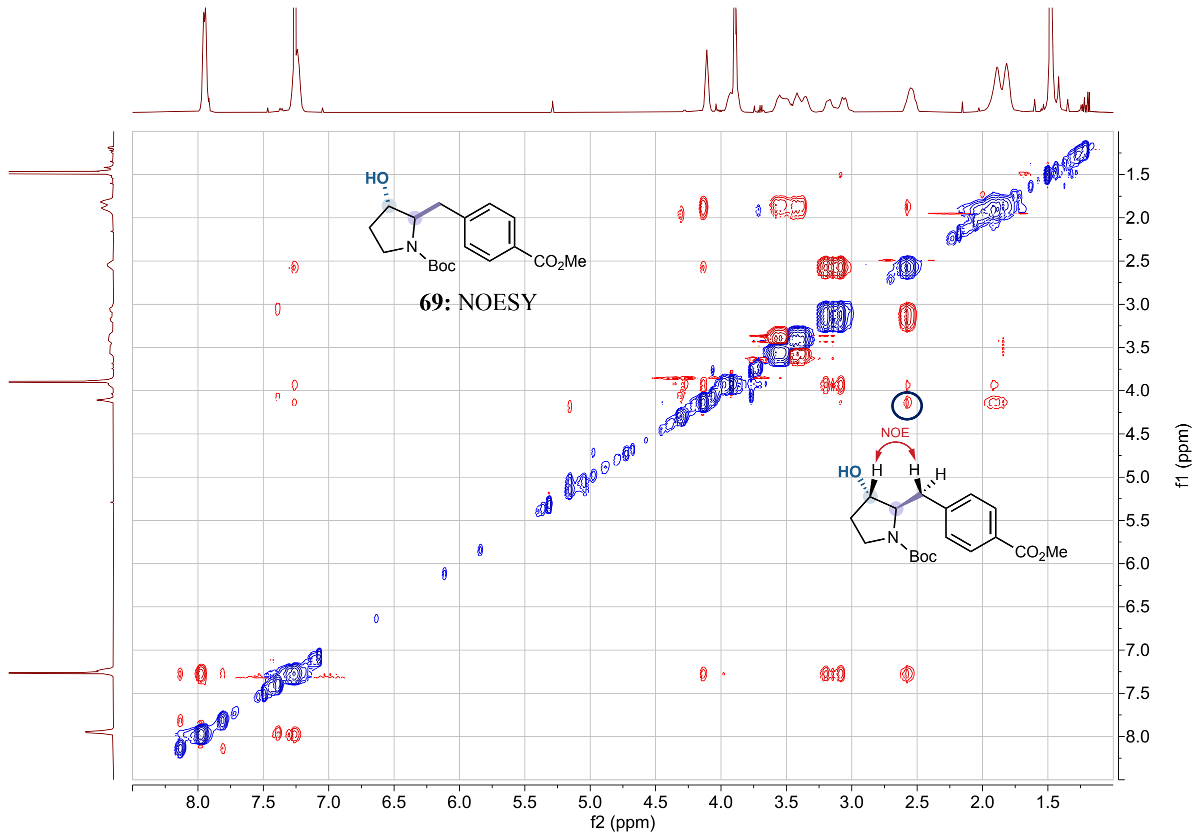
HO
N
Boc
CO2Me
69: NOESY
NOE
HO H
H H
N
Boc
CO2Me
f1 (ppm)
f2 (ppm)

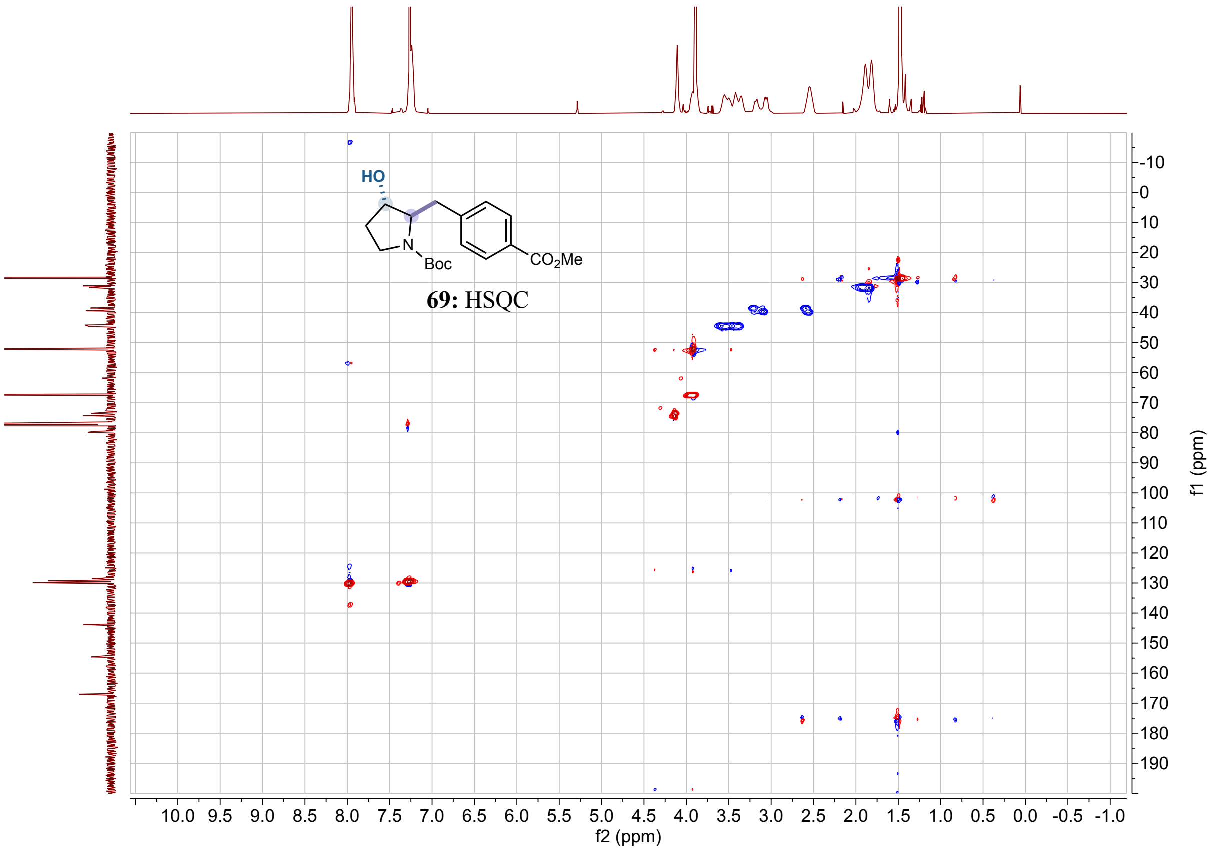
HO
N
Boc
CO2Me
69: HSQC
f1 (ppm)
f2 (ppm)

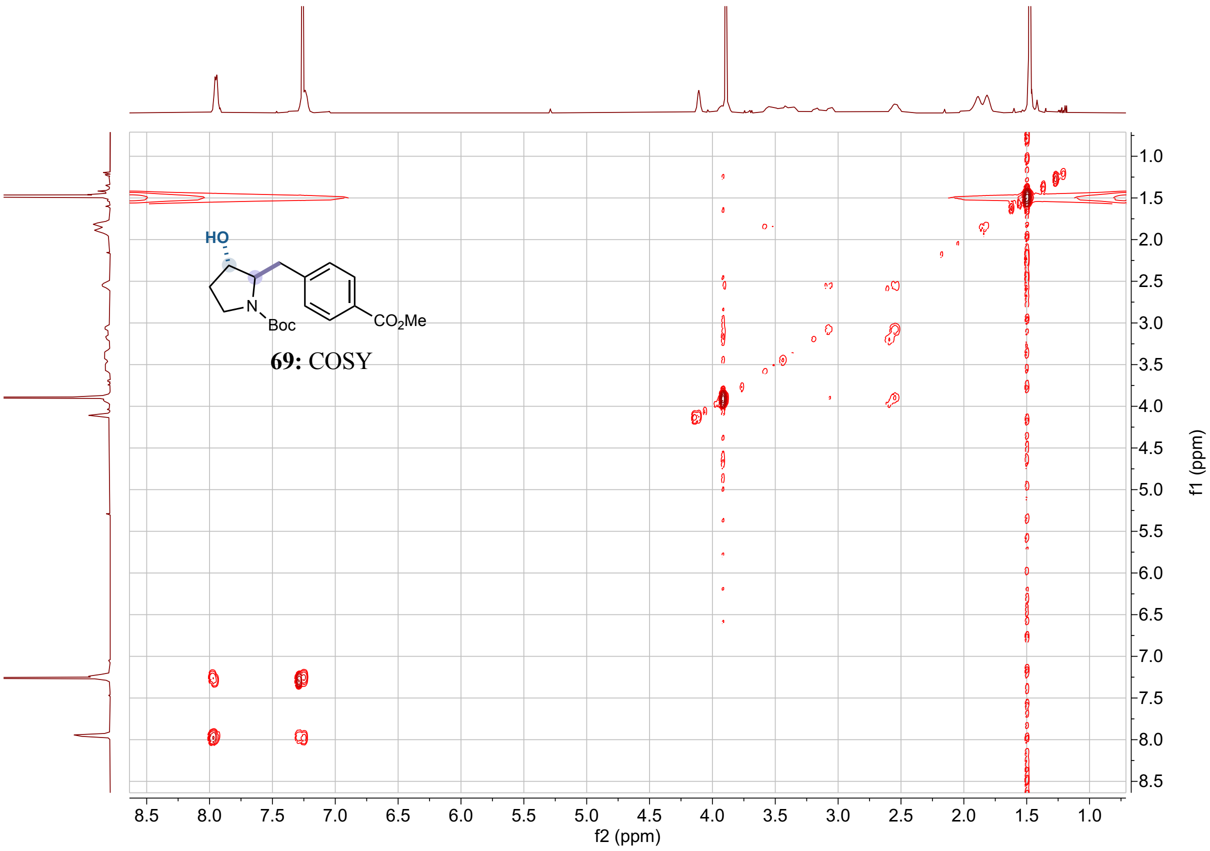

69: COSY

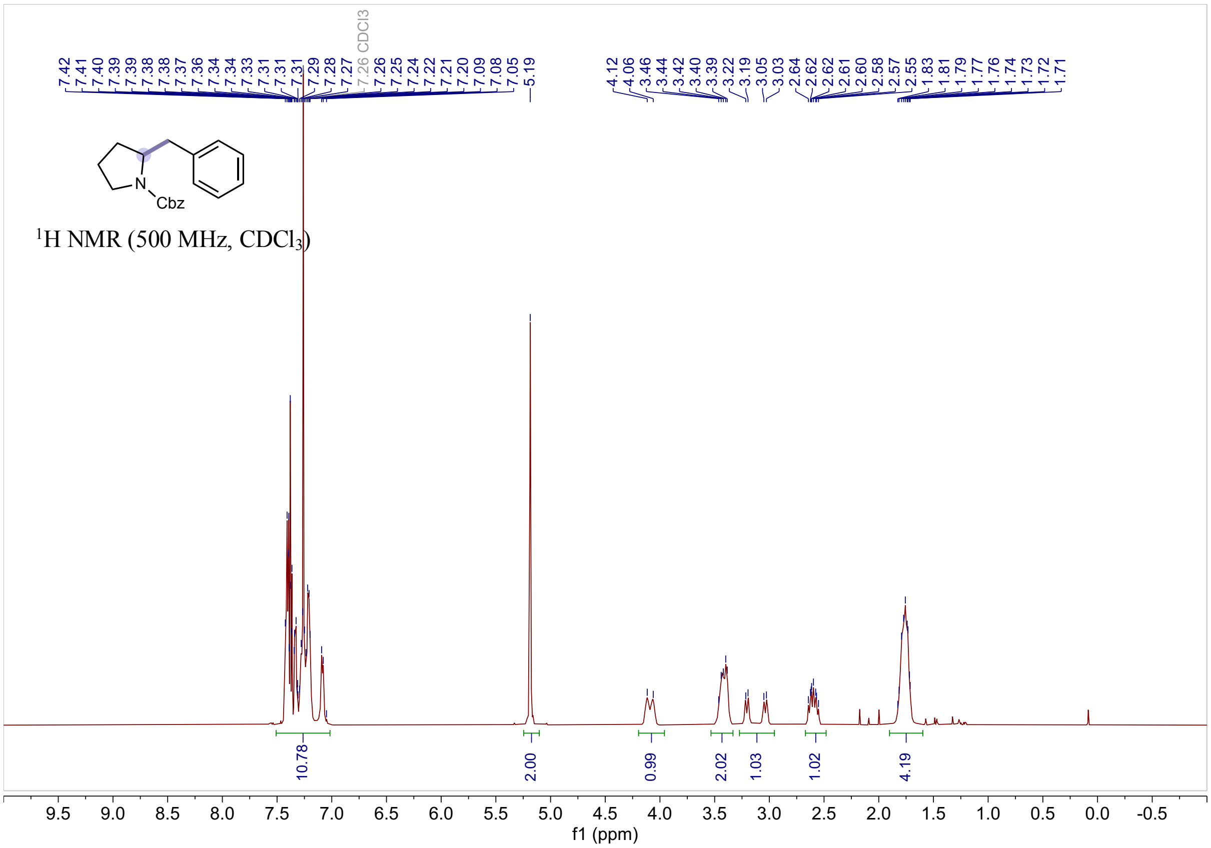
Cbz
^{1}H NMR (500 MHz, CDCl$_3$)
7.26 CDCl3
f1 (ppm)

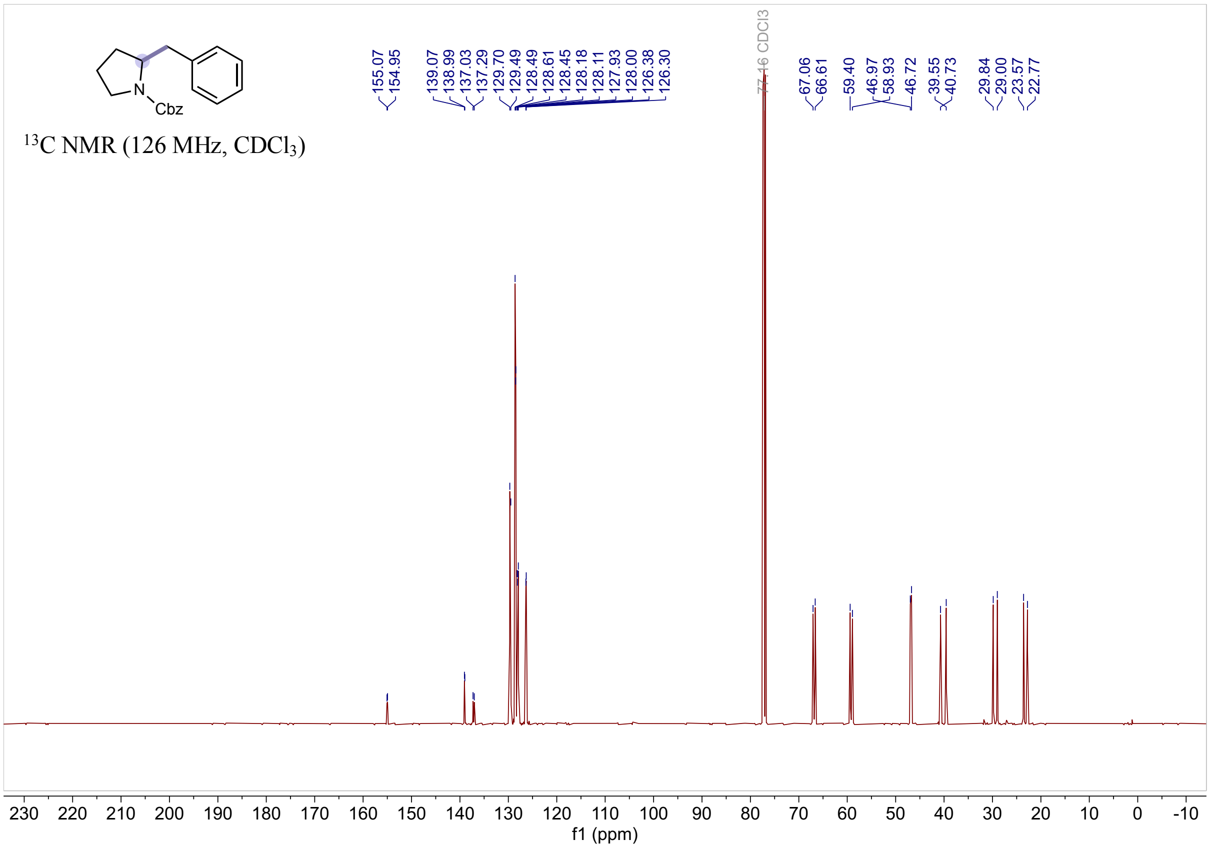
Cbz
^{13}C NMR (126 MHz, CDCl$_3$)
77.16 CDCl3
f1 (ppm)

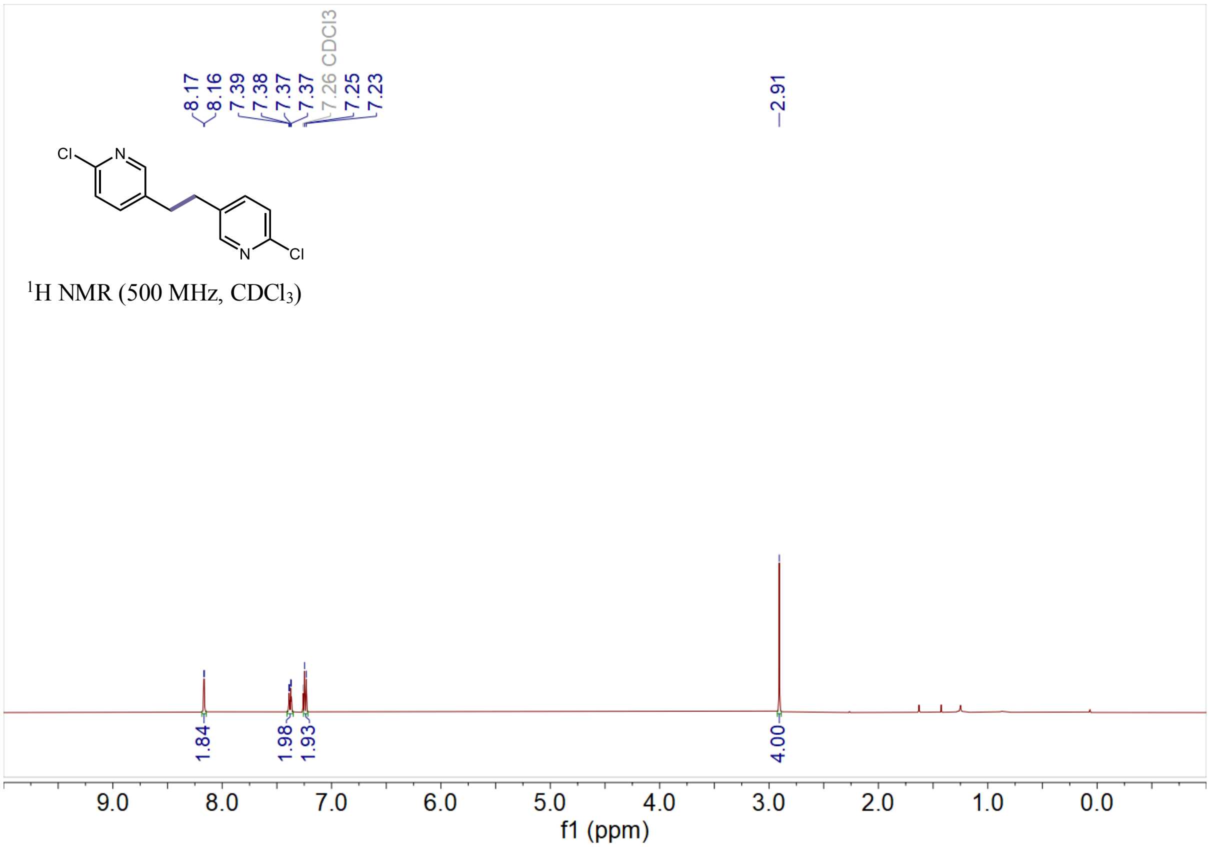

8.17
8.16
7.39
7.38
7.37
7.37
7.26 CDCl3
7.25
7.23
2.91
Cl
N
N
Cl
^{1}H NMR (500 MHz, CDCl$_3$)
1.84
1.98
1.93
4.00
9.0 8.0 7.0 6.0 5.0 4.0 3.0 2.0 1.0 0.0
f1 (ppm)

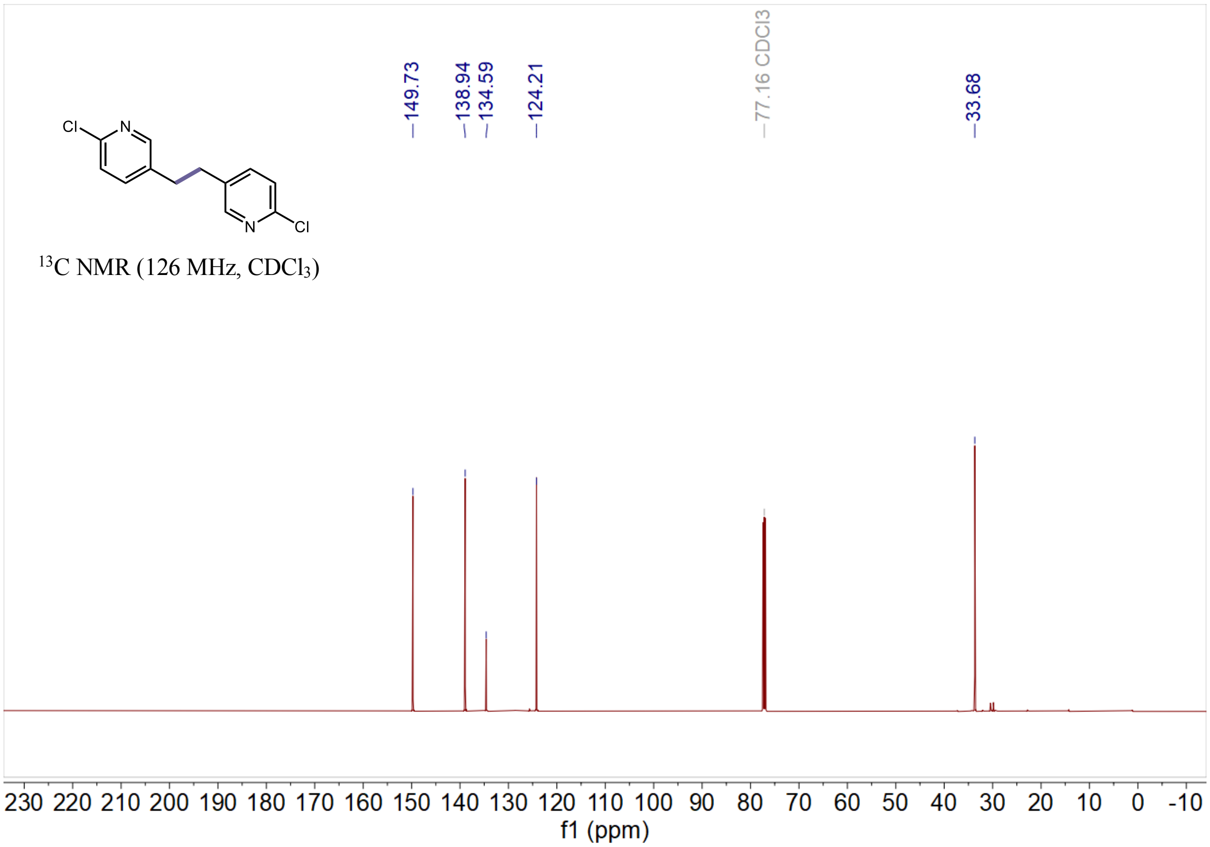

149.73
138.94
134.59
124.21
77.16 CDCl3
33.68
Cl
N
N
Cl
^{13}C NMR (126 MHz, CDCl$_3$)
230 220 210 200 190 180 170 160 150 140 130 120 110 100 90 80 70 60 50 40 30 20 10 0 -10
f1 (ppm)

10. Bibliography

- Aguilar Troyano, F.J.; Ballaschk, F.; Jaschinski, M.; Özkaya, Y.; Gómez-Suárez, A. *Chem. Eur. J.* **2019**, *25*, 14054.
- Basch, C.H; Liao, J.; Xu, J.; Piane, J.J.; Watson, M.P. *J. Am. Chem. Soc.* **2017** *139*, 5313-5316. DOI: 10.1021/jacs.7b02389.
- Bolt Biotherapeutics, Inc. Anti-HER2 Immunoconjugates, and Uses Thereof. WO Patent 2022/125904, 2022.
- Bristol-Myers Squibb Company. Novel 2-Substituted Cyclic Amines as Calcium Sensing Receptor Modulators. WO Patent 2004/069793, 2004.
- Daiichi Sankyo Co., Ltd. Imidazothiazole Derivatives Having a 4,7-Diazaspiro[2.5]octane Ring Structure. EP Patent 2298778, 2011.
- Dong, Z.; MacMillan, D.W.C. Metallaphotoredox-enabled deoxygenative arylation of alcohols. *Nature* **2021**, *598*, 451–456. DOI: 10.1038/s41586-021-03920-6.
- Eli Lilly and Company. Selective
- Beta 3 Adrenergic Agonists. U.S. Patent 5,786,356, 1998.
- Gould, C. A.; Pace, A. L.; MacMillan, D. W. C. Rapid and Modular Access to Quaternary Carbons from Tertiary Alcohols via Bimolecular Homolytic Substitution. *J. Am. Chem. Soc.* **2023,** *145*, 16330–16336. DOI: 10.1021/jacs.3c05405.
- Krasovskiy, A.; Kopp, F.; Knochel, P.; Soluble Lanthanide Salts ($LnCl_3 \cdot 2\ LiCl$) for the Improved Addition of Organomagnesium Reagents to Carbonyl Compounds. *Angew. Chem. Intl. Ed.* **2006** *45*, 497-500. DOI: 10.1002/anie.200502485.
- Liao, J; Basch, C.H.; Hoerrner, M.E.; Talley, M.R.; Boscoe, B.P.; Tucker, J.W.; Garnsey, M.R.; Watson, M.P. *Org. Lett.* **2019** *21*, 2941-2946. DOI: 10.1021/acs.orglett.9b01014.
- Massah, A.R; Ross, A.J.; Jackson, R.F.W. In Situ trapping of Boc-2-pyrrolidinylmethylzinc Iodide with Aryl Iodides: Direct Synthesis of 2-Benzylpyrrolidines. *J. Org. Chem.* **2010,** *75* (23), 8275–8278. DOI: 10.1021/jo101503p.
- Pace A.L.; Xu, F.; Liu W.; Lavagnino, M.N.; MacMillan, D.W.C. *J. Am. Chem Soc.* **2024** *146*, 32925-32932. DOI: 10.1021/jacs.4c14942.

- Shao, C.; Wang, X.; Zhang, Q.; Luo, S.; Zhao, J.; Hu, Y. Acid–Base Jointly Promoted Copper(I)-Catalyzed Azide–Alkyne Cycloaddition. *J. Org. Chem.* **2011,** *76* (16), 6832–6836. DOI: 10.1021/jo200869a.
